# Genotoxicity and molecular response of silver nanoparticle (NP)-based hydrogel

**DOI:** 10.1186/1477-3155-10-16

**Published:** 2012-05-01

**Authors:** Liming Xu, Xuefei Li, Taro Takemura, Nobutaka Hanagata, Gang Wu, Laisheng Lee Chou

**Affiliations:** 1 Institute for Medical Devices Control, National Institutes for Food and Drug Control (NIFDC), No. 2 Temple of Heaven, Beijing, 100050, China; 2 Baotou Medical College, Inner Mongolia University of Science & Technology, Baotou, 014010, China; 3 Interdisciplinary Laboratory for Nanoscale Science and Technology, National Institute for Materials Science, 1-2-1 Sengen, Tsukuba, Ibaraki, 305-0047, Japan; 4 Goldman School of Dental Medicine, Boston University, 801 Albany Street, Suite 200, Boston, MA, 02118-2392, USA

**Keywords:** Silver nanoparticle-based hydrogel (silver-NP-hydrogel), Genotoxicity, Global gene expression, DNA damage, Apoptosis and mitosis pathway, JAK-STAT signal transduction pathway

## Abstract

**Background:**

Since silver-nanoparticles (NPs) possess an antibacterial activity, they were commonly used in medical products and devices, food storage materials, cosmetics, various health care products, and industrial products. Various silver-NP based medical devices are available for clinical uses, such as silver-NP based dressing and silver-NP based hydrogel (silver-NP-hydrogel) for medical applications. Although the previous data have suggested silver-NPs induced toxicity in vivo and in vitro, there is lack information about the mechanisms of biological response and potential toxicity of silver-NP-hydrogel.

**Methods:**

In this study, the genotoxicity of silver-NP-hydrogel was assayed using cytokinesis-block micronucleus (CBMN). The molecular response was studied using DNA microarray and GO pathway analysis.

**Results and discussion:**

The results of global gene expression analysis in HeLa cells showed that thousands of genes were up- or down-regulated at 48 h of silver-NP-hydrogel exposure. Further GO pathway analysis suggested that fourteen theoretical activating signaling pathways were attributed to up-regulated genes; and three signal pathways were attributed to down-regulated genes. It was discussed that the cells protect themselves against silver NP-mediated toxicity through up-regulating metallothionein genes and anti-oxidative stress genes. The changes in DNA damage, apoptosis and mitosis pathway were closely related to silver-NP-induced cytotoxicity and chromosome damage. The down-regulation of CDC14A via mitosis pathway might play a role in potential genotoxicity induced by silver-NPs.

**Conclusions:**

The silver-NP-hydrogel induced micronuclei formation in cellular level and broad spectrum molecular responses in gene expression level. The results of signal pathway analysis suggested that the balances between anti-ROS response and DNA damage, chromosome instability, mitosis inhibition might play important roles in silver-NP induced toxicity. The inflammatory factors were likely involved in silver-NP-hydrogel complex-induced toxic effects via JAK-STAT signal transduction pathway and immune response pathway. These biological responses eventually decide the future of the cells, survival or apoptosis.

## Background

Since the 2000s with the development of nanotechnology, various nanomaterials have been commercially used in a wide range of areas. Due to their antibacterial activity, silver-nanoparticles (NPs) are used commonly in medical products and devices, food storage materials, cosmetics, various health care products, and industrial products. In medical applications, silver-NPs have been used for silver-based dressings [1,2], silver-coated catheters [3,4], silver-based hydrogel [5-7]. Silver-NP-hydrogel composites are composed of silver-NP and hydrogel which are used as carrier for silver particles. Most studies focused on manufacturing methods and antibacterial activity of silver-NP-hydrogel composites [5-7].

In recent years, increasing data demonstrated that silver-NPs could induce toxicity in vivo under a variety of exposure conditions including inhalation [8-10], orally [11,12] and via hypodermic injection [13]. Some in vitro studies revealed that silver-NPs could cause strong cytotoxicity in a broad spectrum of cells [14-25], such as germline stem cells [15], messenchymal stem cells (hMSCs) [16-18], BRL 3A rat liver cells [19], NIH3T3 cells [20], HepG2 human hepatoma cells [21], normal human lung fibroblasts (IMR-90), human glioblastoma cells (U251) [22,23], human normal bronchial epithelial (BEAS-2B) cells [24] and HeLa cells [25]. Many studies also reported that silver-NPs induced potential genotoxicity in several types of cells [21-24,26]. With the concerns about the safety and clinical risks associated with silver-NP-based medical products, however, a little is know about the molecular mechanism of silver-NP induced toxicity.

Metal ions including silver act as catalysts and can produce reactive oxygen species (ROS) in the presence of oxygen, which is considered to be a mechanism of toxicity and genotoxicity for metal nanomaterials. Acting as signal molecules, ROS, can promote cell cycle progression and induce oxidative DNA damage [19,27-29]. CBMN assay [30] is sensitive to ROS-mediated DNA damage, making it suitable for assessing the genotoxicity potentially induced by nanomaterials. Therefore, CBMN assay was selected to assess genotoxicity of silver-NP-hydrogel in this study.

Technique of microarray provides a way of studying biocompatibility of biomaterials at molecular level [31]. The global gene expression analysis using the microarray technique could gain profiling information of nanomaterial-cell interactions [25,32,33].

In this study, in vitro genotoxicity and molecular responses of silver-NP-hydrogel were assessed by CBMN assay and global gene expression analysis. The results provided scientific evidence for understanding the biosafety and potential clinical risk of silver-NP-based products.

## Results

### Genotoxicity

To know whether silver-NP-hydrogel has potential genetoxicity, a CBMN assay was conducted for assessing chromosome damage by silver-NP-hydrogel in HeLa cell cultures. The results were presented as the frequency of micronucleation per 1000 BNCs (Table 1). The MMC treatment (positive control) showed a MNF of 20.6% ± 2.47, showing a significant difference compared to the NaCl solution treatment (negative control), which had a MNF of 2.5% ± 0.79 (*P* < 0.05). It confirmed that the test system worked well. There was a significant increase in the MN frequency at 20 mg/ml (*P* < 0.05), 40 mg/ml (*P* < 0.05) and 60 mg/ml (*P* < 0.05) of silver-NP-hydrogel exposure, this was not observed at the hydrogel treatment alone (*P* = 0.116). These results suggested that the silver-NP-hydrogel induced chromosome damage in HeLa cells.

**Table 1:** The CBMN assay of HeLa cells post-exposed to silver-NP-hydrogel and hydrogel for 24 h

**Test**	**Dose**	**FMN (%)**	**95% Confidence Interval for mean**	
			**Lower Bound**	**Upper Bound**
NC (NaCl sol.)	50 μl	2.5 ± 0.79	5.3	44.7
Hydrogel	60 mg/ml	3.7 ± 0.66	20.7	53.3
Silver-NP Gel	20 mg/ml	7.0 ± 0.82^*^	49.7	90.3
	40 mg/ml	8.67 ± 0.32^*^	78.7	94.7
	60 mg/ml	9.47 ± 0.3^*^	87.6	102.5
PC (MMC)	0.05 μg/ml	20.6 ± 24.7^*^		

Mitomycin C (MMC) was used as a positive control (PC). NaCl solution (NaCl sol.) was used as a negative control (NC). The results were presented as percentage of micronucleation frequency (FMN %) in 1000 binucleation cells. The significance of positive control compared to negative control was identified using *T*-Test. The significance of all test samples compared to negative control was identified using ANOVA and Dunnett tests (2-sided).

^*,^*P* < 0.05. The data indicate the mean ± SD (n = 3).

### Cellular response at molecular levels

**Figure 1:**
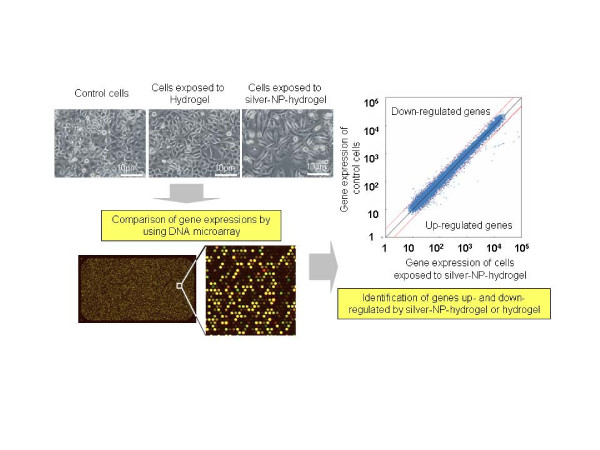
**The graphical abstract of working process for global gene expression analysis.** After exposure to silver-NP-hydrogel and Hydrogel, the treated cells and non-treatment control cells were harvested. Gene expression profiling was analysis by DNA microarray technique. The differential expressed genes were identified by comparing the gene expression levels in treated cells with that in the control cells without treatment.

To assess the cellular response induced by silver-NP-hydrogel exposure at the molecular level and the mechanisms of toxic effects, global gene expression and GO pathway analysis was performed using the DNA microarray technique. The graphical abstract of working process for global gene expression analysis was shown in Figure 1.

### The morphological changes of cells

**Figure 2:**
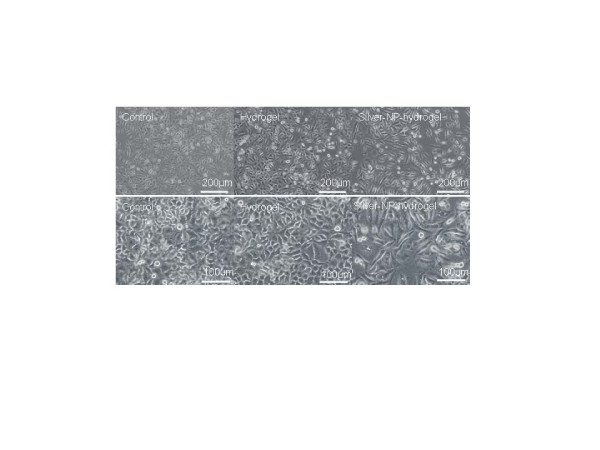
**Cell morphological changes.** The cells were post-exposed with silver-NP-hydrogel (40 mg/ml) and Hydrogel (40 mg/ml) as the study groups and with no treatment as a control for 24 h (up-panel, X 200) and 48 h (bottom-panel, X 400). The changes of cell morphology were visualized by light microscopy.

After exposing cells to 40 mg/ml silver-NP-hydrogel (contained 15.2 μg of silver-NPs) for 24 h and 48 h, the cells lost their normal epithelial cell morphology, becoming longer, and swelled. In contrast, cells exposed to hydrogel (without silver-NPs) did not show significant difference compared to the non-treatment control (Figure 2).

### Gene expression profiling

According to the defined filtering criteria as described in “Materials and methods”, the differentially expressed genes in both silver-NP-hydrogel and hydrogel alone groups, including up-regulated and down-regulated genes, are shown in Additional files 1, 2, 3, 4, 5, 6, 7 and 8. A total of 1,258 genes ( Additional file 1) were up-regulated and 788 genes ( Additional file 2) were down-regulated at 24 h exposure to silver-NP-hydrogel. Also, 1,532 genes ( Additional file 5) were up-regulated and 824 genes ( Additional file 6) were down-regulated at 24 h exposure to hydrogel alone. After the 48 h exposure, a total of 843 genes ( Additional file 3) were up-regulated and 642 genes ( Additional file 4) were down-regulated from silver-NP-hydrogel exposure. In contrast, 99 genes ( Additional file 7) were up-regulated and 370 genes ( Additional file 8) were down-regulated from exposure to hydrogel alone.

**Figure 3:**
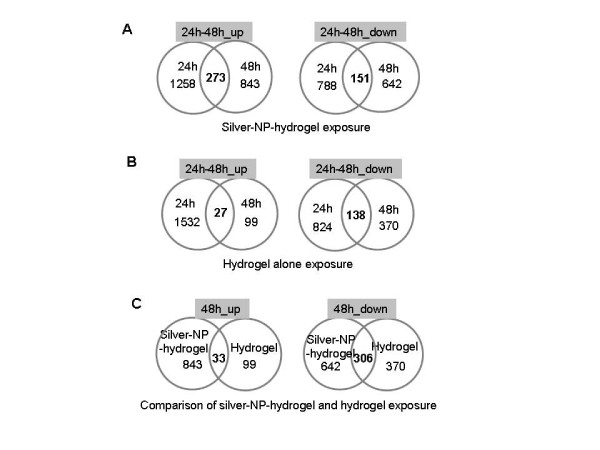
**Gene expression profiling based on DNA microarray data.** A, Up- and down-regulated genes in silver-NP-hydrogel exposed HeLa cells (Additional file 1, 2, 3 and 4); B, Up- and down-regulated genes in hydrogel alone exposed HeLa cells (Additional file 5, 6, 7 and 8); C, A comparison of common expressed genes in the silver-NP-hydrogel and hydrogel alone exposed cells (Additional file 13 and Additional file 14).

By comparing 24 h and 48 h gene expression profiling, it was observed that the 21.7% of genes that were up-regulated at 24 h post-exposure to silver-NP-hydrogel were continuously highly expressed until the 48 h exposure (273 genes, Additional file 9) (Figure 3A). This suggested that the silver-NP-hydrogel continuously induced gene up-regulation at 48 h exposure. In addition, 19.16% of the genes that were down-regulated at 24 h post-exposure, were continuously lower-expressed until the 48 h exposure (151 genes, Additional file 10) (Figure 3A). This suggested that the silver-NP-hydrogel continuously caused gene down-regulation at the 48 h exposure period.

In contrast, most of the up-regulated genes at 24 h exposure to hydrogel alone had recovered after continuous exposure up to 48 h. Only 1.76% of up-regulated genes at 24 h exposure continuously showed higher-expression at 48 h (27 genes, Additional file 11) (Figure 3B). This observation suggested that the gene up-regulation was a transient response in the cells against the extracellular stimulation from the hydrogel. However, 16.75% of genes that were down-regulated at 24 h post-exposure to hydrogel alone, were continuously lower-expressed until 48 h exposure (138 genes, Additional file 12) (Figure 3B). These results suggested that down-regulated genes rather than up-regulated genes might play a role in the cell response against hydrogel alone.

By further comparing changed genes common to both silver-NP-hydrogel and hydrogel alone exposure, it was found that, of the 843 up-regulated genes at 48 h silver-NP-hydrogel exposure, only 3.91% of genes were common to those expressed at hydrogel alone exposure (33 genes, Additional file 13); and 96.09% of the genes were unique for silver-NP-hydrogel exposure (Figure 3C). For the 642 down-regulated genes at 48 h of silver-NP-hydrogel exposure, 46.66% (306 genes, Additional file 14) of genes were common to those expressed at hydrogel alone exposure; and 53.34% of the genes were unique changes for silver-NP-hydrogel exposure (Figure 3C). These results suggested that the up-regulated genes induced by silver-NP-hydrogel could be mainly attributed to silver-NPs and that the down-regulated genes induced by silver-NP-hydrogel could be attributed in part to silver-NP and hydrogel components. It was indicated that silver-NPs could play a key role in the silver-NP-hydrogel induced toxicity, while hydrogel components might also play a role in the toxic response to silver-NP-hydrogel by down-regulating some gene expressions.

### GO function analysis of differential expressed genes

Based on gene ontology (GO) biological processes, the genes which were up- and down-regulated at 48 h of silver-NP-hydrogel exposure were further analyzed using the program of GO Surfer. With the gene number-based signal pathway activation analysis, the GO pathway which has theoretically significant activation (p < 1.0E-03) was further picked-up as shown in Table 2 and Table 3.

**Table 2:** GO function analysis of differential expressed genes at 48 h exposure of silver-NP-hydrogel

**Functional GO pathway**	REF.-LIST*/Up-exp^**#**^**/expected**^**$**^	**P value**
cell communication	4365/231/174.28	p = 1.04E-06
cell-cell signaling	1331/81/53.14	p = 2.14E-04
cell adhesion	1333/77/53.22	p = 8.67E-04
signal transduction	4191/215/167.34	p = 2.37E-05
intracellular signaling cascade (JAK-STAT cascade)	1568/102/62.61	p = 1.02E-06
metabolic process	8267/373/330.08	p = 7.32E-04
lipid metabolic process	1119/94/44.68	p = 7.32E-12
carbohydrate metabolic process	952/69/38.01	p = 1.08E-06
response to stimulus	1798/119/71.79	p = 7.96E-08
transport	2857/164/114.07	p = 6.18E-07
endocytosis	575/43/22.96	p = 9.23E-05
cellular defense response	457/38/18.25	p = 2.75E-05
immune system process	2628/171/104.93	p = 7.69E-11
immune response	756/52/30.19	p = 1.41E-04

* reference list, that is all genes number related to the GO pathway;

^#^ up-expressed genes number in this study;

^$^ expected minimum genes number for activating of signal pathway.

Based on the gene ontology (GO) biological process, the GO pathway, which has theoretically significant activation (p < 1.0E-03), related to up-regulated genes.

**Table 3:** GO function analysis of differential expressed genes at 48 h exposure of silver-NP-hydrogel

**Functional GO pathway**	REF.-LIST*/Down- exp^**#**^**/expected**^**$**^	**P value**
nucleobase, nucleoside, nucleotide and nucleic acid metabolic processes	3825/138/81.84	p = 8.40E-12
cell cycle	1840/62/39.37	p = 2.60E-04
mitosis	635/27/13.59	p = 6.90E-04

* reference list, that is all genes number related to the GO pathway;

^#^ down-expressed genes number in this study;

^$^ expected minimum genes number for activating of signal pathway.

Based on the gene ontology (GO) biological process, the GO pathway, which has theoretically significant activation (p < 1.0E-03), related to down-regulated genes.

Pathway analysis of GO/Biological processes showed that fourteen functional signal pathways were related to up-regulated genes at 48 h of silver-NP-hydrogel exposure (Table 2), suggesting that the up-regulated genes might play an important role in adverse cell responses. These fourteen functional signal pathways were unique for silver-NP-hydrogel exposed cells, but not common to hydrogel alone exposed cells, suggesting that the changes in functional signal pathways were attributed to silver-NPs. Under the same analysis method, in contrast, non-signal pathway which was theoretical significant activation was observed in up-regulated genes at 48 h of hydrogel alone exposure (data not shown). In addition, the most up-regulated genes at 24 h exposure had recovered at 48 h of hydrogel exposure (Figure 3B). It was further suggested that the up-regulated genes induced by the hydrogel components might not affect cell function. Several pathways were related to down-regulated genes at 48 h of silver-NP-hydrogel exposure (Table 3). These included the nucleobase, nucleoside, nucleotide and nucleic acid metabolic processes pathway, cell cycle pathway and mitosis pathway. These results suggested that the down-regulated genes might cause cell damage by affecting cell proliferation, cell cycles and mitosis. The later two pathways, being unique to silver-NP-hydrogel exposure, were not common to hydrogel alone exposure, suggesting that the changes of these functional signal pathways are mainly attributed to silver-NPs. These events were considered to be closely involved with cytotoxicity and genotoxicity. At 48 h of hydrogel exposure, three pathways related to down-regulated genes were also showed theoretical significant activation. They included a metabolic process (p = 4.02E-04), nucleobase, nucleoside, nucleotide and nucleic acid metabolic processes (p = 1.69E-10) and the primary metabolic process (p = 2.97E-04). These results suggested that the down-regulated genes induced by hydrogel alone might have some effect on cell proliferation and metabolism.

### Real-time PCR verification of differential expressed genes

To verify the reliability of differential expressed gene identified by the DNA microarray, five genes selected from up- and down-regulated genes expressed in silver-NP-hydrogel 48 h exposure were further examined with real-time PCR detection. The results showed that the gene expression was basically consistent with that of the microarray analysis, indicating a good reliability and reproducibility of the microarray in the current study (Table 4).

**Table 4:** The gene expression detected by real-time PCR and detected by DNA microarray at 48 h exposure of silver-NP-hydrogel

**determination**	**IL1A**	**HMOX1**	**DDIT3**	**MT1F**	**PDGFRB**
Real-time PCR (2^-△△Ct^)	15.97	6.58	8.18	82.96	0.4
DNA microarray (fold changes)	2.76	2.41	2.17	5.63	−2.14

## Discussion

Genotoxicity evaluation is an idea assessment of biosafety at molecular level for nanomaterials-based medical devices. In this study, a significant increases in the micronucleation frequency (MNF) of HeLa cells was induced by the silver-NP-hydrogel exposure at concentrations of 20-, 40-, and 60-mg/ml (in medium), compared to the negative control (*P* < 0.05), suggesting that the silver-NP-hydrogel has a potential risk of genotoxicity. The study also showed that hydrogel alone did not show significant change, compared to the negative control, suggesting that genotoxicity caused by silver-NP-hydrogel was attributed to silver-NPs. Kawata et al. demonstrated that exposure to 1.0 μg/ml of silver-NPs (7–10 nm in size) induced MNF up to 47.9% in the HepG2 cell line [21]. AshaRani et al. reported that exposure at the 25 μg/ml of silver-NPs (6–20 nm in size) induced chromosomal aberrations in 10% of the IMR-90 normal cell line, and in 20% of the U251 cancer cell line [22,23]. In the comet assay and micronucleus (MN) assay for BEAS-2B cells, silver-NPs (43–260 nm in size, dispersed in medium) stimulated DNA breakage and MN formation in a dose-dependent manner [24]. In this study, the size of silver nanoparticles contained in silver-NP-hydrogel ranged from 5 nm to 30 nm (observed by TEM, dispersed in water). Number of studies has reported for the in vivo genotoxicity and carcinogenicity by silver-NPs. Study by Kim et al. reported that there was no genotoxic effect in rats after 28 days oral exposure to Ag NPs [11]. Kim et al. also reported that no genotoxic effect in rats after 90 days inhalation of Ag NPs [34]. In contrast, however, our recent study reported that silver-NP-hydrogel induced micronuclei, nuclei disruption, chromatin concentration and cell apoptosis in rabbit reproductive organ tissues in silver-NP-hydrogel administration through the vagina [35]. The difference of findings in potential genotoxic and carcinogenic risks of nanomaterials is possibly due to the insufficient characterization of test material, difference in the experimental design, use of different animal models and species, difference in dosimetry, and different targeting organs [36]. It was known that confirmation of asbestos nanofiber as a carcinogen in Japan took over 10 years [37]. Since the silver nanoparticles are still widely used clinically in some countries, it is important to gain a better understanding of their genotoxicity and carcinogenecity.

Two important molecular mechanisms were considered to be involved in the in vitro toxicity and genotoxicity induced by silver-NP-hydrogel, as further discussed below.

### The balance between anti-ROS-toxicity and DNA damage

From the data in this study, the signaling pathways and regulatory proteins involved in anti-ROS-toxicity, DNA damage, apoptosis, cell cycles and mitosis might be associated with genotoxicity caused by silver-NP-hydrogel.

Metallothioneins (MTs) are considered to be essential biomarkers in metal-induced toxicity [38] as facilitating metal detoxification and protection from free radicals [39]. A report on heavy metal toxicity in Javanese medaka showed that MT upregulation occurs in silver mediated toxicity [40]. Hemeoxygenase-1 (HO-1) is an ROS sensor and a cryoprotective agent possessing antioxidant and anti-inflammatory properties. HO-1 breaks down heme to antioxidant biliverdin, carbon monoxide and iron under stress conditions [41,42]. It was reported that oxidative stress response genes (superoxide dismutase 2, glutathione reductase 1, etc.) in mouse brain following silver-NP exposure were upregulated [43]. In the present study, 10 metallothionein genes (MT1F, MT1A, MT2A, MT1B, MT1G, MT1H, MT1X, MT1L, MT1M, MT1E), HO-1 and oxidative stress induced growth inhibitor 1 (OSGIN1) were significantly up-regulated at 48 h of silver-NP-hydrogel exposure Additional file 5 and Additional file 6. All these molecules are believed to protect cells against metal-induced ROS toxicity. Metal ions including silver act as catalysts and can produce reactive oxygen species in the presence of oxygen, which is thought to be a mechanism of toxicity. Previous studies showed that silver-NPs increase the production of intracellular ROS [20]. The ROS can act as signal molecules promoting cell cycle progression, and can induce oxidative DNA damage [27-29]. We previously reported that HeLa cells exposed to silver-NPs consistently over-express isoforms of metallothionein (MT1A, MT1F, MT1G, MT1X, and MT2A) [25]. AshaRani et al. reported that the MT-1 F and HO-1 were upregulated in IMR-90 cells following silver-NP treatment [22]. Kawata et al. also reported that three metallothionein genes (MT1H, MT1X, MT2A) were significantly upregulated in HepG2 cells exposed to silver-NPs [21]. These studies suggested that the cells protect themselves against silver NP-mediated toxicity through up-regulating metallothionein genes and oxidative stress induced genes.

On the other hand, the genes which are related to the DNA damage and apoptosis, such as DNA-damage-inducible transcript 3 (DDIT3), caspase 1, and apoptosis-related cysteine peptidase (CASP1) were up-regulated by silver NPs. Changes in chromosome related genes (24 genes up-regulated and 26 genes down-regulated) ( Additional file 3 and Additional file 4) found in this study might damage the chromosomes. Apoptosis inhibitors, such as BCL2 interacting protein (HRK), BIK (BCL2-interacting killer, apoptosis-inducing), Fas apoptotic inhibitory, molecule 3 (FAIM3), apoptosis inhibitor (FKSG2), suppression of tumorigenicity 13 (ST13), growth arrest and DNA-damage-inducible, alpha (GADD45A) were also significantly up-regulated. This suggested that silver nanoparticles induced apoptosis via a mitochondrial pathway. Apoptosis and chromosome damage could be subsequently involved in cytotoxicity and genotoxicity.

In addition, analysis of the activating signal pathways in this study also suggested that cell cycles and the mitosis signal pathway were significantly down-regulated, which was uniquely represented at the silver-NP-hydrogel 48 h exposure. These pathways are considered to be closely involved in cell proliferation, apoptosis and tumorigenesis progression. There were 62 genes related to cell cycle signal pathways. Among them, 27 genes were related to mitosis pathway ( Additional file 15). In these genes, cell division cycle 14 homolog A (CDC14A) showed a 13 -fold (log _2_ = −3.71) down-regulation. CDC14A is a member of the dual specificity protein tyrosine phosphatase family. It is highly similar to saccharomyces cerevisiae Cdc14, a protein tyrosine phosphatase involved in the exit of cell mitosis and initiation of DNA replication, playing a role in cell cycle control. CDC14A protein has been shown to interact with and dephosphorylate the tumor suppressor protein p53, and is thought to regulate the function of p53 [44]. Human CDC14A shares sequence similarity with the recently identified tumor suppressor, MMAC1/PTEN/TEP1. CDC14A is located at chromosome band 1p21, a region that has been shown to exhibit loss of heterozygosity in highly differentiated breast carcinoma and malignant mesothelioma. Thus, CDC14A has been thought to be a tumor suppressor gene [44]. The down-regulation of CDC14A in this study suggested that it might play a role in the potential genotoxicity induced by silver-NP-hydrogel.

**Figure 4:**
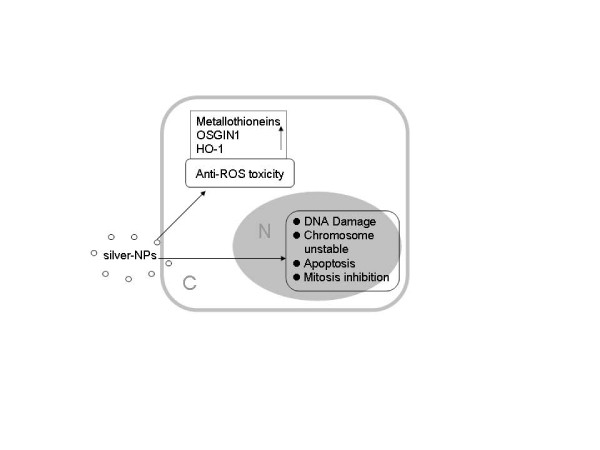
**Scheme of molecular mechanisms of cellular response against silver-NP-hydrogel exposed.** The balance among anti-ROS-toxicity and DNA damage, apoptosis, mitosis inhibition of the cells might play important role in cytotoxicity. These responses were mainly induced by silver-NPs contained in silver-NP-hydrogel.

As summarized in Figure 4, the balance between anti-ROS-toxicity and DNA damage, apoptosis, mitosis inhibition of the cells could be the main events which decide the future of the cells.

### JAK-STAT signal transduction pathway

The JAK-STAT (Janus kinase/signal transducers and activators of transcription) cascade is an important signal pathway which affects basic cell functions such as cell growth, differentiation and apoptosis [45]. STAT is a signal transducer and activator of transcription. It conveys or transduces the signal from the receptor-JAK (Janus Kinase) complex to the DNA in the cell nucleus [45]. In mammals, the JAK-STAT signal pathway is the principal signaling mechanism for a wide array of cytokines and growth factors [45,46]. Defects in JAK-STAT proteins can result in immune deficiency disease and cancer [45]. JAKs, which have tyrosine kinase activity, bind to some cell surface cytokine receptors. So, the cytokines, as ligands, through binding to the receptor would trigger activation of JAKs [46-50]. In this study, it was found that silver-NP-hydrogel exposure induced JAK-STAT cascade-related gene up-regulation, not only at 24 h exposure but also for 48 h exposure Additional file 5 and Additional file 6, and implied that the silver-NP-hydrogel might play a role in JAK-STAT pathway.

It was found by further analysis that many interferon-induced proteins (IFI), interferon-induced protein in the tetratricopeptide (IFIT) family, and interleukin (IL) family were up-regulated ( Additional file 5 and Additional file 6). These inflammatory factors acting as ligands while they participate in immune response pathway, may also trigger the activation of JAK-STAT signal pathway through binding to the JAK receptor.

## Conclusions

In summary, the silver-NP-hydrogel induced micronuleus formation in HeLa cells. The toxic effects caused by silver-NP-hydrogel arrived mainly from silver-NPs. Based on DNA microarray and GO pathway analysis, the molecular response and mechanisms of toxicity induced by silver-NP-hydrogel might relate to some up-regulated genes involved in fourteen theoretical activating signaling pathways and to some down-regulated genes involved in three signal pathways at 48 h of silver-NP-hydrogel exposure in HeLa cells. These signal pathways play important roles in metabolisms, cell communication, signal transduction, cellular defense response, transport, cell cycles and mitosis. The down-regulation of CDC14A via mitosis pathway suggested that it may play a role in the potential genotoxicity induced by silver-NP. The balances between anti-ROS response and DNA damage, chromosome instability and mitosis inhibition might play important roles in silver-NP induced toxicity. It was also demonstrated that activations of both JAK-STAT signal transduction pathway and immune response pathway could be involved in the mechanisms of toxicity caused by silver-NP-hydrogel.

## Materials and methods

### Test materials and chemicals

Silver-NP-based hydrogel (silver-NP-hydrogel) used in this study was a clinical available product, and has been used in clinic for treating cervicitis and cervical erosion of women. The product provided by Egeta Co. (Shenzhen, China, Batch Number 090701) was manufactured by simply mixing aqueous silver-NP solution (concentration of 2,000 ppm, purchased from Nanux, Korea, Cat. No. SL1105001) and hydrogel components to achieve a concentration of 0.38 μg/mg (silver- NP-hydrogel). The silver NPs were not coated by any compounds such as PVP, citrate or BSA. To determine the NP size distribution, silver-NP-hydrogel was dissolved in water. Then, the silver particles were collected by centrifuging and placed on a cupper-net for evaluation of size distribution by TEM ( Additional file 16: Figure S1). As determined through TEM, the size distribution of the nanoparticles was as follows: 3–5 nm, 47.9%; 5–10 nm, 50.8%; 10–30 nm, 1.3%. The hydrogel was composed of sterile water, glycerine, carbomer and triethanolamine (TEA). The hydrogel component alone (without silver-NP) was used as the compared control.

Cytochalasin B (Cyt-B), Mitomycin C (MMC) and Dimethyl sulfoxide (DMSO) were purchased from Sigma-Aldrich (USA). The Cyt-B was dissolved in DMSO (2.0 mg/ml), and the MMC was dissolved in NaCl solution (10 μg/ml) for use as a stock solution. All solutions were sterilized by using 0.2 μm-pore film and stored at −20°C.

### Cell culture and treatment

Silver-NP-hydrogel is currently a clinical product on the markets and is commonly used for treating cervicitis and cervical erosion of women. Therefore, the HeLa cell line was chosen as a cell model in this study. HeLa cells were originally purchased from RIKEN (Wako, Japan) with a RIKEN Cell line number. RCB0007. The cells were cultured in DMEM (GIBCO, USA), with 10% FBS (GIBCO, USA) and 100 U/ml penicillin/100 μg/ml streptomycin (GIBCO, USA), in a humidified 5% CO_2_ atmosphere at 37°C.

To determine a suitable concentration of silver-NP-hydrogel for the study, a preliminary experiment was performed by adding silver-NP-hydrogel to culture medium at concentrations of 2.5, 5, 10, 20, 40, and 60 mg/ml. After ultrasonic treatment (300 W, 42 kHz,) for 10 min, the media with various concentrations of silver-NP-hydrogel was applied to cells which had been pre-cultured for 24 h (70–80% confluence), and the cells were then cultured for another 24 h. Cell viability was determined using a methyl tetrazolium (MTT) assay by measuring the optical density of the formazan product. Briefly, after the exposure to silver-NP-hydrogel solution, the cells were washed. A mixture of 20 μl of MTT (5 mg/ml) and 100 μl of non-phenol-red medium were added to the cells, and incubated for 4 h. After through washes and DMSO treatment, 100 μl of the supernatant of each sample was transferred for optical density (OD) detection. The assay was performed using a plate reader at a wavelength 570 nm, with 630 nm as the reference wavelength. The results represented a percentage of the relative viability of cells against to the untreated control. Based on this preliminary experiment, a middle-level viability inhibition of the cells, compared to that in the non-treatment control cells, was found at a concentration (IC50) about 40 mg/ml of silver-NP-hydrogel (in culture media) which containing 15.2 μg/ml of silver-NPs. This concentration was determined according to the concentration-relative cell viability (%) curve equation, and the non-treatment control cell viability (OD level) was served as 100%. An EC50 NP concentration was selected also because the EC50 or IC50 is a conventional coefficient in ISO and OECD standards for toxicity assessments. To minimize a possible detection of RNA species degraded from dying cells at this toxicity level, the culture dishes were thoroughly washed three times by PBS before harvesting to remove the dead cells and degraded molecules. For the gene expression microarray experiment, 40 mg/ml of silver-NP-hydrogel was used. The cells were seeded at a concentration of 5 × 10^5^ cells/35-cm cell culture dish for CBMN assay, MTT assay and DNA microarray experiment. The cells were exposed to the silver-NP-hydrogel for 24 and 48 h, respectively.

### CBMN assay

The CBMN assay was carried out using the protocol described as below. Briefly, the HeLa cells were seeded and maintained to 70–80% confluence at 24 h. Silver-NP-hydrogel was added to culture media at concentrations of 20, 40 and 60 mg/ml respectively, and after ultrasonic treatment (300 W, 42 kHz for 10 min) the media with silver-NP-hydrogel was applied to the cells. MMC (0.1 μg/ml) was used as a positive control, and NaCl (50 μl/ml) was used as a negative control. The cells were then further cultured for 24 h. After washing thoroughly with three times of PBS, Cytochalasin-B (final conc., 3 μg/ml) was added to the cells, and the cells were cultured for another 18 h. The cells were subsequently collected. After a hypotonic treatment in 2 ml of 0.075 M KCl at room temperature for 5 min, the cells were fixed using methyl alcohol:acetic acid (3:1). The cells were then placed onto a slide, dried at room temperature, and stained using 4% Giemsa solution (pH 6.8) for 30 min.

The slides were scored at 400× magnification blindly by two investigators separately. The micronucleation frequency (MNF, %) was determined for 1000 binucleated cells (BNCs).

### Total RNA isolation and DNA microarray

The cells reached 70–85% confluence at both 24 h and 48 h cultures with the treatment of silver-NP-hydrogel at a concentration of 40 mg/ml, and almost a full conference in the control cultures with non-treatment (Figure 2). The total RNA from each sample was extracted from the HeLa cells using ISOGEN RNA isolation reagent (Nippon gene, Japan) according to the instructions provided by the manufacturer. After decontamination treatment of genomic DNA using DNase I digestion, the quality and integrity of the RNA samples were determined by appearance of the distinct 28 S and 18 S bands of ribosomal RNA on agarose gel electrophoresis. Total RNA purity was measured spectrophotometrically by the absorbance ratio 260/280 nm. Results ranged between 1.7–2.1.

A gene expression study was conducted using “Two-color microarray-based gene expression analysis” (Agilent technologies, USA, Whole Human Genome Microarray 4x44K, G4110F containing 41000 of DNA oligomer unique probes). Briefly, 1 μg of each sample of RNA was amplified using an Amino Allyl MessageAmp II aRNA Amplification Kit (Ambion, USA). Amplified RNA (aRNA) was labeled using Cy5 and Cy3 according to the instructions provided by the manufacturer. The silver-NP-hydrogel or hydrogel-alone exposure samples were labeled with Cy5, and untreated cells were labeled with Cy3 which was used as control against the treated Cy5-labeled sample. After hybridizing the samples for 16 h at 65°C, the gene chips were washed. The hybridized chips were fluorescently scanned with a microarray scanner (GenePix 4000B, USA) to collect the images. The ratios of intensity (Log_2_Cy5/Cy3) were calculated and normalized with GenePixPro 6.1 software. Filtering of the results was done as follows: genes were considered as up-regulated when the Log_2_Cy5/Cy3 ratio was higher than 1 (Cy5/Cy3 was higher than 2) and as down-regulated when the Log_2_Cy5/Cy3 ratio was lower than −1 (Cy5/Cy3 was lower than −2). Genes were considered as unregulated when the Log_2_Cy5/Cy3 ratio was between 1 and −1. The GO pathway data was further classified into functional categories. The genes, which were consistently up-regulated and down-regulated at 48 h of silver-NP-hydrogel exposure, were tabled for further analysis.

### Gene ontology analysis of gene expression

To determine biological relevant gene ontology terms (GO, provided by NCBI) of differentially expressed genes in HeLa cells, the software “PANTHER”, was used. It provides gene expression data analysis/Comparison of gene lists (http://www.pantherdb.org/tools/genexAnalysis.jsp). The analysis was performed using Unigene ID as the identifier for biological process categories.

### Real-time PCR

The reliability of the gene expression profile was validated by real-time PCR (SYBR Green method) for five selected genes, viz., interleukin 1, alpha (IL1A); heme oxygenase (decycling) 1(HMOX1); DNA-damage-inducible transcript 3 (DDIT3); metallothionein 1 F (MT1F) and platelet-derived growth factor receptor, beta polypeptide (PDGFRB) at 48 h exposure of silver-NP-hydrogel. Real-time PCR was performed using the ABI 7900 HT Fast RealTime PCR system (Applied Biosystem, USA). Briefly, total RNA (25 μg) from each sample was DNase I digested by the following reactions: RQ1 RNase-Free DNase 10× Reaction Buffer (5 μl), RQ1 RNase-Free DNase I (2 μl,Promega, USA), Recombinant RNasin RNase Inhibitor (1 μl, Promega, USA), Nuclease-Free Water to total volume 50 μl, with incubation for 30 min at 37°C. Following purification of DNase I digested RNA, 2 μg of RNA was reverse-transcribed into cDNA by using M-MLV reverse transcriptase (Invitrogen, USA). One μl of the cDNA sample was added to the PCR mixture which was composed of 10 μl of Power SYBR Green PCR Master Mix (Applied Biosystem, USA), 0.5 μl of forward primer (10 μM) and 0.5 μl of reverse primer (10 μM). Nuclease-free water was added to bring the volume up to 20 μl, and the mixture was subjected to PCR amplification. The primers used in this study are listed in Table 5. The threshold cycles (Ct) in each sample were measured by comparing their amplification with that of standard samples and was normalized to that of the housekeeping gene actin. An average Ct of triplicate detection for each gene, ΔCt (Detecting gene - House keeping gene); and ΔΔCt (Test sample-Control sample) was obtained; finally calculated 2^-ΔΔCt^ and represented as the differential expression of test genes.

**Table 5:** Primers used in real-time PCR

**Gene**	**GeneBank**	**Primer Sequence 5′ → 3′**
Actin	NM 001101	Forward Primer: CATGTACGTTGCTATCCAGGC Reverse Primer: CTCCTTAATGTCACGCACGAT
MT1F	NM 005949	Forward Primer: CCCACTGCTTCTTCGCTTCT Reverse Primer: GAGAAAGGTTGTCCTGGCATC
IL1A	NM 000575	Forward Primer: AATGACGCCCTCAATCAAAGTA Reverse Primer: CTCCTTCAGCAGCACTGGTTG
HMOX 1	NM002133	Forward Primer: AAGAGGCCAAGACTGCGTTC Reverse Primer: GAGTGTAAGGACCCATCGGAGA
DDIT 3	NM 004083	Forward Primer: GTCCTGTCTTGATGAAAATGG Reverse Primer: GTGCTTGTGACCTCTGCTGG
PDGFRB	NM 002609	Forward Primer: GAGACTGTTGGGCGAAGGTTA Reverse Primer: GAGATGGTTGAGGAGGTGTTGAC

### Statistical analysis

The data were represented as the mean ± SD. The CBMN assay was repeated three independent times. The data was statistically analyzed using the SPSS, version 12.0.1), the *t*-test, ANOVA and Dunnett test (2-sided). Differences were considered significant if the *P*-value was less than 0.05.

## Abbreviations

silver-NP: Silver nanoparticle; silver-NP-hydrogel: Silver nanoparticle based hydrogel; CBMN: Cytokinesis-block micronucleus; ROS: Reactive oxygen species; Cyto-B: Cytochalasin B; DMSO: Dimethyl sulfoxide; MMC: Mitomycin C; MTT: Methyl tetrazolium; GO: Gene ontology; MNF: Micronucleation frequency; BNCs: Binucleated cells; TEA: Triethanolamine; CDC14A: Cell division cycle 14 homolog A; JAK-STAT: Janus kinase/signal transducers and activators of transcription; IFI: Interferon-induced proteins; IFIT: Interferon-induced protein in the tetratricopeptide; IL: Interleukin; IL1A: Interleukin 1 alpha; HMOX1: Heme oxygenase (decycling); DDIT3: DNA-damage-inducible transcript 3; MT1F: Metallothionein 1 F; PDGFRB: Platelet-derived growth factor receptor beta polypeptide.

## Competing interests

The authors declare that they have no competing interests.

## Authors’ contributions

Liming Xu participated in the design of study, sequence alignment, performed the statistical analysis and drafted the manuscript. Xuefei Li carried out the genotoxic assay, participated in the sequence alignment and drafted the manuscript. Taro Takemura and Nobutaka Hanagata carried out the DNA microarray experiment and data analysis. Gang Wu and Laisheng Lee Chou participated in the design of study, data analysis and coordination, and drafting manuscript. All authors read and approved the final manuscript.

## Supplementary Material

Additional file 1 Up-regulated genes in cells exposed to silver-NP-hydrogel for 24 h. Fold-change is logarithmic ratio (log2 ratio) to expression level in control. Click here for file

Additional file 2 Down-regulated genes in cells exposed to silver-NP-hydrogel for 24 h. Fold-change is logarithmic ratio (log2 ratio) to expression level in control. Click here for file

Additional file 3 Up-regulated genes in cells exposed to silver-NPs-hydrogel for 48h. Fold-change is logarithmic ratio (log2 ratio) to expression level in control. Click here for file

Additional file 4 Down-regulated genes in cells exposed to silver-NPs-hydrogel for 48h. Fold-change is logarithmic ratio (log2 ratio) to expression level in control. Click here for file

Additional file 5 Up-regulated genes in cells exposed to Hydrogel for 24 h. Fold-change is logarithmic ratio (log2 ratio) to expression level in control. Click here for file

Additional file 6 Down-regulated genes in cells exposed to Hydrogel for 24 h. Fold-change is logarithmic ratio (log2 ratio) to expression level in control. Click here for file

Additional file 7 Up-regulated genes in cells exposed to Hydrogel for 48h. Fold-change is logarithmic ratio (log2 ratio) to expression level in control. Click here for file

Additional file 8 Down-regulated genes in cells exposed to Hydrogel for 48 h. Fold-change is logarithmic ratio (log2 ratio) to expression level in control. Click here for file

Additional file 9 Common up-regulated genes in cells exposed to silver-NP-hydrogel for 24 h and 48 h. Fold-change is logarithmic ratio (log2 ratio) to expression level in control. Click here for file

Additional file 10 Common down-regulated genes in cells exposed to silver-NP-hydrogel for 24h and 48 h. Fold-change is logarithmic ratio (log2 ratio) to expression level in control. Click here for file

Additional file 11 Common up-regulated genes in cells exposed to hydrogel for 24 h and 48 h. Fold-change is logarithmic ratio (log2 ratio) to expression level in control. Click here for file

Additional file 12 Common down-regulated genes in cells exposed to hydrogel for 24 h and 48 h. Fold-change is logarithmic ratio (log2 ratio) to expression level in control. Click here for file

Additional file 13 Common up-regulated genes in cells exposed to hydrogel and silver-NP-hydrogel for 48h. Fold-change is logarithmic ratio (log2 ratio) to expression level in control. Click here for file

Additional file 14 Common down-regulated genes in cells exposed to hydrogel and silver-NP-hydrogel for 48h. Fold-change is logarithmic ratio (log2 ratio) to expression level in control. Click here for file

Additional file 15 Down-regulated genes related to mitosis pathway at silver-NP-hydrogel treatment for 48h. Click here for file

Additional file 16. Figure S1 The size and size distribution of the silver nanoparticles determined by transmission electron microscopy (TEM). A: X 10000, bar = 200 nm; B: X 20000, bar = 100 nm. Click here for file
