# Supplementary material for: Genotoxicity and molecular response of silver nanoparticle (NP)-based hydrogel
Source: J Nanobiotechnology. 2012 May 1;10:16. doi: 10.1186/1477-3155-10-16 (PMC3430588; doi:10.1186/1477-3155-10-16)
Supplement: Additional file 1 — Up-regulated genes in cells exposed to silver-NP-hydrogel for 24 h. Fold-change is logarithmic ratio (log2 ratio) to expression level in control. [file 1477-3155-10-16-S1.pdf]

Additional File 1. Up-regulated genes in cells exposed to silver-NP-hydrogel for 24 h. Fold-change is logarithmic ratio (log_2 ratio) to expression level in control.

GeneName	Description	Fold-change (log_2 ratio)
C6orf208	Homo sapiens chromosome 6 open reading frame 208, mRNA (cDNA clone MGC:120218 ,IMAGE:40023219), [BC101251]	**6.644**
UNQ9419	Homo sapiens clone DNA180542 AHPA9419 (UNQ9419) mRNA, [AY358263]	**5.082**
MT1F	Homo sapiens metallothionein 1F (MT1F), mRNA [NM_005949]	**4.511**
MT2A	Homo sapiens metallothionein 2A (MT2A), mRNA [NM_005953]	**4.300**
MT1A	Homo sapiens metallothionein 1A (MT1A), mRNA [NM_005946]	**4.244**
MT1G	Homo sapiens metallothionein 1G (MT1G), mRNA [NM_005950]	**4.126**
MT1B	Homo sapiens metallothionein 1B (MT1B), mRNA [NM_005947]	**3.925**
MT1M	Homo sapiens metallothionein 1M (MT1M), mRNA [NM_176870]	**3.854**
MT1H	Homo sapiens metallothionein 1H (MT1H), mRNA [NM_005951]	**3.776**
MT1X	Homo sapiens metallothionein 1X (MT1X), mRNA [NM_005952]	**3.691**
MT1L	Homo sapiens metallothionein 1L (gene/pseudogene) (MT1L), non-coding RNA [NR_001447]	**3.651**
MT1JP	Homo sapiens MTB (MTB) mRNA, complete cds. [AF348994]	**3.477**
NR1H4	Homo sapiens nuclear receptor subfamily 1, group H, member 4 (NR1H4), mRNA [NM_005123]	**3.194**
SERPINH1	Homo sapiens serpin peptidase inhibitor, clade H (heat shock protein 47), member 1, (collagen binding protein 1) (SERPINH1), mRNA [NM_001235]	**3.186**
HSPA6	Homo sapiens heat shock 70kDa protein 6 (HSP70B') (HSPA6), mRNA [NM_002155]	**3.145**
XX-FW88277B6.1	Homo sapiens cancer/testis antigen CT45-1 (CT45-1), mRNA [NM_001017417]	**3.027**
GCNT3	Homo sapiens glucosaminyl (N-acetyl) transferase 3, mucin type (GCNT3), mRNA [NM_004751]	**2.989**
HSPA1A	Homo sapiens heat shock 70kDa protein 1A (HSPA1A), mRNA [NM_005345]	**2.947**
HMOX1	Homo sapiens heme oxygenase (decycling) 1 (HMOX1), mRNA [NM_002133]	**2.926**
CCL26	Homo sapiens chemokine (C-C motif) ligand 26 (CCL26), mRNA [NM_006072]	**2.881**
RP13-36C9.6	Homo sapiens cancer/testis antigen CT45-5 (CT45-5), mRNA [NM_001007551]	**2.822**
SQSTM1	Human phosphotyrosine independent ligand p62B B-cell isoform for the Lck SH2 domain mRNA, [U46752]	**2.817**
GPNMB	Homo sapiens glycoprotein (transmembrane) nmb, mRNA (cDNA clone IMAGE:3345861), [BC011595]	**2.815**
SPINK1	Homo sapiens serine peptidase inhibitor, Kazal type 1 (SPINK1), mRNA [NM_003122]	**2.814**
TRIM62	Homo sapiens tripartite motif-containing 62 (TRIM62), mRNA [NM_018207]	**2.751**
MT4	Homo sapiens metallothionein 4 (MT4), mRNA [NM_032935]	**2.649**
HCFC1	Homo sapiens host cell factor C1 (VP16-accessory protein) (HCFC1), mRNA [NM_005334]	**2.606**

GAS6	Homo sapiens growth arrest-specific 6 (GAS6), mRNA [NM_000820]	**2.560**
KRT34	Homo sapiens keratin 34 (KRT34), mRNA [NM_021013]	**2.554**
KIFC3	Homo sapiens kinesin family member C3 (KIFC3), mRNA [NM_005550]	**2.513**
RRAGD	Homo sapiens Ras-related GTP binding D (RRAGD), mRNA [NM_021244]	**2.488**
GBL	Homo sapiens G protein beta subunit-like (GBL), mRNA [NM_022372]	**2.487**
LIPG	Homo sapiens lipase, endothelial (LIPG), mRNA [NM_006033]	**2.477**
MT1E	Homo sapiens unknown mRNA. [AF495759]	**2.471**
TXNRD1	Homo sapiens thioredoxin reductase 1 (TXNRD1), mRNA [NM_003330]	**2.455**
INPPL1	Homo sapiens inositol polyphosphate phosphatase-like 1 (INPPL1), mRNA [NM_001567]	**2.439**
LOC644936	Homo sapiens cytoplasmic beta-actin pseudogene (LOC644936), non-coding RNA [NR_004845]	**2.395**
KRT8P15	PREDICTED: Homo sapiens misc_RNA (KRT8P15), miscRNA [XR_016994]	**2.382**
AMDHD2	Homo sapiens amidohydrolase domain containing 2 (AMDHD2), mRNA [NM_015944]	**2.377**
INSR	Homo sapiens insulin receptor (INSR), mRNA [NM_000208]	**2.367**
EPHA2	Homo sapiens EPH receptor A2 (EPHA2), mRNA [NM_004431]	**2.360**
PTGES2	Homo sapiens prostaglandin E synthase 2 (PTGES2),mRNA [NM_025072]	**2.358**
MVD	Homo sapiens mevalonate (diphospho) decarboxylase (MVD), mRNA [NM_002461]	**2.344**
POTEE	Homo sapiens POTE ankyrin domain family, member E (POTEE), mRNA [NM_001083538]	**2.343**
PCYT2	Homo sapiens phosphate cytidylyltransferase 2, ethanolamine (PCYT2), mRNA [NM_002861]	**2.325**
LOC647954	PREDICTED: Homo sapiens misc_RNA (LOC647954), miscRNA [XR_018676]	**2.319**
LOC100129113	Homo sapiens cDNA FLJ37158 fis, clone BRACE2026293. [AK094477]	**2.316**
LY96	Homo sapiens lymphocyte antigen 96 (LY96), mRNA [NM_015364]	**2.294**
HSPB8	Homo sapiens heat shock 22kDa protein 8 (HSPB8), mRNA [NM_014365]	**2.291**
EIF4G1	Homo sapiens eukaryotic translation initiation factor 4 gamma, 1 (EIF4G1), mRNA [NM_182917]	**2.285**
ACP2	Homo sapiens acid phosphatase 2, lysosomal (ACP2), mRNA [NM_001610]	**2.279**
POTEF	Homo sapiens POTE ankyrin domain family, member F (POTEF), mRNA [NM_001099771]	**2.276**
MGAT4B	Homo sapiens mannosyl isozyme B (MGAT4B), mRNA [NM_054013]	**2.268**
FBXL14	Homo sapiens F-box and leucine-rich repeat protein 14 (FBXL14), mRNA [NM_152441]	**2.257**
PSAP	Homo sapiens prosaposin (PSAP), mRNA [NM_001042465]	**2.256**
ATPBD3	Homo sapiens ATP binding domain 3 (ATPBD3), mRNA [NM_145232]	**2.235**
HKDC1	Homo sapiens hexokinase domain containing 1 (HKDC1), mRNA [NM_025130]	**2.231**
PPP1R15A	Homo sapiens protein phosphatase 1, regulatory (inhibitor) subunit 15A (PPP1R15A), mRNA [NM_014330]	**2.229**
ACSS2	Homo sapiens acyl-CoA synthetase short-chain family member 2 (ACSS2), mRNA [NM_018677]	**2.225**

LOC100128116	PREDICTED: Homo sapiens misc_RNA (LOC100128116), miscRNA [XR_038299]	**2.203**
ARL4C	Homo sapiens ADP-ribosylation factor-like 4C (ARL4C), mRNA [NM_005737]	**2.197**
LOC407835	Homo sapiens mitogen-activated protein kinase kinase 2 pseudogene (LOC407835), non-coding RNA [NR_002144]	**2.185**
SLC12A8	Homo sapiens solute carrier family 12), member 8 (SLC12A8), mRNA [NM_024628]	**2.177**
TNFSF9	Homo sapiens tumor necrosis factor (ligand) superfamily, member 9 (TNFSF9), mRNA [NM_003811]	**2.177**
TM4SF19	Homo sapiens transmembrane 4 L six family member 19 (TM4SF19), mRNA [NM_138461]	**2.167**
TIMM44	Homo sapiens translocase of inner mitochondrial membrane 44 homolog (yeast) (TIMM44), mRNA [NM_006351]	**2.154**
ARFGAP2	Homo sapiens ADP-ribosylation factor GTPase activating protein 2 (ARFGAP2), mRNA [NM_032389]	**2.150**
CPA4	Homo sapiens carboxypeptidase A4 (CPA4), mRNA [NM_016352]	**2.145**
CEBPE	Homo sapiens CCAAT/enhancer binding protein (C/EBP), epsilon (CEBPE), mRNA [NM_001805]	**2.138**
ALKBH5	Homo sapiens alkB, alkylation repair homolog 5 (E. coli) (ALKBH5), mRNA [NM_017758]	**2.138**
FXR2	Homo sapiens fragile X mental retardation, autosomal homolog 2 (FXR2), mRNA [NM_004860]	**2.133**
CDIPT	Homo sapiens CDP-diacylglycerol--inositol 3-phosphatidyltransferase (phosphatidylinositol synthase) (CDIPT), mRNA [NM_006319]	**2.129**
PDE2A	Homo sapiens phosphodiesterase 2A, cGMP-stimulated (PDE2A),mRNA [NM_002599]	**2.117**
LIMD1	Homo sapiens LIM domains containing 1 (LIMD1), mRNA [NM_014240]	**2.108**
FTHL17	Homo sapiens ferritin, heavy polypeptide-like 17 (FTHL17), mRNA [NM_031894]	**2.106**
ZNF33B	Homo sapiens zinc finger protein 33B (ZNF33B), mRNA [NM_006955]	**2.103**
TM7SF2	Homo sapiens transmembrane 7 superfamily member 2 (TM7SF2), mRNA [NM_003273]	**2.100**
PLA2G3	Homo sapiens phospholipase A2, group III (PLA2G3), mRNA [NM_015715]	**2.093**
IDI1	Homo sapiens isopentenyl-diphosphate delta isomerase 1 (IDI1), mRNA [NM_004508]	**2.089**
PGD	Homo sapiens phosphogluconate dehydrogenase (PGD), mRNA [NM_002631]	**2.081**
ADAMTS17	Homo sapiens ADAM metallopeptidase with thrombospondin type 1 motif, 17 (ADAMTS17), mRNA [NM_139057]	**2.075**
EGLN2	Homo sapiens egl nine homolog 2 (C. elegans) (EGLN2), mRNA [NM_080732]	**2.075**
POR	Homo sapiens P450 (cytochrome) oxidoreductase (POR), mRNA [NM_000941]	**2.073**
TUBB4	Homo sapiens tubulin, beta 4 (TUBB4), mRNA [NM_006087]	**2.073**
TBL3	Homo sapiens transducin (beta)-like 3 (TBL3), mRNA [NM_006453]	**2.070**
PICK1	Homo sapiens protein interacting with PRKCA 1 (PICK1),mRNA [NM_012407]	**2.069**

HMGCS1	Homo sapiens 3-hydroxy-3-methylglutaryl-Coenzyme A synthase 1 (soluble) (HMGCS1), mRNA [NM_002130]	**2.061**
DNASE2	Homo sapiens deoxyribonuclease II, lysosomal (DNASE2), mRNA [NM_001375]	**2.059**
IFIT3	Homo sapiens interferon-induced protein with tetratricopeptide repeats 3 (IFIT3), mRNA [NM_001549]	**2.058**
TRIM16L	Homo sapiens tripartite motif-containing 16-like (TRIM16L), mRNA [NM_001037330]	**2.058**
CYP51A1	Homo sapiens cytochrome P450, family 51, subfamily A, polypeptide 1 (CYP51A1), mRNA [NM_000786]	**2.056**
BAP1	Homo sapiens BRCA1 associated protein-1 (ubiquitin carboxy-terminal hydrolase) (BAP1), mRNA [NM_004656]	**2.049**
LOC392787	PREDICTED: Homo sapiens misc_RNA (LOC392787), miscRNA [XR_018580]	**2.048**
SLC1A5	Homo sapiens solute carrier family 1 (neutral amino acid transporter), member 5 (SLC1A5), mRNA [NM_005628]	**2.046**
USP5	Homo sapiens ubiquitin specific peptidase 5 (isopeptidase T) (USP5), mRNA [NM_003481]	**2.045**
TUBA1A	Homo sapiens tubulin, alpha 1a (TUBA1A), mRNA [NM_006009]	**2.035**
SMARCD2	Homo sapiens SWI/SNF related, matrix associated, actin dependent regulator of chromatin, subfamily d, member 2 (SMARCD2), mRNA [NM_003077]	**2.031**
PACSIN3	Homo sapiens protein kinase C and casein kinase substrate in neurons 3 (PACSIN3), mRNA [NM_016223]	**2.029**
LOC646048	PREDICTED: Homo sapiens similar to cytoskeletal beta actin (LOC646048), mRNA [XR_017059]	**2.028**
ETV5	Homo sapiens ets variant 5 (ETV5), mRNA [NM_004454]	**2.023**
CLCN7	Homo sapiens chloride channel 7 (CLCN7), mRNA [NM_001287]	**2.021**
UAP1L1	Homo sapiens UDP-N-acteylglucosamine pyrophosphorylase 1-like 1 (UAP1L1), mRNA [NM_207309]	**2.020**
HDHD3	Homo sapiens haloacid dehalogenase-like hydrolase domain containing 3 (HDHD3), mRNA [NM_031219]	**2.014**
CCND1	Homo sapiens cyclin D1 (CCND1), mRNA [NM_053056]	**2.012**
APP	Homo sapiens amyloid beta (A4) precursor protein (APP), mRNA [NM_000484]	**2.004**
PLCB3	Homo sapiens phospholipase C, beta 3 (phosphatidylinositol-specific) (PLCB3), mRNA [NM_000932]	**2.003**
GALNT2	Homo sapiens UDP-N-acetyl-alpha-D-galactosamine: polypeptide N-acetylgalactosaminyltransferase 2 (GalNAc-T2), mRNA [NM_004481]	**2.001**
AGPAT2	Homo sapiens 1-acylglycerol-3-phosphate O-acyltransferase 2 (lysophosphatidic acid acyltransferase, beta) (AGPAT2), mRNA [NM_006412]	**2.000**
NSDHL	Homo sapiens NAD(P) dependent steroid dehydrogenase-like (NSDHL), mRNA [NM_015922]	**2.000**
CSK	Homo sapiens c-src tyrosine kinase (CSK), mRNA [NM_004383]	**1.998**
TOM1	Homo sapiens target of myb1 (chicken) (TOM1), mRNA [NM_005488]	**1.995**
FOLR3	Homo sapiens folate receptor 3 (gamma) (FOLR3), mRNA [NM_000804]	**1.994**
G6PD	Homo sapiens glucose-6-phosphate dehydrogenase (G6PD), mRNA	**1.992**

	[NM_000402]	
CYB5B	Homo sapiens cytochrome b5 type B (outer mitochondrial membrane) (CYB5B), mRNA [NM_030579]	**1.991**
INSIG1	Homo sapiens insulin induced gene 1 (INSIG1), mRNA [NM_198336]	**1.991**
RANGAP1	Homo sapiens Ran GTPase activating protein 1 (RANGAP1), mRNA [NM_002883]	**1.985**
SLC27A4	Homo sapiens solute carrier family 27 (fatty acid transporter), member 4 (SLC27A4), mRNA [NM_005094]	**1.983**
HLA-H	Homo sapiens major histocompatibility complex, class I, H (pseudogene) (HLA-H), non-coding RNA [NR_001434]	**1.979**
TNIP2	Homo sapiens TNFAIP3 interacting protein 2 (TNIP2), mRNA [NM_024309]	**1.977**
GNPDA1	Homo sapiens glucosamine-6-phosphate deaminase 1 (GNPDA1), mRNA [NM_005471]	**1.967**
SFN	Homo sapiens stratifin (SFN), mRNA [NM_006142]	**1.964**
ANKRD1	Homo sapiens ankyrin repeat domain 1 (cardiac muscle) (ANKRD1), mRNA [NM_014391]	**1.962**
PTGES	Homo sapiens prostaglandin E synthase (PTGES), mRNA [NM_004878]	**1.961**
TMEM115	Homo sapiens transmembrane protein 115 (TMEM115), mRNA [NM_007024]	**1.956**
C1orf128	Homo sapiens chromosome 1 open reading frame 128 (C1orf128), mRNA [NM_020362]	**1.954**
ELK1	Homo sapiens ELK1, member of ETS oncogene family (ELK1), mRNA [NM_005229]	**1.951**
MAP2K2	Homo sapiens mitogen-activated protein kinase kinase 2 (MAP2K2), mRNA [NM_030662]	**1.950**
TMEM97	Homo sapiens transmembrane protein 97 (TMEM97), mRNA [NM_014573]	**1.948**
LDLR	Homo sapiens low density lipoprotein receptor (LDLR), mRNA [NM_000527]	**1.944**
ATAD3B	Homo sapiens ATPase family, AAA domain containing 3B (ATAD3B), mRNA [NM_031921]	**1.943**
GREB1	Homo sapiens GREB1 protein (GREB1), transcript variant c, mRNA [NM_148903]	**1.943**
IER5L	Homo sapiens immediate early response 5-like (IER5L), mRNA [NM_203434]	**1.943**
RHEBL1	Homo sapiens Ras homolog enriched in brain like 1 (RHEBL1), mRNA [NM_144593]	**1.940**
GIPC1	Homo sapiens GIPC PDZ domain containing family, member 1 (GIPC1), mRNA [NM_005716]	**1.938**
GDF15	Homo sapiens growth differentiation factor 15 (GDF15), mRNA [NM_004864]	**1.936**
FLNC	Homo sapiens filamin C, gamma (actin binding protein 280) (FLNC),mRNA [NM_001458]	**1.933**
LOC100131929	Homo sapiens cDNA FLJ44475 fis, clone UTERU2031521. [AK126439]	**1.933**
LOC399881	Homo sapiens cDNA FLJ44864 fis, clone BRALZ2013621, [AK126814]	**1.931**
EHBP1L1	Homo sapiens EH domain binding protein 1-like 1 (EHBP1L1), mRNA [NM_001099409]	**1.923**
ALDOAP2	Human aldolase pseudogene mRNA, [M21191]	**1.921**

CREB3	Homo sapiens cAMP responsive element binding protein 3 (CREB3), mRNA [NM_006368]	**1.909**
AAMP	Homo sapiens angio-associated, migratory cell protein (AAMP), mRNA [NM_001087]	**1.908**
PLEKHM2	Homo sapiens pleckstrin homology domain containing, family M (with RUN domain) member 2 (PLEKHM2), mRNA [NM_015164]	**1.908**
ACLY	Human ATP:citrate lyase mRNA, complete cds. [U18197]	**1.907**
USP19	Homo sapiens ubiquitin specific peptidase 19 (USP19), mRNA [NM_006677]	**1.907**
GSR	Homo sapiens glutathione reductase (GSR), mRNA [NM_000637]	**1.904**
PSMD2	Homo sapiens proteasome (prosome, macropain) 26S subunit, 2 (PSMD2), mRNA [NM_002808]	**1.901**
LOC100134282	Homo sapiens cDNA FLJ27195 fis, clone SYN02786. [AK130705]	**1.899**
PPM2C	Homo sapiens protein phosphatase 2C, magnesium-dependent, catalytic subunit (PPM2C), mRNA [NM_018444]	**1.899**
POLR2E	Homo sapiens polymerase (RNA) II (DNA directed) polypeptide E, (POLR2E), mRNA [NM_002695]	**1.897**
MAF1	Homo sapiens MAF1 homolog (S. cerevisiae) (MAF1), mRNA [NM_032272]	**1.894**
MGC23284	Homo sapiens hypothetical LOC197187 (MGC23284), non-coding RNA [NR_024399]	**1.894**
ACTBL2	Homo sapiens actin, beta-like 2 (ACTBL2), mRNA [NM_001017992]	**1.893**
NAP1L4	Homo sapiens nucleosome assembly protein 1-like 4 (NAP1L4), mRNA [NM_005969]	**1.892**
CYTH2	Homo sapiens cytohesin 2 (CYTH2), mRNA [NM_004228]	**1.888**
TMEM55B	Homo sapiens transmembrane protein 55B (TMEM55B), mRNA [NM_144568]	**1.888**
CLDN4	Homo sapiens claudin 4 (CLDN4), mRNA [NM_001305]	**1.885**
TEF	Homo sapiens thyrotrophic embryonic factor (TEF), mRNA [NM_003216]	**1.883**
THAP7	Homo sapiens THAP domain containing 7 (THAP7), mRNA [NM_030573]	**1.877**
YARS	Homo sapiens tyrosyl-tRNA synthetase (YARS), mRNA [NM_003680]	**1.876**
EEF2	Homo sapiens eukaryotic translation elongation factor 2 (EEF2), mRNA [NM_001961]	**1.875**
GAPDH	Homo sapiens glyceraldehyde-3-phosphate dehydrogenase (GAPDH), mRNA [NM_002046]	**1.869**
TMEM156	Homo sapiens transmembrane protein 156 (TMEM156), mRNA [NM_024943]	**1.865**
ACTN1	Homo sapiens actinin, alpha 1 (ACTN1), mRNA [NM_001102]	**1.863**
SLC39A1	Homo sapiens solute carrier family 39 (zinc transporter), member 1 (SLC39A1), mRNA [NM_014437]	**1.861**
ASPSCR1	Homo sapiens alveolar soft part sarcoma chromosome region, candidate 1 (ASPSCR1), mRNA [NM_024083]	**1.856**
RAB8A	Homo sapiens RAB8A, member RAS oncogene family (RAB8A), mRNA [NM_005370]	**1.855**
RASA3	Homo sapiens mRNA for Ins(1,3,4,5)P4-binding protein. [X89399]	**1.854**
CTSD	Homo sapiens cathepsin D (CTSD), mRNA [NM_001909]	**1.848**
SLC2A6	Homo sapiens solute carrier family 2 (facilitated glucose transporter), member 6	**1.847**

	(SLC2A6), mRNA [NM_017585]	
SPP1	Homo sapiens secreted phosphoprotein 1 (SPP1), mRNA [NM_001040058]	**1.844**
LOC220729	Homo sapiens succinate dehydrogenase complex, subunit A, flavoprotein pseudogene (LOC220729), non-coding RNA [NR_003266]	**1.841**
FLNA	Homo sapiens filamin A, alpha (actin binding protein 280) (FLNA), mRNA [NM_001456]	**1.840**
ZNF76	Homo sapiens zinc finger protein 76 (expressed in testis) (ZNF76), mRNA [NM_003427]	**1.833**
UNC119	Homo sapiens unc-119 homolog (C. elegans) (UNC119), mRNA [NM_005148]	**1.832**
TBC1D2	Homo sapiens TBC1 domain family, member 2 (TBC1D2), mRNA [NM_018421]	**1.826**
HABP4	Homo sapiens hyaluronan binding protein 4 (HABP4), mRNA [NM_014282]	**1.824**
UPP1	Homo sapiens uridine phosphorylase 1 (UPP1), mRNA [NM_181597]	**1.819**
GGT8P	Homo sapiens gamma-glutamyltransferase 8 pseudogene (GGT8P), non-coding RNA [NR_003503]	**1.815**
KLHDC4	Homo sapiens kelch domain containing 4 (KLHDC4), mRNA [NM_017566]	**1.813**
SEC24C	Homo sapiens SEC24 family, member C (S. cerevisiae) (SEC24C), mRNA [NM_004922]	**1.809**
DUSP5	Homo sapiens dual specificity phosphatase 5 (DUSP5), mRNA [NM_004419]	**1.806**
RAB40C	Homo sapiens RAB40C, member RAS oncogene family (RAB40C), mRNA [NM_021168]	**1.806**
PEF1	Homo sapiens penta-EF-hand domain containing 1 (PEF1), mRNA [NM_012392]	**1.804**
HDGF2	Homo sapiens hepatoma-derived growth factor-related protein 2 (HDGF2), mRNA [NM_032631]	**1.799**
STK32C	Homo sapiens serine/threonine kinase 32C (STK32C), mRNA [NM_173575]	**1.798**
NRBP1	Homo sapiens nuclear receptor binding protein 1 (NRBP1), mRNA [NM_013392]	**1.797**
RUVBL2	Homo sapiens RuvB-like 2 (E. coli) (RUVBL2), mRNA [NM_006666]	**1.797**
MVK	Homo sapiens mevalonate kinase (MVK), mRNA [NM_000431]	**1.790**
SLC7A11	Homo sapiens solute carrier family 7, (cationic amino acid transporter, y+ system) member 11 (SLC7A11), mRNA [NM_014331]	**1.787**
NUMA1	Homo sapiens nuclear mitotic apparatus protein 1 (NUMA1), mRNA [NM_006185]	**1.784**
STARD3	Homo sapiens StAR-related lipid transfer (START) domain containing 3 (STARD3), mRNA [NM_006804]	**1.781**
NEIL2	Homo sapiens nei like 2 (E. coli) (NEIL2), mRNA [NM_145043]	**1.777**
TOMM40	Homo sapiens translocase of outer mitochondrial membrane 40 homolog (yeast) (TOMM40), mRNA [NM_006114]	**1.776**
SERPINE1	Homo sapiens serpin peptidase inhibitor, clade E (nexin, plasminogen activator inhibitor type 1), member 1 (SERPINE1), mRNA [NM_000602]	**1.766**
TRPM2	Homo sapiens transient receptor potential cation channel, subfamily M, member 2 (TRPM2), mRNA [NM_003307]	**1.766**
SPNS1	Homo sapiens spinster homolog 1 (Drosophila) (SPNS1), mRNA [NM_032038]	**1.765**
ZBED1	Homo sapiens zinc finger, BED-type containing 1 (ZBED1), mRNA [NM_004729]	**1.764**

GBAP	Homo sapiens glucosidase, beta; acid, pseudogene (GBAP), non-coding RNA [NR_002188]	**1.763**
ORAI2	Homo sapiens ORAI calcium release-activated calcium modulator 2 (ORAI2), mRNA [NM_032831]	**1.760**
AXIN1	Homo sapiens axin 1 (AXIN1), mRNA [NM_003502]	**1.753**
ORM1	Homo sapiens orosomucoid 1 (ORM1), mRNA [NM_000607]	**1.753**
ATG2A	Homo sapiens ATG2 autophagy related 2 homolog A (S. cerevisiae) (ATG2A), mRNA [NM_015104]	**1.751**
ZNF768	Homo sapiens zinc finger protein 768 (ZNF768), mRNA [NM_024671]	**1.751**
HSP90AB1	Homo sapiens heat shock protein alpha (cytosolic), class B member 1 (HSP90AB1), mRNA [NM_007355]	**1.746**
NBPF20	Homo sapiens neuroblastoma breakpoint family, member 20 (NBPF20), mRNA [NM_001037675]	**1.745**
MAPK12	Homo sapiens mitogen-activated protein kinase 12 (MAPK12), mRNA [NM_002969]	**1.743**
FLJ46111	Homo sapiens FLJ46111 protein, mRNA (cDNA clone IMAGE:8327474). [BC112006]	**1.742**
SPTBN1	Homo sapiens spectrin, beta, non-erythrocytic 1 (SPTBN1), mRNA [NM_003128]	**1.740**
IGFN1	Homo sapiens immunoglobulin-like and fibronectin type III domain containing 1 (IGFN1), mRNA [NM_178275]	**1.736**
CHD4	Homo sapiens chromodomain helicase DNA binding protein 4 (CHD4), mRNA [NM_001273]	**1.732**
ADRM1	Homo sapiens adhesion regulating molecule 1 (ADRM1), mRNA [NM_007002]	**1.731**
DOHH	Homo sapiens deoxyhypusine hydroxylase/monooxygenase (DOHH), mRNA [NM_031304]	**1.731**
APH1A	Homo sapiens anterior pharynx defective 1 homolog A (C. elegans) (APH1A), mRNA [NM_001077628]	**1.726**
LOC147804	Homo sapiens tropomyosin 3 pseudogene (LOC147804), non-coding RNA [NR_003148]	**1.725**
AFG3L2	Homo sapiens AFG3 ATPase family gene 3-like 2 (yeast) (AFG3L2), mRNA [NM_006796]	**1.718**
KRT17P3	PREDICTED: Homo sapiens misc_RNA (KRT17P3), miscRNA [XR_019109]	**1.716**
AGPAT6	Homo sapiens 1-acylglycerol-3-phosphate O-acyltransferase 6 (lysophosphatidic acid acyltransferase, zeta) (AGPAT6), mRNA [NM_178819]	**1.716**
KIAA0415	Homo sapiens KIAA0415 (KIAA0415), mRNA [NM_014855]	**1.713**
IFI35	Homo sapiens interferon-induced protein 35 (IFI35), mRNA [NM_005533]	**1.711**
FDPSL2A	Homo sapiens MGC44478 (FDPSL2A), non-coding RNA [NR_003262]	**1.707**
GPC1	Homo sapiens glypican 1 (GPC1), mRNA [NM_002081]	**1.705**
ADAM15	Homo sapiens ADAM metallopeptidase domain 15 (ADAM15),mRNA [NM_207191]	**1.703**
QARS	Homo sapiens glutaminyl-tRNA synthetase (QARS), mRNA [NM_005051]	**1.701**
H1FX	Homo sapiens H1 histone family, member X (H1FX), mRNA [NM_006026]	**1.700**

MCOLN1	Homo sapiens mucolipin 1 (MCOLN1), mRNA [NM_020533]	**1.698**
DDX42	Homo sapiens DEAD (Asp-Glu-Ala-Asp) box polypeptide 42 (DDX42), mRNA [NM_007372]	**1.694**
FASN	Homo sapiens fatty acid synthase (FASN), mRNA [NM_004104]	**1.694**
IL17RC	Homo sapiens interleukin 17 receptor C (IL17RC), mRNA [NM_153461]	**1.688**
HSP90AB3P	Homo sapiens heat shock protein 90Bc (HSP90Bc) mRNA, [AY956764]	**1.684**
ACIN1	Homo sapiens apoptotic chromatin condensation inducer 1 (ACIN1), mRNA [NM_014977]	**1.681**
DLSTP	E2k=alpha-ketoglutarate dehydrogenase complex dihydrolipoyl succinyltransferase [human, fetal brain, mRNA, 2987 nt]. [S72422]	**1.681**
GPR175	Homo sapiens G protein-coupled receptor 175 (GPR175), mRNA [NM_016372]	**1.679**
RUSC2	Homo sapiens RUN and SH3 domain containing 2 (RUSC2), mRNA [NM_014806]	**1.677**
SLC4A2	Homo sapiens solute carrier family 4, anion exchanger, member 2 (erythrocyte membrane protein band 3-like 1) (SLC4A2), mRNA [NM_003040]	**1.674**
HPCAL1	Homo sapiens hippocalcin-like 1 (HPCAL1), mRNA [NM_134421]	**1.670**
PABPC4	Homo sapiens poly(A) binding protein, cytoplasmic 4 (inducible form) (PABPC4), mRNA [NM_003819]	**1.670**
MIDN	Homo sapiens midnolin (MIDN), mRNA [NM_177401]	**1.667**
RUNDC2C	Homo sapiens RUN domain containing 2C (RUNDC2C), non-coding RNA [NR_002939]	**1.667**
FTH1	Homo sapiens ferritin, heavy polypeptide 1 (FTH1), mRNA [NM_002032]	**1.666**
CLEC2B	Homo sapiens C-type lectin domain family 2, member B (CLEC2B), mRNA [NM_005127]	**1.662**
LOC401717	PREDICTED: Homo sapiens misc_RNA (LOC401717),miscRNA [XR_018189]	**1.661**
NUBP2	Homo sapiens nucleotide binding protein 2 (MinD homolog, E. coli) (NUBP2), mRNA [NM_012225]	**1.661**
CHMP1A	Homo sapiens chromatin modifying protein 1A (CHMP1A), mRNA [NM_002768]	**1.661**
RRBP1	Homo sapiens ribosome binding protein 1 homolog 180kDa (dog) (RRBP1), mRNA [NM_001042576]	**1.658**
AP1B1	Homo sapiens adaptor-related protein complex 1, beta 1 subunit (AP1B1), mRNA [NM_001127]	**1.655**
GALE	Homo sapiens UDP-galactose-4-epimerase (GALE), mRNA [NM_000403]	**1.655**
TMEM144	Homo sapiens transmembrane protein 144 (TMEM144), mRNA [NM_018342]	**1.654**
TNFRSF10A	Homo sapiens tumor necrosis factor receptor superfamily, member 10a (TNFRSF10A), mRNA [NM_003844]	**1.654**
RAB4B	Homo sapiens RAB4B, member RAS oncogene family (RAB4B), mRNA [NM_016154]	**1.651**
NEU1	Homo sapiens sialidase 1 (lysosomal sialidase) (NEU1), mRNA [NM_000434]	**1.650**
RAG1AP1	Homo sapiens recombination activating gene 1 activating protein 1 (RAG1AP1), mRNA [NM_018845]	**1.650**
ARAF	Homo sapiens v-raf murine sarcoma 3611 viral oncogene homolog (ARAF),	**1.648**

	mRNA [NM_001654]	
CDKN1A	Homo sapiens cyclin-dependent kinase inhibitor 1A (p21, Cip1) (CDKN1A), mRNA [NM_000389]	**1.646**
GPAA1	Homo sapiens glycosylphosphatidylinositol anchor attachment protein 1 homolog (yeast) (GPAA1), mRNA [NM_003801]	**1.645**
ALDH3A1	Homo sapiens aldehyde dehydrogenase 3 family, memberA1 (ALDH3A1), mRNA [NM_000691]	**1.643**
TPCN2	Homo sapiens two pore segment channel 2 (TPCN2), mRNA [NM_139075]	**1.642**
ASH1L	Homo sapiens ash1 (absent, small, or homeotic)-like (Drosophila) (ASH1L), mRNA [NM_018489]	**1.641**
IRAK1	Homo sapiens interleukin-1 receptor-associated kinase 1 (IRAK1),mRNA [NM_001569]	**1.641**
hCG_1641703	Putative uncharacterized protein ENSP00000383883 (HCG1641703) [Source:UniProtKB/TrEMBL;Acc:B5MBZ2] [ENST00000401937]	**1.640**
TGFBRAP1	Homo sapiens transforming growth factor, beta receptor associated protein 1 (TGFBRAP1), mRNA [NM_004257]	**1.640**
C1S	Homo sapiens complement component 1, s subcomponent (C1S), mRNA [NM_001734]	**1.639**
VARS	Homo sapiens valyl-tRNA synthetase (VARS), mRNA [NM_006295]	**1.639**
SPANXD	Homo sapiens SPANX family, member D (SPANXD), mRNA [NM_032417]	**1.634**
CCDC22	Homo sapiens coiled-coil domain containing 22 (CCDC22), mRNA [NM_014008]	**1.633**
LOC148709	Homo sapiens actin pseudogene (LOC148709), non-coding RNA [NR_002929]	**1.633**
PPP5C	Homo sapiens protein phosphatase 5, catalytic subunit (PPP5C), mRNA [NM_006247]	**1.629**
NUP214	Homo sapiens nucleoporin 214kDa (NUP214), mRNA [NM_005085]	**1.628**
LTBR	Homo sapiens lymphotoxin beta receptor (TNFR superfamily, member 3) (LTBR), mRNA [NM_002342]	**1.627**
RPS6KA1	Homo sapiens ribosomal protein S6 kinase, 90kDa, polypeptide 1 (RPS6KA1), mRNA [NM_002953]	**1.625**
PPP4C	Homo sapiens protein phosphatase 4 (formerly X), catalytic subunit (PPP4C), mRNA [NM_002720]	**1.624**
INTS5	Homo sapiens integrator complex subunit 5 (INTS5), mRNA [NM_030628]	**1.622**
CCDC94	Homo sapiens coiled-coil domain containing 94 (CCDC94), mRNA [NM_018074]	**1.619**
CSF2RA	Homo sapiens colony stimulating factor 2 receptor, alpha, low-affinity (granulocyte-macrophage) (CSF2RA), mRNA [NM_172247]	**1.618**
KIF7	Homo sapiens kinesin family member 7 (KIF7), mRNA [NM_198525]	**1.615**
HNRNPAB	Homo sapiens heterogeneous nuclear ribonucleoprotein A/B (HNRNPAB), mRNA [NM_004499]	**1.614**
F2RL2	Homo sapiens coagulation factor II (thrombin) receptor-like 2 (F2RL2), mRNA [NM_004101]	**1.613**
PSG9	Homo sapiens pregnancy specific beta-1-glycoprotein 9 (PSG9), mRNA	**1.611**

	[NM_002784]	
RDH13	Homo sapiens retinol dehydrogenase 13 (all-trans/9-cis) (RDH13), mRNA [NM_138412]	**1.609**
IRF1	Homo sapiens interferon regulatory factor 1 (IRF1), mRNA [NM_002198]	**1.608**
KCTD17	Homo sapiens potassium channel tetramerisation domain containing 17 (KCTD17), mRNA [NM_024681]	**1.607**
TTC4	Homo sapiens tetratricopeptide repeat domain 4 (TTC4), mRNA [NM_004623]	**1.606**
SKIV2L	Homo sapiens superkiller viralicidic activity 2-like (S. cerevisiae) (SKIV2L), mRNA [NM_006929]	**1.605**
ASCC2	Homo sapiens activating signal cointegrator 1 complex subunit 2 (ASCC2), mRNA [NM_032204]	**1.604**
DVL2	Homo sapiens dishevelled, dsh homolog 2 (Drosophila) (DVL2), mRNA [NM_004422]	**1.604**
FOXO3	Homo sapiens forkhead box O3 (FOXO3), mRNA [NM_001455]	**1.604**
NR2F6	Homo sapiens nuclear receptor subfamily 2, group F, member 6 (NR2F6), mRNA [NM_005234]	**1.603**
SDHALP1	Homo sapiens succinate dehydrogenase complex, subunit A, flavoprotein pseudogene 1 (SDHALP1), non-coding RNA [NR_003264]	**1.601**
IL6R	Homo sapiens interleukin 6 receptor (IL6R), mRNA [NM_000565]	**1.599**
SH2D5	Homo sapiens SH2 domain containing 5 (SH2D5), mRNA [NM_001103161]	**1.599**
ECE1	Homo sapiens endothelin converting enzyme 1 (ECE1), mRNA [NM_001397]	**1.598**
IGHMBP2	Homo sapiens immunoglobulin mu binding protein 2 (IGHMBP2), mRNA [NM_002180]	**1.592**
PHLDA3	Homo sapiens pleckstrin homology-like domain, family A, member 3 (PHLDA3), mRNA [NM_012396]	**1.592**
SLC35C2	Homo sapiens solute carrier family 35, member C2 (SLC35C2),mRNA [NM_173179]	**1.592**
SCML1	Homo sapiens sex comb on midleg-like 1 (Drosophila) (SCML1),mRNA [NM_001037540]	**1.590**
C7orf57	Homo sapiens chromosome 7 open reading frame 57 (C7orf57), mRNA [NM_001100159]	**1.588**
NBPF10	Homo sapiens neuroblastoma breakpoint family, member 10 (NBPF10), mRNA [NM_001039703]	**1.587**
GMPPB	Homo sapiens GDP-mannose pyrophosphorylase B (GMPPB), mRNA [NM_021971]	**1.586**
LOC729497	PREDICTED: Homo sapiens misc_RNA (LOC729497), miscRNA [XR_015501]	**1.585**
LOC400036	PREDICTED: Homo sapiens misc_RNA (LOC400036), miscRNA [XR_042386]	**1.584**
CHD3	Homo sapiens chromodomain helicase DNA binding protein 3 (CHD3), mRNA [NM_001005273]	**1.583**
JUN	Homo sapiens jun oncogene (JUN), mRNA [NM_002228]	**1.583**
EWSR1	Homo sapiens Ewing sarcoma breakpoint region 1 (EWSR1), mRNA [NM_013986]	**1.583**
LOC441016	PREDICTED: Homo sapiens hypothetical LOC441016 (LOC441016), mRNA	**1.582**

	[XM_001714867]	
EEF1A1	Homo sapiens eukaryotic translation elongation factor 1 alpha 1 (EEF1A1), mRNA [NM_001402]	**1.581**
SNX17	Homo sapiens sorting nexin 17 (SNX17), mRNA [NM_014748]	**1.581**
ACTB	Homo sapiens actin, beta (ACTB), mRNA [NM_001101]	**1.579**
ALKBH6	Homo sapiens alkB, alkylation repair homolog 6 (E. coli) (ALKBH6), mRNA [NM_198867]	**1.578**
TUBG1	Homo sapiens tubulin, gamma 1 (TUBG1), mRNA [NM_001070]	**1.578**
C21orf56	Homo sapiens chromosome 21 open reading frame 56 (C21orf56), mRNA [NM_032261]	**1.576**
PPP1CA	Homo sapiens protein phosphatase 1, catalytic subunit, alpha isoform (PPP1CA), mRNA [NM_001008709]	**1.575**
CCDC137	Homo sapiens coiled-coil domain containing 137 (CCDC137), mRNA [NM_199287]	**1.575**
METRN	Homo sapiens meteorin, glial cell differentiation regulator (METRN), mRNA [NM_024042]	**1.575**
DHX38	Homo sapiens DEAH (Asp-Glu-Ala-His) box polypeptide 38 (DHX38), mRNA [NM_014003]	**1.574**
FDFT1	Homo sapiens farnesyl-diphosphate farnesyltransferase 1 (FDFT1), mRNA [NM_004462]	**1.574**
LOC401010	Homo sapiens nucleolar complex associated 2 homolog (S. cerevisiae) pseudogene (LOC401010), non-coding RNA [NR_002826]	**1.573**
WDR24	Homo sapiens WD repeat domain 24 (WDR24), mRNA [NM_032259]	**1.573**
C19orf48	Homo sapiens chromosome 19 open reading frame 48 (C19orf48), mRNA [NM_199249]	**1.569**
FLJ40113	Homo sapiens golgi autoantigen, golgin subfamily a-like pseudogene (FLJ40113), non-coding RNA [NR_003246]	**1.568**
IDH1	Homo sapiens isocitrate dehydrogenase 1 (NADP+), soluble (IDH1), mRNA [NM_005896]	**1.568**
LOC391334	PREDICTED: Homo sapiens misc_RNA (LOC391334), miscRNA [XR_017601]	**1.568**
PLOD1	Homo sapiens procollagen-lysine 1, 2-oxoglutarate 5-dioxygenase 1 (PLOD1), mRNA [NM_000302]	**1.567**
TWF2	Homo sapiens twinfilin, actin-binding protein, homolog 2 (Drosophila) (TWF2), mRNA [NM_007284]	**1.567**
PES1	Homo sapiens pescadillo homolog 1, containing BRCT domain (zebrafish) (PES1), mRNA [NM_014303]	**1.566**
PPAP2C	Homo sapiens phosphatidic acid phosphatase type 2C (PPAP2C), mRNA [NM_177543]	**1.566**
ZNF787	Homo sapiens zinc finger protein 787 (ZNF787), mRNA [NM_001002836]	**1.565**
ALDOC	Homo sapiens aldolase C, fructose-bisphosphate (ALDOC), mRNA [NM_005165]	**1.564**
SC4MOL	Homo sapiens sterol-C4-methyl oxidase-like (SC4MOL), mRNA [NM_006745]	**1.560**
NXN	Homo sapiens nucleoredoxin (NXN), mRNA [NM_022463]	**1.559**

TOP3B	Homo sapiens topoisomerase (DNA) III beta (TOP3B), mRNA [NM_003935]	**1.559**
TRMT2B	Homo sapiens TRM2 tRNA methyltransferase 2 homolog B (S. cerevisiae) (TRMT2B), mRNA [NM_024917]	**1.558**
DSG2	Homo sapiens desmoglein 2 (DSG2), mRNA [NM_001943]	**1.557**
PEX16	Homo sapiens peroxisomal biogenesis factor 16 (PEX16), mRNA [NM_004813]	**1.557**
ALAS1	Homo sapiens aminolevulinate, delta-, synthase 1 (ALAS1), mRNA [NM_000688]	**1.557**
FAM115A	Homo sapiens family with sequence similarity 115, member A (FAM115A), mRNA [NM_014719]	**1.557**
HSD17B7	Homo sapiens hydroxysteroid (17-beta) dehydrogenase 7 (HSD17B7), mRNA [NM_016371]	**1.555**
RRP7A	Homo sapiens ribosomal RNA processing 7 homolog A (S. cerevisiae), mRNA (cDNA clone IMAGE:4394667), [BC031838]	**1.555**
CDC37	Homo sapiens cell division cycle 37 homolog (S. cerevisiae) (CDC37), mRNA [NM_007065]	**1.552**
PDP2	Homo sapiens pyruvate dehydrogenase phosphatase isoenzyme 2 (PDP2), mRNA [NM_020786]	**1.551**
NOSIP	Homo sapiens nitric oxide synthase interacting protein (NOSIP), mRNA [NM_015953]	**1.551**
ANKRD9	Homo sapiens ankyrin repeat domain 9 (ANKRD9), mRNA [NM_152326]	**1.550**
CRK	Homo sapiens v-crk sarcoma virus CT10 oncogene homolog (avian) (CRK), mRNA [NM_016823]	**1.549**
LPCAT3	Homo sapiens lysophosphatidylcholine acyltransferase 3 (LPCAT3), mRNA [NM_005768]	**1.547**
C16orf42	Homo sapiens chromosome 16 open reading frame 42 (C16orf42), mRNA [NM_001001410]	**1.546**
MAP4	Homo sapiens microtubule-associated protein 4 (MAP4), mRNA [NM_002375]	**1.546**
KIF26A	Homo sapiens kinesin family member 26A (KIF26A), mRNA [NM_015656]	**1.543**
M6PR	Homo sapiens mannose-6-phosphate receptor (cation dependent) (M6PR), mRNA [NM_002355]	**1.542**
ARMC6	Homo sapiens armadillo repeat containing 6 (ARMC6), mRNA [NM_033415]	**1.541**
LSS	Homo sapiens lanosterol synthase (2,3-oxidosqualene-lanosterol cyclase) (LSS), mRNA [NM_001001438]	**1.540**
GNB2	Homo sapiens guanine nucleotide binding protein (G protein), beta polypeptide 2 (GNB2), mRNA [NM_005273]	**1.540**
ARRB1	Homo sapiens arrestin, beta 1 (ARRB1), mRNA [NM_004041]	**1.539**
AUP1	Homo sapiens ancient ubiquitous protein 1 (AUP1), mRNA [NM_181575]	**1.539**
CCDC85B	Homo sapiens coiled-coil domain containing 85B (CCDC85B), mRNA [NM_006848]	**1.539**
BHLHE40	Homo sapiens basic helix-loop-helix domain containing, class B, 2 (BHLHB2), mRNA [NM_003670]	**1.537**
HDGF	Homo sapiens hepatoma-derived growth factor (high-mobility group protein 1-like) (HDGF), mRNA [NM_004494]	**1.535**

SYNE2	Homo sapiens spectrin repeat containing, nuclear envelope 2 (SYNE2), mRNA [NM_182914]	**1.535**
CSRP1	Homo sapiens cysteine and glycine-rich protein 1 (CSRP1), mRNA [NM_004078]	**1.535**
STAT3	Homo sapiens signal transducer and activator of transcription 3 (acute-phase response factor) (STAT3), mRNA [NM_213662]	**1.534**
LPIN1	Homo sapiens lipin 1 (LPIN1), mRNA [NM_145693]	**1.533**
ACOT7	Homo sapiens acyl-CoA thioesterase 7 (ACOT7), mRNA [NM_007274]	**1.531**
TRPC4AP	Homo sapiens transient receptor potential cation channel, subfamily C, member 4 associated protein (TRPC4AP), mRNA [NM_015638]	**1.531**
DGCR6	Homo sapiens DiGeorge syndrome critical region gene 6 (DGCR6), mRNA [NM_005675]	**1.530**
LYPLA2	Homo sapiens lysophospholipase II (LYPLA2), mRNA [NM_007260]	**1.529**
DDIT3	Homo sapiens DNA-damage-inducible transcript 3 (DDIT3), mRNA [NM_004083]	**1.527**
BLVRB	Homo sapiens biliverdin reductase B (flavin reductase (NADPH)) (BLVRB), mRNA [NM_000713]	**1.524**
TNPO3	Homo sapiens transportin 3 (TNPO3), mRNA [NM_012470]	**1.524**
ASS1	Homo sapiens argininosuccinate synthetase 1 (ASS1), mRNA [NM_000050]	**1.523**
MSLN	Homo sapiens mesothelin (MSLN), mRNA [NM_005823]	**1.523**
LDLRAD3	Homo sapiens low density lipoprotein receptor class A domain containing 3 (LDLRAD3), mRNA [NM_174902]	**1.521**
CLN6	Homo sapiens ceroid-lipofuscinosis, neuronal 6, late infantile, mRNA [NM_017882]	**1.519**
DDX49	Homo sapiens DEAD (Asp-Glu-Ala-Asp) box polypeptide 49 (DDX49), mRNA [NM_019070]	**1.518**
DPP3	Homo sapiens dipeptidyl-peptidase 3 (DPP3), mRNA [NM_130443]	**1.518**
FOS	Homo sapiens v-fos FBJ murine osteosarcoma viral oncogene homolog (FOS), mRNA [NM_005252]	**1.517**
GEM	Homo sapiens GTP binding protein overexpressed in skeletal muscle (GEM), mRNA [NM_005261]	**1.516**
SAFB	Homo sapiens scaffold attachment factor B (SAFB), mRNA [NM_002967]	**1.516**
CAPZB	Homo sapiens capping protein (actin filament) muscle Z-line, beta (CAPZB), mRNA [NM_004930]	**1.514**
CCND3	Homo sapiens cyclin D3 (CCND3), mRNA [NM_001760]	**1.514**
PTGER1	Homo sapiens prostaglandin E receptor 1 (subtype EP1), (PTGER1), mRNA [NM_000955]	**1.513**
DAG1	Homo sapiens dystroglycan 1 (dystrophin-associated glycoprotein 1) (DAG1), mRNA [NM_004393]	**1.512**
NUPR1	Homo sapiens nuclear protein 1 (NUPR1), mRNA [NM_001042483]	**1.511**
STX3	Homo sapiens syntaxin 3 (STX3), mRNA [NM_004177]	**1.510**
RRP12	Homo sapiens ribosomal RNA processing 12 homolog (S. cerevisiae) (RRP12), mRNA [NM_015179]	**1.509**

HDLBP	Homo sapiens PRO2900 mRNA, complete cds. [AF116718]	**1.508**
BMP4	Homo sapiens bone morphogenetic protein 4 (BMP4), mRNA [NM_001202]	**1.506**
C1orf115	Homo sapiens chromosome 1 open reading frame 115 (C1orf115), mRNA [NM_024709]	**1.506**
FAM46B	Homo sapiens family with sequence similarity 46, member B (FAM46B), mRNA [NM_052943]	**1.505**
IGF2R	Homo sapiens insulin-like growth factor 2 receptor (IGF2R), mRNA [NM_000876]	**1.505**
RP3-402G11.5	Homo sapiens selenoprotein O (SELO), mRNA [NM_031454]	**1.504**
NGRN	Homo sapiens neugrin, neurite outgrowth associated (NGRN), mRNA [NM_001033088]	**1.504**
OSGIN1	Homo sapiens oxidative stress induced growth inhibitor 1 (OSGIN1), mRNA [NM_013370]	**1.501**
LAMA1	Homo sapiens laminin, alpha 1 (LAMA1), mRNA [NM_005559]	**1.498**
SLC38A5	Homo sapiens solute carrier family 38, member 5 (SLC38A5), mRNA [NM_033518]	**1.497**
PIK3C2A	Homo sapiens phosphoinositide-3-kinase, class 2, alpha polypeptide (PIK3C2A), mRNA [NM_002645]	**1.494**
HLA-A	Homo sapiens major histocompatibility complex, class I, A (HLA-A), mRNA [NM_002116]	**1.494**
PHGDH	Homo sapiens phosphoglycerate dehydrogenase (PHGDH), mRNA [NM_006623]	**1.493**
PGAM5	Homo sapiens phosphoglycerate mutase family member 5 (PGAM5), mRNA [NM_138575]	**1.492**
GNAI2	Homo sapiens guanine nucleotide binding protein (G protein), alpha inhibiting activity polypeptide 2 (GNAI2), mRNA [NM_002070]	**1.491**
TSR2	Homo sapiens TSR2, 20S rRNA accumulation, homolog (S. cerevisiae) (TSR2), mRNA [NM_058163]	**1.491**
MRFAP1	Homo sapiens Mof4 family associated protein 1 (MRFAP1), mRNA [NM_033296]	**1.491**
CARS	Homo sapiens cysteinyl-tRNA synthetase (CARS), mRNA [NM_001014438]	**1.489**
BAZ2A	Homo sapiens bromodomain adjacent to zinc finger domain, 2A (BAZ2A), mRNA [NM_013449]	**1.486**
HS1BP3	Homo sapiens HCLS1 binding protein 3, mRNA (cDNA clone IMAGE:5207261), [BC027947]	**1.485**
FEM1A	Homo sapiens fem-1 homolog a (C. elegans) (FEM1A), mRNA [NM_018708]	**1.485**
KIAA0664	Homo sapiens KIAA0664 (KIAA0664), mRNA [NM_015229]	**1.484**
PLEKHB2	Homo sapiens pleckstrin homology domain containing, family B (evectins) member 2 (PLEKHB2), mRNA [NM_017958]	**1.484**
FABP3	Homo sapiens fatty acid binding protein 3, (FABP3), mRNA [NM_004102]	**1.483**
KRT33A	Homo sapiens keratin 33A (KRT33A), mRNA [NM_004138]	**1.479**
FBXL19	Homo sapiens F-box and leucine-rich repeat protein 19 (FBXL19), mRNA [NM_001099784]	**1.476**
RNPS1	Homo sapiens RNA binding protein S1, serine-rich domain (RNPS1), mRNA	**1.473**

	[NM_006711]	
SLC3A2	Homo sapiens solute carrier family 3 (activators of dibasic and neutral amino acid transport), member 2 (SLC3A2), mRNA [NM_001012661]	**1.472**
RPL37A	Homo sapiens ribosomal protein L37a (RPL37A), mRNA [NM_000998]	**1.471**
RAD23A	Homo sapiens RAD23 homolog A (S. cerevisiae) (RAD23A), mRNA [NM_005053]	**1.469**
UNC84B	Homo sapiens unc-84 homolog B (C. elegans) (UNC84B), mRNA [NM_015374]	**1.469**
MLLT1	Homo sapiens myeloid/lymphoid or mixed-lineage leukemia (trithorax homolog, Drosophila); translocated to, 1 (MLLT1), mRNA [NM_005934]	**1.468**
TAX1BP3	Homo sapiens Tax1 (human T-cell leukemia virus type I) binding protein 3 (TAX1BP3), mRNA [NM_014604]	**1.467**
TRAP1	Homo sapiens TNF receptor-associated protein 1 (TRAP1), mRNA [NM_016292]	**1.467**
HNRNPUL2	Homo sapiens heterogeneous nuclear ribonucleoprotein U-like 2 (HNRNPUL2), mRNA [NM_001079559]	**1.465**
AGTRAP	Homo sapiens angiotensin II receptor-associated protein (AGTRAP), mRNA [NM_001040196]	**1.464**
GEMIN5	Homo sapiens gem (nuclear organelle) associated protein 5 (GEMIN5), mRNA [NM_015465]	**1.464**
SUPT16HP	PREDICTED: Homo sapiens misc_RNA (LOC400011), miscRNA [XR_019334]	**1.464**
ZMAT5	Homo sapiens zinc finger, matrin type 5 (ZMAT5), mRNA [NM_019103]	**1.464**
COPS7A	Homo sapiens COP9 constitutive photomorphogenic homolog subunit 7A (Arabidopsis) (COPS7A), mRNA [NM_016319]	**1.463**
ATP1A4	Homo sapiens cDNA FLJ40757 fis, clone TRACH2001996, highly similar to Sodium/potassium-transporting atpase alpha-1 chain precursor (EC 3.6.1.37). [AK098076]	**1.463**
C9orf69	Homo sapiens chromosome 9 open reading frame 69 (C9orf69), mRNA [NM_152833]	**1.462**
CYP4F11	Homo sapiens cytochrome P450, family 4, subfamily F, polypeptide 11 (CYP4F11), mRNA [NM_021187]	**1.461**
SPANXA1	Homo sapiens sperm protein associated with the nucleus, X-linked, family member A1 (SPANXA1), mRNA [NM_013453]	**1.460**
ADCK5	Homo sapiens aarF domain containing kinase 5 (ADCK5), mRNA [NM_174922]	**1.459**
C16orf5	Homo sapiens chromosome 16 open reading frame 5 (C16orf5), mRNA [NM_013399]	**1.459**
TFDP1	Homo sapiens transcription factor Dp-1 (TFDP1), mRNA [NM_007111]	**1.457**
FTL	Homo sapiens ferritin, light polypeptide (FTL), mRNA [NM_000146]	**1.457**
MOV10	Homo sapiens Mov10, Moloney leukemia virus 10, homolog (mouse) (MOV10), mRNA [NM_020963]	**1.456**
MRPL28	Homo sapiens mitochondrial ribosomal protein L28 (MRPL28), mRNA [NM_006428]	**1.455**
DNPEP	Homo sapiens aspartyl aminopeptidase (DNPEP), mRNA [NM_012100]	**1.453**
PTK7	Homo sapiens PTK7 protein tyrosine kinase 7 (PTK7), mRNA [NM_002821]	**1.452**
FAM73B	Homo sapiens family with sequence similarity 73, member B (FAM73B), mRNA	**1.451**

	[NM_032809]	
NPR1	Homo sapiens natriuretic peptide receptor A/guanylate cyclase A (atrionatriuretic peptide receptor A) (NPR1), mRNA [NM_000906]	**1.451**
IL8	Homo sapiens interleukin 8 (IL8), mRNA [NM_000584]	**1.450**
CYBA	Homo sapiens cytochrome b-245, alpha polypeptide, mRNA (cDNA clone IMAGE:5217162), [BC028224]	**1.449**
TEX264	Homo sapiens testis expressed 264 (TEX264), mRNA [NM_015926]	**1.449**
TMEM102	Homo sapiens transmembrane protein 102 (TMEM102), mRNA [NM_178518]	**1.449**
TSPO	Homo sapiens translocator protein (18kDa) (TSPO), mRNA [NM_000714]	**1.449**
C9orf150	Homo sapiens chromosome 9 open reading frame 150 (C9orf150), mRNA [NM_203403]	**1.445**
PCNXL3	Homo sapiens pecanex-like 3 (Drosophila) (PCNXL3), mRNA [NM_032223]	**1.445**
RPS6KB2	Homo sapiens ribosomal protein S6 kinase, 70kDa, polypeptide 2 (RPS6KB2), mRNA [NM_003952]	**1.445**
GAK	Homo sapiens cyclin G associated kinase (GAK), mRNA [NM_005255]	**1.445**
LPCAT1	Homo sapiens lysophosphatidylcholine acyltransferase 1 (LPCAT1), mRNA [NM_024830]	**1.444**
FOXRED1	Homo sapiens FAD-dependent oxidoreductase domain containing 1 (FOXRED1), mRNA [NM_017547]	**1.444**
SMAD6	Homo sapiens SMAD family member 6 (SMAD6), mRNA [NM_005585]	**1.444**
STIP1	Homo sapiens stress-induced-phosphoprotein 1 (STIP1), mRNA [NM_006819]	**1.444**
CC2D1A	Homo sapiens coiled-coil and C2 domain containing 1A (CC2D1A), mRNA [NM_017721]	**1.443**
PEX5	Homo sapiens peroxisomal biogenesis factor 5 (PEX5), mRNA [NM_000319]	**1.442**
FADS3	Homo sapiens fatty acid desaturase 3 (FADS3), mRNA [NM_021727]	**1.440**
HPCA	Homo sapiens hippocalcin (HPCA), mRNA [NM_002143]	**1.440**
KIAA0406	Homo sapiens KIAA0406 (KIAA0406), mRNA [NM_014657]	**1.439**
DUS3L	Homo sapiens dihydrouridine synthase 3-like (S. cerevisiae) (DUS3L), mRNA [NM_020175]	**1.438**
ATP1A3	Homo sapiens ATPase, Na+/K+ transporting, alpha 3 polypeptide (ATP1A3), mRNA [NM_152296]	**1.437**
LOC731275	PREDICTED: Homo sapiens hypothetical LOC731275 (LOC731275), mRNA [XM_001726998]	**1.434**
SLC25A1	Homo sapiens solute carrier family 25 (mitochondrial carrier; citrate transporter), member 1 (SLC25A1), mRNA [NM_005984]	**1.434**
SMPD1	Homo sapiens sphingomyelin phosphodiesterase 1, acid lysosomal (SMPD1), mRNA [NM_000543]	**1.433**
LOC442211	PREDICTED: Homo sapiens misc_RNA (LOC442211), miscRNA [XR_019545]	**1.432**
SSNA1	Homo sapiens Sjogren syndrome nuclear autoantigen 1 (SSNA1), mRNA [NM_003731]	**1.431**
UBE2G2	Homo sapiens ubiquitin-conjugating enzyme E2G 2 (UBC7 homolog, yeast) (UBE2G2), mRNA [NM_182688]	**1.429**
NCRNA00086	Homo sapiens non-protein coding RNA 86 (NCRNA00086), non-coding RNA	**1.426**

	[NR_024359]	
PRKD2	Homo sapiens protein kinase D2 (PRKD2), mRNA [NM_016457]	**1.426**
TUBGCP2	Homo sapiens tubulin, gamma complex associated protein 2 (TUBGCP2), mRNA [NM_006659]	**1.426**
EIF4H	Homo sapiens eukaryotic translation initiation factor 4H (EIF4H), mRNA [NM_022170]	**1.425**
HLA-B	full-length cDNA clone CS0DG002YJ10 of B cells (Ramos cell line) of Homo sapiens (human). [CR608347]	**1.424**
ORAI3	Homo sapiens ORAI calcium release-activated calcium modulator 3 (ORAI3), mRNA [NM_152288]	**1.424**
RNPEP	Homo sapiens arginyl aminopeptidase (aminopeptidase B) (RNPEP), mRNA [NM_020216]	**1.424**
ZDHHC12	Homo sapiens zinc finger, DHHC-type containing 12 (ZDHHC12), mRNA [NM_032799]	**1.424**
VRK3	Homo sapiens vaccinia related kinase 3 (VRK3), mRNA [NM_016440]	**1.423**
NAPA	Homo sapiens N-ethylmaleimide-sensitive factor attachment protein, alpha (NAPA), mRNA [NM_003827]	**1.422**
RALY	Homo sapiens RNA binding protein, autoantigenic (hnRNP-associated with lethal yellow homolog (mouse)) (RALY), mRNA [NM_016732]	**1.422**
TYSND1	Homo sapiens trypsin domain containing 1 (TYSND1), mRNA [NM_173555]	**1.422**
RBM38	Homo sapiens RNA binding motif protein 38 (RBM38), mRNA [NM_017495]	**1.420**
DHCR24	Homo sapiens 24-dehydrocholesterol reductase (DHCR24), mRNA [NM_014762]	**1.420**
TNFRSF1A	Homo sapiens tumor necrosis factor receptor superfamily, member 1A (TNFRSF1A), mRNA [NM_001065]	**1.419**
TUT1	Homo sapiens terminal uridylyl transferase 1, U6 snRNA-specific (TUT1), mRNA [NM_022830]	**1.418**
ACTN4	Homo sapiens actinin, alpha 4 (ACTN4), mRNA [NM_004924]	**1.418**
PLCD3	Homo sapiens phospholipase C, delta 3 (PLCD3), mRNA [NM_133373]	**1.416**
FUS	Homo sapiens fusion (involved in t(12;16) in malignant liposarcoma) (FUS), mRNA [NM_004960]	**1.415**
LTBP3	Homo sapiens latent transforming growth factor beta binding protein 3 (LTBP3), mRNA [NM_021070]	**1.415**
UBTD1	Homo sapiens ubiquitin domain containing 1 (UBTD1), mRNA [NM_024954]	**1.415**
ANKZF1	Homo sapiens ankyrin repeat and zinc finger domain containing 1 (ANKZF1), mRNA [NM_018089]	**1.413**
GBA	Homo sapiens glucosidase, beta; acid (includes glucosylceramidase) (GBA), mRNA [NM_001005749]	**1.412**
NARFL	Homo sapiens nuclear prelamin A recognition factor-like (NARFL), mRNA [NM_022493]	**1.412**
WDR46	Homo sapiens WD repeat domain 46 (WDR46), mRNA [NM_005452]	**1.412**
NUP210	Homo sapiens nucleoporin 210kDa (NUP210), mRNA [NM_024923]	**1.412**
LMAN2	Homo sapiens lectin, mannose-binding 2 (LMAN2), mRNA [NM_006816]	**1.411**
SPANXB2	Homo sapiens SPANX family, member B2 (SPANXB2), mRNA [NM_145664]	**1.410**

TIMM17B	Homo sapiens translocase of inner mitochondrial membrane 17 homolog B (yeast) (TIMM17B), mRNA [NM_005834]	**1.409**
MCAM	Homo sapiens melanoma cell adhesion molecule (MCAM), mRNA [NM_006500]	**1.406**
NOMO1	Homo sapiens NODAL modulator 1 (NOMO1), mRNA [NM_014287]	**1.405**
NOXO1	Homo sapiens NADPH oxidase organizer 1 (NOXO1), mRNA [NM_144603]	**1.405**
PLD3	Homo sapiens phospholipase D family, member 3 (PLD3),mRNA [NM_012268]	**1.405**
TGFB1I1	Homo sapiens transforming growth factor beta 1 induced transcript 1 (TGFB1I1), mRNA [NM_001042454]	**1.404**
HSD17B7P2	Homo sapiens 17-beta-hydroxysteroid dehydrogenase type VII isoform mRNA, complete cds. [AF165514]	**1.403**
ZNHIT2	Homo sapiens zinc finger, HIT type 2 (ZNHIT2), mRNA [NM_014205]	**1.402**
ZNF114	Homo sapiens zinc finger protein 114 (ZNF114), mRNA [NM_153608]	**1.401**
DBNL	Homo sapiens drebrin-like (DBNL), mRNA [NM_014063]	**1.397**
EIF5A	Homo sapiens eukaryotic translation initiation factor 5A (EIF5A), mRNA [NM_001970]	**1.396**
IL1F7	Homo sapiens interleukin 1 family, member 7 (zeta) (IL1F7), mRNA [NM_014439]	**1.396**
OSBP2	Homo sapiens oxysterol binding protein 2 (OSBP2), mRNA [NM_030758]	**1.395**
LOC100190986	Homo sapiens hypothetical LOC100190986 (LOC100190986), non-coding RNA [NR_024456]	**1.395**
RAB21	Homo sapiens RAB21, member RAS oncogene family (RAB21), mRNA [NM_014999]	**1.395**
IGFBP6	Homo sapiens insulin-like growth factor binding protein 6 (IGFBP6), mRNA [NM_002178]	**1.394**
CPNE1	Homo sapiens copine I (CPNE1), transcript variant 7, mRNA [NM_152930]	**1.392**
KRT7	Homo sapiens keratin 7 (KRT7), mRNA [NM_005556]	**1.392**
PTOV1	Homo sapiens prostate tumor overexpressed 1 (PTOV1), mRNA [NM_017432]	**1.391**
SEMA4D	Homo sapiens sema domain, immunoglobulin domain, transmembrane domain and short cytoplasmic domain, (semaphorin) 4D (SEMA4D), mRNA [NM_001142287]	**1.390**
IDH2	Homo sapiens isocitrate dehydrogenase 2 (NADP+), mitochondrial (IDH2), mRNA [NM_002168]	**1.388**
CAP1	Homo sapiens CAP, adenylate cyclase-associated protein 1 (yeast) (CAP1), mRNA [NM_006367]	**1.387**
KHK	Homo sapiens ketohexokinase (fructokinase) (KHK), mRNA [NM_000221]	**1.387**
SLC6A10P	Homo sapiens solute carrier family 6, member 10 (pseudogene) (SLC6A10P) on chromosome 16 [NR_003083]	**1.387**
AARS	Homo sapiens alanyl-tRNA synthetase (AARS), mRNA [NM_001605]	**1.386**
NOTCH1	Homo sapiens Notch homolog 1, translocation-associated (Drosophila) (NOTCH1), mRNA [NM_017617]	**1.385**
PIGS	Homo sapiens phosphatidylinositol glycan anchor biosynthesis, class S (PIGS), mRNA [NM_033198]	**1.385**
WDR66	Homo sapiens WD repeat domain 66 (WDR66), mRNA [NM_144668]	**1.384**

ZMAT3	Homo sapiens zinc finger, matrin type 3 (ZMAT3), mRNA [NM_022470]	**1.384**
SH2B1	Homo sapiens SH2B adaptor protein 1 (SH2B1), mRNA [NM_015503]	**1.383**
ALDOA	Homo sapiens aldolase A, fructose-bisphosphate (ALDOA),mRNA [NM_184041]	**1.382**
URG4	Homo sapiens up-regulated gene 4 (URG4), mRNA [NM_001077664]	**1.382**
CTBP1	Homo sapiens C-terminal binding protein 1 (CTBP1), mRNA [NM_001012614]	**1.382**
RHBDD3	Homo sapiens rhomboid domain containing 3 (RHBDD3),mRNA [NM_012265]	**1.381**
UBE2M	Homo sapiens ubiquitin-conjugating enzyme E2M (UBC12 homolog, yeast) (UBE2M), mRNA [NM_003969]	**1.381**
TUBA1C	Homo sapiens tubulin, alpha 1c (TUBA1C), mRNA [NM_032704]	**1.379**
GOLGA2L1	Homo sapiens golgi autoantigen, golgin subfamily a, 2-like 1 (GOLGA2L1), mRNA [NM_017600]	**1.378**
GCNT2	Homo sapiens glucosaminyl (N-acetyl) transferase 2, I-branching enzyme (I blood group) (GCNT2), mRNA [NM_001491]	**1.377**
MAFF	Homo sapiens v-maf musculoaponeurotic fibrosarcoma oncogene homolog F (avian) (MAFF), mRNA [NM_012323]	**1.377**
MTHFR	Homo sapiens 5,10-methylenetetrahydrofolate reductase (NADPH) (MTHFR), mRNA [NM_005957]	**1.377**
PRKAR2A	Homo sapiens protein kinase,mRNA (cDNA clone MGC:3606 IMAGE:3629579), [BC002763]	**1.375**
ABCC1	Homo sapiens ATP-binding cassette, sub-family C (CFTR/MRP), member 1 (ABCC1), mRNA [NM_019862]	**1.374**
LLGL2	Homo sapiens lethal giant larvae homolog 2 (Drosophila) (LLGL2),mRNA [NM_001015002]	**1.373**
ATAD3A	Homo sapiens ATPase family, AAA domain containing 3A (ATAD3A), mRNA [NM_018188]	**1.372**
C11orf59	Homo sapiens chromosome 11 open reading frame 59 (C11orf59), mRNA [NM_017907]	**1.370**
ATP6V0E2	Homo sapiens ATPase, H+ transporting V0 subunit e2 (ATP6V0E2), mRNA [NM_145230]	**1.369**
GTF2F1	Homo sapiens general transcription factor IIF, polypeptide 1, (GTF2F1), mRNA [NM_002096]	**1.369**
TMEM161A	Homo sapiens transmembrane protein 161A (TMEM161A),mRNA [NM_017814]	**1.369**
ACTN3	Homo sapiens actinin, alpha 3 (ACTN3), mRNA [NM_001104]	**1.368**
COIL	Homo sapiens coilin (COIL), mRNA [NM_004645]	**1.367**
MED24	Homo sapiens mediator complex subunit 24 (MED24), mRNA [NM_014815]	**1.367**
PISD	Homo sapiens phosphatidylserine decarboxylase (PISD), mRNA [NM_014338]	**1.366**
ZDHHC18	Homo sapiens zinc finger, DHHC-type containing 18 (ZDHHC18), mRNA [NM_032283]	**1.366**
CG012	Novel human gene mapping to chomosome 13. [AL049782]	**1.365**
EPGN	Homo sapiens epithelial mitogen homolog (mouse) (EPGN),mRNA [NM_001013442]	**1.365**
TXLNA	Homo sapiens taxilin alpha (TXLNA), mRNA [NM_175852]	**1.364**
LOC100130288	Homo sapiens cDNA clone IMAGE:5295205, [BC043212]	**1.364**

GRAMD1A	Homo sapiens GRAM domain containing 1A (GRAMD1A), mRNA [NM_020895]	**1.362**
ABHD3	Homo sapiens abhydrolase domain containing 3 (ABHD3), mRNA [NM_138340]	**1.360**
NCAPH2	Homo sapiens non-SMC condensin II complex, subunit H2 (NCAPH2), mRNA [NM_152299]	**1.360**
SV2A	Homo sapiens synaptic vesicle glycoprotein 2A (SV2A), mRNA [NM_014849]	**1.360**
STC2	Homo sapiens stanniocalcin 2 (STC2), mRNA [NM_003714]	**1.360**
PSMC3	Homo sapiens proteasome (prosome, macropain) 26S subunit, ATPase, 3 (PSMC3), mRNA [NM_002804]	**1.359**
TSC22D3	Homo sapiens TSC22 domain family, member 3 (TSC22D3),mRNA [NM_004089]	**1.358**
ARHGEF2	Homo sapiens rho/rac guanine nucleotide exchange factor (GEF) 2 (ARHGEF2), mRNA [NM_004723]	**1.357**
LOC648740	Homo sapiens ACTB pseudogene (LOC648740), non-coding RNA [NR_024438]	**1.355**
CCK	Homo sapiens cholecystokinin (CCK), mRNA [NM_000729]	**1.354**
USP22	Homo sapiens ubiquitin specific peptidase 22 (USP22), mRNA [NM_015276]	**1.354**
MAPKAP1	Homo sapiens mitogen-activated protein kinase associated protein 1 (MAPKAP1), mRNA [NM_001006617]	**1.354**
DKFZp761E198	Homo sapiens DKFZp761E198 protein (DKFZp761E198), mRNA [NM_138368]	**1.353**
M6PRBP1	Homo sapiens mannose-6-phosphate receptor binding protein 1 (M6PRBP1), mRNA [NM_005817]	**1.353**
ZFPL1	Homo sapiens zinc finger protein-like 1 (ZFPL1), mRNA [NM_006782]	**1.353**
LAMP1	Homo sapiens lysosomal-associated membrane protein 1 (LAMP1), mRNA [NM_005561]	**1.352**
ZNF581	Homo sapiens zinc finger protein 581 (ZNF581), mRNA [NM_016535]	**1.351**
SLC16A6	Homo sapiens solute carrier family 16, member 6 (monocarboxylic acid transporter 7) (SLC16A6), mRNA [NM_004694]	**1.351**
LOC728411	Homo sapiens cDNA FLJ58963 complete cds, highly similar to Beta-glucuronidase-like protein SMA3. [AK302866]	**1.349**
ENO1	Homo sapiens enolase 1, (alpha) (ENO1), mRNA [NM_001428]	**1.349**
SETD1A	Homo sapiens SET domain containing 1A (SETD1A), mRNA [NM_014712]	**1.349**
TRAFD1	Homo sapiens TRAF-type zinc finger domain containing 1 (TRAFD1), mRNA [NM_006700]	**1.348**
LOC100132673	PREDICTED: Homo sapiens misc_RNA (LOC100132673),miscRNA [XR_039018]	**1.347**
UGDH	Homo sapiens UDP-glucose dehydrogenase (UGDH), mRNA [NM_003359]	**1.347**
LOC100130193	Homo sapiens cDNA FLJ38783 fis, clone LIVER2001191. [AK096102]	**1.346**
USF2	Homo sapiens clone TCCCIA00046 mRNA sequence. [AY007087]	**1.346**
LOC284344	Homo sapiens cDNA FLJ40353 fis, clone TESTI2033520, weakly similar to BILIARY GLYCOPROTEIN 1 PRECURSOR. [AK097672]	**1.345**
MED15	Homo sapiens mediator complex subunit 15 (MED15),mRNA [NM_001003891]	**1.345**
ZFAND2A	Homo sapiens zinc finger, AN1-type domain 2A (ZFAND2A), mRNA [NM_182491]	**1.345**

PVR	Homo sapiens poliovirus receptor (PVR), mRNA [NM_006505]	**1.345**
TRIML2	Homo sapiens tripartite motif family-like 2 (TRIML2), mRNA [NM_173553]	**1.344**
CEBPB	Homo sapiens CCAAT/enhancer binding protein (C/EBP), beta (CEBPB), mRNA [NM_005194]	**1.343**
TMED1	Homo sapiens transmembrane emp24 protein transport domain containing 1 (TMED1), mRNA [NM_006858]	**1.341**
BAD	Homo sapiens BCL2-associated agonist of cell death (BAD), mRNA [NM_004322]	**1.340**
PSG1	Homo sapiens pregnancy specific beta-1-glycoprotein 1 (PSG1), mRNA [NM_006905]	**1.340**
ELF3	Homo sapiens E74-like factor 3 (ets domain transcription factor, epithelial-specific) (ELF3), mRNA [NM_004433]	**1.339**
MAD1L1	Homo sapiens MAD1 mitotic arrest deficient-like 1 (yeast) (MAD1L1), mRNA [NM_003550]	**1.338**
RPL18A	Homo sapiens ribosomal protein L18a (RPL18A), mRNA [NM_000980]	**1.338**
SIRT7	Homo sapiens sirtuin (silent mating type information regulation 2 homolog) 7 (S. cerevisiae) (SIRT7), mRNA [NM_016538]	**1.338**
TPRG1L	Homo sapiens tumor protein p63 regulated 1-like (TPRG1L), mRNA [NM_182752]	**1.338**
LASP1	Homo sapiens LIM and SH3 protein 1 (LASP1), mRNA [NM_006148]	**1.336**
TBCB	Homo sapiens tubulin folding cofactor B (TBCB), mRNA [NM_001281]	**1.336**
ZNF263	Homo sapiens zinc finger protein 263 (ZNF263), mRNA [NM_005741]	**1.336**
VGLL4	Homo sapiens vestigial like 4 (Drosophila) (VGLL4), mRNA [NM_014667]	**1.336**
OXA1L	Homo sapiens oxidase (cytochrome c) assembly 1-like (OXA1L), mRNA [NM_005015]	**1.335**
C6orf223	Homo sapiens chromosome 6 open reading frame 223 (C6orf223), mRNA [NM_153246]	**1.333**
LOC344887	Homo sapiens mRNA; cDNA DKFZp686B14224 (from clone DKFZp686B14224). [BX640843]	**1.331**
LOC100134400	PREDICTED: Homo sapiens hypothetical protein LOC100134400 (LOC100134400), mRNA [XM_001718660]	**1.330**
SNX9	Homo sapiens sorting nexin 9 (SNX9), mRNA [NM_016224]	**1.330**
CTF8	Homo sapiens chromosome transmission fidelity factor 8 homolog (S. cerevisiae) (CTF8), mRNA [NM_001039690]	**1.328**
MYL9	Homo sapiens myosin, light chain 9, regulatory (MYL9), mRNA [NM_181526]	**1.328**
SFRS9	Homo sapiens splicing factor, arginine/serine-rich 9 (SFRS9), mRNA [NM_003769]	**1.328**
TMBIM1	Homo sapiens transmembrane BAX inhibitor motif containing 1 (TMBIM1), mRNA [NM_022152]	**1.328**
ARRDC1	Homo sapiens arrestin domain containing 1 (ARRDC1), mRNA [NM_152285]	**1.326**
ZNF445	Homo sapiens zinc finger protein 445 (ZNF445), mRNA [NM_181489]	**1.326**
ATRIP	Homo sapiens ATR interacting protein (ATRIP), mRNA [NM_032166]	**1.325**
MDN1	Homo sapiens MDN1, midasin homolog (yeast) (MDN1), mRNA [NM_014611]	**1.324**

CDC34	Homo sapiens cell division cycle 34 homolog (S. cerevisiae) (CDC34), mRNA [NM_004359]	**1.322**
NPC1	Homo sapiens Niemann-Pick disease, type C1 (NPC1), mRNA [NM_000271]	**1.321**
SLC25A11	Homo sapiens solute carrier family 25 (mitochondrial carrier; oxoglutarate carrier), member 11 (SLC25A11), mRNA [NM_003562]	**1.321**
CPN2	Homo sapiens carboxypeptidase N, polypeptide 2 (CPN2), mRNA [NM_001080513]	**1.320**
KCTD21	Homo sapiens potassium channel tetramerisation domain containing 21 (KCTD21), mRNA [NM_001029859]	**1.320**
MAGEA4	Homo sapiens melanoma antigen family A, 4 (MAGEA4), mRNA [NM_002362]	**1.320**
PPP2R1A	Homo sapiens protein phosphatase 2 (formerly 2A), regulatory subunit A, alpha isoform (PPP2R1A), mRNA [NM_014225]	**1.318**
VCX2	Homo sapiens variable charge, X-linked 2 (VCX2), mRNA [NM_016378]	**1.318**
PIK3IP1	Homo sapiens phosphoinositide-3-kinase interacting protein 1 (PIK3IP1), mRNA [NM_052880]	**1.316**
SLC26A2	Homo sapiens solute carrier family 26 (sulfate transporter), member 2 (SLC26A2), mRNA [NM_000112]	**1.316**
PLDN	Homo sapiens pallidin homolog (mouse) (PLDN), mRNA [NM_012388]	**1.316**
NUDT16L1	Homo sapiens nudix (nucleoside diphosphate linked moiety X)-type motif 16-like 1 (NUDT16L1), mRNA [NM_032349]	**1.316**
TUBG2	Homo sapiens tubulin, gamma 2 (TUBG2), mRNA [NM_016437]	**1.315**
RAB7L1	Homo sapiens RAB7, member RAS oncogene family-like 1 (RAB7L1), mRNA [NM_003929]	**1.315**
FAM125A	Homo sapiens family with sequence similarity 125, member A (FAM125A), mRNA [NM_138401]	**1.314**
HK1	Homo sapiens hexokinase 1 (HK1), mRNA [NM_033497]	**1.314**
WDR13	Human MG21 mRNA, partial cds. [L08237]	**1.313**
KIAA1967	Homo sapiens KIAA1967 (KIAA1967), mRNA [NM_021174]	**1.313**
GPATCH3	Homo sapiens G patch domain containing 3 (GPATCH3), mRNA [NM_022078]	**1.312**
HNRNPL	Homo sapiens heterogeneous nuclear ribonucleoprotein L (HNRNPL), mRNA [NM_001533]	**1.311**
PFN1	Homo sapiens profilin 1 (PFN1), mRNA [NM_005022]	**1.311**
GTPBP5	Homo sapiens GTP binding protein 5 (putative) (GTPBP5), mRNA [NM_015666]	**1.311**
C17orf53	Homo sapiens chromosome 17 open reading frame 53 (C17orf53), mRNA [NM_024032]	**1.311**
FLJ39582	Full-length cDNA clone CS0DI082YF06 of Placenta Cot 25-normalized of Homo sapiens (human). [CR600536]	**1.310**
LOC644456	Homo sapiens misc_RNA (LOC644456), miscRNA [XR_039438]	**1.310**
C6orf1	Homo sapiens chromosome 6 open reading frame 1 (C6orf1), mRNA [NM_178508]	**1.310**
MRPL12	Homo sapiens mitochondrial ribosomal protein L12 (MRPL12),mRNA [NM_002949]	**1.309**
DUS2L	Homo sapiens dihydrouridine synthase 2-like, SMM1 homolog (S. cerevisiae)	**1.308**

	(DUS2L), mRNA [NM_017803]	
FRG2	Homo sapiens FSHD region gene 2 (FRG2), mRNA [NM_001005217]	**1.308**
RABEP2	Homo sapiens rabaptin, RAB GTPase binding effector protein 2 (RABEP2), mRNA [NM_024816]	**1.308**
CNN2	Homo sapiens calponin 2 (CNN2), mRNA [NM_004368]	**1.308**
FHL3	Homo sapiens four and a half LIM domains 3 (FHL3), mRNA [NM_004468]	**1.306**
ISY1	Pre-mRNA-splicing factor ISY1 homolog [Source:UniProtKB/ Swiss-Prot; Acc:Q9ULR0] [ENST00000393295]	**1.306**
SNX8	Homo sapiens sorting nexin 8 (SNX8), mRNA [NM_013321]	**1.305**
DGCR6L	Homo sapiens DiGeorge syndrome critical region gene 6-like (DGCR6L), mRNA [NM_033257]	**1.304**
GPS1	Homo sapiens G protein pathway suppressor 1 (GPS1), mRNA [NM_212492]	**1.304**
MMAB	Homo sapiens methylmalonic aciduria (cobalamin deficiency) cblB type (MMAB), mRNA [NM_052845]	**1.303**
PBXIP1	Homo sapiens pre-B-cell leukemia homeobox interacting protein 1 (PBXIP1), mRNA [NM_020524]	**1.303**
PTBP1	Homo sapiens polypyrimidine tract binding protein 1 (PTBP1),mRNA [NM_002819]	**1.303**
UNQ1870	Homo sapiens clone DNA62312 GALI1870 (UNQ1870) mRNA, [AY358688]	**1.303**
ATF3	Homo sapiens activating transcription factor 3 (ATF3),mRNA [NM_001040619]	**1.302**
C9orf114	Homo sapiens chromosome 9 open reading frame 114 (C9orf114), mRNA [NM_016390]	**1.301**
IGFBP3	Homo sapiens insulin-like growth factor binding protein 3 (IGFBP3), mRNA [NM_001013398]	**1.299**
EDC4	Homo sapiens enhancer of mRNA decapping 4 (EDC4), mRNA [NM_014329]	**1.298**
KIAA1486	Homo sapiens KIAA1486 protein (KIAA1486), mRNA [NM_020864]	**1.297**
SLC25A39	Homo sapiens solute carrier family 25, member 39 (SLC25A39),mRNA [NM_016016]	**1.295**
ENSA	Homo sapiens endosulfine alpha (ENSA), mRNA [NM_004436]	**1.293**
DHRS2	Homo sapiens dehydrogenase/reductase (SDR family) member 2 (DHRS2), mRNA [NM_182908]	**1.293**
C9orf16	Homo sapiens chromosome 9 open reading frame 16 (C9orf16), mRNA [NM_024112]	**1.292**
PER1	Homo sapiens period homolog 1 (Drosophila), mRNA (cDNA clone IMAGE:5215552). [BC028207]	**1.292**
CASP1	Homo sapiens caspase 1, apoptosis-related cysteine peptidase (interleukin 1, beta, convertase) (CASP1), mRNA [NM_033292]	**1.291**
GCLC	Homo sapiens glutamate-cysteine ligase, catalytic subunit (GCLC), mRNA [NM_001498]	**1.291**
GADD45A	Homo sapiens growth arrest and DNA-damage-inducible, alpha (GADD45A), mRNA [NM_001924]	**1.290**
S100P	Homo sapiens S100 calcium binding protein P (S100P), mRNA [NM_005980]	**1.290**
ATF4C	PREDICTED: Homo sapiens similar to activating transcription factor 4,	**1.289**

	(LOC643159), mRNA [XM_928637]	
TSSC4	Homo sapiens tumor suppressing subtransferable candidate 4 (TSSC4), mRNA [NM_005706]	**1.289**
HYAL1	Homo sapiens hyaluronoglucosaminidase 1 (HYAL1), mRNA [NM_007312]	**1.288**
YES1	Homo sapiens v-yes-1 Yamaguchi sarcoma viral oncogene homolog 1 (YES1), mRNA [NM_005433]	**1.288**
CTSL1	Homo sapiens cathepsin L1 (CTSL1), mRNA [NM_001912]	**1.287**
RND2	Homo sapiens Rho family GTPase 2 (RND2), mRNA [NM_005440]	**1.287**
TTYH3	Homo sapiens tweety homolog 3 (Drosophila) (TTYH3), mRNA [NM_025250]	**1.287**
ZNF687	Homo sapiens zinc finger protein 687 (ZNF687), mRNA [NM_020832]	**1.286**
POF1B	Homo sapiens premature ovarian failure, 1B (POF1B), mRNA [NM_024921]	**1.285**
NCRNA00087	Homo sapiens non-protein coding RNA 87 (NCRNA00087), non-coding RNA [NR_024493]	**1.284**
CTSA	Homo sapiens cathepsin A (CTSA), mRNA [NM_000308]	**1.284**
KRT8	Homo sapiens keratin 8 (KRT8), mRNA [NM_002273]	**1.284**
STXBP1	Homo sapiens syntaxin binding protein 1 (STXBP1), mRNA [NM_001032221]	**1.280**
UBL4A	Homo sapiens ubiquitin-like 4A (UBL4A), mRNA [NM_014235]	**1.280**
ELOF1	Homo sapiens elongation factor 1 homolog (S. cerevisiae) (ELOF1), mRNA [NM_032377]	**1.279**
MED27	Homo sapiens mediator complex subunit 27 (MED27), mRNA [NM_004269]	**1.279**
B3GAT3	Homo sapiens beta-1,3-glucuronyltransferase 3 (glucuronosyltransferase I) (B3GAT3), mRNA [NM_012200]	**1.278**
GPR108	Homo sapiens G protein-coupled receptor 108 (GPR108), mRNA [NM_020171]	**1.278**
NOB1	Homo sapiens NIN1/RPN12 binding protein 1 homolog (S. cerevisiae) (NOB1), mRNA [NM_014062]	**1.278**
GPATCH1	Homo sapiens G patch domain containing 1 (GPATCH1), mRNA [NM_018025]	**1.277**
SHMT2	Homo sapiens serine hydroxymethyltransferase 2 (mitochondrial) (SHMT2), mRNA [NM_005412]	**1.277**
UBE2J2	Homo sapiens ubiquitin-conjugating enzyme E2, J2 (UBC6 homolog, yeast) (UBE2J2), mRNA [NM_194458]	**1.275**
HNRNPA0	Homo sapiens heterogeneous nuclear ribonucleoprotein A0 (HNRNPA0), mRNA [NM_006805]	**1.275**
KAT5	Homo sapiens K(lysine) acetyltransferase 5 (KAT5), mRNA [NM_006388]	**1.274**
LOC161635	Homo sapiens, clone IMAGE:5166482, mRNA, partial cds. [BC028192]	**1.274**
LRSAM1	Homo sapiens leucine rich repeat and sterile alpha motif containing 1 (LRSAM1), mRNA [NM_138361]	**1.273**
TSPAN4	Homo sapiens tetraspanin 4 (TSPAN4), mRNA [NM_001025237]	**1.273**
RAP1GAP	Homo sapiens RAP1 GTPase activating protein (RAP1GAP), mRNA [NM_002885]	**1.272**
FAM168B	Homo sapiens family with sequence similarity 168, member B (FAM168B), mRNA [NM_001009993]	**1.271**
RPL8	Homo sapiens ribosomal protein L8 (RPL8), mRNA [NM_000973]	**1.271**
ARHGAP29	Homo sapiens Rho GTPase activating protein 29, mRNA (cDNA clone	**1.269**

	IMAGE:4795792), [BC022483]	
SLC17A5	Homo sapiens solute carrier family 17 (anion/sugar transporter), member 5 (SLC17A5), mRNA [NM_012434]	**1.269**
WDR79	Homo sapiens WD repeat domain 79 (WDR79), mRNA [NM_018081]	**1.269**
TSC22D4	Homo sapiens TSC22 domain family, member 4 (TSC22D4), mRNA [NM_030935]	**1.269**
CCDC21	Homo sapiens coiled-coil domain containing 21 (CCDC21), mRNA [NM_022778]	**1.268**
MGC16142	Homo sapiens hypothetical protein MGC16142, mRNA (cDNA clone IMAGE:3630861), [BC007365]	**1.268**
SH3KBP1	Homo sapiens SH3-domain kinase binding protein 1 (SH3KBP1),mRNA [NM_001024666]	**1.268**
MIB1	Homo sapiens mindbomb homolog 1 (Drosophila) (MIB1), mRNA [NM_020774]	**1.267**
TOP1	Homo sapiens topoisomerase (DNA) I (TOP1), mRNA [NM_003286]	**1.266**
DOK7	Homo sapiens docking protein 7 (DOK7), mRNA [NM_173660]	**1.265**
NPB	Homo sapiens cDNA clone IMAGE:5019903, [BC073815]	**1.265**
OGDH	Homo sapiens oxoglutarate (alpha-ketoglutarate) dehydrogenase (lipoamide) (OGDH), mRNA [NM_001003941]	**1.264**
RP5-1077B9.4	Homo sapiens invasion inhibitory protein 45 (IIP45), mRNA [NM_021933]	**1.263**
SEC23B	Homo sapiens Sec23 homolog B (S. cerevisiae) (SEC23B),mRNA [NM_032985]	**1.263**
COQ10B	Homo sapiens coenzyme Q10 homolog B (S. cerevisiae) (COQ10B), mRNA [NM_025147]	**1.262**
GMPPA	Homo sapiens GDP-mannose pyrophosphorylase A (GMPPA),mRNA [NM_013335]	**1.262**
MSTO1	Homo sapiens misato homolog 1 (Drosophila) (MSTO1), mRNA [NM_018116]	**1.261**
NAV3	Homo sapiens neuron navigator 3 (NAV3), mRNA [NM_014903]	**1.260**
DAB2IP	Homo sapiens DAB2 interacting protein (DAB2IP), mRNA [NM_138709]	**1.259**
YIF1B	Homo sapiens Yip1 interacting factor homolog B (S. cerevisiae) (YIF1B), mRNA [NM_033557]	**1.258**
TPD52L2	Homo sapiens tumor protein D52-like 2 (TPD52L2), transcript variant 1, mRNA [NM_199360]	**1.257**
RRP1	Homo sapiens ribosomal RNA processing 1 homolog (S. cerevisiae) (RRP1), mRNA [NM_003683]	**1.257**
FAM111B	Homo sapiens family with sequence similarity 111, member B (FAM111B), mRNA [NM_198947]	**1.256**
MYO16	Homo sapiens myosin XVI (MYO16), mRNA [NM_015011]	**1.256**
TXNRD2	Homo sapiens mitochondrial thioredoxin reductase (TRXR2A) mRNA, [AF201385]	**1.256**
ATP2B1	Homo sapiens ATPase, Ca++ transporting, plasma membrane 1 (ATP2B1), mRNA [NM_001682]	**1.255**
SUOX	Homo sapiens sulfite oxidase (SUOX), mRNA [NM_000456]	**1.255**
MED16	Homo sapiens mediator complex subunit 16 (MED16), mRNA [NM_005481]	**1.253**
SLC31A1	Homo sapiens solute carrier family 31 (copper transporters), member 1	**1.253**

	(SLC31A1), mRNA [NM_001859]	
DAPK3	Homo sapiens death-associated protein kinase 3 (DAPK3), mRNA [NM_001348]	**1.252**
LOC729090	PREDICTED: Homo sapiens similar to Eukaryotic translation elongation factor 1 alpha 1 (LOC729090), mRNA [XR_015449]	**1.252**
NELF	Homo sapiens nasal embryonic LHRH factor (NELF), mRNA [NM_015537]	**1.252**
SMARCA2	Homo sapiens SWI/SNF related, subfamily a, member 2 (SMARCA2), mRNA [NM_139045]	**1.252**
SRP68	Homo sapiens signal recognition particle 68kDa (SRP68), mRNA [NM_014230]	**1.252**
ALDH1B1	Homo sapiens aldehyde dehydrogenase 1 family, member B1 (ALDH1B1), mRNA [NM_000692]	**1.251**
NCL	Homo sapiens nucleolin (NCL), mRNA [NM_005381]	**1.251**
LOC644063	PREDICTED: Homo sapiens misc_RNA (LOC644063), miscRNA [XR_018217]	**1.249**
TMEM160	Homo sapiens transmembrane protein 160 (TMEM160), mRNA [NM_017854]	**1.248**
ZEB1	Homo sapiens zinc finger E-box binding homeobox 1 (ZEB1),mRNA [NM_030751]	**1.247**
RNF20	Homo sapiens ring finger protein 20 (RNF20), mRNA [NM_019592]	**1.245**
RNF40	Homo sapiens ring finger protein 40 (RNF40), mRNA [NM_014771]	**1.245**
COBRA1	Homo sapiens cofactor of BRCA1 (COBRA1), mRNA [NM_015456]	**1.244**
IL1A	Homo sapiens interleukin 1, alpha (IL1A), mRNA [NM_000575]	**1.244**
YKT6	Homo sapiens YKT6 v-SNARE homolog (S. cerevisiae) (YKT6), mRNA [NM_006555]	**1.244**
ABCF2	Homo sapiens ATP-binding cassette, sub-family F (GCN20), member 2 (ABCF2), nmRNA [NM_005692]	**1.243**
CCDC115	Homo sapiens coiled-coil domain containing 115 (CCDC115), mRNA [NM_032357]	**1.243**
CPSF3L	Homo sapiens cleavage and polyadenylation specific factor 3-like (CPSF3L), mRNA [NM_017871]	**1.243**
TUBB	Homo sapiens tubulin, beta (TUBB), mRNA [NM_178014]	**1.242**
FARSA	Homo sapiens phenylalanyl-tRNA synthetase, alpha subunit (FARSA), mRNA [NM_004461]	**1.242**
CPOX	Homo sapiens coproporphyrinogen oxidase (CPOX), mRNA [NM_000097]	**1.240**
GTF3C5	Homo sapiens general transcription factor IIIC, polypeptide 5, 63kDa (GTF3C5), mRNA [NM_012087]	**1.240**
LIMS1	Homo sapiens LIM and senescent cell antigen-like domains 1 (LIMS1), mRNA [NM_004987]	**1.238**
MTX1	Homo sapiens metaxin 1 (MTX1), mRNA [NM_198883]	**1.237**
PRO2852	Homo sapiens clone FLC0578 PRO2852 mRNA, [AF130079]	**1.237**
FOXK1	Homo sapiens forkhead box K1 (FOXK1), mRNA [NM_001037165]	**1.237**
SC65	Homo sapiens synaptonemal complex protein SC65 (SC65), mRNA [NM_006455]	**1.236**
IPO7	Homo sapiens importin 7 (IPO7), mRNA [NM_006391]	**1.235**
IVD	Homo sapiens isovaleryl Coenzyme A dehydrogenase (IVD),mRNA [NM_002225]	**1.235**

CHST10	Homo sapiens carbohydrate sulfotransferase 10 (CHST10), mRNA [NM_004854]	**1.234**
SDHA	Homo sapiens succinate dehydrogenase complex, subunit A, flavoprotein (Fp) (SDHA), mRNA [NM_004168]	**1.234**
SLC2A3	Homo sapiens solute carrier family 2 (facilitated glucose transporter), member 3 (SLC2A3), mRNA [NM_006931]	**1.234**
ELOVL1	Homo sapiens elongation of very long chain fatty acids (FEN1/Elo2, SUR4/Elo3, yeast)-like 1 (ELOVL1), mRNA [NM_022821]	**1.231**
EPB41	Homo sapiens erythrocyte membrane protein band 4.1 (elliptocytosis 1, RH-linked) (EPB41), mRNA [NM_004437]	**1.231**
IRS2	Homo sapiens insulin receptor substrate 2 (IRS2), mRNA [NM_003749]	**1.231**
CCNY	Homo sapiens cyclin Y (CCNY), mRNA [NM_145012]	**1.230**
CHKA	Homo sapiens choline kinase alpha (CHKA), mRNA [NM_001277]	**1.230**
CUEDC1	Homo sapiens CUE domain containing 1 (CUEDC1), mRNA [NM_017949]	**1.230**
ZNF280A	Homo sapiens zinc finger protein 280A (ZNF280A), mRNA [NM_080740]	**1.230**
DUSP10	Homo sapiens dual specificity phosphatase 10 (DUSP10), mRNA [NM_007207]	**1.230**
APEX1	Homo sapiens APEX nuclease (multifunctional DNA repair enzyme) 1 (APEX1), mRNA [NM_080649]	**1.229**
ZBTB43	Homo sapiens zinc finger and BTB domain containing 43 (ZBTB43), mRNA [NM_014007]	**1.229**
SH3TC1	Homo sapiens SH3 domain and tetratricopeptide repeats 1 (SH3TC1), mRNA [NM_018986]	**1.228**
RHOD	Homo sapiens ras homolog gene family, member D (RHOD), mRNA [NM_014578]	**1.227**
RP11-631M21.2	Homo sapiens tubulin, beta 8 (TUBB8), mRNA [NM_177987]	**1.227**
LOC645693	PREDICTED: Homo sapiens misc_RNA (LOC645693), miscRNA [XR_017498]	**1.225**
PSG11	Homo sapiens pregnancy specific beta-1-glycoprotein 11 (PSG11), mRNA [NM_002785]	**1.224**
TSC22D1	Homo sapiens TSC22 domain family, member 1 (TSC22D1),mRNA [NM_183422]	**1.224**
ITSN2	Homo sapiens intersectin 2 (ITSN2), mRNA [NM_147152]	**1.223**
RORC	Homo sapiens RAR-related orphan receptor C (RORC), mRNA [NM_005060]	**1.223**
CDC25B	Homo sapiens cell division cycle 25 homolog B (S. pombe) (CDC25B), mRNA [NM_021873]	**1.222**
CTCF	Homo sapiens CCCTC-binding factor (zinc finger protein) (CTCF), mRNA [NM_006565]	**1.222**
WASF2	Homo sapiens WAS protein family, member 2 (WASF2), mRNA [NM_006990]	**1.222**
TARS2	Homo sapiens threonyl-tRNA synthetase 2, mitochondrial (putative) (TARS2), mRNA [NM_025150]	**1.220**
CHMP7	Homo sapiens CHMP family, member 7 (CHMP7), mRNA [NM_152272]	**1.220**
CUEDC2	Homo sapiens CUE domain containing 2 (CUEDC2), mRNA [NM_024040]	**1.220**
EIF2S3	Homo sapiens eukaryotic translation initiation factor 2, subunit 3 gamma, (EIF2S3), mRNA [NM_001415]	**1.219**
ETV4	Homo sapiens ets variant 4 (ETV4), transcript variant 2, mRNA	**1.219**

	[NM_001079675]	
PROSC	Homo sapiens proline synthetase co-transcribed homolog (bacterial) (PROSC), mRNA [NM_007198]	**1.219**
C19orf20	Homo sapiens chromosome 19 open reading frame 20 (C19orf20), mRNA [NM_033513]	**1.218**
FSTL3	Homo sapiens follistatin-like 3 (secreted glycoprotein) (FSTL3), mRNA [NM_005860]	**1.218**
PEX10	Homo sapiens peroxisomal biogenesis factor 10 (PEX10), mRNA [NM_002617]	**1.218**
HMGCL	Homo sapiens 3-hydroxymethyl-3-methylglutaryl-Coenzyme A lyase (HMGCL), mRNA [NM_000191]	**1.217**
RAB7A	Homo sapiens RAB7A, member RAS oncogene family (RAB7A), mRNA [NM_004637]	**1.217**
RRAGC	Homo sapiens Ras-related GTP binding C (RRAGC), mRNA [NM_022157]	**1.217**
ATP6V1B2	Homo sapiens ATPase, H+ transporting, lysosomal 56/58kDa, V1 subunit B2 (ATP6V1B2), mRNA [NM_001693]	**1.216**
RHOB	Homo sapiens ras homolog gene family, member B (RHOB), mRNA [NM_004040]	**1.216**
TAZ	Homo sapiens tafazzin (TAZ), mRNA [NM_000116]	**1.216**
FADD	Homo sapiens Fas (TNFRSF6)-associated via death domain (FADD), mRNA [NM_003824]	**1.215**
COMMD7	Homo sapiens COMM domain containing 7 (COMMD7), mRNA [NM_053041]	**1.214**
CA5BL	Homo sapiens carbonic anhydrase VB-like (CA5BL), non-coding RNA [NR_026551]	**1.213**
MFHAS1	Homo sapiens malignant fibrous histiocytoma amplified sequence 1 (MFHAS1), mRNA [NM_004225]	**1.213**
APLP2	Homo sapiens amyloid beta (A4) precursor-like protein 2 (APLP2),mRNA [NM_001642]	**1.213**
IFI16	Homo sapiens interferon, gamma-inducible protein 16 (IFI16), mRNA [NM_005531]	**1.212**
MFSD10	Homo sapiens major facilitator superfamily domain containing 10 (MFSD10), mRNA [NM_001120]	**1.212**
DAK	Homo sapiens dihydroxyacetone kinase 2 homolog (S. cerevisiae) (DAK), mRNA [NM_015533]	**1.210**
SEMA3F	Homo sapiens sema domain, immunoglobulin domain , (semaphorin) 3F (SEMA3F), mRNA [NM_004186]	**1.210**
PSG6	Homo sapiens pregnancy specific beta-1-glycoprotein 6 (PSG6), mRNA [NM_002782]	**1.209**
C3orf21	Homo sapiens chromosome 3 open reading frame 21 (C3orf21), mRNA [NM_152531]	**1.207**
UNC13D	Homo sapiens mRNA for FLJ00067 protein, partial cds. [AK024474]	**1.206**
SMARCA4	Homo sapiens SWI/SNF related, matrix associated, subfamily a, member 4 (SMARCA4), mRNA [NM_003072]	**1.206**
CD2BP2	Homo sapiens CD2 (cytoplasmic tail) binding protein 2 (CD2BP2), tramRNA	**1.206**

	[NM_006110]	
PRKAG1	Homo sapiens protein kinase, AMP-activated, gamma 1 non-catalytic subunit (PRKAG1), mRNA [NM_212461]	**1.205**
TRIM47	Homo sapiens tripartite motif-containing 47 (TRIM47), mRNA [NM_033452]	**1.205**
FOLR1	Homo sapiens folate receptor 1 (adult) (FOLR1), mRNA [NM_016725]	**1.204**
PRPF31	Homo sapiens PRP31 pre-mRNA processing factor 31 homolog (S. cerevisiae) (PRPF31), mRNA [NM_015629]	**1.204**
ADSS	Homo sapiens adenylosuccinate synthase (ADSS), mRNA [NM_001126]	**1.204**
B4GALNT1	Homo sapiens beta-1,4-N-acetyl-galactosaminyl transferase 1 (B4GALNT1), mRNA [NM_001478]	**1.203**
FAM120B	Homo sapiens family with sequence similarity 120B (FAM120B), mRNA [NM_032448]	**1.202**
GTF3C1	Homo sapiens general transcription factor IIIC, polypeptide 1, alpha (GTF3C1), mRNA [NM_001520]	**1.201**
UGCG	Homo sapiens UDP-glucose ceramide glucosyltransferase (UGCG), mRNA [NM_003358]	**1.201**
SLC25A19	Homo sapiens solute carrier family 25 (mitochondrial thiamine pyrophosphate carrier), member 19 (SLC25A19), mRNA [NM_021734]	**1.200**
WDR62	Homo sapiens WD repeat domain 62 (WDR62), mRNA [NM_173636]	**1.200**
RBM3	Homo sapiens RNA binding motif (RNP1, RRM) protein 3 (RBM3), mRNA [NM_006743]	**1.199**
STXBP2	Homo sapiens syntaxin binding protein 2 (STXBP2), mRNA [NM_006949]	**1.199**
TAS2R45	Homo sapiens taste receptor, type 2, member 45 (TAS2R45), mRNA [NM_176886]	**1.199**
TSPAN17	Homo sapiens tetraspanin 17 (TSPAN17), mRNA [NM_012171]	**1.199**
JMJD4	Homo sapiens jumonji domain containing 4 (JMJD4), mRNA [NM_023007]	**1.198**
PSMD13	Homo sapiens proteasome (prosome, macropain) 26S subunit, 13 (PSMD13), mRNA [NM_175932]	**1.197**
RNF25	Homo sapiens ring finger protein 25 (RNF25), mRNA [NM_022453]	**1.196**
RPS19BP1	Homo sapiens ribosomal protein S19 binding protein 1 (RPS19BP1), mRNA [NM_194326]	**1.196**
TRAF4	Homo sapiens TNF receptor-associated factor 4 (TRAF4), mRNA [NM_004295]	**1.196**
TNFAIP8L3	Homo sapiens tumor necrosis factor, alpha-induced protein 8-like 3 (TNFAIP8L3), mRNA [NM_207381]	**1.195**
FADS2	Homo sapiens fatty acid desaturase 2 (FADS2), mRNA [NM_004265]	**1.195**
FBXW4	Homo sapiens F-box and WD repeat domain containing 4 (FBXW4), mRNA [NM_022039]	**1.194**
GTF2H4	Homo sapiens general transcription factor IIH, polypeptide 4, 52kDa (GTF2H4), mRNA [NM_001517]	**1.194**
SLC30A1	Homo sapiens solute carrier family 30 (zinc transporter), member 1 (SLC30A1), mRNA [NM_021194]	**1.194**
ST3GAL3	Homo sapiens ST3 beta-galactoside alpha-2,3-sialyltransferase 3 (ST3GAL3), mRNA [NM_174963]	**1.194**

CPEB1	Homo sapiens cytoplasmic polyadenylation element binding protein 1 (CPEB1), mRNA [NM_030594]	**1.193**
EPS8	Homo sapiens epidermal growth factor receptor pathway substrate 8 (EPS8), mRNA [NM_004447]	**1.192**
NFKBIB	Homo sapiens nuclear factor of kappa light polypeptide gene enhancer in B-cells inhibitor, beta (NFKBIB), mRNA [NM_002503]	**1.191**
KIAA1618	Homo sapiens mRNA for KIAA1618 protein, partial cds. [AB046838]	**1.191**
AKT1S1	Homo sapiens AKT1 substrate 1 (proline-rich) (AKT1S1), mRNA [NM_032375]	**1.189**
ASNA1	Homo sapiens arsA arsenite transporter, ATP-binding, homolog 1 (bacterial) (ASNA1), mRNA [NM_004317]	**1.189**
NIPSNAP1	Homo sapiens nipsnap homolog 1 (C. elegans) (NIPSNAP1), mRNA [NM_003634]	**1.189**
NSFL1C	Homo sapiens NSFL1 (p97) cofactor (p47) (NSFL1C), mRNA [NM_016143]	**1.189**
HSP90AB6P	Homo sapiens heat shock protein 90Bf (HSP90Bf) mRNA, [AY956767]	**1.188**
NBPF14	Homo sapiens neuroblastoma breakpoint family, member 14 (NBPF14), mRNA [NM_015383]	**1.188**
ATP6AP1	Homo sapiens ATPase, lysosomal accessory protein 1 (ATP6AP1), mRNA [NM_001183]	**1.188**
FSCN1	Homo sapiens fascin homolog 1, actin-bundling protein (FSCN1), mRNA [NM_003088]	**1.187**
GNA11	Homo sapiens GTP-binding protein alpha 11 (GA11) mRNA, partial cds. [L40630]	**1.187**
NFATC2IP	Homo sapiens nuclear factor of activated T-cells, cytoplasmic, calcineurin-dependent 2 interacting protein (NFATC2IP), mRNA [NM_032815]	**1.187**
EIF3B	Homo sapiens eukaryotic translation initiation factor 3, subunit B (EIF3B), mRNA [NM_001037283]	**1.186**
RBM9	Homo sapiens RNA binding motif protein 9 (RBM9), mRNA [NM_001031695]	**1.184**
MGC27348	Homo sapiens ribosomal protein S2 pseudogene, mRNA (cDNA clone IMAGE:4671259). [BC026177]	**1.183**
MPDU1	Homo sapiens mannose-P-dolichol utilization defect 1 (MPDU1),mRNA [NM_004870]	**1.183**
POLRMT	Homo sapiens polymerase (RNA) mitochondrial (DNA directed) (POLRMT), mRNA [NM_005035]	**1.183**
ABCA7	Homo sapiens ATP-binding cassette, sub-family A (ABC1), member 7 (ABCA7), mRNA [NM_019112]	**1.182**
ENG	Homo sapiens endoglin (ENG), mRNA [NM_000118]	**1.182**
STARD4	Homo sapiens StAR-related lipid transfer (START) domain containing 4 (STARD4), mRNA [NM_139164]	**1.182**
C1orf201	Homo sapiens chromosome 1 open reading frame 201 (C1orf201), mRNA [NM_178122]	**1.181**
COPE	Homo sapiens coatomer protein complex, subunit epsilon (COPE),mRNA [NM_199444]	**1.181**
KIAA1161	Homo sapiens KIAA1161 (KIAA1161), mRNA [NM_020702]	**1.181**

SRXN1	Homo sapiens sulfiredoxin 1 homolog (S. cerevisiae) (SRXN1), mRNA [NM_080725]	**1.181**
CANT1	Homo sapiens calcium activated nucleotidase 1 (CANT1), mRNA [NM_138793]	**1.181**
TMED9	Homo sapiens transmembrane emp24 protein transport domain containing 9 (TMED9), mRNA [NM_017510]	**1.180**
SHARPIN	Homo sapiens SHANK-associated RH domain interactor (SHARPIN), mRNA [NM_030974]	**1.180**
PER3	Homo sapiens period homolog 3 (Drosophila) (PER3), mRNA [NM_016831]	**1.179**
CBX4	Homo sapiens chromobox homolog 4 (Pc class homolog, Drosophila) (CBX4), mRNA [NM_003655]	**1.179**
MEPCE	Homo sapiens methylphosphate capping enzyme (MEPCE), mRNA [NM_019606]	**1.179**
BIN3	Homo sapiens bridging integrator 3 (BIN3), mRNA [NM_018688]	**1.178**
PHF16	Homo sapiens PHD finger protein 16 (PHF16), mRNA [NM_014735]	**1.178**
C1orf43	Homo sapiens chromosome 1 open reading frame 43 (C1orf43), mRNA [NM_138740]	**1.177**
LOC401218	Homo sapiens misc_RNA (LOC401218), miscRNA [XR_042344]	**1.177**
TP53INP2	Homo sapiens tumor protein p53 inducible nuclear protein 2 (TP53INP2), mRNA [NM_021202]	**1.177**
IRF3	Homo sapiens interferon regulatory factor 3 (IRF3), mRNA [NM_001571]	**1.176**
LRRC61	Homo sapiens leucine rich repeat containing 61 (LRRC61),mRNA [NM_023942]	**1.176**
ADCY6	Homo sapiens adenylate cyclase 6 (ADCY6), mRNA [NM_015270]	**1.175**
DDX24	Homo sapiens DEAD (Asp-Glu-Ala-Asp) box polypeptide 24 (DDX24), mRNA [NM_020414]	**1.175**
FAIM3	Homo sapiens Fas apoptotic inhibitory molecule 3 (FAIM3),mRNA [NM_005449]	**1.174**
MAPKAPK3	Homo sapiens mitogen-activated protein kinase-activated protein kinase 3 (MAPKAPK3), mRNA [NM_004635]	**1.174**
TP53I13	Homo sapiens tumor protein p53 inducible protein 13 (TP53I13), mRNA [NM_138349]	**1.174**
AFMID	Homo sapiens arylformamidase (AFMID), mRNA [NM_001010982]	**1.173**
FUBP3	Homo sapiens far upstream element (FUSE) binding protein 3 (FUBP3), mRNA [NM_003934]	**1.173**
IFIT2	Homo sapiens interferon-induced protein with tetratricopeptide repeats 2 (IFIT2), mRNA [NM_001547]	**1.172**
GRLF1	Homo sapiens glucocorticoid receptor DNA binding factor 1 (GRLF1), mRNA [NM_004491]	**1.171**
KCTD5	Homo sapiens potassium channel tetramerisation domain containing 5 (KCTD5), mRNA [NM_018992]	**1.171**
CDC2L5	Homo sapiens cell division cycle 2-like 5 (cholinesterase-related cell division controller) (CDC2L5), mRNA [NM_031267]	**1.171**
AHR	Homo sapiens aryl hydrocarbon receptor (AHR), mRNA [NM_001621]	**1.170**
DTNA	Homo sapiens dystrobrevin, alpha (DTNA), mRNA [NM_001392]	**1.169**

FBXW5	Homo sapiens F-box and WD repeat domain containing 5 (FBXW5), mRNA [NM_018998]	**1.169**
SMG5	Homo sapiens Smg-5 homolog, nonsense mediated mRNA decay factor (C. elegans) (SMG5), mRNA [NM_015327]	**1.169**
ISOC2	Homo sapiens isochorismatase domain containing 2 (ISOC2),mRNA [NM_024710]	**1.168**
INTS3	Homo sapiens integrator complex subunit 3 (INTS3), mRNA [NM_023015]	**1.168**
ZBTB38	Homo sapiens zinc finger and BTB domain containing 38 (ZBTB38), mRNA [NM_001080412]	**1.168**
GOLGA4	Homo sapiens golgi autoantigen, golgin subfamily a, 4 (GOLGA4), mRNA [NM_002078]	**1.167**
RPP25	Homo sapiens ribonuclease P/MRP 25kDa subunit (RPP25), mRNA [NM_017793]	**1.167**
TXNDC11	Homo sapiens thioredoxin domain containing 11 (TXNDC11), mRNA [NM_015914]	**1.166**
SNRPB	Homo sapiens small nuclear ribonucleoprotein polypeptides B and B1 (SNRPB), mRNA [NM_198216]	**1.165**
THOC4	Homo sapiens THO complex 4 (THOC4), mRNA [NM_005782]	**1.164**
TJP2	Homo sapiens tight junction protein 2 (zona occludens 2) (TJP2),mRNA [NM_004817]	**1.164**
EEF1G	Homo sapiens eukaryotic translation elongation factor 1 gamma (EEF1G), mRNA [NM_001404]	**1.163**
TNIP1	Homo sapiens TNFAIP3 interacting protein 1 (TNIP1), mRNA [NM_006058]	**1.163**
EIF4A1	Homo sapiens eukaryotic translation initiation factor 4A, isoform 1 (EIF4A1), mRNA [NM_001416]	**1.162**
GLE1	Homo sapiens GLE1 RNA export mediator homolog (yeast) (GLE1),mRNA [NM_001499]	**1.162**
VEGFA	Homo sapiens vascular endothelial growth factor A (VEGFA), mRNA [NM_001025366]	**1.161**
HRASLS5	Homo sapiens HRAS-like suppressor family, member 5 (HRASLS5), mRNA [NM_054108]	**1.161**
VPS18	Homo sapiens vacuolar protein sorting 18 homolog (S. cerevisiae) (VPS18), mRNA [NM_020857]	**1.160**
SIRT6	Homo sapiens sirtuin (silent mating type information regulation 2 homolog) 6 (S. cerevisiae) (SIRT6), mRNA [NM_016539]	**1.159**
C17orf59	Homo sapiens chromosome 17 open reading frame 59 (C17orf59), mRNA [NM_017622]	**1.158**
NCOA4	Homo sapiens nuclear receptor coactivator 4 (NCOA4), mRNA [NM_005437]	**1.158**
BEX2	Homo sapiens brain expressed X-linked 2 (BEX2), mRNA [NM_032621]	**1.157**
DDX3Y	Homo sapiens DEAD (Asp-Glu-Ala-Asp) box polypeptide 3, Y-linked (DDX3Y), mRNA [NM_004660]	**1.157**
SART3	Homo sapiens squamous cell carcinoma antigen recognized by T cells 3 (SART3), mRNA [NM_014706]	**1.157**

NADK	Homo sapiens NAD kinase (NADK), mRNA [NM_023018]	**1.157**
ATG4D	Homo sapiens ATG4 autophagy related 4 homolog D (S. cerevisiae) (ATG4D), mRNA [NM_032885]	**1.156**
RSPRY1	Homo sapiens cDNA FLJ14643 fis, clone NT2RP2001597, weakly similar to Ryanodine Receptor, Cardiac muscle. [AK027549]	**1.156**
LYPLA2P1	Homo sapiens lysophospholipase II pseudogene 1 (LYPLA2P1), non-coding RNA [NR_001444]	**1.155**
C8orf55	Homo sapiens chromosome 8 open reading frame 55 (C8orf55), mRNA [NM_016647]	**1.153**
METTL7B	Homo sapiens methyltransferase like 7B (METTL7B), mRNA [NM_152637]	**1.153**
BCL7C	Homo sapiens B-cell CLL/lymphoma 7C (BCL7C), mRNA [NM_004765]	**1.152**
TYMP	Homo sapiens thymidine phosphorylase (TYMP), mRNA [NM_001113756]	**1.152**
POLD2	Homo sapiens polymerase (DNA directed), delta 2, regulatory subunit (POLD2), mRNA [NM_006230]	**1.151**
PRPF4	Homo sapiens PRP4 pre-mRNA processing factor 4 homolog (yeast) (PRPF4), mRNA [NM_004697]	**1.151**
CRMP1	Homo sapiens collapsin response mediator protein 1 (CRMP1),mRNA [NM_001014809]	**1.151**
CLIC2	Homo sapiens chloride intracellular channel 2 (CLIC2), mRNA [NM_001289]	**1.150**
HOXA5	Homo sapiens homeobox A5 (HOXA5), mRNA [NM_019102]	**1.150**
SPANXN3	Homo sapiens SPANX family, member N3 (SPANXN3), mRNA [NM_001009609]	**1.150**
HNRNPCL1	Homo sapiens heterogeneous nuclear ribonucleoprotein C-like 1 (HNRNPCL1), mRNA [NM_001013631]	**1.148**
SLC35B2	Homo sapiens solute carrier family 35, member B2 (SLC35B2), mRNA [NM_178148]	**1.148**
RAB40AL	Homo sapiens RAB40A, member RAS oncogene family-like (RAB40AL), mRNA [NM_001031834]	**1.147**
NCOA5	Homo sapiens nuclear receptor coactivator 5 (NCOA5), mRNA [NM_020967]	**1.147**
PACSIN2	Homo sapiens mRNA; cDNA DKFZp434H1130 (from clone DKFZp434H1130). [AL136845]	**1.146**
SPHK1	Homo sapiens sphingosine kinase 1 (SPHK1), mRNA [NM_021972]	**1.145**
ARL4D	Homo sapiens ADP-ribosylation factor-like 4D (ARL4D), mRNA [NM_001661]	**1.145**
TRIM25	Homo sapiens tripartite motif-containing 25 (TRIM25), mRNA [NM_005082]	**1.144**
LOC402026	PREDICTED: Homo sapiens misc_RNA (LOC402026), miscRNA [XR_019171]	**1.143**
TOX3	Homo sapiens TOX high mobility group box family member 3 (TOX3), mRNA [NM_001080430]	**1.142**
SMARCC1	Homo sapiens SWI/SNF related, matrix associated, actin dependent regulator of chromatin, subfamily c, member 1 (SMARCC1), mRNA [NM_003074]	**1.141**
MEN1	Homo sapiens multiple endocrine neoplasia I (MEN1), mRNA [NM_130803]	**1.140**
SOCS3	Homo sapiens suppressor of cytokine signaling 3 (SOCS3), mRNA [NM_003955]	**1.140**
ADAM17	Homo sapiens ADAM metallopeptidase domain 17 (ADAM17), mRNA [NM_003183]	**1.140**

LARP1	Homo sapiens La ribonucleoprotein domain family, member 1 (LARP1), mRNA [NM_033551]	**1.139**
ENO2	Homo sapiens enolase 2 (gamma, neuronal) (ENO2), mRNA [NM_001975]	**1.139**
PFKL	Homo sapiens phosphofructokinase, liver (PFKL), mRNA [NM_002626]	**1.139**
SPG7	Homo sapiens spastic paraplegia 7 (pure and complicated autosomal recessive) (SPG7), mRNA [NM_003119]	**1.139**
ARPC1B	Homo sapiens actin related protein 2/3 complex, subunit 1B, 41kDa (ARPC1B), mRNA [NM_005720]	**1.138**
CRAT	Homo sapiens carnitine acetyltransferase (CRAT), mRNA [NM_000755]	**1.138**
PSMA7	Homo sapiens proteasome (prosome, macropain) subunit, alpha type, 7 (PSMA7), mRNA [NM_002792]	**1.138**
SULT1A1	Homo sapiens sulfotransferase family, cytosolic, 1A, phenol-preferring, member 1 (SULT1A1), mRNA [NM_177529]	**1.138**
LITAF	Homo sapiens lipopolysaccharide-induced TNF factor (LITAF),mRNA [NM_004862]	**1.137**
CD180	Homo sapiens CD180 molecule (CD180), mRNA [NM_005582]	**1.136**
LGALS3BP	Homo sapiens lectin, galactoside-binding, soluble, 3 binding protein (LGALS3BP), mRNA [NM_005567]	**1.136**
USP40	Homo sapiens ubiquitin specific peptidase 40 (USP40), mRNA [NM_018218]	**1.136**
ANKRD11	Homo sapiens ankyrin repeat domain 11 (ANKRD11), mRNA [NM_013275]	**1.136**
AP4M1	Homo sapiens adaptor-related protein complex 4, mu 1 subunit (AP4M1), mRNA [NM_004722]	**1.135**
NOP14	Homo sapiens NOP14 nucleolar protein homolog (yeast) (NOP14), mRNA [NM_003703]	**1.135**
ZFAND3	Homo sapiens zinc finger, AN1-type domain 3 (ZFAND3), mRNA [NM_021943]	**1.134**
DDX56	Homo sapiens cDNA FLJ43607 fis, clone SPLEN2010912, highly similar to Homo sapiens nucleolar RNA helicase (NOH61). [AK125595]	**1.134**
B4GALT7	Homo sapiens xylosylprotein beta 1,4-galactosyltransferase, polypeptide 7 (galactosyltransferase I) (B4GALT7), mRNA [NM_007255]	**1.133**
FAM3A	Homo sapiens family with sequence similarity 3, member A (FAM3A), mRNA [NM_021806]	**1.133**
LGMN	Homo sapiens legumain (LGMN), mRNA [NM_001008530]	**1.133**
SDHC	Homo sapiens succinate dehydrogenase complex, subunit C, integral membrane protein, 15kDa (SDHC), mRNA [NM_003001]	**1.133**
HIRIP3	Homo sapiens HIRA interacting protein 3 (HIRIP3), mRNA [NM_003609]	**1.132**
PPP1R12C	Homo sapiens protein phosphatase 1, regulatory (inhibitor) subunit 12C (PPP1R12C), mRNA [NM_017607]	**1.132**
C22orf13	Homo sapiens chromosome 22 open reading frame 13 (C22orf13), mRNA [NM_031444]	**1.131**
FKBPL	Homo sapiens FK506 binding protein like (FKBPL), mRNA [NM_022110]	**1.130**
LOC341230	PREDICTED: Homo sapiens misc_RNA (LOC341230), miscRNA [XR_018617]	**1.130**
RFC2	Homo sapiens replication factor C (activator 1) 2, 40kDa (RFC2), mRNA [NM_181471]	**1.130**

RNF5	Homo sapiens ring finger protein 5 (RNF5), mRNA [NM_006913]	**1.130**
RPS6KA4	Homo sapiens ribosomal protein S6 kinase, 90kDa, polypeptide 4 (RPS6KA4), mRNA [NM_003942]	**1.129**
C9orf109	Homo sapiens chromosome 9 open reading frame 109 (C9orf109), non-coding RNA [NR_024366]	**1.128**
H2AFY	Homo sapiens H2A histone family, member Y (H2AFY), mRNA [NM_138610]	**1.128**
RAB11B	Homo sapiens RAB11B, member RAS oncogene family (RAB11B), mRNA [NM_004218]	**1.128**
TRAF2	Homo sapiens TNF receptor-associated factor 2 (TRAF2), mRNA [NM_021138]	**1.128**
MRPL49	Homo sapiens mitochondrial ribosomal protein L49 (MRPL49),mRNA [NM_004927]	**1.127**
C1orf85	Homo sapiens chromosome 1 open reading frame 85 (C1orf85), mRNA [NM_144580]	**1.127**
PICALM	Homo sapiens phosphatidylinositol binding clathrin assembly protein (PICALM), mRNA [NM_007166]	**1.126**
RNF144B	Homo sapiens ring finger protein 144B (RNF144B), mRNA [NM_182757]	**1.126**
SBF1	Homo sapiens SET binding factor 1 (SBF1), mRNA [NM_002972]	**1.126**
CDC2L1	Homo sapiens cell division cycle 2-like 1 (PITSLRE proteins) (CDC2L1), mRNA [NM_033489]	**1.126**
WRNIP1	Homo sapiens Werner helicase interacting protein 1 (WRNIP1),mRNA [NM_130395]	**1.124**
ZNF644	Homo sapiens zinc finger protein 644 (ZNF644), mRNA [NM_201269]	**1.122**
TAPBPL	Homo sapiens TAP binding protein-like (TAPBPL), mRNA [NM_018009]	**1.122**
C6orf145	Homo sapiens chromosome 6 open reading frame 145 (C6orf145), mRNA [NM_183373]	**1.121**
EDARADD	Homo sapiens EDAR-associated death domain (EDARADD),mRNA [NM_080738]	**1.121**
KLHDC8B	Homo sapiens kelch domain containing 8B (KLHDC8B), mRNA [NM_173546]	**1.120**
STUB1	Homo sapiens STIP1 homology and U-box containing protein 1 (STUB1), mRNA [NM_005861]	**1.120**
COG4	Homo sapiens component of oligomeric golgi complex 4 (COG4), mRNA [NM_015386]	**1.119**
TOLLIP	Homo sapiens toll interacting protein (TOLLIP), mRNA [NM_019009]	**1.119**
CARD16	Homo sapiens caspase recruitment domain family, member 16 (CARD16), mRNA [NM_001017534]	**1.117**
GNB2L1	Homo sapiens guanine nucleotide binding protein (G protein), beta polypeptide 2-like 1 (GNB2L1), mRNA [NM_006098]	**1.117**
LOC441455	Homo sapiens similar to Makorin-1 (RING finger protein 61), mRNA (cDNA clone IMAGE:5556543),[BC031344]	**1.117**
SH3GLB2	Homo sapiens SH3-domain GRB2-like endophilin B2 (SH3GLB2), mRNA [NM_020145]	**1.117**
PPP2R4	Homo sapiens protein phosphatase 2A activator, regulatory subunit 4 (PPP2R4), mRNA [NM_178001]	**1.116**

SNRNP70	Homo sapiens small nuclear ribonucleoprotein 70kDa (U1) (SNRNP70), mRNA [NM_003089]	**1.116**
FLAD1	Homo sapiens FAD1 flavin adenine dinucleotide synthetase homolog (S. cerevisiae) (FLAD1), mRNA [NM_025207]	**1.116**
EXOSC5	Homo sapiens exosome component 5 (EXOSC5), mRNA [NM_020158]	**1.114**
FAH	Homo sapiens fumarylacetoacetate hydrolase (fumarylacetoacetase) (FAH), mRNA [NM_000137]	**1.114**
FDPS	Homo sapiens farnesyl diphosphate synthase (farnesyl pyrophosphate synthetase, dimethylallyltranstransferase, geranyltranstransferase) (FDPS), mRNA [NM_002004]	**1.114**
HYAL2	Homo sapiens hyaluronoglucosaminidase 2 (HYAL2), mRNA [NM_003773]	**1.114**
NQO2	Homo sapiens NAD(P)H dehydrogenase, quinone 2 (NQO2), mRNA [NM_000904]	**1.114**
RCOR2	Homo sapiens REST corepressor 2 (RCOR2), mRNA [NM_173587]	**1.114**
AIP	Homo sapiens aryl hydrocarbon receptor interacting protein (AIP), mRNA [NM_003977]	**1.113**
C8orf41	Homo sapiens chromosome 8 open reading frame 41 (C8orf41),mRNA [NM_025115]	**1.113**
LOC100131067	Homo sapiens cDNA FLJ38695 fis, clone KIDNE2001897. [AK096014]	**1.113**
NCDN	Homo sapiens neurochondrin (NCDN), mRNA [NM_014284]	**1.113**
STK35	Homo sapiens serine/threonine kinase 35 (STK35), mRNA [NM_080836]	**1.113**
DHRS9	Homo sapiens dehydrogenase/reductase (SDR family) member 9 (DHRS9), transcript variant 1, mRNA [NM_005771]	**1.111**
GALNS	Homo sapiens galactosamine (N-acetyl)-6-sulfate sulfatase (GALNS), mRNA [NM_000512]	**1.111**
TRMT2A	Homo sapiens TRM2 tRNA methyltransferase 2 homolog A (S. cerevisiae) (TRMT2A), mRNA [NM_022727]	**1.111**
MED12	Homo sapiens mediator complex subunit 12 (MED12), mRNA [NM_005120]	**1.110**
MGC2752	Homo sapiens hypothetical LOC65996 (MGC2752), non-coding RNA [NR_026052]	**1.110**
TTC5	Homo sapiens tetratricopeptide repeat domain 5 (TTC5), mRNA [NM_138376]	**1.110**
NOC2L	Homo sapiens nucleolar complex associated 2 homolog (S. cerevisiae) (NOC2L), mRNA [NM_015658]	**1.109**
HSP90AA1	Homo sapiens heat shock protein 90kDa alpha (cytosolic), class A member 1 (HSP90AA1), mRNA [NM_005348]	**1.109**
SH3BP5	Homo sapiens SH3-domain binding protein 5 (BTK-associated) (SH3BP5), mRNA [NM_004844]	**1.108**
HIF1AN	Homo sapiens hypoxia inducible factor 1, alpha subunit inhibitor (HIF1AN), mRNA [NM_017902]	**1.107**
TRPM7	Homo sapiens transient receptor potential cation channel, subfamily M, member 7 (TRPM7), mRNA [NM_017672]	**1.107**
FLOT1	Homo sapiens flotillin 1 (FLOT1), mRNA [NM_005803]	**1.107**
TMED2	Homo sapiens transmembrane emp24 domain trafficking protein 2 (TMED2),	**1.107**

	mRNA [NM_006815]	
KATNB1	Homo sapiens katanin p80 (WD repeat containing) subunit B 1 (KATNB1), mRNA [NM_005886]	**1.106**
MYO1E	Homo sapiens myosin IE (MYO1E), mRNA [NM_004998]	**1.106**
EXOC6B	Homo sapiens cDNA FLJ13729 fis, clone PLACE3000121, weakly similar to Vesicular Traffic Control ProteinO Sec15. [AK023791]	**1.105**
LMF2	Homo sapiens lipase maturation factor 2 (LMF2), mRNA [NM_033200]	**1.104**
NAGK	Homo sapiens N-acetylglucosamine kinase (NAGK), mRNA [NM_017567]	**1.104**
LMNA	Homo sapiens lamin A/C (LMNA), mRNA [NM_005572]	**1.104**
CYC1	Homo sapiens cytochrome c-1 (CYC1), mRNA [NM_001916]	**1.103**
PMS2CL	Homo sapiens PMS2 C-terminal like pseudogene, mRNA (cDNA clone IMAGE:5273238). [BC041364]	**1.103**
ZNF668	Homo sapiens zinc finger protein 668 (ZNF668), mRNA [NM_024706]	**1.103**
CIC	Homo sapiens capicua homolog (Drosophila) (CIC), mRNA [NM_015125]	**1.100**
SIK1	Homo sapiens salt-inducible kinase 1 (SIK1), mRNA [NM_173354]	**1.100**
SLC6A6	Homo sapiens solute carrier family 6 (neurotransmitter transporter, taurine), member 6 (SLC6A6), mRNA [NM_003043]	**1.099**
NDRG1	Homo sapiens N-myc downstream regulated 1 (NDRG1), mRNA [NM_006096]	**1.098**
CDK10	Homo sapiens cyclin-dependent kinase 10 (CDK10), mRNA [NM_052987]	**1.098**
NPLOC4	Homo sapiens nuclear protein localization 4 homolog (S. cerevisiae) (NPLOC4), mRNA [NM_017921]	**1.097**
NQO1	Homo sapiens NAD(P)H dehydrogenase, quinone 1 (NQO1),mRNA [NM_000903]	**1.097**
PNPLA3	Homo sapiens patatin-like phospholipase domain containing 3 (PNPLA3), mRNA [NM_025225]	**1.097**
VCP	Homo sapiens valosin-containing protein (VCP), mRNA [NM_007126]	**1.097**
TBCD	Homo sapiens tubulin folding cofactor D (TBCD), mRNA [NM_005993]	**1.096**
GORASP1	Homo sapiens golgi reassembly stacking protein 1, 65kDa (GORASP1), mRNA [NM_031899]	**1.095**
ITGB5	Homo sapiens integrin, beta 5 (ITGB5), mRNA [NM_002213]	**1.095**
SF4	Homo sapiens splicing factor 4 (SF4), mRNA [NM_172231]	**1.095**
VAT1	Homo sapiens vesicle amine transport protein 1 homolog (T. californica) (VAT1), mRNA [NM_006373]	**1.095**
SAMD1	Homo sapiens sterile alpha motif domain containing 1 (SAMD1), mRNA [NM_138352]	**1.095**
ACBD4	Homo sapiens acyl-Coenzyme A binding domain containing 4 (ACBD4), mRNA [NM_024722]	**1.094**
HRK	Homo sapiens harakiri, BCL2 interacting protein (contains only BH3 domain) (HRK), mRNA [NM_003806]	**1.093**
PHB	Homo sapiens prohibitin (PHB), mRNA [NM_002634]	**1.093**
GABARAPL1	Homo sapiens GABA(A) receptor-associated protein like 1 (GABARAPL1), mRNA [NM_031412]	**1.093**
ITSN1	Homo sapiens intersectin 1 (SH3 domain protein) (ITSN1),mRNA	**1.092**

	[NM_001001132]	
UBAP1	Homo sapiens ubiquitin associated protein 1 (UBAP1), mRNA [NM_016525]	**1.092**
HOXA10	Homo sapiens homeobox A10 (HOXA10), mRNA [NM_018951]	**1.092**
EPHB4	Homo sapiens EPH receptor B4 (EPHB4), mRNA [NM_004444]	**1.091**
MGC16703	Homo sapiens tubulin, alpha pseudogene (MGC16703), non-coding RNA [NR_003608]	**1.091**
CLPTM1	Homo sapiens cleft lip and palate associated transmembrane protein 1 (CLPTM1), mRNA [NM_001294]	**1.090**
GALK1	Homo sapiens galactokinase 1 (GALK1), mRNA [NM_000154]	**1.090**
GTPBP6	Homo sapiens GTP binding protein 6 (putative) (GTPBP6), mRNA [NM_012227]	**1.089**
FLCN	Homo sapiens mRNA; cDNA DKFZp547A118 (from clone DKFZp547A118). [AL831885]	**1.087**
THOC6	Homo sapiens THO complex 6 homolog (Drosophila) (THOC6),mRNA [NM_024339]	**1.087**
ZMYND19	Homo sapiens zinc finger, MYND-type containing 19 (ZMYND19), mRNA [NM_138462]	**1.087**
SCAMP2	Homo sapiens secretory carrier membrane protein 2 (SCAMP2), mRNA [NM_005697]	**1.086**
SLC39A14	Homo sapiens solute carrier family 39 (zinc transporter), member 14 (SLC39A14), mRNA [NM_015359]	**1.086**
ARF1	Homo sapiens ADP-ribosylation factor 1 (ARF1), mRNA [NM_001024227]	**1.085**
PTPRR	Homo sapiens protein tyrosine phosphatase, receptor type, R (PTPRR), mRNA [NM_002849]	**1.085**
NT5C3L	Homo sapiens 5'-nucleotidase, cytosolic III-like (NT5C3L),mRNA [NM_052935]	**1.084**
RGS19	Homo sapiens regulator of G-protein signaling 19 (RGS19), mRNA [NM_005873]	**1.084**
NFRKB	Homo sapiens nuclear factor related to kappaB binding protein (NFRKB), mRNA [NM_006165]	**1.083**
RBM23	Homo sapiens RNA binding motif protein 23 (RBM23), mRNA [NM_001077351]	**1.082**
CD320	Homo sapiens CD320 molecule (CD320), mRNA [NM_016579]	**1.082**
DNM2	Homo sapiens dynamin 2 (DNM2), mRNA [NM_001005360]	**1.081**
F2RL1	Homo sapiens coagulation factor II (thrombin) receptor-like 1 (F2RL1), mRNA [NM_005242]	**1.081**
MTUS1	Homo sapiens mitochondrial tumor suppressor 1 (MTUS1),mRNA [NM_001001924]	**1.081**
LRRC47	Homo sapiens leucine rich repeat containing 47 (LRRC47), mRNA [NM_020710]	**1.081**
SCYL1	Homo sapiens SCY1-like 1 (S. cerevisiae) (SCYL1), mRNA [NM_001048218]	**1.080**
FNIP2	Homo sapiens folliculin interacting protein 2 (FNIP2), mRNA [NM_020840]	**1.080**
FEN1	Homo sapiens flap structure-specific endonuclease 1 (FEN1), mRNA [NM_004111]	**1.079**
CLCN2	Homo sapiens chloride channel 2 (CLCN2), mRNA [NM_004366]	**1.078**
DEDD2	Homo sapiens death effector domain containing 2 (DEDD2), mRNA	**1.078**

	[NM_133328]	
MADD	Homo sapiens MAP-kinase activating death domain (MADD),mRNA [NM_003682]	**1.078**
AKT1	Homo sapiens v-akt murine thymoma viral oncogene homolog 1 (AKT1), mRNA [NM_005163]	**1.077**
ZIC2	Homo sapiens Zic family member 2 (odd-paired homolog, Drosophila) (ZIC2), mRNA [NM_007129]	**1.077**
IPO8	Homo sapiens importin 8 (IPO8), mRNA [NM_006390]	**1.076**
LAMA5	Homo sapiens laminin, alpha 5 (LAMA5), mRNA [NM_005560]	**1.076**
NME6	Homo sapiens non-metastatic cells 6, protein expressed in (nucleoside-diphosphate kinase) (NME6), mRNA [NM_005793]	**1.076**
N-PAC	Homo sapiens cytokine-like nuclear factor n-pac (N-PAC), mRNA [NM_032569]	**1.076**
TWIST1	Homo sapiens twist homolog 1 (Drosophila) (TWIST1), mRNA [NM_000474]	**1.076**
TMEM192	Homo sapiens transmembrane protein 192 (TMEM192), mRNA [NM_001100389]	**1.076**
BCL2L1	Homo sapiens BCL2-like 1 (BCL2L1), mRNA [NM_138578]	**1.075**
PIH1D1	Homo sapiens PIH1 domain containing 1 (PIH1D1), mRNA [NM_017916]	**1.074**
TULP3	Homo sapiens tubby like protein 3 (TULP3) mRNA, complete cds. [AF045583]	**1.074**
GRHPR	Homo sapiens glyoxylate reductase/hydroxypyruvate reductase (GRHPR), mRNA [NM_012203]	**1.074**
CDK2AP2	Homo sapiens cyclin-dependent kinase 2 associated protein 2 (CDK2AP2), mRNA [NM_005851]	**1.073**
VPS26A	Homo sapiens vacuolar protein sorting 26 homolog A (S. pombe) (VPS26A), mRNA [NM_004896]	**1.073**
PLEKHG1	Homo sapiens pleckstrin homology domain containing, family G (with RhoGef domain) member 1 (PLEKHG1), mRNA [NM_001029884]	**1.072**
MYH9	Homo sapiens myosin, heavy chain 9, non-muscle (MYH9), mRNA [NM_002473]	**1.072**
NPTN	Homo sapiens neuroplastin (NPTN), transcript variant beta, mRNA [NM_012428]	**1.071**
UVRAG	Homo sapiens UV radiation resistance associated gene (UVRAG), mRNA [NM_003369]	**1.071**
BRD9	Homo sapiens bromodomain containing 9 (BRD9), mRNA [NM_023924]	**1.070**
DULLARD	Homo sapiens dullard homolog (Xenopus laevis) (DULLARD), mRNA [NM_015343]	**1.070**
SHMT1	Homo sapiens mRNA for putative 14kD protein containing SHMT homology, clone pUS1215. [Y14488]	**1.070**
PPAN	Homo sapiens peter pan homolog (Drosophila) (PPAN), mRNA [NM_020230]	**1.070**
ARF5	Homo sapiens ADP-ribosylation factor 5 (ARF5), mRNA [NM_001662]	**1.069**
MTP18	Homo sapiens mitochondrial protein 18 kDa (MTP18), mRNA [NM_016498]	**1.069**
TMEM159	Homo sapiens transmembrane protein 159 (TMEM159), mRNA [NM_020422]	**1.069**
TNFRSF14	Homo sapiens tumor necrosis factor receptor superfamily, member 14 (herpesvirus entry mediator) (TNFRSF14), mRNA [NM_003820]	**1.069**
PLP2	Homo sapiens proteolipid protein 2 (colonic epithelium-enriched) (PLP2), mRNA	**1.068**

	[NM_002668]	
CXorf56	Homo sapiens chromosome X open reading frame 56 (CXorf56), mRNA [NM_022101]	**1.068**
GGTLC2	Homo sapiens gamma-glutamyltransferase light chain 2 (GGTLC2), mRNA [NM_199127]	**1.067**
CDK9	Homo sapiens cyclin-dependent kinase 9 (CDK9), mRNA [NM_001261]	**1.066**
GGTLC3	Gamma-glutamyl transpeptidase 1 Precursor (EC 2.3.2.2) (Gamma-glutamyltransferase 1)(GGT 1)(CD224 antigen) [Source:UniProtKB/ Swiss-Prot;Acc:P19440] [ENST00000404223]	**1.066**
IFRD2	Homo sapiens interferon-related developmental regulator 2 (IFRD2), mRNA [NM_006764]	**1.066**
MAP3K12	Homo sapiens mitogen-activated protein kinase kinase kinase 12 (MAP3K12), mRNA [NM_006301]	**1.066**
C12orf44	Homo sapiens chromosome 12 open reading frame 44 (C12orf44), mRNA [NM_021934]	**1.065**
LPAR5	Homo sapiens lysophosphatidic acid receptor 5 (LPAR5), mRNA [NM_020400]	**1.065**
UST	Homo sapiens uronyl-2-sulfotransferase (UST), mRNA [NM_005715]	**1.065**
TUBA3C	Homo sapiens tubulin, alpha 3c (TUBA3C), mRNA [NM_006001]	**1.064**
BAG3	Homo sapiens BCL2-associated athanogene 3 (BAG3), mRNA [NM_004281]	**1.064**
C17orf70	Homo sapiens chromosome 17 open reading frame 70 (C17orf70),mRNA [NM_025161]	**1.062**
EIF6	Homo sapiens eukaryotic translation initiation factor 6 (EIF6),mRNA [NM_181468]	**1.062**
HOOK2	Homo sapiens hook homolog 2 (Drosophila) (HOOK2), mRNA [NM_013312]	**1.062**
DLST	Homo sapiens dihydrolipoamide S-succinyltransferase (E2 component of 2-oxo-glutarate complex) (DLST), mRNA [NM_001933]	**1.061**
DAXX	Homo sapiens death-domain associated protein (DAXX), mRNA [NM_001350]	**1.060**
LOC643960	PREDICTED: Homo sapiens misc_RNA (LOC643960), miscRNA [XR_019250]	**1.060**
PIN1	Homo sapiens peptidylprolyl cis/trans isomerase, NIMA-interacting 1 (PIN1), mRNA [NM_006221]	**1.060**
TRIP13	Homo sapiens thyroid hormone receptor interactor 13 (TRIP13), mRNA [NM_004237]	**1.060**
WBP2	Homo sapiens WW domain binding protein 2 (WBP2), mRNA [NM_012478]	**1.060**
CDK5RAP2	Homo sapiens CDK5 regulatory subunit associated protein 2 (CDK5RAP2), mRNA [NM_018249]	**1.059**
HCCA2	Homo sapiens HCCA2 protein (HCCA2), mRNA [NM_053005]	**1.059**
PLAUR	Homo sapiens plasminogen activator, urokinase receptor (PLAUR), mRNA [NM_001005377]	**1.059**
CD276	Homo sapiens CD276 molecule (CD276), mRNA [NM_001024736]	**1.058**
ADAMTS16	Homo sapiens ADAM metallopeptidase with thrombospondin type 1 motif, 16 (ADAMTS16), mRNA [NM_139056]	**1.057**
ZNF28	Homo sapiens zinc finger protein 28 (ZNF28), mRNA [NM_006969]	**1.057**
NOV	Homo sapiens nephroblastoma overexpressed gene (NOV), mRNA [NM_002514]	**1.057**

ACSL4	Homo sapiens acyl-CoA synthetase long-chain family member 4 (ACSL4), mRNA [NM_004458]	**1.056**
ACTR1B	Homo sapiens ARP1 actin-related protein 1 homolog B, centractin beta (yeast) (ACTR1B), mRNA [NM_005735]	**1.056**
SREBF2	Homo sapiens sterol regulatory element binding transcription factor 2 (SREBF2), mRNA [NM_004599]	**1.056**
ZBTB41	Homo sapiens zinc finger and BTB domain containing 41 (ZBTB41), mRNA [NM_194314]	**1.056**
GPR115	Homo sapiens G protein-coupled receptor 115 (GPR115), mRNA [NM_153838]	**1.055**
KRT72	Homo sapiens keratin 72 (KRT72), mRNA [NM_080747]	**1.055**
ZC3H18	Homo sapiens zinc finger CCCH-type containing 18 (ZC3H18), mRNA [NM_144604]	**1.055**
LOC644214	PREDICTED: Homo sapiens misc_RNA (LOC644214), miscRNA [XR_018965]	**1.054**
B3GNTL1	Homo sapiens UDP-GlcNAc:betaGal beta-1,3-N-acetyl glucosaminyl transferase-like 1 (B3GNTL1), mRNA [NM_001009905]	**1.053**
CXXC1	Homo sapiens CXXC finger 1 (PHD domain) (CXXC1), mRNA [NM_014593]	**1.053**
KCNN4	Homo sapiens potassium intermediate/small conductance calcium-activated channel, subfamily N, member 4 (KCNN4), mRNA [NM_002250]	**1.053**
LOC646808	Homo sapiens misc_RNA (LOC646808), miscRNA [XR_017339]	**1.053**
GLTSCR2	Homo sapiens glioma tumor suppressor candidate region gene 2 (GLTSCR2), mRNA [NM_015710]	**1.052**
FAM83H	Homo sapiens family with sequence similarity 83, member H (FAM83H), mRNA [NM_198488]	**1.051**
LOC148413	Homo sapiens hypothetical LOC148413 (LOC148413), non-coding RNA [NR_015434]	**1.051**
LOC285412	PREDICTED: Homo sapiens misc_RNA (LOC285412), miscRNA [XR_019409]	**1.051**
PDIA4	Homo sapiens protein disulfide isomerase family A, member 4 (PDIA4), mRNA [NM_004911]	**1.051**
PIWIL2	Homo sapiens piwi-like 2 (Drosophila) (PIWIL2), mRNA [NM_018068]	**1.051**
UBE2MP1	Homo sapiens ubiquitin-conjugating enzyme E2M pseudogene 1 (UBE2MP1), non-coding RNA [NR_002837]	**1.051**
ZNF346	Homo sapiens zinc finger protein 346 (ZNF346), mRNA [NM_012279]	**1.051**
HIST1H4C	Homo sapiens histone cluster 1, H4c (HIST1H4C), mRNA [NM_003542]	**1.050**
EVX1	Homo sapiens even-skipped homeobox 1 (EVX1), mRNA [NM_001989]	**1.049**
NDUFB7	Homo sapiens NADH dehydrogenase (ubiquinone) 1 beta subcomplex, 7, 18kDa (NDUFB7), mRNA [NM_004146]	**1.049**
TMEM134	Homo sapiens transmembrane protein 134 (TMEM134), mRNA [NM_025124]	**1.049**
GNB1	Homo sapiens guanine nucleotide binding protein (G protein), beta polypeptide 1 (GNB1), mRNA [NM_002074]	**1.048**
RPL7L1	Homo sapiens ribosomal protein L7-like 1 (RPL7L1), mRNA [NM_198486]	**1.048**
SART1	Homo sapiens squamous cell carcinoma antigen recognized by T cells (SART1), mRNA [NM_005146]	**1.047**
BBX	Homo sapiens bobby sox homolog (Drosophila) (BBX), mRNA [NM_020235]	**1.046**

C9orf64	Homo sapiens chromosome 9 open reading frame 64 (C9orf64), mRNA [NM_032307]	**1.046**
DPP9	Homo sapiens dipeptidyl-peptidase 9 (DPP9), mRNA [NM_139159]	**1.046**
KIAA1627	Homo sapiens KIAA1627 protein (KIAA1627), mRNA [NM_020961]	**1.046**
LOC100132831	PREDICTED: Homo sapiens misc_RNA (LOC100132831), miscRNA [XR_037012]	**1.046**
CCM2	Homo sapiens cerebral cavernous malformation 2 (CCM2), mRNA [NM_001029835]	**1.042**
CPSF1	Homo sapiens cleavage and polyadenylation specific factor 1, (CPSF1), mRNA [NM_013291]	**1.042**
PRMT1	Homo sapiens protein arginine methyltransferase 1 (PRMT1),mRNA [NM_198319]	**1.042**
SLC2A14	Homo sapiens solute carrier family 2 (facilitated glucose transporter), member 14, mRNA (cDNA clone MGC:71510 IMAGE:5297510), [BC060766]	**1.042**
TAPBP	Homo sapiens TAP binding protein (tapasin) (TAPBP), mRNA [NM_172208]	**1.042**
INO80	Homo sapiens INO80 homolog (S. cerevisiae) (INO80), mRNA [NM_017553]	**1.041**
SDSL	Homo sapiens serine dehydratase-like (SDSL), mRNA [NM_138432]	**1.040**
SNIP1	Homo sapiens Smad nuclear interacting protein 1 (SNIP1), mRNA [NM_024700]	**1.040**
SRM	Homo sapiens spermidine synthase (SRM), mRNA [NM_003132]	**1.040**
SCRN2	Homo sapiens secernin 2 (SCRN2), mRNA [NM_138355]	**1.039**
BAT2	Homo sapiens HLA-B associated transcript 2 (BAT2), mRNA [NM_080686]	**1.039**
PI4KB	Homo sapiens phosphatidylinositol 4-kinase, catalytic, beta (PI4KB), mRNA [NM_002651]	**1.038**
RELA	Homo sapiens v-rel reticuloendotheliosis viral oncogene homolog A (avian), mRNA (cDNA clone MGC:131774 IMAGE:6019711), [BC110830]	**1.038**
HLA-E	Homo sapiens major histocompatibility complex, class I, E (HLA-E), mRNA [NM_005516]	**1.038**
PRMT5	Homo sapiens protein arginine methyltransferase 5 (PRMT5), mRNA [NM_001039619]	**1.038**
CEACAM20	Homo sapiens carcinoembryonic antigen-related cell adhesion molecule 20 (CEACAM20), mRNA [NM_001102598]	**1.037**
MICALL1	Homo sapiens MICAL-like 1 (MICALL1), mRNA [NM_033386]	**1.037**
MMP15	Homo sapiens matrix metallopeptidase 15 (membrane-inserted) (MMP15), mRNA [NM_002428]	**1.037**
MRPS5	Homo sapiens mitochondrial ribosomal protein S5 (MRPS5),mRNA [NM_031902]	**1.037**
KIAA0495	Homo sapiens KIAA0495 (KIAA0495), mRNA [NM_207306]	**1.036**
RPUSD3	Homo sapiens RNA pseudouridylate synthase domain containing 3 (RPUSD3), mRNA [NM_173659]	**1.036**
BRD3	Homo sapiens bromodomain containing 3 (BRD3), mRNA [NM_007371]	**1.036**
L1CAM	Homo sapiens L1 cell adhesion molecule (L1CAM), mRNA [NM_024003]	**1.035**
MLF2	Homo sapiens myeloid leukemia factor 2 (MLF2), mRNA [NM_005439]	**1.035**
TP53	Homo sapiens tumor protein p53 (TP53), mRNA [NM_000546]	**1.035**

C6orf47	Homo sapiens chromosome 6 open reading frame 47 (C6orf47), mRNA [NM_021184]	**1.034**
OAZ2	Homo sapiens ornithine decarboxylase antizyme 2 (OAZ2), mRNA [NM_002537]	**1.034**
ASAH1	Homo sapiens cDNA: FLJ21558 fis, clone COL06372. [AK025211]	**1.034**
GADD45GIP1	Homo sapiens growth arrest and DNA-damage-inducible,(GADD45GIP1), mRNA [NM_052850]	**1.033**
CYBASC3	Homo sapiens cytochrome b, ascorbate dependent 3 (CYBASC3), mRNA [NM_153611]	**1.032**
ASL	Homo sapiens argininosuccinate lyase (ASL), mRNA [NM_001024943]	**1.031**
BSG	Homo sapiens basigin (Ok blood group) (BSG), mRNA [NM_001728]	**1.031**
HMGCR	Homo sapiens 3-hydroxy-3-methylglutaryl-Coenzyme A reductase (HMGCR), mRNA [NM_000859]	**1.030**
C2orf24	Homo sapiens chromosome 2 open reading frame 24 (C2orf24), mRNA [NM_015680]	**1.029**
CD81	Homo sapiens CD81 molecule (CD81), mRNA [NM_004356]	**1.029**
METT10D	Homo sapiens methyltransferase 10 domain containing (METT10D), mRNA [NM_024086]	**1.029**
NR1H2	Homo sapiens nuclear receptor subfamily 1, group H, member 2 (NR1H2), mRNA [NM_007121]	**1.028**
PSMD3	Homo sapiens proteasome (prosome, macropain) 26S subunit, non-ATPase, 3 (PSMD3), mRNA [NM_002809]	**1.028**
FKBP9L	Homo sapiens FK506 binding protein 9-like (FKBP9L), non-coding RNA [NR_003949]	**1.027**
ITPR3	Homo sapiens inositol 1,4,5-triphosphate receptor, type 3 (ITPR3), mRNA [NM_002224]	**1.027**
LOC100128295	PREDICTED: Homo sapiens similar to hCG1639947 (LOC100128295), mRNA [XM_001720544]	**1.027**
CDGAP	Homo sapiens Cdc42 GTPase-activating protein (CDGAP), mRNA [NM_020754]	**1.026**
CXXC5	Homo sapiens CXXC finger 5 (CXXC5), mRNA [NM_016463]	**1.026**
UTP14A	Homo sapiens UTP14, U3 small nucleolar ribonucleoprotein, homolog A (yeast) (UTP14A), mRNA [NM_006649]	**1.026**
PGS1	Homo sapiens phosphatidylglycerophosphate synthase 1 (PGS1), mRNA [NM_024419]	**1.025**
HAGHL	Homo sapiens hydroxyacylglutathione hydrolase-like (HAGHL),mRNA [NM_032304]	**1.025**
POLDIP2	Homo sapiens polymerase (DNA-directed), delta interacting protein 2 (POLDIP2), mRNA [NM_015584]	**1.025**
SFPQ	Homo sapiens splicing factor proline/glutamine-rich (polypyrimidine tract binding protein associated) (SFPQ), mRNA [NM_005066]	**1.025**
C1orf159	Homo sapiens chromosome 1 open reading frame 159 (C1orf159), mRNA [NM_017891]	**1.024**
CTNS	Homo sapiens cystinosis, nephropathic (CTNS), mRNA [NM_004937]	**1.024**
LOC200810	Homo sapiens beta-1,4-mannosyltransferase-like (LOC200810), mRNA	**1.024**

	[NM_001015050]	
C10orf58	Homo sapiens chromosome 10 open reading frame 58 (C10orf58),mRNA [NM_032333]	**1.023**
NUDC	Homo sapiens nuclear distribution gene C homolog (A. nidulans) (NUDC), mRNA [NM_006600]	**1.023**
CENPM	Homo sapiens centromere protein M (CENPM), mRNA [NM_001002876]	**1.022**
GRINA	Homo sapiens glutamate receptor, ionotropic, N-methyl D-aspartate-associated protein 1 (glutamate binding) (GRINA), mRNA [NM_000837]	**1.022**
CTRB2	Homo sapiens chymotrypsinogen B2 (CTRB2), mRNA [NM_001025200]	**1.021**
KIAA0319L	Homo sapiens KIAA0319-like (KIAA0319L), mRNA [NM_182686]	**1.020**
KRT17	Homo sapiens keratin 17 (KRT17), mRNA [NM_000422]	**1.019**
TRAF3IP2	Homo sapiens TRAF3 interacting protein 2 (TRAF3IP2), mRNA [NM_147686]	**1.018**
UBN1	Homo sapiens ubinuclein 1 (UBN1), transcript variant 1, mRNA [NM_016936]	**1.018**
ABCF1	Homo sapiens ATP-binding cassette, sub-family F (GCN20), member 1 (ABCF1), mRNA [NM_001025091]	**1.017**
MAD2L2	Homo sapiens MAD2 mitotic arrest deficient-like 2 (yeast) (MAD2L2), mRNA [NM_006341]	**1.017**
PPP2R3B	Homo sapiens protein phosphatase 2 (formerly 2A), regulatory subunit B'', beta (PPP2R3B), mRNA [NM_013239]	**1.017**
ALDH3B1	Homo sapiens cDNA FLJ26433 fis, clone KDN01585, highly similar to Aldehyde dehydrogenase 7 (EC 1.2.1.5). [AK129943]	**1.016**
TELO2	Homo sapiens TEL2, telomere maintenance 2, homolog (S. cerevisiae) (TELO2), mRNA [NM_016111]	**1.015**
LOC494150	Homo sapiens prohibitin pseudogene, mRNA (cDNA clone IMAGE:4547239). [BC014228]	**1.015**
LOC442308	Homo sapiens similar to tubulin, beta 5 (LOC442308), non-coding RNA [NR_003598]	**1.014**
EHMT1	Homo sapiens euchromatic histone-lysine N-methyltransferase 1 (EHMT1), mRNA [NM_024757]	**1.014**
PAFAH2	Homo sapiens platelet-activating factor acetylhydrolase 2, 40kDa (PAFAH2), mRNA [NM_000437]	**1.012**
BRCA2	Homo sapiens breast cancer 2, early onset (BRCA2), mRNA [NM_000059]	**1.011**
NOL12	Homo sapiens nucleolar protein 12 (NOL12), mRNA [NM_024313]	**1.011**
UBE2NL	Homo sapiens ubiquitin-conjugating enzyme E2N-like (UBE2NL), mRNA [NM_001012989]	**1.011**
TMED3	Homo sapiens transmembrane emp24 protein transport domain containing 3 (TMED3), mRNA [NM_007364]	**1.010**
PLCL1	Homo sapiens phospholipase C-like 1 (PLCL1), mRNA [NM_006226]	**1.010**
hCG_1988300	full-length cDNA clone CS0DI072YA21 of Placenta Cot 25-normalized of Homo sapiens (human). [CR613736]	**1.009**
PNPLA6	Homo sapiens patatin-like phospholipase domain containing 6 (PNPLA6), mRNA [NM_006702]	**1.009**
COX1	Cytochrome c oxidase subunit 1 (EC 1.9.3.1)(Cytochrome c oxidase polypeptide	**1.008**

	I) [Source:UniProtKB/Swiss-Prot;Acc:P00395] [ENST00000361624]	
FKRP	Homo sapiens fukutin related protein (FKRP), mRNA [NM_001039885]	**1.008**
SRCAP	Homo sapiens mRNA for KIAA0309 gene, partial cds. [AB002307]	**1.008**
SAMD4B	Homo sapiens sterile alpha motif domain containing 4B (SAMD4B), mRNA [NM_018028]	**1.007**
SCMH1	Homo sapiens sex comb on midleg homolog 1 (Drosophila) (SCMH1), mRNA [NM_012236]	**1.007**
NAGPA	Homo sapiens N-acetylglucosamine-1-phosphodiester alpha-N-acetylglucosaminidase (NAGPA), mRNA [NM_016256]	**1.007**
KEAP1	Homo sapiens kelch-like ECH-associated protein 1 (KEAP1),mRNA [NM_203500]	**1.006**
ZNF766	Homo sapiens zinc finger protein 766 (ZNF766), mRNA [NM_001010851]	**1.006**
BMP1	Homo sapiens bone morphogenetic protein 1 (BMP1), mRNA [NM_006128]	**1.006**
CCS	Homo sapiens copper chaperone for superoxide dismutase (CCS), mRNA [NM_005125]	**1.004**
CORO1B	Homo sapiens coronin, actin binding protein, 1B (CORO1B),mRNA [NM_020441]	**1.004**
HERPUD2	Homo sapiens HERPUD family member 2 (HERPUD2), mRNA [NM_022373]	**1.004**
KPTN	Homo sapiens kaptin (actin binding protein) (KPTN), mRNA [NM_007059]	**1.004**
LASS5	Homo sapiens LAG1 homolog, ceramide synthase 5 (LASS5), mRNA [NM_147190]	**1.004**
PDK4	Homo sapiens pyruvate dehydrogenase kinase, isozyme 4 (PDK4), mRNA [NM_002612]	**1.004**
PRPF6	Homo sapiens PRP6 pre-mRNA processing factor 6 homolog (PRPF6), mRNA [NM_012469]	**1.004**
GOLGA2	Homo sapiens golgi autoantigen, golgin subfamily a, 2 (GOLGA2), mRNA [NM_004486]	**1.004**
GSTCD	Homo sapiens glutathione S-transferase, C-terminal domain containing (GSTCD), mRNA [NM_024751]	**1.004**
ZNF146	Homo sapiens zinc finger protein 146 (ZNF146), mRNA [NM_007145]	**1.004**
JOSD1	Homo sapiens Josephin domain containing 1 (JOSD1), mRNA [NM_014876]	**1.004**
KBTBD4	Homo sapiens kelch repeat and BTB (POZ) domain containing 4 (KBTBD4), mRNA [NM_016506]	**1.004**
KIAA0652	Homo sapiens KIAA0652 (KIAA0652), mRNA [NM_014741]	**1.003**
LENG1	Homo sapiens leukocyte receptor cluster (LRC) member 1 (LENG1), mRNA [NM_024316]	**1.002**
ACTR5	Homo sapiens ARP5 actin-related protein 5 homolog (yeast) (ACTR5), mRNA [NM_024855]	**1.001**
KARS	Homo sapiens lysyl-tRNA synthetase (KARS), mRNA [NM_005548]	**1.001**
SEMA4B	Homo sapiens sema domain, immunoglobulin domain, transmembrane domain and short cytoplasmic domain, (semaphorin) 4B (SEMA4B), mRNA [NM_020210]	**1.001**
ZNF513	Homo sapiens zinc finger protein 513 (ZNF513), mRNA [NM_144631]	**1.001**

COASY	Homo sapiens Coenzyme A synthase (COASY), mRNA [NM_025233]	**1.000**
MARCH4	Homo sapiens membrane-associated ring finger (C3HC4) 4 (MARCH4), mRNA [NM_020814]	**1.000**
NFKBIL1	Homo sapiens nuclear factor of kappa light polypeptide gene enhancer in B-cells inhibitor-like 1 (NFKBIL1), mRNA [NM_005007]	**1.000**
