# Supplementary material for: Genotoxicity and molecular response of silver nanoparticle (NP)-based hydrogel
Source: J Nanobiotechnology. 2012 May 1;10:16. doi: 10.1186/1477-3155-10-16 (PMC3430588; doi:10.1186/1477-3155-10-16)
Supplement: Additional file 2 — Down-regulated genes in cells exposed to silver-NP-hydrogel for 24 h. Fold-change is logarithmic ratio (log2 ratio) to expression level in control. [file 1477-3155-10-16-S2.pdf]

Additional File 2. Down-regulated genes in cells exposed to silver-NP-hydrogel for 24 h. Fold-change is logarithmic ratio (log_2 ratio) to expression level in control.

Gene Name	Description	Fold-change (log2 ratio)
EDN2	Homo sapiens endothelin 2 (EDN2), mRNA [NM_001956]	**-3.356**
ITGA11	Homo sapiens integrin, alpha 11 (ITGA11), mRNA [NM_001004439]	**-2.988**
DLC1	Homo sapiens deleted in liver cancer 1 (DLC1), mRNA [NM_182643]	**-2.870**
MGC16121	Homo sapiens hypothetical protein MGC16121, mRNA (cDNA clone IMAGE:3627113), [BC007360]	**-2.590**
C18orf56	Homo sapiens chromosome 18 open reading frame 56 (C18orf56), mRNA [NM_001012716]	**-2.574**
TMSB15A	Homo sapiens thymosin-like 8 (TMSL8), mRNA [NM_021992]	**-2.391**
ACCN2	Homo sapiens amiloride-sensitive cation channel 2, neuronal (ACCN2),mRNA [NM_020039]	**-2.364**
HOXC8	Homo sapiens homeobox C8 (HOXC8), mRNA [NM_022658]	**-2.360**
LFNG	Homo sapiens LFNG O-fucosylpeptide 3-beta-N-acetylglucosaminyltransferase (LFNG), mRNA [NM_001040167]	**-2.339**
SEPW1	Homo sapiens selenoprotein W, 1 (SEPW1), mRNA [NM_003009]	**-2.313**
IL17RB	Homo sapiens interleukin 17 receptor B (IL17RB), mRNA [NM_018725]	**-2.308**
NCRNA00115	Homo sapiens non-protein coding RNA 115 (NCRNA00115), non-coding RNA [NR_024321]	**-2.292**
CLEC14A	Homo sapiens C-type lectin domain family 14, member A (CLEC14A), mRNA [NM_175060]	**-2.284**
ANO3	Homo sapiens anoctamin 3 (ANO3), mRNA [NM_031418]	**-2.265**
AFAP1L2	Homo sapiens actin filament associated protein 1-like 2 (AFAP1L2),mRNA [NM_032550]	**-2.262**
NOX4	Homo sapiens NADPH oxidase 4 (NOX4), mRNA [NM_016931]	**-2.216**
ZBED2	Homo sapiens zinc finger, BED-type containing 2 (ZBED2), mRNA [NM_024508]	**-2.175**
KLRC1	Homo sapiens killer cell lectin-like receptor subfamily C, member 1 (KLRC1), mRNA [NM_007328]	**-2.133**
CCDC85A	Homo sapiens coiled-coil domain containing 85A (CCDC85A), mRNA [NM_001080433]	**-2.125**
C20orf71	Homo sapiens chromosome 20 open reading frame 71 (C20orf71),mRNA [NM_178466]	**-2.114**
ADRA2A	Homo sapiens adrenergic, alpha-2A-, receptor (ADRA2A), mRNA [NM_000681]	**-2.113**
RICH2	Homo sapiens Rho-type GTPase-activating protein RICH2 (RICH2), mRNA [NM_014859]	**-2.103**
RASGRP1	Homo sapiens RAS guanyl releasing protein 1 (calcium and DAG-regulated) (RASGRP1), mRNA [NM_005739]	**-2.079**

LOC100133154	Homo sapiens hypothetical protein LOC100133154, mRNA [XM_001714925]	**-2.075**
E2F8	Homo sapiens E2F , mRNA [NM_024680]	**-2.073**
DOK3	Homo sapiens cDNA FLJ39939 fis, clone SPLEN2022227, mRNA. [AK097258]	**-2.072**
DDIT4L	Homo sapiens DNA-damage-inducible transcript 4-like (DDIT4L),mRNA [NM_145244]	**-2.046**
SMPD3	Homo sapiens sphingomyelin phosphodiesterase 3, mRNA [NM_018667]	**-2.025**
GPR87	Homo sapiens G protein-coupled receptor 87 (GPR87), mRNA [NM_023915]	**-2.009**
COL9A3	Homo sapiens collagen, type IX, alpha 3 (COL9A3), mRNA [NM_001853]	**-1.993**
KRT86	Homo sapiens keratin 86 (KRT86), mRNA [NM_002284]	**-1.993**
FGF12	Homo sapiens fibroblast growth factor 12 (FGF12), mRNA [NM_004113]	**-1.984**
PRKCB	Homo sapiens protein kinase C, beta (PRKCB), mRNA [NM_002738]	**-1.983**
ADAMTS4	Homo sapiens ADAM metallopeptidase with thrombospondin type 1 motif, mRNA [NM_005099]	**-1.980**
CPB1	Homo sapiens carboxypeptidase B1 (tissue) (CPB1), mRNA [NM_001871]	**-1.953**
BEGAIN	Homo sapiens brain-enriched guanylate kinase-associated homolog (rat) (BEGAIN), mRNA [NM_020836]	**-1.952**
IKZF4	Homo sapiens IKAROS family zinc finger 4 (Eos) (IKZF4), mRNA [NM_022465]	**-1.951**
LOC284454	Homo sapiens mRNA; cDNA DKFZp686K181 (from clone DKFZp686K181). [BX640708]	**-1.945**
GPX1	Homo sapiens glutathione peroxidase 1 (GPX1), mRNA [NM_201397]	**-1.940**
DAPL1	Homo sapiens death associated protein-like 1 (DAPL1), mRNA [NM_001017920]	**-1.939**
MFAP4	Homo sapiens microfibrillar-associated protein 4 (MFAP4), mRNA [NM_002404]	**-1.930**
PLCXD3	Homo sapiens phosphatidylinositol-specific phospholipase C,mRNA [NM_001005473]	**-1.918**
LOC100144602	Homo sapiens hypothetical, mRNA (cDNA clone IMAGE:4429392), partial cds. [BC017721]	**-1.904**
AGXT2L1	Homo sapiens alanine-glyoxylate aminotransferase 2-like 1 (AGXT2L1), mRNA [NM_031279]	**-1.884**
LOC100127984	Homo sapiens hypothetical protein LOC100127984 (LOC100127984), mRNA [XM_001719916]	**-1.877**
CHRNB3	Homo sapiens cholinergic receptor, nicotinic, beta 3 (CHRNB3), mRNA [NM_000749]	**-1.876**
ZNF594	Homo sapiens zinc finger protein 594 (ZNF594), mRNA [NM_032530]	**-1.875**
LOC339483	Homo sapiens cDNA FLJ38790 fis, clone LIVER2002842. [AK096109]	**-1.860**
KLRC2	Homo sapiens killer cell lectin-like receptor subfamily C, member 2 (KLRC2), mRNA [NM_002260]	**-1.855**
F13A1	Homo sapiens coagulation factor XIII, A1 polypeptide (F13A1), mRNA [NM_000129]	**-1.845**

C8orf22	Homo sapiens chromosome 8 open reading frame 22 (C8orf22), mRNA [NM_001007176]	**-1.844**
BCL3	Homo sapiens B-cell CLL/lymphoma 3 (BCL3), mRNA [NM_005178]	**-1.843**
MMP12	Homo sapiens matrix metallopeptidase 12 (macrophage elastase) (MMP12), mRNA [NM_002426]	**-1.835**
TNFRSF25	Homo sapiens tumor necrosis factor receptor superfamily, member 25 (TNFRSF25), mRNA [NM_148965]	**-1.829**
ABCA1	Homo sapiens ATP-binding cassette, sub-family A (ABC1), member 1 (ABCA1), mRNA [NM_005502]	**-1.828**
LOC54492	Homo sapiens neuralized-2 (LOC54492), mRNA [NM_001142651]	**-1.828**
C10orf95	Homo sapiens chromosome 10 open reading frame 95 (C10orf95), mRNA [NM_024886]	**-1.813**
SLC29A4	Homo sapiens solute carrier family 29,member 4 (SLC29A4), mRNA [NM_001040661]	**-1.797**
KLRC3	Homo sapiens killer cell lectin-like receptor subfamily C, member 3 (KLRC3), mRNA [NM_007333]	**-1.786**
SAMD12	AV724325 HTB Homo sapiens cDNA clone HTBAYE05 5', mRNA [AV724325]	**-1.784**
LOC388889	Homo sapiens cDNA: FLJ22849 fis, clone KAIA987. [AK026502]	**-1.778**
SPATA9	Homo sapiens cDNA FLJ35906 fis, clone TESTI2009727. [AK093225]	**-1.776**
LOC338799	Homo sapiens hypothetical locus LOC338799, non-coding RNA [NR_002809]	**-1.771**
C22orf27	Homo sapiens cDNA FLJ35801 fis, clone TESTI2005937. [AK093120]	**-1.770**
VGLL3	Homo sapiens vestigial like 3 (Drosophila) (VGLL3), mRNA [NM_016206]	**-1.769**
PHB2	Homo sapiens prohibitin 2 (PHB2), mRNA [NM_007273]	**-1.764**
SPHAR	Homo sapiens S-phase response (cyclin-related) (SPHAR), mRNA [NM_006542]	**-1.759**
PPP1R3C	Homo sapiens protein phosphatase 1, regulatory (inhibitor) subunit 3C (PPP1R3C), mRNA [NM_005398]	**-1.758**
SHOX	Human pseudoautosomal homeodomain-containing protein (PHOG) mRNA, complete cds. [U89331]	**-1.758**
PRRT2	Homo sapiens proline-rich transmembrane protein 2 (PRRT2), mRNA [NM_145239]	**-1.756**
MAPK4	H.sapiens 63 kDa protein kinase related to rat ERK3. [X59727]	**-1.754**
RP11-265F14.2	Homo sapiens elastase 2B (ELA2B), mRNA [NM_015849]	**-1.737**
DLX2	Homo sapiens distal-less homeobox 2 (DLX2), mRNA [NM_004405]	**-1.721**
ADAP2	Homo sapiens ArfGAP with dual PH domains 2 (ADAP2), mRNA [NM_018404]	**-1.711**
LOC791120	Homo sapiens hypothetical LOC791120 (LOC791120), non-coding RNA [NR_015357]	**-1.709**
GAS7	Homo sapiens growth arrest-specific 7 (GAS7), mRNA [NM_201433]	**-1.706**

HS6ST3	Homo sapiens heparan sulfate 6-O-sulfotransferase 3 (HS6ST3), mRNA [NM_153456]	**-1.701**
LOC645726	Homo sapiens misc_RNA (LOC645726), miscRNA [XR_018230]	**-1.694**
ZNF423	Homo sapiens zinc finger protein 423 (ZNF423), mRNA [NM_015069]	**-1.688**
DEFB129	Homo sapiens defensin, beta 129 (DEFB129), mRNA [NM_080831]	**-1.684**
CFHR1	Homo sapiens complement factor H-related 1 (CFHR1), mRNA [NM_002113]	**-1.683**
HPGD	Homo sapiens hydroxyprostaglandin dehydrogenase 15-(NAD) (HPGD), mRNA [NM_000860]	**-1.683**
LOC90499	Homo sapiens mRNA; cDNA DKFZp434H0923 (from clone DKFZp434H0923). [AL137712]	**-1.681**
C8orf4	Homo sapiens chromosome 8 open reading frame 4 (C8orf4), mRNA [NM_020130]	**-1.677**
SNHG10	Homo sapiens small nucleolar RNA host gene 10 (non-protein coding) (SNHG10), non-coding RNA [NR_003138]	**-1.677**
KLHL1	Homo sapiens kelch-like 1 (Drosophila) (KLHL1), mRNA [NM_020866]	**-1.675**
CYP2A13	Homo sapiens cytochrome P450, family 2, polypeptide 13 (CYP2A13), mRNA [NM_000766]	**-1.672**
LOC100129104	Full-length cDNA clone CS0DI026YO05 of Placenta Cot 25-normalized of Homo sapiens (human). [CR610759]	**-1.670**
PRDM12	Homo sapiens PR domain containing 12 (PRDM12), mRNA [NM_021619]	**-1.670**
FAM187B	Homo sapiens family with sequence similarity 187,(FAM187B), mRNA [NM_152481]	**-1.665**
MDGA1	Homo sapiens MAM domain containing glycosylphosphatidylinositol anchor 1 (MDGA1), mRNA [NM_153487]	**-1.665**
SLC4A11	Homo sapiens solute carrier family 4,member 11 (SLC4A11), mRNA [NM_032034]	**-1.657**
LXN	Homo sapiens latexin (LXN), mRNA [NM_020169]	**-1.654**
USP11	Homo sapiens ubiquitin specific peptidase 11 (USP11), mRNA [NM_004651]	**-1.648**
PROC	Homo sapiens protein C (PROC), mRNA [NM_000312]	**-1.645**
TEK	Homo sapiens TEK tyrosine kinase, endothelial (TEK), mRNA [NM_000459]	**-1.642**
CNN1	Homo sapiens calponin 1, basic, smooth muscle (CNN1), mRNA [NM_001299]	**-1.640**
CFLP1	Homo sapiens cofilin pseudogene 1, mRNA (cDNA clone IMAGE:5168640). [BC031631]	**-1.638**
OSR2	Homo sapiens odd-skipped related 2 (Drosophila) (OSR2),mRNA [NM_053001]	**-1.638**
EED	Homo sapiens embryonic ectoderm development (EED), mRNA [NM_152991]	**-1.637**
MXRA8	Homo sapiens matrix-remodelling associated 8 (MXRA8), mRNA [NM_032348]	**-1.632**
HSPB2	Homo sapiens heat shock 27kDa protein 2 (HSPB2), mRNA [NM_001541]	**-1.631**
C1orf133	Homo sapiens chromosome 1 open reading frame 133 (C1orf133), non-coding RNA [NR_024337]	**-1.630**
MYO5C	Homo sapiens myosin VC (MYO5C), mRNA [NM_018728]	**-1.630**
ZFHX2	Homo sapiens mRNA for KIAA1762 protein, [AB051549]	**-1.630**

MTA2	Homo sapiens metastasis associated 1 family, member 2 (MTA2), mRNA [NM_004739]	**-1.629**
C6orf206	Homo sapiens chromosome 6 open reading frame 206 (C6orf206), mRNA [NM_152732]	**-1.628**
C5orf4	Homo sapiens chromosome 5 open reading frame 4 (C5orf4),mRNA [NM_032385]	**-1.625**
ATP6V1G2	Homo sapiens ATPase, H+ transporting,V1 subunit G2 (ATP6V1G2), mRNA [NM_130463]	**-1.623**
SCHIP1	Homo sapiens schwannomin interacting protein 1 (SCHIP1),mRNA [NM_014575]	**-1.621**
LOC645676	Homo sapiens cDNA FLJ44595 fis, clone BLADE2004849. [AK126559]	**-1.620**
TFPI2	Homo sapiens tissue factor pathway inhibitor 2 (TFPI2), mRNA [NM_006528]	**-1.619**
ODAM	Homo sapiens odontogenic, ameloblast asssociated (ODAM),mRNA [NM_017855]	**-1.619**
LOC728537	Homo sapiens cDNA clone IMAGE:5271446. [BC039374]	**-1.617**
FLJ40142	Homo sapiens FLJ40142 protein (FLJ40142), mRNA [NM_207435]	**-1.611**
TROAP	Homo sapiens trophinin associated protein (tastin) (TROAP),mRNA [NM_005480]	**-1.610**
BTBD16	Homo sapiens BTB (POZ) domain containing 16 (BTBD16), mRNA [NM_144587]	**-1.608**
PDGFA	Homo sapiens platelet-derived growth factor alpha polypeptide (PDGFA), mRNA [NM_002607]	**-1.605**
GOLGA8E	Homo sapiens golgi autoantigen, golgin subfamily a, 8E (GOLGA8E), mRNA [NM_001012423]	**-1.600**
ZNF521	Homo sapiens zinc finger protein 521 (ZNF521), mRNA [NM_015461]	**-1.593**
PRG4	Homo sapiens proteoglycan 4 (PRG4), mRNA [NM_005807]	**-1.592**
ZAN	Homo sapiens zonadhesin (ZAN), mRNA [NM_173059]	**-1.590**
TMEM130	Homo sapiens transmembrane protein 130 (TMEM130), mRNA [NM_152913]	**-1.583**
LOC100132439	Homo sapiens similar to Protein FAM27E3 (LOC100132439), mRNA [XM_001719283]	**-1.575**
OR7E47P	Homo sapiens olfactory receptor, family 7,mRNA (cDNA clone IMAGE:5590288). [BC042060]	**-1.574**
EFHC1	Homo sapiens EF-hand domain (C-terminal) containing 1 (EFHC1), mRNA [NM_018100]	**-1.573**
LOC100133616	Putative uncharacterized protein FLJ35883 [Source:UniProtKB/Swiss-Prot; Acc:Q8NA34] [ENST00000357301]	**-1.572**
LOC340508	Homo sapiens hypothetical protein LOC340508 (LOC340508), non-coding RNA [NR_002942]	**-1.572**
TREML2	Homo sapiens triggering receptor expressed on myeloid cells-like 2 (TREML2), mRNA [NM_024807]	**-1.568**

TTBK1	Homo sapiens cDNA FLJ16105 fis, clone TESTI2025924, weakly similar to CASEIN KINASE I, EPSILON ISOFORM (EC 2.7.1.-). [AK131217]	**-1.568**
GABRE	Homo sapiens gamma-aminobutyric acid (GABA) A receptor, epsilon (GABRE), mRNA [NM_004961]	**-1.567**
FGFR3	Homo sapiens fibroblast growth factor receptor 3 (FGFR3),mRNA [NM_000142]	**-1.565**
KAL1	Homo sapiens Kallmann syndrome 1 sequence (KAL1), mRNA [NM_000216]	**-1.562**
LOC644450	Homo sapiens hypothetical protein LOC644450, mRNA (cDNA clone IMAGE:4606942), [BC022881]	**-1.560**
MGC3207	Homo sapiens translation initiation factor eIF-2B subunit alpha/beta/delta-like protein (MGC3207), mRNA [NM_001031727]	**-1.560**
PRRX2	Homo sapiens paired related homeobox 2 (PRRX2), mRNA [NM_016307]	**-1.560**
KLRC4	Homo sapiens killer cell lectin-like receptor subfamily C, member 4 (KLRC4), mRNA [NM_013431]	**-1.559**
HOXA13	Homo sapiens homeobox A13 (HOXA13), mRNA [NM_000522]	**-1.557**
SVOPL	Homo sapiens SVOP-like (SVOPL), mRNA [NM_174959]	**-1.556**
LMO1	Homo sapiens LIM domain only 1 (rhombotin 1) (LMO1), mRNA [NM_002315]	**-1.554**
SLC45A3	Homo sapiens solute carrier family 45, member 3 (SLC45A3), mRNA [NM_033102]	**-1.553**
MCM7	Homo sapiens minichromosome maintenance complex component 7 (MCM7), mRNA [NM_182776]	**-1.552**
REEP2	Homo sapiens receptor accessory protein 2 (REEP2), mRNA [NM_016606]	**-1.551**
NDE1	Homo sapiens nudE nuclear distribution gene E homolog 1 (A. nidulans) (NDE1), mRNA [NM_017668]	**-1.548**
LOC338651	Homo sapiens hypothetical protein LOC338651 (LOC338651), non-coding RNA [NR_021489]	**-1.546**
DLX4	Homo sapiens distal-less homeobox 4 (DLX4), mRNA [NM_138281]	**-1.543**
TMEM45B	Homo sapiens transmembrane protein 45B (TMEM45B), mRNA [NM_138788]	**-1.536**
KIAA1267	Homo sapiens KIAA1267 (KIAA1267), mRNA [NM_015443]	**-1.535**
B3GALT4	Homo sapiens UDP-Gal:betaGlcNAc beta 1,3-galactosyltransferase, polypeptide 4 (B3GALT4), mRNA [NM_003782]	**-1.534**
LOC392335	Homo sapiens misc_RNA (LOC392335),miscRNA [XR_037043]	**-1.534**
C10orf140	Homo sapiens chromosome 10 open reading frame 140 (C10orf140), mRNA [NM_207371]	**-1.532**
PGM5	Homo sapiens phosphoglucomutase 5 (PGM5), mRNA [NM_021965]	**-1.532**
RASD2	Homo sapiens RASD family, member 2 (RASD2), mRNA [NM_014310]	**-1.531**
ID2	Homo sapiens inhibitor of DNA binding 2, dominant negative helix-loop-helix protein (ID2), mRNA [NM_002166]	**-1.529**
HOXC9	Homo sapiens homeobox C9 (HOXC9), mRNA [NM_006897]	**-1.528**

NPTX1	Homo sapiens neuronal pentraxin I (NPTX1), mRNA [NM_002522]	**-1.528**
LRP4	Homo sapiens low density lipoprotein receptor-related protein 4 (LRP4), mRNA [NM_002334]	**-1.526**
UCN2	Homo sapiens urocortin 2 (UCN2), mRNA [NM_033199]	**-1.525**
MRPL16	Homo sapiens mitochondrial ribosomal protein L16 (MRPL16),mRNA [NM_017840]	**-1.522**
STARD8	Homo sapiens StAR-related lipid transfer (START) domain containing 8 (STARD8), mRNA [NM_014725]	**-1.522**
CTSK	Homo sapiens cathepsin K (CTSK), mRNA [NM_000396]	**-1.521**
MYLK2	Homo sapiens myosin light chain kinase 2 (MYLK2), mRNA [NM_033118]	**-1.519**
C17orf39	Homo sapiens chromosome 17 open reading frame 39 (C17orf39), mRNA [NM_024052]	**-1.517**
NANOS1	Homo sapiens nanos homolog 1 (Drosophila) (NANOS1), mRNA [NM_199461]	**-1.515**
THBD	Homo sapiens thrombomodulin (THBD), mRNA [NM_000361]	**-1.509**
NEBL	Homo sapiens nebulette (NEBL), mRNA [NM_006393]	**-1.505**
GKAP1	Homo sapiens G kinase anchoring protein 1 (GKAP1), mRNA [NM_025211]	**-1.504**
COL15A1	Homo sapiens collagen, type XV, alpha 1 (COL15A1), mRNA [NM_001855]	**-1.503**
ZNF167	Homo sapiens zinc finger protein 167 (ZNF167), mRNA [NM_025169]	**-1.500**
FHOD3	Homo sapiens formin homology 2 domain containing 3 (FHOD3), mRNA [NM_025135]	**-1.499**
LRTM2	Homo sapiens leucine-rich repeats and transmembrane domains 2 (LRTM2), mRNA [NM_001039029]	**-1.497**
TMEM72	Homo sapiens transmembrane protein 72 (TMEM72), mRNA [NM_001123376]	**-1.497**
PLAC8	Homo sapiens placenta-specific 8 (PLAC8), mRNA [NM_016619]	**-1.490**
GATM	Homo sapiens glycine amidinotransferase (L-arginine:glycine amidinotransferase) (GATM), mRNA [NM_001482]	**-1.489**
RFX2	Homo sapiens regulatory factor X, 2 (influences HLA class II expression) (RFX2), mRNA [NM_000635]	**-1.489**
KIAA0802	Homo sapiens KIAA0802 (KIAA0802), mRNA [NM_015210]	**-1.486**
CA5A	Homo sapiens carbonic anhydrase VA, mitochondrial (CA5A),mRNA [NM_001739]	**-1.485**
NPY5R	Homo sapiens neuropeptide Y receptor Y5 (NPY5R), mRNA [NM_006174]	**-1.485**
ASB9	Homo sapiens ankyrin repeat and SOCS box-containing 9 (ASB9),mRNA [NM_001031739]	**-1.481**
KRT15	Homo sapiens keratin 15 (KRT15), mRNA [NM_002275]	**-1.479**
LINGO2	Homo sapiens leucine rich repeat and Ig domain containing 2 (LINGO2), mRNA [NM_152570]	**-1.479**
CDC14A	Homo sapiens CDC14 cell division cycle 14 homolog A (S. cerevisiae) (CDC14A), mRNA [NM_003672]	**-1.478**
LOC653071	Homo sapiens similar to CG32820-PA, mRNA (cDNA clone IMAGE:4812880), [BC068588]	**-1.476**
C6orf155	Homo sapiens cDNA FLJ13189 fis, clone NT2RP3004253. [AK023251]	**-1.475**

PDGFRL	Homo sapiens platelet-derived growth factor receptor-like (PDGFRL), mRNA [NM_006207]	**-1.475**
HIVEP3	Homo sapiens human immunodeficiency virus type I enhancer binding protein 3 (HIVEP3), mRNA [NM_024503]	**-1.474**
C2orf15	Homo sapiens chromosome 2 open reading frame 15 (C2orf15), mRNA [NM_144706]	**-1.473**
LOC647500	Homo sapiens misc_RNA (LOC647500), miscRNA [XR_041430]	**-1.471**
GTPBP3	Homo sapiens GTP binding protein 3 (mitochondrial) (GTPBP3),mRNA [NM_133644]	**-1.469**
ZNF671	Homo sapiens zinc finger protein 671 (ZNF671), mRNA [NM_024833]	**-1.469**
SLITRK6	Homo sapiens SLIT and NTRK-like family, member 6 (SLITRK6), mRNA [NM_032229]	**-1.468**
LOC344595	Homo sapiens hypothetical LOC344595, mRNA (cDNA clone IMAGE:5760770). [BC039550]	**-1.467**
GPM6B	Homo sapiens glycoprotein M6B (GPM6B), mRNA [NM_001001996]	**-1.466**
HIG2	Homo sapiens hypoxia-inducible protein 2 (HIG2), mRNA [NM_013332]	**-1.466**
RHBDL3	Homo sapiens rhomboid, veinlet-like 3 (Drosophila) (RHBDL3), mRNA [NM_138328]	**-1.466**
MGC12935	Homo sapiens hypothetical protein MGC12935, mRNA (cDNA clone IMAGE:4309284), [BC004565]	**-1.464**
FGFBP3	Homo sapiens fibroblast growth factor binding protein 3 (FGFBP3), mRNA [NM_152429]	**-1.463**
FRS3	Homo sapiens fibroblast growth factor receptor substrate 3 (FRS3), mRNA [NM_006653]	**-1.462**
ANG	Homo sapiens angiogenin, ribonuclease, 5 (ANG), mRNA [NM_001145]	**-1.458**
EZH2	Homo sapiens enhancer of zeste homolog 2 (Drosophila) (EZH2),mRNA [NM_004456]	**-1.458**
ADAMTS5	Homo sapiens ADAM metallopeptidase with thrombospondin type 1 motif, 5 (ADAMTS5), mRNA [NM_007038]	**-1.457**
SPINK4	Homo sapiens serine peptidase inhibitor, Kazal type 4 (SPINK4), mRNA [NM_014471]	**-1.454**
TRPA1	Homo sapiens transient receptor potential cation channel, member 1 (TRPA1), mRNA [NM_007332]	**-1.454**
LOC440983	Homo sapiens hypothetical gene supported by BC066916, mRNA (cDNA clone IMAGE:4838452). [BC066916]	**-1.452**
PDZD2	Homo sapiens PDZ domain containing 2 (PDZD2), mRNA [NM_178140]	**-1.449**
KCNS3	Homo sapiens potassium voltage-gated channel, member 3 (KCNS3), mRNA [NM_002252]	**-1.448**
DISP1	Homo sapiens dispatched homolog 1 (Drosophila) (DISP1), mRNA [NM_032890]	**-1.447**
ST7OT1	Homo sapiens ST7 overlapping transcript 1 (non-protein coding) (ST7OT1), non-coding RNA [NR_002330]	**-1.441**

FPGS	Homo sapiens folylpolyglutamate synthase (FPGS), mRNA [NM_004957]	**-1.437**
TNFAIP8	Homo sapiens tumor necrosis factor, alpha-induced protein 8 (TNFAIP8), mRNA [NM_014350]	**-1.435**
STX1A	Homo sapiens syntaxin 1A (brain) (STX1A), mRNA [NM_004603]	**-1.432**
SSH3	Homo sapiens slingshot homolog 3 (Drosophila) (SSH3), mRNA [NM_017857]	**-1.430**
EPCAM	Homo sapiens epithelial cell adhesion molecule (EPCAM), mRNA [NM_002354]	**-1.426**
DAND5	Homo sapiens DAN domain family, member 5 (DAND5), mRNA [NM_152654]	**-1.425**
CGREF1	Homo sapiens cell growth regulator with EF-hand domain 1 (CGREF1), mRNA [NM_006569]	**-1.419**
NMU	Homo sapiens neuromedin U (NMU), mRNA [NM_006681]	**-1.419**
OIP5	Homo sapiens Opa interacting protein 5 (OIP5), mRNA [NM_007280]	**-1.419**
TMEM49	Homo sapiens transmembrane protein 49 (TMEM49), mRNA [NM_030938]	**-1.419**
DCN	Homo sapiens decorin (DCN), mRNA [NM_001920]	**-1.418**
GLIPR2	Homo sapiens GLI pathogenesis-related 2 (GLIPR2), mRNA [NM_022343]	**-1.418**
MYB	Homo sapiens v-myb myeloblastosis viral oncogene homolog (avian) (MYB), mRNA [NM_005375]	**-1.416**
LOC203510	Homo sapiens similar to hCG1644442 (LOC203510), mRNA [XM_001719132]	**-1.412**
C15orf23	Homo sapiens chromosome 15 open reading frame 23 (C15orf23),mRNA [NM_001142761]	**-1.411**
GRRP1	Homo sapiens glycine/arginine rich protein 1 (GRRP1), mRNA [NM_024869]	**-1.411**
TYMS	Homo sapiens thymidylate synthetase (TYMS), mRNA [NM_001071]	**-1.411**
MPZL2	Homo sapiens myelin protein zero-like 2 (MPZL2), mRNA [NM_144765]	**-1.410**
HIST1H2BD	Homo sapiens histone cluster 1, H2bd (HIST1H2BD), mRNA [NM_021063]	**-1.407**
NR4A3	Homo sapiens nuclear receptor subfamily 4, group A, member 3 (NR4A3), mRNA [NM_173199]	**-1.407**
GCNT1	Homo sapiens glucosaminyl (N-acetyl) transferase 1, core 2 (GCNT1), mRNA [NM_001490]	**-1.404**
ZNF550	Homo sapiens zinc finger protein 550, mRNA (cDNA clone IMAGE:6044705). [BC053858]	**-1.404**
OR7E24	Homo sapiens olfactory receptor, family 7, member 24 (OR7E24), mRNA [NM_001079935]	**-1.402**
SYTL5	Homo sapiens synaptotagmin-like 5 (SYTL5), mRNA [NM_138780]	**-1.402**
C13orf33	Homo sapiens chromosome 13 open reading frame 33 (C13orf33), mRNA [NM_032849]	**-1.400**
IGFBP2	Homo sapiens insulin-like growth factor binding protein 2,(IGFBP2), mRNA [NM_000597]	**-1.400**
APEX2	Homo sapiens APEX nuclease (apurinic/apyrimidinic endonuclease) 2 (APEX2), mRNA [NM_014481]	**-1.398**
LOC344178	Homo sapiens similar to hCG1794703 (LOC344178), mRNA [XM_001721796]	**-1.398**

PL-5283	Homo sapiens PL-5283 protein (PL-5283), mRNA [NM_001130929]	**-1.398**
BEND5	Homo sapiens BEN domain containing 5 (BEND5), mRNA [NM_024603]	**-1.397**
CCIN	Homo sapiens calicin (CCIN), mRNA [NM_005893]	**-1.396**
LOC100128519	Homo sapiens misc_RNA (LOC100128519), miscRNA [XR_038473]	**-1.396**
RRM1	Homo sapiens ribonucleotide reductase M1 (RRM1), mRNA [NM_001033]	**-1.395**
GUCY2G	Homo sapiens guanylate cyclase 2G homolog (mouse) pseudogene (GUCY2G), mRNA [XM_001718434]	**-1.394**
IGFL2	Homo sapiens IGF-like family member 2 (IGFL2), mRNA [NM_001002915]	**-1.393**
WFDC11	Homo sapiens WAP four-disulfide core domain 11 (WFDC11), mRNA [NM_147197]	**-1.393**
PABPC4L	Homo sapiens poly(A) binding protein, cytoplasmic 4-like (PABPC4L), mRNA [NM_001114734]	**-1.391**
PROCA1	Homo sapiens proline-rich cyclin A1-interacting protein (PROCA1), mRNA [NM_152465]	**-1.391**
VIL1	Homo sapiens villin 1 (VIL1), mRNA [NM_007127]	**-1.391**
ASCL1	Homo sapiens achaete-scute complex homolog 1 (Drosophila) (ASCL1), mRNA [NM_004316]	**-1.388**
C17orf63	Homo sapiens chromosome 17 open reading frame 63 (C17orf63),mRNA [NM_018182]	**-1.388**
MICAL2	Homo sapiens microtubule associated monoxygenase, calponin and LIM domain containing 2 (MICAL2), mRNA [NM_014632]	**-1.387**
DENND2D	0	**-1.387**
LGR6	Homo sapiens leucine-rich repeat-containing G protein-coupled receptor 6 (LGR6), mRNA [NM_001017403]	**-1.386**
MEX3A	Homo sapiens mex-3 homolog A (C. elegans) (MEX3A), mRNA [NM_001093725]	**-1.383**
LOC731724	Homo sapiens hypothetical LOC731724 (LOC731724), mRNA [XM_001726528]	**-1.379**
AGBL1	Homo sapiens cDNA FLJ32310 fis, clone PROST2003102. [AK056872]	**-1.375**
LOC199800	Homo sapiens hypothetical protein LOC199800 (LOC199800), mRNA [NM_001101340]	**-1.374**
NECAB1	Homo sapiens N-terminal EF-hand calcium binding protein 1 (NECAB1), mRNA [NM_022351]	**-1.373**
PLEKHG5	Homo sapiens pleckstrin homology domain containing, family G member 5 (PLEKHG5), mRNA [NM_198681]	**-1.368**
FAM83D	Homo sapiens family with sequence similarity 83, member D (FAM83D), mRNA [NM_030919]	**-1.366**
ZNF177	Homo sapiens zinc finger protein 177 (ZNF177), mRNA [NM_003451]	**-1.365**
SLCO1C1	Homo sapiens solute carrier organic anion transporter family, member 1C1 (SLCO1C1), mRNA [NM_017435]	**-1.362**
STAC2	Homo sapiens SH3 and cysteine rich domain 2 (STAC2), mRNA [NM_198993]	**-1.361**

MED4	Homo sapiens mediator complex subunit 4 (MED4), mRNA [NM_014166]	**-1.360**
NR4A1	Homo sapiens nuclear receptor subfamily 4, group A, member 1 (NR4A1), mRNA [NM_002135]	**-1.360**
IL23A	Homo sapiens interleukin 23, alpha subunit p19 (IL23A), mRNA [NM_016584]	**-1.358**
ANKRD43	Homo sapiens ankyrin repeat domain 43 (ANKRD43), mRNA [NM_175873]	**-1.357**
TMEM25	Homo sapiens transmembrane protein 25 (TMEM25), mRNA [NM_032780]	**-1.357**
MADCAM1	Homo sapiens mucosal vascular addressin cell adhesion molecule 1 (MADCAM1), mRNA [NM_130760]	**-1.356**
ZNF692	Homo sapiens zinc finger protein 692 (ZNF692), mRNA [NM_017865]	**-1.352**
RUFY1	Homo sapiens RUN and FYVE domain containing 1 (RUFY1),mRNA [NM_025158]	**-1.351**
TRAF5	Homo sapiens TNF receptor-associated factor 5 (TRAF5), mRNA [NM_004619]	**-1.346**
SPNS2	Homo sapiens spinster homolog 2 (Drosophila) (SPNS2), mRNA [NM_001124758]	**-1.342**
LOC285535	Homo sapiens cDNA FLJ33738 fis, clone BRAWH2018527. [AK091057]	**-1.340**
KIAA0774	Homo sapiens KIAA0774 (KIAA0774), mRNA [NM_001033602]	**-1.339**
GNGT1	Homo sapiens guanine nucleotide binding protein (G protein), (GNGT1), mRNA [NM_021955]	**-1.339**
AMY1C	Homo sapiens amylase, alpha 1C (salivary) (AMY1C),mRNA [NM_001008219]	**-1.338**
RAVER2	Homo sapiens cDNA FLJ10770 fis, clone NT2RP4000159. [AK001632]	**-1.338**
TBC1D24	Homo sapiens TBC1 domain family, member 24 (TBC1D24), mRNA [NM_020705]	**-1.338**
KANK4	Homo sapiens KN motif and ankyrin repeat domains 4 (KANK4), mRNA [NM_181712]	**-1.336**
ALG8	Homo sapiens asparagine-linked glycosylation 8, alpha-1,3-glucosyltransferase homolog (ALG8), mRNA [NM_001007027]	**-1.335**
CDCA8	Homo sapiens cell division cycle associated 8 (CDCA8), mRNA [NM_018101]	**-1.334**
GRK5	Homo sapiens G protein-coupled receptor kinase 5 (GRK5), mRNA [NM_005308]	**-1.328**
C11orf86	Homo sapiens chromosome 11 open reading frame 86 (C11orf86), mRNA [NM_001136485]	**-1.326**
E2F3	Homo sapiens E2F transcription factor 3 (E2F3), mRNA [NM_001949]	**-1.325**
ACADL	Homo sapiens acyl-Coenzyme A dehydrogenase, long chain (ACADL),mRNA [NM_001608]	**-1.324**
HDC	Homo sapiens histidine decarboxylase (HDC), mRNA [NM_002112]	**-1.324**
PAQR8	Homo sapiens progestin and adipoQ receptor family member VIII (PAQR8), mRNA [NM_133367]	**-1.324**
LOC644246	Homo sapiens hypothetical protein LOC644246, mRNA (cDNA clone IMAGE:4730995), partial cds. [BC020847]	**-1.323**
CDH10	Homo sapiens cadherin 10, type 2 (T2-cadherin) (CDH10), mRNA	**-1.322**

	[NM_006727]	
TBX19	Homo sapiens T-box 19 (TBX19), mRNA [NM_005149]	**-1.322**
MYLK	Homo sapiens myosin light chain kinase (MYLK), mRNA [NM_053025]	**-1.322**
C9orf68	Homo sapiens chromosome 9 open reading frame 68 (C9orf68), mRNA [NM_001039395]	**-1.321**
LUZP2	Homo sapiens leucine zipper protein 2 (LUZP2), mRNA [NM_001009909]	**-1.319**
ZSCAN1	Homo sapiens zinc finger and SCAN domain containing 1 (ZSCAN1), mRNA [NM_182572]	**-1.319**
CDKN2C	Homo sapiens cyclin-dependent kinase inhibitor 2C (p18, inhibits CDK4) (CDKN2C), mRNA [NM_078626]	**-1.316**
KLHL29	Homo sapiens cDNA FLJ37281 fis, clone BRAMY2013200. [AK094600]	**-1.316**
ANKRD23	Homo sapiens ankyrin repeat domain 23 (ANKRD23), mRNA [NM_144994]	**-1.315**
LOC729983	PREDICTED: Homo sapiens hypothetical LOC729983 (LOC729983), mRNA [XM_001718065]	**-1.313**
WNT4	Homo sapiens wingless-type MMTV integration site family, member 4 (WNT4), mRNA [NM_030761]	**-1.313**
FAM179A	Homo sapiens family with sequence similarity 179, member A (FAM179A), mRNA [NM_199280]	**-1.311**
ZFHX3	Homo sapiens zinc finger homeobox 3 (ZFHX3), mRNA [NM_006885]	**-1.311**
MYOM2	Homo sapiens myomesin (M-protein) 2, (MYOM2), mRNA [NM_003970]	**-1.309**
LOC100128822	Homo sapiens cDNA clone IMAGE:5286843. [BC036622]	**-1.308**
LOC728705	Homo sapiens cDNA FLJ31150 fis, clone IMR322001534. [AK055712]	**-1.308**
TAF1C	Homo sapiens TATA box binding protein (TBP)-associated factor, C(TAF1C), mRNA [NM_005679]	**-1.306**
LOC730834	DB090170 TESTI4 Homo sapiens cDNA clone TESTI4038997 5', mRNA sequence [DB090170]	**-1.305**
HHIP	Homo sapiens hedgehog interacting protein (HHIP), mRNA [NM_022475]	**-1.303**
PPEF1	Homo sapiens protein phosphatase, EF-hand calcium binding domain 1 (PPEF1), mRNA [NM_006240]	**-1.303**
DNM3	Homo sapiens dynamin 3 (DNM3), mRNA [NM_015569]	**-1.302**
ZNF710	Homo sapiens mRNA for FLJ00306 protein. [AK160373]	**-1.301**
IGFBP5	Homo sapiens insulin-like growth factor binding protein 5 (IGFBP5), mRNA [NM_000599]	**-1.301**
CDSN	Homo sapiens corneodesmosin (CDSN), mRNA [NM_001264]	**-1.300**
C13orf34	Homo sapiens chromosome 13 open reading frame 34 (C13orf34), mRNA [NM_024808]	**-1.299**
DND1	Homo sapiens dead end homolog 1 (zebrafish) (DND1), mRNA [NM_194249]	**-1.295**
RLTPR	Homo sapiens mRNA for FLJ00330 protein. [AK090421]	**-1.295**
C2CD3	Homo sapiens C2 calcium-dependent domain containing 3 (C2CD3), mRNA [NM_015531]	**-1.292**

CIT	Homo sapiens citron (rho-interacting, serine/threonine kinase 21) (CIT), mRNA [NM_007174]	**-1.291**
DKFZp686E2433	Homo sapiens similar to hypothetical protein 9630041N07 (DKFZp686E2433), mRNA [NM_001136116]	**-1.287**
SNHG12	Homo sapiens small nucleolar RNA host gene 12 (non-protein coding) (SNHG12), non-coding RNA [NR_024127]	**-1.286**
FLJ27365	Homo sapiens cDNA FLJ13362 fis, clone PLACE1000236. [AK023424]	**-1.283**
AIG1	Homo sapiens androgen-induced 1 (AIG1), mRNA [NM_016108]	**-1.282**
FBN1	Homo sapiens fibrillin 1 (FBN1), mRNA [NM_000138]	**-1.282**
C4orf19	Homo sapiens chromosome 4 open reading frame 19 (C4orf19),mRNA [NM_018302]	**-1.282**
C9orf103	Homo sapiens chromosome 9 open reading frame 103 (C9orf103), mRNA [NM_001001551]	**-1.280**
FZD10	Homo sapiens frizzled homolog 10 (Drosophila) (FZD10),mRNA [NM_007197]	**-1.279**
HSPA12A	Homo sapiens heat shock protein 12A (HSPA12A),mRNA [NM_025015]	**-1.277**
RAD52	Homo sapiens RAD52 homolog (RAD52), mRNA [NM_134424]	**-1.273**
FAM64A	Homo sapiens family with sequence similarity 64, member A (FAM64A), mRNA [NM_019013]	**-1.272**
LHX2	Homo sapiens LIM homeobox 2 (LHX2), mRNA [NM_004789]	**-1.272**
CECR1	Homo sapiens cat eye syndrome chromosome region, candidate 1 (CECR1), mRNA [NM_017424]	**-1.270**
KIAA0895L	Homo sapiens cDNA FLJ34984 fis, clone OCBBF2001639. [AK092303]	**-1.270**
TSPAN2	Homo sapiens tetraspanin 2 (TSPAN2), mRNA [NM_005725]	**-1.269**
TCP10L	Homo sapiens t-complex 10 (mouse)-like (TCP10L), mRNA [NM_144659]	**-1.268**
SLC10A4	Homo sapiens solute carrier family 10, member 4 (SLC10A4), mRNA [NM_152679]	**-1.267**
C2orf60	Homo sapiens chromosome 2 open reading frame 60 (C2orf60),mRNA [NM_001039693]	**-1.263**
C5orf45	Homo sapiens chromosome 5 open reading frame 45 (C5orf45), mRNA [NM_016175]	**-1.261**
SEMA3D	Homo sapiens sema domain, immunoglobulin domain,(semaphorin) 3D (SEMA3D), mRNA [NM_152754]	**-1.261**
TLE6	Homo sapiens transducin-like enhancer of split 6 (E(sp1) homolog, Drosophila), mRNA (cDNA clone IMAGE:3687767). [BC007329]	**-1.261**
IQCH	Homo sapiens IQ motif containing H (IQCH), mRNA [NM_022784]	**-1.260**
ZNF48	Zinc finger protein 48 (Zinc finger protein 553) [Source:UniProtKB/Swiss-Prot; Acc:Q96MX3] [ENST00000320159]	**-1.259**
BACE1	Homo sapiens beta-site APP-cleaving enzyme 1 (BACE1),mRNA [NM_012104]	**-1.256**
ELP2P	Homo sapiens endozepine-like peptide 2 pseudogene (ELP2P), non-coding RNA [NR_024120]	**-1.255**
COX4I2	Homo sapiens cytochrome c oxidase subunit IV isoform 2 (lung) (COX4I2), mRNA [NM_032609]	**-1.253**

F8A1	Homo sapiens coagulation factor VIII-associated (intronic transcript) 1 (F8A1), mRNA [NM_012151]	**-1.253**
GGN	Homo sapiens gametogenetin (GGN), mRNA [NM_152657]	**-1.253**
CASP9	Homo sapiens caspase 9, apoptosis-related cysteine peptidase (CASP9), mRNA [NM_001229]	**-1.252**
FLJ30430	Homo sapiens cDNA FLJ30430 fis, clone BRACE2008960. [AK054992]	**-1.251**
LEPREL1	Homo sapiens leprecan-like 1 (LEPREL1), mRNA [NM_018192]	**-1.251**
SULF2	Homo sapiens sulfatase 2 (SULF2), mRNA [NM_018837]	**-1.251**
CCDC74B	Homo sapiens coiled-coil domain containing 74B (CCDC74B), mRNA [NM_207310]	**-1.250**
LEAP2	Homo sapiens liver expressed antimicrobial peptide 2 (LEAP2), mRNA [NM_052971]	**-1.250**
SMARCD3	Homo sapiens SWI/SNF related, matrix associated, member 3 (SMARCD3), mRNA [NM_003078]	**-1.250**
SUZ12P	Full-length cDNA clone CS0DC012YL18 of Neuroblastoma Cot 25-normalized of Homo sapiens (human). [CR597846]	**-1.250**
BDKRB2	Homo sapiens bradykinin receptor B2 (BDKRB2), mRNA [NM_000623]	**-1.249**
EID2	Homo sapiens EP300 interacting inhibitor of differentiation 2 (EID2), mRNA [NM_153232]	**-1.248**
ITGB1BP2	Homo sapiens integrin beta 1 binding protein (melusin) 2 (ITGB1BP2), mRNA [NM_012278]	**-1.248**
PIN4	Homo sapiens protein (peptidylprolyl cis/trans isomerase) NIMA-interacting, 4 (parvulin) (PIN4), mRNA [NM_006223]	**-1.248**
GCSH	Homo sapiens glycine cleavage system protein H (aminomethyl carrier) (GCSH), mRNA [NM_004483]	**-1.247**
SLC25A21	Homo sapiens solute carrier family 25, member 21 (SLC25A21), mRNA [NM_030631]	**-1.247**
EDN1	Homo sapiens endothelin 1 (EDN1), mRNA [NM_001955]	**-1.246**
PRCP	Homo sapiens prolylcarboxypeptidase (angiotensinase C) (PRCP),mRNA [NM_199418]	**-1.246**
VAV3	Homo sapiens vav 3 guanine nucleotide exchange factor (VAV3),mRNA [NM_006113]	**-1.246**
OR7E13P	Homo sapiens olfactory-like receptor PJCG2 (PJCG2) mRNA, [AF238487]	**-1.245**
CNFN	Homo sapiens cornifelin (CNFN), mRNA [NM_032488]	**-1.243**
LOC100131721	Homo sapiens hypothetical protein LOC100131721, mRNA [XM_001716076]	**-1.243**
CHAF1B	Homo sapiens chromatin assembly factor 1, subunit B (p60) (CHAF1B), mRNA [NM_005441]	**-1.242**
SV2B	Homo sapiens synaptic vesicle glycoprotein 2B (SV2B), mRNA [NM_014848]	**-1.242**
LOC644656	Homo sapiens cDNA clone IMAGE:5286302. [BC036621]	**-1.241**
CDC42EP3	Homo sapiens CDC42 effector protein (Rho GTPase binding) 3 (CDC42EP3), mRNA [NM_006449]	**-1.240**

TCOF1	Homo sapiens Treacher Collins-Franceschetti syndrome 1 (TCOF1),mRNA [NM_001008656]	**-1.240**
HMGB2	Homo sapiens high-mobility group box 2 (HMGB2), mRNA [NM_002129]	**-1.239**
TRPV3	Homo sapiens transient receptor potential cation channel, subfamily V, member 3 (TRPV3), mRNA [NM_145068]	**-1.239**
S100A2	Homo sapiens S100 calcium binding protein A2 (S100A2), mRNA [NM_005978]	**-1.237**
FAM100B	Homo sapiens family with sequence similarity 100, member B (FAM100B), mRNA [NM_182565]	**-1.236**
GFOD1	Homo sapiens glucose-fructose oxidoreductase domain containing 1 (GFOD1), mRNA [NM_018988]	**-1.236**
TGM1	Homo sapiens transglutaminase 1 (K polypeptide epidermal type I, protein-glutamine-gamma-glutamyltransferase) (TGM1), mRNA [NM_000359]	**-1.236**
LGALS7B	Homo sapiens lectin, galactoside-binding, soluble, 7B (LGALS7B), mRNA [NM_001042507]	**-1.235**
NET1	Homo sapiens neuroepithelial cell transforming 1 (NET1),mRNA [NM_001047160]	**-1.235**
FGF18	Homo sapiens fibroblast growth factor 18 (FGF18), mRNA [NM_003862]	**-1.233**
PBX4	Homo sapiens pre-B-cell leukemia homeobox 4 (PBX4), mRNA [NM_025245]	**-1.233**
CNR1	Homo sapiens cannabinoid receptor 1 (brain) (CNR1), mRNA [NM_033181]	**-1.231**
LOC202451	Homo sapiens, clone IMAGE:2899977, mRNA [BC022980]	**-1.231**
C1orf56	Homo sapiens chromosome 1 open reading frame 56 (C1orf56), mRNA [NM_017860]	**-1.231**
NKX3-1	Homo sapiens NK3 homeobox 1 (NKX3-1), mRNA [NM_006167]	**-1.229**
MST1	Homo sapiens macrophage stimulating 1 (hepatocyte growth factor-like), mRNA (cDNA clone IMAGE:4821945), [BC044862]	**-1.229**
PRR12	Homo sapiens proline rich 12 (PRR12), mRNA [NM_020719]	**-1.228**
LOC338620	Homo sapiens hypothetical protein LOC338620, mRNA (cDNA clone IMAGE:6023208), [BC043009]	**-1.227**
GNG11	Homo sapiens guanine nucleotide binding protein (G protein), gamma 11 (GNG11), mRNA [NM_004126]	**-1.226**
GRM1	Homo sapiens glutamate receptor, metabotropic 1 (GRM1),mRNA [NM_000838]	**-1.226**
GSK3A	Homo sapiens glycogen synthase kinase 3 alpha (GSK3A), mRNA [NM_019884]	**-1.225**
KLF9	Homo sapiens Kruppel-like factor 9 (KLF9), mRNA [NM_001206]	**-1.225**
DDX53	Homo sapiens DEAD (Asp-Glu-Ala-Asp) box polypeptide 53 (DDX53), mRNA [NM_182699]	**-1.224**
PDE4A	Homo sapiens phosphodiesterase 4A, cAMP-specific (phosphodiesterase E2 dunce homolog, Drosophila) (PDE4A), mRNA [NM_006202]	**-1.224**
A2M	Homo sapiens alpha-2-macroglobulin (A2M), mRNA [NM_000014]	**-1.223**
ETFDH	Homo sapiens electron-transferring-flavoprotein dehydrogenase (ETFDH),	**-1.222**

	mRNA [NM_004453]	
LRRC3B	Homo sapiens leucine rich repeat containing 3B (LRRC3B), mRNA [NM_052953]	**-1.222**
CACNA2D3	Homo sapiens calcium channel, voltage-dependent, alpha 2/delta subunit 3 (CACNA2D3), mRNA [NM_018398]	**-1.222**
HSD11B2	Homo sapiens hydroxysteroid (11-beta) dehydrogenase 2 (HSD11B2), mRNA [NM_000196]	**-1.219**
LOC400960	Homo sapiens cDNA FLJ32260 fis, clone PROST1000334. [AK056822]	**-1.218**
C17orf55	Homo sapiens chromosome 17 open reading frame 55 (C17orf55), mRNA [NM_178519]	**-1.216**
NR4A2	Homo sapiens nuclear receptor subfamily 4, group A, member 2 (NR4A2), mRNA [NM_006186]	**-1.216**
RDM1	Homo sapiens RAD52 motif 1 (RDM1), mRNA [NM_001034836]	**-1.216**
LOC100127980	Homo sapiens cDNA FLJ33508 fis, clone BRAMY2005094. [AK090827]	**-1.215**
BAMBI	Homo sapiens BMP and activin membrane-bound inhibitor homolog (Xenopus laevis) (BAMBI), mRNA [NM_012342]	**-1.214**
LOC388630	Homo sapiens hypothetical LOC388630 (LOC388630), mRNA [XM_371250]	**-1.213**
FLJ10357	Homo sapiens hypothetical protein FLJ10357 (FLJ10357), mRNA [NM_018071]	**-1.212**
CUZD1	Homo sapiens CUB and zona pellucida-like domains 1 (CUZD1), mRNA [NM_022034]	**-1.210**
NF-E4	Homo sapiens, clone IMAGE:5404753, mRNA. [BC036938]	**-1.210**
hCG_1815491	Homo sapiens PNAS-108 mRNA, partial sequence. [AF275804]	**-1.208**
LOC100134331	Immunoglobulin heavy chain V gene segment [Source:IMGT/GENE-DB; Acc:IGHV4-61] [ENST00000390630]	**-1.208**
VPREB3	Homo sapiens pre-B lymphocyte 3 (VPREB3), mRNA [NM_013378]	**-1.208**
THAP2	Homo sapiens THAP domain containing, (THAP2), mRNA [NM_031435]	**-1.205**
ABCC6	Homo sapiens ATP-binding cassette, sub-family C (CFTR/MRP), member 6 (ABCC6), mRNA [NM_001171]	**-1.203**
TRIM72	Homo sapiens tripartite motif-containing 72 (TRIM72),mRNA [NM_001008274]	**-1.203**
IGLON5	Homo sapiens hCG1651476 (LOC402665), mRNA [NM_001101372]	**-1.201**
PLAT	Homo sapiens plasminogen activator, tissue (PLAT), mRNA [NM_000930]	**-1.200**
MECP2	Homo sapiens methyl CpG binding protein 2 (Rett syndrome) (MECP2), mRNA [NM_004992]	**-1.200**
VWDE	Homo sapiens von Willebrand factor D and EGF domains (VWDE), mRNA [NM_001135924]	**-1.199**
ZNF180	Homo sapiens zinc finger protein 180 (ZNF180), mRNA [NM_013256]	**-1.197**
LOC1001	Homo sapiens similar to hCG2018924 (LOC100134119), mRNA	**-1.196**

34119	[XM_001713939]	
ABCD4	Homo sapiens ATP-binding cassette, sub-family D (ALD), member 4 (ABCD4), mRNA [NM_005050]	**-1.195**
FLJ25694	Homo sapiens cDNA FLJ46084 fis, clone TESTI2006543. [AK127969]	**-1.195**
C10orf32	Homo sapiens chromosome 10 open reading frame 32 (C10orf32),mRNA [NM_144591]	**-1.194**
IL15	Homo sapiens interleukin 15 (IL15), transcript variant 1, mRNA [NM_172174]	**-1.194**
LOC441461	Homo sapiens hypothetical gene supported by BC030123, mRNA (cDNA clone IMAGE:4815474). [BC030123]	**-1.194**
LOXL1	Homo sapiens lysyl oxidase-like 1 (LOXL1), mRNA [NM_005576]	**-1.194**
C3orf26	Homo sapiens chromosome 3 open reading frame 26 (C3orf26), mRNA [NM_032359]	**-1.193**
FA2H	Homo sapiens fatty acid 2-hydroxylase (FA2H), mRNA [NM_024306]	**-1.193**
ZNF200	Homo sapiens zinc finger protein 200 (ZNF200), mRNA [NM_003454]	**-1.193**
ADCY9	Homo sapiens adenylate cyclase 9 (ADCY9), mRNA [NM_001116]	**-1.191**
MKI67	Homo sapiens antigen identified by monoclonal antibody Ki-67 (MKI67), mRNA [NM_002417]	**-1.191**
SALL2	Homo sapiens sal-like 2 (Drosophila) (SALL2), mRNA [NM_005407]	**-1.191**
MTHFD2L	Homo sapiens methylenetetrahydrofolate dehydrogenase (NADP+ dependent) 2-like, mRNA (cDNA clone IMAGE:4794959), [BC037529]	**-1.190**
TXNIP	Homo sapiens thioredoxin interacting protein (TXNIP), mRNA [NM_006472]	**-1.185**
ADAMTS3	Homo sapiens ADAM metallopeptidase with thrombospondin type 1 motif, 3 (ADAMTS3), mRNA [NM_014243]	**-1.184**
KAZALD1	Homo sapiens Kazal-type serine peptidase inhibitor domain 1 (KAZALD1), mRNA [NM_030929]	**-1.184**
LOC100190890	Homo sapiens cDNA FLJ39164 fis, clone OCBBF2002656. [AK096483]	**-1.184**
RAB3C	Homo sapiens RAB3C, member RAS oncogene family (RAB3C), mRNA [NM_138453]	**-1.183**
C18orf1	Homo sapiens chromosome 18 open reading frame 1 (C18orf1),mRNA [NM_181482]	**-1.182**
MGP	Homo sapiens matrix Gla protein (MGP), mRNA [NM_000900]	**-1.182**
KCNAB3	Homo sapiens potassium voltage-gated channel,beta member 3 (KCNAB3), mRNA [NM_004732]	**-1.180**
FAM113B	Homo sapiens family with sequence similarity 113, member B (FAM113B), mRNA [NM_138371]	**-1.179**
LOC441208	Homo sapiens hypothetical gene supported by AK094370 (LOC441208), non-coding RNA [NR_003502]	**-1.179**
SEC31B	Homo sapiens SEC31 homolog B (S. cerevisiae) (SEC31B), mRNA [NM_015490]	**-1.178**
IRF2BP1	Homo sapiens interferon regulatory factor 2 binding protein 1 (IRF2BP1), mRNA [NM_015649]	**-1.175**
SPRYD4	Homo sapiens SPRY domain containing 4 (SPRYD4), mRNA [NM_207344]	**-1.175**

TAF6	Homo sapiens TAF6 RNA polymerase II, TATA box binding protein (TBP)-associated factor, (TAF6), mRNA [NM_005641]	**-1.174**
C1orf198	Homo sapiens chromosome 1 open reading frame 198 (C1orf198), mRNA [NM_032800]	**-1.173**
CCNE1	Homo sapiens cyclin E1 (CCNE1), mRNA [NM_001238]	**-1.173**
LOC100130171	Homo sapiens misc_RNA (LOC100130171), miscRNA [XR_038676]	**-1.173**
DLEU1	Homo sapiens deleted in lymphocytic leukemia 1 (non-protein coding) (DLEU1), non-coding RNA [NR_002605]	**-1.172**
EID2B	Homo sapiens EP300 interacting inhibitor of differentiation 2B (EID2B), mRNA [NM_152361]	**-1.172**
PBK	Homo sapiens PDZ binding kinase (PBK), mRNA [NM_018492]	**-1.172**
SPRY3	Homo sapiens sprouty homolog 3 (Drosophila) (SPRY3), mRNA [NM_005840]	**-1.172**
SUSD1	Homo sapiens sushi domain containing 1 (SUSD1), mRNA [NM_022486]	**-1.172**
KLRG2	Homo sapiens cDNA FLJ44186 fis, clone THYMU2038797, weakly similar to B locus C type Lectin. [AK126174]	**-1.169**
IQCD	Homo sapiens IQ motif containing D (IQCD), mRNA [NM_138451]	**-1.168**
ITM2B	Homo sapiens integral membrane protein 2B (ITM2B), mRNA [NM_021999]	**-1.167**
HIRA	Homo sapiens HIR histone cell cycle regulation defective homolog A (S. cerevisiae) (HIRA), mRNA [NM_003325]	**-1.166**
HIF3A	Homo sapiens hypoxia inducible factor 3, alpha subunit (HIF3A),mRNA [NM_022462]	**-1.165**
PRDM11	Homo sapiens PR domain containing 11 (PRDM11), mRNA [NM_020229]	**-1.165**
RREB1	Homo sapiens ras responsive element binding protein 1 (RREB1), mRNA [NM_001003699]	**-1.164**
FCER1G	Homo sapiens Fc fragment of IgE, high affinity I, receptor for gamma polypeptide (FCER1G), mRNA [NM_004106]	**-1.162**
SPINK5	Homo sapiens serine peptidase inhibitor, Kazal type 5 (SPINK5),mRNA [NM_006846]	**-1.162**
SYT12	Homo sapiens synaptotagmin XII (SYT12), mRNA [NM_177963]	**-1.162**
AR	Homo sapiens androgen receptor (AR), mRNA [NM_000044]	**-1.161**
EFEMP1	Homo sapiens EGF-containing fibulin-like extracellular matrix protein 1 (EFEMP1), mRNA [NM_004105]	**-1.161**
LOC441795	Homo sapiens similar to high-mobility group box 3 (LOC441795), partial mRNA [XM_497547]	**-1.161**
SEC24A	Homo sapiens SEC24 family, member A (S. cerevisiae) (SEC24A), mRNA [NM_021982]	**-1.161**
C1orf162	Homo sapiens chromosome 1 open reading frame 162 (C1orf162), mRNA [NM_174896]	**-1.160**
LOC100137724	Homo sapiens hypothetical LOC100137724, mRNA (cDNA clone IMAGE:3608551),[BC005077]	**-1.160**
TSPYL4	Homo sapiens TSPY-like 4 (TSPYL4), mRNA [NM_021648]	**-1.160**
P11	Homo sapiens 26 serine protease (P11), mRNA [NM_006025]	**-1.159**

SETD6	Homo sapiens SET domain containing 6 (SETD6), mRNA [NM_024860]	**-1.159**
SSPN	Homo sapiens sarcospan (Kras oncogene-associated gene) (SSPN),mRNA [NM_005086]	**-1.159**
SCEL	Homo sapiens sciellin (SCEL), mRNA [NM_144777]	**-1.158**
COMTD1	Homo sapiens catechol-O-methyltransferase domain containing 1 (COMTD1), mRNA [NM_144589]	**-1.155**
APBB2	Homo sapiens amyloid beta (A4) precursor protein-binding, family B, member 2 (APBB2), mRNA [NM_173075]	**-1.154**
KIAA0329	Homo sapiens KIAA0329 (KIAA0329), mRNA [NM_014844]	**-1.154**
OR7E156P	Homo sapiens olfactory receptor, family 7, subfamily E, member 156 pseudogene (OR7E156P), non-coding RNA [NR_002171]	**-1.153**
DNAJC25	Homo sapiens DnaJ (Hsp40) homolog, subfamily C , member 25 (DNAJC25), mRNA [NM_001015882]	**-1.152**
PTGER2	Homo sapiens prostaglandin E receptor 2 (subtype EP2),(PTGER2), mRNA [NM_000956]	**-1.152**
AHSA2	Homo sapiens AHA1, activator of heat shock 90kDa protein ATPase homolog 2 (yeast) (AHSA2), mRNA [NM_152392]	**-1.151**
INSL4	Homo sapiens insulin-like 4 (placenta) (INSL4), mRNA [NM_002195]	**-1.151**
POPDC2	Homo sapiens popeye domain containing 2 (POPDC2), mRNA [NM_022135]	**-1.151**
RFX1	Homo sapiens regulatory factor X, 1 (influences HLA class II expression) (RFX1), mRNA [NM_002918]	**-1.151**
MGC26597	Homo sapiens PIP5K1A pseudogene, mRNA (cDNA clone IMAGE:4828163). [BC028580]	**-1.150**
CTNNBIP1	Homo sapiens catenin, beta interacting protein 1 (CTNNBIP1),mRNA [NM_020248]	**-1.149**
TTC9	Homo sapiens tetratricopeptide repeat domain 9 (TTC9), mRNA [NM_015351]	**-1.149**
ARNTL	Homo sapiens aryl hydrocarbon receptor nuclear translocator-like (ARNTL), mRNA [NM_001030273]	**-1.148**
IGF1	Homo sapiens insulin-like growth factor 1 (somatomedin C) (IGF1),mRNA [NM_000618]	**-1.146**
NEUROG3	Homo sapiens neurogenin 3 (NEUROG3), mRNA [NM_020999]	**-1.146**
PIH1D2	Homo sapiens PIH1 domain containing 2 (PIH1D2), mRNA [NM_138789]	**-1.146**
CLDN11	Homo sapiens full length insert cDNA clone YO11A01. [AF085871]	**-1.145**
KCNMB4	Homo sapiens potassium large conductance calcium-activated channel, subfamily M, beta member 4 (KCNMB4), mRNA [NM_014505]	**-1.145**
SPAG5	Homo sapiens sperm associated antigen 5 (SPAG5), mRNA [NM_006461]	**-1.145**
ZNF567	Homo sapiens zinc finger protein 567 (ZNF567), mRNA [NM_152603]	**-1.145**
C1orf135	Homo sapiens chromosome 1 open reading frame 135 (C1orf135), mRNA [NM_024037]	**-1.144**
ANO8	Homo sapiens anoctamin 8 (ANO8), mRNA [NM_020959]	**-1.143**
GPER	Homo sapiens G protein-coupled estrogen receptor 1 (GPER),mRNA	**-1.143**

	[NM_001039966]	
MICALCL	Homo sapiens MICAL C-terminal like (MICALCL), mRNA [NM_032867]	**-1.143**
SULF1	Homo sapiens sulfatase 1 (SULF1), mRNA [NM_015170]	**-1.142**
CCNB2	Homo sapiens cDNA FLJ13342 fis, clone OVARC1001950. [AK023404]	**-1.142**
GLI3	Homo sapiens GLI-Kruppel family member GLI3 (GLI3), mRNA [NM_000168]	**-1.140**
GNRH1	Homo sapiens gonadotropin-releasing hormone 1 (luteinizing-releasing hormone) (GNRH1), mRNA [NM_000825]	**-1.140**
UPB1	Homo sapiens ureidopropionase, beta (UPB1), mRNA [NM_016327]	**-1.140**
SYT17	Homo sapiens synaptotagmin XVII (SYT17), mRNA [NM_016524]	**-1.139**
C16orf59	Homo sapiens chromosome 16 open reading frame 59 (C16orf59), mRNA [NM_025108]	**-1.136**
C18orf18	Homo sapiens chromosome 18 open reading frame 18, mRNA (cDNA clone MGC:17515 IMAGE:3457488), [BC010538]	**-1.136**
WNT5A	Homo sapiens wingless-type MMTV integration site family, member 5A (WNT5A), mRNA [NM_003392]	**-1.136**
FAM71E1	Homo sapiens family with sequence similarity 71, member E1 (FAM71E1), mRNA [NM_138411]	**-1.135**
FAM89B	Homo sapiens family with sequence similarity 89, member B (FAM89B), mRNA [NM_152832]	**-1.135**
TMC8	Homo sapiens transmembrane channel-like 8 (TMC8), mRNA [NM_152468]	**-1.134**
HPRT1	Homo sapiens hypoxanthine phosphoribosyltransferase 1 (HPRT1), mRNA [NM_000194]	**-1.134**
IL11	Homo sapiens interleukin 11 (IL11), mRNA [NM_000641]	**-1.134**
PLD4	Homo sapiens phospholipase D family, member 4 (PLD4), mRNA [NM_138790]	**-1.134**
ATP8B3	Homo sapiens cDNA FLJ43235 fis, clone HCHON2004007, highly similar to Potential phospholipid-transporting ATPase IK (EC 3.6.3.13). [AK125225]	**-1.133**
CRLF1	Homo sapiens cytokine receptor-like factor 1 (CRLF1), mRNA [NM_004750]	**-1.133**
HECTD2	Homo sapiens cDNA FLJ37306 fis, clone BRAMY2016325. [AK094625]	**-1.133**
PRKRA	Homo sapiens protein kinase, interferon-inducible double stranded RNA dependent activator (PRKRA), mRNA [NM_003690]	**-1.133**
C1orf113	Homo sapiens chromosome 1 open reading frame 113 (C1orf113), mRNA [NM_024676]	**-1.133**
LOC401588	Homo sapiens hypothetical LOC401588 (LOC401588), non-coding RNA [NR_015378]	**-1.132**
RBP1	Homo sapiens retinol binding protein 1, cellular (RBP1), mRNA [NM_002899]	**-1.130**
UNC5D	Homo sapiens unc-5 homolog D (C. elegans) (UNC5D), mRNA [NM_080872]	**-1.130**
LOC100129397	Homo sapiens cDNA FLJ38522 fis, clone HCHON2000818. [AK095841]	**-1.129**
ZNF486	Homo sapiens zinc finger protein 486 (ZNF486), mRNA [NM_052852]	**-1.129**
NUP35	Homo sapiens nucleoporin 35kDa (NUP35), mRNA [NM_138285]	**-1.128**
ZCCHC10	Homo sapiens zinc finger, CCHC domain containing 10 (ZCCHC10), mRNA	**-1.128**

	[NM_017665]	
ZFP62	Homo sapiens zinc finger protein 62 homolog (mouse) (ZFP62), mRNA [NM_152283]	**-1.128**
C15orf5	Homo sapiens chromosome 15 open reading frame 5, mRNA (cDNA clone MGC:97283 IMAGE:7262532), [BC069765]	**-1.127**
RFC4	Homo sapiens replication factor C (activator 1) 4, 37kDa (RFC4),mRNA [NM_002916]	**-1.126**
SVIP	Homo sapiens small VCP/p97-interacting protein (SVIP), mRNA [NM_148893]	**-1.126**
FBXL16	Homo sapiens F-box and leucine-rich repeat protein 16 (FBXL16), mRNA [NM_153350]	**-1.125**
IQCK	Homo sapiens IQ motif containing K (IQCK), mRNA [NM_153208]	**-1.123**
FLJ45244	Homo sapiens hypothetical locus FLJ45244 (FLJ45244), non-coding RNA [NR_015415]	**-1.122**
PHF10	Homo sapiens PHD finger protein 10 (PHF10), mRNA [NM_018288]	**-1.122**
GPR133	Homo sapiens G protein-coupled receptor 133 (GPR133), mRNA [NM_198827]	**-1.121**
SLC27A2	Homo sapiens solute carrier family 27, member 2 (SLC27A2), mRNA [NM_003645]	**-1.120**
CACNA2D2	Homo sapiens calcium channel, voltage-dependent, alpha 2/delta subunit 2 (CACNA2D2), mRNA [NM_001005505]	**-1.119**
FAM154A	Homo sapiens family with sequence similarity 154, member A (FAM154A), mRNA [NM_153707]	**-1.118**
FLJ22662	Homo sapiens hypothetical protein FLJ22662 (FLJ22662), mRNA [NM_024829]	**-1.118**
C1orf110	Homo sapiens chromosome 1 open reading frame 110 (C1orf110), mRNA [NM_178550]	**-1.117**
DYNLT1	Homo sapiens dynein, light chain, Tctex-type 1 (DYNLT1), mRNA [NM_006519]	**-1.117**
DCP1A	Homo sapiens DCP1 decapping enzyme homolog A (DCP1A), mRNA [NM_018403]	**-1.117**
BTG3	Homo sapiens BTG family, member 3 (BTG3), mRNA [NM_006806]	**-1.116**
CMC1	Homo sapiens COX assembly mitochondrial protein homolog (CMC1), mRNA [NM_182523]	**-1.116**
LOC645733	Homo sapiens similar to hCG1812929 (LOC645733), mRNA [XM_001130623]	**-1.116**
PLAGL2	Homo sapiens pleiomorphic adenoma gene-like 2 (PLAGL2), mRNA [NM_002657]	**-1.116**
LOC170082	TFIIS central domain-containing protein 1 [Source:UniProtKB/Swiss-Prot; Acc:Q8N8B7] [ENST00000380600]	**-1.115**
RIBC2	Homo sapiens RIB43A domain with coiled-coils 2 (RIBC2), mRNA [NM_015653]	**-1.115**
GPHB5	Homo sapiens glycoprotein hormone beta 5 (GPHB5), mRNA [NM_145171]	**-1.114**
KIF18B	Homo sapiens hypothetical protein LOC146909, mRNA (cDNA clone IMAGE:4418755), [BC048263]	**-1.114**

GALC	Homo sapiens galactosylceramidase (GALC), mRNA [NM_000153]	**-1.113**
C17orf97	Homo sapiens chromosome 17 open reading frame 97 (C17orf97), mRNA [NM_001013672]	**-1.112**
LONRF2	Homo sapiens LON peptidase N-terminal domain and ring finger 2 (LONRF2), mRNA [NM_198461]	**-1.112**
TAF12	Homo sapiens TAF12 RNA polymerase II, TATA box binding protein (TBP)-associated factor, (TAF12), mRNA [NM_005644]	**-1.112**
BRAF	Homo sapiens v-raf murine sarcoma viral oncogene homolog B1 (BRAF), mRNA [NM_004333]	**-1.111**
KIAA1199	Homo sapiens KIAA1199 (KIAA1199), mRNA [NM_018689]	**-1.111**
C20orf197	Homo sapiens chromosome 20 open reading frame 197 (C20orf197), mRNA [NM_173644]	**-1.110**
IDI2	Homo sapiens isopentenyl-diphosphate delta isomerase 2 (IDI2), mRNA [NM_033261]	**-1.110**
NCRNA00052	Homo sapiens cDNA FLJ31461 fis, clone NT2NE2001247. [AK056023]	**-1.110**
HS6ST2	Homo sapiens heparan sulfate 6-O-sulfotransferase 2 (HS6ST2),mRNA [NM_001077188]	**-1.109**
JARID2	Homo sapiens jumonji, AT rich interactive domain 2 (JARID2), mRNA [NM_004973]	**-1.108**
LILRA1	Homo sapiens leucocyte immunoglobulin-like receptor-6b (LIR-6) mRNA, complete cds. [AF025529]	**-1.107**
BCORL1	Homo sapiens BCL6 co-repressor-like 1 (BCORL1), mRNA [NM_021946]	**-1.106**
TNFAIP1	Homo sapiens tumor necrosis factor, alpha-induced protein 1 (endothelial) (TNFAIP1), mRNA [NM_021137]	**-1.106**
TNS4	Homo sapiens tensin 4 (TNS4), mRNA [NM_032865]	**-1.105**
CCDC34	Homo sapiens coiled-coil domain containing 34 (CCDC34),mRNA [NM_080654]	**-1.105**
EPHA5	Homo sapiens EPH receptor A5 (EPHA5), mRNA [NM_004439]	**-1.104**
PDGFRB	Homo sapiens platelet-derived growth factor receptor, beta polypeptide (PDGFRB), mRNA [NM_002609]	**-1.104**
KAT2A	Homo sapiens K(lysine) acetyltransferase 2A (KAT2A), mRNA [NM_021078]	**-1.103**
KCTD16	Homo sapiens potassium channel tetramerisation domain containing 16 (KCTD16), mRNA [NM_020768]	**-1.103**
PPP2R2B	Homo sapiens protein phosphatase 2 (formerly 2A), regulatory subunit B, beta isoform (PPP2R2B), mRNA [NM_004576]	**-1.103**
SLC4A4	Homo sapiens solute carrier family 4, sodium bicarbonate cotransporter, member 4 (SLC4A4), mRNA [NM_003759]	**-1.103**
BZW2	Homo sapiens basic leucine zipper and W2 domains 2 (BZW2), mRNA [NM_014038]	**-1.102**
MST150	Homo sapiens MSTP150 (MST150), mRNA [NM_032947]	**-1.102**
NUDT19	Homo sapiens nudix (nucleoside diphosphate linked moiety X)-type motif 19	**-1.101**

	(NUDT19), mRNA [NM_001105570]	
ALDH6A1	Homo sapiens aldehyde dehydrogenase 6 family, member A1 (ALDH6A1), mRNA [NM_005589]	**-1.100**
CRB1	Homo sapiens crumbs homolog 1 (Drosophila) (CRB1), mRNA [NM_201253]	**-1.100**
TNP2	Homo sapiens transition protein 2 (during histone to protamine replacement) (TNP2), mRNA [NM_005425]	**-1.099**
GLP2R	Glucagon-like peptide 2 receptor Precursor (GLP-2-R) [Source:UniProtKB/Swiss-Prot; Acc:O95838] [ENST00000396206]	**-1.098**
RPL32P3	Homo sapiens ribosomal protein L32 pseudogene 3 (RPL32P3), non-coding RNA [NR_003111]	**-1.097**
CDCA7	Homo sapiens cell division cycle associated 7 (CDCA7), mRNA [NM_031942]	**-1.097**
AKAP1	Homo sapiens A kinase (PRKA) anchor protein 1 (AKAP1),mRNA [NM_003488]	**-1.096**
CYB5R2	Homo sapiens cytochrome b5 reductase 2 (CYB5R2), mRNA [NM_016229]	**-1.096**
RAB15	Homo sapiens RAB15, member RAS onocogene family (RAB15), mRNA [NM_198686]	**-1.096**
ZC3H12A	Homo sapiens zinc finger CCCH-type containing 12A (ZC3H12A), mRNA [NM_025079]	**-1.096**
METTL7A	Homo sapiens methyltransferase like 7A (METTL7A), mRNA [NM_014033]	**-1.095**
CYP24A1	Homo sapiens cytochrome P450, family 24, subfamily A, polypeptide 1 (CYP24A1), mRNA [NM_000782]	**-1.094**
KIAA0101	Homo sapiens KIAA0101 (KIAA0101), mRNA [NM_014736]	**-1.092**
KRT19	Homo sapiens keratin 19 (KRT19), mRNA [NM_002276]	**-1.091**
RAD21	Homo sapiens RAD21 homolog (RAD21), mRNA [NM_006265]	**-1.091**
WDR78	Homo sapiens WD repeat domain 78 (WDR78), mRNA [NM_024763]	**-1.091**
BFSP1	Homo sapiens beaded filament structural protein 1, filensin (BFSP1), mRNA [NM_001195]	**-1.090**
GAS2L1	Homo sapiens growth arrest-specific 2 like 1 (GAS2L1), mRNA [NM_152236]	**-1.090**
PLSCR3	Homo sapiens phospholipid scramblase 3 (PLSCR3), mRNA [NM_020360]	**-1.090**
SERPINB1	Homo sapiens serpin peptidase inhibitor, clade B (ovalbumin), member 1 (SERPINB1), mRNA [NM_030666]	**-1.089**
CC2D2B	Homo sapiens coiled-coil and C2 domain containing 2B (CC2D2B), mRNA [NM_001001732]	**-1.088**
SETBP1	Homo sapiens SET binding protein 1 (SETBP1), mRNA [NM_015559]	**-1.086**
LOC440348	Homo sapiens similar to nuclear pore complex interacting protein (LOC440348), mRNA [NM_001018059]	**-1.085**
IGH@	Homo sapiens cDNA FLJ27104 fis, clone SPL04981, [AK130614]	**-1.084**
GNAO1	Homo sapiens cDNA clone IMAGE:4181241. [BC012202]	**-1.083**
FRAT2	Homo sapiens frequently rearranged in advanced T-cell lymphomas 2 (FRAT2), mRNA [NM_012083]	**-1.082**
PPM1D	Homo sapiens protein phosphatase 1D magnesium-dependent, delta isoform	**-1.082**

	(PPM1D), mRNA [NM_003620]	
EP400NL	Homo sapiens EP400 N-terminal like, mRNA (cDNA clone IMAGE:4374107). [BC066974]	**-1.081**
MGC4473	Homo sapiens hypothetical LOC79100 (MGC4473), non-coding RNA [NR_024160]	**-1.081**
PODXL	Homo sapiens podocalyxin-like (PODXL), mRNA [NM_001018111]	**-1.081**
IKZF2	Homo sapiens IKAROS family zinc finger 2 (Helios) (IKZF2),mRNA [NM_001079526]	**-1.080**
BUD13	Homo sapiens BUD13 homolog (S. cerevisiae) (BUD13), mRNA [NM_032725]	**-1.077**
FEM1B	Homo sapiens fem-1 homolog b (C. elegans) (FEM1B), mRNA [NM_015322]	**-1.077**
DUSP12	Homo sapiens dual specificity phosphatase 12 (DUSP12), mRNA [NM_007240]	**-1.076**
GTF3A	Homo sapiens general transcription factor IIIA (GTF3A), mRNA [NM_002097]	**-1.076**
E2F2	Homo sapiens E2F transcription factor 2 (E2F2), mRNA [NM_004091]	**-1.074**
EFCAB10	Homo sapiens cDNA clone IMAGE:6616931, [BC062748]	**-1.074**
FAM27A	Homo sapiens family with sequence similarity 27, member A (FAM27A), non-coding RNA [NR_024060]	**-1.074**
NFKBIA	Homo sapiens nuclear factor of kappa light polypeptide gene enhancer in B-cells inhibitor, alpha (NFKBIA), mRNA [NM_020529]	**-1.074**
CD58	Homo sapiens CD58 molecule (CD58), mRNA [NM_001779]	**-1.073**
C3orf15	Homo sapiens chromosome 3 open reading frame 15 (C3orf15), mRNA [NM_033364]	**-1.072**
SLC34A3	Homo sapiens solute carrier family 34 (sodium phosphate), member 3 (SLC34A3), mRNA [NM_080877]	**-1.072**
CNKSR3	Homo sapiens CNKSR family member 3 (CNKSR3), mRNA [NM_173515]	**-1.071**
FANCA	Homo sapiens Fanconi anemia, complementation group A (FANCA), mRNA [NM_000135]	**-1.071**
ORMDL1	Homo sapiens ORM1-like 1 (S. cerevisiae) (ORMDL1), mRNA [NM_016467]	**-1.071**
LAMP3	Homo sapiens lysosomal-associated membrane protein 3 (LAMP3), mRNA [NM_014398]	**-1.070**
HEBP1	Homo sapiens heme binding protein 1 (HEBP1), mRNA [NM_015987]	**-1.068**
KIAA1524	Homo sapiens KIAA1524 (KIAA1524), mRNA [NM_020890]	**-1.068**
LLGL1	Homo sapiens lethal giant larvae homolog 1 (Drosophila) (LLGL1), mRNA [NM_004140]	**-1.068**
MATN3	Homo sapiens matrilin 3 (MATN3), mRNA [NM_002381]	**-1.068**
OCIAD1	Homo sapiens OCIA domain containing 1 (OCIAD1), mRNA [NM_001079839]	**-1.068**
ST6GAL1	Homo sapiens ST6 beta-galactosamide alpha-2,6-sialyltranferase 1 (ST6GAL1), mRNA [NM_173216]	**-1.068**
CLEC16A	Homo sapiens C-type lectin domain family 16, member A (CLEC16A), mRNA [NM_015226]	**-1.067**
LOC730091	Homo sapiens cDNA FLJ31839 fis, clone NT2RP7000086. [AK056401]	**-1.067**
TRIM66	Homo sapiens tripartite motif-containing 66 (TRIM66), mRNA [NM_014818]	**-1.067**

RACGAP1	Homo sapiens Rac GTPase activating protein 1 (RACGAP1),mRNA [NM_013277]	**-1.067**
C10orf114	Homo sapiens chromosome 10 open reading frame 114 (C10orf114), mRNA [NM_001010911]	**-1.066**
FOXS1	Homo sapiens forkhead box S1 (FOXS1), mRNA [NM_004118]	**-1.066**
LOC442175	Homo sapiens similar to hCG1811681 (LOC442175), mRNA [XM_001130492]	**-1.066**
ZSCAN20	Homo sapiens zinc finger and SCAN domain containing 20 (ZSCAN20), mRNA [NM_145238]	**-1.065**
DERA	Homo sapiens 2-deoxyribose-5-phosphate aldolase homolog (C. elegans) (DERA), mRNA [NM_015954]	**-1.064**
SERPINB9	Homo sapiens serpin peptidase inhibitor, clade B (ovalbumin), member 9 (SERPINB9), mRNA [NM_004155]	**-1.064**
LOC283481	Homo sapiens hypothetical protein LOC283481, mRNA (cDNA clone IMAGE:5296747). [BC033993]	**-1.062**
SYT15	Homo sapiens synaptotagmin XV (SYT15), mRNA [NM_181519]	**-1.062**
MRPS18B	Homo sapiens mitochondrial ribosomal protein S18B (MRPS18B),mRNA [NM_014046]	**-1.061**
ZYG11A	Protein zyg-11 homolog A [Source:UniProtKB/Swiss-Prot;Acc:Q6WRX3] [ENST00000371528]	**-1.061**
CDKN2D	Homo sapiens cyclin-dependent kinase inhibitor 2D (p19, inhibits CDK4) (CDKN2D), mRNA [NM_001800]	**-1.060**
ROBO2	Homo sapiens roundabout, axon guidance receptor, homolog 2 (Drosophila) (ROBO2), mRNA [NM_002942]	**-1.060**
SENP6	Homo sapiens SUMO1/sentrin specific peptidase 6 (SENP6),mRNA [NM_015571]	**-1.060**
FAIM	Homo sapiens Fas apoptotic inhibitory molecule (FAIM),mRNA [NM_001033030]	**-1.059**
LOC442245	Homo sapiens glutathione S-transferase M1 pseudogene (LOC442245), non-coding RNA [NR_002932]	**-1.058**
SOLH	Homo sapiens small optic lobes homolog (Drosophila) (SOLH), mRNA [NM_005632]	**-1.058**
FLJ20674	Homo sapiens hypothetical protein FLJ20674 (FLJ20674), mRNA [NM_019086]	**-1.058**
ARMCX2	Homo sapiens armadillo repeat containing, X-linked 2 (ARMCX2), mRNA [NM_014782]	**-1.057**
EFCAB2	Homo sapiens EF-hand calcium binding domain 2 (EFCAB2), mRNA [NM_032328]	**-1.057**
OR11A1	Homo sapiens olfactory receptor, family 11, subfamily A, member 1 (OR11A1), mRNA [NM_013937]	**-1.057**
RAB3A	Homo sapiens RAB3A, member RAS oncogene family (RAB3A), mRNA [NM_002866]	**-1.057**
C18orf55	Homo sapiens chromosome 18 open reading frame 55 (C18orf55), mRNA	**-1.056**

	[NM_014177]	
H1FNT	Homo sapiens H1 histone family, member N, testis-specific (H1FNT), mRNA [NM_181788]	**-1.056**
NID2	Homo sapiens nidogen 2 (osteonidogen) (NID2), mRNA [NM_007361]	**-1.056**
PITX2	Homo sapiens paired-like homeodomain 2 (PITX2), mRNA [NM_153426]	**-1.056**
LOC100130890	Homo sapiens mRNA; cDNA DKFZp781B0241 (from clone DKFZp781B0241). [BX647075]	**-1.055**
RABEPK	Homo sapiens Rab9 effector protein with kelch motifs (RABEPK), mRNA [NM_005833]	**-1.055**
BCKDHB	Homo sapiens branched chain keto acid dehydrogenase E1, beta polypeptide (BCKDHB),mRNA [NM_183050]	**-1.055**
C5orf13	Homo sapiens chromosome 5 open reading frame 13 (C5orf13),mRNA [NM_004772]	**-1.054**
FKBP2	Homo sapiens FK506 binding protein 2, 13kDa (FKBP2), mRNA [NM_004470]	**-1.054**
GARNL3	Homo sapiens GTPase activating Rap/RanGAP domain-like 3 (GARNL3), mRNA [NM_032293]	**-1.054**
MKX	Homo sapiens mohawk homeobox (MKX), mRNA [NM_173576]	**-1.054**
KLRG1	Homo sapiens killer cell lectin-like receptor subfamily G, member 1 (KLRG1), mRNA [NM_005810]	**-1.053**
LOC154822	Homo sapiens hypothetical LOC154822 (LOC154822), non-coding RNA [NR_024394]	**-1.053**
RBPMS2	Homo sapiens RNA binding protein with multiple splicing 2 (RBPMS2), mRNA [NM_194272]	**-1.050**
LOC400027	Homo sapiens cDNA clone IMAGE:5288894. [BC047417]	**-1.050**
GPHA2	Homo sapiens glycoprotein hormone alpha 2 (GPHA2), mRNA [NM_130769]	**-1.049**
ZNF284	Zinc finger protein 284 [Source:UniProtKB/ Swiss-Prot;Acc:Q2VY69] [ENST00000328297]	**-1.049**
ADA	Homo sapiens adenosine deaminase (ADA), mRNA [NM_000022]	**-1.048**
RNF214	Homo sapiens ring finger protein 214 (RNF214), mRNA [NM_001077239]	**-1.048**
TMEM129	Homo sapiens transmembrane protein 129 (TMEM129), mRNA [NM_138385]	**-1.048**
TUBA8	Homo sapiens tubulin, alpha 8 (TUBA8), mRNA [NM_018943]	**-1.048**
LOC100130967	Putative uncharacterized protein C6orf99 [Source:UniProtKB/Swiss-Prot; Acc:Q4VX62] [ENST00000367073]	**-1.047**
LOC728198	Homo sapiens similar to transcription associated factor TAFII31L (LOC728198), mRNA [XM_001126120]	**-1.046**
STAG3	Homo sapiens stromal antigen 3 (STAG3), mRNA [NM_012447]	**-1.046**
CELSR2	Homo sapiens cadherin, EGF LAG seven-pass G-type receptor 2(CELSR2), mRNA [NM_001408]	**-1.045**
RASSF5	Homo sapiens Ras association (RalGDS/AF-6) domain family member 5 (RASSF5), mRNA [NM_182663]	**-1.045**
MAGEA1	Homo sapiens melanoma antigen family A, 1 (directs expression of antigen	**-1.044**

	MZ2-E) (MAGEA1), mRNA [NM_004988]	
OXCT2	Homo sapiens 3-oxoacid CoA transferase 2 (OXCT2), mRNA [NM_022120]	**-1.044**
FAM136A	Homo sapiens family with sequence similarity 136, member A (FAM136A), mRNA [NM_032822]	**-1.043**
SORL1	Homo sapiens sortilin-related receptor, L(DLR class) A repeats-containing (SORL1), mRNA [NM_003105]	**-1.043**
NSBP1	Homo sapiens nucleosomal binding protein 1 (NSBP1), mRNA [NM_030763]	**-1.043**
DEPDC7	Homo sapiens DEP domain containing 7 (DEPDC7), mRNA [NM_139160]	**-1.042**
FAM162A	Homo sapiens family with sequence similarity 162, member A (FAM162A), mRNA [NM_014367]	**-1.041**
KIAA1466	Homo sapiens mRNA for KIAA1466 protein, [AB040899]	**-1.040**
HOXB3	Homo sapiens homeobox B3 (HOXB3), mRNA [NM_002146]	**-1.039**
EPN2	Homo sapiens epsin 2 (EPN2), mRNA [NM_014964]	**-1.038**
SHC3	Homo sapiens SHC (Src homology 2 domain containing) transforming protein 3 (SHC3), mRNA [NM_016848]	**-1.037**
MYCBP	Homo sapiens c-myc binding protein (MYCBP), mRNA [NM_012333]	**-1.037**
C17orf74	Homo sapiens chromosome 17 open reading frame 74 (C17orf74), mRNA [NM_175734]	**-1.036**
CCDC87	Homo sapiens coiled-coil domain containing 87 (CCDC87), mRNA [NM_018219]	**-1.036**
SEMA6C	Homo sapiens sema domain, transmembrane domain (TM), and cytoplasmic domain, (semaphorin) 6C (SEMA6C), mRNA [NM_030913]	**-1.036**
ABHD1	Homo sapiens abhydrolase domain containing 1, mRNA (cDNA clone IMAGE:4812401), [BC028378]	**-1.035**
XPA	Homo sapiens xeroderma pigmentosum, complementation group A (XPA), mRNA [NM_000380]	**-1.035**
APC2	Homo sapiens adenomatosis polyposis coli 2 (APC2), mRNA [NM_005883]	**-1.034**
GPR3	Homo sapiens G protein-coupled receptor 3 (GPR3), mRNA [NM_005281]	**-1.034**
ZCRB1	Homo sapiens zinc finger CCHC-type and RNA binding motif 1 (ZCRB1), mRNA [NM_033114]	**-1.034**
COL18A1	Homo sapiens collagen, type XVIII, alpha 1 (COL18A1), mRNA [NM_030582]	**-1.033**
FANCL	Homo sapiens Fanconi anemia, complementation group L (FANCL),mRNA [NM_018062]	**-1.033**
TMEM107	Homo sapiens transmembrane protein 107 (TMEM107),mRNA [NM_032354]	**-1.033**
LOC390413	Homo sapiens misc_RNA (LOC390413), miscRNA [XR_018341]	**-1.032**
XK	Homo sapiens X-linked Kx blood group (McLeod syndrome) (XK), mRNA [NM_021083]	**-1.031**
VCY	Homo sapiens variable charge, Y-linked (VCY), mRNA [NM_004679]	**-1.030**
FAM63A	Homo sapiens mRNA for KIAA1390 protein, [AB037811]	**-1.030**
PRDM13	Homo sapiens PR domain containing 13 (PRDM13), mRNA [NM_021620]	**-1.029**

LEPROTL1	Homo sapiens leptin receptor overlapping transcript-like 1 (LEPROTL1), mRNA [NM_015344]	**-1.028**
FER1L4	Homo sapiens fer-1-like 4 (C. elegans) (FER1L4), non-coding RNA [NR_024377]	**-1.027**
PAQR6	Homo sapiens progestin and adipoQ receptor family member VI (PAQR6), mRNA [NM_024897]	**-1.027**
LOC126235	Full-length cDNA clone CS0DE004YN04 of Placenta of Homo sapiens (human). [CR622909]	**-1.026**
LOC729684	Homo sapiens misc_RNA (LOC729684), miscRNA [XR_039360]	**-1.026**
OGDHL	Homo sapiens oxoglutarate dehydrogenase-like (OGDHL), mRNA [NM_018245]	**-1.026**
PMS2L1	Homo sapiens postmeiotic segregation increased 2-like 1, mRNA (cDNA clone IMAGE:6049115), [BC044214]	**-1.026**
RGL1	Homo sapiens ral guanine nucleotide dissociation stimulator-like 1 (RGL1), mRNA [NM_015149]	**-1.026**
PLA2R1	Human 180 kDa transmembrane PLA2 receptor mRNA, [U17033]	**-1.025**
THAP1	Homo sapiens THAP domain containing, apoptosis associated protein 1 (THAP1), mRNA [NM_018105]	**-1.025**
C20orf29	Homo sapiens chromosome 20 open reading frame 29 (C20orf29), mRNA [NM_018347]	**-1.024**
GGCT	Homo sapiens gamma-glutamyl cyclotransferase (GGCT), mRNA [NM_024051]	**-1.024**
IRAK1BP1	Homo sapiens interleukin-1 receptor-associated kinase 1 binding protein 1 (IRAK1BP1), mRNA [NM_001010844]	**-1.024**
COCH	Homo sapiens coagulation factor C homolog, cochlin (Limulus polyphemus) (COCH), mRNA [NM_004086]	**-1.023**
CENPK	Homo sapiens centromere protein K (CENPK), mRNA [NM_022145]	**-1.023**
KCNJ14	Homo sapiens potassium inwardly-rectifying channel, subfamily J, member 14 (KCNJ14), mRNA [NM_170720]	**-1.023**
LOC389992	Homo sapiens similar to hCG2040259 (LOC389992), mRNA [XM_001720568]	**-1.023**
PC	Homo sapiens pyruvate carboxylase (PC), mRNA [NM_001040716]	**-1.023**
BNIP3	Homo sapiens BCL2/adenovirus E1B 19kDa interacting protein 3 (BNIP3), mRNA [NM_004052]	**-1.022**
EML1	Homo sapiens echinoderm microtubule associated protein like 1 (EML1), mRNA [NM_001008707]	**-1.022**
TDP1	Homo sapiens tyrosyl-DNA phosphodiesterase 1 (TDP1),mRNA [NM_018319]	**-1.022**
PON2	Homo sapiens paraoxonase 2 (PON2), mRNA [NM_000305]	**-1.021**
GPR183	Homo sapiens G protein-coupled receptor 183 (GPR183), mRNA [NM_004951]	**-1.020**
MAP6D1	Homo sapiens MAP6 domain containing 1 (MAP6D1), mRNA [NM_024871]	**-1.020**
MYLC2PL	Myosin light chain 2, lymphocyte-specific (Precursor lymphocyte-specific regulatory light chain) [Source:UniProtKB/ Swiss-Prot; Acc:Q9BUA6] [ENST00000223167]	**-1.020**

PAIP2	Homo sapiens poly(A) binding protein interacting protein 2 (PAIP2), mRNA [NM_001033112]	**-1.020**
BIC	Homo sapiens BIC transcript (BIC), non-coding RNA [NR_001458]	**-1.019**
SPTBN4	Homo sapiens spectrin, beta, non-erythrocytic 4 (SPTBN4),mRNA [NM_025213]	**-1.019**
WFDC10B	Homo sapiens WAP four-disulfide core domain 10B (WFDC10B),mRNA [NM_172006]	**-1.019**
RELL1	Homo sapiens RELT-like 1 (RELL1), mRNA [NM_001085400]	**-1.018**
TPD52L1	Homo sapiens tumor protein D52-like 1 (TPD52L1), mRNA [NM_001003395]	**-1.018**
FYCO1	Homo sapiens FYVE and coiled-coil domain containing 1 (FYCO1), mRNA [NM_024513]	**-1.016**
COL4A2	Homo sapiens collagen, type IV, alpha 2 (COL4A2), mRNA [NM_001846]	**-1.015**
FLJ10213	Homo sapiens hypothetical protein FLJ10213 (FLJ10213), mRNA [NM_018029]	**-1.015**
AP3S1	Homo sapiens adaptor-related protein complex 3, sigma 1 subunit (AP3S1), mRNA [NM_001284]	**-1.014**
HIGD1A	Homo sapiens HIG1 domain family, member 1A (HIGD1A),mRNA [NM_001099668]	**-1.014**
NEFH	Homo sapiens neurofilament, heavy polypeptide (NEFH), mRNA [NM_021076]	**-1.014**
PLEK2	Homo sapiens pleckstrin 2 (PLEK2), mRNA [NM_016445]	**-1.014**
HGSNAT	Homo sapiens heparan-alpha-glucosaminide N-acetyltransferase (HGSNAT), mRNA [NM_152419]	**-1.014**
BTBD8	Homo sapiens BTB (POZ) domain containing 8 (BTBD8), mRNA [NM_183242]	**-1.013**
C3orf45	Homo sapiens chromosome 3 open reading frame 45 (C3orf45), mRNA [NM_153215]	**-1.013**
FOXD2	Homo sapiens forkhead box D2 (FOXD2), mRNA [NM_004474]	**-1.013**
SNX6	Homo sapiens sorting nexin 6 (SNX6), mRNA [NM_021249]	**-1.012**
C4orf12	Homo sapiens cDNA FLJ42672 fis, clone BRAMY2026533. [AK124663]	**-1.012**
AMOTL2	Homo sapiens angiomotin like 2 (AMOTL2), mRNA [NM_016201]	**-1.011**
LOC439911	Homo sapiens cDNA FLJ45542 fis, clone BRTHA2033320. [AK127450]	**-1.010**
MCEE	Homo sapiens methylmalonyl CoA epimerase (MCEE), mRNA [NM_032601]	**-1.010**
TNFRSF11A	Homo sapiens mRNA for tumor necrosis factor receptor superfamily, member 11a precursor variant protein. [AB209762]	**-1.010**
AP4S1	Homo sapiens adaptor-related protein complex 4, sigma 1 subunit (AP4S1), mRNA [NM_001128126]	**-1.010**
C13orf15	Homo sapiens chromosome 13 open reading frame 15 (C13orf15), mRNA [NM_014059]	**-1.009**
KPNA5	Homo sapiens karyopherin alpha 5 (importin alpha 6) (KPNA5), mRNA [NM_002269]	**-1.009**
LOC286254	Homo sapiens cDNA FLJ35432 fis, clone SMINT2002311. [AK092751]	**-1.009**

TCP11L1	Homo sapiens t-complex 11 (mouse)-like 1 (TCP11L1), mRNA [NM_018393]	**-1.009**
PPP1R1C	Homo sapiens protein phosphatase 1, regulatory (inhibitor) subunit 1C (PPP1R1C), mRNA [NM_001080545]	**-1.008**
TRIT1	Homo sapiens tRNA isopentenyltransferase 1 (TRIT1), mRNA [NM_017646]	**-1.008**
ZDHHC20	Homo sapiens zinc finger, DHHC-type containing 20 (ZDHHC20), mRNA [NM_153251]	**-1.008**
ZNF828	Homo sapiens zinc finger protein 828 (ZNF828), mRNA [NM_032436]	**-1.008**
USP39	Homo sapiens ubiquitin specific peptidase 39 (USP39), mRNA [NM_006590]	**-1.007**
C16orf35	Homo sapiens chromosome 16 open reading frame 35 (C16orf35),mRNA [NM_001039476]	**-1.007**
ARL3	Homo sapiens ADP-ribosylation factor-like 3 (ARL3), mRNA [NM_004311]	**-1.006**
ARMC4	Homo sapiens cDNA FLJ10376 fis, clone NT2RM2001982. [AK001238]	**-1.006**
BEND3	Homo sapiens BEN domain containing 3 (BEND3), mRNA [NM_001080450]	**-1.006**
IL22	Homo sapiens interleukin 22 (IL22), mRNA [NM_020525]	**-1.006**
STOX1	Homo sapiens storkhead box 1 (STOX1), mRNA [NM_152709]	**-1.006**
C12orf60	Homo sapiens chromosome 12 open reading frame 60 (C12orf60), mRNA [NM_175874]	**-1.005**
CRYL1	Homo sapiens crystallin, lambda 1 (CRYL1), mRNA [NM_015974]	**-1.005**
HIST1H2BK	Homo sapiens histone cluster 1, H2bk (HIST1H2BK), mRNA [NM_080593]	**-1.005**
LOC646993	Homo sapiens similar to high-mobility group box 3 (LOC646993), mRNA [XM_929965]	**-1.005**
ZNF446	Homo sapiens zinc finger protein 446 (ZNF446), mRNA [NM_017908]	**-1.005**
IFNE	Homo sapiens interferon, epsilon (IFNE), mRNA [NM_176891]	**-1.004**
TMEM106C	Homo sapiens transmembrane protein 106C (TMEM106C),mRNA [NM_024056]	**-1.004**
ZNF175	Homo sapiens zinc finger protein 175, mRNA (cDNA clone IMAGE:4301632), [BC007778]	**-1.004**
LOC221442	Homo sapiens cDNA FLJ44104 fis, clone TESTI4044123. [AK126092]	**-1.003**
RBM35B	Homo sapiens RNA binding motif protein 35B (RBM35B), mRNA [NM_024939]	**-1.003**
RWDD1	Homo sapiens RWD domain containing 1 (RWDD1), mRNA [NM_016104]	**-1.003**
C9orf125	Transmembrane protein C9orf125 [Source:UniProtKB/ Swiss-Prot; Acc:Q9BRR3] [ENST00000374851]	**-1.002**
DKFZP434B061	Homo sapiens mRNA; cDNA DKFZp434B061 (from clone DKFZp434B061); [AL117481]	**-1.002**
LOC402360	Homo sapiens similar to hCG1742476 (LOC402360), mRNA [XM_001722407]	**-1.002**
MAP2K6	Human MAP kinase kinase 6 (MKK6) mRNA, complete cds. [U39657]	**-1.000**
ZNF580	Homo sapiens zinc finger protein 580 (ZNF580), mRNA [NM_016202]	**-1.000**
