# Supplementary material for: Genotoxicity and molecular response of silver nanoparticle (NP)-based hydrogel
Source: J Nanobiotechnology. 2012 May 1;10:16. doi: 10.1186/1477-3155-10-16 (PMC3430588; doi:10.1186/1477-3155-10-16)
Supplement: Additional file 3 — Up-regulated genes in cells exposed to silver-NPs-hydrogel for 48h. Fold-change is logarithmic ratio (log2 ratio) to expression level in control. [file 1477-3155-10-16-S3.pdf]

Additional File 3. Up-regulated genes in cells exposed to silver-NPs-hydrogel for 48h. Fold-change is logarithmic ratio (log_2 ratio) to expression level in control.

GeneName	Description	Fold-change (log_2 ratio)
MT1F	Homo sapiens metallothionein 1F (MT1F), mRNA [NM_005949]	**5.638**
SPINK1	Homo sapiens serine peptidase inhibitor, Kazal type 1 (SPINK1), mRNA [NM_003122]	**5.205**
MT1JP	Homo sapiens MTB (MTB) mRNA. [AF348994]	**4.942**
LY96	Homo sapiens lymphocyte antigen 96 (LY96), mRNA [NM_015364]	**4.702**
MT1A	Homo sapiens metallothionein 1A (MT1A), mRNA [NM_005946]	**4.340**
GCNT3	Homo sapiens glucosaminyl (N-acetyl) transferase 3, mucin type (GCNT3), mRNA [NM_004751]	**4.091**
MT2A	Homo sapiens metallothionein 2A (MT2A), mRNA [NM_005953]	**3.922**
MT1B	Homo sapiens metallothionein 1B (MT1B), mRNA [NM_005947]	**3.900**
MT1G	Homo sapiens metallothionein 1G (MT1G), mRNA [NM_005950]	**3.853**
PTPRR	Homo sapiens protein tyrosine phosphatase, receptor type, R (PTPRR), mRNA [NM_002849]	**3.828**
TM4SF19	Homo sapiens transmembrane 4 L six family member 19 (TM4SF19), mRNA [NM_138461]	**3.783**
MT1H	Homo sapiens metallothionein 1H (MT1H), mRNA [NM_005951]	**3.749**
GPNMB	Homo sapiens glycoprotein (transmembrane) nmb, mRNA (cDNA clone IMAGE:3345861). [BC011595]	**3.735**
KRT34	Homo sapiens keratin 34 (KRT34), mRNA [NM_021013]	**3.699**
KBTBD10	Homo sapiens kelch repeat and BTB (POZ) domain containing 10 (KBTBD10), mRNA [NM_006063]	**3.697**
MT1X	Homo sapiens metallothionein 1X (MT1X), mRNA [NM_005952]	**3.681**
IFIH1	Homo sapiens interferon induced with helicase C domain 1 (IFIH1), mRNA [NM_022168]	**3.510**
IFIT2	Homo sapiens interferon-induced protein with tetratricopeptide repeats 2 (IFIT2), mRNA [NM_001547]	**3.467**
OASL	Homo sapiens 2'-5'-oligoadenylate synthetase-like (OASL), mRNA [NM_003733]	**3.447**
PSG6	Homo sapiens pregnancy specific beta-1-glycoprotein 6 (PSG6), mRNA [NM_002782]	**3.438**
SH2D5	Homo sapiens SH2 domain containing 5 (SH2D5), mRNA [NM_001103161]	**3.394**
TNIP3	Homo sapiens TNFAIP3 interacting protein 3 (TNIP3), mRNA [NM_024873]	**3.384**
IFIT3	Homo sapiens interferon-induced protein with tetratricopeptide repeats 3 (IFIT3), mRNA [NM_001549]	**3.378**
SCG5	Homo sapiens secretogranin V (7B2 protein) (SCG5), mRNA [NM_003020]	**3.361**
PSG9	Homo sapiens pregnancy specific beta-1-glycoprotein 9 (PSG9), mRNA [NM_002784]	**3.295**
CASP1	Homo sapiens caspase 1, apoptosis-related cysteine peptidase (interleukin 1, beta, convertase) (CASP1), mRNA [NM_033292]	**3.273**

LOC284344	Homo sapiens cDNA FLJ40353 fis, clone TESTI2033520, weakly similar to Biliary glycoprotein 1 precursor [AK097672]	**3.220**
PSG8	Homo sapiens pregnancy specific beta-1-glycoprotein 8 (PSG8), mRNA [NM_182707]	**3.208**
MT1L	Homo sapiens metallothionein 1L (gene/pseudogene) (MT1L), non-coding RNA [NR_001447]	**3.160**
MME	Homo sapiens membrane metallo-endopeptidase (MME), mRNA [NM_007289]	**3.143**
C4orf18	Homo sapiens chromosome 4 open reading frame 18 (C4orf18), mRNA [NM_016613]	**3.130**
GDF15	Homo sapiens growth differentiation factor 15 (GDF15), mRNA [NM_004864]	**3.111**
IL21R	Homo sapiens interleukin 21 receptor (IL21R), mRNA [NM_181078]	**3.059**
CLEC2B	Homo sapiens C-type lectin domain family 2, member B (CLEC2B), mRNA [NM_005127]	**3.039**
IFIT1	Homo sapiens interferon-induced protein with tetratricopeptide repeats 1 (IFIT1), mRNA [NM_001548]	**3.034**
AGT	Homo sapiens angiotensinogen (serpin peptidase inhibitor, clade A, member 8) (AGT), mRNA [NM_000029]	**3.010**
SPANXD	Homo sapiens SPANX family, member D (SPANXD), mRNA [NM_032417]	**2.988**
TM7SF2	Homo sapiens transmembrane 7 superfamily member 2 (TM7SF2), mRNA [NM_003273]	**2.981**
FOLR1	Homo sapiens folate receptor 1 (adult) (FOLR1), mRNA [NM_016725]	**2.980**
RTP4	Homo sapiens receptor (chemosensory) transporter protein 4 (RTP4), mRNA [NM_022147]	**2.928**
MLPH	Homo sapiens melanophilin (MLPH), mRNA [NM_024101]	**2.911**
IL24	Homo sapiens interleukin 24 (IL24), mRNA [NM_006850]	**2.891**
HKDC1	Homo sapiens hexokinase domain containing 1 (HKDC1), mRNA [NM_025130]	**2.882**
PSG4	Homo sapiens pregnancy specific beta-1-glycoprotein 4 (PSG4), mRNA [NM_213633]	**2.876**
C1S	Homo sapiens complement component 1, s subcomponent (C1S), mRNA [NM_001734]	**2.858**
WDR66	Homo sapiens WD repeat domain 66 (WDR66), mRNA [NM_144668]	**2.851**
SPANXB2	Homo sapiens SPANX family, member B2 (SPANXB2), mRNA [NM_145664]	**2.828**
PSG1	Homo sapiens pregnancy specific beta-1-glycoprotein 1 (PSG1), mRNA [NM_006905]	**2.820**
CCL26	Homo sapiens chemokine (C-C motif) ligand 26 (CCL26), mRNA [NM_006072]	**2.802**
PSG7	Homo sapiens pregnancy specific beta-1-glycoprotein 7 (PSG7), mRNA [NM_002783]	**2.794**
MCOLN3	Homo sapiens mucolipin 3 (MCOLN3), mRNA [NM_018298]	**2.789**
PDE2A	Homo sapiens phosphodiesterase 2A, cGMP-stimulated (PDE2A), mRNA	**2.777**

	[NM_002599]	
IL1A	Homo sapiens interleukin 1, alpha (IL1A), mRNA [NM_000575]	**2.761**
LIPG	Homo sapiens lipase, endothelial (LIPG), mRNA [NM_006033]	**2.748**
PLA2G3	Homo sapiens phospholipase A2, group III (PLA2G3), mRNA [NM_015715]	**2.746**
CYTIP	Homo sapiens cytohesin 1 interacting protein (CYTIP), mRNA [NM_004288]	**2.730**
SPP1	Homo sapiens secreted phosphoprotein 1 (SPP1), mRNA [NM_001040058]	**2.711**
STAT4	Homo sapiens signal transducer and activator of transcription 4 (STAT4), mRNA [NM_003151]	**2.710**
DHX58	Homo sapiens DEXH (Asp-Glu-X-His) box polypeptide 58 (DHX58), mRNA [NM_024119]	**2.707**
MGC23284	Homo sapiens hypothetical LOC197187 (MGC23284), non-coding RNA [NR_024399]	**2.685**
RSPO3	Homo sapiens R-spondin 3 homolog (Xenopus laevis) (RSPO3), mRNA [NM_032784]	**2.683**
PGM5	Homo sapiens phosphoglucomutase 5 (PGM5), mRNA [NM_021965]	**2.677**
GLRA3	Homo sapiens glycine receptor, alpha 3 (GLRA3), mRNA [NM_006529]	**2.631**
IL2RB	Homo sapiens interleukin 2 receptor, beta (IL2RB), mRNA [NM_000878]	**2.619**
DUSP5	Homo sapiens dual specificity phosphatase 5 (DUSP5), mRNA [NM_004419]	**2.599**
CCL5	Homo sapiens chemokine (C-C motif) ligand 5 (CCL5), mRNA [NM_002985]	**2.584**
SPANXA1	Homo sapiens sperm protein associated with the nucleus, X-linked, family member A1 (SPANXA1), mRNA [NM_013453]	**2.579**
HSPB3	Homo sapiens heat shock 27kDa protein 3 (HSPB3), mRNA [NM_006308]	**2.569**
MX1	Homo sapiens myxovirus (influenza virus) resistance 1, interferon-inducible protein p78 (mouse) (MX1), mRNA [NM_002462]	**2.557**
C15orf48	Homo sapiens chromosome 15 open reading frame 48 (C15orf48), mRNA [NM_032413]	**2.549**
SQSTM1	Human phosphotyrosine independent ligand p62B B-cell isoform for the Lck SH2 domain mRNA. [U46752]	**2.539**
CSTA	Homo sapiens cystatin A (stefin A) (CSTA), mRNA [NM_005213]	**2.531**
CXCL11	Homo sapiens chemokine (C-X-C motif) ligand 11 (CXCL11), mRNA [NM_005409]	**2.530**
FAM49A	Homo sapiens family with sequence similarity 49, member A (FAM49A), mRNA [NM_030797]	**2.528**
C3orf55	Homo sapiens chromosome 3 open reading frame 55 (C3orf55), mRNA [NM_001099777]	**2.520**
FAM26E	Homo sapiens family with sequence similarity 26, member E (FAM26E), mRNA [NM_153711]	**2.519**
MT1M	Homo sapiens metallothionein 1M (MT1M), mRNA [NM_176870]	**2.513**
RGS4	Homo sapiens regulator of G-protein signaling 4 (RGS4), mRNA [NM_005613]	**2.499**
OSGIN1	Homo sapiens oxidative stress induced growth inhibitor 1 (OSGIN1), mRNA [NM_013370]	**2.488**
IFI44	Homo sapiens interferon-induced protein 44 (IFI44), mRNA [NM_006417]	**2.485**

PPP1R15A	Homo sapiens protein phosphatase 1, regulatory (inhibitor) subunit 15A (PPP1R15A), mRNA [NM_014330]	**2.478**
ALDOC	Homo sapiens aldolase C, fructose-bisphosphate (ALDOC), mRNA [NM_005165]	**2.475**
ICAM1	Homo sapiens intercellular adhesion molecule 1 (ICAM1), mRNA [NM_000201]	**2.474**
DTNA	Homo sapiens dystrobrevin, alpha (DTNA), mRNA [NM_001392]	**2.463**
MYO16	Homo sapiens myosin XVI (MYO16), mRNA [NM_015011]	**2.458**
DDX60	Homo sapiens DEAD (Asp-Glu-Ala-Asp) box polypeptide 60 (DDX60), mRNA [NM_017631]	**2.452**
RAP1GAP	Homo sapiens RAP1 GTPase activating protein (RAP1GAP), mRNA [NM_002885]	**2.434**
INSIG1	Homo sapiens insulin induced gene 1 (INSIG1), mRNA [NM_198336]	**2.430**
SAA1	Homo sapiens serum amyloid A1 (SAA1), mRNA [NM_000331]	**2.423**
DNMT3L	Homo sapiens DNA (cytosine-5-)-methyltransferase 3-like (DNMT3L), mRNA [NM_013369]	**2.420**
TMEM140	Homo sapiens transmembrane protein 140 (TMEM140), mRNA [NM_018295]	**2.414**
ACSS2	Homo sapiens acyl-CoA synthetase short-chain family member 2 (ACSS2), mRNA [NM_018677]	**2.411**
HMOX1	Homo sapiens heme oxygenase (decycling) 1 (HMOX1), mRNA [NM_002133]	**2.410**
ISG20	Homo sapiens interferon stimulated exonuclease gene 20kDa (ISG20), mRNA [NM_002201]	**2.409**
ARL4C	Homo sapiens ADP-ribosylation factor-like 4C (ARL4C), mRNA [NM_005737]	**2.386**
EEPD1	Homo sapiens endonuclease/exonuclease/phosphatase family domain containing 1 (EEPD1), mRNA [NM_030636]	**2.371**
HERC6	Homo sapiens hect domain and RLD 6 (HERC6), mRNA [NM_017912]	**2.345**
RENBP	Homo sapiens renin binding protein (RENBP), mRNA [NM_002910]	**2.343**
TRIML2	Homo sapiens tripartite motif family-like 2 (TRIML2), mRNA [NM_173553]	**2.343**
LOC643143	Homo sapiens hypothetical LOC643143 (LOC643143), mRNA [XM_931358]	**2.325**
F2RL2	Homo sapiens coagulation factor II (thrombin) receptor-like 2 (F2RL2), mRNA [NM_004101]	**2.308**
LOC100131576	Homo sapiens hypothetical protein LOC100131576 (LOC100131576), mRNA [XM_001723507]	**2.288**
NRCAM	Homo sapiens neuronal cell adhesion molecule (NRCAM), mRNA [NM_001037132]	**2.282**
FAIM3	Homo sapiens Fas apoptotic inhibitory molecule 3 (FAIM3), mRNA [NM_005449]	**2.278**
PCYT2	Homo sapiens phosphate cytidylyltransferase 2, ethanolamine (PCYT2), mRNA [NM_002861]	**2.278**
TNFRSF14	Homo sapiens tumor necrosis factor receptor superfamily, member 14 (herpesvirus entry mediator) (TNFRSF14), mRNA [NM_003820]	**2.261**

DHCR7	Homo sapiens 7-dehydrocholesterol reductase (DHCR7), mRNA [NM_001360]	**2.253**
FAM43A	Homo sapiens family with sequence similarity 43, member A (FAM43A), mRNA [NM_153690]	**2.246**
HS1BP3	Homo sapiens HCLS1 binding protein 3, mRNA (cDNA clone IMAGE:5207261), with apparent retained intron. [BC027947]	**2.233**
ADM	Homo sapiens adrenomedullin (ADM), mRNA [NM_001124]	**2.232**
CTSS	Homo sapiens cathepsin S (CTSS), mRNA [NM_004079]	**2.225**
MVD	Homo sapiens mevalonate (diphospho) decarboxylase (MVD), mRNA [NM_002461]	**2.221**
GSDMC	Homo sapiens gasdermin C (GSDMC), mRNA [NM_031415]	**2.209**
FGFBP1	Homo sapiens fibroblast growth factor binding protein 1 (FGFBP1), mRNA [NM_005130]	**2.204**
SDR16C5	Homo sapiens short chain dehydrogenase/reductase family 16C, member 5 (SDR16C5), mRNA [NM_138969]	**2.184**
RAB3IL1	Homo sapiens RAB3A interacting protein (rabin3)-like 1 (RAB3IL1), mRNA [NM_013401]	**2.181**
DDIT3	Homo sapiens DNA-damage-inducible transcript 3 (DDIT3), mRNA [NM_004083]	**2.174**
LOC344887	Homo sapiens mRNA; cDNA DKFZp686B14224 (from clone DKFZp686B14224). [BX640843]	**2.171**
PNMA3	Homo sapiens paraneoplastic antigen MA3 (PNMA3), mRNA [NM_013364]	**2.167**
RARRES3	Homo sapiens retinoic acid receptor responder (tazarotene induced) 3 (RARRES3), mRNA [NM_004585]	**2.167**
UAP1L1	Homo sapiens UDP-N-acteylglucosamine pyrophosphorylase 1-like 1 (UAP1L1), mRNA [NM_207309]	**2.148**
IL6R	Homo sapiens interleukin 6 receptor (IL6R), mRNA [NM_000565]	**2.146**
HLA-DMA	Homo sapiens major histocompatibility complex, class II, DM alpha (HLA-DMA), mRNA [NM_006120]	**2.128**
RSAD2	Homo sapiens radical S-adenosyl methionine domain containing 2 (RSAD2), mRNA [NM_080657]	**2.105**
S1PR1	Homo sapiens sphingosine-1-phosphate receptor 1 (S1PR1), mRNA [NM_001400]	**2.103**
EMR1	Homo sapiens egf-like module containing, mucin-like, hormone receptor-like 1 (EMR1), mRNA [NM_001974]	**2.095**
CPEB1	Homo sapiens cytoplasmic polyadenylation element binding protein 1 (CPEB1), mRNA [NM_030594]	**2.092**
LETM2	Homo sapiens leucine zipper-EF-hand containing transmembrane protein 2 (LETM2), mRNA [NM_144652]	**2.091**
MT1E	Homo sapiens unknown mRNA. [AF495759]	**2.087**
CTSL1	Homo sapiens cathepsin L1 (CTSL1), transcript variant 1, mRNA [NM_001912]	**2.085**
SLC30A1	Homo sapiens solute carrier family 30 (zinc transporter), member 1	**2.076**

	(SLC30A1), mRNA [NM_021194]	
IDI1	Homo sapiens isopentenyl-diphosphate delta isomerase 1 (IDI1), mRNA [NM_004508]	**2.074**
ARHGAP20	Homo sapiens Rho GTPase activating protein 20 (ARHGAP20), mRNA [NM_020809]	**2.070**
CTSD	Homo sapiens cathepsin D (CTSD), mRNA [NM_001909]	**2.064**
AQP9	Homo sapiens aquaporin 9 (AQP9), mRNA [NM_020980]	**2.062**
ELOVL3	Homo sapiens elongation of very long chain fatty acids (FEN1/Elo2, SUR4/Elo3, yeast)-like 3 (ELOVL3), mRNA [NM_152310]	**2.060**
TMEM156	Homo sapiens transmembrane protein 156 (TMEM156), mRNA [NM_024943]	**2.054**
LTF	Homo sapiens lactotransferrin (LTF), mRNA [NM_002343]	**2.051**
LOC283454	Homo sapiens cDNA FLJ37411 fis, clone BRAMY2028682. [AK094730]	**2.039**
FILIP1	Homo sapiens mRNA; cDNA DKFZp451B134 (from clone DKFZp451B134). [AL832009]	**2.033**
MACROD2	Homo sapiens MACRO domain containing 2 (MACROD2), mRNA [NM_080676]	**2.024**
UPP1	Homo sapiens uridine phosphorylase 1 (UPP1), mRNA [NM_181597]	**2.022**
NOV	Homo sapiens nephroblastoma overexpressed gene (NOV), mRNA [NM_002514]	**2.021**
HSPA6	Homo sapiens heat shock 70kDa protein 6 (HSP70B') (HSPA6), mRNA [NM_002155]	**2.015**
NCRNA00087	Homo sapiens non-protein coding RNA 87 (NCRNA00087), non-coding RNA [NR_024493]	**2.014**
APOL1	Homo sapiens apolipoprotein L, 1 (APOL1), mRNA [NM_145343]	**2.011**
SUNC1	Homo sapiens Sad1 and UNC84 domain containing 1 (SUNC1), mRNA [NM_001030019]	**2.010**
OLR1	Homo sapiens oxidized low density lipoprotein (lectin-like) receptor 1 (OLR1), mRNA [NM_002543]	**1.989**
C1R	Homo sapiens complement component 1 r subcomponent (C1R), mRNA [NM_001733]	**1.982**
PSG2	Homo sapiens pregnancy specific beta-1-glycoprotein 2 (PSG2), mRNA [NM_031246]	**1.977**
ANKRD29	Homo sapiens ankyrin repeat domain 29 (ANKRD29), mRNA [NM_173505]	**1.972**
HRK	Homo sapiens harakiri, BCL2 interacting protein (contains only BH3 domain) (HRK), mRNA [NM_003806]	**1.953**
NEU1	Homo sapiens sialidase 1 (lysosomal sialidase) (NEU1), mRNA [NM_000434]	**1.953**
SLAMF7	Homo sapiens SLAM family member 7 (SLAMF7), mRNA [NM_021181]	**1.953**
LSS	Homo sapiens lanosterol synthase (2,3-oxidosqualene-lanosterol cyclase) (LSS), mRNA [NM_001001438]	**1.953**
CSF2RA	Homo sapiens colony stimulating factor 2 receptor, alpha, low-affinity (CSF2RA), mRNA [NM_172247]	**1.949**
DMRT1	Homo sapiens doublesex and mab-3 related transcription factor 1 (DMRT1), mRNA [NM_021951]	**1.949**

STX11	Homo sapiens syntaxin 11 (STX11), mRNA [NM_003764]	**1.907**
FTL	Homo sapiens ferritin, light polypeptide (FTL), mRNA [NM_000146]	**1.904**
TOX3	Homo sapiens TOX high mobility group box family member 3 (TOX3), mRNA [NM_001080430]	**1.890**
MAFA	Homo sapiens v-maf musculoaponeurotic fibrosarcoma oncogene homolog A (MAFA), mRNA [NM_201589]	**1.886**
TRIM16L	Homo sapiens tripartite motif-containing 16-like (TRIM16L), mRNA [NM_001037330]	**1.885**
FABP3	Homo sapiens fatty acid binding protein 3, muscle and heart(FABP3), mRNA [NM_004102]	**1.879**
BEX2	Homo sapiens brain expressed X-linked 2 (BEX2), mRNA [NM_032621]	**1.875**
CYR61	Homo sapiens cysteine-rich, angiogenic inducer, 61 (CYR61), mRNA [NM_001554]	**1.874**
SLC22A18	Homo sapiens solute carrier family 22, member 18 (SLC22A18), mRNA [NM_183233]	**1.873**
FOLR3	Homo sapiens folate receptor 3 (gamma) (FOLR3), mRNA [NM_000804]	**1.871**
C1orf85	Homo sapiens chromosome 1 open reading frame 85 (C1orf85), mRNA [NM_144580]	**1.864**
HDAC9	Homo sapiens histone deacetylase 9 (HDAC9), mRNA [NM_058176]	**1.862**
HMCN1	Homo sapiens hemicentin 1 (HMCN1), mRNA [NM_031935]	**1.858**
ABLIM2	Homo sapiens actin binding LIM protein family, member 2 (ABLIM2), mRNA [NM_032432]	**1.854**
SLC9A9	Homo sapiens solute carrier family 9 (sodium/hydrogen exchanger), member 9 (SLC9A9), mRNA [NM_173653]	**1.843**
C6orf1	Homo sapiens chromosome 6 open reading frame 1 (C6orf1), mRNA [NM_178508]	**1.843**
S100P	Homo sapiens S100 calcium binding protein P (S100P), mRNA [NM_005980]	**1.841**
RAB27B	Homo sapiens RAB27B, member RAS oncogene family (RAB27B), mRNA [NM_004163]	**1.840**
IL6	Homo sapiens interleukin 6 (interferon, beta 2) (IL6), mRNA [NM_000600]	**1.839**
KIAA1486	Homo sapiens KIAA1486 protein (KIAA1486), mRNA [NM_020864]	**1.839**
ACP5	Homo sapiens acid phosphatase 5, tartrate resistant (ACP5), mRNA [NM_001611]	**1.838**
C14orf1	Homo sapiens chromosome 14 open reading frame 1 (C14orf1), mRNA [NM_007176]	**1.835**
DHRS9	Homo sapiens dehydrogenase/reductase (SDR family) member 9 (DHRS9), mRNA [NM_005771]	**1.830**
C10orf90	Homo sapiens chromosome 10 open reading frame 90 (C10orf90), mRNA [NM_001004298]	**1.827**
PRKAG2	Homo sapiens cDNA FLJ90194 fis, clone MAMMA1001284. [AK074675]	**1.827**
PSCA	Homo sapiens prostate stem cell antigen (PSCA), mRNA [NM_005672]	**1.824**
SLC22A15	Homo sapiens solute carrier family 22, member 15 (SLC22A15), mRNA [NM_018420]	**1.823**

PSAP	Homo sapiens prosaposin (PSAP), mRNA [NM_001042465]	**1.823**
SLC38A6	Homo sapiens solute carrier family 38, member 6 (SLC38A6), mRNA [NM_153811]	**1.822**
PNMA5	Homo sapiens paraneoplastic antigen like 5 (PNMA5), mRNA [NM_052926]	**1.821**
PTPRO	Homo sapiens protein tyrosine phosphatase, receptor type, O, mRNA (cDNA clone IMAGE:4610767). [BC035960]	**1.817**
TOM1	Homo sapiens target of myb1 (chicken) (TOM1), mRNA [NM_005488]	**1.817**
LOC387763	Protein Ag2 homolog [Source:UniProtKB/Swiss-Prot;Acc:Q7Z7L8] [ENST00000339446]	**1.812**
CYP1A1	Homo sapiens cytochrome P450, family 1, subfamily A, polypeptide 1 (CYP1A1), mRNA [NM_000499]	**1.810**
PLA2G7	Homo sapiens phospholipase A2, group VII (platelet-activating factor acetylhydrolase, plasma) (PLA2G7), mRNA [NM_005084]	**1.806**
ORAI3	Homo sapiens ORAI calcium release-activated calcium modulator 3 (ORAI3), mRNA [NM_152288]	**1.797**
FLJ31715	Homo sapiens cDNA FLJ31715 fis, clone NT2RI2006553. [AK056277]	**1.794**
PPP1R3B	Homo sapiens protein phosphatase1, regulatory (inhibitor) subunit 3B (PPP1R3B), mRNA [NM_024607]	**1.793**
HRASLS2	Homo sapiens HRAS-like suppressor 2 (HRASLS2), mRNA [NM_017878]	**1.792**
MCOLN2	Homo sapiens mucolipin 2 (MCOLN2), mRNA [NM_153259]	**1.791**
PLCG2	Homo sapiens phospholipase C, gamma 2 (PLCG2), mRNA [NM_002661]	**1.790**
GPR175	Homo sapiens G protein-coupled receptor 175 (GPR175), mRNA [NM_016372]	**1.789**
WFDC3	Homo sapiens WAP four-disulfide core domain 3 (WFDC3), mRNA [NM_080614]	**1.788**
SLC16A6	Homo sapiens solute carrier family 16, member 6 (SLC16A6), mRNA [NM_004694]	**1.782**
TBC1D2	Homo sapiens TBC1 domain family, member 2 (TBC1D2), mRNA [NM_018421]	**1.778**
CXCL2	Homo sapiens chemokine (C-X-C motif) ligand 2 (CXCL2), mRNA [NM_002089]	**1.773**
CARD17	Homo sapiens caspase recruitment domain family, member 17 (CARD17), mRNA [NM_001007232]	**1.771**
FBXO32	Homo sapiens F-box protein 32 (FBXO32), mRNA [NM_058229]	**1.763**
MGST1	Homo sapiens microsomal glutathione S-transferase 1 (MGST1), mRNA [NM_145791]	**1.763**
IGFN1	Homo sapiens immunoglobulin-like and fibronectin type III domain containing 1 (IGFN1), mRNA [NM_178275]	**1.759**
AMDHD2	Homo sapiens amidohydrolase domain containing 2 (AMDHD2), mRNA [NM_015944]	**1.757**
CES1	Homo sapiens carboxylesterase 1 (monocyte/macrophage serine esterase 1) (CES1), mRNA [NM_001266]	**1.756**
FDFT1	Homo sapiens farnesyl-diphosphate farnesyltransferase 1 (FDFT1), mRNA	**1.756**

	[NM_004462]	
PHEX	Homo sapiens phosphate regulating endopeptidase homolog, X-linked (PHEX), mRNA [NM_000444]	**1.748**
KYNU	Homo sapiens kynureninase (L-kynurenine hydrolase) (KYNU), mRNA [NM_003937]	**1.739**
BASP1	Homo sapiens brain abundant, membrane attached signal protein 1 (BASP1), mRNA [NM_006317]	**1.736**
CREG2	Homo sapiens cellular repressor of E1A-stimulated genes 2 (CREG2), mRNA [NM_153836]	**1.734**
KIAA0319	Homo sapiens KIAA0319 (KIAA0319), mRNA [NM_014809]	**1.733**
UBE2L6	Homo sapiens ubiquitin-conjugating enzyme E2L 6 (UBE2L6), mRNA [NM_198183]	**1.732**
CD68	Homo sapiens CD68 molecule (CD68), mRNA [NM_001251]	**1.731**
NPC2	Homo sapiens Niemann-Pick disease, type C2 (NPC2), mRNA [NM_006432]	**1.727**
MFGE8	Homo sapiens sperm surface protein hP47, partial. [Y11718]	**1.725**
LYG2	Homo sapiens lysozyme G-like 2 (LYG2), mRNA [NM_175735]	**1.724**
LCN2	Homo sapiens lipocalin 2 (LCN2), mRNA [NM_005564]	**1.722**
SLC12A8	Homo sapiens solute carrier family 12 (potassium/chloride transporters), member 8 (SLC12A8), mRNA [NM_024628]	**1.719**
C10orf58	Homo sapiens chromosome 10 open reading frame 58 (C10orf58), mRNA [NM_032333]	**1.716**
RELB	Homo sapiens v-rel reticuloendotheliosis viral oncogene homolog B (RELB), mRNA [NM_006509]	**1.714**
DUSP27	Homo sapiens dual specificity phosphatase 27 (putative) (DUSP27), mRNA [NM_001080426]	**1.713**
TMEM144	Homo sapiens transmembrane protein 144 (TMEM144), mRNA [NM_018342]	**1.713**
VAT1	Homo sapiens vesicle amine transport protein 1 homolog (T. californica) (VAT1), mRNA [NM_006373]	**1.708**
PTPRB	Homo sapiens protein tyrosine phosphatase, receptor type, B (PTPRB), transcript variant 2, mRNA [NM_002837]	**1.706**
ABHD4	Homo sapiens abhydrolase domain containing 4 (ABHD4), mRNA [NM_022060]	**1.705**
GNPDA1	Homo sapiens glucosamine-6-phosphate deaminase 1 (GNPDA1), mRNA [NM_005471]	**1.705**
MVK	Homo sapiens mevalonate kinase (MVK), mRNA [NM_000431]	**1.702**
LOC401317	Homo sapiens cDNA clone IMAGE:30398108. [BC087859]	**1.701**
C6orf223	Homo sapiens chromosome 6 open reading frame 223 (C6orf223), mRNA [NM_153246]	**1.701**
MAP3K8	Homo sapiens mitogen-activated protein kinase kinase kinase 8 (MAP3K8), mRNA [NM_005204]	**1.695**
POR	Homo sapiens P450 (cytochrome) oxidoreductase (POR), mRNA [NM_000941]	**1.691**
SOD2	Homo sapiens superoxide dismutase 2, mitochondrial, mRNA (cDNA clone	**1.687**

	MGC:21350 IMAGE:4184203). [BC016934]	
PDK4	Homo sapiens pyruvate dehydrogenase kinase, isozyme 4 (PDK4), mRNA [NM_002612]	**1.685**
GREB1	Homo sapiens GREB1 protein (GREB1), mRNA [NM_148903]	**1.684**
TLR3	Homo sapiens toll-like receptor 3 (TLR3), mRNA [NM_003265]	**1.683**
TRIM54	Homo sapiens tripartite motif-containing 54 (TRIM54), mRNA [NM_187841]	**1.680**
EBP	Homo sapiens emopamil binding protein (sterol isomerase) (EBP), mRNA [NM_006579]	**1.679**
CD177	Homo sapiens CD177 molecule (CD177), mRNA [NM_020406]	**1.677**
CYP4F8	Homo sapiens cytochrome P450, family 4, subfamily F, polypeptide 8 (CYP4F8), mRNA [NM_007253]	**1.676**
NOXO1	Homo sapiens NADPH oxidase organizer 1 (NOXO1), mRNA [NM_144603]	**1.676**
HMGCR	Homo sapiens 3-hydroxy-3-methylglutaryl-Coenzyme A reductase (HMGCR), mRNA [NM_000859]	**1.674**
C1orf201	Homo sapiens chromosome 1 open reading frame 201 (C1orf201), mRNA [NM_178122]	**1.672**
NPB	Homo sapiens cDNA clone IMAGE:5019903. [BC073815]	**1.671**
RCOR2	Homo sapiens REST corepressor 2 (RCOR2), mRNA [NM_173587]	**1.670**
GALNT13	Homo sapiens UDP-N-acetyl-alpha-D-galactosamine:polypeptide N-acetyl-galactosaminyltransferase 13 (GalNAc-T13), mRNA [NM_052917]	**1.666**
GPRC5C	Homo sapiens G protein-coupled receptor, family C, group 5, member C (GPRC5C), mRNA [NM_022036]	**1.659**
HERC5	Homo sapiens hect domain and RLD 5 (HERC5), mRNA [NM_016323]	**1.658**
LOC646241	Homo sapiens cDNA clone IMAGE:5201079. [BC028204]	**1.654**
RHOB	Homo sapiens ras homolog gene family, member B (RHOB), mRNA [NM_004040]	**1.654**
G6PD	Homo sapiens glucose-6-phosphate dehydrogenase (G6PD), mRNA [NM_000402]	**1.648**
CPN2	Homo sapiens carboxypeptidase N, polypeptide 2 (CPN2), mRNA [NM_001080513]	**1.646**
CSAG1	Homo sapiens chondrosarcoma associated gene 1 (CSAG1), transcript variant a, mRNA [NM_153478]	**1.645**
GP9	Homo sapiens glycoprotein IX (platelet) (GP9), mRNA [NM_000174]	**1.639**
CXCL3	Homo sapiens chemokine (C-X-C motif) ligand 3 (CXCL3), mRNA [NM_002090]	**1.638**
MAGEB6	Homo sapiens melanoma antigen family B, 6 (MAGEB6), mRNA [NM_173523]	**1.637**
ANO2	Homo sapiens anoctamin 2 (ANO2), mRNA [NM_020373]	**1.636**
ITGA7	Homo sapiens integrin, alpha 7 (ITGA7), mRNA [NM_002206]	**1.636**
TUBAL3	Homo sapiens tubulin, alpha-like 3 (TUBAL3), mRNA [NM_024803]	**1.636**
TPK1	Homo sapiens thiamin pyrophosphokinase 1 (TPK1), mRNA [NM_022445]	**1.631**
SLC17A5	Homo sapiens solute carrier family 17 (anion/sugar transporter), member 5 (SLC17A5), mRNA [NM_012434]	**1.622**

CPVL	Homo sapiens carboxypeptidase, vitellogenic-like (CPVL),mRNA [NM_019029]	**1.612**
QPRT	Homo sapiens quinolinate phosphoribosyltransferase (QPRT), mRNA [NM_014298]	**1.601**
TRPM3	Homo sapiens transient receptor potential cation channel, subfamily M, member 3 (TRPM3), mRNA [NM_001007471]	**1.601**
FADS2	Homo sapiens fatty acid desaturase 2 (FADS2), mRNA [NM_004265]	**1.599**
SH3TC1	Homo sapiens SH3 domain and tetratricopeptide repeats 1 (SH3TC1), mRNA [NM_018986]	**1.599**
PLA2G16	Homo sapiens phospholipase A2, group XVI (PLA2G16), mRNA [NM_007069]	**1.597**
STARD4	Homo sapiens StAR-related lipid transfer (START) domain containing 4 (STARD4), mRNA [NM_139164]	**1.595**
FLCN	Homo sapiens mRNA; cDNA DKFZp547A118 (from clone DKFZp547A118). [AL831885]	**1.594**
RIMKLA	Homo sapiens ribosomal modification protein rimK-like family member A (RIMKLA), mRNA [NM_173642]	**1.591**
GRAMD3	Homo sapiens GRAM domain containing 3 (GRAMD3), mRNA [NM_023927]	**1.582**
GEM	Homo sapiens GTP binding protein overexpressed in skeletal muscle (GEM), mRNA [NM_005261]	**1.571**
KIR2DL2	Homo sapiens killer cell immunoglobulin-like receptor, two domains, long cytoplasmic tail, 2 (KIR2DL2), mRNA [NM_014219]	**1.571**
CD36	Homo sapiens CD36 molecule (thrombospondin receptor) (CD36), mRNA [NM_001001547]	**1.570**
TEC	Homo sapiens tec protein tyrosine kinase (TEC), mRNA [NM_003215]	**1.567**
INPPL1	Homo sapiens inositol polyphosphate phosphatase-like 1 (INPPL1), mRNA [NM_001567]	**1.560**
FOXQ1	Homo sapiens forkhead box Q1 (FOXQ1), mRNA [NM_033260]	**1.559**
EDNRA	Homo sapiens endothelin receptor type A (EDNRA), mRNA [NM_001957]	**1.558**
FNIP2	Homo sapiens folliculin interacting protein 2 (FNIP2), mRNA [NM_020840]	**1.556**
NAV3	Homo sapiens neuron navigator 3 (NAV3), mRNA [NM_014903]	**1.554**
ATP8B1	Homo sapiens ATPase, class I, type 8B, member 1 (ATP8B1), mRNA [NM_005603]	**1.552**
NUPR1	Homo sapiens nuclear protein 1 (NUPR1), mRNA [NM_001042483]	**1.552**
DOK7	Homo sapiens docking protein 7 (DOK7), mRNA [NM_173660]	**1.550**
METTL7B	Homo sapiens methyltransferase like 7B (METTL7B), mRNA [NM_152637]	**1.549**
IFI35	Homo sapiens interferon-induced protein 35 (IFI35), mRNA [NM_005533]	**1.547**
TMEM116	Homo sapiens transmembrane protein 116 (TMEM116), mRNA [NM_138341]	**1.547**
SLC12A7	Homo sapiens solute carrier family 12 (potassium/chloride transporters), member 7 (SLC12A7), mRNA [NM_006598]	**1.547**
ASB5	Homo sapiens ankyrin repeat and SOCS box-containing 5 (ASB5), mRNA [NM_080874]	**1.543**

IFI6	Homo sapiens interferon, alpha-inducible protein 6 (IFI6), mRNA [NM_022873]	**1.543**
PAG1	Homo sapiens phosphoprotein associated with glycosphingolipid microdomains 1 (PAG1), mRNA [NM_018440]	**1.543**
CPA4	Homo sapiens carboxypeptidase A4 (CPA4), mRNA [NM_016352]	**1.542**
AGTRAP	Homo sapiens angiotensin II receptor-associated protein (AGTRAP), mRNA [NM_001040196]	**1.538**
SLC2A5	Homo sapiens solute carrier family 2 (facilitated glucose/fructose transporter), member 5, mRNA (cDNA clone IMAGE:4520699). [BC035878]	**1.536**
VCX3A	Homo sapiens variable charge, X-linked 3A (VCX3A), mRNA [NM_016379]	**1.536**
APOL6	Homo sapiens apolipoprotein L, 6 (APOL6), mRNA [NM_030641]	**1.533**
NDRG4	Homo sapiens NDRG family member 4 (NDRG4), mRNA [NM_022910]	**1.532**
APOE	Homo sapiens apolipoprotein E (APOE), mRNA [NM_000041]	**1.531**
STXBP1	Homo sapiens syntaxin binding protein 1 (STXBP1), mRNA [NM_001032221]	**1.530**
JPH1	Homo sapiens junctophilin 1 (JPH1), mRNA [NM_020647]	**1.524**
ABCC2	Homo sapiens ATP-binding cassette, sub-family C (CFTR/MRP), member 2 (ABCC2), mRNA [NM_000392]	**1.524**
SAMD9L	Homo sapiens sterile alpha motif domain containing 9-like (SAMD9L), mRNA [NM_152703]	**1.523**
IL33	Homo sapiens interleukin 33 (IL33), mRNA [NM_033439]	**1.522**
ANXA10	Homo sapiens annexin A10 (ANXA10), mRNA [NM_007193]	**1.520**
BHLHE41	Homo sapiens basic helix-loop-helix domain containing, class B, 3 (BHLHB3), mRNA [NM_030762]	**1.520**
CYP51A1	Homo sapiens cytochrome P450, family 51, subfamily A, polypeptide 1 (CYP51A1), mRNA [NM_000786]	**1.520**
CXCL16	Homo sapiens chemokine (C-X-C motif) ligand 16 (CXCL16), mRNA [NM_022059]	**1.519**
GALNT12	Homo sapiens UDP-N-acetyl-alpha-D-galactosamine:polypeptide N-acetylgalactosaminyltransferase 12 (GalNAc-T12) (GALNT12), mRNA [NM_024642]	**1.517**
ATP6V0A4	Homo sapiens ATPase, H+ transporting, lysosomal V0 subunit a4 (ATP6V0A4), mRNA [NM_020632]	**1.515**
ABCG2	Homo sapiens ATP-binding cassette, sub-family G (WHITE), member 2 (ABCG2), mRNA [NM_004827]	**1.513**
RHEBL1	Homo sapiens Ras homolog enriched in brain like 1 (RHEBL1), mRNA [NM_144593]	**1.510**
APH1B	Homo sapiens anterior pharynx defective 1 homolog B (C. elegans) (APH1B), mRNA [NM_031301]	**1.505**
ECM2	Homo sapiens extracellular matrix protein 2, female organ and adipocyte specific (ECM2), mRNA [NM_001393]	**1.504**
TRIB3	Homo sapiens tribbles homolog 3 (Drosophila) (TRIB3), mRNA [NM_021158]	**1.504**

ANKRD1	Homo sapiens ankyrin repeat domain 1 (cardiac muscle) (ANKRD1), mRNA [NM_014391]	**1.503**
C1orf88	Homo sapiens chromosome 1 open reading frame 88 (C1orf88), mRNA [NM_181643]	**1.503**
CDKN1A	Homo sapiens cyclin-dependent kinase inhibitor 1A (p21, Cip1) (CDKN1A), mRNA [NM_000389]	**1.502**
CREG1	Homo sapiens cellular repressor of E1A-stimulated genes 1 (CREG1), mRNA [NM_003851]	**1.502**
LNX1	E3 ubiquitin-protein ligase LNX (EC 6.3.2.-)(Numb-binding protein 1)(Ligand of Numb-protein X 1) [Source:UniProtKB/Swiss-Prot;Acc:Q8TBB1] [ENST00000381380]	**1.501**
BLVRB	Homo sapiens biliverdin reductase B (flavin reductase (NADPH)) (BLVRB), mRNA [NM_000713]	**1.500**
ADAM23	Homo sapiens ADAM metallopeptidase domain 23 (ADAM23), mRNA [NM_003812]	**1.498**
CLCN7	Homo sapiens chloride channel 7 (CLCN7), mRNA [NM_001287]	**1.492**
PGCP	Homo sapiens plasma glutamate carboxypeptidase (PGCP), mRNA [NM_016134]	**1.492**
NDRG1	Homo sapiens N-myc downstream regulated 1 (NDRG1), mRNA [NM_006096]	**1.491**
PIR	Homo sapiens pirin (iron-binding nuclear protein) (PIR), mRNA [NM_003662]	**1.489**
BAALC	Homo sapiens brain and acute leukemia, cytoplasmic (BAALC), mRNA [NM_001024372]	**1.488**
TNFSF9	Homo sapiens tumor necrosis factor (ligand) superfamily, member 9 (TNFSF9), mRNA [NM_003811]	**1.488**
AKR1B10	Homo sapiens aldo-keto reductase family 1, member B10 (aldose reductase) (AKR1B10), mRNA [NM_020299]	**1.488**
TM4SF1	Homo sapiens transmembrane 4 L six family member 1 (TM4SF1), mRNA [NM_014220]	**1.486**
SCAMP5	Homo sapiens secretory carrier membrane protein 5 (SCAMP5), mRNA [NM_138967]	**1.485**
TCN2	Homo sapiens transcobalamin II; macrocytic anemia (TCN2), mRNA [NM_000355]	**1.483**
PSG11	Homo sapiens pregnancy specific beta-1-glycoprotein 11 (PSG11), mRNA [NM_002785]	**1.479**
ACSL1	Homo sapiens acyl-CoA synthetase long-chain family member 1 (ACSL1), mRNA [NM_001995]	**1.476**
GRINA	Homo sapiens glutamate receptor, ionotropic, N-methyl D-aspartate-associated protein 1 (glutamate binding) (GRINA), mRNA [NM_000837]	**1.476**
FAM134B	Homo sapiens family with sequence similarity 134, member B (FAM134B), mRNA [NM_001034850]	**1.475**
SLC25A1	Homo sapiens solute carrier family 25 (mitochondrial carrier; citrate transporter), member 1 (SLC25A1), mRNA [NM_005984]	**1.475**

THAP8	Homo sapiens THAP domain containing 8 (THAP8), mRNA [NM_152658]	**1.472**
GPR183	Homo sapiens G protein-coupled receptor 183 (GPR183), mRNA [NM_004951]	**1.470**
KIAA0513	Homo sapiens KIAA0513 (KIAA0513), mRNA [NM_014732]	**1.470**
FXYD3	Homo sapiens FXYD domain containing ion transport regulator 3 (FXYD3), mRNA [NM_005971]	**1.468**
B4GALNT1	Homo sapiens beta-1,4-N-acetyl-galactosaminyl transferase 1 (B4GALNT1), mRNA [NM_001478]	**1.466**
SV2A	Homo sapiens synaptic vesicle glycoprotein 2A (SV2A), mRNA [NM_014849]	**1.464**
NKX2-8	Homo sapiens NK2 homeobox 8 (NKX2-8), mRNA [NM_014360]	**1.463**
LCP1	Homo sapiens lymphocyte cytosolic protein 1 (L-plastin) (LCP1), mRNA [NM_002298]	**1.457**
NFKB2	Homo sapiens nuclear factor of kappa light polypeptide gene enhancer in B-cells 2 (p49/p100) (NFKB2), mRNA [NM_001077493]	**1.454**
RRAGC	Homo sapiens Ras-related GTP binding C (RRAGC), mRNA [NM_022157]	**1.453**
CD180	Homo sapiens CD180 molecule (CD180), mRNA [NM_005582]	**1.451**
LMCD1	Homo sapiens LIM and cysteine-rich domains 1 (LMCD1), mRNA [NM_014583]	**1.450**
NFIL3	Homo sapiens nuclear factor, interleukin 3 regulated (NFIL3), mRNA [NM_005384]	**1.449**
PTGS2	Homo sapiens prostaglandin-endoperoxide synthase 2 (prostaglandin G/H synthase and cyclooxygenase) (PTGS2), mRNA [NM_000963]	**1.449**
IGFBP1	Homo sapiens insulin-like growth factor binding protein 1 (IGFBP1), mRNA [NM_000596]	**1.447**
GSR	Homo sapiens glutathione reductase (GSR), mRNA [NM_000637]	**1.444**
PMAIP1	Homo sapiens phorbol-12-myristate-13-acetate-induced protein 1 (PMAIP1), mRNA [NM_021127]	**1.444**
HECW2	Homo sapiens HECT, C2 and WW domain containing E3 ubiquitin protein ligase 2 (HECW2), mRNA [NM_020760]	**1.444**
CLIC2	Homo sapiens chloride intracellular channel 2 (CLIC2), mRNA [NM_001289]	**1.443**
KIAA1128	Homo sapiens KIAA1128 (KIAA1128), mRNA [NM_018999]	**1.442**
TRAPPC6A	Homo sapiens trafficking protein particle complex 6A (TRAPPC6A), mRNA [NM_024108]	**1.442**
ASAH1	Homo sapiens cDNA: FLJ21558 fis, clone COL06372. [AK025211]	**1.439**
MEGF6	Homo sapiens multiple EGF-like-domains 6 (MEGF6), mRNA [NM_001409]	**1.438**
SRPX2	Homo sapiens sushi-repeat-containing protein, X-linked 2 (SRPX2), mRNA [NM_014467]	**1.437**
EREG	Homo sapiens epiregulin (EREG), mRNA [NM_001432]	**1.434**
UGT1A6	Homo sapiens UDP glucuronosyltransferase 1 family, polypeptide A6 (UGT1A6), mRNA [NM_001072]	**1.434**
DDX58	Homo sapiens DEAD (Asp-Glu-Ala-Asp) box polypeptide 58 (DDX58), mRNA [NM_014314]	**1.429**

GFI1B	Homo sapiens growth factor independent 1B transcription repressor (GFI1B), mRNA [NM_004188]	**1.429**
ZNF467	Homo sapiens zinc finger protein 467 (ZNF467), mRNA [NM_207336]	**1.429**
DKK1	Homo sapiens dickkopf homolog 1 (Xenopus laevis) (DKK1), mRNA [NM_012242]	**1.425**
FDPSL2A	Homo sapiens MGC44478 (FDPSL2A), non-coding RNA [NR_003262]	**1.425**
TMEM97	Homo sapiens transmembrane protein 97 (TMEM97), mRNA [NM_014573]	**1.422**
SNAI2	Homo sapiens snail homolog 2 (Drosophila) (SNAI2), mRNA [NM_003068]	**1.419**
HBE1	Homo sapiens hemoglobin, epsilon 1 (HBE1), mRNA [NM_005330]	**1.419**
SDSL	Homo sapiens serine dehydratase-like (SDSL), mRNA [NM_138432]	**1.418**
SLC7A2	Homo sapiens solute carrier family 7 (cationic amino acid transporter, y+ system), member 2 (SLC7A2), mRNA [NM_003046]	**1.418**
MVP	Homo sapiens major vault protein (MVP), mRNA [NM_017458]	**1.417**
FUT1	Homo sapiens fucosyltransferase 1 (galactoside 2-alpha-L-fucosyltransferase, H blood group) (FUT1), mRNA [NM_000148]	**1.416**
SCARF2	Homo sapiens scavenger receptor class F, member 2 (SCARF2), mRNA [NM_153334]	**1.415**
ALDH1B1	Homo sapiens aldehyde dehydrogenase 1 family, member B1 (ALDH1B1), mRNA [NM_000692]	**1.411**
IL1R1	Homo sapiens interleukin 1 receptor, type I (IL1R1), mRNA [NM_000877]	**1.409**
FAM50B	Homo sapiens family with sequence similarity 50, member B (FAM50B), mRNA [NM_012135]	**1.406**
FADS1	Homo sapiens fatty acid desaturase 1 (FADS1), mRNA [NM_013402]	**1.404**
KLF7	Homo sapiens Kruppel-like factor 7 (ubiquitous) (KLF7), mRNA [NM_003709]	**1.403**
MAOB	Homo sapiens monoamine oxidase B (MAOB), mRNA [NM_000898]	**1.403**
TYRP1	Homo sapiens tyrosinase-related protein 1 (TYRP1), mRNA [NM_000550]	**1.403**
SCD	Homo sapiens stearoyl-CoA desaturase (delta-9-desaturase) (SCD), mRNA [NM_005063]	**1.402**
CLIP2	Homo sapiens CAP-GLY domain containing linker protein 2 (CLIP2), mRNA [NM_003388]	**1.399**
VCX2	Homo sapiens variable charge, X-linked 2 (VCX2), mRNA [NM_016378]	**1.399**
SCPEP1	Homo sapiens serine carboxypeptidase 1 (SCPEP1), mRNA [NM_021626]	**1.398**
FCGR2A	Homo sapiens Fc fragment of IgG, low affinity IIa, receptor (CD32) (FCGR2A), mRNA [NM_021642]	**1.394**
HTR1D	Homo sapiens 5-hydroxytryptamine (serotonin) receptor 1D (HTR1D), mRNA [NM_000864]	**1.394**
ITPRIP	Homo sapiens inositol 1,4,5-triphosphate receptor interacting protein (ITPRIP), mRNA [NM_033397]	**1.394**
DYNC1I1	Homo sapiens dynein, cytoplasmic 1, intermediate chain 1 (DYNC1I1), mRNA [NM_004411]	**1.393**
HSD17B7	Homo sapiens hydroxysteroid (17-beta) dehydrogenase 7 (HSD17B7), mRNA [NM_016371]	**1.393**

SRXN1	Homo sapiens sulfiredoxin 1 homolog (S. cerevisiae) (SRXN1), mRNA [NM_080725]	**1.392**
DISP2	Homo sapiens dispatched homolog 2 (Drosophila) (DISP2), mRNA [NM_033510]	**1.391**
HOXA3	Homo sapiens homeobox A3 (HOXA3), mRNA [NM_153631]	**1.389**
CARD16	Homo sapiens caspase recruitment domain family, member 16 (CARD16), mRNA [NM_001017534]	**1.387**
SERINC2	Homo sapiens serine incorporator 2 (SERINC2), mRNA [NM_178865]	**1.387**
SH3TC2	Homo sapiens SH3 domain and tetratricopeptide repeats 2 (SH3TC2), mRNA [NM_024577]	**1.383**
PGD	Homo sapiens phosphogluconate dehydrogenase (PGD), mRNA [NM_002631]	**1.381**
NPC1	Homo sapiens Niemann-Pick disease, type C1 (NPC1), mRNA [NM_000271]	**1.379**
GPR143	Homo sapiens G protein-coupled receptor 143 (GPR143), mRNA [NM_000273]	**1.376**
LOC100129113	Homo sapiens cDNA FLJ37158 fis, clone BRACE2026293. [AK094477]	**1.375**
TMPRSS5	Homo sapiens transmembrane protease, serine 5 (TMPRSS5), mRNA [NM_030770]	**1.375**
PHLDA3	Homo sapiens pleckstrin homology-like domain, family A, member 3 (PHLDA3), mRNA [NM_012396]	**1.373**
CAMTA2	Homo sapiens calmodulin binding transcription activator 2 (CAMTA2), mRNA [NM_015099]	**1.370**
FAM167B	Homo sapiens family with sequence similarity 167, member B (FAM167B), mRNA [NM_032648]	**1.369**
RUNX3	Homo sapiens runt-related transcription factor 3 (RUNX3), mRNA [NM_001031680]	**1.369**
AGPAT2	Homo sapiens 1-acylglycerol-3-phosphate O-acyltransferase 2 (lysophosphatidic acid acyltransferase, beta) (AGPAT2), mRNA [NM_006412]	**1.368**
C5orf51	Homo sapiens chromosome 5 open reading frame 51 (C5orf51), mRNA [NM_175921]	**1.367**
OTUD1	Homo sapiens mRNA, clone: TH020D07. [AB188491]	**1.363**
CLDN4	Homo sapiens claudin 4 (CLDN4), mRNA [NM_001305]	**1.362**
LOC133874	Homo sapiens hypothetical gene LOC133874 (LOC133874), mRNA [NM_001102609]	**1.361**
RP5-1022P6.2	Homo sapiens hypothetical protein KIAA1434 (RP5-1022P6.2), mRNA [NM_019593]	**1.361**
FMNL2	Homo sapiens formin-like 2 (FMNL2), mRNA [NM_052905]	**1.358**
JAKMIP3	Homo sapiens janus kinase and microtubule interacting protein 3 (JAKMIP3), mRNA [NM_001105521]	**1.358**
PPM2C	Homo sapiens protein phosphatase 2C, magnesium-dependent, catalytic subunit (PPM2C), nuclear gene encoding mitochondrial protein, mRNA [NM_018444]	**1.357**
DUSP4	Homo sapiens dual specificity phosphatase 4 (DUSP4), mRNA [NM_001394]	**1.352**
ANPEP	Homo sapiens alanyl (membrane) aminopeptidase (ANPEP), mRNA	**1.351**

	[NM_001150]	
SLC16A14	Homo sapiens solute carrier family 16, member 14 (monocarboxylic acid transporter 14) (SLC16A14), mRNA [NM_152527]	**1.351**
MAL2	Homo sapiens mal, T-cell differentiation protein 2 (MAL2), mRNA [NM_052886]	**1.349**
CDA	Homo sapiens cytidine deaminase (CDA), mRNA [NM_001785]	**1.347**
TNIP1	Homo sapiens TNFAIP3 interacting protein 1 (TNIP1), mRNA [NM_006058]	**1.346**
ATP6V1B2	Homo sapiens ATPase, H+ transporting, lysosomal 56/58kDa, V1 subunit B2 (ATP6V1B2), mRNA [NM_001693]	**1.345**
THRB	Homo sapiens thyroid hormone receptor, beta (erythroblastic leukemia viral (v-erb-a) oncogene homolog 2, avian) (THRB), mRNA [NM_000461]	**1.344**
OPTN	Homo sapiens optineurin (OPTN), mRNA [NM_001008211]	**1.343**
SLC38A7	Homo sapiens solute carrier family 38, member 7 (SLC38A7), mRNA [NM_018231]	**1.343**
DUSP13	Homo sapiens dual specificity phosphatase 13 (DUSP13), mRNA [NM_001007271]	**1.340**
hCG_1811732	Homo sapiens hypothetical LOC151534 (LOC151534), non-coding RNA [NR_024606]	**1.340**
RRAD	Homo sapiens Ras-related associated with diabetes (RRAD), mRNA [NM_004165]	**1.338**
AIM1L	Homo sapiens absent in melanoma 1-like (AIM1L), mRNA [NM_001039775]	**1.335**
CAPSL	Homo sapiens calcyphosine-like (CAPSL), mRNA [NM_144647]	**1.334**
MAPKAPK3	Homo sapiens mitogen-activated protein kinase-activated protein kinase 3 (MAPKAPK3), mRNA [NM_004635]	**1.334**
FOLR2	Homo sapiens folate receptor 2 (fetal) (FOLR2), mRNA [NM_000803]	**1.330**
PLB1	Homo sapiens phospholipase B1 (PLB1), mRNA [NM_153021]	**1.330**
NUDT14	Homo sapiens nudix (nucleoside diphosphate linked moiety X)-type motif 14 (NUDT14), mRNA [NM_177533]	**1.330**
C19orf66	Homo sapiens chromosome 19 open reading frame 66 (C19orf66), mRNA [NM_018381]	**1.328**
PPAP2C	Homo sapiens phosphatidic acid phosphatase type 2C (PPAP2C), mRNA [NM_177543]	**1.327**
GDPD3	Homo sapiens glycerophosphodiester phosphodiesterase domain containing 3 (GDPD3), mRNA [NM_024307]	**1.322**
SLC2A6	Homo sapiens solute carrier family 2 (facilitated glucose transporter), member 6 (SLC2A6), mRNA [NM_017585]	**1.321**
IL1R2	Homo sapiens interleukin 1 receptor, type II (IL1R2), mRNA [NM_004633]	**1.320**
GADD45A	Homo sapiens growth arrest and DNA-damage-inducible, alpha (GADD45A), mRNA [NM_001924]	**1.318**
KIFC3	Homo sapiens kinesin family member C3 (KIFC3), mRNA [NM_005550]	**1.318**
TSPAN8	Homo sapiens tetraspanin 8 (TSPAN8), mRNA [NM_004616]	**1.318**
USP54	Homo sapiens ubiquitin specific peptidase 54 (USP54), mRNA [NM_152586]	**1.318**
ALDOA	Homo sapiens aldolase A, fructose-bisphosphate (ALDOA), mRNA	**1.317**

	[NM_184041]	
BIRC3	Homo sapiens baculoviral IAP repeat-containing 3 (BIRC3), mRNA [NM_001165]	**1.316**
VLDLR	Homo sapiens very low density lipoprotein receptor (VLDLR), mRNA [NM_003383]	**1.316**
EGF	Homo sapiens epidermal growth factor (beta-urogastrone) (EGF), mRNA [NM_001963]	**1.315**
RNASEK	Homo sapiens ribonuclease, RNase K (RNASEK), mRNA [NM_001004333]	**1.313**
ATP6V0D1	Homo sapiens ATPase, H+ transporting, lysosomal 38kDa, V0 subunit d1 (ATP6V0D1), mRNA [NM_004691]	**1.311**
NCRNA00166	Homo sapiens cDNA FLJ36032 fis, clone TESTI2017069. [AK093351]	**1.310**
SYTL2	Homo sapiens synaptotagmin-like 2 (SYTL2), mRNA [NM_032943]	**1.309**
CYP4F12	Homo sapiens cytochrome P450, family 4, subfamily F, polypeptide 12 (CYP4F12), mRNA [NM_023944]	**1.308**
C15orf27	Homo sapiens chromosome 15 open reading frame 27 (C15orf27), mRNA [NM_152335]	**1.305**
PAEP	Homo sapiens progestagen-associated endometrial protein (PAEP), mRNA [NM_002571]	**1.305**
HHAT	Homo sapiens hedgehog acyltransferase (HHAT), mRNA [NM_018194]	**1.304**
TAPBPL	Homo sapiens TAP binding protein-like (TAPBPL), mRNA [NM_018009]	**1.303**
PLEKHG1	Homo sapiens pleckstrin homology domain containing, family G (with RhoGef domain) member 1 (PLEKHG1), mRNA [NM_001029884]	**1.301**
PROS1	Homo sapiens protein S (alpha) (PROS1), mRNA [NM_000313]	**1.299**
EPS8	Homo sapiens epidermal growth factor receptor pathway substrate 8 (EPS8), mRNA [NM_004447]	**1.298**
ZFYVE1	Homo sapiens zinc finger, FYVE domain containing 1 (ZFYVE1), mRNA [NM_021260]	**1.298**
TNNC1	Homo sapiens troponin C type 1 (slow) (TNNC1), mRNA [NM_003280]	**1.297**
ATP6V0C	Homo sapiens ATPase, H+ transporting, lysosomal 16kDa, V0 subunit c (ATP6V0C), mRNA [NM_001694]	**1.297**
MAFF	Homo sapiens v-maf musculoaponeurotic fibrosarcoma oncogene homolog F (avian) (MAFF), mRNA [NM_012323]	**1.296**
SNX8	Homo sapiens sorting nexin 8 (SNX8), mRNA [NM_013321]	**1.296**
ARMC9	Homo sapiens armadillo repeat containing 9 (ARMC9), mRNA [NM_025139]	**1.293**
HSD17B7P2	Homo sapiens 17-beta-hydroxysteroid dehydrogenase type VII isoform mRNA. [AF165514]	**1.293**
ISG15	Homo sapiens ISG15 ubiquitin-like modifier (ISG15), mRNA [NM_005101]	**1.292**
DMKN	Homo sapiens dermokine (DMKN), mRNA [NM_001035516]	**1.291**
HEXB	Homo sapiens hexosaminidase B (beta polypeptide) (HEXB), mRNA [NM_000521]	**1.291**
SNTB1	Homo sapiens syntrophin, beta 1 (dystrophin-associated protein A1) (SNTB1), mRNA [NM_021021]	**1.291**
CPA3	Homo sapiens carboxypeptidase A3 (mast cell) (CPA3), mRNA [NM_001870]	**1.289**

PIM3	Homo sapiens pim-3 oncogene (PIM3), mRNA [NM_001001852]	**1.287**
FTH1	Homo sapiens ferritin, heavy polypeptide 1 (FTH1), mRNA [NM_002032]	**1.286**
DYSF	Homo sapiens dysferlin, limb girdle muscular dystrophy 2B (autosomal recessive) (DYSF), mRNA [NM_003494]	**1.283**
MCOLN1	Homo sapiens mucolipin 1 (MCOLN1), mRNA [NM_020533]	**1.282**
ADRBK2	Homo sapiens adrenergic, beta, receptor kinase 2 (ADRBK2), mRNA [NM_005160]	**1.279**
CTNS	Homo sapiens cystinosis, nephropathic (CTNS), mRNA [NM_004937]	**1.277**
RPS27L	Homo sapiens ribosomal protein S27-like (RPS27L), mRNA [NM_015920]	**1.275**
SGIP1	Homo sapiens SH3-domain GRB2-like (endophilin) interacting protein 1 (SGIP1), mRNA [NM_032291]	**1.273**
COMMD7	Homo sapiens COMM domain containing 7 (COMMD7), mRNA [NM_053041]	**1.273**
MBD5	Homo sapiens methyl-CpG binding domain protein 5, mRNA (cDNA clone IMAGE:3996924). [BC014534]	**1.271**
HES6	Homo sapiens hairy and enhancer of split 6 (Drosophila) (HES6), mRNA [NM_018645]	**1.270**
CMBL	Homo sapiens carboxymethylenebutenolidase homolog (Pseudomonas) (CMBL), mRNA [NM_138809]	**1.269**
TXNRD1	Homo sapiens thioredoxin reductase 1 (TXNRD1), mRNA [NM_003330]	**1.269**
GAB2	Homo sapiens GRB2-associated binding protein 2 (GAB2), mRNA [NM_012296]	**1.268**
GPR115	Homo sapiens G protein-coupled receptor 115 (GPR115), mRNA [NM_153838]	**1.267**
ATF3	Homo sapiens activating transcription factor 3 (ATF3), mRNA [NM_001040619]	**1.266**
GPR64	Homo sapiens G protein-coupled receptor 64 (GPR64), mRNA [NM_001079858]	**1.266**
CSTB	Homo sapiens cystatin B (stefin B) (CSTB), mRNA [NM_000100]	**1.263**
NUAK2	Homo sapiens NUAK family, SNF1-like kinase, 2 (NUAK2), mRNA [NM_030952]	**1.263**
C19orf20	Homo sapiens chromosome 19 open reading frame 20 (C19orf20), mRNA [NM_033513]	**1.262**
CRYBB3	Homo sapiens crystallin, beta B3 (CRYBB3), mRNA [NM_004076]	**1.262**
GGT8P	Homo sapiens gamma-glutamyltransferase 8 pseudogene (GGT8P), non-coding RNA [NR_003503]	**1.261**
GMPR	Homo sapiens guanosine monophosphate reductase (GMPR), mRNA [NM_006877]	**1.261**
RBPMS	Homo sapiens RNA binding protein with multiple splicing (RBPMS), mRNA [NM_001008712]	**1.260**
PLEKHF1	Homo sapiens pleckstrin homology domain containing, family F (with FYVE domain) member 1 (PLEKHF1), mRNA [NM_024310]	**1.258**
GPR160	Homo sapiens G protein-coupled receptor 160 (GPR160), mRNA	**1.258**

	[NM_014373]	
ACSF2	Homo sapiens acyl-CoA synthetase family member 2 (ACSF2), mRNA [NM_025149]	**1.256**
GREM1	Homo sapiens gremlin 1, cysteine knot superfamily, homolog (Xenopus laevis) (GREM1), mRNA [NM_013372]	**1.256**
OGDHL	Homo sapiens oxoglutarate dehydrogenase-like (OGDHL), mRNA [NM_018245]	**1.256**
TAP2	Homo sapiens transporter 2, ATP-binding cassette, sub-family B (MDR/TAP) (TAP2), transcript variant 1, mRNA [NM_000544]	**1.255**
C22orf25	Homo sapiens chromosome 22 open reading frame 25 (C22orf25), mRNA [NM_152906]	**1.254**
LOC439951	Homo sapiens hypothetical LOC439951, mRNA (cDNA clone MGC:33041 IMAGE:4838780), complete cds. [BC037281]	**1.254**
LRRC61	Homo sapiens leucine rich repeat containing 61 (LRRC61), mRNA [NM_023942]	**1.253**
GLIS3	Homo sapiens GLIS family zinc finger 3 (GLIS3), mRNA [NM_001042413]	**1.252**
ACLY	Human ATP:citrate lyase mRNA, complete cds. [U18197]	**1.247**
ALPL	Homo sapiens alkaline phosphatase, liver/bone/kidney (ALPL), mRNA [NM_000478]	**1.247**
DNM3	Homo sapiens dynamin 3 (DNM3), transcript variant 1, mRNA [NM_015569]	**1.247**
UST	Homo sapiens uronyl-2-sulfotransferase (UST), mRNA [NM_005715]	**1.246**
ATP10D	Homo sapiens ATPase, class V, type 10D (ATP10D), mRNA [NM_020453]	**1.245**
IDH1	Homo sapiens isocitrate dehydrogenase 1 (NADP+), soluble (IDH1), mRNA [NM_005896]	**1.244**
COL24A1	Homo sapiens collagen, type XXIV, alpha 1 (COL24A1), mRNA [NM_152890]	**1.243**
NQO2	Homo sapiens NAD(P)H dehydrogenase, quinone 2 (NQO2), mRNA [NM_000904]	**1.243**
SCIN	Homo sapiens scinderin (SCIN), mRNA [NM_033128]	**1.243**
CFB	Homo sapiens complement factor B (CFB), mRNA [NM_001710]	**1.242**
TMEM53	Homo sapiens transmembrane protein 53 (TMEM53), mRNA [NM_024587]	**1.239**
ANTXR2	Homo sapiens anthrax toxin receptor 2 (ANTXR2), mRNA [NM_058172]	**1.237**
PTK6	Homo sapiens PTK6 protein tyrosine kinase 6 (PTK6), mRNA [NM_005975]	**1.235**
RASA3	Homo sapiens mRNA for Ins(1,3,4,5)P4-binding protein. [X89399]	**1.234**
KIF3C	Homo sapiens kinesin family member 3C (KIF3C), mRNA [NM_002254]	**1.234**
PI3	Homo sapiens peptidase inhibitor 3, skin-derived (PI3), mRNA [NM_002638]	**1.233**
RAB7L1	Homo sapiens RAB7, member RAS oncogene family-like 1 (RAB7L1), mRNA [NM_003929]	**1.233**
CRISPLD2	Homo sapiens cysteine-rich secretory protein LCCL domain containing 2 (CRISPLD2), mRNA [NM_031476]	**1.230**
PRDX5	Homo sapiens peroxiredoxin 5 (PRDX5), nuclear gene encoding mitochondrial protein, mRNA [NM_012094]	**1.230**
KIF26A	Homo sapiens kinesin family member 26A (KIF26A), mRNA [NM_015656]	**1.229**

PROP1	Homo sapiens PROP paired-like homeobox 1 (PROP1), mRNA [NM_006261]	**1.227**
GBA	Homo sapiens glucosidase, beta; acid (includes glucosylceramidase) (GBA), mRNA [NM_001005749]	**1.226**
SNX10	Homo sapiens sorting nexin 10 (SNX10), mRNA [NM_013322]	**1.224**
FLJ35024	Homo sapiens hypothetical LOC401491 (FLJ35024), non-coding RNA [NR_015375]	**1.223**
C7orf57	Homo sapiens chromosome 7 open reading frame 57 (C7orf57), mRNA [NM_001100159]	**1.221**
HEXA	HEXA {HEXA4bpDeltaA mutation, exon 11} [human, Tay-Sachs disease patient, mRNA Partial Mutant, 78 nt]. [S76980]	**1.221**
LAMA1	Homo sapiens laminin, alpha 1 (LAMA1), mRNA [NM_005559]	**1.221**
FASN	Homo sapiens fatty acid synthase (FASN), mRNA [NM_004104]	**1.220**
BNIP3	Homo sapiens BCL2/adenovirus E1B 19kDa interacting protein 3 (BNIP3), nuclear gene encoding mitochondrial protein, mRNA [NM_004052]	**1.217**
CORO1B	Homo sapiens coronin, actin binding protein, 1B (CORO1B), mRNA [NM_020441]	**1.216**
GAPDH	Homo sapiens glyceraldehyde-3-phosphate dehydrogenase (GAPDH), mRNA [NM_002046]	**1.215**
NAT2	Homo sapiens N-acetyltransferase 2 (arylamine N-acetyltransferase) (NAT2), mRNA [NM_000015]	**1.215**
RRAGD	Homo sapiens Ras-related GTP binding D (RRAGD), mRNA [NM_021244]	**1.215**
DOCK8	Homo sapiens dedicator of cytokinesis 8 (DOCK8), mRNA [NM_203447]	**1.214**
C1orf211	Homo sapiens chromosome 1 open reading frame 211, mRNA (cDNA clone MGC:40168 IMAGE:5141008), complete cds. [BC030279]	**1.210**
MMP15	Homo sapiens matrix metallopeptidase 15 (membrane-inserted) (MMP15), mRNA [NM_002428]	**1.209**
BIK	Homo sapiens BCL2-interacting killer (apoptosis-inducing) (BIK), mRNA [NM_001197]	**1.208**
PCDHGA7	Homo sapiens protocadherin gamma subfamily A, 7 (PCDHGA7), mRNA [NM_032087]	**1.208**
BRI3	Homo sapiens brain protein I3 (BRI3), mRNA [NM_015379]	**1.206**
TRAM1	Homo sapiens translocation associated membrane protein 1 (TRAM1), mRNA [NM_014294]	**1.206**
MMAB	Homo sapiens methylmalonic aciduria (cobalamin deficiency) cblB type (MMAB), nuclear gene encoding mitochondrial protein, mRNA [NM_052845]	**1.203**
HDAC5	Homo sapiens histone deacetylase 5 (HDAC5), mRNA [NM_001015053]	**1.201**
GSTM4	Homo sapiens glutathione S-transferase mu 4 (GSTM4), mRNA [NM_147148]	**1.200**
HIP1R	Homo sapiens huntingtin interacting protein 1 related (HIP1R), mRNA [NM_003959]	**1.200**
MYEOV	Homo sapiens myeloma overexpressed (in a subset of t(11;14) positive multiple myelomas) (MYEOV), mRNA [NM_138768]	**1.200**
IL17RC	Homo sapiens interleukin 17 receptor C (IL17RC), mRNA [NM_153461]	**1.199**
LPAR5	Homo sapiens lysophosphatidic acid receptor 5 (LPAR5), mRNA	**1.197**

	[NM_020400]	
TPP1	Homo sapiens tripeptidyl peptidase I (TPP1), mRNA [NM_000391]	**1.197**
ZBTB41	Homo sapiens zinc finger and BTB domain containing 41 (ZBTB41), mRNA [NM_194314]	**1.196**
EID3	Homo sapiens EP300 interacting inhibitor of differentiation 3 (EID3), mRNA [NM_001008394]	**1.195**
MMP7	Homo sapiens matrix metallopeptidase 7 (matrilysin, uterine) (MMP7), mRNA [NM_002423]	**1.195**
C16orf3	Homo sapiens chromosome 16 open reading frame 3 (C16orf3), mRNA [NM_001214]	**1.194**
STON2	Homo sapiens stonin 2 (STON2), mRNA [NM_033104]	**1.193**
TMEM173	Homo sapiens transmembrane protein 173 (TMEM173), mRNA [NM_198282]	**1.192**
HMGCS1	Homo sapiens 3-hydroxy-3-methylglutaryl-Coenzyme A synthase 1 (soluble) (HMGCS1), mRNA [NM_002130]	**1.192**
TXN	Homo sapiens thioredoxin (TXN), mRNA [NM_003329]	**1.191**
FAM9B	Homo sapiens family with sequence similarity 9, member B (FAM9B), mRNA [NM_205849]	**1.187**
LIMS3	Homo sapiens LIM and senescent cell antigen-like domains 3 (LIMS3), mRNA [NM_033514]	**1.187**
FADS3	Homo sapiens fatty acid desaturase 3 (FADS3), mRNA [NM_021727]	**1.186**
RELL2	Homo sapiens RELT-like 2 (RELL2), mRNA [NM_173828]	**1.186**
IFITM5	Homo sapiens interferon induced transmembrane protein 5 (IFITM5), mRNA [NM_001025295]	**1.185**
HYAL1	Homo sapiens hyaluronoglucosaminidase 1 (HYAL1), mRNA [NM_007312]	**1.184**
LRRC32	Homo sapiens leucine rich repeat containing 32 (LRRC32), mRNA [NM_005512]	**1.184**
FSTL3	Homo sapiens follistatin-like 3 (secreted glycoprotein) (FSTL3), mRNA [NM_005860]	**1.183**
SHISA4	Homo sapiens shisa homolog 4 (Xenopus laevis) (SHISA4), mRNA [NM_198149]	**1.182**
HABP4	Homo sapiens hyaluronan binding protein 4 (HABP4), mRNA [NM_014282]	**1.181**
SLC27A6	Homo sapiens solute carrier family 27 (fatty acid transporter), member 6 (SLC27A6), mRNA [NM_001017372]	**1.181**
ZFPM1	Homo sapiens zinc finger protein, multitype 1 (ZFPM1), mRNA [NM_153813]	**1.181**
EPGN	Homo sapiens epithelial mitogen homolog (mouse) (EPGN), mRNA [NM_001013442]	**1.179**
GRN	Homo sapiens granulin (GRN), mRNA [NM_002087]	**1.178**
AMBP	Homo sapiens alpha-1-microglobulin/bikunin precursor (AMBP), mRNA [NM_001633]	**1.177**
SNCA	Homo sapiens synuclein, alpha (non A4 component of amyloid precursor) (SNCA), mRNA [NM_007308]	**1.174**
FAM46B	Homo sapiens family with sequence similarity 46, member B (FAM46B),	**1.173**

	mRNA [NM_052943]	
PKN1	Homo sapiens protein kinase N1 (PKN1), mRNA [NM_002741]	**1.173**
ARHGEF4	Homo sapiens Rho guanine nucleotide exchange factor (GEF) 4 (ARHGEF4), mRNA [NM_032995]	**1.172**
HTRA3	Homo sapiens HtrA serine peptidase 3 (HTRA3), mRNA [NM_053044]	**1.172**
GRAMD1C	Homo sapiens GRAM domain containing 1C (GRAMD1C), mRNA [NM_017577]	**1.169**
ACO1	Homo sapiens aconitase 1, soluble (ACO1), mRNA [NM_002197]	**1.168**
FRMD5	Homo sapiens FERM domain containing 5 (FRMD5), mRNA [NM_032892]	**1.166**
PPARGC1A	Homo sapiens peroxisome proliferator-activated receptor gamma, coactivator 1 alpha (PPARGC1A), mRNA [NM_013261]	**1.166**
STXBP6	Homo sapiens cDNA FLJ39638 fis, clone SMINT2003317. [AK096957]	**1.166**
C3	Homo sapiens complement component 3 (C3), mRNA [NM_000064]	**1.164**
CYBRD1	Homo sapiens cytochrome b reductase 1 (CYBRD1), mRNA [NM_024843]	**1.161**
FMN1	Homo sapiens formin 1, mRNA (cDNA clone IMAGE:4213061). [BC029107]	**1.161**
NR0B1	Homo sapiens nuclear receptor subfamily 0, group B, member 1 (NR0B1), mRNA [NM_000475]	**1.160**
SPIRE1	Homo sapiens mRNA; cDNA DKFZp434O034 (from clone DKFZp434O034). [AL833817]	**1.160**
TIPARP	Homo sapiens TCDD-inducible poly(ADP-ribose) polymerase (TIPARP), mRNA [NM_015508]	**1.160**
C2orf18	Homo sapiens chromosome 2 open reading frame 18 (C2orf18), mRNA [NM_017877]	**1.159**
MAPK13	Homo sapiens mitogen-activated protein kinase 13 (MAPK13), mRNA [NM_002754]	**1.159**
CASP4	Homo sapiens caspase 4, apoptosis-related cysteine peptidase (CASP4), mRNA [NM_033306]	**1.158**
PLEKHM2	Homo sapiens pleckstrin homology domain containing, family M (with RUN domain) member 2 (PLEKHM2), mRNA [NM_015164]	**1.157**
PSORS1C1	Homo sapiens psoriasis susceptibility 1 candidate 1 (PSORS1C1), mRNA [NM_014068]	**1.156**
DAB2	Homo sapiens disabled homolog 2, mitogen-responsive phosphoprotein (Drosophila) (DAB2), mRNA [NM_001343]	**1.155**
SPG21	Homo sapiens spastic paraplegia 21 (autosomal recessive, Mast syndrome) (SPG21), mRNA [NM_016630]	**1.155**
GSTA4	Homo sapiens glutathione S-transferase alpha 4 (GSTA4), mRNA [NM_001512]	**1.153**
RAB4B	Homo sapiens RAB4B, member RAS oncogene family (RAB4B), mRNA [NM_016154]	**1.152**
PPP1R14C	Homo sapiens protein phosphatase 1, regulatory (inhibitor) subunit 14C (PPP1R14C), mRNA [NM_030949]	**1.152**
STEAP1	Homo sapiens six transmembrane epithelial antigen of the prostate 1 (STEAP1), mRNA [NM_012449]	**1.151**

ABLIM3	Homo sapiens actin binding LIM protein family, member 3 (ABLIM3), mRNA [NM_014945]	**1.150**
ARL8A	Homo sapiens ADP-ribosylation factor-like 8A (ARL8A), mRNA [NM_138795]	**1.150**
KCNJ2	Homo sapiens potassium inwardly-rectifying channel, subfamily J, member 2 (KCNJ2), mRNA [NM_000891]	**1.150**
NAPA	Homo sapiens N-ethylmaleimide-sensitive factor attachment protein, alpha (NAPA), mRNA [NM_003827]	**1.148**
DAPK2	Homo sapiens death-associated protein kinase 2 (DAPK2), mRNA [NM_014326]	**1.147**
SQRDL	Homo sapiens sulfide quinone reductase-like (yeast) (SQRDL), nuclear gene encoding mitochondrial protein, mRNA [NM_021199]	**1.147**
CRAT	Homo sapiens carnitine acetyltransferase (CRAT), nuclear gene encoding mitochondrial protein, mRNA [NM_000755]	**1.146**
FDPS	Homo sapiens farnesyl diphosphate synthase (farnesyl pyrophosphate synthetase, dimethylallyltranstransferase, geranyltranstransferase) (FDPS), mRNA [NM_002004]	**1.146**
STIM1	Homo sapiens stromal interaction molecule 1 (STIM1), mRNA [NM_003156]	**1.145**
TESC	Homo sapiens tescalcin (TESC), mRNA [NM_017899]	**1.144**
LDLR	Homo sapiens low density lipoprotein receptor (LDLR), mRNA [NM_000527]	**1.144**
ECM1	Homo sapiens extracellular matrix protein 1 (ECM1), mRNA [NM_004425]	**1.143**
OR5L2	Homo sapiens olfactory receptor, family 5, subfamily L, member 2 (OR5L2), mRNA [NM_001004739]	**1.143**
CLCN4	Homo sapiens chloride channel 4 (CLCN4), mRNA [NM_001830]	**1.142**
MOXD1	Homo sapiens monooxygenase, DBH-like 1 (MOXD1), mRNA [NM_015529]	**1.141**
SIRT2	Homo sapiens sirtuin (silent mating type information regulation 2 homolog) 2 (S. cerevisiae) (SIRT2), mRNA [NM_012237]	**1.140**
YIF1B	Homo sapiens Yip1 interacting factor homolog B (S. cerevisiae) (YIF1B), mRNA [NM_033557]	**1.140**
SAT1	Homo sapiens spermidine/spermine N1-acetyltransferase 1 (SAT1), mRNA [NM_002970]	**1.140**
NT5E	Homo sapiens 5'-nucleotidase, ecto (CD73) (NT5E), mRNA [NM_002526]	**1.139**
SLC7A11	Homo sapiens solute carrier family 7,(cationic amino acid transporter, y+ system) member 11 (SLC7A11), mRNA [NM_014331]	**1.137**
AMPD3	Homo sapiens adenosine monophosphate deaminase (isoform E) (AMPD3), mRNA [NM_001025390]	**1.137**
RILP	Homo sapiens Rab interacting lysosomal protein (RILP), mRNA [NM_031430]	**1.136**
NPAS2	Homo sapiens neuronal PAS domain protein 2 (NPAS2), mRNA [NM_002518]	**1.135**
COL6A1	Homo sapiens collagen, type VI, alpha 1 (COL6A1), mRNA [NM_001848]	**1.133**
RBKS	Homo sapiens ribokinase (RBKS), mRNA [NM_022128]	**1.133**
NAMPT	Homo sapiens nicotinamide phosphoribosyltransferase (NAMPT), mRNA [NM_005746]	**1.131**

ANXA4	Homo sapiens annexin A4 (ANXA4), mRNA [NM_001153]	**1.131**
SAA2	Homo sapiens cDNA clone IMAGE:4716555, with apparent retained intron. [BC058008]	**1.130**
TMEM198	Homo sapiens transmembrane protein 198 (TMEM198), mRNA [NM_001005209]	**1.130**
ATP6V0A1	Homo sapiens ATPase, H+ transporting, lysosomal V0 subunit a1 (ATP6V0A1), transcript variant 3, mRNA [NM_005177]	**1.129**
RAB23	Homo sapiens RAB23, member RAS oncogene family (RAB23), mRNA [NM_016277]	**1.129**
AGPAT9	Homo sapiens 1-acylglycerol-3-phosphate O-acyltransferase 9 (AGPAT9), mRNA [NM_032717]	**1.128**
CD274	Homo sapiens CD274 molecule (CD274), mRNA [NM_014143]	**1.128**
KIR2DS4	Homo sapiens killer cell immunoglobulin-like receptor, two domains, short cytoplasmic tail, 4 (KIR2DS4), mRNA [NM_012314]	**1.128**
LOC100126784	Homo sapiens cDNA clone IMAGE:4822429. [BC047636]	**1.127**
PRDX1	Homo sapiens peroxiredoxin 1 (PRDX1), mRNA [NM_002574]	**1.123**
PTX3	Homo sapiens pentraxin-related gene, rapidly induced by IL-1 beta (PTX3), mRNA [NM_002852]	**1.123**
ACAT2	Homo sapiens acetyl-Coenzyme A acetyltransferase 2 (ACAT2), mRNA [NM_005891]	**1.123**
STARD5	Homo sapiens StAR-related lipid transfer (START) domain containing 5 (STARD5), mRNA [NM_181900]	**1.123**
INA	Homo sapiens internexin neuronal intermediate filament protein, alpha (INA), mRNA [NM_032727]	**1.122**
RPUSD3	Homo sapiens RNA pseudouridylate synthase domain containing 3 (RPUSD3), mRNA [NM_173659]	**1.122**
GGTLC2	Homo sapiens gamma-glutamyltransferase light chain 2 (GGTLC2), mRNA [NM_199127]	**1.121**
MAPK3	Homo sapiens mitogen-activated protein kinase 3 (MAPK3), mRNA [NM_002746]	**1.120**
G0S2	Homo sapiens G0/G1switch 2 (G0S2), mRNA [NM_015714]	**1.118**
GAS6	Homo sapiens growth arrest-specific 6 (GAS6), mRNA [NM_000820]	**1.117**
SCAMP3	Homo sapiens secretory carrier membrane protein 3 (SCAMP3), mRNA [NM_052837]	**1.117**
APOBEC3B	Homo sapiens apolipoprotein B mRNA editing enzyme, catalytic polypeptide-like 3B (APOBEC3B), mRNA [NM_004900]	**1.116**
SLC30A3	Homo sapiens solute carrier family 30 (zinc transporter), member 3 (SLC30A3), mRNA [NM_003459]	**1.116**
TMEM55B	Homo sapiens transmembrane protein 55B (TMEM55B), mRNA [NM_144568]	**1.116**
RAPGEF1	Homo sapiens Rap guanine nucleotide exchange factor (GEF) 1 (RAPGEF1), mRNA [NM_198679]	**1.115**
TMEM120A	Homo sapiens transmembrane protein 120A (TMEM120A), mRNA	**1.115**

	[NM_031925]	
SEMA3F	Homo sapiens sema domain, immunoglobulin domain (Ig), short basic domain, secreted, (semaphorin) 3F (SEMA3F), mRNA [NM_004186]	**1.115**
AMDHD1	Homo sapiens amidohydrolase domain containing 1 (AMDHD1), mRNA [NM_152435]	**1.114**
IFI30	Homo sapiens interferon, gamma-inducible protein 30 (IFI30), mRNA [NM_006332]	**1.114**
MRCL3	Homo sapiens myosin regulatory light chain MRCL3 (MRCL3), mRNA [NM_006471]	**1.114**
PLCL1	Homo sapiens phospholipase C-like 1 (PLCL1), mRNA [NM_006226]	**1.114**
PPP2R2C	Homo sapiens protein phosphatase 2 (formerly 2A), regulatory subunit B, gamma isoform (PPP2R2C), mRNA [NM_020416]	**1.114**
C11orf9	Homo sapiens chromosome 11 open reading frame 9 (C11orf9), mRNA [NM_013279]	**1.111**
SOD3	Homo sapiens superoxide dismutase 3, extracellular (SOD3), mRNA [NM_003102]	**1.110**
TMEM161A	Homo sapiens transmembrane protein 161A (TMEM161A), mRNA [NM_017814]	**1.110**
TAPBP	Homo sapiens TAP binding protein (tapasin) (TAPBP), mRNA [NM_172208]	**1.109**
RASD1	Homo sapiens RAS, dexamethasone-induced 1 (RASD1), mRNA [NM_016084]	**1.108**
RPL41	Homo sapiens ribosomal protein L41 (RPL41), mRNA [NM_001035267]	**1.107**
ENDOD1	Homo sapiens endonuclease domain containing 1 (ENDOD1), mRNA [NM_015036]	**1.107**
RGNEF	Homo sapiens Rho-guanine nucleotide exchange factor (RGNEF), mRNA [NM_001080479]	**1.106**
CD63	Homo sapiens CD63 molecule (CD63), mRNA [NM_001040034]	**1.105**
WIPI1	Homo sapiens WD repeat domain, phosphoinositide interacting 1 (WIPI1), mRNA [NM_017983]	**1.105**
ZEB2	Homo sapiens zinc finger E-box binding homeobox 2 (ZEB2), mRNA [NM_014795]	**1.105**
EPHX2	Homo sapiens epoxide hydrolase 2, cytoplasmic (EPHX2), mRNA [NM_001979]	**1.104**
LOC645195	Homo sapiens cDNA FLJ41456 fis, clone BRSTN2012320. [AK123450]	**1.104**
KIAA0415	Homo sapiens KIAA0415 (KIAA0415), mRNA [NM_014855]	**1.103**
LRRC18	Homo sapiens clone DNA193663 VKGE9338 (UNQ9338) mRNA, complete cds. [AY358137]	**1.103**
MLLT11	Homo sapiens myeloid/lymphoid or mixed-lineage leukemia (trithorax homolog, Drosophila); translocated to, 11 (MLLT11), mRNA [NM_006818]	**1.102**
RAG1AP1	Homo sapiens recombination activating gene 1 activating protein 1 (RAG1AP1), mRNA [NM_018845]	**1.102**
ARMCX1	Homo sapiens armadillo repeat containing, X-linked 1 (ARMCX1), mRNA [NM_016608]	**1.100**

NRG1	Homo sapiens neuregulin 1 (NRG1), transcript variant GGF2, mRNA [NM_013962]	**1.100**
RAB30	Homo sapiens RAB30, member RAS oncogene family (RAB30), mRNA [NM_014488]	**1.100**
FBLN2	Homo sapiens fibulin 2 (FBLN2), mRNA [NM_001004019]	**1.099**
TKT	Homo sapiens transketolase (TKT), mRNA [NM_001064]	**1.099**
ABCA5	Homo sapiens ATP-binding cassette, sub-family A (ABC1), member 5 (ABCA5), mRNA [NM_018672]	**1.098**
PTGR1	Homo sapiens prostaglandin reductase 1 (PTGR1), mRNA [NM_012212]	**1.098**
STX17	Homo sapiens syntaxin 17 (STX17), mRNA [NM_017919]	**1.098**
CEBPB	Homo sapiens CCAAT/enhancer binding protein (C/EBP), beta (CEBPB), mRNA [NM_005194]	**1.098**
LOC340888	Homo sapiens misc_RNA (LOC340888), miscRNA [XR_018726]	**1.096**
VEGFC	Homo sapiens vascular endothelial growth factor C (VEGFC), mRNA [NM_005429]	**1.096**
ATP6AP1	Homo sapiens ATPase, H+ transporting, lysosomal accessory protein 1 (ATP6AP1), mRNA [NM_001183]	**1.095**
CYP4F11	Homo sapiens cytochrome P450, family 4, subfamily F, polypeptide 11 (CYP4F11), mRNA [NM_021187]	**1.094**
TSC22D3	Homo sapiens TSC22 domain family, member 3 (TSC22D3), mRNA [NM_004089]	**1.094**
VCX	Homo sapiens variable charge, X-linked (VCX), mRNA [NM_013452]	**1.092**
MAGIX	Homo sapiens MAGI family member, X-linked (MAGIX), mRNA [NM_024859]	**1.091**
MAP2K2	Homo sapiens mitogen-activated protein kinase kinase 2 (MAP2K2), mRNA [NM_030662]	**1.091**
POPDC3	Homo sapiens popeye domain containing 3 (POPDC3), mRNA [NM_022361]	**1.091**
TSKU	Homo sapiens tsukushin (TSKU), mRNA [NM_015516]	**1.091**
UCA1	Homo sapiens urothelial cancer associated 1 (UCA1), non-coding RNA [NR_015379]	**1.091**
ATP6V0B	Homo sapiens ATPase, H+ transporting, lysosomal 21kDa, V0 subunit b (ATP6V0B), mRNA [NM_004047]	**1.090**
SERPINB7	Homo sapiens serpin peptidase inhibitor, clade B (ovalbumin), member 7 (SERPINB7), mRNA [NM_001040147]	**1.090**
LOC389033	Homo sapiens hypothetical LOC389033 (LOC389033), mRNA [XM_374010]	**1.089**
POTEF	Homo sapiens POTE ankyrin domain family, member F (POTEF), mRNA [NM_001099771]	**1.089**
COX1	Cytochrome c oxidase subunit 1 (Cytochrome c oxidase polypeptide I) [Source:UniProtKB/Swiss-Prot;Acc:P00395] [ENST00000361624]	**1.087**
CDIPT	Homo sapiens CDP-diacylglycerol--inositol 3-phosphatidyltransferase (CDIPT), mRNA [NM_006319]	**1.085**
GRAMD1A	Homo sapiens GRAM domain containing 1A (GRAMD1A), mRNA [NM_020895]	**1.085**

PANX2	Homo sapiens pannexin 2 isoform 2 (PANX2) mRNA. [AF398511]	**1.084**
SLC39A1	Homo sapiens solute carrier family 39 (zinc transporter), member 1 (SLC39A1), mRNA [NM_014437]	**1.084**
UCHL1	Homo sapiens ubiquitin carboxyl-terminal esterase L1 (ubiquitin thiolesterase) (UCHL1), mRNA [NM_004181]	**1.083**
SERPINE1	Homo sapiens serpin peptidase inhibitor, clade E (nexin, plasminogen activator inhibitor type 1), member 1 (SERPINE1), mRNA [NM_000602]	**1.082**
ABCA4	Homo sapiens ATP-binding cassette, sub-family A (ABC1), member 4 (ABCA4), mRNA [NM_000350]	**1.081**
ABHD3	Homo sapiens abhydrolase domain containing 3 (ABHD3), mRNA [NM_138340]	**1.081**
MGAT4B	Homo sapiens mannosyl (alpha-1,3-)-glycoprotein beta-1,4-N-acetylglucos-aminyltransferase, isozyme B (MGAT4B), mRNA [NM_054013]	**1.081**
IL7R	Homo sapiens interleukin 7 receptor (IL7R), mRNA [NM_002185]	**1.080**
BCL7A	Homo sapiens B-cell CLL/lymphoma 7A (BCL7A), mRNA [NM_020993]	**1.079**
WBSCR27	Homo sapiens Williams Beuren syndrome chromosome region 27 (WBSCR27), mRNA [NM_152559]	**1.079**
DNAJB9	Homo sapiens DnaJ (Hsp40) homolog, subfamily B, member 9 (DNAJB9), mRNA [NM_012328]	**1.078**
RIT1	Homo sapiens Ras-like without CAAX 1 (RIT1), mRNA [NM_006912]	**1.078**
B2M	Homo sapiens beta-2-microglobulin (B2M), mRNA [NM_004048]	**1.077**
FAR2	Homo sapiens fatty acyl CoA reductase 2 (FAR2), mRNA [NM_018099]	**1.077**
ERMP1	Homo sapiens endoplasmic reticulum metallopeptidase 1 (ERMP1), mRNA [NM_024896]	**1.076**
RND3	Homo sapiens Rho family GTPase 3 (RND3), mRNA [NM_005168]	**1.076**
SV2B	Homo sapiens synaptic vesicle glycoprotein 2B (SV2B), mRNA [NM_014848]	**1.075**
ZNF213	Homo sapiens zinc finger protein 213 (ZNF213), mRNA [NM_004220]	**1.074**
FNDC3A	Homo sapiens fibronectin type III domain containing 3A (FNDC3A), mRNA [NM_001079673]	**1.072**
EPDR1	Homo sapiens ependymin related protein 1 (zebrafish) (EPDR1), mRNA [NM_017549]	**1.071**
AKR1B1	Homo sapiens aldo-keto reductase family 1, member B1 (aldose reductase) (AKR1B1), mRNA [NM_001628]	**1.070**
LPIN1	Homo sapiens lipin 1 (LPIN1), mRNA [NM_145693]	**1.070**
GBP4	Homo sapiens guanylate binding protein 4 (GBP4), mRNA [NM_052941]	**1.069**
NRIP3	Homo sapiens nuclear receptor interacting protein 3 (NRIP3), mRNA [NM_020645]	**1.069**
C1orf38	Homo sapiens chromosome 1 open reading frame 38 (C1orf38), mRNA [NM_001039477]	**1.068**
HIF1AN	Homo sapiens hypoxia inducible factor 1, alpha subunit inhibitor (HIF1AN), mRNA [NM_017902]	**1.068**
PDGFB	Homo sapiens platelet-derived growth factor beta polypeptide (simian sarcoma viral (v-sis) oncogene homolog) (PDGFB), mRNA [NM_002608]	**1.068**

LOC145783	Homo sapiens hypothetical LOC145783 (LOC145783), non-coding RNA [NR_015419]	**1.067**
PIWIL4	Homo sapiens piwi-like 4 (Drosophila) (PIWIL4), mRNA [NM_152431]	**1.067**
LHFPL2	Homo sapiens lipoma HMGIC fusion partner-like 2 (LHFPL2), mRNA [NM_005779]	**1.066**
DUSP10	Homo sapiens dual specificity phosphatase 10 (DUSP10), mRNA [NM_007207]	**1.064**
MAP3K9	Homo sapiens cDNA FLJ41436 fis, clone BRHIP2007741. [AK123430]	**1.064**
PAK1	Homo sapiens p21 protein (Cdc42/Rac)-activated kinase 1 (PAK1), mRNA [NM_002576]	**1.064**
ACE	Homo sapiens angiotensin I converting enzyme (peptidyl-dipeptidase A) 1 (ACE), mRNA [NM_000789]	**1.063**
BACE2	Homo sapiens beta-site APP-cleaving enzyme 2 (BACE2), mRNA [NM_012105]	**1.063**
C9orf91	Homo sapiens chromosome 9 open reading frame 91 (C9orf91), mRNA [NM_153045]	**1.061**
EML2	Homo sapiens echinoderm microtubule associated protein like 2 (EML2), mRNA [NM_012155]	**1.061**
MAPK8IP2	Homo sapiens mitogen-activated protein kinase 8 interacting protein 2 (MAPK8IP2), mRNA [NM_012324]	**1.061**
DIRC1	Homo sapiens disrupted in renal carcinoma 1 (DIRC1), mRNA [NM_052952]	**1.060**
FGL1	Homo sapiens fibrinogen-like 1 (FGL1), mRNA [NM_201553]	**1.060**
GNS	Homo sapiens glucosamine (N-acetyl)-6-sulfatase (GNS), mRNA [NM_002076]	**1.059**
CYP11A1	Homo sapiens cytochrome P450, family 11, subfamily A, polypeptide 1 (CYP11A1), mRNA [NM_000781]	**1.058**
NCRNA00120	Homo sapiens non-protein coding RNA 120 (NCRNA00120), non-coding RNA [NR_002767]	**1.058**
F8A1	Homo sapiens coagulation factor VIII-associated (intronic transcript) 1 (F8A1), mRNA [NM_012151]	**1.057**
PPP1R16B	Homo sapiens protein phosphatase 1, regulatory (inhibitor) subunit 16B (PPP1R16B), mRNA [NM_015568]	**1.057**
ABCB9	Homo sapiens ATP-binding cassette, sub-family B (MDR/TAP), member 9 (ABCB9), mRNA [NM_019625]	**1.056**
SLC35F5	Homo sapiens solute carrier family 35, member F5 (SLC35F5), mRNA [NM_025181]	**1.056**
GPR137B	Homo sapiens G protein-coupled receptor 137B (GPR137B), mRNA [NM_003272]	**1.055**
P2RX6P	Homo sapiens purinergic receptor P2X, ligand-gated ion channel, 6 pseudogene (P2RX6P), non-coding RNA [NR_002829]	**1.055**
PDE5A	Homo sapiens phosphodiesterase 5A, cGMP-specific (PDE5A), mRNA [NM_033430]	**1.055**
SULT1A1	Homo sapiens sulfotransferase family, cytosolic, 1A, phenol-preferring,	**1.055**

	member 1 (SULT1A1), mRNA [NM_177529]	
EHD3	Homo sapiens EH-domain containing 3 (EHD3), mRNA [NM_014600]	**1.054**
JPH2	Homo sapiens junctophilin 2 (JPH2), mRNA [NM_020433]	**1.054**
RBCK1	Homo sapiens RanBP-type and C3HC4-type zinc finger containing 1 (RBCK1), mRNA [NM_031229]	**1.054**
PGPEP1	Homo sapiens pyroglutamyl-peptidase I (PGPEP1), mRNA [NM_017712]	**1.054**
SOCS3	Homo sapiens suppressor of cytokine signaling 3 (SOCS3), mRNA [NM_003955]	**1.053**
PLAU	Homo sapiens plasminogen activator, urokinase (PLAU), mRNA [NM_002658]	**1.052**
SPOCK1	Homo sapiens sparc/osteonectin, cwcv and kazal-like domains proteoglycan (testican) 1 (SPOCK1), mRNA [NM_004598]	**1.051**
EMP3	Homo sapiens epithelial membrane protein 3 (EMP3), mRNA [NM_001425]	**1.049**
MED16	Homo sapiens mediator complex subunit 16 (MED16), mRNA [NM_005481]	**1.049**
RP6-166C19.11	Homo sapiens cancer/testis CT47 family, member 11 (CT47.11), mRNA [NM_173571]	**1.049**
LOC158960	Homo sapiens, clone IMAGE:3613029, mRNA, partial cds. [BC009467]	**1.048**
MS4A7	Homo sapiens membrane-spanning 4-domains, subfamily A, member 7 (MS4A7), mRNA [NM_021201]	**1.047**
CTSA	Homo sapiens cathepsin A (CTSA), transcript variant 1, mRNA [NM_000308]	**1.046**
EPS15L1	Homo sapiens epidermal growth factor receptor pathway substrate 15-like 1 (EPS15L1), mRNA [NM_021235]	**1.044**
CTH	Homo sapiens cystathionase (cystathionine gamma-lyase) (CTH), mRNA [NM_001902]	**1.042**
STAT5A	Homo sapiens signal transducer and activator of transcription 5A (STAT5A), mRNA [NM_003152]	**1.042**
CUTA	Homo sapiens cutA divalent cation tolerance homolog (E. coli) (CUTA), transcript variant 2, mRNA [NM_015921]	**1.041**
GALE	Homo sapiens UDP-galactose-4-epimerase (GALE), mRNA [NM_000403]	**1.041**
CDC25B	Homo sapiens cell division cycle 25 homolog B (S. pombe) (CDC25B), transcript variant 1, mRNA [NM_021873]	**1.040**
NICN1	Homo sapiens nicolin 1 (NICN1), mRNA [NM_032316]	**1.039**
ENO1	Homo sapiens enolase 1, (alpha) (ENO1), mRNA [NM_001428]	**1.039**
RNF14	Homo sapiens ring finger protein 14 (RNF14), mRNA [NM_004290]	**1.038**
AFF1	Homo sapiens AF4/FMR2 family, member 1 (AFF1), mRNA [NM_005935]	**1.038**
TMBIM1	Homo sapiens transmembrane BAX inhibitor motif containing 1 (TMBIM1), mRNA [NM_022152]	**1.038**
TNFAIP8L3	Homo sapiens tumor necrosis factor, alpha-induced protein 8-like 3 (TNFAIP8L3), mRNA [NM_207381]	**1.038**
C6orf145	Homo sapiens chromosome 6 open reading frame 145 (C6orf145), mRNA [NM_183373]	**1.037**
CLDN23	Homo sapiens claudin 23 (CLDN23), mRNA [NM_194284]	**1.037**
SIRT6	Homo sapiens sirtuin (silent mating type information regulation 2 homolog) 6	**1.037**

	(S. cerevisiae) (SIRT6), mRNA [NM_016539]	
TMEM8	Homo sapiens transmembrane protein 8 (five membrane-spanning domains) (TMEM8), mRNA [NM_021259]	**1.037**
FLYWCH1	Homo sapiens FLYWCH-type zinc finger 1 (FLYWCH1), mRNA [NM_020912]	**1.037**
MGAT5B	Homo sapiens mannosyl (alpha-1,6-)-glycoprotein beta-1,6-N-acetyl-glucosaminyltransferase, isozyme B (MGAT5B), mRNA [NM_144677]	**1.036**
PLA2G6	Homo sapiens phospholipase A2, group VI (cytosolic, calcium-independent) (PLA2G6), transcript variant 1, mRNA [NM_003560]	**1.036**
PSMB8	Homo sapiens proteasome (prosome, macropain) subunit, beta type, 8 (large multifunctional peptidase 7) (PSMB8), mRNA [NM_004159]	**1.036**
IL8	Homo sapiens interleukin 8 (IL8), mRNA [NM_000584]	**1.035**
PDZK1IP1	Homo sapiens PDZK1 interacting protein 1 (PDZK1IP1), mRNA [NM_005764]	**1.035**
TTC9	Homo sapiens tetratricopeptide repeat domain 9 (TTC9), mRNA [NM_015351]	**1.035**
ICAM2	Homo sapiens intercellular adhesion molecule 2 (ICAM2), mRNA [NM_000873]	**1.034**
SLC26A9	Homo sapiens solute carrier family 26, member 9 (SLC26A9), mRNA [NM_052934]	**1.034**
FIBIN	Homo sapiens fin bud initiation factor homolog (zebrafish) (FIBIN), mRNA [NM_203371]	**1.033**
TCTA	Homo sapiens T-cell leukemia translocation altered gene (TCTA), mRNA [NM_022171]	**1.033**
CD276	Homo sapiens CD276 molecule (CD276), mRNA [NM_001024736]	**1.033**
ATP8B2	Homo sapiens ATPase, class I, type 8B, member 2 (ATP8B2), mRNA [NM_020452]	**1.030**
CHAC1	Homo sapiens ChaC, cation transport regulator homolog 1 (E. coli) (CHAC1), transcript variant 1, mRNA [NM_024111]	**1.029**
IFIT5	Homo sapiens interferon-induced protein with tetratricopeptide repeats 5 (IFIT5), mRNA [NM_012420]	**1.029**
GMDS	Homo sapiens GDP-mannose 4,6-dehydratase (GMDS), mRNA [NM_001500]	**1.028**
EPAS1	Homo sapiens endothelial PAS domain protein 1 (EPAS1), mRNA [NM_001430]	**1.028**
ATG2A	Homo sapiens ATG2 autophagy related 2 homolog A (S. cerevisiae) (ATG2A), mRNA [NM_015104]	**1.027**
FAM129B	Homo sapiens MEG3 (MEG3) mRNA. [AF151783]	**1.025**
DKK3	Homo sapiens dickkopf homolog 3 (Xenopus laevis) (DKK3), mRNA [NM_015881]	**1.024**
FXYD5	Homo sapiens FXYD domain containing ion transport regulator 5 (FXYD5), mRNA [NM_144779]	**1.024**
GIPC1	Homo sapiens GIPC PDZ domain containing family, member 1 (GIPC1), mRNA [NM_005716]	**1.023**

LDOC1	Homo sapiens leucine zipper, down-regulated in cancer 1 (LDOC1), mRNA [NM_012317]	**1.022**
TMEM139	Homo sapiens transmembrane protein 139 (TMEM139), mRNA [NM_153345]	**1.022**
ATF4	Homo sapiens activating transcription factor 4 (tax-responsive enhancer element B67) (ATF4), mRNA [NM_001675]	**1.022**
EHD4	Homo sapiens EH-domain containing 4 (EHD4), mRNA [NM_139265]	**1.021**
VCAN	Homo sapiens versican (VCAN), mRNA [NM_004385]	**1.021**
LOC407835	Homo sapiens mitogen-activated protein kinase kinase 2 pseudogene (LOC407835), non-coding RNA [NR_002144]	**1.020**
ZFPL1	Homo sapiens zinc finger protein-like 1 (ZFPL1), mRNA [NM_006782]	**1.018**
SPNS1	Homo sapiens spinster homolog 1 (Drosophila) (SPNS1), mRNA [NM_032038]	**1.017**
PSEN2	Homo sapiens presenilin 2 (Alzheimer disease 4) (PSEN2), mRNA [NM_000447]	**1.015**
CYBB	Homo sapiens cytochrome b-245, beta polypeptide (CYBB), mRNA [NM_000397]	**1.015**
TMEM38B	Homo sapiens transmembrane protein 38B (TMEM38B), mRNA [NM_018112]	**1.015**
ITK	Homo sapiens IL2-inducible T-cell kinase (ITK), mRNA [NM_005546]	**1.013**
WNT6	Homo sapiens wingless-type MMTV integration site family, member 6 (WNT6), mRNA [NM_006522]	**1.013**
SH3BP4	Homo sapiens SH3-domain binding protein 4 (SH3BP4), mRNA [NM_014521]	**1.012**
AKR1C3	Homo sapiens aldo-keto reductase family 1, member C3 (3-alpha hydroxysteroid dehydrogenase, type II) (AKR1C3), mRNA [NM_003739]	**1.011**
MPL	Homo sapiens myeloproliferative leukemia virus oncogene (MPL), mRNA [NM_005373]	**1.011**
JUP	Homo sapiens junction plakoglobin (JUP), mRNA [NM_002230]	**1.010**
TMEM99	Homo sapiens transmembrane protein 99 (TMEM99), mRNA [NM_145274]	**1.009**
GPC1	Homo sapiens glypican 1 (GPC1), mRNA [NM_002081]	**1.008**
LOC100130776	full-length cDNA clone CS0DC010YD15 of Neuroblastoma Cot 25-normalized of Homo sapiens (human). [CR590071]	**1.008**
TLR6	Homo sapiens toll-like receptor 6 (TLR6), mRNA [NM_006068]	**1.008**
JHDM1D	Homo sapiens jumonji C domain containing histone demethylase 1 homolog D (S. cerevisiae) (JHDM1D), mRNA [NM_030647]	**1.007**
P76	Homo sapiens mannose-6-phosphate protein p76 (P76), mRNA [NM_173542]	**1.006**
FLJ43692	Homo sapiens ARHGEF5-like (FLJ43692), mRNA [NM_001003702]	**1.005**
GM2A	Homo sapiens GM2 ganglioside activator (GM2A), mRNA [NM_000405]	**1.005**
HSPC152	Homo sapiens hypothetical protein HSPC152 (HSPC152), mRNA [NM_016404]	**1.003**
AVPI1	Homo sapiens arginine vasopressin-induced 1 (AVPI1), mRNA [NM_021732]	**1.002**
CTRB2	Homo sapiens chymotrypsinogen B2 (CTRB2), mRNA [NM_001025200]	**1.002**
LMBRD1	Homo sapiens LMBR1 domain containing 1 (LMBRD1), mRNA	**1.001**

	[NM_018368]	
UBTD1	Homo sapiens ubiquitin domain containing 1 (UBTD1), mRNA [NM_024954]	**1.001**
SDS	Homo sapiens serine dehydratase (SDS), mRNA [NM_006843]	**1.001**
CBX4	Homo sapiens chromobox homolog 4 (Pc class homolog, Drosophila) (CBX4), mRNA [NM_003655]	**1.000**
CHCHD10	Homo sapiens coiled-coil-helix-coiled-coil-helix domain containing 10 (CHCHD10), mRNA [NM_213720]	**1.000**
GPM6A	Homo sapiens glycoprotein M6A (GPM6A), mRNA [NM_201591]	**1.000**
RNF115	Homo sapiens ring finger protein 115 (RNF115), mRNA [NM_014455]	**1.000**
