# Supplementary material for: Genotoxicity and molecular response of silver nanoparticle (NP)-based hydrogel
Source: J Nanobiotechnology. 2012 May 1;10:16. doi: 10.1186/1477-3155-10-16 (PMC3430588; doi:10.1186/1477-3155-10-16)
Supplement: Additional file 4 — Down-regulated genes in cells exposed to silver-NPs-hydrogel for 48h. Fold-change is logarithmic ratio (log2 ratio) to expression level in control. [file 1477-3155-10-16-S4.pdf]

Additional File 4. Down-regulated genes in cells exposed to silver-NPs-hydrogel for 48h. Fold-change is logarithmic ratio (log_2 ratio) to expression level in control.

GeneName	Description	Fold-change (log_2 ratio)
TRIM73	Homo sapiens tripartite motif-containing 73, mRNA (cDNA clone IMAGE:5181460). [BC033812]	**-4.567**
CDC14A	Homo sapiens CDC14 cell division cycle 14 homolog A (S. cerevisiae) (CDC14A), mRNA [NM_003672]	**-3.711**
ID2	Homo sapiens inhibitor of DNA binding 2, dominant negative helix-loop-helix protein (ID2), mRNA [NM_002166]	**-3.424**
LOC344178	Homo sapiens similar to hCG1794703 (LOC344178), mRNA [XM_001721796]	**-3.203**
SULT1E1	Homo sapiens sulfotransferase family 1E, estrogen-preferring, member 1 (SULT1E1), mRNA [NM_005420]	**-3.051**
ZNF479	Homo sapiens zinc finger protein 479 (ZNF479), mRNA [NM_033273]	**-3.045**
LOC653071	Homo sapiens similar to CG32820-PA, isoform A, mRNA (cDNA clone IMAGE:4812880), [BC068588]	**-3.032**
LOC100128519	Homo sapiens misc_RNA (LOC100128519), miscRNA [XR_038473]	**-2.967**
HSPD1P5	Homo sapiens misc_RNA (LOC345041), miscRNA [XR_018747]	**-2.920**
ARNT2	Homo sapiens aryl-hydrocarbon receptor nuclear translocator 2 (ARNT2), mRNA [NM_014862]	**-2.776**
SNHG10	Homo sapiens small nucleolar RNA host gene 10 (non-protein coding) (SNHG10), non-coding RNA [NR_003138]	**-2.772**
ZNF43	Homo sapiens zinc finger protein 43 (ZNF43), mRNA [NM_003423]	**-2.761**
LOC343495	Homo sapiens misc_RNA (LOC343495), miscRNA [XR_016540]	**-2.733**
LOC646446	Homo sapiens similar to hCG2040301 (LOC646446), mRNA [XM_001722653]	**-2.726**
ZNF737	Homo sapiens zinc finger protein 737, mRNA (cDNA clone IMAGE:4854518), [BC015765]	**-2.708**
LOC100132658	Homo sapiens misc_RNA (LOC100132658), miscRNA [XR_038952]	**-2.674**
ID1	Homo sapiens inhibitor of DNA binding 1, dominant negative helix-loop-helix protein (ID1), mRNA [NM_002165]	**-2.673**
LOC392382	Homo sapiens misc_RNA (LOC392382), miscRNA [XR_019110]	**-2.648**
LOC100131883	Homo sapiens similar to PRO2751 (LOC100131883), mRNA [XM_001714201]	**-2.642**
KIAA0492	Homo sapiens mRNA, chromosome 1 specific transcript KIAA0492. [AB007961]	**-2.622**
ZNF676	Homo sapiens zinc finger protein 676 (ZNF676), mRNA [NM_001001411]	**-2.616**
LOC389992	Homo sapiens similar to hCG2040259 (LOC389992), mRNA [XM_001720568]	**-2.597**
LOC441666	Homo sapiens zinc finger protein 91 pseudogene (LOC441666), non-coding RNA [NR_024380]	**-2.590**
LOC100130518	Zinc finger protein ENSP00000350085 [Source:UniProtKB/Swiss-Prot;Acc: A6NN14] [ENST00000357491]	**-2.575**
LOC643015	Homo sapiens misc_RNA (LOC643015), miscRNA [XR_018290]	**-2.570**
LOC728344	Homo sapiens misc_RNA (LOC728344), miscRNA [XR_018424]	**-2.557**
LOC203510	Homo sapiens similar to hCG1644442 (LOC203510), mRNA [XM_001719132]	**-2.524**

EFCAB10	Homo sapiens cDNA clone IMAGE:6616931, partial cds. [BC062748]	**-2.506**
C10orf140	Homo sapiens chromosome 10 open reading frame 140 (C10orf140), mRNA [NM_207371]	**-2.500**
ZNF509	Homo sapiens zinc finger protein 509 (ZNF509), mRNA [NM_145291]	**-2.490**
HERC3	Homo sapiens hect domain and RLD 3, mRNA (cDNA clone IMAGE:6050308), [BC038960]	**-2.476**
DLC1	Homo sapiens deleted in liver cancer 1 (DLC1), mRNA [NM_182643]	**-2.473**
GTF2A1	Homo sapiens general transcription factor IIA, 1, 19/37kDa (GTF2A1), mRNA [NM_015859]	**-2.458**
ARVCF	Homo sapiens armadillo repeat gene deletes in velocardiofacial syndrome (ARVCF), mRNA [NM_001670]	**-2.454**
LOC100128701	Homo sapiens misc_RNA (LOC100128701), miscRNA [XR_037462]	**-2.428**
LOC653773	Homo sapiens misc_RNA (LOC653773), partial miscRNA [XR_042355]	**-2.422**
PLCXD3	Homo sapiens phosphatidylinositol-specific phospholipase C, X domain containing 3 (PLCXD3), mRNA [NM_001005473]	**-2.403**
MAGEA1	Homo sapiens melanoma antigen family A, 1 (directs expression of antigen MZ2-E) (MAGEA1), mRNA [NM_004988]	**-2.361**
IGFBP5	Homo sapiens insulin-like growth factor binding protein 5 (IGFBP5), mRNA [NM_000599]	**-2.339**
DAGLA	Homo sapiens diacylglycerol lipase, alpha (DAGLA), mRNA [NM_006133]	**-2.337**
LOC728774	Homo sapiens similar to hCG1994130 (LOC728774), mRNA [XM_001129390]	**-2.337**
HSP90AB6P	Homo sapiens heat shock protein 90Bf (HSP90Bf) mRNA [AY956767]	**-2.329**
UBE2D3P	Homo sapiens similar to Putative ubiquitin-conjugating enzyme E2 D3-like protein (LOC100128647), mRNA [XM_001725173]	**-2.326**
LOC645231	Full-length cDNA clone CS0DI026YJ08 of Placenta Cot 25-normalized of Homo sapiens (human). [CR590757]	**-2.322**
LOC283523	Homo sapiens similar to telomeric repeat binding factor (NIMA-interacting) 1 (LOC283523), mRNA [XM_001714856]	**-2.316**
LOC100132086	Homo sapiens misc_RNA (LOC100132086), miscRNA [XR_037769]	**-2.297**
LOC729332	Homo sapiens hypothetical LOC729332 (LOC729332), mRNA [XM_001129827]	**-2.285**
C5orf36	Homo sapiens chromosome 5 open reading frame 36 (C5orf36), mRNA [NM_173665]	**-2.267**
HIST1H3J	Homo sapiens histone cluster 1, H3j (HIST1H3J), mRNA [NM_003535]	**-2.265**
ZNF208	Homo sapiens zinc finger protein 208 (ZNF208), mRNA [NM_007153]	**-2.246**
LOC100130171	Homo sapiens misc_RNA (LOC100130171), miscRNA [XR_038676]	**-2.243**
PLA2R1	Human 180 kDa transmembrane PLA2 receptor mRNA. [U17033]	**-2.230**
LOC728927	Homo sapiens cDNA FLJ57041 complete cds, moderately similar to Zinc finger protein 92. [AK301806]	**-2.228**
CBWD6	Homo sapiens clone 1659351 unknown mRNA. [AF293368]	**-2.207**
tcag7.873	Homo sapiens hypothetical LOC402644 (LOC402644), mRNA [NM_001126493]	**-2.185**
LOC392335	Homo sapiens misc_RNA (LOC392335), miscRNA [XR_037043]	**-2.181**
FKSG2	Homo sapiens apoptosis inhibitor (FKSG2), mRNA [NM_021631]	**-2.179**
RPS6P1	Homo sapiens misc_RNA (RPS6P1), miscRNA [XR_016837]	**-2.174**

FAM179A	Homo sapiens family with sequence similarity 179, member A (FAM179A), mRNA [NM_199280]	**-2.160**
LOC100128266	Homo sapiens misc_RNA (LOC100128266), miscRNA [XR_037888]	**-2.157**
MICALCL	Homo sapiens MICAL C-terminal like (MICALCL), mRNA [NM_032867]	**-2.147**
PDGFRB	Homo sapiens platelet-derived growth factor receptor, beta polypeptide (PDGFRB), mRNA [NM_002609]	**-2.142**
LFNG	Homo sapiens LFNG O-fucosylpeptide 3-beta-N-acetylglucosaminyltransferase (LFNG), mRNA [NM_001040167]	**-2.134**
ZNF730	Homo sapiens cDNA FLJ16640 fis, clone TESTI4028938, moderately similar to Zinc finger protein 85. [AK131472]	**-2.133**
DLX2	Homo sapiens distal-less homeobox 2 (DLX2), mRNA [NM_004405]	**-2.128**
TCF7L2	Homo sapiens transcription factor 7-like 2 (T-cell specific, HMG-box) (TCF7L2), mRNA [NM_030756]	**-2.127**
LOC390413	Homo sapiens misc_RNA (LOC390413), miscRNA [XR_018341]	**-2.125**
SAR1P3	Homo sapiens SAR1 gene homolog (S. cerevisiae) pseudogene 3 (SAR1P3), mRNA [XM_001714154]	**-2.119**
LOC388532	Homo sapiens hypothetical LOC388532 (LOC388532), mRNA [XM_001721671]	**-2.114**
PABPC3	Homo sapiens poly(A) binding protein, cytoplasmic 3 (PABPC3), mRNA [NM_030979]	**-2.089**
GOLGA8E	Homo sapiens golgi autoantigen, golgin subfamily a, 8E (GOLGA8E), mRNA [NM_001012423]	**-2.086**
GSPT2	Homo sapiens G1 to S phase transition 2 (GSPT2), mRNA [NM_018094]	**-2.083**
LOC100131323	Homo sapiens misc_RNA (LOC100131323), miscRNA [XR_039461]	**-2.069**
LOC389842	Homo sapiens similar to RanBP1 (LOC389842), mRNA [XM_372200]	**-2.062**
LOC643981	Homo sapiens misc_RNA (LOC643981), miscRNA [XR_018444]	**-2.046**
hCG_1771830	Homo sapiens zinc finger protein LOC654254 (LOC654254), mRNA [NM_001137608]	**-2.044**
IL17RB	Homo sapiens interleukin 17 receptor B (IL17RB), mRNA [NM_018725]	**-2.044**
KIAA0802	Homo sapiens KIAA0802 (KIAA0802), mRNA [NM_015210]	**-2.011**
MYSM1	Homo sapiens mRNA for KIAA1915 protein. [AB067502]	**-1.987**
TNFRSF25	Homo sapiens tumor necrosis factor receptor superfamily, member 25 (TNFRSF25), mRNA [NM_148965]	**-1.987**
C14orf143	Homo sapiens mRNA; cDNA DKFZp547I1415 (from clone DKFZp547I1415). [AL832321]	**-1.969**
NACAP1	Homo sapiens nascent-polypeptide-associated complex alpha polypeptide pseudogene 1 (NACAP1), non-coding RNA [NR_002182]	**-1.962**
GPX1	Homo sapiens glutathione peroxidase 1 (GPX1), transcript variant 2, mRNA [NM_201397]	**-1.957**
INTS2	Homo sapiens integrator complex subunit 2 (INTS2), mRNA [NM_020748]	**-1.954**
SPATA9	Homo sapiens cDNA FLJ35906 fis, clone TESTI2009727. [AK093225]	**-1.946**
HSD11B2	Homo sapiens hydroxysteroid (11-beta) dehydrogenase 2 (HSD11B2), mRNA [NM_000196]	**-1.944**
ZNF85	Homo sapiens zinc finger protein 85 (ZNF85), mRNA [NM_003429]	**-1.935**

LOC730834	DB090170 TESTI4 Homo sapiens cDNA clone TESTI4038997 5', mRNA sequence [DB090170]	**-1.923**
ZNF141	Homo sapiens zinc finger protein 141 (ZNF141), mRNA [NM_003441]	**-1.913**
KRT19P2	Homo sapiens mRNA for keratin 19, partial cds, isolate:K19-141. [AB041269]	**-1.911**
LMNB2	Homo sapiens lamin B2 (LMNB2), mRNA [NM_032737]	**-1.909**
ADRA2A	Homo sapiens adrenergic, alpha-2A-, receptor (ADRA2A), mRNA [NM_000681]	**-1.904**
ZCRB1	Homo sapiens zinc finger CCHC-type and RNA binding motif 1 (ZCRB1), mRNA [NM_033114]	**-1.903**
ZNF665	Homo sapiens zinc finger protein 665 (ZNF665), mRNA [NM_024733]	**-1.899**
TRIM66	Homo sapiens tripartite motif-containing 66 (TRIM66), mRNA [NM_014818]	**-1.898**
DFFB	Homo sapiens DNA fragmentation factor, 40kDa, beta polypeptide (caspase-activated DNase) (DFFB), mRNA [NM_004402]	**-1.892**
LOC100129743	Homo sapiens similar to hCG2042936 (LOC100129743), mRNA [XM_001719980]	**-1.886**
TXNIP	Homo sapiens thioredoxin interacting protein (TXNIP), mRNA [NM_006472]	**-1.882**
LOC442459	Homo sapiens X-ray repair complementing defective repair pseudogene (LOC442459), non-coding RNA [NR_024608]	**-1.877**
TCEB1P3	Homo sapiens transcription elongation factor B (SIII), polypeptide 1 (15kDa, elongin C) pseudogene 3 (TCEB1P3), mRNA [XM_927664]	**-1.877**
ZNF486	Homo sapiens zinc finger protein 486 (ZNF486), mRNA [NM_052852]	**-1.874**
SUMO1P3	Homo sapiens SUMO1 pseudogene 3 (SUMO1P3), non-coding RNA [NR_002190]	**-1.870**
SMN2	Homo sapiens survival of motor neuron 2, centromeric (SMN2) mRNA [NM_022877]	**-1.855**
LOC391282	Homo sapiens similar to ribosomal protein L23a (LOC391282), mRNA [XM_372878]	**-1.851**
ISL1	Homo sapiens ISL LIM homeobox 1 (ISL1), mRNA [NM_002202]	**-1.848**
LOC100130211	Homo sapiens similar to translation elongation factor 1 alpha 1-like 14 (LOC100130211), mRNA [XM_001717632]	**-1.842**
TTC14	Homo sapiens tetratricopeptide repeat domain 14 (TTC14), mRNA [NM_001042601]	**-1.839**
RPL32P3	Homo sapiens ribosomal protein L32 pseudogene 3 (RPL32P3), non-coding RNA [NR_003111]	**-1.831**
LOC646973	Homo sapiens similar to eukaryotic translation elongation factor 1 beta 2 (LOC646973), mRNA [XR_019410]	**-1.825**
RAB33B	Homo sapiens RAB33B, member RAS oncogene family (RAB33B), mRNA [NM_031296]	**-1.822**
TBC1D3B	Homo sapiens TBC1 domain family, member 3B (TBC1D3B), mRNA [NM_001001417]	**-1.819**
CCDC74B	Homo sapiens coiled-coil domain containing 74B (CCDC74B), mRNA [NM_207310]	**-1.815**
CHRNA9	Homo sapiens cholinergic receptor, nicotinic, alpha 9 (CHRNA9), mRNA [NM_017581]	**-1.808**

RPS12	Homo sapiens ribosomal protein S12 (RPS12), mRNA [NM_001016]	**-1.806**
LOC376693	Homo sapiens hypothetical LOC376693, mRNA (cDNA clone MGC:45392 IMAGE:5526694), [BC030568]	**-1.804**
PARP4	Homo sapiens poly (ADP-ribose) polymerase family, member 4 (PARP4), mRNA [NM_006437]	**-1.804**
LOC100131581	Homo sapiens cDNA FLJ35225 fis, clone PROST2001116. [AK092544]	**-1.802**
C15orf31	Human pre-T/NK cell associated protein (6H9A) mRNA, complete cds. [L17330]	**-1.797**
ZNF345	Homo sapiens zinc finger protein 345 (ZNF345), mRNA [NM_003419]	**-1.797**
LOC646909	Homo sapiens misc_RNA (LOC646909), miscRNA [XR_019013]	**-1.795**
LOC400750	Homo sapiens misc_RNA (LOC400750), miscRNA [XR_039070]	**-1.794**
LOC100130794	Homo sapiens similar to NADH dehydrogenase [ubiquinone] iron-sulfur protein 5 (NADH-ubiquinone oxidoreductase 15 kDa subunit) (LOC100130794), mRNA [XM_001724177]	**-1.792**
LOC390876	Homo sapiens similar to ribosomal protein L35 (LOC390876), mRNA [XM_001718967]	**-1.786**
LOC400061	Homo sapiens misc_RNA (LOC400061), miscRNA [XR_019557]	**-1.780**
SLAIN1	Homo sapiens SLAIN motif family, member 1 (SLAIN1), transcript variant 1, mRNA [NM_001040153]	**-1.780**
UNC5B	Homo sapiens unc-5 homolog B (C. elegans) (UNC5B), mRNA [NM_170744]	**-1.779**
ITM2B	Homo sapiens integral membrane protein 2B (ITM2B), mRNA [NM_021999]	**-1.773**
BAG4	BAG family molecular chaperone regulator 4 (BAG-4) [Source:UniProtKB/Swiss-Prot;Acc:O95429] [ENST00000287322]	**-1.771**
B3GAT2	Homo sapiens beta-1,3-glucuronyltransferase 2 (B3GAT2), mRNA [NM_080742]	**-1.770**
LOC130728	Homo sapiens misc_RNA (LOC130728), miscRNA [XR_019248]	**-1.758**
NOX4	Homo sapiens NADPH oxidase 4 (NOX4), mRNA [NM_016931]	**-1.755**
MGC3207	Homo sapiens translation initiation factor eIF-2B subunit alpha/beta/delta-like protein (MGC3207), mRNA [NM_001031727]	**-1.751**
ZNF728	Homo sapiens similar to Zinc finger protein 208 (LOC388523), mRNA [XM_001726961]	**-1.740**
LOC643205	Homo sapiens hypothetical LOC643205 (LOC643205), mRNA [XM_001716733]	**-1.738**
PTBP1	Homo sapiens polypyrimidine tract binding protein 1 (PTBP1), mRNA [NM_002819]	**-1.730**
ABCA10	Homo sapiens ATP-binding cassette, sub-family A (ABC1), member 10 (ABCA10), mRNA [NM_080282]	**-1.727**
C18orf32	Homo sapiens chromosome 18 open reading frame 32 (C18orf32), mRNA [NM_001035005]	**-1.727**
ESF1	Homo sapiens ESF1, nucleolar pre-rRNA processing protein, homolog (S. cerevisiae) (ESF1), mRNA [NM_016649]	**-1.726**
CENPK	Homo sapiens centromere protein K (CENPK), mRNA [NM_022145]	**-1.724**
WWC2	Homo sapiens WW and C2 domain containing 2 (WWC2), mRNA [NM_024949]	**-1.721**
PRR11	Homo sapiens cDNA FLJ11029 fis, clone PLACE1004156. [AK001891]	**-1.715**
PL-5283	Homo sapiens PL-5283 protein (PL-5283), mRNA [NM_001130929]	**-1.705**
KIF18B	Homo sapiens hypothetical protein LOC146909, mRNA (cDNA clone	**-1.704**

	IMAGE:4418755), [BC048263]	
ZNF680	Homo sapiens zinc finger protein 680 (ZNF680), mRNA [NM_178558]	**-1.704**
ANO8	Homo sapiens anoctamin 8 (ANO8), mRNA [NM_020959]	**-1.702**
TRIM15	Homo sapiens tripartite motif-containing 15 (TRIM15), mRNA [NM_033229]	**-1.700**
SNRPG	Homo sapiens small nuclear ribonucleoprotein polypeptide G (SNRPG), mRNA [NM_003096]	**-1.697**
ZNF675	Homo sapiens zinc finger protein 675 (ZNF675), mRNA [NM_138330]	**-1.697**
LOC730232	Homo sapiens misc_RNA (LOC730232), miscRNA [XR_016075]	**-1.692**
ZNF826	Homo sapiens zinc finger protein 826 (ZNF826), mRNA [NM_001039884]	**-1.690**
LOC100128974	Homo sapiens misc_RNA (LOC100128974), miscRNA [XR_037045]	**-1.689**
IQGAP2	Homo sapiens IQ motif containing GTPase activating protein 2 (IQGAP2), mRNA [NM_006633]	**-1.679**
FAM13C1	Homo sapiens family with sequence similarity 13, member C1 (FAM13C1), mRNA [NM_001001971]	**-1.677**
KANK4	Homo sapiens KN motif and ankyrin repeat domains 4 (KANK4), mRNA [NM_181712]	**-1.670**
LOC100128626	Homo sapiens misc_RNA (LOC100128626), miscRNA [XR_038662]	**-1.666**
LOC100132218	Homo sapiens hypothetical protein LOC100132218 (LOC100132218), mRNA [XM_001715393]	**-1.666**
GPER	Homo sapiens G protein-coupled estrogen receptor 1 (GPER), mRNA [NM_001039966]	**-1.661**
LOC729684	Homo sapiens misc_RNA (LOC729684), miscRNA [XR_039360]	**-1.658**
TFPI2	Homo sapiens tissue factor pathway inhibitor 2 (TFPI2), mRNA [NM_006528]	**-1.658**
ZNF117	Homo sapiens zinc finger protein 117 (ZNF117), mRNA [NM_015852]	**-1.656**
FAM175B	Homo sapiens family with sequence similarity 175, member B (FAM175B), mRNA [NM_032182]	**-1.650**
hCG_18290	Homo sapiens hCG18290 (LOC644907), mRNA [NM_001090027]	**-1.650**
LOC730167	Homo sapiens similar to protein tyrosine phosphatase 4a1 (LOC730167), mRNA [XM_001134097]	**-1.648**
ITGA11	Homo sapiens integrin, alpha 11 (ITGA11), mRNA [NM_001004439]	**-1.647**
PMCH	Homo sapiens pro-melanin-concentrating hormone (PMCH), mRNA [NM_002674]	**-1.644**
SLCO1C1	Homo sapiens solute carrier organic anion transporter family, member 1C1 (SLCO1C1), mRNA [NM_017435]	**-1.644**
KIAA1524	Homo sapiens KIAA1524 (KIAA1524), mRNA [NM_020890]	**-1.643**
NSBP1	Homo sapiens nucleosomal binding protein 1 (NSBP1), mRNA [NM_030763]	**-1.642**
TRPM6	Homo sapiens transient receptor potential cation channel, subfamily M, member 6 (TRPM6), mRNA [NM_017662]	**-1.638**
AFAP1L2	Homo sapiens actin filament associated protein 1-like 2 (AFAP1L2), mRNA [NM_032550]	**-1.628**
CHL1	Homo sapiens cell adhesion molecule with homology to L1CAM (close homolog of L1) (CHL1), mRNA [NM_006614]	**-1.626**
FRG1B	Homo sapiens FSHD region gene 1 family, member B (FRG1B), non-coding RNA	**-1.620**

	[NR_003579]	
RPL31P10	Homo sapiens misc_RNA (RPL31P10), miscRNA [XR_018695]	**-1.620**
LOC90834	Homo sapiens, clone IMAGE:3535910, mRNA, partial cds. [BC001742]	**-1.619**
NUDT12	Homo sapiens nudix (nucleoside diphosphate linked moiety X)-type motif 12 (NUDT12), mRNA [NM_031438]	**-1.618**
RBP1	Homo sapiens retinol binding protein 1, cellular (RBP1), mRNA [NM_002899]	**-1.618**
ZNF850P	Homo sapiens mRNA; cDNA DKFZp686J154 (from clone DKFZp686J154). [CR627133]	**-1.616**
LOC730107	Homo sapiens similar to Glycine cleavage system H protein, mitochondrial (LOC730107), mRNA [XM_001721064]	**-1.614**
EDN2	Homo sapiens endothelin 2 (EDN2), mRNA [NM_001956]	**-1.610**
LOC100131737	Homo sapiens misc_RNA (LOC100131737), miscRNA [XR_038332]	**-1.608**
LOC100132439	Homo sapiens similar to Protein FAM27E3 (LOC100132439), mRNA [XM_001719283]	**-1.603**
FMC1	Homo sapiens formation of mitochondrial complexes 1 homolog (S. cerevisiae) (FMC1), mRNA [NM_197964]	**-1.598**
EIF1AY	Homo sapiens eukaryotic translation initiation factor 1A, Y-linked (EIF1AY), mRNA [NM_004681]	**-1.589**
TMEM129	Homo sapiens transmembrane protein 129 (TMEM129), mRNA [NM_138385]	**-1.587**
C18orf55	Homo sapiens chromosome 18 open reading frame 55 (C18orf55), mRNA [NM_014177]	**-1.585**
LOC729983	Homo sapiens hypothetical LOC729983 (LOC729983), mRNA [XM_001718065]	**-1.584**
ANP32C	Homo sapiens acidic (leucine-rich) nuclear phosphoprotein 32 family, member C (ANP32C), mRNA [NM_012403]	**-1.580**
GPM6B	Homo sapiens glycoprotein M6B (GPM6B), mRNA [NM_001001996]	**-1.576**
LOC442160	Homo sapiens misc_RNA (LOC442160), miscRNA [XR_042339]	**-1.575**
POLB	Homo sapiens polymerase (DNA directed), beta (POLB), mRNA [NM_002690]	**-1.572**
COL9A3	Homo sapiens collagen, type IX, alpha 3 (COL9A3), mRNA [NM_001853]	**-1.571**
ODAM	Homo sapiens odontogenic, ameloblast asssociated (ODAM), mRNA [NM_017855]	**-1.564**
C9orf140	Homo sapiens chromosome 9 open reading frame 140 (C9orf140), mRNA [NM_178448]	**-1.557**
LOC100130289	Homo sapiens misc_RNA (LOC100130289), miscRNA [XR_039183]	**-1.557**
RHOBTB3	Homo sapiens Rho-related BTB domain containing 3 (RHOBTB3), mRNA [NM_014899]	**-1.555**
POSTN	Homo sapiens periostin, osteoblast specific factor (POSTN), , mRNA [NM_006475]	**-1.554**
LOC100129742	Homo sapiens misc_RNA (LOC100129742), miscRNA [XR_036968]	**-1.550**
ZNF594	Homo sapiens zinc finger protein 594 (ZNF594), mRNA [NM_032530]	**-1.547**
AK2P2	Homo sapiens adenylate kinase 2 pseudogene 2 (AK2P2), mRNA [XR_017211]	**-1.545**
E2F8	Homo sapiens E2F transcription factor 8 (E2F8), mRNA [NM_024680]	**-1.541**
LOC100131149	Homo sapiens misc_RNA (LOC100131149), miscRNA [XR_039101]	**-1.535**
ZNF569	Homo sapiens zinc finger protein 569 (ZNF569), mRNA [NM_152484]	**-1.532**

C9orf41	UPF0586 protein C9orf41 [Source:UniProtKB/Swiss-Prot;Acc:Q8N4J0] [ENST00000376834]	**-1.530**
LOC644384	Full-length cDNA clone CS0DI028YD16 of Placenta Cot 25-normalized of Homo sapiens (human). [CR595167]	**-1.530**
FLJ39609	Homo sapiens cDNA FLJ39609 fis, clone SKNSH2008043. [AK096928]	**-1.529**
LOC100128328	Homo sapiens hypothetical protein LOC100128328 (LOC100128328), mRNA [XM_001715053]	**-1.528**
ABCD4	Homo sapiens ATP-binding cassette, sub-family D (ALD), member 4 (ABCD4), mRNA [NM_005050]	**-1.526**
LOC642236	Homo sapiens mRNA; cDNA DKFZp686M08106 (from clone DKFZp686M08106). [CR933606]	**-1.525**
ANXA2P3	Homo sapiens annexin A2 pseudogene 3 (ANXA2P3), non-coding RNA [NR_001446]	**-1.521**
LOC646993	Homo sapiens similar to high-mobility group box 3 (LOC646993), mRNA [XM_929965]	**-1.516**
SNRPN	Homo sapiens clone Rt-16 SNURF-SNRPN mRNA, downstream untranslated exons, alternatively spliced. [AF400500]	**-1.512**
CROP	Homo sapiens cisplatin resistance-associated overexpressed protein (CROP), mRNA [NM_006107]	**-1.509**
LOC641844	Homo sapiens misc_RNA (LOC641844), miscRNA [XR_018036]	**-1.509**
C18orf56	Homo sapiens chromosome 18 open reading frame 56 (C18orf56), mRNA [NM_001012716]	**-1.507**
LOC645294	Homo sapiens misc_RNA (LOC645294), miscRNA [XR_019042]	**-1.507**
LOC257039	Homo sapiens similar to hCG2040268 (LOC257039), mRNA [XM_172230]	**-1.504**
ANKRD50	Homo sapiens ankyrin repeat domain 50 (ANKRD50), mRNA [NM_020337]	**-1.503**
KCTD16	Homo sapiens potassium channel tetramerisation domain containing 16 (KCTD16), mRNA [NM_020768]	**-1.503**
SVIP	Homo sapiens small VCP/p97-interacting protein (SVIP), mRNA [NM_148893]	**-1.495**
LOC729046	Homo sapiens misc_RNA (LOC729046), miscRNA [XR_015710]	**-1.493**
ZNF319	Homo sapiens zinc finger protein 319 (ZNF319), mRNA [NM_020807]	**-1.492**
AKAP5	Homo sapiens A kinase (PRKA) anchor protein 5 (AKAP5), mRNA [NM_004857]	**-1.491**
BEND3	Homo sapiens BEN domain containing 3 (BEND3), mRNA [NM_001080450]	**-1.488**
INE1	Homo sapiens inactivation escape 1 (non-protein coding) (INE1), non-coding RNA [NR_024616]	**-1.488**
LOC285550	Homo sapiens cDNA FLJ42660 fis, clone BRAMY2010808. [AK124651]	**-1.482**
C11orf31	Homo sapiens chromosome 11 open reading frame 31 (C11orf31), mRNA [NM_170746]	**-1.474**
ZNF626	Homo sapiens zinc finger protein 626 (ZNF626), mRNA [NM_001076675]	**-1.474**
KRT18P46	Homo sapiens keratin 18 pseudogene 46 (KRT18P46), mRNA [XM_001720107]	**-1.472**
DHX40	Homo sapiens DEAH (Asp-Glu-Ala-His) box polypeptide 40 (DHX40), mRNA [NM_024612]	**-1.467**
TMC8	Homo sapiens transmembrane channel-like 8 (TMC8), mRNA [NM_152468]	**-1.466**

C15orf37	Homo sapiens chromosome 15 open reading frame 37 (C15orf37), mRNA [NM_175898]	**-1.464**
COL15A1	Homo sapiens collagen, type XV, alpha 1 (COL15A1), mRNA [NM_001855]	**-1.463**
FLYWCH2	Homo sapiens FLYWCH family member 2 (FLYWCH2), mRNA [NM_138439]	**-1.463**
C3orf64	Homo sapiens chromosome 3 open reading frame 64 (C3orf64), mRNA [NM_173654]	**-1.462**
KRT18P40	Homo sapiens misc_RNA (KRT18P40), miscRNA [XR_017288]	**-1.458**
C8orf38	Homo sapiens cDNA FLJ23887 fis, clone LNG14332. [AK074467]	**-1.457**
NPTX1	Homo sapiens neuronal pentraxin I (NPTX1), mRNA [NM_002522]	**-1.457**
LOC440737	Homo sapiens similar to ribosomal protein L35 (LOC440737), mRNA [XM_496446]	**-1.455**
C1orf133	Homo sapiens chromosome 1 open reading frame 133 (C1orf133), non-coding RNA [NR_024337]	**-1.454**
SMA4	Homo sapiens glucuronidase, beta pseudogene 1, mRNA (cDNA clone IMAGE:4824349). [BC035411]	**-1.454**
LOC643014	Homo sapiens misc_RNA (LOC643014), miscRNA [XR_018450]	**-1.453**
SRP14P1	Homo sapiens signal recognition particle 14kDa (homologous Alu RNA binding protein) pseudogene 1 (SRP14P1), non-coding RNA [NR_003273]	**-1.452**
SSBP1	Homo sapiens single-stranded DNA binding protein 1 (SSBP1), mRNA [NM_003143]	**-1.451**
LOC100128203	Homo sapiens similar to hCG2040272 (LOC100128203), mRNA [XM_001715724]	**-1.449**
LOC400657	Homo sapiens hypothetical LOC400657 (LOC400657), non-coding RNA [NR_024484]	**-1.448**
GOLIM4	Homo sapiens golgi integral membrane protein 4 (GOLIM4), mRNA [NM_014498]	**-1.445**
MYLK2	Homo sapiens myosin light chain kinase 2 (MYLK2), mRNA [NM_033118]	**-1.444**
tcag7.1239	Homo sapiens misc_RNA (LOC643438), miscRNA [XR_015268]	**-1.442**
ZNF429	Homo sapiens zinc finger protein 429 (ZNF429), mRNA [NM_001001415]	**-1.441**
C12orf24	Homo sapiens chromosome 12 open reading frame 24 (C12orf24), mRNA [NM_013300]	**-1.435**
LOC100129397	Homo sapiens cDNA FLJ38522 fis, clone HCHON2000818. [AK095841]	**-1.435**
SPINK5L3	Homo sapiens serine PI Kazal type 5-like 3 (SPINK5L3), mRNA [NM_001040129]	**-1.435**
LRP5L	Homo sapiens low density lipoprotein receptor-related protein 5-like (LRP5L), mRNA [NM_182492]	**-1.431**
LOC646999	Homo sapiens hypothetical LOC646999 (LOC646999), non-coding RNA [NR_024390]	**-1.430**
LOC254057	Homo sapiens cDNA: FLJ21000 fis, clone CAE03359. [AK024653]	**-1.427**
KRT83	Homo sapiens keratin 83 (KRT83), mRNA [NM_002282]	**-1.425**
HIST1H3E	Homo sapiens histone cluster 1, H3e (HIST1H3E), mRNA [NM_003532]	**-1.424**
EPHA7	Homo sapiens EPH receptor A7 (EPHA7), mRNA [NM_004440]	**-1.424**
C15orf23	Homo sapiens chromosome 15 open reading frame 23 (C15orf23), mRNA	**-1.421**

	[NM_001142761]	
MGC16121	Homo sapiens hypothetical protein MGC16121, mRNA (cDNA clone IMAGE:3627113), [BC007360]	**-1.418**
CENPV	Homo sapiens centromere protein V (CENPV), mRNA [NM_181716]	**-1.416**
CCNA1	Homo sapiens cyclin A1 (CCNA1), mRNA [NM_003914]	**-1.415**
ARRDC3	Homo sapiens arrestin domain containing 3 (ARRDC3), mRNA [NM_020801]	**-1.412**
LOC440396	Homo sapiens similar to Heterogeneous nuclear ribonucleoprotein A1 (hnRNP core protein A1) (HDP-1) (LOC440396), non-coding RNA [NR_002943]	**-1.411**
AL022344.6	Homo sapiens similar to hCG1640833 (LOC100129622), mRNA [XM_001724175]	**-1.409**
THAP2	Homo sapiens THAP domain containing, apoptosis associated protein 2 (THAP2), mRNA [NM_031435]	**-1.408**
LOC100125556	Homo sapiens family with sequence similarity 86, member A pseudogene (LOC100125556), non-coding RNA [NR_024251]	**-1.405**
LGR6	Homo sapiens leucine-rich repeat-containing G protein-coupled receptor 6 (LGR6), mRNA [NM_001017403]	**-1.405**
KIAA0831	Homo sapiens KIAA0831 (KIAA0831), mRNA [NM_014924]	**-1.403**
STX2	Homo sapiens syntaxin 2 (STX2), mRNA [NM_001980]	**-1.400**
NOX1	Homo sapiens NADPH oxidase 1 (NOX1), mRNA [NM_007052]	**-1.399**
SFPQ	Homo sapiens splicing factor proline/glutamine-rich (SFPQ), mRNA [NM_005066]	**-1.398**
CAPZA2	Homo sapiens capping protein (actin filament) muscle Z-line, alpha 2 (CAPZA2), mRNA [NM_006136]	**-1.397**
FGF18	Homo sapiens fibroblast growth factor 18 (FGF18), mRNA [NM_003862]	**-1.396**
LRRC37A3	Homo sapiens leucine rich repeat containing 37, member A3 (LRRC37A3), mRNA [NM_199340]	**-1.396**
RASGRP1	Homo sapiens RAS guanyl releasing protein 1 (calcium and DAG-regulated) (RASGRP1), mRNA [NM_005739]	**-1.390**
RPL31P4	Homo sapiens misc_RNA (LOC729646), miscRNA [XR_037308]	**-1.387**
USP38	Homo sapiens ubiquitin specific peptidase 38 (USP38), mRNA [NM_032557]	**-1.385**
ZNF556	Homo sapiens zinc finger protein 556 (ZNF556), mRNA [NM_024967]	**-1.384**
NSL1	Homo sapiens NSL1, MIND kinetochore complex component, homolog (S. cerevisiae) (NSL1), mRNA [NM_015471]	**-1.382**
PLAGL2	Homo sapiens pleiomorphic adenoma gene-like 2 (PLAGL2), mRNA [NM_002657]	**-1.382**
USP11	Homo sapiens ubiquitin specific peptidase 11 (USP11), mRNA [NM_004651]	**-1.382**
ZNF69	Homo sapiens zinc finger protein 69 (ZNF69), mRNA [NM_021915]	**-1.382**
IPPK	Homo sapiens inositol 1,3,4,5,6-pentakisphosphate 2-kinase (IPPK), mRNA [NM_022755]	**-1.380**
LOC100132816	Homo sapiens hypothetical protein LOC100132816 (LOC100132816), mRNA [XM_001718703]	**-1.377**
ZBTB1	Homo sapiens zinc finger and BTB domain containing 1 (ZBTB1), mRNA [NM_014950]	**-1.376**

ACAA2	Homo sapiens acetyl-Coenzyme A acyltransferase 2 (ACAA2), nuclear gene encoding mitochondrial protein, mRNA [NM_006111]	**-1.372**
CCDC34	Homo sapiens coiled-coil domain containing 34 (CCDC34), mRNA [NM_080654]	**-1.368**
LOC646119	Homo sapiens similar to hCG2040247 (LOC646119), mRNA [XM_929084]	**-1.368**
LOC340508	Homo sapiens hypothetical protein LOC340508 (LOC340508), non-coding RNA [NR_002942]	**-1.367**
LOC100129387	Homo sapiens hypothetical LOC100129387 (LOC100129387), non-coding RNA [NR_024490]	**-1.363**
KRT13	Homo sapiens keratin 13 (KRT13), mRNA [NM_002274]	**-1.362**
NAT8L	Homo sapiens N-acetyltransferase 8-like (GCN5-related, putative) (NAT8L), mRNA [NM_178557]	**-1.362**
RECQL	Homo sapiens RecQ protein-like (DNA helicase Q1-like) (RECQL), mRNA [NM_032941]	**-1.360**
MNAT1	Homo sapiens menage a trois homolog 1, cyclin H assembly factor (Xenopus laevis) (MNAT1), mRNA [NM_002431]	**-1.359**
EIF1B	Homo sapiens eukaryotic translation initiation factor 1B (EIF1B), mRNA [NM_005875]	**-1.355**
PTTG3	Homo sapiens pituitary tumor-transforming 3 (PTTG3), non-coding RNA [NR_002734]	**-1.351**
FGFBP3	Homo sapiens fibroblast growth factor binding protein 3 (FGFBP3), mRNA [NM_152429]	**-1.349**
ZNF107	Homo sapiens zinc finger protein 107 (ZNF107), mRNA [NM_016220]	**-1.345**
KIAA1466	Homo sapiens mRNA for KIAA1466 protein, partial cds. [AB040899]	**-1.343**
LOC100131206	Homo sapiens similar to hCG1794073 (LOC100131206), mRNA [XM_001718536]	**-1.340**
NAP1L1	Homo sapiens nucleosome assembly protein 1-like 1 (NAP1L1), mRNA [NM_004537]	**-1.339**
DKFZp313P036	Homo sapiens mRNA; cDNA DKFZp313P036 (from clone DKFZp313P036). [BX537874]	**-1.339**
SMAD9	Mothers against decapentaplegic homolog 9 (Mothers against DPP homolog 9) [Source:UniProtKB/Swiss-Prot;Acc:O15198] [ENST00000399275]	**-1.338**
TRUB1	Homo sapiens TruB pseudouridine (psi) synthase homolog 1 (E. coli) (TRUB1), mRNA [NM_139169]	**-1.338**
FAM100B	Homo sapiens family with sequence similarity 100, member B (FAM100B), mRNA [NM_182565]	**-1.337**
LOC400013	Homo sapiens misc_RNA (LOC400013), miscRNA [XR_019347]	**-1.336**
SPIN4	Homo sapiens spindlin family, member 4 (SPIN4), mRNA [NM_001012968]	**-1.336**
KLRC1	Homo sapiens killer cell lectin-like receptor subfamily C, member 1 (KLRC1), mRNA [NM_007328]	**-1.335**
LPHN3	Homo sapiens latrophilin 3 (LPHN3), mRNA [NM_015236]	**-1.334**
IGFBP7	Homo sapiens insulin-like growth factor binding protein 7 (IGFBP7), mRNA [NM_001553]	**-1.332**

LOC645683	Homo sapiens ribosomal protein L13a pseudogene (LOC645683), non-coding RNA [NR_004844]	**-1.331**
KIAA1586	Homo sapiens KIAA1586 (KIAA1586), mRNA [NM_020931]	**-1.331**
LOC791120	Homo sapiens hypothetical LOC791120 (LOC791120), non-coding RNA [NR_015357]	**-1.330**
DNAJC5	Full-length cDNA clone CS0DN003YL17 of Adult brain of Homo sapiens (human). [CR607484]	**-1.329**
LOC137107	Ribosomal protein L1 [Source:UniProtKB/TrEMBL;Acc: A8MXH3] [ENST00000399893]	**-1.328**
LOC440900	Full-length cDNA clone CS0DI015YJ05 of Placenta Cot 25-normalized of Homo sapiens (human). [CR601322]	**-1.328**
NR4A2	Homo sapiens nuclear receptor subfamily 4, group A, member 2 (NR4A2), mRNA [NM_006186]	**-1.327**
ELP2P	Homo sapiens endozepine-like peptide 2 pseudogene (ELP2P), non-coding RNA [NR_024120]	**-1.322**
HMGB2	Homo sapiens high-mobility group box 2 (HMGB2), mRNA [NM_002129]	**-1.322**
C13orf33	Homo sapiens chromosome 13 open reading frame 33 (C13orf33), mRNA [NM_032849]	**-1.321**
LOC341378	Homo sapiens similar to Golgi-associated microtubule-binding protein (LOC341378), mRNA [XM_001715424]	**-1.319**
APBA3	Homo sapiens amyloid beta (A4) precursor protein-binding, family A, member 3 (APBA3), mRNA [NM_004886]	**-1.316**
ZNF251	Homo sapiens zinc finger protein 251 (ZNF251), mRNA [NM_138367]	**-1.316**
ZC3H7B	Homo sapiens zinc finger CCCH-type containing 7B (ZC3H7B), mRNA [NM_017590]	**-1.313**
ABHD10	Homo sapiens abhydrolase domain containing 10 (ABHD10), mRNA [NM_018394]	**-1.313**
LOC731688	Homo sapiens misc_RNA (LOC731688), miscRNA [XR_015992]	**-1.304**
ZNF254	Homo sapiens zinc finger protein 254 (ZNF254), mRNA [NM_203282]	**-1.304**
HOXC8	Homo sapiens homeobox C8 (HOXC8), mRNA [NM_022658]	**-1.303**
TNFSF10	Homo sapiens tumor necrosis factor (ligand) superfamily, member 10 (TNFSF10), mRNA [NM_003810]	**-1.303**
TGFBI	AL573456 Homo sapiens PLACENTA COT 25-NORMALIZED Homo sapiens cDNA clone CS0DI051YC22 3-PRIME, mRNA sequence [AL573456]	**-1.301**
FAM83D	Homo sapiens family with sequence similarity 83, member D (FAM83D), mRNA [NM_030919]	**-1.300**
LOC392425	Homo sapiens hypothetical LOC392425 (LOC392425), mRNA [XM_001714831]	**-1.300**
SFRS12	Homo sapiens mRNA for splicing factor, arginine/serine-rich 12 variant protein. [AB209694]	**-1.297**
LOC253482	Homo sapiens misc_RNA (LOC253482), miscRNA [XR_016415]	**-1.296**
LOC344328	Homo sapiens misc_RNA (LOC344328), miscRNA [XR_019373]	**-1.296**
LOC442249	Homo sapiens misc_RNA (LOC442249), miscRNA [XR_019231]	**-1.296**
DLX1	Homo sapiens distal-less homeobox 1 (DLX1), mRNA [NM_178120]	**-1.296**

LOC286434	Homo sapiens cDNA FLJ20463 fis, clone KAT06143. [AK000470]	**-1.295**
NACA2	Homo sapiens nascent polypeptide-associated complex alpha subunit 2 (NACA2), mRNA [NM_199290]	**-1.295**
HES1	Homo sapiens hairy and enhancer of split 1, (Drosophila) (HES1), mRNA [NM_005524]	**-1.294**
LYPD1	Homo sapiens LY6/PLAUR domain containing 1 (LYPD1), mRNA [NM_144586]	**-1.293**
TMEM181	Homo sapiens transmembrane protein 181 (TMEM181), mRNA [NM_020823]	**-1.293**
LOC646576	Full-length cDNA clone CS0DH007YA24 of T cells (Jurkat cell line) of Homo sapiens (human). [CR620567]	**-1.292**
TRPS1	Homo sapiens trichorhinophalangeal syndrome I (TRPS1), mRNA [NM_014112]	**-1.285**
CDC5L	Cell division cycle 5-like protein (Cdc5-like protein) [Source:UniProtKB /Swiss-Prot;Acc:Q99459] [ENST00000371477]	**-1.285**
FLJ14327	Homo sapiens cDNA FLJ14327 fis, clone PLACE4000250. [AK024389]	**-1.284**
SMARCA5	Homo sapiens SWI/SNF related, matrix associated, actin dependent regulator of chromatin, subfamily a, member 5 (SMARCA5), mRNA [NM_003601]	**-1.284**
HMGB3L1	Homo sapiens high-mobility group (nonhistone chromosomal) protein 4-like (HMG4L), non-coding RNA [NR_002165]	**-1.280**
ADAMTSL4	Homo sapiens cDNA FLJ13544 fis, clone PLACE1006815. [AK023606]	**-1.279**
TCTEX1D2	Homo sapiens Tctex1 domain containing 2 (TCTEX1D2), mRNA [NM_152773]	**-1.275**
KAL1	Homo sapiens Kallmann syndrome 1 sequence (KAL1), mRNA [NM_000216]	**-1.272**
UBXN7	Homo sapiens UBX domain protein 7 (UBXN7), mRNA [NM_015562]	**-1.268**
ZNF525	Homo sapiens zinc finger protein 525 (ZNF525), non-coding RNA [NR_003699]	**-1.263**
IGF1	Homo sapiens insulin-like growth factor 1 (somatomedin C) (IGF1), mRNA [NM_000618]	**-1.263**
SRP19	Homo sapiens signal recognition particle 19kDa (SRP19), mRNA [NM_003135]	**-1.263**
FNBP4	Homo sapiens formin binding protein 4 (FNBP4), mRNA [NM_015308]	**-1.262**
PXMP2	Homo sapiens peroxisomal membrane protein 2, 22kDa, mRNA (cDNA clone IMAGE:4098463), [BC009836]	**-1.260**
LYSMD3	Homo sapiens LysM, putative peptidoglycan-binding, domain containing 3 (LYSMD3), mRNA [NM_198273]	**-1.256**
CAV3	Homo sapiens caveolin 3 (CAV3), mRNA [NM_001234]	**-1.255**
C3orf51	Homo sapiens chromosome 3 open reading frame 51 (C3orf51), non-coding RNA [NR_024615]	**-1.254**
FAM86A	Homo sapiens family with sequence similarity 86, member A (FAM86A), mRNA [NM_201400]	**-1.254**
SYAP1	Homo sapiens synapse associated protein 1, SAP47 homolog (Drosophila) (SYAP1), mRNA [NM_032796]	**-1.252**
EID2	Homo sapiens EP300 interacting inhibitor of differentiation 2 (EID2), mRNA [NM_153232]	**-1.251**
ARGLU1	Homo sapiens arginine and glutamate rich 1 (ARGLU1), mRNA [NM_018011]	**-1.250**
BHLHE22	Homo sapiens basic helix-loop-helix domain containing, class B, 5 (BHLHB5), mRNA [NM_152414]	**-1.248**
CCIN	Homo sapiens calicin (CCIN), mRNA [NM_005893]	**-1.247**

LOC339843	Homo sapiens misc_RNA (LOC339843), miscRNA [XR_016598]	**-1.247**
CRYAA	Homo sapiens crystallin, alpha A (CRYAA), mRNA [NM_000394]	**-1.245**
EFNA5	Homo sapiens ephrin-A5 (EFNA5), mRNA [NM_001962]	**-1.244**
CIT	Homo sapiens citron (rho-interacting, serine/threonine kinase 21) (CIT), mRNA [NM_007174]	**-1.242**
hCG_21078	Homo sapiens hCG21078 (LOC389435), mRNA [NM_001089587]	**-1.242**
RASSF5	Homo sapiens Ras association (RalGDS/AF-6) domain family member 5 (RASSF5), mRNA [NM_182663]	**-1.238**
ZNF714	Homo sapiens zinc finger protein 714 (ZNF714), mRNA [NM_182515]	**-1.238**
SEPT13	Homo sapiens septin 13 (SEPT13), non-coding RNA [NR_024271]	**-1.234**
MED7	Homo sapiens mediator complex subunit 7 (MED7), mRNA [NM_004270]	**-1.233**
LOC391160	Homo sapiens misc_RNA (LOC391160), miscRNA [XR_018489]	**-1.232**
LOC284288	Homo sapiens similar to Chromosome 15 open reading frame 15 (LOC284288), mRNA [XM_001714653]	**-1.231**
LOC402176	Homo sapiens similar to 60S ribosomal protein L21 (LOC402176), mRNA [NM_001011538]	**-1.230**
LOC100129566	Homo sapiens hypothetical protein LOC100129566 (LOC100129566), mRNA [XM_001718519]	**-1.229**
LOC392522	Homo sapiens misc_RNA (LOC392522), miscRNA [XR_018292]	**-1.228**
ANKRD37	Homo sapiens ankyrin repeat domain 37 (ANKRD37), mRNA [NM_181726]	**-1.227**
IGFL2	Homo sapiens IGF-like family member 2 (IGFL2), mRNA [NM_001002915]	**-1.227**
ZNF638	Homo sapiens zinc finger protein 638 (ZNF638),mRNA [NM_014497]	**-1.227**
RBM12B	Homo sapiens RNA binding motif protein 12B (RBM12B), mRNA [NM_203390]	**-1.224**
LMNB1	Homo sapiens lamin B1 (LMNB1), mRNA [NM_005573]	**-1.223**
BPNT1	Homo sapiens 3'(2'), 5'-bisphosphate nucleotidase 1 (BPNT1), mRNA [NM_006085]	**-1.220**
KRT18P49	Homo sapiens keratin 18 pseudogene 49 (KRT18P49), mRNA [XM_001714362]	**-1.219**
ZNF284	Zinc finger protein 284 [Source:UniProtKB/Swiss-Prot;Acc:Q2VY69] [ENST00000328297]	**-1.219**
RHOH	Homo sapiens ras homolog gene family, member H (RHOH), mRNA [NM_004310]	**-1.218**
DKFZp547G183	Homo sapiens mRNA; cDNA DKFZp547G183 (from clone DKFZp547G183). [AL359570]	**-1.216**
GNRH1	Homo sapiens gonadotropin-releasing hormone 1 (luteinizing-releasing hormone) (GNRH1), mRNA [NM_000825]	**-1.216**
LOC645884	Homo sapiens cDNA FLJ25739 fis, clone TST05834. [AK098605]	**-1.208**
LOC401076	Homo sapiens misc_RNA (LOC401076), miscRNA [XR_018154]	**-1.205**
PRDM15	Homo sapiens PR domain containing 15 (PRDM15), mRNA [NM_022115]	**-1.205**
FBXO10	Homo sapiens F-box protein 10 (FBXO10), mRNA [NM_012166]	**-1.204**
ADAMTS3	Homo sapiens ADAM metallopeptidase with thrombospondin type 1 motif, 3 (ADAMTS3), mRNA [NM_014243]	**-1.201**
C1orf103	Homo sapiens chromosome 1 open reading frame 103 (C1orf103), mRNA [NM_018372]	**-1.199**

ZNF223	Homo sapiens zinc finger protein 223 (ZNF223), mRNA [NM_013361]	**-1.195**
ZIC2	Homo sapiens Zic family member 2 (odd-paired homolog, Drosophila) (ZIC2), mRNA [NM_007129]	**-1.193**
C17orf89	Homo sapiens chromosome 17 open reading frame 89 (C17orf89), mRNA [NM_001086521]	**-1.192**
LOC338620	Homo sapiens hypothetical protein LOC338620, mRNA (cDNA clone IMAGE:6023208), [BC043009]	**-1.189**
SLFN13	Homo sapiens schlafen family member 13 (SLFN13), mRNA [NM_144682]	**-1.189**
LOC402360	Homo sapiens similar to hCG1742476 (LOC402360), mRNA [XM_001722407]	**-1.188**
RPL24	Homo sapiens ribosomal protein L24 (RPL24), mRNA [NM_000986]	**-1.188**
PIK3R3	Homo sapiens phosphoinositide-3-kinase, regulatory subunit 3 (gamma) (PIK3R3), mRNA [NM_003629]	**-1.187**
SET	Homo sapiens SET nuclear oncogene (SET), mRNA [NM_003011]	**-1.187**
MGC70870	Homo sapiens C-terminal binding protein 2 pseudogene (MGC70870), non-coding RNA [NR_003682]	**-1.185**
RIBC2	Homo sapiens RIB43A domain with coiled-coils 2 (RIBC2), mRNA [NM_015653]	**-1.183**
ITGA2	Homo sapiens integrin, alpha 2 (CD49B, alpha 2 subunit of VLA-2 receptor) (ITGA2), mRNA [NM_002203]	**-1.182**
NMU	Homo sapiens neuromedin U (NMU), mRNA [NM_006681]	**-1.180**
DLX4	Homo sapiens distal-less homeobox 4 (DLX4),mRNA [NM_138281]	**-1.179**
ZNF789	Homo sapiens cDNA FLJ16554 fis, clone SPLEN2016135. [AK131429]	**-1.178**
ZNF493	Homo sapiens zinc finger protein 493 (ZNF493), mRNA [NM_001076678]	**-1.178**
DEPDC7	Homo sapiens DEP domain containing 7 (DEPDC7), mRNA [NM_139160]	**-1.177**
NOS1	Homo sapiens nitric oxide synthase 1 (neuronal) (NOS1), mRNA [NM_000620]	**-1.176**
LOC147804	Homo sapiens tropomyosin 3 pseudogene (LOC147804), non-coding RNA [NR_003148]	**-1.175**
RPS6KB1	Homo sapiens ribosomal protein S6 kinase, 70kDa, polypeptide 1 (RPS6KB1), mRNA [NM_003161]	**-1.174**
FLJ13197	Homo sapiens cDNA FLJ13197 fis, clone NT2RP3004451. [AK023259]	**-1.174**
SFRS18	Homo sapiens splicing factor, arginine/serine-rich 18 (SFRS18), mRNA [NM_032870]	**-1.174**
FLJ31813	Homo sapiens cDNA FLJ31813 fis, clone NT2RI2009517. [AK056375]	**-1.173**
LOC729608	Homo sapiens similar to Brix domain containing 1 (LOC729608), mRNA [XM_001130778]	**-1.173**
CGA	Homo sapiens glycoprotein hormones, alpha polypeptide (CGA), mRNA [NM_000735]	**-1.173**
C17orf86	Homo sapiens clone FLB3442 PRO0872 mRNA. [AF130050]	**-1.171**
LOC344332	Homo sapiens similar to hCG1812048 (LOC344332), mRNA [XM_001125944]	**-1.171**
CAPS	Homo sapiens calcyphosine (CAPS), mRNA [NM_004058]	**-1.168**
POLI	Homo sapiens polymerase (DNA directed) iota (POLI), mRNA [NM_007195]	**-1.167**
MARS2	Homo sapiens methionyl-tRNA synthetase 2, mitochondrial (MARS2),mRNA [NM_138395]	**-1.165**

LOC727835	Homo sapiens misc_RNA (LOC727835), miscRNA [XR_015767]	**-1.164**
LOC120364	Homo sapiens similar to heterogeneous nuclear ribonucleoprotein A1 (LOC120364), mRNA [XM_001718684]	**-1.163**
POLR2H	Homo sapiens polymerase (RNA) II (DNA directed) polypeptide H (POLR2H), mRNA [NM_006232]	**-1.160**
SAP30	Homo sapiens Sin3A-associated protein, 30kDa (SAP30), mRNA [NM_003864]	**-1.159**
MGC12935	Homo sapiens hypothetical protein MGC12935, mRNA (cDNA clone IMAGE:4309284), [BC004565]	**-1.158**
SNRPC	Homo sapiens small nuclear ribonucleoprotein polypeptide C (SNRPC), mRNA [NM_003093]	**-1.158**
GNGT1	Homo sapiens guanine nucleotide binding protein (G protein), gamma transducing activity polypeptide 1 (GNGT1), mRNA [NM_021955]	**-1.156**
HNRNPR	Homo sapiens heterogeneous nuclear ribonucleoprotein R (HNRNPR), mRNA [NM_005826]	**-1.156**
AP4S1	Homo sapiens adaptor-related protein complex 4, sigma 1 subunit (AP4S1), mRNA [NM_001128126]	**-1.152**
KLF10	Homo sapiens Kruppel-like factor 10 (KLF10), mRNA [NM_005655]	**-1.150**
HEXIM1	Homo sapiens hexamethylene bis-acetamide inducible 1 (HEXIM1), mRNA [NM_006460]	**-1.150**
NCRNA00081	Homo sapiens non-protein coding RNA 81 (NCRNA00081), non-coding RNA [NR_024140]	**-1.149**
FLJ45244	Homo sapiens hypothetical locus FLJ45244 (FLJ45244), non-coding RNA [NR_015415]	**-1.148**
GCNT1	Homo sapiens glucosaminyl (N-acetyl) transferase 1, core 2 (beta-1,6-N-acetylglucosaminyltransferase) (GCNT1), mRNA [NM_001490]	**-1.148**
IKZF4	Homo sapiens IKAROS family zinc finger 4 (Eos) (IKZF4), mRNA [NM_022465]	**-1.148**
COL3A1	Homo sapiens collagen, type III, alpha 1 (COL3A1), mRNA [NM_000090]	**-1.148**
RP9P	Homo sapiens retinitis pigmentosa 9 pseudogene (RP9P), non-coding RNA [NR_003500]	**-1.147**
TFDP3	Homo sapiens transcription factor Dp family, member 3 (TFDP3), mRNA [NM_016521]	**-1.147**
TRIM23	Homo sapiens tripartite motif-containing 23 (TRIM23), mRNA [NM_001656]	**-1.147**
LOC100127983	Homo sapiens hypothetical protein LOC100127983 (LOC100127983), mRNA [XM_001722571]	**-1.146**
ATP5E	Homo sapiens ATP synthase, H+ transporting, mitochondrial F1 complex, epsilon subunit (ATP5E), mRNA [NM_006886]	**-1.145**
DGUOK	Homo sapiens deoxyguanosine kinase (DGUOK), nuclear gene encoding mitochondrial protein, mRNA [NM_080916]	**-1.143**
LOC646346	Homo sapiens misc_RNA (LOC646346), miscRNA [XR_019544]	**-1.142**
TUBA8	Homo sapiens tubulin, alpha 8 (TUBA8), mRNA [NM_018943]	**-1.142**
CCDC66	Homo sapiens cDNA FLJ38369 fis, clone FEBRA2001828. [AK095688]	**-1.141**
NASP	Homo sapiens nuclear autoantigenic sperm protein (histone-binding) (NASP),	**-1.141**

	mRNA [NM_172164]	
LOC730235	Homo sapiens hypothetical LOC730235 (LOC730235), mRNA [XM_001732867]	**-1.140**
TSPAN12	Homo sapiens tetraspanin 12 (TSPAN12), mRNA [NM_012338]	**-1.139**
CDKN3	Homo sapiens cyclin-dependent kinase inhibitor 3 (CDKN3), mRNA [NM_005192]	**-1.138**
EXOSC2	Homo sapiens exosome component 2 (EXOSC2), mRNA [NM_014285]	**-1.136**
LOC100130288	Homo sapiens cDNA clone IMAGE:5295205, with apparent retained intron. [BC043212]	**-1.135**
BRPF3	Homo sapiens bromodomain and PHD finger containing, 3 (BRPF3), mRNA [NM_015695]	**-1.133**
CKAP4	Homo sapiens cytoskeleton-associated protein 4 (CKAP4), mRNA [NM_006825]	**-1.132**
GABRE	Homo sapiens gamma-aminobutyric acid (GABA) A receptor, epsilon (GABRE), mRNA [NM_004961]	**-1.132**
LOC100131575	Homo sapiens misc_RNA (LOC100131575), miscRNA [XR_038726]	**-1.132**
ZFP36L2	Homo sapiens zinc finger protein 36, C3H type-like 2 (ZFP36L2), mRNA [NM_006887]	**-1.131**
CPSF2	Homo sapiens cleavage and polyadenylation specific factor 2, 100kDa (CPSF2), mRNA [NM_017437]	**-1.131**
POFUT1	Homo sapiens protein O-fucosyltransferase 1 (POFUT1), mRNA [NM_172236]	**-1.131**
CCDC58	Homo sapiens coiled-coil domain containing 58 (CCDC58), mRNA [NM_001017928]	**-1.130**
DKFZp667E0512	Homo sapiens mRNA; cDNA DKFZp667E0512 (from clone DKFZp667E0512). [AL713660]	**-1.129**
C1orf104	Homo sapiens cDNA FLJ43522 fis, clone PLACE5000260. [AK125510]	**-1.128**
PSD3	Homo sapiens pleckstrin and Sec7 domain containing 3 (PSD3), mRNA [NM_015310]	**-1.126**
AGAP7	Homo sapiens ArfGAP with GTPase domain, ankyrin repeat and PH domain 7 (AGAP7), mRNA [NM_001077685]	**-1.126**
QKI	Homo sapiens quaking homolog, KH domain RNA binding (mouse) (QKI), mRNA [NM_006775]	**-1.125**
RNF214	Homo sapiens ring finger protein 214 (RNF214), transcript variant 2, mRNA [NM_001077239]	**-1.125**
BMPER	BMP-binding endothelial regulator protein Precursor (Protein crossveinless-2)(hCV2) [Source:UniProtKB/Swiss-Prot;Acc: Q8N8U9] [ENST00000297161]	**-1.124**
PMEPA1	Homo sapiens prostate transmembrane protein, androgen induced 1 (PMEPA1), mRNA [NM_020182]	**-1.123**
MEX3A	Homo sapiens mex-3 homolog A (C. elegans) (MEX3A), mRNA [NM_001093725]	**-1.122**
GOLGA6	Homo sapiens golgi autoantigen, golgin subfamily a, 6 (GOLGA6), mRNA [NM_001038640]	**-1.121**
ANP32A	Homo sapiens acidic (leucine-rich) nuclear phosphoprotein 32 family, member A (ANP32A), mRNA [NM_006305]	**-1.120**

OSCAR	Homo sapiens osteoclast associated, immunoglobulin-like receptor (OSCAR), mRNA [NM_206818]	**-1.120**
TRDN	Homo sapiens mRNA for triadin (TRDN gene), Trisk 51 isoform. [AJ489257]	**-1.120**
C3orf34	Homo sapiens chromosome 3 open reading frame 34 (C3orf34), mRNA [NM_032898]	**-1.119**
KLHL15	Homo sapiens kelch-like 15 (Drosophila) (KLHL15), mRNA [NM_030624]	**-1.119**
C10orf18	Homo sapiens chromosome 10 open reading frame 18 (C10orf18), mRNA [NM_017782]	**-1.118**
PTHLH	Parathyroid hormone-related protein Precursor (PTH-rP)(PTHrP) [Contains PTHrP[1-36];PTHrP[38-94]; Osteostatin(PTHrP[107-139])] [Source:UniProtKB/Swiss-Prot;Acc:P12272] [ENST00000354417]	**-1.118**
DIRAS3	Homo sapiens DIRAS family, GTP-binding RAS-like 3 (DIRAS3), mRNA [NM_004675]	**-1.117**
LOC401602	Homo sapiens misc_RNA (LOC401602), miscRNA [XR_018322]	**-1.117**
RPL37	Homo sapiens ribosomal protein L37 (RPL37), mRNA [NM_000997]	**-1.116**
WDR75	Homo sapiens WD repeat domain 75 (WDR75), mRNA [NM_032168]	**-1.112**
ZNF813	Homo sapiens zinc finger protein 813 (ZNF813), mRNA [NM_001004301]	**-1.112**
PUS7L	Homo sapiens pseudouridylate synthase 7 homolog (S. cerevisiae)-like (PUS7L), mRNA [NM_031292]	**-1.111**
FLJ11292	Homo sapiens hypothetical protein FLJ11292, mRNA (cDNA clone MGC:151043 IMAGE:40125985),. [BC117434]	**-1.111**
MKX	Homo sapiens mohawk homeobox (MKX), mRNA [NM_173576]	**-1.110**
SMCHD1	Homo sapiens structural maintenance of chromosomes flexible hinge domain containing 1 (SMCHD1), mRNA [NM_015295]	**-1.109**
CCDC61	Homo sapiens coiled-coil domain containing 61 (CCDC61), mRNA [NM_001080402]	**-1.109**
PAX4	Homo sapiens paired box 4 (PAX4), mRNA [NM_006193]	**-1.109**
LOC100133869	Homo sapiens misc_RNA (LOC100133869), miscRNA [XR_037019]	**-1.108**
GNG11	Homo sapiens guanine nucleotide binding protein (G protein), gamma 11 (GNG11), mRNA [NM_004126]	**-1.107**
LOC729279	Homo sapiens misc_RNA (LOC729279), miscRNA [XR_015921]	**-1.107**
SHC3	Homo sapiens SHC (Src homology 2 domain containing) transforming protein 3 (SHC3), mRNA [NM_016848]	**-1.106**
FRMD8	Homo sapiens FKSG44 (FKSG44) mRNA, complete cds. [AF334946]	**-1.106**
GALNT4	Homo sapiens UDP-N-acetyl-alpha-D-galactosamine:polypep tide N-acetylgalactosaminyltransferase 4 (GalNAc-T4) (GALNT4), mRNA [NM_003774]	**-1.103**
HOTAIR	Homo sapiens hox transcript antisense RNA (non-protein coding) (HOTAIR), non-coding RNA [NR_003716]	**-1.103**
FAM58A	Homo sapiens family with sequence similarity 58, member A (FAM58A), mRNA [NM_152274]	**-1.100**
GARS	Homo sapiens glycyl-tRNA synthetase (GARS), mRNA [NM_002047]	**-1.100**

MYCBP	Homo sapiens c-myc binding protein (MYCBP), mRNA [NM_012333]	**-1.100**
PRPF4B	Homo sapiens PRP4 pre-mRNA processing factor 4 homolog B (yeast) (PRPF4B), mRNA [NM_003913]	**-1.099**
COL1A1	H.sapiens mRNA for prepro-alpha1(I) collagen. [Z74615]	**-1.098**
C5orf13	Homo sapiens chromosome 5 open reading frame 13 (C5orf13), mRNA [NM_004772]	**-1.098**
LOC644950	Homo sapiens similar to histone (LOC644950), mRNA [XM_001726996]	**-1.097**
CHMP4C	Homo sapiens chromatin modifying protein 4C (CHMP4C), mRNA [NM_152284]	**-1.096**
LOC390595	Homo sapiens mRNA; cDNA DKFZp686J168 (from clone DKFZp686J168). [CR749500]	**-1.095**
ZNF738	Homo sapiens zinc finger protein 738, mRNA (cDNA clone IMAGE:4838161), [BC034499]	**-1.095**
LOC283701	Homo sapiens mRNA for FLJ00278 protein. [AK090401]	**-1.093**
SOX7	Homo sapiens SRY (sex determining region Y)-box 7 (SOX7), mRNA [NM_031439]	**-1.093**
LOC388621	Homo sapiens hypothetical LOC388621 (LOC388621), mRNA [XM_001725488]	**-1.092**
SEPW1	Homo sapiens selenoprotein W, 1 (SEPW1), mRNA [NM_003009]	**-1.092**
SERF1A	Homo sapiens small EDRK-rich factor 1A (telomeric) (SERF1A), mRNA [NM_022968]	**-1.091**
SULF2	Homo sapiens sulfatase 2 (SULF2), mRNA [NM_018837]	**-1.091**
ZNF431	Homo sapiens zinc finger protein 431 (ZNF431), mRNA [NM_133473]	**-1.089**
LOC100133224	Homo sapiens similar tohCG2041320(LOC100133224),mRNA [XM_001716151]	**-1.087**
LOC51152	Homo sapiens melanoma antigen mRNA, [AF172850]	**-1.085**
HSPA9	Homo sapiens heat shock 70kDa protein 9 (mortalin) (HSPA9), nuclear gene encoding mitochondrial protein, mRNA [NM_004134]	**-1.084**
LOC100129720	full-length cDNA clone CS0DI044YN21 of Placenta Cot 25-normalized of Homo sapiens (human). [CR619772]	**-1.084**
LOC339483	Homo sapiens cDNA FLJ38790 fis, clone LIVER2002842. [AK096109]	**-1.083**
DIS3	Homo sapiens DIS3 mitotic control homolog (S. cerevisiae) (DIS3), mRNA [NM_014953]	**-1.082**
C15orf5	Homo sapiens chromosome 15 open reading frame 5, mRNA (cDNA clone MGC:97283 IMAGE:7262532), [BC069765]	**-1.081**
LOC284230	Homo sapiens similar to mCG7611 (LOC284230), mRNA [XM_208185]	**-1.081**
PSTK	Homo sapiens phosphoseryl-tRNA kinase (PSTK), mRNA [NM_153336]	**-1.081**
UBE2V2	Homo sapiens ubiquitin-conjugating enzyme E2 variant 2 (UBE2V2), mRNA [NM_003350]	**-1.081**
IRAK1BP1	Homo sapiens interleukin-1 receptor-associated kinase 1 binding protein 1 (IRAK1BP1), mRNA [NM_001010844]	**-1.080**
LOC100133528	Homo sapiens similar to HIG1 domain family, member 1A (LOC100133528), mRNA [XM_001721514]	**-1.080**
C15orf40	Homo sapiens chromosome 15 open reading frame 40 (C15orf40), mRNA [NM_144597]	**-1.079**

LOC285216	Homo sapiens cDNA FLJ34909 fis, clone NT2RI2009301, moderately similar to biofunctional methylenetetrahydroflate dehydrogenase/cyclohydrolase, mitochondrial precursor. [AK092228]	**-1.079**
CG012	Novel human gene mapping to chomosome 13. [AL049782]	**-1.078**
MAP9	Homo sapiens microtubule-associated protein 9(MAP9),mRNA [NM_001039580]	**-1.078**
NNT	NAD(P) transhydrogenase, mitochondrial Precursor (EC 1.6.1.2)[Source:UniProtKB/Swiss-Prot;Acc:Q13423] [ENST00000264663]	**-1.078**
SASS6	Homo sapiens spindle assembly 6 homolog (C. elegans) (SASS6), mRNA [NM_194292]	**-1.078**
SGK493	Homo sapiens protein kinase-like protein SgK493 (SGK493), mRNA [NM_138370]	**-1.078**
EPHA3	Homo sapiens EPH receptor A3 (EPHA3), mRNA [NM_005233]	**-1.077**
DCUN1D2	Homo sapiens DCN1, defective in cullin neddylation 1, domain containing 2 (S. cerevisiae) (DCUN1D2), mRNA [NM_001014283]	**-1.077**
LOC391655	Homo sapiens misc_RNA (LOC391655), miscRNA [XR_018405]	**-1.076**
FGF12	Homo sapiens fibroblast growth factor 12 (FGF12), mRNA [NM_004113]	**-1.076**
IRS1	Homo sapiens insulin receptor substrate 1 (IRS1), mRNA [NM_005544]	**-1.075**
ACTR2	Homo sapiens ARP2 actin-related protein 2 homolog (yeast) (ACTR2), mRNA [NM_001005386]	**-1.074**
CUL2	Homo sapiens cullin 2 (CUL2), mRNA [NM_003591]	**-1.074**
TYMS	Homo sapiens thymidylate synthetase (TYMS), mRNA [NM_001071]	**-1.074**
KRT18P28	Homo sapiens similar to Keratin, type I cytoskeletal 18 (Cytokeratin-18) (CK-18) (Keratin-18) (K18) (LOC343326), mRNA [XR_019568]	**-1.073**
C12orf48	Homo sapiens PNAS-15 mRNA, [AF274940]	**-1.072**
FLJ10357	Homo sapiens hypothetical protein FLJ10357 (FLJ10357), mRNA [NM_018071]	**-1.070**
MATN3	Homo sapiens matrilin 3 (MATN3), mRNA [NM_002381]	**-1.069**
FAM133B	Homo sapiens family with sequence similarity 133, member B (FAM133B), mRNA [NM_001040057]	**-1.068**
RPL23AP13	Homo sapiens ribosomal protein L23a pseudogene 13 (RPL23AP13), non-coding RNA [NR_002229]	**-1.067**
TMEM56	Homo sapiens transmembrane protein 56 (TMEM56), mRNA [NM_152487]	**-1.067**
LOC220429	Homo sapiens CTAGE family, member 5 pseudogene, mRNA (cDNA clone IMAGE:5270026). [BC030655]	**-1.065**
CCDC144A	Homo sapiens mRNA for KIAA0565 protein, partial cds. [AB011137]	**-1.064**
EFR3B	Homo sapiens EFR3 homolog B (S. cerevisiae) (EFR3B), mRNA [NM_014971]	**-1.063**
TFAM	Homo sapiens transcription factor A, mitochondrial (TFAM), nuclear gene encoding mitochondrial protein, mRNA [NM_003201]	**-1.063**
FAM64A	Homo sapiens family with sequence similarity 64, member A (FAM64A), mRNA [NM_019013]	**-1.062**
MYB	Homo sapiens v-myb myeloblastosis viral oncogene homolog (avian) (MYB), mRNA [NM_005375]	**-1.062**
BCCIP	Homo sapiens cDNA FLJ54246, highly similar to Homo sapiens BRCA2 and CDKN1A interacting protein (BCCIP), mRNA. [AK304529]	**-1.060**

PFDN4	Homo sapiens prefoldin subunit 4 (PFDN4), mRNA [NM_002623]	**-1.060**
DDX6	Homo sapiens DEAD (Asp-Glu-Ala-Asp) box polypeptide 6 (DDX6), mRNA [NM_004397]	**-1.060**
ANKRD36B	Homo sapiens CLL-associated antigen KW-1 splice variant 2 mRNA; alternatively spliced. [AF432209]	**-1.060**
BIC	Homo sapiens BIC transcript (BIC), non-coding RNA [NR_001458]	**-1.059**
HSPA4L	Homo sapiens heat shock 70kDa protein 4-like (HSPA4L), mRNA [NM_014278]	**-1.058**
LOC646093	Homo sapiens misc_RNA (LOC646093), miscRNA [XR_017054]	**-1.055**
ZNF681	Homo sapiens zinc finger protein 681 (ZNF681), mRNA [NM_138286]	**-1.055**
LOC728179	Homo sapiens misc_RNA (LOC728179), miscRNA [XR_015445]	**-1.054**
RINT1	Homo sapiens RAD50 interactor 1 (RINT1), mRNA [NM_021930]	**-1.054**
ITGAV	Homo sapiens integrin, alpha V (vitronectin receptor, alpha polypeptide, antigen CD51) (ITGAV), mRNA [NM_002210]	**-1.053**
DUT	Homo sapiens deoxyuridine triphosphatase (DUT), nuclear gene encoding mitochondrial protein, mRNA [NM_001025248]	**-1.052**
LOC729779	Homo sapiens misc_RNA (LOC729779), miscRNA [XR_016024]	**-1.052**
TBX15	Homo sapiens T-box 15 (TBX15), mRNA [NM_152380]	**-1.052**
CCDC68	Homo sapiens coiled-coil domain containing 68 (CCDC68),mRNA [NM_025214]	**-1.051**
ZBED5	Homo sapiens zinc finger, BED-type containing 5 (ZBED5),mRNA [NM_021211]	**-1.051**
CETN3	Homo sapiens centrin, EF-hand protein, 3 (CDC31 homolog, yeast) (CETN3), mRNA [NM_004365]	**-1.050**
ZBED2	Homo sapiens zinc finger, BED-type containing 2 (ZBED2),mRNA [NM_024508]	**-1.050**
GJA1	Homo sapiens gap junction protein, alpha 1 (GJA1), mRNA [NM_000165]	**-1.050**
LIG4	Homo sapiens ligase IV, DNA, ATP-dependent (LIG4), mRNA [NM_002312]	**-1.048**
LOC100133154	Homo sapiens hypothetical protein LOC100133154 (LOC100133154), mRNA [XM_001714925]	**-1.048**
FGFR1OP	Homo sapiens FGFR1 oncogene partner (FGFR1OP), mRNA [NM_007045]	**-1.047**
KLHL23	Homo sapiens kelch-like 23 (Drosophila) (KLHL23), mRNA [NM_144711]	**-1.046**
MGC26597	Homo sapiens PIP5K1A pseudogene, mRNA (cDNA clone IMAGE:4828163). [BC028580]	**-1.046**
ZC3H13	Homo sapiens zinc finger CCCH-type containing 13 (ZC3H13), mRNA [NM_015070]	**-1.043**
SAMHD1	Homo sapiens SAM domain and HD domain 1 (SAMHD1), mRNA [NM_015474]	**-1.041**
FUT6	Homo sapiens fucosyltransferase 6 (alpha (1,3) fucosyltransferase) (FUT6), mRNA [NM_000150]	**-1.040**
TERF1	Homo sapiens telomeric repeat binding factor (NIMA-interacting) 1 (TERF1), mRNA [NM_017489]	**-1.040**
CCNJ	Homo sapiens cyclin J (CCNJ), mRNA [NM_019084]	**-1.039**
NCRNA00115	Homo sapiens non-protein coding RNA 115 (NCRNA00115), non-coding RNA [NR_024321]	**-1.038**
SIGLEC9	Homo sapiens sialic acid binding Ig-like lectin 9 (SIGLEC9), mRNA [NM_014441]	**-1.035**

RPL26	Homo sapiens ribosomal protein L26 (RPL26), mRNA [NM_000987]	**-1.035**
SALL2	Homo sapiens sal-like 2 (Drosophila) (SALL2), mRNA [NM_005407]	**-1.034**
TXNDC17	Homo sapiens thioredoxin domain containing 17 (TXNDC17), mRNA [NM_032731]	**-1.034**
WNT11	Homo sapiens wingless-type MMTV integration site family, member 11 (WNT11), mRNA [NM_004626]	**-1.034**
RPL27A	Homo sapiens ribosomal protein L27a (RPL27A), mRNA [NM_000990]	**-1.033**
AUTS2	Homo sapiens autism susceptibility candidate 2 (AUTS2), mRNA [NM_001127232]	**-1.032**
FLJ31306	Full-length cDNA clone CS0DF021YK18 of Fetal brain of Homo sapiens (human). [CR606347]	**-1.032**
LOC648795	Homo sapiens hypothetical LOC648795 (LOC648795), mRNA [XM_001717954]	**-1.032**
CENPA	Homo sapiens centromere protein A (CENPA), mRNA [NM_001809]	**-1.031**
SEC16B	Homo sapiens SEC16 homolog B (S. cerevisiae), mRNA (cDNA clone MGC:17455 IMAGE:3448742), [BC009106]	**-1.031**
MSL3	Homo sapiens male-specific lethal 3 homolog (Drosophila) (MSL3), mRNA [NM_078629]	**-1.030**
PEX3	Homo sapiens peroxisomal biogenesis factor 3 (PEX3), mRNA [NM_003630]	**-1.029**
RNF215	Homo sapiens ring finger protein 215 (RNF215), mRNA [NM_001017981]	**-1.029**
COX11	Homo sapiens COX11 homolog, cytochrome c oxidase assembly protein (yeast) (COX11), nuclear gene encoding mitochondrial protein, mRNA [NM_004375]	**-1.029**
ASPM	Homo sapiens asp (abnormal spindle) homolog, microcephaly associated (Drosophila) (ASPM), mRNA [NM_018136]	**-1.027**
DPY19L4	Homo sapiens dpy-19-like 4 (C. elegans) (DPY19L4), mRNA [NM_181787]	**-1.027**
RBAK	Homo sapiens RB-associated KRAB zinc finger (RBAK), mRNA [NM_021163]	**-1.027**
RPL5	Homo sapiens ribosomal protein L5 (RPL5), mRNA [NM_000969]	**-1.027**
UPF1	Homo sapiens UPF1 regulator of nonsense transcripts homolog (yeast) (UPF1), mRNA [NM_002911]	**-1.027**
IFT74	Homo sapiens intraflagellar transport 74 homolog (Chlamydomonas) (IFT74), mRNA [NM_025103]	**-1.026**
LOC441383	Homo sapiens hypothetical gene supported by AF086559; BC065734, mRNA (cDNA clone IMAGE:30352956). [BC065734]	**-1.026**
CKAP2	Homo sapiens cytoskeleton associated protein 2 (CKAP2), mRNA [NM_018204]	**-1.024**
CYP1A2	Homo sapiens cytochrome P450, family 1, subfamily A, polypeptide 2 (CYP1A2), mRNA [NM_000761]	**-1.024**
HEY2	Homo sapiens hairy/enhancer-of-split related with YRPW motif 2 (HEY2), mRNA [NM_012259]	**-1.023**
CCT6P1	Homo sapiens cDNA FLJ34861 fis, clone NT2NE2012847. [AK092180]	**-1.023**
ITGAE	Homo sapiens integrin, alpha E (antigen CD103, human mucosal lymphocyte antigen 1; alpha polypeptide) (ITGAE), mRNA [NM_002208]	**-1.021**
UCP3	Homo sapiens uncoupling protein 3 (mitochondrial, proton carrier) (UCP3), nuclear gene encoding mitochondrial protein, mRNA [NM_022803]	**-1.020**
RSRC2	Homo sapiens arginine/serine-rich coiled-coil 2 (RSRC2), mRNA [NM_198261]	**-1.019**

ZNF155	Homo sapiens zinc finger protein 155 (ZNF155), mRNA [NM_003445]	**-1.019**
LOC100131310	Homo sapiens hypothetical protein (LOC100131310), mRNA [XM_001715169]	**-1.017**
SMC5	Homo sapiens structural maintenance of chromosomes 5 (SMC5), mRNA [NM_015110]	**-1.016**
RACGAP1	Homo sapiens Rac GTPase activating protein 1 (RACGAP1), mRNA [NM_013277]	**-1.014**
TNFRSF19	Homo sapiens tumor necrosis factor receptor superfamily, member 19 (TNFRSF19), mRNA [NM_018647]	**-1.014**
ANP32D	Homo sapiens acidic (leucine-rich) nuclear phosphoprotein 32 family, member D (ANP32D), mRNA [NM_012404]	**-1.013**
RAVER2	Homo sapiens cDNA FLJ10770 fis, clone NT2RP4000159. [AK001632]	**-1.013**
SPAG1	Homo sapiens sperm associated antigen 1 (SPAG1), mRNA [NM_003114]	**-1.013**
RPL21	Homo sapiens ribosomal protein L21 (RPL21), mRNA [NM_000982]	**-1.012**
FAM53C	Homo sapiens family with sequence similarity 53, member C (FAM53C), mRNA [NM_016605]	**-1.012**
DKFZp434L192	Homo sapiens mRNA; cDNA DKFZp434L192 (from clone DKFZp434L192). [AL713776]	**-1.011**
PCMTD1	Homo sapiens protein-L-isoaspartate (D-aspartate) O-methyltransferase domain containing 1 (PCMTD1), mRNA [NM_052937]	**-1.011**
RPL29P2	Homo sapiens ribosomal protein L29 pseudogene 2 (RPL29P2), non-coding RNA [NR_002778]	**-1.011**
HS6ST1	Homo sapiens heparan sulfate 6-O-sulfotransferase 1 (HS6ST1), mRNA [NM_004807]	**-1.010**
RPL31	Homo sapiens ribosomal protein L31 (RPL31), mRNA [NM_001099693]	**-1.009**
CALML4	Homo sapiens calmodulin-like 4 (CALML4), mRNA [NM_033429]	**-1.008**
CISH	Homo sapiens cytokine inducible SH2-containing protein (CISH), mRNA [NM_145071]	**-1.008**
LGR5	Homo sapiens leucine-rich repeat-containing G protein-coupled receptor 5 (LGR5), mRNA [NM_003667]	**-1.008**
TMSB15A	Homo sapiens thymosin-like 8 (TMSL8), mRNA [NM_021992]	**-1.006**
C1orf63	Homo sapiens chromosome 1 open reading frame 63, mRNA (cDNA clone MGC:74698 IMAGE:6147639), [BC065040]	**-1.005**
INSL4	Homo sapiens insulin-like 4 (placenta) (INSL4), mRNA [NM_002195]	**-1.003**
LOC401588	Homo sapiens hypothetical LOC401588 (LOC401588), non-coding RNA [NR_015378]	**-1.003**
ZCCHC11	Homo sapiens zinc finger, CCHC domain containing 11 (ZCCHC11), mRNA [NM_001009881]	**-1.003**
CENPF	Homo sapiens centromere protein F, 350/400ka (mitosin) (CENPF), mRNA [NM_016343]	**-1.001**
