# Supplementary material for: Genotoxicity and molecular response of silver nanoparticle (NP)-based hydrogel
Source: J Nanobiotechnology. 2012 May 1;10:16. doi: 10.1186/1477-3155-10-16 (PMC3430588; doi:10.1186/1477-3155-10-16)
Supplement: Additional file 5 — Up-regulated genes in cells exposed to Hydrogel for 24 h. Fold-change is logarithmic ratio (log2 ratio) to expression level in control. [file 1477-3155-10-16-S5.pdf]

Additional File 5. Up-regulated genes in cells exposed to Hydrogel for 24 h. Fold-change is logarithmic ratio (log_2 ratio) to expression level in control.

GeneName	Description	Fold-change (log2 ratio)
SLC22A8	Homo sapiens solute carrier family 22, mRNA [NM_004254]	**8.693**
CORO1A	Homo sapiens coronin, actin binding protein, 1A, mRNA [NM_007074]	**6.848**
FLJ40606	Homo sapiens cDNA FLJ40606 fis, clone THYMU2011939. [AK097925]	**6.644**
IGFALS	Homo sapiens insulin-like growth factor binding protein, mRNA [NM_004970]	**6.644**
TMPRSS4	Homo sapiens transmembrane protease, mRNA [NM_019894]	**6.644**
MYO15A	Homo sapiens myosin XVA (MYO15A), mRNA [NM_016239]	**6.075**
PLLP	Homo sapiens plasma membrane proteolipid, mRNA [NM_015993]	**5.034**
GDF2	Homo sapiens growth differentiation factor 2 (GDF2), mRNA [NM_016204]	**4.960**
PRKAG3	Homo sapiens protein kinase, AMP-activated, mRNA [NM_017431]	**4.541**
C12orf68	Homo sapiens chromosome 12 open reading frame 68, mRNA [NM_001013635]	**4.528**
LOC401351	Homo sapiens cDNA FLJ46332 fis, clone TESTI4045470. [AK128835]	**4.507**
LOC729652	Homo sapiens mRNA; cDNA DKFZp434L055 (from clone DKFZp434L055). [AL834536]	**4.388**
HAPLN4	Homo sapiens hyaluronan and proteoglycan link protein 4 (HAPLN4), mRNA [NM_023002]	**4.341**
FGA	Homo sapiens fibrinogen alpha chain (FGA), mRNA [NM_000508]	**4.155**
COQ9	Homo sapiens cDNA PSEC0129 fis, clone PLACE1004170. [AK075438]	**4.081**
KCNS2	Homo sapiens potassium voltage-gated channel,(KCNS2), mRNA [NM_020697]	**3.826**
FLJ23834	Homo sapiens hypothetical protein FLJ23834 (FLJ23834), mRNA [NM_152750]	**3.774**
UNQ9419	Homo sapiens clone DNA180542 AHPA9419 (UNQ9419), mRNA [AY358263]	**3.691**
VWA5A	Homo sapiens von Willebrand factor A domain containing 5A (VWA5A), mRNA [NM_198315]	**3.528**
RBBP9	Homo sapiens retinoblastoma binding protein (RBBP9) mRNA, complete cds. [AF039564]	**3.519**
CHRDL1	Homo sapiens chordin-like 1 (CHRDL1), mRNA [NM_145234]	**3.321**
LOC647954	Homo sapiens misc_RNA (LOC647954), miscRNA [XR_018676]	**3.217**
ZNF718	Homo sapiens zinc finger protein 718 (ZNF718), mRNA [NM_001039127]	**3.131**
SERPINH1	Homo sapiens serpin peptidase inhibitor,(SERPINH1), mRNA [NM_001235]	**3.084**
POTEE	Homo sapiens POTE ankyrin domain family, member E (POTEE), mRNA [NM_001083538]	**3.061**
KRT8P15	Homo sapiens misc_RNA (KRT8P15), miscRNA [XR_016994]	**3.018**
LOC644936	Homo sapiens cytoplasmic beta-actin pseudogene (LOC644936), non-coding RNA [NR_004845]	**2.954**
POTEF	Homo sapiens POTE ankyrin domain family, member F (POTEF), mRNA [NM_001099771]	**2.911**
LOC100128116	Homo sapiens misc_RNA (LOC100128116), miscRNA [XR_038299]	**2.846**
LOC646976	Homo sapiens cDNA FLJ38763 fis, clone KIDNE2014119. [AK096082]	**2.838**

PACSIN3	Homo sapiens protein kinase C and casein kinase substrate in neurons 3 (PACSIN3), mRNA [NM_016223]	**2.829**
EPHA2	Homo sapiens EPH receptor A2 (EPHA2), mRNA [NM_004431]	**2.767**
GAS6	Homo sapiens growth arrest-specific 6 (GAS6), mRNA [NM_000820]	**2.753**
ZNF76	Homo sapiens zinc finger protein 76 (expressed in testis) (ZNF76), mRNA [NM_003427]	**2.747**
GPR4	Homo sapiens G protein-coupled receptor 4 (GPR4), mRNA [NM_005282]	**2.733**
EIF4G1	Homo sapiens eukaryotic translation initiation factor 4 gamma, 1 (EIF4G1), mRNA [NM_182917]	**2.720**
ACP2	Homo sapiens acid phosphatase 2, lysosomal (ACP2), mRNA [NM_001610]	**2.683**
ALDH5A1	Homo sapiens aldehyde dehydrogenase 5 family, member A1 (ALDH5A1), mRNA [NM_170740]	**2.657**
ATAD3B	Homo sapiens ATPase family, AAA domain containing 3B (ATAD3B), mRNA [NM_031921]	**2.621**
PLCB3	Homo sapiens phospholipase C, beta 3 (phosphatidylinositol-specific) (PLCB3), mRNA [NM_000932]	**2.618**
TUBB4	Homo sapiens tubulin, beta 4 (TUBB4), mRNA [NM_006087]	**2.603**
TOM1	Homo sapiens target of myb1 (chicken) (TOM1), mRNA [NM_005488]	**2.598**
HCFC1	Homo sapiens host cell factor C1 (VP16-accessory protein) (HCFC1), mRNA [NM_005334]	**2.597**
TUBA1A	Homo sapiens tubulin, alpha 1a (TUBA1A), mRNA [NM_006009]	**2.570**
EHBP1L1	Homo sapiens EH domain binding protein 1-like 1 (EHBP1L1), mRNA [NM_001099409]	**2.540**
SLC27A4	Homo sapiens solute carrier family 27 (fatty acid transporter), member 4 (SLC27A4), mRNA [NM_005094]	**2.536**
FLNA	Homo sapiens filamin A, alpha (actin binding protein 280) (FLNA), mRNA [NM_001456]	**2.521**
KRT13	Homo sapiens keratin 13 (KRT13), mRNA [NM_002274]	**2.518**
KRT17P3	Homo sapiens misc_RNA (KRT17P3), miscRNA [XR_019109]	**2.514**
ATPBD3	Homo sapiens ATP binding domain 3 (ATPBD3), mRNA [NM_145232]	**2.497**
LTBP3	Homo sapiens latent transforming growth factor beta binding protein 3 (LTBP3), mRNA [NM_021070]	**2.484**
PICK1	Homo sapiens protein interacting with PRKCA 1 (PICK1), mRNA [NM_012407]	**2.480**
FXR2	Homo sapiens fragile X mental retardation, autosomal homolog 2 (FXR2), mRNA [NM_004860]	**2.479**
POLR2E	Homo sapiens polymerase (RNA) II (DNA directed) polypeptide E, 25kDa (POLR2E), mRNA [NM_002695]	**2.470**
PTGES2	Homo sapiens prostaglandin E synthase 2 (PTGES2), mRNA [NM_025072]	**2.467**
NFIC	Homo sapiens nuclear factor I/C (CCAAT-binding transcription factor) (NFIC),mRNA [NM_005597]	**2.452**
ALKBH5	Homo sapiens alkB, alkylation repair homolog 5 (E. coli) (ALKBH5), mRNA [NM_017758]	**2.441**
TIMM44	Homo sapiens translocase of inner mitochondrial membrane 44 homolog (yeast)	**2.440**

	(TIMM44), mRNA [NM_006351]	
CDIPT	Homo sapiens CDP-diacylglycerol--inositol 3-phosphatidyltransferase(CDIPT), mRNA [NM_006319]	**2.437**
ANKRD31	Ankyrin repeat domain-containing protein 31 [Source:UniProtKB/ Swiss-Prot; Acc:Q8N7Z5] [ENST00000274361]	**2.427**
CALCOCO1	Homo sapiens calcium binding and coiled-coil domain 1 (CALCOCO1),mRNA [NM_020898]	**2.427**
GAPDH	Homo sapiens glyceraldehyde-3-phosphate dehydrogenase (GAPDH), mRNA [NM_002046]	**2.412**
LOC646048	Homo sapiens similar to cytoskeletal beta actin (LOC646048), mRNA [XR_017059]	**2.405**
CYTH2	Homo sapiens cytohesin 2 (CYTH2), mRNA [NM_004228]	**2.393**
ACTBL2	Homo sapiens actin, beta-like 2 (ACTBL2), mRNA [NM_001017992]	**2.387**
HDGF2	Homo sapiens hepatoma-derived growth factor-related protein 2 (HDGF2), mRNA [NM_032631]	**2.386**
MAPK12	Homo sapiens mitogen-activated protein kinase 12 (MAPK12), mRNA [NM_002969]	**2.361**
H1FX	Homo sapiens H1 histone family, member X (H1FX), mRNA [NM_006026]	**2.358**
GALNT2	Homo sapiens UDP-N-acetyl-alpha-D-galactosamine: polypeptide N-acetylgalactosaminyl transferase 2 (GalNAc-T2) (GALNT2), mRNA [NM_004481]	**2.350**
USP19	Homo sapiens ubiquitin specific peptidase 19 (USP19), mRNA [NM_006677]	**2.333**
MAF1	Homo sapiens MAF1 homolog (S. cerevisiae) (MAF1), mRNA [NM_032272]	**2.328**
USP5	Homo sapiens ubiquitin specific peptidase 5 (isopeptidase T) (USP5), mRNA [NM_003481]	**2.321**
DLSTP	E2k=alpha-ketoglutarate dehydrogenase complex dihydrolipoyl succinyltransferase [human, fetal brain, mRNA, 2987 nt]. [S72422]	**2.304**
KIFC3	Homo sapiens kinesin family member C3 (KIFC3), mRNA [NM_005550]	**2.298**
HLA-H	Homo sapiens major histocompatibility complex, class I, H (pseudogene) (HLA-H), non-coding RNA [NR_001434]	**2.297**
GBL	Homo sapiens G protein beta subunit-like (GBL), mRNA [NM_022372]	**2.296**
ACTB	Homo sapiens actin, beta (ACTB), mRNA [NM_001101]	**2.292**
BAP1	Homo sapiens BRCA1 associated protein-1 (ubiquitin carboxy-terminal hydrolase) (BAP1), mRNA [NM_004656]	**2.291**
THAP7	Homo sapiens THAP domain containing 7 (THAP7), mRNA [NM_030573]	**2.282**
CSK	Homo sapiens c-src tyrosine kinase (CSK), transcript variant 1, mRNA [NM_004383]	**2.260**
STARD3	Homo sapiens StAR-related lipid transfer (START) domain containing 3 (STARD3), mRNA [NM_006804]	**2.258**
RHOB	Homo sapiens ras homolog gene family, member B (RHOB), mRNA [NM_004040]	**2.239**
STK32C	Homo sapiens serine/threonine kinase 32C (STK32C), mRNA [NM_173575]	**2.227**
LOC407835	Homo sapiens mitogen-activated protein kinase kinase 2 pseudogene (LOC407835), non-coding RNA [NR_002144]	**2.223**
EDC4	Homo sapiens enhancer of mRNA decapping 4 (EDC4), mRNA [NM_014329]	**2.220**
ACTN1	Homo sapiens actinin, alpha 1 (ACTN1), mRNA [NM_001102]	**2.220**
LOC391334	Homo sapiens misc_RNA (LOC391334), miscRNA [XR_017601]	**2.208**
CEBPE	Homo sapiens CCAAT/enhancer binding protein (C/EBP), epsilon (CEBPE), mRNA	**2.192**

	[NM_001805]	
GIPC1	Homo sapiens GIPC PDZ domain containing family, member 1 (GIPC1),mRNA [NM_005716]	**2.189**
SMARCD2	Homo sapiens SWI/SNF related, matrix associated, subfamily d, member 2 (SMARCD2), mRNA [NM_003077]	**2.189**
SPTBN1	Homo sapiens spectrin, beta, non-erythrocytic 1 (SPTBN1), mRNA [NM_003128]	**2.185**
MGAT4B	Homo sapiens mannosyl (alpha-1,3-)-glycoprotein beta-1,4-N-acetylglucosaminyl transferase, isozyme B (MGAT4B), mRNA [NM_054013]	**2.181**
ACTN3	Homo sapiens actinin, alpha 3 (ACTN3), mRNA [NM_001104]	**2.175**
LOC399881	Homo sapiens cDNA FLJ44864 fis, clone BRALZ2013621, moderately similar to Heterogeneous nuclear ribonucleoprotein K. [AK126814]	**2.174**
RAB8A	Homo sapiens RAB8A, member RAS oncogene family (RAB8A), mRNA [NM_005370]	**2.169**
CREB3	Homo sapiens cAMP responsive element binding protein 3 (CREB3), mRNA [NM_006368]	**2.164**
HDHD3	Homo sapiens haloacid dehalogenase-like hydrolase domain containing 3 (HDHD3), mRNA [NM_031219]	**2.154**
TAX1BP3	Homo sapiens Tax1 (human T-cell leukemia virus type I) binding protein 3 (TAX1BP3), mRNA [NM_014604]	**2.152**
MSLN	Homo sapiens mesothelin (MSLN), mRNA [NM_005823]	**2.147**
NAP1L4	Homo sapiens nucleosome assembly protein 1-like 4 (NAP1L4), mRNA [NM_005969]	**2.134**
INPPL1	Homo sapiens inositol polyphosphate phosphatase-like 1 (INPPL1), mRNA [NM_001567]	**2.131**
AGPAT2	Homo sapiens 1-acylglycerol-3-phosphate O-acyltransferase 2 (lysophosphatidic acid acyltransferase, beta) (AGPAT2), mRNA [NM_006412]	**2.128**
KLHDC4	Homo sapiens kelch domain containing 4 (KLHDC4), mRNA [NM_017566]	**2.127**
HOXC5	Homo sapiens homeobox C5 (HOXC5), mRNA [NM_018953]	**2.126**
JUN	Homo sapiens jun oncogene (JUN), mRNA [NM_002228]	**2.125**
LOC100134282	Homo sapiens cDNA FLJ27195 fis, clone SYN02786. [AK130705]	**2.122**
ENO1	Homo sapiens enolase 1, (alpha) (ENO1), mRNA [NM_001428]	**2.115**
TOMM40	Homo sapiens translocase of outer mitochondrial membrane 40 homolog (yeast) (TOMM40), nuclear gene encoding mitochondrial protein, mRNA [NM_006114]	**2.113**
SERPINE1	Homo sapiens serpin peptidase inhibitor, clade E (nexin, plasminogen activator inhibitor type 1), member 1 (SERPINE1), mRNA [NM_000602]	**2.111**
TBL3	Homo sapiens transducin (beta)-like 3 (TBL3), mRNA [NM_006453]	**2.111**
GRAMD1A	Homo sapiens GRAM domain containing 1A (GRAMD1A), mRNA [NM_020895]	**2.103**
ZNF445	Homo sapiens zinc finger protein 445 (ZNF445), mRNA [NM_181489]	**2.102**
QARS	Homo sapiens glutaminyl-tRNA synthetase (QARS), mRNA [NM_005051]	**2.100**
SLC1A5	Homo sapiens solute carrier family 1 (neutral amino acid transporter), member 5 (SLC1A5), mRNA [NM_005628]	**2.091**
CCND1	Homo sapiens cyclin D1 (CCND1), mRNA [NM_053056]	**2.091**
MAP2K2	Homo sapiens mitogen-activated protein kinase kinase 2 (MAP2K2), mRNA [NM_030662]	**2.090**
YARS	Homo sapiens tyrosyl-tRNA synthetase (YARS), mRNA [NM_003680]	**2.090**

HSPB8	Homo sapiens heat shock 22kDa protein 8 (HSPB8), mRNA [NM_014365]	**2.084**
IRAK1	Homo sapiens interleukin-1 receptor-associated kinase 1 (IRAK1), mRNA [NM_001569]	**2.084**
CLDN4	Homo sapiens claudin 4 (CLDN4), mRNA [NM_001305]	**2.082**
HNRNPAB	Homo sapiens heterogeneous nuclear ribonucleoprotein A/B (HNRNPAB),mRNA [NM_004499]	**2.082**
TNFSF9	Homo sapiens tumor necrosis factor (ligand) superfamily, member 9 (TNFSF9), mRNA [NM_003811]	**2.081**
AAMP	Homo sapiens angio-associated, migratory cell protein (AAMP), mRNA [NM_001087]	**2.080**
APP	Homo sapiens amyloid beta (A4) precursor protein (APP), mRNA [NM_000484]	**2.078**
EEF2	Homo sapiens eukaryotic translation elongation factor 2 (EEF2), mRNA [NM_001961]	**2.077**
RUVBL2	Homo sapiens RuvB-like 2 (E. coli) (RUVBL2), mRNA [NM_006666]	**2.073**
EPHB4	Homo sapiens EPH receptor B4 (EPHB4), mRNA [NM_004444]	**2.065**
KRT33A	Homo sapiens keratin 33A (KRT33A), mRNA [NM_004138]	**2.061**
ARFGAP2	Homo sapiens ADP-ribosylation factor GTPase activating protein 2 (ARFGAP2), mRNA [NM_032389]	**2.054**
APH1A	Homo sapiens anterior pharynx defective 1 homolog A (C. elegans) (APH1A),mRNA [NM_001077628]	**2.048**
FLJ46111	Homo sapiens FLJ46111 protein, mRNA (cDNA clone IMAGE:8327474). [BC112006]	**2.047**
IDH2	Homo sapiens isocitrate dehydrogenase 2 (NADP+), mitochondrial (IDH2),mRNA [NM_002168]	**2.046**
SDHALP1	Homo sapiens succinate dehydrogenase complex, subunit A, flavoprotein pseudogene 1 (SDHALP1), non-coding RNA [NR_003264]	**2.042**
RASA3	Homo sapiens mRNA for Ins(1,3,4,5)P4-binding protein. [X89399]	**2.041**
DBNL	Homo sapiens drebrin-like (DBNL), mRNA [NM_014063]	**2.031**
NUMA1	Homo sapiens nuclear mitotic apparatus protein 1 (NUMA1), mRNA [NM_006185]	**2.029**
C19orf48	Homo sapiens chromosome 19 open reading frame 48 (C19orf48), mRNA [NM_199249]	**2.025**
TNIP2	Homo sapiens TNFAIP3 interacting protein 2 (TNIP2), mRNA [NM_024309]	**2.023**
TMEM161A	Homo sapiens transmembrane protein 161A (TMEM161A), mRNA [NM_017814]	**2.020**
MCAM	Homo sapiens melanoma cell adhesion molecule (MCAM), mRNA [NM_006500]	**2.020**
WDR24	Homo sapiens WD repeat domain 24 (WDR24), mRNA [NM_032259]	**2.018**
HPCAL1	Homo sapiens hippocalcin-like 1 (HPCAL1), mRNA [NM_134421]	**2.015**
MVD	Homo sapiens mevalonate (diphospho) decarboxylase (MVD), mRNA [NM_002461]	**2.014**
PLOD1	Homo sapiens procollagen-lysine 1, 2-oxoglutarate 5-dioxygenase 1 (PLOD1), mRNA [NM_000302]	**2.014**
DNASE2	Homo sapiens deoxyribonuclease II, lysosomal (DNASE2), mRNA [NM_001375]	**2.011**
GNB2	Homo sapiens guanine nucleotide binding protein (G protein), beta polypeptide 2 (GNB2), mRNA [NM_005273]	**2.011**
LOC148709	Homo sapiens actin pseudogene (LOC148709), non-coding RNA [NR_002929]	**2.009**
ARAF	Homo sapiens v-raf murine sarcoma 3611 viral oncogene homolog (ARAF), mRNA [NM_001654]	**2.000**
SH2D3A	Homo sapiens SH2 domain containing 3A (SH2D3A), mRNA [NM_005490]	**2.000**
ADRM1	Homo sapiens adhesion regulating molecule 1 (ADRM1), mRNA [NM_007002]	**1.999**
KIF7	Homo sapiens kinesin family member 7 (KIF7), mRNA [NM_198525]	**1.999**

LOC100128108	Homo sapiens hypothetical protein LOC100128108 (LOC100128108), mRNA [XM_001721901]	**1.999**
ECE1	Homo sapiens endothelin converting enzyme 1 (ECE1), mRNA [NM_001397]	**1.993**
LOC100134400	Homo sapiens hypothetical protein LOC100134400 (LOC100134400), partial mRNA [XM_001718660]	**1.991**
KRT16	Homo sapiens keratin 16 (KRT16), mRNA [NM_005557]	**1.989**
GPAA1	Homo sapiens glycosylphosphatidylinositol anchor attachment protein 1 homolog (yeast) (GPAA1), mRNA [NM_003801]	**1.985**
INTS5	Homo sapiens integrator complex subunit 5 (INTS5), mRNA [NM_030628]	**1.985**
TUBG1	Homo sapiens tubulin, gamma 1 (TUBG1), mRNA [NM_001070]	**1.982**
PI4KAP2	Homo sapiens phosphatidylinositol 4-kinase, catalytic, alpha pseudogene 2 (PI4KAP2), non-coding RNA [NR_003700]	**1.981**
SNX17	Homo sapiens sorting nexin 17 (SNX17), mRNA [NM_014748]	**1.980**
GPS1	Homo sapiens G protein pathway suppressor 1 (GPS1), mRNA [NM_212492]	**1.976**
AP1B1	Homo sapiens adaptor-related protein complex 1, beta 1 subunit (AP1B1),mRNA [NM_001127]	**1.975**
NEIL2	Homo sapiens nei like 2 (E. coli) (NEIL2), transcript variant 1, mRNA [NM_145043]	**1.973**
GNAI2	Homo sapiens guanine nucleotide binding protein (G protein), alpha inhibiting activity polypeptide 2 (GNAI2), mRNA [NM_002070]	**1.972**
TGFB1I1	Homo sapiens transforming growth factor beta 1 induced transcript 1 (TGFB1I1), mRNA [NM_001042454]	**1.970**
SFN	Homo sapiens stratifin (SFN), mRNA [NM_006142]	**1.966**
DAK	Homo sapiens dihydroxyacetone kinase 2 homolog (S. cerevisiae) (DAK), mRNA [NM_015533]	**1.963**
NFRKB	Homo sapiens nuclear factor related to kappaB binding protein (NFRKB),mRNA [NM_006165]	**1.963**
G6PD	Homo sapiens glucose-6-phosphate dehydrogenase (G6PD), mRNA [NM_000402]	**1.962**
ELF3	Homo sapiens E74-like factor 3 (ets domain transcription factor, epithelial-specific) (ELF3), mRNA [NM_004433]	**1.961**
KRT7	Homo sapiens keratin 7 (KRT7), mRNA [NM_005556]	**1.958**
ORM1	Homo sapiens orosomucoid 1 (ORM1), mRNA [NM_000607]	**1.955**
CLN6	Homo sapiens ceroid-lipofuscinosis, neuronal 6, late infantile, variant (CLN6), mRNA [NM_017882]	**1.950**
TWF2	Homo sapiens twinfilin, actin-binding protein, homolog 2 (Drosophila) (TWF2), mRNA [NM_007284]	**1.949**
HSPA1A	Homo sapiens heat shock 70kDa protein 1A (HSPA1A), mRNA [NM_005345]	**1.949**
CCDC85B	Homo sapiens coiled-coil domain containing 85B (CCDC85B), mRNA [NM_006848]	**1.947**
HSD3B7	Homo sapiens mRNA; cDNA DKFZp761C1717 (from clone DKFZp761C1717). [AL834347]	**1.946**
LOC220729	Homo sapiens succinate dehydrogenase complex, subunit A, flavoprotein pseudogene (LOC220729), non-coding RNA [NR_003266]	**1.941**
PSAP	Homo sapiens prosaposin (PSAP), transcript variant 2, mRNA [NM_001042465]	**1.940**
FLNC	Homo sapiens filamin C, gamma (actin binding protein 280) (FLNC), mRNA	**1.939**

	[NM_001458]	
RANGAP1	Homo sapiens Ran GTPase activating protein 1 (RANGAP1), mRNA [NM_002883]	**1.938**
MFAP2	Homo sapiens microfibrillar-associated protein 2 (MFAP2), mRNA [NM_017459]	**1.928**
CHD4	Homo sapiens chromodomain helicase DNA binding protein 4 (CHD4), mRNA [NM_001273]	**1.927**
FASN	Homo sapiens fatty acid synthase (FASN), mRNA [NM_004104]	**1.927**
DHX38	Homo sapiens DEAH (Asp-Glu-Ala-His) box polypeptide 38 (DHX38), mRNA [NM_014003]	**1.926**
SLC4A2	Homo sapiens solute carrier family 4, anion exchanger, member 2 (erythrocyte membrane protein band 3-like 1) (SLC4A2), mRNA [NM_003040]	**1.926**
NR2F6	Homo sapiens nuclear receptor subfamily 2, group F, member 6 (NR2F6), mRNA [NM_005234]	**1.925**
VARS	Homo sapiens valyl-tRNA synthetase (VARS), nuclear gene encoding mitochondrial protein, mRNA [NM_006295]	**1.925**
DSG2	Homo sapiens desmoglein 2 (DSG2), mRNA [NM_001943]	**1.919**
TOP3B	Homo sapiens topoisomerase (DNA) III beta (TOP3B), mRNA [NM_003935]	**1.918**
PEF1	Homo sapiens penta-EF-hand domain containing 1 (PEF1), mRNA [NM_012392]	**1.914**
RRBP1	Homo sapiens ribosome binding protein 1 homolog 180kDa (dog) (RRBP1),mRNA [NM_001042576]	**1.914**
CDC37	Homo sapiens cell division cycle 37 homolog (S. cerevisiae) (CDC37), mRNA [NM_007065]	**1.910**
PEX16	Homo sapiens peroxisomal biogenesis factor 16 (PEX16), mRNA [NM_004813]	**1.910**
AGPAT6	Homo sapiens 1-acylglycerol-3-phosphate O-acyltransferase 6 (lysophosphatidic acid acyltransferase, zeta) (AGPAT6), mRNA [NM_178819]	**1.906**
TMEM106A	Homo sapiens transmembrane protein 106A (TMEM106A), mRNA [NM_145041]	**1.901**
ZNHIT2	Homo sapiens zinc finger, HIT type 2 (ZNHIT2), mRNA [NM_014205]	**1.900**
C16orf42	Homo sapiens chromosome 16 open reading frame 42 (C16orf42), mRNA [NM_001001410]	**1.898**
PSMD2	Homo sapiens proteasome (prosome, macropain) 26S subunit, non-ATPase, 2 (PSMD2), mRNA [NM_002808]	**1.897**
UNC84B	Homo sapiens unc-84 homolog B (C. elegans) (UNC84B), mRNA [NM_015374]	**1.896**
PITPNM1	Homo sapiens phosphatidylinositol transfer protein, membrane-associated 1 (PITPNM1), mRNA [NM_004910]	**1.892**
RRP12	Homo sapiens ribosomal RNA processing 12 homolog (S. cerevisiae) (RRP12), mRNA [NM_015179]	**1.890**
AFG3L2	Homo sapiens AFG3 ATPase family gene 3-like 2 (yeast) (AFG3L2), mRNA [NM_006796]	**1.889**
ASH1L	Homo sapiens ash1 (absent, small, or homeotic)-like (Drosophila) (ASH1L), mRNA [NM_018489]	**1.888**
RAG1AP1	Homo sapiens recombination activating gene 1 activating protein 1 (RAG1AP1), mRNA [NM_018845]	**1.888**
MOV10	Homo sapiens Mov10, Moloney leukemia virus 10, homolog (mouse) (MOV10), mRNA [NM_020963]	**1.887**

ALDOAP2	Human aldolase pseudogene mRNA, complete cds. [M21191]	**1.883**
ZBED1	Homo sapiens zinc finger, BED-type containing 1 (ZBED1), mRNA [NM_004729]	**1.882**
LOC100132673	Homo sapiens misc_RNA (LOC100132673), miscRNA [XR_039018]	**1.879**
KIAA0664	Homo sapiens KIAA0664 (KIAA0664), mRNA [NM_015229]	**1.877**
WNT7B	Homo sapiens wingless-type MMTV integration site family, member 7B (WNT7B), mRNA [NM_058238]	**1.875**
LAMA5	Homo sapiens laminin, alpha 5 (LAMA5), mRNA [NM_005560]	**1.872**
CHST10	Homo sapiens carbohydrate sulfotransferase 10 (CHST10), mRNA [NM_004854]	**1.871**
ZNF581	Homo sapiens zinc finger protein 581 (ZNF581), mRNA [NM_016535]	**1.871**
LOC400036	Homo sapiens misc_RNA (LOC400036), miscRNA [XR_042386]	**1.870**
TRAFD1	Homo sapiens TRAF-type zinc finger domain containing 1 (TRAFD1),mRNA [NM_006700]	**1.869**
HPCA	Homo sapiens hippocalcin (HPCA), mRNA [NM_002143]	**1.868**
CNN2	Homo sapiens calponin 2 (CNN2), mRNA [NM_004368]	**1.868**
ATP1A3	Homo sapiens ATPase, Na+/K+ transporting, alpha 3 polypeptide (ATP1A3), mRNA [NM_152296]	**1.862**
TUBA1C	Homo sapiens tubulin, alpha 1c (TUBA1C), mRNA [NM_032704]	**1.862**
EGLN2	Homo sapiens egl nine homolog 2 (C. elegans) (EGLN2), mRNA [NM_080732]	**1.860**
SLC38A5	Homo sapiens solute carrier family 38, member 5 (SLC38A5), mRNA [NM_033518]	**1.859**
RPS6KA1	Homo sapiens ribosomal protein S6 kinase, 90kDa, polypeptide 1 (RPS6KA1), mRNA [NM_002953]	**1.858**
HDGF	Homo sapiens hepatoma-derived growth factor (high-mobility group protein 1-like) (HDGF), mRNA [NM_004494]	**1.857**
CBX4	Homo sapiens chromobox homolog 4 (Pc class homolog, Drosophila) (CBX4), mRNA [NM_003655]	**1.856**
SEC24C	Homo sapiens SEC24 family, member C (S. cerevisiae) (SEC24C), mRNA [NM_004922]	**1.856**
HLA-A	Homo sapiens major histocompatibility complex, class I, A (HLA-A), mRNA [NM_002116]	**1.855**
IGHMBP2	Homo sapiens immunoglobulin mu binding protein 2 (IGHMBP2), mRNA [NM_002180]	**1.854**
LIMD1	Homo sapiens LIM domains containing 1 (LIMD1), mRNA [NM_014240]	**1.852**
TRAP1	Homo sapiens TNF receptor-associated protein 1 (TRAP1), mRNA [NM_016292]	**1.844**
ATP1A4	Homo sapiens cDNA FLJ40757 fis, clone TRACH2001996, highly similar to Sodium/Potassium-Transporting Atpase Alpha-1 Chain Precursor(EC 3.6.1.37). [AK098076]	**1.844**
DOHH	Homo sapiens deoxyhypusine hydroxylase/monooxygenase (DOHH), mRNA [NM_031304]	**1.836**
LOC648740	Homo sapiens ACTB pseudogene (LOC648740), non-coding RNA [NR_024438]	**1.835**
FEM1A	Homo sapiens fem-1 homolog a (C. elegans) (FEM1A), mRNA [NM_018708]	**1.834**
SLC39A1	Homo sapiens solute carrier family 39 (zinc transporter), member 1 (SLC39A1), mRNA [NM_014437]	**1.833**
ADAM15	Homo sapiens ADAM metallopeptidase domain 15 (ADAM15), mRNA [NM_207191]	**1.830**

RPL18A	Homo sapiens ribosomal protein L18a (RPL18A), mRNA [NM_000980]	**1.830**
PPP1CA	Homo sapiens protein phosphatase 1, catalytic subunit, alpha isoform (PPP1CA), mRNA [NM_001008709]	**1.828**
TP53I13	Homo sapiens tumor protein p53 inducible protein 13 (TP53I13), mRNA [NM_138349]	**1.828**
SFRS9	Homo sapiens splicing factor, arginine/serine-rich 9 (SFRS9), mRNA [NM_003769]	**1.826**
TMED1	Homo sapiens transmembrane emp24 protein transport domain containing 1 (TMED1), mRNA [NM_006858]	**1.826**
NBPF10	Homo sapiens neuroblastoma breakpoint family, member 10 (NBPF10), mRNA [NM_001039703]	**1.826**
ATAD3A	Homo sapiens ATPase family, AAA domain containing 3A (ATAD3A), mRNA [NM_018188]	**1.825**
INSR	Homo sapiens insulin receptor (INSR), transcript variant 1, mRNA [NM_000208]	**1.823**
IGFBP3	Homo sapiens insulin-like growth factor binding protein 3 (IGFBP3),mRNA [NM_001013398]	**1.822**
PPP4C	Homo sapiens protein phosphatase 4 (formerly X), catalytic subunit (PPP4C), mRNA [NM_002720]	**1.821**
RRP7A	Homo sapiens ribosomal RNA processing 7 homolog A (S. cerevisiae), mRNA (cDNA clone IMAGE:4394667), partial cds. [BC031838]	**1.820**
SPNS1	Homo sapiens spinster homolog 1 (Drosophila) (SPNS1), mRNA [NM_032038]	**1.819**
TRPC4AP	Homo sapiens transient receptor potential cation channel, subfamily C, member 4 associated protein (TRPC4AP), mRNA [NM_015638]	**1.819**
NOMO1	Homo sapiens NODAL modulator 1 (NOMO1), mRNA [NM_014287]	**1.818**
GNB2L1	Homo sapiens guanine nucleotide binding protein (G protein), beta polypeptide 2-like 1 (GNB2L1), mRNA [NM_006098]	**1.812**
SLC12A8	Homo sapiens solute carrier family 12 (potassium/chloride transporters), member 8 (SLC12A8), mRNA [NM_024628]	**1.811**
CHD3	Homo sapiens chromodomain helicase DNA binding protein 3 (CHD3),mRNA [NM_001005273]	**1.810**
SIRT6	Homo sapiens sirtuin (silent mating type information regulation 2 homolog) 6 (S. cerevisiae) (SIRT6), mRNA [NM_016539]	**1.809**
FAM46B	Homo sapiens family with sequence similarity 46, member B (FAM46B), mRNA [NM_052943]	**1.804**
FOXRED1	Homo sapiens FAD-dependent oxidoreductase domain containing 1 (FOXRED1), mRNA [NM_017547]	**1.804**
RPS6KB2	Homo sapiens ribosomal protein S6 kinase, polypeptide 2 (RPS6KB2), mRNA [NM_003952]	**1.802**
MED16	Homo sapiens mediator complex subunit 16 (MED16), mRNA [NM_005481]	**1.801**
KRT8	Homo sapiens keratin 8 (KRT8), mRNA [NM_002273]	**1.801**
DDX49	Homo sapiens DEAD (Asp-Glu-Ala-Asp) box polypeptide 49 (DDX49), mRNA [NM_019070]	**1.797**
WDR46	Homo sapiens WD repeat domain 46 (WDR46), mRNA [NM_005452]	**1.796**
DGCR6	Homo sapiens DiGeorge syndrome critical region gene 6 (DGCR6), mRNA [NM_005675]	**1.795**

NCAPH2	Homo sapiens non-SMC condensin II complex, subunit H2 (NCAPH2), mRNA [NM_152299]	**1.795**
UNQ1870	Homo sapiens clone DNA62312 GALI1870 (UNQ1870) mRNA, [AY358688]	**1.794**
CPNE1	Homo sapiens copine I (CPNE1), mRNA [NM_152930]	**1.792**
NRBP1	Homo sapiens nuclear receptor binding protein 1 (NRBP1), mRNA [NM_013392]	**1.791**
DKFZp761E198	Homo sapiens DKFZp761E198 protein (DKFZp761E198), mRNA [NM_138368]	**1.790**
LOC392787	Homo sapiens misc_RNA (LOC392787), miscRNA [XR_018580]	**1.790**
KRT17	Homo sapiens keratin 17 (KRT17), mRNA [NM_000422]	**1.789**
AUP1	Homo sapiens ancient ubiquitous protein 1 (AUP1), mRNA [NM_181575]	**1.788**
CYBA	Homo sapiens cytochrome b-245, alpha polypeptide, mRNA (cDNA clone IMAGE:5217162), [BC028224]	**1.788**
PPP1R12C	Homo sapiens protein phosphatase 1, regulatory (inhibitor) subunit 12C (PPP1R12C), mRNA [NM_017607]	**1.788**
ZDHHC12	Homo sapiens zinc finger, DHHC-type containing 12 (ZDHHC12), mRNA [NM_032799]	**1.788**
KIAA1161	Homo sapiens KIAA1161 (KIAA1161), mRNA [NM_020702]	**1.782**
PGAM5	Homo sapiens phosphoglycerate mutase family member 5 (PGAM5), mRNA [NM_138575]	**1.780**
PABPC4	Homo sapiens poly(A) binding protein, cytoplasmic 4 (inducible form) (PABPC4), mRNA [NM_003819]	**1.779**
MRPL28	Homo sapiens mitochondrial ribosomal protein L28 (MRPL28), mRNA [NM_006428]	**1.775**
ZNF768	Homo sapiens zinc finger protein 768 (ZNF768), mRNA [NM_024671]	**1.774**
TRIM16L	Homo sapiens tripartite motif-containing 16-like (TRIM16L), mRNA [NM_001037330]	**1.773**
FOS	Homo sapiens v-fos FBJ murine osteosarcoma viral oncogene homolog (FOS), mRNA [NM_005252]	**1.772**
RAB40C	Homo sapiens RAB40C, member RAS oncogene family (RAB40C), mRNA [NM_021168]	**1.771**
TMEM115	Homo sapiens transmembrane protein 115 (TMEM115), mRNA [NM_007024]	**1.770**
PGD	Homo sapiens phosphogluconate dehydrogenase (PGD), mRNA [NM_002631]	**1.769**
CCDC137	Homo sapiens coiled-coil domain containing 137 (CCDC137), mRNA [NM_199287]	**1.768**
PPP5C	Homo sapiens protein phosphatase 5, catalytic subunit (PPP5C), mRNA [NM_006247]	**1.766**
CRK	Homo sapiens v-crk sarcoma virus CT10 oncogene homolog (avian) (CRK),mRNA [NM_016823]	**1.766**
NUBP2	Homo sapiens nucleotide binding protein 2 (MinD homolog, E. coli) (NUBP2), mRNA [NM_012225]	**1.764**
MAGEA4	Homo sapiens melanoma antigen family A, 4 (MAGEA4), mRNA [NM_002362]	**1.763**
TMED9	Homo sapiens transmembrane emp24 protein transport domain containing 9 (TMED9), mRNA [NM_017510]	**1.763**
EEF1A1	Homo sapiens eukaryotic translation elongation factor 1 alpha 1 (EEF1A1), mRNA [NM_001402]	**1.762**
TEF	Homo sapiens thyrotrophic embryonic factor (TEF), mRNA [NM_003216]	**1.761**
TSR2	Homo sapiens TSR2, 20S rRNA accumulation, homolog (S. cerevisiae) (TSR2), mRNA [NM_058163]	**1.760**

TSPAN33	Homo sapiens tetraspanin 33 (TSPAN33), mRNA [NM_178562]	**1.755**
FAM125A	Homo sapiens family with sequence similarity 125, member A (FAM125A), mRNA [NM_138401]	**1.754**
MAP4	Homo sapiens microtubule-associated protein 4 (MAP4), trmRNA [NM_002375]	**1.754**
RALY	Homo sapiens RNA binding protein, autoantigenic (hnRNP-associated with lethal yellow homolog (mouse)) (RALY), mRNA [NM_016732]	**1.750**
TET3	Homo sapiens tet oncogene family member 3 (TET3), mRNA [NM_144993]	**1.749**
GPC1	Homo sapiens glypican 1 (GPC1), mRNA [NM_002081]	**1.747**
KIAA0415	Homo sapiens KIAA0415 (KIAA0415), mRNA [NM_014855]	**1.744**
LASP1	Homo sapiens LIM and SH3 protein 1 (LASP1), mRNA [NM_006148]	**1.744**
CPSF1	Homo sapiens cleavage and polyadenylation specific factor 1, 160kDa (CPSF1), mRNA [NM_013291]	**1.743**
PLD3	Homo sapiens phospholipase D family, member 3 (PLD3), mRNA [NM_012268]	**1.743**
LTBR	Homo sapiens lymphotoxin beta receptor (TNFR superfamily, member 3) (LTBR), mRNA [NM_002342]	**1.742**
SAFB	Homo sapiens scaffold attachment factor B (SAFB), mRNA [NM_002967]	**1.742**
STAT3	Homo sapiens signal transducer and activator of transcription 3 (acute-phase response factor) (STAT3), mRNA [NM_213662]	**1.742**
DPP3	Homo sapiens dipeptidyl-peptidase 3 (DPP3), mRNA [NM_130443]	**1.739**
LPCAT3	Homo sapiens lysophosphatidylcholine acyltransferase 3 (LPCAT3), mRNA [NM_005768]	**1.739**
TUBG2	Homo sapiens tubulin, gamma 2 (TUBG2), mRNA [NM_016437]	**1.739**
ASS1	Homo sapiens argininosuccinate synthetase 1 (ASS1), mRNA [NM_000050]	**1.736**
IL17RC	Homo sapiens interleukin 17 receptor C (IL17RC), mRNA [NM_153461]	**1.735**
FSCN1	Homo sapiens fascin homolog 1, actin-bundling protein (Strongylocentrotus purpuratus) (FSCN1), mRNA [NM_003088]	**1.733**
RDH13	Homo sapiens retinol dehydrogenase 13 (all-trans/9-cis) (RDH13), mRNA [NM_138412]	**1.732**
LOC441016	Homo sapiens hypothetical LOC441016 (LOC441016), mRNA [XM_001714867]	**1.730**
GTPBP5	Homo sapiens GTP binding protein 5 (putative) (GTPBP5), mRNA [NM_015666]	**1.728**
TSPO	Homo sapiens translocator protein (18kDa) (TSPO), mRNA [NM_000714]	**1.727**
HSP90AB1	Homo sapiens heat shock protein 90kDa alpha (cytosolic), class B member 1 (HSP90AB1), mRNA [NM_007355]	**1.724**
KIAA1967	Homo sapiens KIAA1967 (KIAA1967), transcript variant 1, mRNA [NM_021174]	**1.723**
CCDC80	Homo sapiens coiled-coil domain containing 80 (CCDC80), mRNA [NM_199511]	**1.723**
PLAUR	Homo sapiens plasminogen activator, urokinase receptor (PLAUR),mRNA [NM_001005377]	**1.722**
CSRP1	Homo sapiens cysteine and glycine-rich protein 1 (CSRP1), mRNA [NM_004078]	**1.717**
SLC39A3	Homo sapiens solute carrier family 39 (zinc transporter), member 3 (SLC39A3), mRNA [NM_213568]	**1.716**
GTF2F1	Homo sapiens general transcription factor IIF, polypeptide 1, 74kDa (GTF2F1), mRNA [NM_002096]	**1.713**
COQ4	Homo sapiens coenzyme Q4 homolog (S. cerevisiae) (COQ4), mRNA [NM_016035]	**1.712**
GAK	Homo sapiens cyclin G associated kinase (GAK), mRNA [NM_005255]	**1.712**

MED24	Homo sapiens mediator complex subunit 24 (MED24), mRNA [NM_014815]	**1.712**
TSHZ3	Homo sapiens teashirt zinc finger homeobox 3 (TSHZ3), mRNA [NM_020856]	**1.710**
SC65	Homo sapiens synaptonemal complex protein SC65 (SC65), mRNA [NM_006455]	**1.709**
NR1H2	Homo sapiens nuclear receptor subfamily 1, group H, member 2 (NR1H2), mRNA [NM_007121]	**1.708**
RUSC2	Homo sapiens RUN and SH3 domain containing 2 (RUSC2), mRNA [NM_014806]	**1.707**
ACOT7	Homo sapiens acyl-CoA thioesterase 7 (ACOT7), mRNA [NM_007274]	**1.706**
HABP4	Homo sapiens hyaluronan binding protein 4 (HABP4), mRNA [NM_014282]	**1.706**
LLGL2	Homo sapiens lethal giant larvae homolog 2 (Drosophila) (LLGL2), mRNA [NM_001015002]	**1.706**
RNPEP	Homo sapiens arginyl aminopeptidase (aminopeptidase B) (RNPEP), mRNA [NM_020216]	**1.706**
MRFAP1	Homo sapiens Mof4 family associated protein 1 (MRFAP1), mRNA [NM_033296]	**1.705**
DAG1	Homo sapiens dystroglycan 1 (dystrophin-associated glycoprotein 1) (DAG1), mRNA [NM_004393]	**1.702**
MCOLN1	Homo sapiens mucolipin 1 (MCOLN1), mRNA [NM_020533]	**1.701**
TRMT2B	Homo sapiens TRM2 tRNA methyltransferase 2 homolog B (S. cerevisiae) (TRMT2B), mRNA [NM_024917]	**1.701**
MTX1	Homo sapiens metaxin 1 (MTX1), mRNA [NM_198883]	**1.698**
TXNRD1	Homo sapiens thioredoxin reductase 1 (TXNRD1), mRNA [NM_003330]	**1.696**
RPL8	Homo sapiens ribosomal protein L8 (RPL8), mRNA [NM_000973]	**1.695**
MKL1	Homo sapiens megakaryoblastic leukemia (translocation) 1 (MKL1), mRNA [NM_020831]	**1.694**
ORAI2	Homo sapiens ORAI calcium release-activated calcium modulator 2 (ORAI2), mRNA [NM_032831]	**1.694**
PES1	Homo sapiens pescadillo homolog 1, containing BRCT domain (zebrafish) (PES1), mRNA [NM_014303]	**1.694**
ABCA7	Homo sapiens ATP-binding cassette, sub-family A (ABC1), member 7 (ABCA7), mRNA [NM_019112]	**1.689**
IER5L	Homo sapiens immediate early response 5-like (IER5L), mRNA [NM_203434]	**1.689**
SSNA1	Homo sapiens Sjogren syndrome nuclear autoantigen 1 (SSNA1), mRNA [NM_003731]	**1.689**
CTSD	Homo sapiens cathepsin D (CTSD), mRNA [NM_001909]	**1.686**
C3orf21	Homo sapiens chromosome 3 open reading frame 21 (C3orf21), mRNA [NM_152531]	**1.685**
LMAN2	Homo sapiens lectin, mannose-binding 2 (LMAN2), mRNA [NM_006816]	**1.685**
TUBGCP2	Homo sapiens tubulin, gamma complex associated protein 2 (TUBGCP2), mRNA [NM_006659]	**1.683**
TYSND1	Homo sapiens trypsin domain containing 1 (TYSND1), mRNA [NM_173555]	**1.682**
PTOV1	Homo sapiens prostate tumor overexpressed 1 (PTOV1), mRNA [NM_017432]	**1.680**
COL7A1	Homo sapiens collagen, type VII, alpha 1 (COL7A1), mRNA [NM_000094]	**1.676**
MDN1	Homo sapiens MDN1, midasin homolog (yeast) (MDN1), mRNA [NM_014611]	**1.676**
LOC100131929	Homo sapiens cDNA FLJ44475 fis, clone UTERU2031521. [AK126439]	**1.675**
LY6G6F	Homo sapiens lymphocyte antigen 6 complex, locus G6F (LY6G6F), mRNA [NM_001003693]	**1.672**

EWSR1	Homo sapiens Ewing sarcoma breakpoint region 1 (EWSR1), mRNA [NM_013986]	**1.669**
GALK1	Homo sapiens galactokinase 1 (GALK1), mRNA [NM_000154]	**1.669**
RBM9	Homo sapiens RNA binding motif protein 9 (RBM9), mRNA [NM_001031695]	**1.669**
B4GALT7	Homo sapiens xylosylprotein beta 1,4-galactosyltransferase, polypeptide 7 (galactosyltransferase I) (B4GALT7), mRNA [NM_007255]	**1.668**
hCG_1988300	full-length cDNA clone CS0DI072YA21 of Placenta Cot 25-normalized of Homo sapiens (human). [CR613736]	**1.668**
HLA-B	full-length cDNA clone CS0DG002YJ10 of B cells (Ramos cell line) of Homo sapiens (human). [CR608347]	**1.664**
MRC2	Homo sapiens mannose receptor, C type 2 (MRC2), mRNA [NM_006039]	**1.663**
DGCR6L	Homo sapiens DiGeorge syndrome critical region gene 6-like (DGCR6L), mRNA [NM_033257]	**1.662**
TRPM2	Homo sapiens transient receptor potential cation channel, subfamily M, member 2 (TRPM2), mRNA [NM_003307]	**1.662**
TXNRD2	Homo sapiens mitochondrial thioredoxin reductase (TRXR2A) mRNA, complete cds, alternatively spliced; nuclear gene for mitochondrial product. [AF201385]	**1.661**
ASCC2	Homo sapiens activating signal cointegrator 1 complex subunit 2 (ASCC2), mRNA [NM_032204]	**1.659**
RP5-1077B9.4	Homo sapiens invasion inhibitory protein 45 (IIP45), mRNA [NM_021933]	**1.659**
RGS19	Homo sapiens regulator of G-protein signaling 19 (RGS19), mRNA [NM_005873]	**1.658**
ACTN4	Homo sapiens actinin, alpha 4 (ACTN4), mRNA [NM_004924]	**1.657**
GPR175	Homo sapiens G protein-coupled receptor 175 (GPR175), mRNA [NM_016372]	**1.656**
UNC13D	Homo sapiens mRNA for FLJ00067 protein, partial cds. [AK024474]	**1.656**
HAGHL	Homo sapiens hydroxyacylglutathione hydrolase-like (HAGHL), mRNA [NM_032304]	**1.655**
BMP4	Homo sapiens bone morphogenetic protein 4 (BMP4), mRNA [NM_001202]	**1.654**
MED15	Homo sapiens mediator complex subunit 15 (MED15), mRNA [NM_001003891]	**1.654**
RP3-402G11.5	Homo sapiens selenoprotein O (SELO), mRNA [NM_031454]	**1.653**
RNF40	Homo sapiens ring finger protein 40 (RNF40), mRNA [NM_014771]	**1.653**
TMEM100	Homo sapiens transmembrane protein 100 (TMEM100), mRNA [NM_018286]	**1.653**
CDK10	Homo sapiens cyclin-dependent kinase 10 (CDK10), mRNA [NM_052987]	**1.653**
GTF3C1	Homo sapiens general transcription factor IIIC, polypeptide 1, (GTF3C1), mRNA [NM_001520]	**1.652**
LYPLA2	Homo sapiens lysophospholipase II (LYPLA2), mRNA [NM_007260]	**1.651**
MFSD10	Homo sapiens major facilitator superfamily domain containing 10 (MFSD10), mRNA [NM_001120]	**1.651**
NUDT16L1	Homo sapiens nudix (nucleoside diphosphate linked moiety X)-type motif 16-like 1 (NUDT16L1), mRNA [NM_032349]	**1.650**
CAPZB	Homo sapiens capping protein (actin filament) muscle Z-line, beta (CAPZB), mRNA [NM_004930]	**1.649**
hCG_1641703	Putative uncharacterized protein ENSP00000383883 (HCG1641703) [Source:UniProtKB/TrEMBL;Acc:B5MBZ2] [ENST00000401937]	**1.648**
TEX264	Homo sapiens testis expressed 264 (TEX264), mRNA [NM_015926]	**1.648**
COL4A1	Homo sapiens collagen, type IV, alpha 1 (COL4A1), mRNA [NM_001845]	**1.648**

THBS1	Homo sapiens thrombospondin 1 (THBS1), mRNA [NM_003246]	**1.647**
CAP1	Homo sapiens CAP, adenylate cyclase-associated protein 1 (yeast) (CAP1), mRNA [NM_006367]	**1.646**
CTBP1	Homo sapiens C-terminal binding protein 1 (CTBP1), mRNA [NM_001012614]	**1.645**
ECEL1	Homo sapiens endothelin converting enzyme-like 1 (ECEL1), mRNA [NM_004826]	**1.645**
TRAF4	Homo sapiens TNF receptor-associated factor 4 (TRAF4), mRNA [NM_004295]	**1.645**
ST6GALNAC6	Homo sapiens ST6 (alpha-N-acetyl-neuraminyl-2,3-beta-galactosyl-1,3) -N-acetylgalactosaminide alpha-2,6-sialyltransferase 6 (ST6GALNAC6), mRNA [NM_013443]	**1.644**
TUBB	Homo sapiens tubulin, beta (TUBB), mRNA [NM_178014]	**1.643**
HNRNPL	Homo sapiens heterogeneous nuclear ribonucleoprotein L (HNRNPL),mRNA [NM_001533]	**1.643**
TFAP4	Homo sapiens transcription factor AP-4 (activating enhancer binding protein 4) (TFAP4), mRNA [NM_003223]	**1.642**
STK35	Homo sapiens serine/threonine kinase 35 (STK35), mRNA [NM_080836]	**1.641**
RAD23A	Homo sapiens RAD23 homolog A (S. cerevisiae) (RAD23A), mRNA [NM_005053]	**1.640**
M6PRBP1	Homo sapiens mannose-6-phosphate receptor binding protein 1 (M6PRBP1), mRNA [NM_005817]	**1.639**
NPR1	Homo sapiens natriuretic peptide receptor A/guanylate cyclase A (atrionatriuretic peptide receptor A) (NPR1), mRNA [NM_000906]	**1.639**
SNRPB	Homo sapiens small nuclear ribonucleoprotein polypeptides B and B1 (SNRPB), mRNA [NM_198216]	**1.638**
TBCB	Homo sapiens tubulin folding cofactor B (TBCB), mRNA [NM_001281]	**1.637**
SKIV2L	Homo sapiens superkiller viralicidic activity 2-like (S. cerevisiae) (SKIV2L), mRNA [NM_006929]	**1.633**
PLEKHM2	Homo sapiens pleckstrin homology domain containing, family M (with RUN domain) member 2 (PLEKHM2), mRNA [NM_015164]	**1.632**
RP11-631M21.2	Homo sapiens tubulin, beta 8 (TUBB8), mRNA [NM_177987]	**1.631**
EEF1G	Homo sapiens eukaryotic translation elongation factor 1 gamma (EEF1G), mRNA [NM_001404]	**1.630**
ENG	Homo sapiens endoglin (ENG), transcript variant 2, mRNA [NM_000118]	**1.630**
NUP210	Homo sapiens nucleoporin 210kDa (NUP210), mRNA [NM_024923]	**1.629**
PSMC3	Homo sapiens proteasome (prosome, macropain) 26S subunit, ATPase, 3 (PSMC3), mRNA [NM_002804]	**1.629**
GEMIN5	Homo sapiens gem (nuclear organelle) associated protein 5 (GEMIN5), mRNA [NM_015465]	**1.626**
LOC401717	Homo sapiens misc_RNA (LOC401717), miscRNA [XR_018189]	**1.626**
PCYT2	Homo sapiens phosphate cytidylyltransferase 2, ethanolamine (PCYT2), mRNA [NM_002861]	**1.626**
MAD1L1	Homo sapiens MAD1 mitotic arrest deficient-like 1 (yeast) (MAD1L1), mRNA [NM_003550]	**1.625**
PRMT1	Homo sapiens protein arginine methyltransferase 1 (PRMT1), mRNA [NM_198319]	**1.625**
PSMA7	Homo sapiens proteasome (prosome, macropain) subunit, alpha type, 7 (PSMA7),	**1.625**

	mRNA [NM_002792]	
ACIN1	Homo sapiens apoptotic chromatin condensation inducer 1 (ACIN1), mRNA [NM_014977]	**1.624**
GMPPB	Homo sapiens GDP-mannose pyrophosphorylase B (GMPPB), mRNA [NM_021971]	**1.620**
NOSIP	Homo sapiens nitric oxide synthase interacting protein (NOSIP), mRNA [NM_015953]	**1.620**
C9orf69	Homo sapiens chromosome 9 open reading frame 69 (C9orf69), mRNA [NM_152833]	**1.619**
CC2D1A	Homo sapiens coiled-coil and C2 domain containing 1A (CC2D1A), mRNA [NM_017721]	**1.619**
HSPB1	Homo sapiens heat shock 27kDa protein 1 (HSPB1), mRNA [NM_001540]	**1.619**
PCNXL3	Homo sapiens pecanex-like 3 (Drosophila) (PCNXL3), mRNA [NM_032223]	**1.619**
NBEAL2	Homo sapiens neurobeachin-like 2 (NBEAL2), mRNA [NM_015175]	**1.617**
TTYH3	Homo sapiens tweety homolog 3 (Drosophila) (TTYH3), mRNA [NM_025250]	**1.616**
AGTRAP	Homo sapiens angiotensin II receptor-associated protein (AGTRAP), mRNA [NM_001040196]	**1.614**
SDHA	Homo sapiens succinate dehydrogenase complex, subunit A, flavoprotein (Fp) (SDHA), mRNA [NM_004168]	**1.612**
DDX42	Homo sapiens DEAD (Asp-Glu-Ala-Asp) box polypeptide 42 (DDX42), mRNA [NM_007372]	**1.612**
ARRDC1	Homo sapiens arrestin domain containing 1 (ARRDC1), mRNA [NM_152285]	**1.611**
SETD1A	Homo sapiens SET domain containing 1A (SETD1A), mRNA [NM_014712]	**1.611**
MICALL1	Homo sapiens MICAL-like 1 (MICALL1), mRNA [NM_033386]	**1.609**
GPR108	Homo sapiens G protein-coupled receptor 108 (GPR108), mRNA [NM_020171]	**1.603**
TYMP	Homo sapiens thymidine phosphorylase (TYMP), mRNA [NM_001113756]	**1.603**
TSPAN15	Homo sapiens tetraspanin 15 (TSPAN15), mRNA [NM_012339]	**1.601**
SLC35C2	Homo sapiens solute carrier family 35, member C2 (SLC35C2), mRNA [NM_173179]	**1.601**
NFKBIL1	Homo sapiens nuclear factor of kappa light polypeptide gene enhancer in B-cells inhibitor-like 1 (NFKBIL1), mRNA [NM_005007]	**1.600**
NOG	Homo sapiens noggin (NOG), mRNA [NM_005450]	**1.600**
TFDP1	Homo sapiens transcription factor Dp-1 (TFDP1), mRNA [NM_007111]	**1.600**
GMPPA	Homo sapiens GDP-mannose pyrophosphorylase A (GMPPA), mRNA [NM_013335]	**1.600**
FOXO3	Homo sapiens forkhead box O3 (FOXO3), mRNA [NM_001455]	**1.599**
CCDC22	Homo sapiens coiled-coil domain containing 22 (CCDC22), mRNA [NM_014008]	**1.597**
MYST4	Homo sapiens MYST histone acetyltransferase (monocytic leukemia) 4 (MYST4), mRNA [NM_012330]	**1.596**
C9orf122	Homo sapiens cDNA clone IMAGE:5288595. [BC036230]	**1.594**
COPS7A	Homo sapiens COP9 constitutive photomorphogenic homolog subunit 7A (Arabidopsis) (COPS7A), mRNA [NM_016319]	**1.593**
IFNAR2	Homo sapiens interferon (alpha, beta and omega) receptor 2 (IFNAR2), transcript variant 1, mRNA [NM_207585]	**1.593**
MAP3K12	Homo sapiens mitogen-activated protein kinase kinase kinase 12 (MAP3K12), mRNA [NM_006301]	**1.593**
ZMAT3	Homo sapiens zinc finger, matrin type 3 (ZMAT3), mRNA [NM_022470]	**1.593**
FOXK1	Homo sapiens forkhead box K1 (FOXK1), mRNA [NM_001037165]	**1.593**

PCDHGA7	Homo sapiens protocadherin gamma subfamily A, 7 (PCDHGA7), mRNA [NM_032087]	**1.592**
ELOF1	Homo sapiens elongation factor 1 homolog (S. cerevisiae) (ELOF1), mRNA [NM_032377]	**1.591**
DNPEP	Homo sapiens aspartyl aminopeptidase (DNPEP), mRNA [NM_012100]	**1.591**
BHLHE40	Homo sapiens basic helix-loop-helix domain containing, class B, 2 (BHLHB2), mRNA [NM_003670]	**1.589**
C9orf16	Homo sapiens chromosome 9 open reading frame 16 (C9orf16), mRNA [NM_024112]	**1.589**
MGC16703	Homo sapiens tubulin, alpha pseudogene (MGC16703), non-coding RNA [NR_003608]	**1.589**
PTK7	Homo sapiens PTK7 protein tyrosine kinase 7 (PTK7), mRNA [NM_002821]	**1.589**
NBPF20	Homo sapiens neuroblastoma breakpoint family, member 20 (NBPF20), mRNA [NM_001037675]	**1.588**
NGRN	Homo sapiens neugrin, neurite outgrowth associated (NGRN), mRNA [NM_001033088]	**1.588**
STK25	Homo sapiens serine/threonine kinase 25 (STE20 homolog, yeast) (STK25), mRNA [NM_006374]	**1.588**
TPCN2	Homo sapiens two pore segment channel 2 (TPCN2), mRNA [NM_139075]	**1.588**
ACTR1B	Homo sapiens ARP1 actin-related protein 1 homolog B, centractin beta (yeast) (ACTR1B), mRNA [NM_005735]	**1.587**
GGT8P	Homo sapiens gamma-glutamyltransferase 8 pseudogene (GGT8P), non-coding RNA [NR_003503]	**1.585**
PLXNB1	Homo sapiens plexin B1 (PLXNB1), mRNA [NM_002673]	**1.585**
USP22	Homo sapiens ubiquitin specific peptidase 22 (USP22), mRNA [NM_015276]	**1.585**
PHLDA3	Homo sapiens pleckstrin homology-like domain, family A, member 3 (PHLDA3), mRNA [NM_012396]	**1.583**
HSP90AB3P	Homo sapiens heat shock protein 90Bc (HSP90Bc) mRNA, complete cds. [AY956764]	**1.582**
MIDN	Homo sapiens midnolin (MIDN), mRNA [NM_177401]	**1.582**
SUV39H1	Homo sapiens suppressor of variegation 3-9 homolog 1 (Drosophila) (SUV39H1), mRNA [NM_003173]	**1.582**
IGFBP6	Homo sapiens insulin-like growth factor binding protein 6 (IGFBP6), mRNA [NM_002178]	**1.581**
TXLNA	Homo sapiens taxilin alpha (TXLNA), mRNA [NM_175852]	**1.579**
HNRNPA0	Homo sapiens heterogeneous nuclear ribonucleoprotein A0 (HNRNPA0), mRNA [NM_006805]	**1.577**
ZFPL1	Homo sapiens zinc finger protein-like 1 (ZFPL1), mRNA [NM_006782]	**1.577**
PEX5	Homo sapiens peroxisomal biogenesis factor 5 (PEX5), mRNA [NM_000319]	**1.576**
TAS2R45	Homo sapiens taste receptor, type 2, member 45 (TAS2R45), mRNA [NM_176886]	**1.576**
APLP2	Homo sapiens amyloid beta (A4) precursor-like protein 2 (APLP2), mRNA [NM_001642]	**1.575**
TUT1	Homo sapiens terminal uridylyl transferase 1, U6 snRNA-specific (TUT1), mRNA [NM_022830]	**1.574**
TSSC4	Homo sapiens tumor suppressing subtransferable candidate 4 (TSSC4), mRNA [NM_005706]	**1.571**
PCSK7	Homo sapiens proprotein convertase subtilisin/kexin type 7 (PCSK7), mRNA [NM_004716]	**1.570**

KLHL17	Homo sapiens kelch-like 17 (Drosophila) (KLHL17), mRNA [NM_198317]	**1.570**
LOC148413	Homo sapiens hypothetical LOC148413 (LOC148413), non-coding RNA [NR_015434]	**1.569**
PDIA4	Homo sapiens protein disulfide isomerase family A, member 4 (PDIA4), mRNA [NM_004911]	**1.569**
UBE2M	Homo sapiens ubiquitin-conjugating enzyme E2M (UBC12 homolog, yeast) (UBE2M), mRNA [NM_003969]	**1.568**
FADS3	Homo sapiens fatty acid desaturase 3 (FADS3), mRNA [NM_021727]	**1.567**
CDC34	Homo sapiens cell division cycle 34 homolog (S. cerevisiae) (CDC34), mRNA [NM_004359]	**1.567**
CTRB2	Homo sapiens chymotrypsinogen B2 (CTRB2), mRNA [NM_001025200]	**1.566**
LOC100130193	Homo sapiens cDNA FLJ38783 fis, clone LIVER2001191. [AK096102]	**1.565**
CCM2	Homo sapiens cerebral cavernous malformation 2 (CCM2), mRNA [NM_001029835]	**1.563**
GALE	Homo sapiens UDP-galactose-4-epimerase (GALE), mRNA [NM_000403]	**1.563**
TNPO3	Homo sapiens transportin 3 (TNPO3), mRNA [NM_012470]	**1.563**
WDR13	Human MG21 mRNA, partial cds. [L08237]	**1.560**
SMAD6	Homo sapiens SMAD family member 6 (SMAD6), mRNA [NM_005585]	**1.558**
CYC1	Homo sapiens cytochrome c-1 (CYC1), mRNA [NM_001916]	**1.556**
FAM111B	Homo sapiens family with sequence similarity 111, member B (FAM111B),mRNA [NM_198947]	**1.554**
PER1	Homo sapiens period homolog 1 (Drosophila), mRNA (cDNA clone IMAGE:5215552). [BC028207]	**1.553**
POLD2	Homo sapiens polymerase (DNA directed), delta 2, regulatory subunit (POLD2), mRNA [NM_006230]	**1.552**
TIMM17B	Homo sapiens translocase of inner mitochondrial membrane 17 homolog B (yeast) (TIMM17B), mRNA [NM_005834]	**1.550**
NOP14	Homo sapiens NOP14 nucleolar protein homolog (yeast) (NOP14), mRNA [NM_003703]	**1.549**
FBXW5	Homo sapiens F-box and WD repeat domain containing 5 (FBXW5), mRNA [NM_018998]	**1.548**
UBE2J2	Homo sapiens ubiquitin-conjugating enzyme E2, J2 (UBC6 homolog, yeast) (UBE2J2), t, mRNA [NM_194458]	**1.548**
MAPKAP1	Homo sapiens mitogen-activated protein kinase associated protein 1 (MAPKAP1), mRNA [NM_001006617]	**1.548**
DPP9	Homo sapiens dipeptidyl-peptidase 9 (DPP9), mRNA [NM_139159]	**1.546**
HIRIP3	Homo sapiens HIRA interacting protein 3 (HIRIP3), mRNA [NM_003609]	**1.546**
NUP214	Homo sapiens nucleoporin 214kDa (NUP214), mRNA [NM_005085]	**1.545**
ZNF787	Homo sapiens zinc finger protein 787 (ZNF787), mRNA [NM_001002836]	**1.545**
KIF22	Homo sapiens kinesin family member 22 (KIF22), mRNA [NM_007317]	**1.544**
TTC4	Homo sapiens tetratricopeptide repeat domain 4 (TTC4), mRNA [NM_004623]	**1.542**
HM13	Homo sapiens histocompatibility (minor) 13 (HM13), mRNA [NM_178582]	**1.541**
C16orf5	Homo sapiens chromosome 16 open reading frame 5 (C16orf5), mRNA [NM_013399]	**1.540**
SBF1	Homo sapiens SET binding factor 1 (SBF1), mRNA [NM_002972]	**1.538**
IVD	Homo sapiens isovaleryl Coenzyme A dehydrogenase (IVD), mRNA [NM_002225]	**1.538**

ATP6V0E2	Homo sapiens ATPase, H+ transporting V0 subunit e2 (ATP6V0E2), mRNA [NM_145230]	**1.537**
COG4	Homo sapiens component of oligomeric golgi complex 4 (COG4), mRNA [NM_015386]	**1.537**
AP4M1	Homo sapiens adaptor-related protein complex 4, mu 1 subunit (AP4M1), mRNA [NM_004722]	**1.536**
ARMC6	Homo sapiens armadillo repeat containing 6 (ARMC6), mRNA [NM_033415]	**1.535**
PDLIM2	Homo sapiens PDZ and LIM domain 2 (mystique) (PDLIM2), mRNA [NM_176871]	**1.535**
PPP2R1A	Homo sapiens protein phosphatase 2 (formerly 2A), regulatory subunit A, alpha isoform (PPP2R1A), mRNA [NM_014225]	**1.535**
BMF	Homo sapiens Bcl2 modifying factor (BMF), mRNA [NM_001003940]	**1.534**
SLC25A39	Homo sapiens solute carrier family 25, member 39 (SLC25A39), mRNA [NM_016016]	**1.534**
AMDHD2	Homo sapiens amidohydrolase domain containing 2 (AMDHD2), mRNA [NM_015944]	**1.533**
FTHL17	Homo sapiens ferritin, heavy polypeptide-like 17 (FTHL17), mRNA [NM_031894]	**1.532**
XPC	Homo sapiens xeroderma pigmentosum, complementation group C (XPC), mRNA [NM_004628]	**1.531**
ANKRD9	Homo sapiens ankyrin repeat domain 9 (ANKRD9), mRNA [NM_152326]	**1.530**
FARSA	Homo sapiens phenylalanyl-tRNA synthetase, alpha subunit (FARSA), mRNA [NM_004461]	**1.530**
NXN	Homo sapiens nucleoredoxin (NXN), mRNA [NM_022463]	**1.530**
CUEDC1	Homo sapiens CUE domain containing 1 (CUEDC1), mRNA [NM_017949]	**1.529**
LOC149501	Homo sapiens misc_RNA (LOC149501), miscRNA [XR_018597]	**1.527**
WDR79	Homo sapiens WD repeat domain 79 (WDR79), mRNA [NM_018081]	**1.526**
STIP1	Homo sapiens stress-induced-phosphoprotein 1 (STIP1), mRNA [NM_006819]	**1.524**
P2RX5	Homo sapiens purinergic receptor P2X, ligand-gated ion channel, 5 (P2RX5), mRNA [NM_002561]	**1.522**
PTGES	Homo sapiens prostaglandin E synthase (PTGES), mRNA [NM_004878]	**1.521**
THOC5	Homo sapiens mRNA for KIAA0983 protein, partial cds. [AB023200]	**1.521**
BMP1	Homo sapiens bone morphogenetic protein 1 (BMP1), transcript variant BMP1-2, mRNA [NM_006128]	**1.520**
EIF5A	Homo sapiens eukaryotic translation initiation factor 5A (EIF5A), mRNA [NM_001970]	**1.520**
FAM3A	Homo sapiens family with sequence similarity 3, member A (FAM3A), mRNA [NM_021806]	**1.520**
ASPSCR1	Homo sapiens alveolar soft part sarcoma chromosome region, candidate 1 (ASPSCR1), mRNA [NM_024083]	**1.518**
POR	Homo sapiens P450 (cytochrome) oxidoreductase (POR), mRNA [NM_000941]	**1.518**
CCS	Homo sapiens copper chaperone for superoxide dismutase (CCS), mRNA [NM_005125]	**1.517**
NPTN	Homo sapiens neuroplastin (NPTN), mRNA [NM_012428]	**1.517**
MGC27348	Homo sapiens ribosomal protein S2 pseudogene, mRNA (cDNA clone IMAGE:4671259). [BC026177]	**1.516**
TNFAIP8L3	Homo sapiens tumor necrosis factor, alpha-induced protein 8-like 3 (TNFAIP8L3), mRNA [NM_207381]	**1.516**
FLAD1	Homo sapiens FAD1 flavin adenine dinucleotide synthetase homolog (S. cerevisiae) (FLAD1), mRNA [NM_025207]	**1.516**

FLJ40113	Homo sapiens golgi autoantigen, golgin subfamily a-like pseudogene (FLJ40113), non-coding RNA [NR_003246]	**1.513**
FLJ45422	Homo sapiens FLJ45422 protein (FLJ45422), mRNA [NM_001004349]	**1.511**
FSTL3	Homo sapiens follistatin-like 3 (secreted glycoprotein) (FSTL3), mRNA [NM_005860]	**1.510**
ACLY	Human ATP:citrate lyase mRNA, complete cds. [U18197]	**1.509**
CLCN7	Homo sapiens chloride channel 7 (CLCN7), mRNA [NM_001287]	**1.509**
GALNS	Homo sapiens galactosamine (N-acetyl)-6-sulfate sulfatase (GALNS), mRNA [NM_000512]	**1.509**
KRT79	Homo sapiens keratin 79 (KRT79), mRNA [NM_175834]	**1.509**
LOC401218	Homo sapiens misc_RNA (LOC401218), miscRNA [XR_042344]	**1.508**
DAPK3	Homo sapiens death-associated protein kinase 3 (DAPK3), mRNA [NM_001348]	**1.507**
METT10D	Homo sapiens methyltransferase 10 domain containing (METT10D), mRNA [NM_024086]	**1.506**
PRO0132	Homo sapiens PRO0132 protein (PRO0132), non-coding RNA [NR_002763]	**1.505**
DNMT1	Homo sapiens DNA (cytosine-5-)-methyltransferase 1 (DNMT1), mRNA [NM_001379]	**1.502**
KIAA0406	Homo sapiens KIAA0406 (KIAA0406), mRNA [NM_014657]	**1.501**
C17orf70	Homo sapiens chromosome 17 open reading frame 70 (C17orf70), mRNA [NM_025161]	**1.500**
PRAMEF3	Homo sapiens PRAME family member 3 (PRAMEF3), mRNA [NM_001013692]	**1.500**
TOP1	Homo sapiens topoisomerase (DNA) I (TOP1), mRNA [NM_003286]	**1.500**
KATNB1	Homo sapiens katanin p80 (WD repeat containing) subunit B 1 (KATNB1), mRNA [NM_005886]	**1.499**
PPM2C	Homo sapiens protein phosphatase 2C, magnesium-dependent, catalytic subunit (PPM2C), nuclear gene encoding mitochondrial protein, mRNA [NM_018444]	**1.499**
ENSA	Homo sapiens endosulfine alpha (ENSA), transcript variant 3, mRNA [NM_004436]	**1.498**
HIST1H2BJ	Homo sapiens histone cluster 1, H2bj, mRNA (cDNA clone IMAGE:4048288), [BC014312]	**1.497**
PRPF31	Homo sapiens PRP31 pre-mRNA processing factor 31 homolog (S. cerevisiae) (PRPF31), mRNA [NM_015629]	**1.497**
PIGS	Homo sapiens phosphatidylinositol glycan anchor biosynthesis, class S (PIGS), mRNA [NM_033198]	**1.495**
PFN1	Homo sapiens profilin 1 (PFN1), mRNA [NM_005022]	**1.495**
YKT6	Homo sapiens YKT6 v-SNARE homolog (S. cerevisiae) (YKT6), mRNA [NM_006555]	**1.494**
LOC644063	PREDICTED: Homo sapiens misc_RNA (LOC644063), miscRNA [XR_018217]	**1.491**
KCNN4	Homo sapiens potassium intermediate/small conductance calcium-activated channel, subfamily N, member 4 (KCNN4), mRNA [NM_002250]	**1.490**
RPL37A	Homo sapiens ribosomal protein L37a (RPL37A), mRNA [NM_000998]	**1.490**
STK38	Homo sapiens serine/threonine kinase 38 (STK38), mRNA [NM_007271]	**1.490**
RBM38	Homo sapiens RNA binding motif protein 38 (RBM38), mRNA [NM_017495]	**1.489**
CTDP1	Homo sapiens CTD (carboxy-terminal domain, RNA polymerase II, polypeptide A) phosphatase, subunit 1 (CTDP1), transcript variant FCP1a, mRNA [NM_004715]	**1.488**
CPSF3L	Homo sapiens cleavage and polyadenylation specific factor 3-like (CPSF3L), mRNA [NM_017871]	**1.487**
FLJ10404	Homo sapiens hypothetical protein FLJ10404 (FLJ10404), mRNA [NM_019057]	**1.486**

UNC119	Homo sapiens unc-119 homolog (C. elegans) (UNC119), mRNA [NM_005148]	**1.485**
PIN1L	Homo sapiens peptidylprolyl cis/trans isomerase, NIMA-interacting 1-like (pseudogene) (PIN1L), non-coding RNA [NR_023916]	**1.483**
ALDH3A1	Homo sapiens aldehyde dehydrogenase 3 family, memberA1 (ALDH3A1),mRNA [NM_000691]	**1.482**
COBRA1	Homo sapiens cofactor of BRCA1 (COBRA1), mRNA [NM_015456]	**1.482**
RBM23	Homo sapiens RNA binding motif protein 23 (RBM23), mRNA [NM_001077351]	**1.481**
NOC2L	Homo sapiens nucleolar complex associated 2 homolog (S. cerevisiae) (NOC2L), mRNA [NM_015658]	**1.480**
ARL4D	Homo sapiens ADP-ribosylation factor-like 4D (ARL4D), mRNA [NM_001661]	**1.480**
FUS	Homo sapiens fusion (involved in t(12;16) in malignant liposarcoma) (FUS), mRNA [NM_004960]	**1.477**
SMPD1	Homo sapiens sphingomyelin phosphodiesterase 1, acid lysosomal (SMPD1),mRNA [NM_000543]	**1.477**
CUEDC2	Homo sapiens CUE domain containing 2 (CUEDC2), mRNA [NM_024040]	**1.476**
SRCAP	Homo sapiens mRNA for KIAA0309 gene, [AB002307]	**1.476**
SRP68	Homo sapiens signal recognition particle 68kDa (SRP68), mRNA [NM_014230]	**1.476**
BCL9	Homo sapiens B-cell CLL/lymphoma 9 (BCL9), mRNA [NM_004326]	**1.475**
C9orf114	Homo sapiens chromosome 9 open reading frame 114 (C9orf114), mRNA [NM_016390]	**1.475**
KIAA0495	Homo sapiens KIAA0495 (KIAA0495), mRNA [NM_207306]	**1.474**
NUP188	Homo sapiens nucleoporin 188kDa, mRNA (cDNA clone IMAGE:3461492). [BC005407]	**1.472**
GTF3C5	Homo sapiens general transcription factor IIIC, polypeptide 5, 63kDa (GTF3C5), mRNA [NM_012087]	**1.471**
ZNF263	Homo sapiens zinc finger protein 263 (ZNF263), mRNA [NM_005741]	**1.471**
DUS2L	Homo sapiens dihydrouridine synthase 2-like, SMM1 homolog (DUS2L), mRNA [NM_017803]	**1.469**
METRN	Homo sapiens meteorin, glial cell differentiation regulator (METRN), mRNA [NM_024042]	**1.469**
TMEM134	Homo sapiens transmembrane protein 134 (TMEM134), mRNA [NM_025124]	**1.467**
BRD9	Homo sapiens bromodomain containing 9 (BRD9), mRNA [NM_023924]	**1.465**
CCDC94	Homo sapiens coiled-coil domain containing 94 (CCDC94), mRNA [NM_018074]	**1.465**
UBE2G2	Homo sapiens ubiquitin-conjugating enzyme E2G 2 (UBC7 homolog, yeast) (UBE2G2), mRNA [NM_182688]	**1.465**
SLC7A5	Homo sapiens solute carrier family 7 (cationic amino acid transporter, y+ system), member 5 (SLC7A5), mRNA [NM_003486]	**1.464**
GTF2H4	Homo sapiens general transcription factor IIH, polypeptide 4, 52kDa (GTF2H4), mRNA [NM_001517]	**1.463**
NARFL	Homo sapiens nuclear prelamin A recognition factor-like (NARFL), mRNA [NM_022493]	**1.462**
LOC147804	Homo sapiens tropomyosin 3 pseudogene (LOC147804), non-coding RNA [NR_003148]	**1.461**
LOC728411	Homo sapiens cDNA FLJ58963 complete cds, highly similar to Beta-glucuronidase-like protein SMA3. [AK302866]	**1.461**

NFATC2IP	Homo sapiens nuclear factor of activated T-cells, cytoplasmic, calcineurin-dependent 2 interacting protein (NFATC2IP), mRNA [NM_032815]	**1.461**
PGS1	Homo sapiens phosphatidylglycerophosphate synthase 1 (PGS1), mRNA [NM_024419]	**1.460**
EIF4H	Homo sapiens eukaryotic translation initiation factor 4H (EIF4H), mRNA [NM_022170]	**1.460**
MYL9	Homo sapiens myosin, light chain 9, regulatory (MYL9), mRNA [NM_181526]	**1.458**
PSMD13	Homo sapiens proteasome (prosome, macropain) 26S subunit, non-ATPase, 13 (PSMD13), mRNA [NM_175932]	**1.458**
TNFRSF1A	Homo sapiens tumor necrosis factor receptor superfamily, member 1A (TNFRSF1A), mRNA [NM_001065]	**1.458**
LAMP1	Homo sapiens lysosomal-associated membrane protein 1 (LAMP1), mRNA [NM_005561]	**1.457**
RNPS1	Homo sapiens RNA binding protein S1, serine-rich domain (RNPS1), mRNA [NM_006711]	**1.456**
TNFSF13	Homo sapiens tumor necrosis factor (ligand) superfamily, member 13 (TNFSF13), mRNA [NM_172088]	**1.453**
L1CAM	Homo sapiens L1 cell adhesion molecule (L1CAM), mRNA [NM_024003]	**1.452**
RSPRY1	Homo sapiens cDNA FLJ14643 fis, clone NT2RP2001597, weakly similar to Ryanodine Receptor, Cardiac Muscle. [AK027549]	**1.452**
GGT1	Homo sapiens gamma-glutamyltransferase 1 (GGT1), mRNA [NM_005265]	**1.451**
CXXC5	Homo sapiens CXXC finger 5 (CXXC5), mRNA [NM_016463]	**1.450**
MRPL12	Homo sapiens mitochondrial ribosomal protein L12 (MRPL12), mRNA [NM_002949]	**1.450**
ZDHHC18	Homo sapiens zinc finger, DHHC-type containing 18 (ZDHHC18), mRNA [NM_032283]	**1.450**
TSC22D1	Homo sapiens TSC22 domain family, member 1 (TSC22D1), mRNA [NM_183422]	**1.449**
USF2	Homo sapiens clone TCCCIA00046 mRNA sequence. [AY007087]	**1.449**
CRMP1	Homo sapiens collapsin response mediator protein 1 (CRMP1), mRNA [NM_001014809]	**1.447**
NKAIN4	Homo sapiens Na+/K+ transporting ATPase interacting 4 (NKAIN4), mRNA [NM_152864]	**1.447**
CTF8	Homo sapiens chromosome transmission fidelity factor 8 homolog (CTF8),mRNA [NM_001039690]	**1.446**
PRPF4	Homo sapiens PRP4 pre-mRNA processing factor 4 homolog (yeast) (PRPF4), mRNA [NM_004697]	**1.446**
SCMH1	Homo sapiens sex comb on midleg homolog 1 (Drosophila) (SCMH1),mRNA [NM_012236]	**1.446**
IFRD2	Homo sapiens interferon-related developmental regulator 2 (IFRD2), mRNA [NM_006764]	**1.444**
CREG2	Homo sapiens cellular repressor of E1A-stimulated genes 2 (CREG2), mRNA [NM_153836]	**1.443**
SLC3A2	Homo sapiens solute carrier family 3 (activators of dibasic and neutral amino acid transport), member 2 (SLC3A2), mRNA [NM_001012661]	**1.443**
FAM62A	Homo sapiens family with sequence similarity 62 (C2 domain containing), member A (FAM62A), mRNA [NM_015292]	**1.441**

ZNF687	Homo sapiens zinc finger protein 687 (ZNF687), mRNA [NM_020832]	**1.441**
HJURP	Homo sapiens Holliday junction recognition protein (HJURP), mRNA [NM_018410]	**1.440**
NFIX	Homo sapiens nuclear factor I/X (CCAAT-binding transcription factor) (NFIX), mRNA [NM_002501]	**1.440**
ELK1	Homo sapiens ELK1, member of ETS oncogene family (ELK1), mRNA [NM_005229]	**1.438**
HOXA3	Homo sapiens homeobox A3 (HOXA3), transcript variant 2, mRNA [NM_153631]	**1.438**
EXOSC5	Homo sapiens exosome component 5 (EXOSC5), mRNA [NM_020158]	**1.437**
JAG2	Homo sapiens jagged 2 (JAG2), mRNA [NM_002226]	**1.437**
OXA1L	Homo sapiens oxidase (cytochrome c) assembly 1-like (OXA1L), mRNA [NM_005015]	**1.437**
MYH9	Homo sapiens myosin, heavy chain 9, non-muscle (MYH9), mRNA [NM_002473]	**1.436**
C8orf55	Homo sapiens chromosome 8 open reading frame 55 (C8orf55), mRNA [NM_016647]	**1.434**
ZMAT5	Homo sapiens zinc finger, matrin type 5 (ZMAT5), mRNA [NM_019103]	**1.432**
FKBP9L	Homo sapiens FK506 binding protein 9-like (FKBP9L), non-coding RNA [NR_003949]	**1.431**
ITGA5	Homo sapiens integrin, alpha 5 (fibronectin receptor, alpha polypeptide) (ITGA5), mRNA [NM_002205]	**1.430**
RPS19BP1	Homo sapiens ribosomal protein S19 binding protein 1 (RPS19BP1), mRNA [NM_194326]	**1.429**
SCYL1	Homo sapiens SCY1-like 1 (S. cerevisiae) (SCYL1), mRNA [NM_001048218]	**1.429**
MYBL2	Homo sapiens v-myb myeloblastosis viral oncogene homolog (avian)-like 2 (MYBL2), mRNA [NM_002466]	**1.428**
RPS16	Homo sapiens ribosomal protein S16 (RPS16), mRNA [NM_001020]	**1.428**
ZC3H18	Homo sapiens zinc finger CCCH-type containing 18 (ZC3H18), mRNA [NM_144604]	**1.428**
IPO7	Homo sapiens importin 7 (IPO7), mRNA [NM_006391]	**1.427**
TXNDC11	Homo sapiens thioredoxin domain containing 11 (TXNDC11), mRNA [NM_015914]	**1.425**
MED27	Homo sapiens mediator complex subunit 27 (MED27), mRNA [NM_004269]	**1.425**
CYBASC3	Homo sapiens cytochrome b, ascorbate dependent 3 (CYBASC3), mRNA [NM_153611]	**1.423**
NT5DC2	Homo sapiens 5'-nucleotidase domain containing 2 (NT5DC2), mRNA [NM_022908]	**1.421**
STUB1	Homo sapiens STIP1 homology and U-box containing protein 1 (STUB1), mRNA [NM_005861]	**1.421**
ATXN7L3	Homo sapiens ataxin 7-like 3 (ATXN7L3), mRNA [NM_020218]	**1.420**
RPS19	Homo sapiens ribosomal protein S19 (RPS19), mRNA [NM_001022]	**1.420**
SART3	Homo sapiens squamous cell carcinoma antigen recognized by T cells 3 (SART3), mRNA [NM_014706]	**1.420**
TMEM160	Homo sapiens transmembrane protein 160 (TMEM160), mRNA [NM_017854]	**1.420**
KRAS	Homo sapiens v-Ki-ras2 Kirsten rat sarcoma viral oncogene homolog, mRNA (cDNA clone IMAGE:5301134). [BC029545]	**1.419**
EIF3B	Homo sapiens eukaryotic translation initiation factor 3, subunit B (EIF3B), mRNA [NM_001037283]	**1.416**
JOSD1	Homo sapiens Josephin domain containing 1 (JOSD1), mRNA [NM_014876]	**1.416**
TCTN2	Homo sapiens tectonic family member 2 (TCTN2), mRNA [NM_024809]	**1.413**
GNA11	Homo sapiens GTP-binding protein alpha 11 (GA11) mRNA, partial cds. [L40630]	**1.413**
MXD3	Homo sapiens MAX dimerization protein 3 (MXD3), mRNA [NM_001142935]	**1.412**
UBE2MP1	Homo sapiens ubiquitin-conjugating enzyme E2M pseudogene 1 (UBE2MP1),	**1.411**

	non-coding RNA [NR_002837]	
SMARCC1	Homo sapiens SWI/SNF related, matrix associated, subfamily c, member 1 (SMARCC1), mRNA [NM_003074]	**1.411**
AMPD2	Homo sapiens adenosine monophosphate deaminase 2 (isoform L) (AMPD2), mRNA [NM_004037]	**1.410**
EHMT1	Homo sapiens euchromatic histone-lysine N-methyltransferase 1 (EHMT1), mRNA [NM_024757]	**1.410**
CRIP2	Homo sapiens cysteine-rich protein 2 (CRIP2), mRNA [NM_001312]	**1.409**
HOOK2	Homo sapiens hook homolog 2 (Drosophila) (HOOK2), mRNA [NM_013312]	**1.409**
H2AFY	Homo sapiens H2A histone family, member Y (H2AFY), mRNA [NM_138610]	**1.408**
KIAA1539	Homo sapiens KIAA1539 (KIAA1539), mRNA [NM_025182]	**1.408**
TH1L	Homo sapiens TH1-like (Drosophila) (TH1L), mRNA [NM_198976]	**1.408**
WDR18	Homo sapiens WD repeat domain 18 (WDR18), mRNA [NM_024100]	**1.408**
SLC25A19	Homo sapiens solute carrier family 25 (mitochondrial thiamine pyrophosphate carrier), member 19 (SLC25A19), mRNA [NM_021734]	**1.407**
ACE	Homo sapiens angiotensin I converting enzyme (peptidyl-dipeptidase A) 1 (ACE), mRNA [NM_000789]	**1.406**
PRKD2	Homo sapiens protein kinase D2 (PRKD2), mRNA [NM_016457]	**1.404**
C19orf20	Homo sapiens chromosome 19 open reading frame 20 (C19orf20), mRNA [NM_033513]	**1.403**
GRLF1	Homo sapiens glucocorticoid receptor DNA binding factor 1 (GRLF1), mRNA [NM_004491]	**1.403**
OSBP2	Homo sapiens oxysterol binding protein 2 (OSBP2), mRNA [NM_030758]	**1.403**
SMPD2	Homo sapiens sphingomyelin phosphodiesterase 2, neutral membrane (neutral sphingomyelinase) (SMPD2), mRNA [NM_003080]	**1.402**
IRF3	Homo sapiens interferon regulatory factor 3 (IRF3), mRNA [NM_001571]	**1.401**
TUBA3C	Homo sapiens tubulin, alpha 3c (TUBA3C), mRNA [NM_006001]	**1.401**
CCND3	Homo sapiens cyclin D3 (CCND3), mRNA [NM_001760]	**1.400**
PLP2	Homo sapiens proteolipid protein 2 (colonic epithelium-enriched) (PLP2), mRNA [NM_002668]	**1.399**
YES1	Homo sapiens v-yes-1 Yamaguchi sarcoma viral oncogene homolog 1 (YES1), mRNA [NM_005433]	**1.399**
PKN3	Homo sapiens protein kinase N3 (PKN3), mRNA [NM_013355]	**1.398**
NOTCH1	Homo sapiens Notch homolog 1, translocation-associated (Drosophila) (NOTCH1), mRNA [NM_017617]	**1.396**
ASL	Homo sapiens argininosuccinate lyase (ASL), mRNA [NM_001024943]	**1.395**
FHL3	Homo sapiens four and a half LIM domains 3 (FHL3), mRNA [NM_004468]	**1.394**
CAD	Homo sapiens carbamoyl-phosphate synthetase 2, aspartate transcarbamylase, and dihydroorotase (CAD), mRNA [NM_004341]	**1.393**
CDK2AP2	Homo sapiens cyclin-dependent kinase 2 associated protein 2 (CDK2AP2), mRNA [NM_005851]	**1.392**
DUS3L	Homo sapiens dihydrouridine synthase 3-like (DUS3L), mRNA [NM_020175]	**1.392**
LOC100128760	Homo sapiens misc_RNA (LOC100128760), miscRNA [XR_037264]	**1.392**
BAI2	Homo sapiens brain-specific angiogenesis inhibitor 2 (BAI2), mRNA [NM_001703]	**1.391**

ELOVL1	Homo sapiens elongation of very long chain fatty acids (FEN1/Elo2, SUR4/Elo3, yeast)-like 1 (ELOVL1), mRNA [NM_022821]	**1.391**
KLHDC8B	Homo sapiens kelch domain containing 8B (KLHDC8B), mRNA [NM_173546]	**1.391**
PMPCA	Homo sapiens peptidase (mitochondrial processing) alpha (PMPCA), mRNA [NM_015160]	**1.391**
NCL	Homo sapiens nucleolin (NCL), mRNA [NM_005381]	**1.391**
G6PC3	Homo sapiens glucose 6 phosphatase, catalytic, 3 (G6PC3), mRNA [NM_138387]	**1.390**
PODXL2	Homo sapiens podocalyxin-like 2 (PODXL2), mRNA [NM_015720]	**1.390**
ROGDI	Homo sapiens rogdi homolog (Drosophila) (ROGDI), mRNA [NM_024589]	**1.390**
ARPC1B	Homo sapiens actin related protein 2/3 complex, subunit 1B, 41kDa (ARPC1B), mRNA [NM_005720]	**1.389**
URG4	Homo sapiens up-regulated gene 4 (URG4), mRNA [NM_001077664]	**1.389**
SHARPIN	Homo sapiens SHANK-associated RH domain interactor (SHARPIN), mRNA [NM_030974]	**1.389**
ID3	Homo sapiens inhibitor of DNA binding 3, dominant negative helix-loop-helix protein (ID3), mRNA [NM_002167]	**1.387**
SPHK1	Homo sapiens sphingosine kinase 1 (SPHK1), transcript variant 1, mRNA [NM_021972]	**1.387**
ZNF696	Homo sapiens zinc finger protein 696 (ZNF696), mRNA [NM_030895]	**1.386**
ISOC2	Homo sapiens isochorismatase domain containing 2 (ISOC2), mRNA [NM_024710]	**1.386**
C6orf47	Homo sapiens chromosome 6 open reading frame 47 (C6orf47), mRNA [NM_021184]	**1.385**
DAB2IP	Homo sapiens DAB2 interacting protein (DAB2IP), mRNA [NM_138709]	**1.385**
MSTO1	Homo sapiens misato homolog 1 (Drosophila) (MSTO1), mRNA [NM_018116]	**1.385**
RAB21	Homo sapiens RAB21, member RAS oncogene family (RAB21), mRNA [NM_014999]	**1.385**
ANPEP	Homo sapiens alanyl (membrane) aminopeptidase (ANPEP), mRNA [NM_001150]	**1.384**
GARNL4	Homo sapiens GTPase activating Rap/RanGAP domain-like 4 (GARNL4),mRNA [NM_015085]	**1.384**
ATP13A2	Homo sapiens ATPase type 13A2 (ATP13A2), mRNA [NM_022089]	**1.383**
RRP1	Homo sapiens ribosomal RNA processing 1 homolog (S. cerevisiae) (RRP1), mRNA [NM_003683]	**1.383**
HYAL2	Homo sapiens hyaluronoglucosaminidase 2 (HYAL2), mRNA [NM_003773]	**1.382**
CRAT	Homo sapiens carnitine acetyltransferase (CRAT), mRNA [NM_000755]	**1.380**
PNKP	Homo sapiens polynucleotide kinase 3'-phosphatase (PNKP), mRNA [NM_007254]	**1.380**
EIF4A1	Homo sapiens eukaryotic translation initiation factor 4A, isoform 1 (EIF4A1), mRNA [NM_001416]	**1.380**
CDC20	Homo sapiens cell division cycle 20 homolog (CDC20), mRNA [NM_001255]	**1.379**
C9orf75	Homo sapiens chromosome 9 open reading frame 75 (C9orf75), mRNA [NM_173691]	**1.376**
GRHPR	Homo sapiens glyoxylate reductase/hydroxypyruvate reductase (GRHPR), mRNA [NM_012203]	**1.376**
LSM4	Homo sapiens LSM4 homolog, U6 small nuclear RNA associated (LSM4), mRNA [NM_012321]	**1.376**
ANKZF1	Homo sapiens ankyrin repeat and zinc finger domain containing 1 (ANKZF1), mRNA [NM_018089]	**1.374**
CLCN2	Homo sapiens chloride channel 2 (CLCN2), mRNA [NM_004366]	**1.374**

SAMD11	Homo sapiens sterile alpha motif domain containing 11 (SAMD11), mRNA [NM_152486]	**1.374**
CORO1B	Homo sapiens coronin, actin binding protein, 1B (CORO1B), mRNA [NM_020441]	**1.372**
JMJD8	Homo sapiens jumonji domain containing 8 (JMJD8), mRNA [NM_001005920]	**1.372**
TRIM47	Homo sapiens tripartite motif-containing 47 (TRIM47), mRNA [NM_033452]	**1.372**
C6orf108	Homo sapiens chromosome 6 open reading frame 108 (C6orf108), mRNA [NM_006443]	**1.370**
GDPD3	Homo sapiens glycerophosphodiester phosphodiesterase domain containing 3 (GDPD3), mRNA [NM_024307]	**1.370**
TRIM41	Homo sapiens tripartite motif-containing 41 (TRIM41), mRNA [NM_033549]	**1.370**
UBL4A	Homo sapiens ubiquitin-like 4A (UBL4A), mRNA [NM_014235]	**1.370**
SHMT2	Homo sapiens serine hydroxymethyltransferase 2 (mitochondrial) (SHMT2), mRNA [NM_005412]	**1.369**
RPS15	Homo sapiens ribosomal protein S15 (RPS15), mRNA [NM_001018]	**1.368**
SYNGR3	Homo sapiens synaptogyrin 3 (SYNGR3), mRNA [NM_004209]	**1.368**
NSFL1C	Homo sapiens NSFL1 (p97) cofactor (p47) (NSFL1C), mRNA [NM_016143]	**1.367**
KLHL22	Homo sapiens kelch-like 22 (Drosophila) (KLHL22), mRNA [NM_032775]	**1.366**
UGCG	Homo sapiens UDP-glucose ceramide glucosyltransferase (UGCG), mRNA [NM_003358]	**1.366**
SPG7	Homo sapiens spastic paraplegia 7 (pure and complicated autosomal recessive) (SPG7), mRNA [NM_003119]	**1.365**
CXXC1	Homo sapiens CXXC finger 1 (PHD domain) (CXXC1), mRNA [NM_014593]	**1.365**
MPG	Homo sapiens N-methylpurine-DNA glycosylase (MPG), mRNA [NM_002434]	**1.365**
RAC3	Homo sapiens ras-related C3 botulinum toxin substrate 3 (rho family, small GTP binding protein Rac3) (RAC3), mRNA [NM_005052]	**1.365**
FAM115A	Homo sapiens family with sequence similarity 115, member A (FAM115A), mRNA [NM_014719]	**1.365**
SNRNP70	Homo sapiens small nuclear ribonucleoprotein 70kDa (U1) (SNRNP70), mRNA [NM_003089]	**1.364**
BIN3	Homo sapiens bridging integrator 3 (BIN3), mRNA [NM_018688]	**1.362**
FADD	Homo sapiens Fas (TNFRSF6)-associated via death domain (FADD), mRNA [NM_003824]	**1.362**
FAM176B	Homo sapiens family with sequence similarity 176, member B (FAM176B), mRNA [NM_018166]	**1.362**
LMNA	Homo sapiens lamin A/C (LMNA), transcript variant 2, mRNA [NM_005572]	**1.362**
PICALM	Homo sapiens phosphatidylinositol binding clathrin assembly protein (PICALM), mRNA [NM_007166]	**1.362**
ITPR3	Homo sapiens inositol 1,4,5-triphosphate receptor, type 3 (ITPR3), mRNA [NM_002224]	**1.361**
TMEM63B	Homo sapiens transmembrane protein 63B (TMEM63B), mRNA [NM_018426]	**1.361**
C4orf8	Homo sapiens chromosome 4 open reading frame 8 (C4orf8), mRNA [NM_003704]	**1.361**
TMUB2	Homo sapiens transmembrane and ubiquitin-like domain containing 2 (TMUB2), mRNA [NM_177441]	**1.360**
ABCC1	Homo sapiens ATP-binding cassette, sub-family C (CFTR/MRP), member 1 (ABCC1), mRNA [NM_019862]	**1.360**

BAD	Homo sapiens BCL2-associated agonist of cell death (BAD), mRNA [NM_004322]	**1.359**
COPE	Homo sapiens coatomer protein complex, subunit epsilon (COPE), mRNA [NM_199444]	**1.358**
NDST1	Homo sapiens N-deacetylase/N-sulfotransferase (heparan glucosaminyl) 1, mRNA (cDNA clone MGC:9410 IMAGE:3882074), [BC012888]	**1.358**
BCL7C	Homo sapiens B-cell CLL/lymphoma 7C (BCL7C), mRNA [NM_004765]	**1.357**
VPS11	Homo sapiens vacuolar protein sorting 11 homolog (S. cerevisiae) (VPS11), mRNA [NM_021729]	**1.356**
POLR1A	Homo sapiens polymerase (RNA) I polypeptide A, (POLR1A), mRNA [NM_015425]	**1.355**
THOC4	Homo sapiens THO complex 4 (THOC4), mRNA [NM_005782]	**1.354**
COL5A1	Homo sapiens collagen, type V, alpha 1 (COL5A1), mRNA [NM_000093]	**1.353**
KAT5	Homo sapiens K(lysine) acetyltransferase 5 (KAT5), mRNA [NM_006388]	**1.352**
LOC442211	Homo sapiens misc_RNA (LOC442211), miscRNA [XR_019545]	**1.352**
NAPA	Homo sapiens N-ethylmaleimide-sensitive factor attachment protein, alpha (NAPA), mRNA [NM_003827]	**1.352**
VASP	Homo sapiens vasodilator-stimulated phosphoprotein (VASP), mRNA [NM_003370]	**1.352**
COASY	Homo sapiens Coenzyme A synthase (COASY), mRNA [NM_025233]	**1.351**
WRNIP1	Homo sapiens Werner helicase interacting protein 1 (WRNIP1), t mRNA [NM_130395]	**1.349**
JMJD4	Homo sapiens jumonji domain containing 4 (JMJD4), mRNA [NM_023007]	**1.348**
LASS5	Homo sapiens LAG1 homolog, ceramide synthase 5 (LASS5), mRNA [NM_147190]	**1.348**
TBC1D22A	Homo sapiens TBC1 domain family, member 22A (TBC1D22A), mRNA [NM_014346]	**1.348**
DHX30	Homo sapiens cDNA FLJ11214 fis, clone PLACE1007990. [AK002076]	**1.347**
POLRMT	Homo sapiens polymerase (RNA) mitochondrial (DNA directed) (POLRMT), mRNA [NM_005035]	**1.347**
GABARAPL1	Homo sapiens GABA(A) receptor-associated protein like 1 (GABARAPL1), mRNA [NM_031412]	**1.346**
FAM38A	Homo sapiens family with sequence similarity 38, member A (FAM38A), mRNA [NM_001142864]	**1.345**
NBPF11	Neuroblastoma breakpoint family member 8 [Source:UniProtKB/ Swiss-Prot; Acc:Q3BBV2] [ENST00000339388]	**1.345**
SRM	Homo sapiens spermidine synthase (SRM), mRNA [NM_003132]	**1.345**
TRAF2	Homo sapiens TNF receptor-associated factor 2 (TRAF2), mRNA [NM_021138]	**1.345**
CIDEC	Homo sapiens cell death-inducing DFFA-like effector c (CIDEC), mRNA [NM_022094]	**1.343**
GPATCH3	Homo sapiens G patch domain containing 3 (GPATCH3), mRNA [NM_022078]	**1.343**
YIF1B	Homo sapiens Yip1 interacting factor homolog B (S. cerevisiae) (YIF1B), mRNA [NM_033557]	**1.343**
PBXIP1	Homo sapiens pre-B-cell leukemia homeobox interacting protein 1 (PBXIP1), mRNA [NM_020524]	**1.342**
GGTLC2	Homo sapiens gamma-glutamyltransferase light chain 2 (GGTLC2), mRNA [NM_199127]	**1.342**
MPRIP	Homo sapiens myosin phosphatase Rho interacting protein (MPRIP), mRNA [NM_015134]	**1.342**
STX10	Full-length cDNA clone CS0DI053YA21 of Placenta Cot 25-normalized of Homo sapiens (human). [CR601641]	**1.342**

N-PAC	Homo sapiens cytokine-like nuclear factor n-pac (N-PAC), mRNA [NM_032569]	**1.341**
ZMYND19	Homo sapiens zinc finger, MYND-type containing 19 (ZMYND19), mRNA [NM_138462]	**1.341**
GGT3P	Homo sapiens gamma-glutamyltransferase 3 pseudogene (GGT3P), non-coding RNA [NR_003267]	**1.340**
GOLGA2L1	Homo sapiens golgi autoantigen, golgin subfamily a, 2-like 1 (GOLGA2L1), mRNA [NM_017600]	**1.340**
RHOD	Homo sapiens ras homolog gene family, member D (RHOD), mRNA [NM_014578]	**1.339**
ZFAND3	Homo sapiens zinc finger, AN1-type domain 3 (ZFAND3), mRNA [NM_021943]	**1.339**
TMEM102	Homo sapiens transmembrane protein 102 (TMEM102), mRNA [NM_178518]	**1.339**
SOS1	Homo sapiens son of sevenless homolog 1 (Drosophila) (SOS1), mRNA [NM_005633]	**1.338**
CDC2L5	Homo sapiens cell division cycle 2-like 5 (cholinesterase-related cell division controller) (CDC2L5), mRNA [NM_031267]	**1.337**
LOC402026	Homo sapiens misc_RNA (LOC402026), miscRNA [XR_019171]	**1.335**
CTCF	Homo sapiens CCCTC-binding factor (zinc finger protein) (CTCF), mRNA [NM_006565]	**1.335**
PRELID1	Homo sapiens PRELI domain containing 1 (PRELID1), mRNA [NM_013237]	**1.334**
BARX1	Homo sapiens BARX homeobox 1 (BARX1), mRNA [NM_021570]	**1.333**
IFI35	Homo sapiens interferon-induced protein 35 (IFI35), mRNA [NM_005533]	**1.333**
IQSEC1	Homo sapiens IQ motif and Sec7 domain 1 (IQSEC1), mRNA [NM_014869]	**1.333**
SUOX	Homo sapiens sulfite oxidase (SUOX), mRNA [NM_000456]	**1.333**
TGFBRAP1	Homo sapiens transforming growth factor, beta receptor associated protein 1 (TGFBRAP1), mRNA [NM_004257]	**1.332**
ARHGEF1	Homo sapiens Rho guanine nucleotide exchange factor (GEF) 1 (ARHGEF1), mRNA [NM_199002]	**1.332**
PANK4	Homo sapiens pantothenate kinase 4 (PANK4), mRNA [NM_018216]	**1.331**
LOC644214	Homo sapiens misc_RNA (LOC644214), miscRNA [XR_018965]	**1.330**
TRA2A	Homo sapiens hAWMS1 mRNA, complete cds. [AB052759]	**1.330**
PRKAR2A	Homo sapiens protein kinase, cAMP-dependent, regulatory, type II, alpha, mRNA (cDNA clone MGC:3606 IMAGE:3629579), [BC002763]	**1.330**
TSC22D4	Homo sapiens TSC22 domain family, member 4 (TSC22D4), mRNA [NM_030935]	**1.329**
LGALS3BP	Homo sapiens lectin, galactoside-binding, soluble, 3 binding protein (LGALS3BP), mRNA [NM_005567]	**1.329**
LGALS7B	Homo sapiens lectin, galactoside-binding, soluble, 7B (LGALS7B), mRNA [NM_001042507]	**1.329**
PPAP2C	Homo sapiens phosphatidic acid phosphatase type 2C (PPAP2C), mRNA [NM_177543]	**1.329**
PRKAG1	Homo sapiens protein kinase, AMP-activated, gamma 1 non-catalytic subunit (PRKAG1), mRNA [NM_212461]	**1.328**
TMSB10	Homo sapiens thymosin beta 10 (TMSB10), mRNA [NM_021103]	**1.328**
C1orf43	Homo sapiens chromosome 1 open reading frame 43 (C1orf43), mRNA [NM_138740]	**1.328**
SEMA4B	Homo sapiens sema domain, immunoglobulin domain, transmembrane domain and short cytoplasmic domain, (semaphorin) 4B (SEMA4B), mRNA [NM_020210]	**1.327**
TSPAN4	Homo sapiens tetraspanin 4 (TSPAN4), mRNA [NM_001025237]	**1.327**

DULLARD	Homo sapiens dullard homolog (Xenopus laevis) (DULLARD), mRNA [NM_015343]	**1.326**
LOC644456	Homo sapiens misc_RNA (LOC644456), miscRNA [XR_039438]	**1.326**
LOC645251	Homo sapiens misc_RNA (LOC645251), miscRNA [XR_037112]	**1.323**
CHMP7	Homo sapiens CHMP family, member 7 (CHMP7), mRNA [NM_152272]	**1.323**
RFNG	Homo sapiens RFNG O-fucosylpeptide 3-beta-N-acetylglucosaminyltransferase (RFNG), mRNA [NM_002917]	**1.322**
PIN1	Homo sapiens peptidylprolyl cis/trans isomerase, NIMA-interacting 1 (PIN1), mRNA [NM_006221]	**1.321**
SERINC2	Homo sapiens serine incorporator 2 (SERINC2), mRNA [NM_178865]	**1.320**
GBAP	Homo sapiens glucosidase, beta; acid, pseudogene (GBAP), non-coding RNA [NR_002188]	**1.320**
KPTN	Homo sapiens kaptin (actin binding protein) (KPTN), mRNA [NM_007059]	**1.320**
CCNF	Homo sapiens cyclin F (CCNF), mRNA [NM_001761]	**1.320**
DUS1L	Homo sapiens dihydrouridine synthase 1-like (S. cerevisiae) (DUS1L), mRNA [NM_022156]	**1.319**
JPH2	Homo sapiens junctophilin 2 (JPH2), mRNA [NM_020433]	**1.319**
IPO4	Homo sapiens importin 4 (IPO4), mRNA [NM_024658]	**1.318**
DDX56	Homo sapiens cDNA FLJ43607 fis, clone SPLEN2010912, highly similar to Homo sapiens nucleolar RNA helicase (NOH61). [AK125595]	**1.317**
AARS	Homo sapiens alanyl-tRNA synthetase (AARS), mRNA [NM_001605]	**1.317**
TMBIM1	Homo sapiens transmembrane BAX inhibitor motif containing 1 (TMBIM1), mRNA [NM_022152]	**1.317**
FBXW9	Homo sapiens F-box and WD repeat domain containing 9 (FBXW9), mRNA [NM_032301]	**1.313**
FAM109B	Homo sapiens family with sequence similarity 109, member B (FAM109B), mRNA [NM_001002034]	**1.311**
ATRIP	Homo sapiens ATR interacting protein (ATRIP), mRNA [NM_032166]	**1.311**
SMARCA4	Homo sapiens SWI/SNF related, matrix associated, subfamily a, member 4 (SMARCA4), mRNA [NM_003072]	**1.309**
ARHGEF2	Homo sapiens rho/rac guanine nucleotide exchange factor (GEF) 2 (ARHGEF2), mRNA [NM_004723]	**1.309**
NMRAL1	Homo sapiens NmrA-like family domain containing 1 (NMRAL1), mRNA [NM_020677]	**1.309**
GLE1	Homo sapiens GLE1 RNA export mediator homolog (yeast) (GLE1), mRNA [NM_001499]	**1.308**
PHGDH	Homo sapiens phosphoglycerate dehydrogenase (PHGDH), mRNA [NM_006623]	**1.308**
CLPTM1	Homo sapiens cleft lip and palate associated transmembrane protein 1 (CLPTM1), mRNA [NM_001294]	**1.307**
MOSC1	Homo sapiens MOCO sulphurase C-terminal domain containing 1 (MOSC1), mRNA [NM_022746]	**1.306**
SLC25A11	Homo sapiens solute carrier family 25 (mitochondrial carrier; oxoglutarate carrier), member 11 (SLC25A11), mRNA [NM_003562]	**1.306**
CDC25B	Homo sapiens cell division cycle 25 homolog B (S. pombe) (CDC25B), mRNA	**1.305**

	[NM_021873]	
PDXP	Homo sapiens pyridoxal (pyridoxine, vitamin B6) phosphatase (PDXP), mRNA [NM_020315]	**1.305**
SLC2A6	Homo sapiens solute carrier family 2 (facilitated glucose transporter), member 6 (SLC2A6), mRNA [NM_017585]	**1.305**
SIK1	Homo sapiens salt-inducible kinase 1 (SIK1), mRNA [NM_173354]	**1.304**
C12orf44	Homo sapiens chromosome 12 open reading frame 44 (C12orf44), mRNA [NM_021934]	**1.304**
GNG8	Homo sapiens guanine nucleotide binding protein (G protein), gamma 8 (GNG8), mRNA [NM_033258]	**1.304**
FAM168B	Homo sapiens family with sequence similarity 168, member B (FAM168B), mRNA [NM_001009993]	**1.303**
FTSJ2	Homo sapiens FtsJ homolog 2 (E. coli) (FTSJ2), mRNA [NM_013393]	**1.303**
UBB	Homo sapiens ubiquitin B (UBB), mRNA [NM_018955]	**1.302**
CCDC124	Homo sapiens coiled-coil domain containing 124 (CCDC124), mRNA [NM_138442]	**1.301**
GCDH	Homo sapiens glutaryl-Coenzyme A dehydrogenase (GCDH), mRNA [NM_013976]	**1.301**
RBM3	Homo sapiens RNA binding motif (RNP1, RRM) protein 3 (RBM3), mRNA [NM_006743]	**1.301**
NAT14	Homo sapiens N-acetyltransferase 14 (GCN5-related, putative) (NAT14), mRNA [NM_020378]	**1.300**
BAZ2A	Homo sapiens bromodomain adjacent to zinc finger domain, 2A (BAZ2A), mRNA [NM_013449]	**1.299**
CLU	Homo sapiens clusterin (CLU), mRNA [NM_203339]	**1.299**
TMED3	Homo sapiens transmembrane emp24 protein transport domain containing 3 (TMED3), mRNA [NM_007364]	**1.299**
FAM73B	Homo sapiens family with sequence similarity 73, member B (FAM73B), mRNA [NM_032809]	**1.298**
NT5C3L	Homo sapiens 5'-nucleotidase, cytosolic III-like (NT5C3L), mRNA [NM_052935]	**1.298**
TINAGL1	Homo sapiens tubulointerstitial nephritis antigen-like 1 (TINAGL1), mRNA [NM_022164]	**1.297**
C1orf159	Homo sapiens chromosome 1 open reading frame 159 (C1orf159), mRNA [NM_017891]	**1.297**
CD81	Homo sapiens CD81 molecule (CD81), mRNA [NM_004356]	**1.297**
GLTSCR2	Homo sapiens glioma tumor suppressor candidate region gene 2 (GLTSCR2), mRNA [NM_015710]	**1.297**
LMNB1	Homo sapiens lamin B1 (LMNB1), mRNA [NM_005573]	**1.296**
DLST	Homo sapiens dihydrolipoamide S-succinyltransferase (E2 component of 2-oxo-glutarate complex) (DLST), mRNA [NM_001933]	**1.296**
HLA-E	Homo sapiens major histocompatibility complex, class I, E (HLA-E), mRNA [NM_005516]	**1.295**
SEMA3F	Homo sapiens sema domain, immunoglobulin domain (Ig), (semaphorin) 3F (SEMA3F), mRNA [NM_004186]	**1.295**
TSC22D3	Homo sapiens TSC22 domain family, member 3 (TSC22D3), mRNA [NM_004089]	**1.295**
FBXL6	Homo sapiens F-box and leucine-rich repeat protein 6 (FBXL6), mRNA [NM_012162]	**1.294**
SAMD1	Homo sapiens sterile alpha motif domain containing 1 (SAMD1), mRNA [NM_138352]	**1.294**

HLX	Homo sapiens H2.0-like homeobox (HLX), mRNA [NM_021958]	**1.293**
MCM5	Homo sapiens minichromosome maintenance complex component 5 (MCM5), mRNA [NM_006739]	**1.291**
NBPF14	Homo sapiens neuroblastoma breakpoint family, member 14 (NBPF14), mRNA [NM_015383]	**1.291**
REEP4	Homo sapiens receptor accessory protein 4 (REEP4), mRNA [NM_025232]	**1.291**
TUFM	Homo sapiens Tu translation elongation factor, mitochondrial (TUFM), mRNA [NM_003321]	**1.291**
RUNDC2C	Homo sapiens RUN domain containing 2C (RUNDC2C), non-coding RNA [NR_002939]	**1.290**
FTSJ1	Homo sapiens FtsJ homolog 1 (E. coli) (FTSJ1), mRNA [NM_177439]	**1.289**
DUSP9	Homo sapiens dual specificity phosphatase 9 (DUSP9), mRNA [NM_001395]	**1.288**
MLLT1	Homo sapiens myeloid/lymphoid or mixed-lineage leukemia (trithorax homolog, Drosophila); translocated to, 1 (MLLT1), mRNA [NM_005934]	**1.288**
SLC25A1	Homo sapiens solute carrier family 25 , member 1 (SLC25A1), mRNA [NM_005984]	**1.288**
LOC401010	Homo sapiens nucleolar complex associated 2 homolog pseudogene (LOC401010), non-coding RNA [NR_002826]	**1.286**
MFHAS1	Homo sapiens malignant fibrous histiocytoma amplified sequence 1 (MFHAS1), mRNA [NM_004225]	**1.285**
MAGED2	Homo sapiens melanoma antigen family D, 2 (MAGED2), \mRNA [NM_201222]	**1.284**
PIH1D1	Homo sapiens PIH1 domain containing 1 (PIH1D1), mRNA [NM_017916]	**1.284**
THOC6	Homo sapiens THO complex 6 homolog (Drosophila) (THOC6), mRNA [NM_024339]	**1.283**
TLE3	Homo sapiens transducin-like enhancer of split 3 (E(sp1) homolog, Drosophila) (TLE3), mRNA [NM_005078]	**1.282**
MPDU1	Homo sapiens mannose-P-dolichol utilization defect 1 (MPDU1), mRNA [NM_004870]	**1.281**
SLC6A10P	Homo sapiens solute carrier family 6 (neurotransmitter transporter, creatine), member 10 (pseudogene) (SLC6A10P) on chromosome 16 [NR_003083]	**1.281**
AXIN1	Homo sapiens axin 1 (AXIN1), mRNA [NM_003502]	**1.280**
LOC646808	Homo sapiens misc_RNA (LOC646808), miscRNA [XR_017339]	**1.280**
TNC	Homo sapiens tenascin C (TNC), mRNA [NM_002160]	**1.279**
WNT5B	Homo sapiens wingless-type MMTV integration site family, member 5B (WNT5B), transcript variant 2, mRNA [NM_030775]	**1.279**
PLCD3	Homo sapiens phospholipase C, delta 3 (PLCD3), mRNA [NM_133373]	**1.278**
RFC2	Homo sapiens replication factor C (activator 1) 2, 40kDa (RFC2), mRNA [NM_181471]	**1.278**
ACAP3	Homo sapiens ArfGAP with coiled-coil, ankyrin repeat and PH domains 3 (ACAP3), mRNA [NM_030649]	**1.278**
AP2M1	Homo sapiens adaptor-related protein complex 2, mu 1 subunit (AP2M1),mRNA [NM_004068]	**1.277**
C9orf150	Homo sapiens chromosome 9 open reading frame 150 (C9orf150), mRNA [NM_203403]	**1.277**
CDKN2B	Homo sapiens cyclin-dependent kinase inhibitor 2B (p15, inhibits CDK4) (CDKN2B), mRNA [NM_078487]	**1.277**
AKT1S1	Homo sapiens AKT1 substrate 1 (proline-rich) (AKT1S1), mRNA [NM_032375]	**1.275**
COPG	Homo sapiens coatomer protein complex, subunit gamma (COPG), mRNA	**1.275**

	[NM_016128]	
DVL1	Homo sapiens dishevelled, dsh homolog 1 (Drosophila) (DVL1), mRNA [NM_004421]	**1.275**
ISY1	Pre-mRNA-splicing factor ISY1 homolog [Source:UniProtKB/Swiss-Prot;Acc:Q9ULR0] [ENST00000393295]	**1.275**
NFKBIB	Homo sapiens nuclear factor of kappa light polypeptide gene enhancer in B-cells inhibitor, beta (NFKBIB), mRNA [NM_002503]	**1.274**
C21orf56	Homo sapiens chromosome 21 open reading frame 56 (C21orf56), mRNA [NM_032261]	**1.273**
LDLRAD3	Homo sapiens low density lipoprotein receptor class A domain containing 3 (LDLRAD3), mRNA [NM_174902]	**1.273**
CPA4	Homo sapiens carboxypeptidase A4 (CPA4), mRNA [NM_016352]	**1.272**
NAGK	Homo sapiens N-acetylglucosamine kinase (NAGK), mRNA [NM_017567]	**1.272**
WASF2	Homo sapiens WAS protein family, member 2 (WASF2), mRNA [NM_006990]	**1.272**
MADD	Homo sapiens MAP-kinase activating death domain (MADD), mRNA [NM_003682]	**1.271**
CHMP6	Homo sapiens chromatin modifying protein 6 (CHMP6), mRNA [NM_024591]	**1.270**
MARCKSL1	Homo sapiens MARCKS-like 1 (MARCKSL1), mRNA [NM_023009]	**1.270**
QTRT1	Homo sapiens queuine tRNA-ribosyltransferase 1 (QTRT1), mRNA [NM_031209]	**1.270**
LOC341230	Homo sapiens misc_RNA (LOC341230), miscRNA [XR_018617]	**1.269**
PMS2CL	Homo sapiens PMS2 C-terminal like pseudogene, mRNA (cDNA clone IMAGE:5273238). [BC041364]	**1.268**
SQSTM1	Human phosphotyrosine independent ligand p62B B-cell isoform for the Lck SH2 domain mRNA, partial cds. [U46752]	**1.268**
C1orf128	Homo sapiens chromosome 1 open reading frame 128 (C1orf128), mRNA [NM_020362]	**1.267**
CD2BP2	Homo sapiens CD2 (cytoplasmic tail) binding protein 2 (CD2BP2), mRNA [NM_006110]	**1.267**
RNF25	Homo sapiens ring finger protein 25 (RNF25), mRNA [NM_022453]	**1.267**
AP1M1	Homo sapiens adaptor-related protein complex 1, mu 1 subunit (AP1M1), mRNA [NM_032493]	**1.266**
MBOAT7	Homo sapiens membrane bound O-acyltransferase domain containing 7 (MBOAT7), mRNA [NM_024298]	**1.266**
SHKBP1	Homo sapiens SH3KBP1 binding protein 1 (SHKBP1), mRNA [NM_138392]	**1.266**
RND2	Homo sapiens Rho family GTPase 2 (RND2), mRNA [NM_005440]	**1.265**
STAT5A	Homo sapiens signal transducer and activator of transcription 5A (STAT5A), mRNA [NM_003152]	**1.265**
TPD52L2	Homo sapiens tumor protein D52-like 2 (TPD52L2), mRNA [NM_199360]	**1.265**
PLXNB2	Homo sapiens plexin B2 (PLXNB2), mRNA [NM_012401]	**1.265**
BCL2L1	Homo sapiens BCL2-like 1 (BCL2L1), mRNA [NM_138578]	**1.264**
MAP4K2	Homo sapiens mitogen-activated protein kinase kinase kinase kinase 2 (MAP4K2), mRNA [NM_004579]	**1.262**
ARF5	Homo sapiens ADP-ribosylation factor 5 (ARF5), mRNA [NM_001662]	**1.262**
C9orf64	Homo sapiens chromosome 9 open reading frame 64 (C9orf64), mRNA [NM_032307]	**1.261**
EPB41L2	Homo sapiens erythrocyte membrane protein band 4.1-like 2 (EPB41L2), mRNA [NM_001431]	**1.261**
CPOX	Homo sapiens coproporphyrinogen oxidase (CPOX), mRNA [NM_000097]	**1.260**

DDX3Y	Homo sapiens DEAD (Asp-Glu-Ala-Asp) box polypeptide 3, Y-linked (DDX3Y), mRNA [NM_004660]	**1.260**
PLXND1	Homo sapiens plexin D1 (PLXND1), mRNA [NM_015103]	**1.260**
FLOT1	Homo sapiens flotillin 1 (FLOT1), mRNA [NM_005803]	**1.259**
STOML2	Homo sapiens stomatin (EPB72)-like 2 (STOML2), mRNA [NM_013442]	**1.259**
USP41	Putative ubiquitin carboxyl-terminal hydrolase 41 (EC 3.1.2.15)(Ubiquitin thioesterase 41) [Source:UniProtKB/Swiss-Prot;Acc:Q3LFD5] [ENST00000292729]	**1.259**
KIAA1618	Homo sapiens mRNA for KIAA1618 protein, partial cds. [AB046838]	**1.258**
LYPLA2P1	Homo sapiens lysophospholipase II pseudogene 1 (LYPLA2P1), non-coding RNA [NR_001444]	**1.258**
ADCY6	Homo sapiens adenylate cyclase 6 (ADCY6), mRNA [NM_015270]	**1.257**
FLJ39582	full-length cDNA clone CS0DI082YF06 of Placenta Cot 25-normalized of Homo sapiens (human). [CR600536]	**1.257**
PCOLCE	Homo sapiens procollagen C-endopeptidase enhancer (PCOLCE), mRNA [NM_002593]	**1.257**
KIAA0652	Homo sapiens KIAA0652 (KIAA0652), mRNA [NM_014741]	**1.257**
DAXX	Homo sapiens death-domain associated protein (DAXX), mRNA [NM_001350]	**1.256**
CD320	Homo sapiens CD320 molecule (CD320), mRNA [NM_016579]	**1.254**
FOXO4	Homo sapiens forkhead box O4 (FOXO4), mRNA [NM_005938]	**1.253**
SH3GLB2	Homo sapiens SH3-domain GRB2-like endophilin B2 (SH3GLB2), mRNA [NM_020145]	**1.253**
COIL	Homo sapiens coilin (COIL), mRNA [NM_004645]	**1.252**
COX1	Cytochrome c oxidase subunit 1 (EC 1.9.3.1)(Cytochrome c oxidase polypeptide I) [Source:UniProtKB/Swiss-Prot;Acc:P00395] [ENST00000361624]	**1.252**
DGAT2	Homo sapiens diacylglycerol O-acyltransferase homolog 2 (mouse) (DGAT2), mRNA [NM_032564]	**1.252**
OGDH	Homo sapiens oxoglutarate (alpha-ketoglutarate) dehydrogenase (lipoamide) (OGDH), mRNA [NM_001003941]	**1.251**
CYB5B	Homo sapiens cytochrome b5 type B (outer mitochondrial membrane) (CYB5B), mRNA [NM_030579]	**1.251**
GMEB2	Homo sapiens glucocorticoid modulatory element binding protein 2 (GMEB2), mRNA [NM_012384]	**1.251**
RUSC1	Homo sapiens RUN and SH3 domain containing 1 (RUSC1), mRNA [NM_014328]	**1.251**
AP3D1	Homo sapiens adaptor-related protein complex 3, delta 1 subunit (AP3D1), mRNA [NM_003938]	**1.249**
VIM	Homo sapiens vimentin (VIM), mRNA [NM_003380]	**1.249**
WDR82	Homo sapiens WD repeat domain 82 (WDR82), mRNA [NM_025222]	**1.248**
BRD3	Homo sapiens bromodomain containing 3 (BRD3), mRNA [NM_007371]	**1.248**
GORASP1	Homo sapiens golgi reassembly stacking protein 1, 65kDa (GORASP1), mRNA [NM_031899]	**1.248**
NOL12	Homo sapiens nucleolar protein 12 (NOL12), mRNA [NM_024313]	**1.248**
PVR	Homo sapiens poliovirus receptor (PVR), mRNA [NM_006505]	**1.248**
SF4	Homo sapiens splicing factor 4 (SF4), mRNA [NM_172231]	**1.248**
TARS2	Homo sapiens threonyl-tRNA synthetase 2, mitochondrial (putative) (TARS2), mRNA	**1.248**

	[NM_025150]	
ZEB1	Homo sapiens zinc finger E-box binding homeobox 1 (ZEB1), mRNA [NM_030751]	**1.248**
PI4KB	Homo sapiens phosphatidylinositol 4-kinase, catalytic, beta (PI4KB), mRNA [NM_002651]	**1.248**
ABCF1	Homo sapiens ATP-binding cassette, sub-family F (GCN20), member 1 (ABCF1), mRNA [NM_001025091]	**1.247**
CAMK1	Homo sapiens calcium/calmodulin-dependent protein kinase I (CAMK1), mRNA [NM_003656]	**1.247**
LPCAT4	Homo sapiens lysophosphatidylcholine acyltransferase 4 (LPCAT4), mRNA [NM_153613]	**1.247**
TUBGCP6	Homo sapiens tubulin, gamma complex associated protein 6 (TUBGCP6), mRNA [NM_020461]	**1.247**
KIF14	Homo sapiens kinesin family member 14 (KIF14), mRNA [NM_014875]	**1.245**
LL22NC03-75B3.6	Homo sapiens KIAA1644 protein (KIAA1644), mRNA [NM_001099294]	**1.243**
SEC61A1	Homo sapiens Sec61 alpha 1 subunit (S. cerevisiae) (SEC61A1), mRNA [NM_013336]	**1.241**
FEN1	Homo sapiens flap structure-specific endonuclease 1 (FEN1), mRNA [NM_004111]	**1.240**
FUBP3	Homo sapiens far upstream element (FUSE) binding protein 3 (FUBP3), mRNA [NM_003934]	**1.239**
TCF25	Homo sapiens transcription factor 25 (basic helix-loop-helix) (TCF25), mRNA [NM_014972]	**1.238**
RCOR2	Homo sapiens REST corepressor 2 (RCOR2), mRNA [NM_173587]	**1.238**
LOC441455	Homo sapiens similar to Makorin-1 (RING finger protein 61), mRNA (cDNA clone IMAGE:5556543), [BC031344]	**1.237**
SPSB3	Homo sapiens splA/ryanodine receptor domain and SOCS box containing 3 (SPSB3), mRNA [NM_080861]	**1.237**
INPP5A	Homo sapiens inositol polyphosphate-5-phosphatase, 40kDa (INPP5A), mRNA [NM_005539]	**1.234**
ATF4C	Homo sapiens similar to activating transcription factor 4, (LOC643159), mRNA [XM_928637]	**1.233**
SMG5	Homo sapiens Smg-5 homolog, nonsense mediated mRNA decay factor (C. elegans) (SMG5), mRNA [NM_015327]	**1.233**
MAZ	Homo sapiens MYC-associated zinc finger protein (purine-binding transcription factor) (MAZ), mRNA [NM_001042539]	**1.232**
LOC442308	Homo sapiens similar to tubulin, beta 5 (LOC442308), non-coding RNA [NR_003598]	**1.232**
PRMT5	Homo sapiens protein arginine methyltransferase 5 (PRMT5), mRNA [NM_001039619]	**1.232**
ASNA1	Homo sapiens arsA arsenite transporter, ATP-binding, homolog 1 (bacterial) (ASNA1), mRNA [NM_004317]	**1.231**
SV2A	Homo sapiens synaptic vesicle glycoprotein 2A (SV2A), mRNA [NM_014849]	**1.230**
ALDH1B1	Homo sapiens aldehyde dehydrogenase 1 family, member B1 (ALDH1B1), mRNA [NM_000692]	**1.229**
TXN2	Homo sapiens thioredoxin 2 (TXN2), mRNA [NM_012473]	**1.229**
MYO1C	Homo sapiens myosin IC (MYO1C), mRNA [NM_033375]	**1.229**
LOC729497	Homo sapiens misc_RNA (LOC729497), miscRNA [XR_015501]	**1.228**

GUSBL1	Homo sapiens glucuronidase, beta-like 1 (GUSBL1), non-coding RNA [NR_003504]	**1.227**
CXorf56	Homo sapiens chromosome X open reading frame 56 (CXorf56), mRNA [NM_022101]	**1.227**
BSG	Homo sapiens basigin (Ok blood group) (BSG), mRNA [NM_001728]	**1.227**
BCAT2	Homo sapiens branched chain aminotransferase 2, mitochondrial (BCAT2), nmRNA [NM_001190]	**1.227**
EVX1	Homo sapiens even-skipped homeobox 1 (EVX1), mRNA [NM_001989]	**1.226**
LOC204010	Homo sapiens ribosomal protein SA pseudogene, mRNA (cDNA clone IMAGE:4095371). [BC107865]	**1.226**
NOB1	Homo sapiens NIN1/RPN12 binding protein 1 homolog (NOB1), mRNA [NM_014062]	**1.226**
NFKBIL2	Homo sapiens nuclear factor of kappa light polypeptide gene enhancer in B-cells inhibitor-like 2 (NFKBIL2), mRNA [NM_013432]	**1.225**
PPIE	Homo sapiens peptidylprolyl isomerase E (cyclophilin E) (PPIE), mRNA [NM_203456]	**1.225**
USP43	Homo sapiens ubiquitin specific peptidase 43 (USP43), mRNA [NM_153210]	**1.224**
TBCD	Homo sapiens tubulin folding cofactor D (TBCD), mRNA [NM_005993]	**1.224**
KCTD5	Homo sapiens potassium channel tetramerisation domain containing 5 (KCTD5), mRNA [NM_018992]	**1.221**
SLC16A1	Homo sapiens solute carrier family 16, member 1 (SLC16A1), mRNA [NM_003051]	**1.221**
VRK3	Homo sapiens vaccinia related kinase 3 (VRK3), mRNA [NM_016440]	**1.221**
ZNF653	Homo sapiens zinc finger protein 653 (ZNF653), mRNA [NM_138783]	**1.221**
EHMT2	Homo sapiens euchromatic histone-lysine N-methyltransferase 2 (EHMT2),mRNA [NM_025256]	**1.221**
CCNY	Homo sapiens cyclin Y (CCNY), mRNA [NM_145012]	**1.220**
LIMS1	Homo sapiens LIM and senescent cell antigen-like domains 1 (LIMS1), mRNA [NM_004987]	**1.219**
CNTROB	Homo sapiens centrobin, centrosomal BRCA2 interacting protein (CNTROB),mRNA [NM_001037144]	**1.219**
TELO2	Homo sapiens TEL2, telomere maintenance 2, homolog (S. cerevisiae) (TELO2), mRNA [NM_016111]	**1.218**
KCTD17	Homo sapiens potassium channel tetramerisation domain containing 17 (KCTD17), mRNA [NM_024681]	**1.218**
EZR	Homo sapiens ezrin (EZR), mRNA [NM_003379]	**1.217**
HNRNPCL1	Homo sapiens heterogeneous nuclear ribonucleoprotein C-like 1 (HNRNPCL1), mRNA [NM_001013631]	**1.217**
MKS1	Homo sapiens cDNA FLJ20345 fis, clone HEP13723. [AK000352]	**1.217**
NAGPA	Homo sapiens N-acetylglucosamine-1-phosphodiester alpha-N-acetylglucosaminidase (NAGPA), mRNA [NM_016256]	**1.217**
EIF2S3	Homo sapiens eukaryotic translation initiation factor 2, subunit 3 gamma, 52kDa (EIF2S3), mRNA [NM_001415]	**1.216**
SULT1A1	Homo sapiens sulfotransferase family, cytosolic, 1A, phenol-preferring, member 1 (SULT1A1), mRNA [NM_177529]	**1.216**
MGC2752	Homo sapiens hypothetical LOC65996 (MGC2752), non-coding RNA [NR_026052]	**1.215**
UAP1L1	Homo sapiens UDP-N-acteylglucosamine pyrophosphorylase 1-like 1 (UAP1L1), mRNA [NM_207309]	**1.215**

B4GALNT4	Homo sapiens beta-1,4-N-acetyl-galactosaminyl transferase 4 (B4GALNT4), mRNA [NM_178537]	**1.214**
MTP18	Homo sapiens mitochondrial protein 18 kDa (MTP18), nmRNA [NM_016498]	**1.213**
TBC1D2	Homo sapiens TBC1 domain family, member 2 (TBC1D2), mRNA [NM_018421]	**1.213**
PURA	Homo sapiens purine-rich element binding protein A (PURA), mRNA [NM_005859]	**1.212**
WBSCR16	Homo sapiens Williams-Beuren syndrome chromosome region 16, mRNA (cDNA clone MGC:49849 IMAGE:5785681), [BC040695]	**1.212**
C17orf53	Homo sapiens chromosome 17 open reading frame 53 (C17orf53), mRNA [NM_024032]	**1.212**
NONO	Homo sapiens non-POU domain containing, octamer-binding (NONO), mRNA [NM_007363]	**1.211**
PRSS21	Homo sapiens protease, serine, 21 (testisin) (PRSS21), mRNA [NM_006799]	**1.211**
ENDOG	Homo sapiens endonuclease G (ENDOG), mRNA [NM_004435]	**1.209**
RXRB	Homo sapiens retinoid X receptor, beta (RXRB), mRNA [NM_021976]	**1.209**
CDKN1A	Homo sapiens cyclin-dependent kinase inhibitor 1A (p21, Cip1) (CDKN1A), mRNA [NM_000389]	**1.209**
C1orf115	Homo sapiens chromosome 1 open reading frame 115 (C1orf115), mRNA [NM_024709]	**1.208**
TGS1	Homo sapiens trimethylguanosine synthase homolog (TGS1), mRNA [NM_024831]	**1.208**
NKIRAS2	Homo sapiens NFKB inhibitor interacting Ras-like 2 (NKIRAS2), mRNA [NM_001001349]	**1.208**
LOC100131067	Homo sapiens cDNA FLJ38695 fis, clone KIDNE2001897. [AK096014]	**1.207**
PDE3A	Homo sapiens phosphodiesterase 3A, cGMP-inhibited (PDE3A), mRNA [NM_000921]	**1.207**
SART1	Homo sapiens squamous cell carcinoma antigen recognized by T cells (SART1), mRNA [NM_005146]	**1.206**
AKT1	Homo sapiens v-akt murine thymoma viral oncogene homolog 1 (AKT1), transcript variant 1, mRNA [NM_005163]	**1.205**
RBCK1	Homo sapiens RanBP-type and C3HC4-type zinc finger containing 1 (RBCK1), transcript variant 2, mRNA [NM_031229]	**1.205**
CPNE2	Homo sapiens copine II (CPNE2), mRNA [NM_152727]	**1.204**
SLCO3A1	Homo sapiens solute carrier organic anion transporter family, member 3A1 (SLCO3A1), mRNA [NM_013272]	**1.203**
ZNF747	Homo sapiens zinc finger protein 747 (ZNF747), mRNA [NM_023931]	**1.203**
VGLL4	Homo sapiens vestigial like 4 (Drosophila) (VGLL4), mRNA [NM_014667]	**1.203**
RABGGTA	Homo sapiens Rab geranylgeranyltransferase, alpha subunit (RABGGTA),mRNA [NM_004581]	**1.202**
SCRN2	Homo sapiens secernin 2 (SCRN2), mRNA [NM_138355]	**1.202**
MEPCE	Homo sapiens methylphosphate capping enzyme (MEPCE), mRNA [NM_019606]	**1.201**
TJP2	Homo sapiens tight junction protein 2 (zona occludens 2) (TJP2), mRNA [NM_004817]	**1.201**
AKR7A3	Homo sapiens aldo-keto reductase family 7, member A3 (AKR7A3), mRNA [NM_012067]	**1.197**
TP53	Homo sapiens tumor protein p53 (TP53), mRNA [NM_000546]	**1.197**
CD276	Homo sapiens CD276 molecule (CD276), mRNA [NM_001024736]	**1.196**
MAP1S	Homo sapiens microtubule-associated protein 1S (MAP1S), mRNA [NM_018174]	**1.196**
SDHC	Homo sapiens succinate dehydrogenase complex, subunit C, integral membrane protein,	**1.196**

	15kDa (SDHC), mRNA [NM_003001]	
PEX10	Homo sapiens peroxisomal biogenesis factor 10 (PEX10), mRNA [NM_002617]	**1.195**
PLDN	Homo sapiens pallidin homolog (mouse) (PLDN), mRNA [NM_012388]	**1.194**
ARID3A	Homo sapiens AT rich interactive domain 3A (BRIGHT-like) (ARID3A), mRNA [NM_005224]	**1.194**
FBXW4	Homo sapiens F-box and WD repeat domain containing 4 (FBXW4), mRNA [NM_022039]	**1.194**
HMGB1L1	Homo sapiens high-mobility group box 1-like 1 (HMGB1L1), mRNA [NM_001008735]	**1.194**
SEC16A	Homo sapiens SEC16 homolog A (S. cerevisiae) (SEC16A), mRNA [NM_014866]	**1.194**
ATG2A	Homo sapiens ATG2 autophagy related 2 homolog A (S. cerevisiae) (ATG2A), mRNA [NM_015104]	**1.193**
POLL	Homo sapiens polymerase (DNA directed), lambda (POLL), mRNA [NM_013274]	**1.193**
TSC2	Homo sapiens tuberous sclerosis 2 (TSC2), mRNA [NM_000548]	**1.192**
MAN1B1	Homo sapiens mannosidase, alpha, class 1B, member 1 (MAN1B1), mRNA [NM_016219]	**1.192**
AFMID	Homo sapiens arylformamidase (AFMID), mRNA [NM_001010982]	**1.191**
RASL10B	Homo sapiens RAS-like, family 10, member B (RASL10B), mRNA [NM_033315]	**1.191**
OLFML2A	Homo sapiens olfactomedin-like 2A (OLFML2A), mRNA [NM_182487]	**1.190**
UBE2NL	Homo sapiens ubiquitin-conjugating enzyme E2N-like (UBE2NL), mRNA [NM_001012989]	**1.189**
FAM120B	Homo sapiens family with sequence similarity 120B (FAM120B), mRNA [NM_032448]	**1.188**
FAM83H	Homo sapiens family with sequence similarity 83, member H (FAM83H), mRNA [NM_198488]	**1.188**
LOC440292	Homo sapiens similar to hCG1731366 (LOC440292), mRNA [XM_496078]	**1.187**
LOC161635	Homo sapiens, clone IMAGE:5166482, mRNA, [BC028192]	**1.186**
RELA	Homo sapiens v-rel reticuloendotheliosis viral oncogene homolog A (avian), mRNA (cDNA clone MGC:131774 IMAGE:6019711), [BC110830]	**1.185**
ZNF346	Homo sapiens zinc finger protein 346 (ZNF346), mRNA [NM_012279]	**1.185**
ALG12	Homo sapiens asparagine-linked glycosylation 12, alpha-1,6-mannosyltransferase homolog (S. cerevisiae) (ALG12), mRNA [NM_024105]	**1.183**
PDE2A	Homo sapiens phosphodiesterase 2A, cGMP-stimulated (PDE2A), mRNA [NM_002599]	**1.183**
NCOA5	Homo sapiens nuclear receptor coactivator 5 (NCOA5), mRNA [NM_020967]	**1.183**
FURIN	Homo sapiens furin (paired basic amino acid cleaving enzyme) (FURIN), mRNA [NM_002569]	**1.181**
LOC100132831	Homo sapiens misc_RNA (LOC100132831), miscRNA [XR_037012]	**1.181**
LOC728820	Homo sapiens misc_RNA (LOC728820), miscRNA [XR_015551]	**1.181**
MIB2	Homo sapiens mindbomb homolog 2 (Drosophila) (MIB2), mRNA [NM_080875]	**1.181**
NIPSNAP1	Homo sapiens nipsnap homolog 1 (C. elegans) (NIPSNAP1), mRNA [NM_003634]	**1.181**
SLC35E1	Homo sapiens solute carrier family 35, member E1 (SLC35E1), mRNA [NM_024881]	**1.181**
PPAN	Homo sapiens peter pan homolog (Drosophila) (PPAN), mRNA [NM_020230]	**1.180**
BBX	Homo sapiens bobby sox homolog (Drosophila) (BBX), mRNA [NM_020235]	**1.179**
GADD45A	Homo sapiens growth arrest and DNA-damage-inducible, alpha (GADD45A), mRNA [NM_001924]	**1.179**

DNAJC11	Homo sapiens DnaJ (Hsp40) homolog, subfamily C, member 11 (DNAJC11), mRNA [NM_018198]	**1.179**
APEX1	Homo sapiens APEX nuclease (multifunctional DNA repair enzyme) 1 (APEX1), mRNA [NM_080649]	**1.178**
EDARADD	Homo sapiens EDAR-associated death domain (EDARADD), mRNA [NM_080738]	**1.178**
DDX51	Homo sapiens DEAD (Asp-Glu-Ala-Asp) box polypeptide 51 (DDX51), mRNA [NM_175066]	**1.177**
KRT19P2	Homo sapiens mRNA for keratin 19, partial cds, isolate:K19-141. [AB041269]	**1.177**
NCDN	Homo sapiens neurochondrin (NCDN), mRNA [NM_014284]	**1.177**
PDAP1	Homo sapiens PDGFA associated protein 1 (PDAP1), mRNA [NM_014891]	**1.177**
ZNF205	Homo sapiens zinc finger protein 205 (ZNF205), mRNA [NM_003456]	**1.176**
LPCAT1	Homo sapiens lysophosphatidylcholine acyltransferase 1 (LPCAT1), mRNA [NM_024830]	**1.176**
CLPTM1L	Homo sapiens CLPTM1-like (CLPTM1L), mRNA [NM_030782]	**1.174**
RNF5	Homo sapiens ring finger protein 5 (RNF5), mRNA [NM_006913]	**1.173**
GDPD5	Homo sapiens glycerophosphodiester phosphodiesterase domain containing 5 (GDPD5), mRNA [NM_030792]	**1.173**
NUDC	Homo sapiens nuclear distribution gene C homolog (A. nidulans) (NUDC), mRNA [NM_006600]	**1.173**
DAP	Homo sapiens death-associated protein (DAP), mRNA [NM_004394]	**1.173**
CKB	Homo sapiens creatine kinase, brain (CKB), mRNA [NM_001823]	**1.173**
DDX24	Homo sapiens DEAD (Asp-Glu-Ala-Asp) box polypeptide 24 (DDX24), mRNA [NM_020414]	**1.172**
TOMM34	Homo sapiens translocase of outer mitochondrial membrane 34 (TOMM34), mRNA [NM_006809]	**1.172**
BAT2	Homo sapiens HLA-B associated transcript 2 (BAT2), mRNA [NM_080686]	**1.171**
CDK5	Homo sapiens cyclin-dependent kinase 5 (CDK5), mRNA [NM_004935]	**1.171**
NPLOC4	Homo sapiens nuclear protein localization 4 homolog (S. cerevisiae) (NPLOC4), mRNA [NM_017921]	**1.171**
ZNF146	Homo sapiens zinc finger protein 146 (ZNF146), mRNA [NM_007145]	**1.171**
KIF26A	Homo sapiens kinesin family member 26A (KIF26A), mRNA [NM_015656]	**1.170**
MAN2B2	Homo sapiens mannosidase, alpha, class 2B, member 2 (MAN2B2), mRNA [NM_015274]	**1.170**
ZNF607	Homo sapiens zinc finger protein 607 (ZNF607), mRNA [NM_032689]	**1.170**
RPL7L1	Homo sapiens ribosomal protein L7-like 1 (RPL7L1), mRNA [NM_198486]	**1.169**
ETS1	Homo sapiens v-ets erythroblastosis virus E26 oncogene homolog 1 (avian) (ETS1), mRNA [NM_005238]	**1.169**
AGAP8	Homo sapiens ArfGAP with GTPase domain, ankyrin repeat and PH domain 8 (AGAP8), mRNA [NM_001077686]	**1.168**
LENG1	Homo sapiens leukocyte receptor cluster (LRC) member 1 (LENG1), mRNA [NM_024316]	**1.167**
LOC100128056	Homo sapiens similar to hCG2013701 (LOC100128056), mRNA [XM_001720210]	**1.167**
PEMT	Homo sapiens phosphatidylethanolamine N-methyltransferase (PEMT), mRNA	**1.166**

	[NM_007169]	
PKN1	Homo sapiens protein kinase N1 (PKN1), mRNA [NM_002741]	**1.166**
MCAT	Homo sapiens malonyl CoA:ACP acyltransferase (mitochondrial) (MCAT), mRNA [NM_014507]	**1.166**
EPHB2	Homo sapiens EPH receptor B2 (EPHB2), mRNA [NM_004442]	**1.165**
FDPSL2A	Homo sapiens MGC44478 (FDPSL2A), non-coding RNA [NR_003262]	**1.165**
MAP3K1	Homo sapiens mitogen-activated protein kinase kinase kinase 1 (MAP3K1), mRNA [NM_005921]	**1.164**
PRPF8	Homo sapiens PRP8 pre-mRNA processing factor 8 homolog (S. cerevisiae) (PRPF8), mRNA [NM_006445]	**1.164**
ADAMTS16	Homo sapiens ADAM metallopeptidase with thrombospondin type 1 motif, 16 (ADAMTS16), mRNA [NM_139056]	**1.163**
TMEM179B	Homo sapiens transmembrane protein 179B (TMEM179B), mRNA [NM_199337]	**1.163**
CANT1	Homo sapiens calcium activated nucleotidase 1 (CANT1), mRNA [NM_138793]	**1.161**
HERPUD2	Homo sapiens HERPUD family member 2 (HERPUD2), mRNA [NM_022373]	**1.160**
PPP2R4	Homo sapiens protein phosphatase 2A activator, regulatory subunit 4 (PPP2R4), mRNA [NM_178001]	**1.160**
AIP	Homo sapiens aryl hydrocarbon receptor interacting protein (AIP), mRNA [NM_003977]	**1.160**
ALDH3B1	Homo sapiens cDNA FLJ26433 fis, clone KDN01585, highly similar to Aldehyde dehydrogenase 7 (EC 1.2.1.5). [AK129943]	**1.159**
ASF1B	Homo sapiens ASF1 anti-silencing function 1 homolog B (S. cerevisiae) (ASF1B), mRNA [NM_018154]	**1.159**
hCG_1984468	Homo sapiens hCG1984468 (LOC389672), mRNA [NM_001089588]	**1.159**
LARP1	Homo sapiens La ribonucleoprotein domain family, member 1 (LARP1), mRNA [NM_033551]	**1.159**
DHRS4	Homo sapiens dehydrogenase/reductase (SDR family) member 4 (DHRS4), mRNA [NM_021004]	**1.158**
KRT18P33	Homo sapiens keratin 18 pseudogene 33 (KRT18P33), mRNA [XR_019330]	**1.158**
RYK	Homo sapiens RYK receptor-like tyrosine kinase (RYK), mRNA [NM_001005861]	**1.158**
TTC5	Homo sapiens tetratricopeptide repeat domain 5 (TTC5), mRNA [NM_138376]	**1.157**
DAGLB	Homo sapiens diacylglycerol lipase, beta (DAGLB), mRNA [NM_139179]	**1.156**
RPN2	Homo sapiens ribophorin II (RPN2), mRNA [NM_002951]	**1.156**
CHMP1A	Homo sapiens chromatin modifying protein 1A (CHMP1A), mRNA [NM_002768]	**1.155**
SLC39A14	Homo sapiens solute carrier family 39 (zinc transporter), member 14 (SLC39A14), mRNA [NM_015359]	**1.155**
RNPEPL1	Homo sapiens arginyl aminopeptidase (aminopeptidase B)-like 1 (RNPEPL1), mRNA [NM_018226]	**1.154**
LTBP2	Homo sapiens latent transforming growth factor beta binding protein 2 (LTBP2), mRNA [NM_000428]	**1.153**
MCRS1	Homo sapiens microspherule protein 1 (MCRS1), mRNA [NM_006337]	**1.153**
NME4	Homo sapiens non-metastatic cells 4, protein expressed in (NME4), mRNA [NM_005009]	**1.153**
VCP	Homo sapiens valosin-containing protein (VCP), mRNA [NM_007126]	**1.153**

ITGB1	Homo sapiens integrin, beta 1 (fibronectin receptor, beta polypeptide, antigen CD29 includes MDF2, MSK12) (ITGB1), mRNA [NM_133376]	**1.152**
DDRGK1	Homo sapiens DDRGK domain containing 1 (DDRGK1), mRNA [NM_023935]	**1.152**
TEAD2	Homo sapiens TEA domain family member 2 (TEAD2), mRNA [NM_003598]	**1.152**
ECH1	Homo sapiens enoyl Coenzyme A hydratase 1, peroxisomal (ECH1), mRNA [NM_001398]	**1.151**
SMEK1	Homo sapiens SMEK homolog 1, suppressor of mek1 (Dictyostelium) (SMEK1), mRNA [NM_032560]	**1.151**
LZTR1	Homo sapiens leucine-zipper-like transcription regulator 1 (LZTR1), mRNA [NM_006767]	**1.150**
MZF1	Homo sapiens myeloid zinc finger 1 (MZF1), mRNA [NM_198055]	**1.150**
CCDC115	Homo sapiens coiled-coil domain containing 115 (CCDC115), mRNA [NM_032357]	**1.149**
CSDA	Homo sapiens cold shock domain protein A (CSDA), mRNA [NM_003651]	**1.149**
NADK	Homo sapiens NAD kinase (NADK), mRNA [NM_023018]	**1.149**
PFKL	Homo sapiens phosphofructokinase, liver (PFKL), mRNA [NM_002626]	**1.148**
INTS3	Homo sapiens integrator complex subunit 3 (INTS3), mRNA [NM_023015]	**1.147**
KHK	Homo sapiens ketohexokinase (fructokinase) (KHK), mRNA [NM_000221]	**1.147**
PROSC	Homo sapiens proline synthetase co-transcribed homolog (bacterial) (PROSC), mRNA [NM_007198]	**1.147**
LOC643562	Homo sapiens misc_RNA (LOC643562), miscRNA [XR_016539]	**1.146**
DYNC1H1	Homo sapiens dynein, cytoplasmic 1, heavy chain 1 (DYNC1H1), mRNA [NM_001376]	**1.145**
LOC646672	Homo sapiens misc_RNA (LOC646672), miscRNA [XR_018091]	**1.145**
SEMA4D	Homo sapiens sema domain, immunoglobulin domain, transmembrane domain and short cytoplasmic domain, (semaphorin) 4D (SEMA4D), mRNA [NM_001142287]	**1.145**
ICAM5	Homo sapiens intercellular adhesion molecule 5, telencephalin (ICAM5), mRNA [NM_003259]	**1.144**
LRPAP1	Homo sapiens low density lipoprotein receptor-related protein associated protein 1 (LRPAP1), mRNA [NM_002337]	**1.143**
ITSN2	Homo sapiens intersectin 2 (ITSN2), mRNA [NM_147152]	**1.143**
TRPM7	Homo sapiens transient receptor potential cation channel, subfamily M, member 7 (TRPM7), mRNA [NM_017672]	**1.143**
AP1S1	Homo sapiens adaptor-related protein complex 1, sigma 1 subunit (AP1S1), mRNA [NM_001283]	**1.142**
GTSE1	Homo sapiens G-2 and S-phase expressed 1 (GTSE1), mRNA [NM_016426]	**1.142**
LRFN4	Homo sapiens leucine rich repeat and fibronectin type III domain containing 4 (LRFN4), mRNA [NM_024036]	**1.142**
RPL4	Homo sapiens ribosomal protein L4 (RPL4), mRNA [NM_000968]	**1.142**
CAPNS1	Homo sapiens calpain, small subunit 1 (CAPNS1), mRNA [NM_001749]	**1.141**
DEDD2	Homo sapiens death effector domain containing 2 (DEDD2), mRNA [NM_133328]	**1.141**
TUBGCP3	Homo sapiens tubulin, gamma complex associated protein 3 (TUBGCP3), mRNA [NM_006322]	**1.140**
VPS52	Homo sapiens vacuolar protein sorting 52 homolog (S. cerevisiae) (VPS52), mRNA [NM_022553]	**1.140**

TAGLN	Homo sapiens transgelin (TAGLN), mRNA [NM_001001522]	**1.140**
CCDC21	Homo sapiens coiled-coil domain containing 21 (CCDC21), mRNA [NM_022778]	**1.139**
TRMT61A	Homo sapiens chromosome 14 open reading frame 172 (C14orf172), mRNA [NM_152307]	**1.139**
GTPBP6	Homo sapiens GTP binding protein 6 (putative) (GTPBP6), mRNA [NM_012227]	**1.138**
ROCK2	Homo sapiens Rho-associated, coiled-coil containing protein kinase 2 (ROCK2), mRNA [NM_004850]	**1.138**
TRIP13	Homo sapiens thyroid hormone receptor interactor 13 (TRIP13), mRNA [NM_004237]	**1.138**
PPME1	Homo sapiens protein phosphatase methylesterase 1 (PPME1), mRNA [NM_016147]	**1.137**
UBAC2	Homo sapiens UBA domain containing 2 (UBAC2), mRNA [NM_177967]	**1.136**
CYB561	Homo sapiens cytochrome b-561 (CYB561), mRNA [NM_001017916]	**1.136**
RNF20	Homo sapiens ring finger protein 20 (RNF20), mRNA [NM_019592]	**1.135**
RPP25	Homo sapiens ribonuclease P/MRP 25kDa subunit (RPP25), mRNA [NM_017793]	**1.135**
WBP1	Homo sapiens WW domain binding protein 1 (WBP1), mRNA [NM_012477]	**1.135**
LDLR	Homo sapiens low density lipoprotein receptor (LDLR), mRNA [NM_000527]	**1.134**
DNM2	Homo sapiens dynamin 2 (DNM2), mRNA [NM_001005360]	**1.134**
GNB1	Homo sapiens guanine nucleotide binding protein (G protein), beta polypeptide 1 (GNB1), mRNA [NM_002074]	**1.134**
GRIPAP1	Homo sapiens GRIP1 associated protein 1 (GRIPAP1), mRNA [NM_020137]	**1.134**
MORG1	Homo sapiens mitogen-activated protein kinase organizer 1 (MORG1),mRNA [NM_032332]	**1.134**
CSPG5	Homo sapiens chondroitin sulfate proteoglycan 5 (neuroglycan C) (CSPG5), mRNA [NM_006574]	**1.133**
EIF3C	Homo sapiens eukaryotic translation initiation factor 3, subunit C (EIF3C),mRNA [NM_001037808]	**1.133**
LCE1D	Homo sapiens late cornified envelope 1D (LCE1D), mRNA [NM_178352]	**1.133**
LIG1	Homo sapiens ligase I, DNA, ATP-dependent (LIG1), mRNA [NM_000234]	**1.133**
PHB	Homo sapiens prohibitin (PHB), mRNA [NM_002634]	**1.133**
BMP8B	Homo sapiens bone morphogenetic protein 8b (BMP8B), mRNA [NM_001720]	**1.132**
LOC731275	Homo sapiens hypothetical LOC731275 (LOC731275), mRNA [XM_001726998]	**1.132**
PISD	Homo sapiens phosphatidylserine decarboxylase (PISD), mRNA [NM_014338]	**1.132**
PTBP1	Homo sapiens polypyrimidine tract binding protein 1 (PTBP1), mRNA [NM_002819]	**1.131**
TACC3	Homo sapiens transforming, acidic coiled-coil containing protein 3 (TACC3), mRNA [NM_006342]	**1.131**
TFDP3	Homo sapiens transcription factor Dp family, member 3 (TFDP3), mRNA [NM_016521]	**1.131**
HCCA2	Homo sapiens HCCA2 protein (HCCA2), mRNA [NM_053005]	**1.130**
RHBDD3	Homo sapiens rhomboid domain containing 3 (RHBDD3), mRNA [NM_012265]	**1.130**
NCOA4	Homo sapiens nuclear receptor coactivator 4 (NCOA4), mRNA [NM_005437]	**1.130**
TMEM222	Homo sapiens transmembrane protein 222 (TMEM222), mRNA [NM_032125]	**1.129**
AKAP2	Homo sapiens A kinase (PRKA) anchor protein 2 (AKAP2), mRNA [NM_001004065]	**1.128**
RPS6KA4	Homo sapiens ribosomal protein S6 kinase, 90kDa, polypeptide 4 (RPS6KA4), mRNA [NM_003942]	**1.128**
TAZ	Homo sapiens tafazzin (TAZ), mRNA [NM_000116]	**1.128**

C8orf41	Homo sapiens chromosome 8 open reading frame 41 (C8orf41), mRNA [NM_025115]	**1.127**
PTPN14	Homo sapiens protein tyrosine phosphatase, non-receptor type 14 (PTPN14), mRNA [NM_005401]	**1.127**
ABCF2	Homo sapiens ATP-binding cassette, sub-family F (GCN20), member 2 (ABCF2), n mRNA [NM_005692]	**1.127**
ZYX	Homo sapiens zyxin (ZYX), mRNA [NM_003461]	**1.126**
PRKAR1B	Homo sapiens protein kinase, cAMP-dependent, regulatory, type I, beta (PRKAR1B), mRNA [NM_002735]	**1.126**
SCAND1	Homo sapiens SCAN domain containing 1 (SCAND1), mRNA [NM_016558]	**1.126**
C10orf75	Homo sapiens cDNA FLJ12974 fis, clone NT2RP2006103. [AK023036]	**1.125**
DDX41	Homo sapiens DEAD (Asp-Glu-Ala-Asp) box polypeptide 41 (DDX41), mRNA [NM_016222]	**1.125**
hCG_1783494	Homo sapiens cDNA FLJ59642 complete cds, highly similar to Supervillin. [AK302694]	**1.125**
MEIS3	Homo sapiens Meis homeobox 3 (MEIS3), mRNA [NM_001009813]	**1.125**
FBLIM1	Homo sapiens filamin binding LIM protein 1 (FBLIM1), mRNA [NM_001024215]	**1.123**
LIF	Homo sapiens leukemia inhibitory factor (cholinergic differentiation factor) (LIF), mRNA [NM_002309]	**1.123**
LOC285412	PREDICTED: Homo sapiens misc_RNA (LOC285412), miscRNA [XR_019409]	**1.122**
MN1	Homo sapiens meningioma (disrupted in balanced translocation) 1 (MN1), mRNA [NM_002430]	**1.122**
FAM32A	Homo sapiens family with sequence similarity 32, member A (FAM32A), mRNA [NM_014077]	**1.121**
MAST2	Homo sapiens microtubule associated serine/threonine kinase 2 (MAST2), mRNA [NM_015112]	**1.121**
NTHL1	Homo sapiens nth endonuclease III-like 1 (E. coli) (NTHL1), mRNA [NM_002528]	**1.121**
SGSM2	Homo sapiens small G protein signaling modulator 2 (SGSM2), mRNA [NM_014853]	**1.121**
FAM108A1	Homo sapiens family with sequence similarity 108, member A1 (FAM108A1),mRNA [NM_031213]	**1.120**
KLF6	Homo sapiens Kruppel-like factor 6 (KLF6), mRNA [NM_001300]	**1.120**
CYR61	Homo sapiens cysteine-rich, angiogenic inducer, 61 (CYR61), mRNA [NM_001554]	**1.119**
KIF20A	Homo sapiens kinesin family member 20A (KIF20A), mRNA [NM_005733]	**1.118**
CDK3	Homo sapiens cyclin-dependent kinase 3 (CDK3), mRNA [NM_001258]	**1.117**
JMJD2A	Homo sapiens jumonji domain containing 2A (JMJD2A), mRNA [NM_014663]	**1.117**
NDUFV1	Homo sapiens NADH dehydrogenase (ubiquinone) flavoprotein 1, 51kDa (NDUFV1), mRNA [NM_007103]	**1.117**
ARF1	Homo sapiens ADP-ribosylation factor 1 (ARF1), mRNA [NM_001024227]	**1.117**
ADCK5	Homo sapiens aarF domain containing kinase 5 (ADCK5), mRNA [NM_174922]	**1.116**
RPS3	Homo sapiens ribosomal protein S3 (RPS3), mRNA [NM_001005]	**1.116**
SAMD4B	Homo sapiens sterile alpha motif domain containing 4B (SAMD4B), mRNA [NM_018028]	**1.116**
SF3B2	Homo sapiens splicing factor 3b, subunit 2, 145kDa (SF3B2), mRNA [NM_006842]	**1.116**
UBR4	Homo sapiens ubiquitin protein ligase E3 component n-recognin 4 (UBR4), mRNA [NM_020765]	**1.116**

OBSL1	Homo sapiens cDNA FLJ13792 fis, clone THYRO1000072, weakly similar to Myosin Light Chain Kinase, Smooth Muscle And Non-Muscle Isozymes (EC 2.7.1.117). [AK023854]	**1.115**
RPUSD3	Homo sapiens RNA pseudouridylate synthase domain containing 3 (RPUSD3), mRNA [NM_173659]	**1.115**
SUPT16HP	Homo sapiens misc_RNA (LOC400011), miscRNA [XR_019334]	**1.115**
NELF	Homo sapiens nasal embryonic LHRH factor (NELF), mRNA [NM_015537]	**1.115**
KRT18P28	Homo sapiens similar to Keratin, type I cytoskeletal 18 (Cytokeratin-18) (CK-18) (Keratin-18) (K18) (LOC343326), mRNA [XR_019568]	**1.114**
POLA2	Homo sapiens polymerase (DNA directed), alpha 2 (70kD subunit) (POLA2), mRNA [NM_002689]	**1.114**
LOC494150	Homo sapiens prohibitin pseudogene, mRNA (cDNA clone IMAGE:4547239). [BC014228]	**1.114**
CWF19L1	Homo sapiens CWF19-like 1, cell cycle control (S. pombe) (CWF19L1), mRNA [NM_018294]	**1.113**
BAALC	Homo sapiens brain and acute leukemia, cytoplasmic (BAALC), mRNA [NM_001024372]	**1.112**
CDK5RAP1	Homo sapiens CDK5 regulatory subunit associated protein 1 (CDK5RAP1), mRNA [NM_016082]	**1.112**
PPL	Homo sapiens periplakin (PPL), mRNA [NM_002705]	**1.112**
UCKL1	Homo sapiens uridine-cytidine kinase 1-like 1 (UCKL1), mRNA [NM_017859]	**1.112**
LOC399900	Homo sapiens hypothetical gene supported by AK093779, mRNA (cDNA clone IMAGE:40146916). [BC132894]	**1.111**
DNAH2	Homo sapiens dynein, axonemal, heavy chain 2, mRNA (cDNA clone IMAGE:4839091), complete cds. [BC034225]	**1.110**
C9orf37	Homo sapiens chromosome 9 open reading frame 37 (C9orf37), mRNA [NM_032937]	**1.109**
ACTA2	Homo sapiens actin, alpha 2, smooth muscle, aorta (ACTA2), mRNA [NM_001613]	**1.107**
PRPF6	Homo sapiens PRP6 pre-mRNA processing factor 6 homolog (S. cerevisiae) (PRPF6), mRNA [NM_012469]	**1.107**
CARS	Homo sapiens cysteinyl-tRNA synthetase (CARS), mRNA [NM_001014438]	**1.106**
LIG3	Homo sapiens ligase III, DNA, ATP-dependent (LIG3), mRNA [NM_002311]	**1.106**
NOL6	Homo sapiens nucleolar protein family 6 (RNA-associated) (NOL6), mRNA [NM_022917]	**1.106**
BLVRB	Homo sapiens biliverdin reductase B (flavin reductase (NADPH)) (BLVRB), mRNA [NM_000713]	**1.105**
ALKBH6	Homo sapiens alkB, alkylation repair homolog 6 (E. coli) (ALKBH6), mRNA [NM_198867]	**1.104**
CYP1A1	Homo sapiens cytochrome P450, family 1, subfamily A, polypeptide 1 (CYP1A1), mRNA [NM_000499]	**1.104**
ERF	Homo sapiens Ets2 repressor factor (ERF), mRNA [NM_006494]	**1.103**
RTEL1	Homo sapiens regulator of telomere elongation helicase 1 (RTEL1), mRNA [NM_016434]	**1.103**
PCCB	Homo sapiens propionyl Coenzyme A carboxylase, beta polypeptide (PCCB), mRNA	**1.102**

	[NM_000532]	
RIN2	Homo sapiens Ras and Rab interactor 2 (RIN2), mRNA [NM_018993]	**1.102**
BDH1	Homo sapiens 3-hydroxybutyrate dehydrogenase, type 1 (BDH1), mRNA [NM_203314]	**1.101**
BOK	Homo sapiens Bcl-2 related ovarian killer (BOK) mRNA, complete cds. [AF089746]	**1.101**
F3	Homo sapiens coagulation factor III (thromboplastin, tissue factor) (F3), mRNA [NM_001993]	**1.101**
BCR	Homo sapiens breakpoint cluster region (BCR), mRNA [NM_004327]	**1.100**
MAD2L2	Homo sapiens MAD2 mitotic arrest deficient-like 2 (yeast) (MAD2L2), mRNA [NM_006341]	**1.099**
TMEM55B	Homo sapiens transmembrane protein 55B (TMEM55B), mRNA [NM_144568]	**1.099**
LOC100190986	Homo sapiens hypothetical LOC100190986 (LOC100190986), non-coding RNA [NR_024456]	**1.099**
EIF6	Homo sapiens eukaryotic translation initiation factor 6 (EIF6), mRNA [NM_181468]	**1.098**
MEN1	Homo sapiens multiple endocrine neoplasia I (MEN1), mRNA [NM_130803]	**1.098**
SH3TC1	Homo sapiens SH3 domain and tetratricopeptide repeats 1 (SH3TC1), mRNA [NM_018986]	**1.098**
TGIF2	Homo sapiens TGFB-induced factor homeobox 2 (TGIF2), mRNA [NM_021809]	**1.098**
TNFRSF10A	Homo sapiens tumor necrosis factor receptor superfamily, member 10a (TNFRSF10A), mRNA [NM_003844]	**1.098**
C21orf70	Homo sapiens chromosome 21 open reading frame 70 (C21orf70), mRNA [NM_058190]	**1.098**
ATP2B1	Homo sapiens ATPase, Ca++ transporting, plasma membrane 1 (ATP2B1),mRNA [NM_001682]	**1.097**
FKBPL	Homo sapiens FK506 binding protein like (FKBPL), mRNA [NM_022110]	**1.097**
HNRNPUL2	Homo sapiens heterogeneous nuclear ribonucleoprotein U-like 2 (HNRNPUL2), mRNA [NM_001079559]	**1.097**
LUZP1	Homo sapiens mRNA for FLJ00226 protein. [AK074153]	**1.097**
BCKDK	Homo sapiens branched chain ketoacid dehydrogenase kinase (BCKDK),mRNA [NM_005881]	**1.095**
MYL6	Homo sapiens myosin, light chain 6, alkali, smooth muscle and non-muscle (MYL6), mRNA [NM_079423]	**1.095**
PPP1R15A	Homo sapiens protein phosphatase 1, regulatory (inhibitor) subunit 15A (PPP1R15A), mRNA [NM_014330]	**1.095**
FLJ40504	Homo sapiens hypothetical protein FLJ40504 (FLJ40504), mRNA [NM_173624]	**1.094**
MIF	Homo sapiens macrophage migration inhibitory factor (glycosylation-inhibiting factor) (MIF), mRNA [NM_002415]	**1.094**
TRIB3	Homo sapiens tribbles homolog 3 (Drosophila) (TRIB3), mRNA [NM_021158]	**1.094**
DIO2	Homo sapiens deiodinase, iodothyronine, type II (DIO2), mRNA [NM_013989]	**1.091**
KDELR1	Homo sapiens KDEL (Lys-Asp-Glu-Leu) endoplasmic reticulum protein retention receptor 1 (KDELR1), mRNA [NM_006801]	**1.091**
FBXW4P1	Homo sapiens F-box protein Fbw3 (FBW3) mRNA, complete cds. [AF174606]	**1.090**
PIP5K1A	Homo sapiens phosphatidylinositol-4-phosphate 5-kinase, type I, alpha (PIP5K1A), mRNA [NM_003557]	**1.090**
TMED2	Homo sapiens transmembrane emp24 domain trafficking protein 2 (TMED2), mRNA	**1.090**

	[NM_006815]	
BTD	Homo sapiens biotinidase (BTD), mRNA [NM_000060]	**1.089**
MRFAP1L1	Homo sapiens Morf4 family associated protein 1-like 1 (MRFAP1L1),mRNA [NM_203462]	**1.089**
PTTG1IP	Homo sapiens pituitary tumor-transforming 1 interacting protein (PTTG1IP), mRNA [NM_004339]	**1.089**
LOC645693	Homo sapiens misc_RNA (LOC645693), miscRNA [XR_017498]	**1.086**
NUSAP1	Homo sapiens nucleolar and spindle associated protein 1 (NUSAP1), mRNA [NM_016359]	**1.086**
RPL35	Homo sapiens ribosomal protein L35 (RPL35), mRNA [NM_007209]	**1.085**
COMMD7	Homo sapiens COMM domain containing 7 (COMMD7), mRNA [NM_053041]	**1.085**
MGAT1	Homo sapiens mannosyl (alpha-1,3-)-glycoprotein beta-1,2-N-acetylglucosaminyltransferase (MGAT1), mRNA [NM_002406]	**1.083**
BSCL2	Homo sapiens Bernardinelli-Seip congenital lipodystrophy 2 (seipin) (BSCL2), mRNA [NM_032667]	**1.081**
C12orf52	Homo sapiens chromosome 12 open reading frame 52 (C12orf52), mRNA [NM_032848]	**1.081**
BCL2L13	Homo sapiens BCL2-like 13 (apoptosis facilitator) (BCL2L13), mRNA [NM_015367]	**1.080**
APEH	Homo sapiens N-acylaminoacyl-peptide hydrolase (APEH), mRNA [NM_001640]	**1.080**
ATP6AP1	Homo sapiens ATPase, H+ transporting, lysosomal accessory protein 1 (ATP6AP1), mRNA [NM_001183]	**1.080**
C6orf145	Homo sapiens chromosome 6 open reading frame 145 (C6orf145), mRNA [NM_183373]	**1.080**
MYO1E	Homo sapiens myosin IE (MYO1E), mRNA [NM_004998]	**1.080**
PACS2	Homo sapiens phosphofurin acidic cluster sorting protein 2 (PACS2), mRNA [NM_015197]	**1.080**
PQBP1	Homo sapiens polyglutamine binding protein 1 (PQBP1), mRNA [NM_005710]	**1.080**
SOX9	Homo sapiens SRY (sex determining region Y)-box 9 (SOX9), mRNA [NM_000346]	**1.079**
SNRNP40	Homo sapiens small nuclear ribonucleoprotein 40kDa (U5) (SNRNP40), mRNA [NM_004814]	**1.079**
KIAA1659	Homo sapiens mRNA for KIAA1659 protein, partial cds. [AB051446]	**1.078**
C19orf21	Homo sapiens chromosome 19 open reading frame 21 (C19orf21), mRNA [NM_173481]	**1.077**
TUBB2A	Homo sapiens tubulin, beta 2A (TUBB2A), mRNA [NM_001069]	**1.077**
PPP2R3B	Homo sapiens protein phosphatase 2 (formerly 2A), regulatory subunit B", beta (PPP2R3B), mRNA [NM_013239]	**1.076**
ROR2	Homo sapiens receptor tyrosine kinase-like orphan receptor 2 (ROR2), mRNA [NM_004560]	**1.076**
TTC38	Homo sapiens tetratricopeptide repeat domain 38 (TTC38), mRNA [NM_017931]	**1.076**
NME6	Homo sapiens non-metastatic cells 6, protein expressed in (nucleoside-diphosphate kinase) (NME6), mRNA [NM_005793]	**1.075**
EEF1D	Homo sapiens eukaryotic translation elongation factor 1 delta (guanine nucleotide exchange protein) (EEF1D), mRNA [NM_032378]	**1.074**
ARFGAP1	Homo sapiens ADP-ribosylation factor GTPase activating protein 1 (ARFGAP1), mRNA [NM_175609]	**1.073**
PRKCDBP	Homo sapiens protein kinase C, delta binding protein (PRKCDBP), mRNA	**1.073**

	[NM_145040]	
ABCC10	Homo sapiens ATP-binding cassette, sub-family C (CFTR/MRP), member 10 (ABCC10), mRNA [NM_033450]	**1.072**
DOK4	Homo sapiens docking protein 4 (DOK4), mRNA [NM_018110]	**1.072**
MUC1	Homo sapiens mucin 1, cell surface associated (MUC1), mRNA [NM_002456]	**1.072**
NLGN2	Homo sapiens neuroligin 2 (NLGN2), mRNA [NM_020795]	**1.072**
IMPDH1	Homo sapiens IMP (inosine monophosphate) dehydrogenase 1 (IMPDH1), mRNA [NM_000883]	**1.072**
KRT14	Homo sapiens keratin 14 (KRT14), mRNA [NM_000526]	**1.072**
CDK2	Homo sapiens cyclin-dependent kinase 2 (CDK2), mRNA [NM_001798]	**1.071**
INO80	Homo sapiens INO80 homolog (S. cerevisiae) (INO80), mRNA [NM_017553]	**1.071**
TMEM79	Homo sapiens transmembrane protein 79 (TMEM79), mRNA [NM_032323]	**1.071**
MLF2	Homo sapiens myeloid leukemia factor 2 (MLF2), mRNA [NM_005439]	**1.069**
FAH	Homo sapiens fumarylacetoacetate hydrolase (fumarylacetoacetase) (FAH), mRNA [NM_000137]	**1.068**
GNPDA1	Homo sapiens glucosamine-6-phosphate deaminase 1 (GNPDA1), mRNA [NM_005471]	**1.068**
CDC25A	Homo sapiens cell division cycle 25 homolog A (S. pombe) (CDC25A), mRNA [NM_001789]	**1.068**
DUSP2	Homo sapiens dual specificity phosphatase 2 (DUSP2), mRNA [NM_004418]	**1.068**
ERGIC3	Homo sapiens ERGIC and golgi 3 (ERGIC3), mRNA [NM_015966]	**1.068**
USP40	Homo sapiens ubiquitin specific peptidase 40 (USP40), mRNA [NM_018218]	**1.068**
TNFRSF6B	Homo sapiens tumor necrosis factor receptor superfamily, member 6b, decoy (TNFRSF6B), mRNA [NM_032945]	**1.067**
ZNF513	Homo sapiens zinc finger protein 513 (ZNF513), mRNA [NM_144631]	**1.067**
FMNL1	Homo sapiens formin-like 1 (FMNL1), mRNA [NM_005892]	**1.065**
STAG3L2	Homo sapiens stromal antigen 3-like 2 (STAG3L2), mRNA [NM_001025202]	**1.065**
DDX17	Homo sapiens DEAD (Asp-Glu-Ala-Asp) box polypeptide 17 (DDX17), mRNA [NM_006386]	**1.065**
ETS2	Homo sapiens v-ets erythroblastosis virus E26 oncogene homolog 2 (avian) (ETS2), mRNA [NM_005239]	**1.064**
CRYBB2P1	Homo sapiens crystallin, beta B2 pseudogene 1, mRNA (cDNA clone IMAGE:5288881). [BC047380]	**1.064**
GADD45GIP1	Homo sapiens growth arrest and DNA-damage-inducible, gamma interacting protein 1 (GADD45GIP1), mRNA [NM_052850]	**1.064**
LOC728428	Homo sapiens similar to 40S ribosomal protein S15 (RIG protein) (LOC728428), mRNA [XR_015753]	**1.064**
SNURF	Homo sapiens SNRPN upstream reading frame (SNURF), mRNA [NM_022804]	**1.064**
IPO8	Homo sapiens importin 8 (IPO8), mRNA [NM_006390]	**1.063**
PDIA6	Homo sapiens protein disulfide isomerase family A, member 6 (PDIA6), mRNA [NM_005742]	**1.063**
ZNF668	Homo sapiens zinc finger protein 668 (ZNF668), mRNA [NM_024706]	**1.063**
DUSP15	Homo sapiens dual specificity phosphatase 15 (DUSP15), mRNA [NM_080611]	**1.062**
FN1	Homo sapiens fibronectin 1 (FN1), mRNA [NM_212482]	**1.062**

RAF1	Homo sapiens v-raf-1 murine leukemia viral oncogene homolog 1 (RAF1), mRNA [NM_002880]	**1.062**
TAPBP	Homo sapiens TAP binding protein (tapasin) (TAPBP), mRNA [NM_172208]	**1.061**
CCDC101	Homo sapiens coiled-coil domain containing 101 (CCDC101), mRNA [NM_138414]	**1.060**
PAQR4	Homo sapiens progestin and adipoQ receptor family member IV (PAQR4), mRNA [NM_152341]	**1.060**
SIRT7	Homo sapiens sirtuin (silent mating type information regulation 2 homolog) 7 (S. cerevisiae) (SIRT7), mRNA [NM_016538]	**1.060**
COQ10B	Homo sapiens coenzyme Q10 homolog B (S. cerevisiae) (COQ10B), mRNA [NM_025147]	**1.059**
SAPS3	Homo sapiens SAPS domain family, member 3 (SAPS3), mRNA [NM_018312]	**1.059**
SLC35A4	Homo sapiens solute carrier family 35, member A4 (SLC35A4), mRNA [NM_080670]	**1.059**
VPS33B	Homo sapiens vacuolar protein sorting 33 homolog B (yeast) (VPS33B), mRNA [NM_018668]	**1.059**
MAST4	Homo sapiens microtubule associated serine/threonine kinase family member 4 (MAST4), mRNA [NM_015183]	**1.059**
RSF1	Homo sapiens remodeling and spacing factor 1 (RSF1), mRNA [NM_016578]	**1.057**
TGFA	Homo sapiens transforming growth factor, alpha (TGFA), mRNA [NM_003236]	**1.057**
ATPAF2	Homo sapiens ATP synthase mitochondrial F1 complex assembly factor 2 (ATPAF2), mRNA [NM_145691]	**1.056**
AURKB	Homo sapiens aurora kinase B (AURKB), mRNA [NM_004217]	**1.056**
SUMF2	Homo sapiens sulfatase modifying factor 2 (SUMF2), mRNA [NM_001042468]	**1.056**
HADHA	Homo sapiens hydroxyacyl-Coenzyme A dehydrogenase/3-ketoacyl-Coenzyme A thiolase/enoyl-Coenzyme A hydratase (trifunctional protein), alpha subunit (HADHA), mRNA [NM_000182]	**1.056**
M6PR	Homo sapiens mannose-6-phosphate receptor (cation dependent) (M6PR), mRNA [NM_002355]	**1.055**
DICER1	Homo sapiens dicer 1, ribonuclease type III (DICER1), mRNA [NM_177438]	**1.055**
PREB	Homo sapiens prolactin regulatory element binding (PREB), mRNA [NM_013388]	**1.055**
AIM1L	Homo sapiens absent in melanoma 1-like (AIM1L), mRNA [NM_001039775]	**1.054**
NDUFB7	Homo sapiens NADH dehydrogenase (ubiquinone) 1 beta subcomplex, 7, 18kDa (NDUFB7), mRNA [NM_004146]	**1.054**
C22orf13	Homo sapiens chromosome 22 open reading frame 13 (C22orf13), mRNA [NM_031444]	**1.054**
METTL11A	Homo sapiens methyltransferase like 11A (METTL11A), mRNA [NM_014064]	**1.053**
RBM10	Homo sapiens RNA binding motif protein 10 (RBM10), mRNA [NM_005676]	**1.053**
CA5BL	Homo sapiens carbonic anhydrase VB-like (CA5BL), non-coding RNA [NR_026551]	**1.052**
LOC440894	Homo sapiens cDNA FLJ31522 fis, clone NT2RI2000270. [AK056084]	**1.052**
SP3P	Homo sapiens Sp3 transcription factor pseudogene, mRNA (cDNA clone IMAGE:5266022). [BC036697]	**1.052**
TPX2	Homo sapiens TPX2, microtubule-associated, homolog (Xenopus laevis) (TPX2), mRNA [NM_012112]	**1.052**
CDC2L1	Homo sapiens cell division cycle 2-like 1 (PITSLRE proteins) (CDC2L1), mRNA [NM_033489]	**1.051**

DCTN5	Homo sapiens dynactin 5 (p25) (DCTN5), mRNA [NM_032486]	**1.051**
EEFSEC	Homo sapiens eukaryotic elongation factor, selenocysteine-tRNA-specific (EEFSEC), mRNA [NM_021937]	**1.051**
SAFB2	Homo sapiens scaffold attachment factor B2 (SAFB2), mRNA [NM_014649]	**1.051**
CGN	Homo sapiens cingulin (CGN), mRNA [NM_020770]	**1.050**
LOC200810	Homo sapiens beta-1,4-mannosyltransferase-like (LOC200810), mRNA [NM_001015050]	**1.050**
CHST3	Homo sapiens carbohydrate (chondroitin 6) sulfotransferase 3 (CHST3), mRNA [NM_004273]	**1.049**
HLA-F	Homo sapiens major histocompatibility complex, class I, F (HLA-F), mRNA [NM_018950]	**1.049**
SUPT6H	Homo sapiens suppressor of Ty 6 homolog (S. cerevisiae) (SUPT6H), mRNA [NM_003170]	**1.049**
C16orf67	Homo sapiens chromosome 16 open reading frame 67 (C16orf67), non-coding RNA [NR_024034]	**1.048**
EML2	Homo sapiens echinoderm microtubule associated protein like 2 (EML2), mRNA [NM_012155]	**1.048**
IGF2R	Homo sapiens insulin-like growth factor 2 receptor (IGF2R), mRNA [NM_000876]	**1.048**
MAPKAPK3	Homo sapiens mitogen-activated protein kinase-activated protein kinase 3 (MAPKAPK3), mRNA [NM_004635]	**1.048**
ADPRHL2	Homo sapiens ADP-ribosylhydrolase like 2 (ADPRHL2), mRNA [NM_017825]	**1.047**
C19orf22	Homo sapiens chromosome 19 open reading frame 22 (C19orf22), mRNA [NM_138774]	**1.047**
EMD	Homo sapiens emerin (EMD), mRNA [NM_000117]	**1.047**
LIMK2	Homo sapiens LIM domain kinase 2 (LIMK2), mRNA [NM_001031801]	**1.047**
VPS25	Homo sapiens vacuolar protein sorting 25 homolog (S. cerevisiae) (VPS25), mRNA [NM_032353]	**1.047**
C19orf70	Homo sapiens chromosome 19 open reading frame 70 (C19orf70), mRNA [NM_205767]	**1.046**
MAPK3	Homo sapiens mitogen-activated protein kinase 3 (MAPK3), mRNA [NM_002746]	**1.046**
ZBTB48	Homo sapiens zinc finger and BTB domain containing 48 (ZBTB48), mRNA [NM_005341]	**1.046**
MGC34796	Homo sapiens SPR pseudogene, mRNA (cDNA clone IMAGE:5166892). [BC034822]	**1.045**
UBQLN4	Homo sapiens ubiquilin 4 (UBQLN4), mRNA [NM_020131]	**1.045**
GPRC5A	Homo sapiens G protein-coupled receptor, family C, group 5, member A (GPRC5A), mRNA [NM_003979]	**1.045**
JUNB	Homo sapiens jun B proto-oncogene (JUNB), mRNA [NM_002229]	**1.045**
ZNF644	Homo sapiens zinc finger protein 644 (ZNF644), mRNA [NM_201269]	**1.045**
KIF3A	Homo sapiens kinesin family member 3A (KIF3A), mRNA [NM_007054]	**1.043**
LOC100128893	Homo sapiens cDNA FLJ41084 fis, clone ADRGL2010974. [AK123079]	**1.043**
TOMM22	Homo sapiens translocase of outer mitochondrial membrane 22 homolog (yeast) (TOMM22), mRNA [NM_020243]	**1.043**
FAM44C	Homo sapiens family with sequence similarity 44, member C, mRNA (cDNA clone IMAGE:4825530). [BC021740]	**1.041**
PACSIN2	Homo sapiens mRNA; cDNA DKFZp434H1130 (from clone DKFZp434H1130).	**1.041**

	[AL136845]	
HARS	Homo sapiens histidyl-tRNA synthetase (HARS), mRNA [NM_002109]	**1.041**
LOC728126	Homo sapiens misc_RNA (LOC728126), miscRNA [XR_036919]	**1.040**
TSPAN9	Homo sapiens tetraspanin 9 (TSPAN9), mRNA [NM_006675]	**1.040**
MBTPS1	Homo sapiens membrane-bound transcription factor peptidase, site 1 (MBTPS1), mRNA [NM_003791]	**1.039**
RHOC	Homo sapiens ras homolog gene family, member C (RHOC), mRNA [NM_175744]	**1.038**
TPRG1L	Homo sapiens tumor protein p63 regulated 1-like (TPRG1L), mRNA [NM_182752]	**1.038**
MYD88	Homo sapiens myeloid differentiation primary response gene (88) (MYD88), mRNA [NM_002468]	**1.038**
C3orf37	Homo sapiens chromosome 3 open reading frame 37 (C3orf37), mRNA [NM_001006109]	**1.037**
LRRC47	Homo sapiens leucine rich repeat containing 47 (LRRC47), mRNA [NM_020710]	**1.037**
WDR74	Homo sapiens WD repeat domain 74 (WDR74), mRNA [NM_018093]	**1.037**
AOF2	Homo sapiens amine oxidase (flavin containing) domain 2 (AOF2), mRNA [NM_015013]	**1.036**
KARS	Homo sapiens lysyl-tRNA synthetase (KARS), mRNA [NM_005548]	**1.035**
ATP1A1	Homo sapiens ATPase, Na+/K+ transporting, alpha 1 polypeptide (ATP1A1), mRNA [NM_000701]	**1.034**
GLT25D1	Homo sapiens glycosyltransferase 25 domain containing 1 (GLT25D1), mRNA [NM_024656]	**1.034**
LGMN	Homo sapiens legumain (LGMN), mRNA [NM_001008530]	**1.034**
LPPR2	Homo sapiens lipid phosphate phosphatase-related protein type 2 (LPPR2), mRNA [NM_022737]	**1.034**
FTH1	Homo sapiens ferritin, heavy polypeptide 1 (FTH1), mRNA [NM_002032]	**1.033**
HDAC3	Homo sapiens histone deacetylase 3 (HDAC3), mRNA [NM_003883]	**1.033**
LOC728264	Homo sapiens mRNA; cDNA DKFZp586C0721 (from clone DKFZp586C0721). [AL137734]	**1.033**
LOC729090	Homo sapiens similar to Eukaryotic translation elongation factor 1 alpha 1 (LOC729090), mRNA [XR_015449]	**1.033**
NSUN5B	Homo sapiens NOL1/NOP2/Sun domain family, member 5B (NSUN5B), mRNA [NM_001039575]	**1.032**
RIN1	Homo sapiens Ras and Rab interactor 1 (RIN1), mRNA [NM_004292]	**1.032**
ACTG1	Homo sapiens actin, gamma 1 (ACTG1), mRNA [NM_001614]	**1.031**
RPL18	Homo sapiens ribosomal protein L18 (RPL18), mRNA [NM_000979]	**1.031**
DALRD3	Homo sapiens DALR anticodon binding domain containing 3 (DALRD3), mRNA [NM_018114]	**1.031**
SETD5	Homo sapiens SET domain containing 5 (SETD5), mRNA [NM_001080517]	**1.031**
C11orf80	Homo sapiens chromosome 11 open reading frame 80 (C11orf80), mRNA [NM_024650]	**1.029**
C12orf10	Homo sapiens chromosome 12 open reading frame 10 (C12orf10), mRNA [NM_021640]	**1.029**
EXOC2	Homo sapiens exocyst complex component 2 (EXOC2), mRNA [NM_018303]	**1.029**
ITGB5	Homo sapiens integrin, beta 5 (ITGB5), mRNA [NM_002213]	**1.029**
OAZ2	Homo sapiens ornithine decarboxylase antizyme 2 (OAZ2), mRNA [NM_002537]	**1.028**

PRDX2	Homo sapiens peroxiredoxin 2 (PRDX2), mRNA [NM_005809]	**1.028**
P53AIP1	Homo sapiens mRNA for p53AIP1beta, complete cds. [AB045831]	**1.026**
MUL1	Homo sapiens mitochondrial E3 ubiquitin ligase 1 (MUL1), mRNA [NM_024544]	**1.025**
ALKBH7	Homo sapiens alkB, alkylation repair homolog 7 (E. coli) (ALKBH7), mRNA [NM_032306]	**1.024**
ACOT8	Homo sapiens acyl-CoA thioesterase 8 (ACOT8), mRNA [NM_005469]	**1.023**
C11orf59	Homo sapiens chromosome 11 open reading frame 59 (C11orf59), mRNA [NM_017907]	**1.022**
GBA2	Homo sapiens glucosidase, beta (bile acid) 2 (GBA2), mRNA [NM_020944]	**1.022**
HIF1AN	Homo sapiens hypoxia inducible factor 1, alpha subunit inhibitor (HIF1AN), mRNA [NM_017902]	**1.022**
PHC2	Homo sapiens polyhomeotic homolog 2 (Drosophila) (PHC2), mRNA [NM_198040]	**1.022**
SLC5A6	Homo sapiens solute carrier family 5 , member 6 (SLC5A6), mRNA [NM_021095]	**1.022**
SSBP2	Homo sapiens single-stranded DNA binding protein 2 (SSBP2), mRNA [NM_012446]	**1.022**
SEPX1	Homo sapiens selenoprotein X, 1 (SEPX1), mRNA [NM_016332]	**1.022**
ABCB8	Homo sapiens ATP-binding cassette, sub-family B (MDR/TAP), member 8 (ABCB8), nuclear gene encoding mitochondrial protein, mRNA [NM_007188]	**1.021**
CNNM3	Homo sapiens cyclin M3 (CNNM3), mRNA [NM_017623]	**1.021**
TNPO2	Homo sapiens transportin 2 (importin 3, karyopherin beta 2b) (TNPO2), mRNA [NM_013433]	**1.021**
RHOF	Homo sapiens ras homolog gene family, member F (in filopodia) (RHOF), mRNA [NM_019034]	**1.021**
ATP13A1	Homo sapiens ATPase type 13A1 (ATP13A1), mRNA [NM_020410]	**1.020**
MCM4	Homo sapiens minichromosome maintenance complex component 4 (MCM4), mRNA [NM_005914]	**1.020**
PIK3C2A	Homo sapiens phosphoinositide-3-kinase, class 2, alpha polypeptide (PIK3C2A), mRNA [NM_002645]	**1.020**
SFPQ	Homo sapiens splicing factor proline/glutamine-rich (polypyrimidine tract binding protein associated) (SFPQ), mRNA [NM_005066]	**1.020**
ADCK4	Homo sapiens aarF domain containing kinase 4 (ADCK4), mRNA [NM_024876]	**1.019**
ADCK1	Homo sapiens aarF domain containing kinase 1 (ADCK1), mRNA [NM_020421]	**1.019**
KLHL26	Homo sapiens kelch-like 26 (Drosophila) (KLHL26), mRNA [NM_018316]	**1.019**
LBX1	H.sapiens mRNA for transcription factor, Lbx1. [X90828]	**1.019**
RRP7B	Homo sapiens ribosomal RNA processing 7 homolog B (S. cerevisiae) (RRP7B), non-coding RNA [NR_002184]	**1.019**
AKR1B1	Homo sapiens aldo-keto reductase family 1, member B1 (aldose reductase) (AKR1B1), mRNA [NM_001628]	**1.018**
CTBP2	Homo sapiens C-terminal binding protein 2 (CTBP2), mRNA [NM_001329]	**1.018**
GBA	Homo sapiens glucosidase, beta; acid (includes glucosylceramidase) (GBA),rmRNA [NM_001005749]	**1.018**
MET	Homo sapiens met proto-oncogene (hepatocyte growth factor receptor) (MET), mRNA [NM_000245]	**1.017**
CNP	Homo sapiens 2',3'-cyclic nucleotide 3' phosphodiesterase (CNP), mRNA [NM_033133]	**1.017**
TAGLN2	Homo sapiens transgelin 2 (TAGLN2), mRNA [NM_003564]	**1.017**

ALS2CL	Homo sapiens ALS2 C-terminal like (ALS2CL), mRNA [NM_147129]	**1.017**
FHL1	Homo sapiens four and a half LIM domains 1 (FHL1), mRNA [NM_001449]	**1.016**
LOC729234	Homo sapiens, clone IMAGE:3849975, mRNA, [BC015216]	**1.016**
RAB26	Homo sapiens RAB26, member RAS oncogene family (RAB26), mRNA [NM_014353]	**1.016**
SLC9A7	Homo sapiens solute carrier family 9 (sodium/hydrogen exchanger), member 7 (SLC9A7), mRNA [NM_032591]	**1.016**
SMARCA2	Homo sapiens SWI/SNF related, matrix associated, subfamily a, member 2 (SMARCA2), mRNA [NM_139045]	**1.016**
TNK2	Homo sapiens tyrosine kinase, non-receptor, 2 (TNK2), mRNA [NM_001010938]	**1.016**
ZER1	Homo sapiens zer-1 homolog (C. elegans) (ZER1), mRNA [NM_006336]	**1.016**
SNTA1	Homo sapiens syntrophin, alpha 1 (dystrophin-associated protein A1, 59kDa, acidic component) (SNTA1), mRNA [NM_003098]	**1.016**
ANO10	Homo sapiens anoctamin 10 (ANO10), mRNA [NM_018075]	**1.015**
C20orf27	Homo sapiens chromosome 20 open reading frame 27 (C20orf27), mRNA [NM_001039140]	**1.015**
POLDIP3	Homo sapiens polymerase (DNA-directed), delta interacting protein 3 (POLDIP3), mRNA [NM_032311]	**1.015**
ILF3	Homo sapiens interleukin enhancer binding factor 3, 90kDa (ILF3), mRNA [NM_012218]	**1.014**
COBL	Homo sapiens cordon-bleu homolog (mouse) (COBL), mRNA [NM_015198]	**1.014**
GANAB	Homo sapiens glucosidase, alpha; neutral AB (GANAB), mRNA [NM_198335]	**1.014**
PSMG3	Homo sapiens proteasome (prosome, macropain) assembly chaperone 3 (PSMG3), mRNA [NM_032302]	**1.013**
B4GALT2	Homo sapiens UDP-Gal:betaGlcNAc beta 1,4- galactosyltransferase, polypeptide 2 (B4GALT2), mRNA [NM_003780]	**1.013**
GGTLC3	Gamma-glutamyltranspeptidase 1 Precursor (EC 2.3.2.2) [Source:UniProtKB/ Swiss-Prot; Acc:P19440] [ENST00000404223]	**1.012**
GPR39	Homo sapiens G protein-coupled receptor 39 (GPR39), mRNA [NM_001508]	**1.012**
TSPAN17	Homo sapiens tetraspanin 17 (TSPAN17), mRNA [NM_012171]	**1.012**
INPP5J	Homo sapiens inositol polyphosphate-5-phosphatase J (INPP5J), mRNA [NM_001002837]	**1.011**
ITGA3	Homo sapiens integrin, alpha 3 (antigen CD49C, alpha 3 subunit of VLA-3 receptor) (ITGA3), mRNA [NM_002204]	**1.011**
TMEM31	Homo sapiens transmembrane protein 31 (TMEM31), mRNA [NM_182541]	**1.011**
SRF	Homo sapiens serum response factor (c-fos serum response element-binding transcription factor) (SRF), mRNA [NM_003131]	**1.010**
ALDOA	Homo sapiens aldolase A, fructose-bisphosphate (ALDOA), mRNA [NM_184041]	**1.010**
LOC100128295	Homo sapiens similar to hCG1639947 (LOC100128295), mRNA [XM_001720544]	**1.009**
LOC100130744	Homo sapiens clone pp7583 unknown mRNA. [AF289590]	**1.009**
PHF16	Homo sapiens PHD finger protein 16 (PHF16), mRNA [NM_014735]	**1.009**
SLC25A10	Homo sapiens solute carrier family 25 (mitochondrial carrier; dicarboxylate transporter), member 10 (SLC25A10), mRNA [NM_012140]	**1.009**
UBTF	Homo sapiens upstream binding transcription factor, RNA polymerase I (UBTF), mRNA	**1.009**

	[NM_001076683]	
KLHL35	Homo sapiens kelch-like 35 (Drosophila) (KLHL35), mRNA [NM_001039548]	**1.008**
LOC389033	Homo sapiens hypothetical LOC389033 (LOC389033), mRNA [XM_374010]	**1.008**
NUMBL	Homo sapiens numb homolog (Drosophila)-like (NUMBL), mRNA [NM_004756]	**1.008**
TRMT2A	Homo sapiens TRM2 tRNA methyltransferase 2 homolog A (S. cerevisiae) (TRMT2A), mRNA [NM_022727]	**1.008**
UNG	Homo sapiens uracil-DNA glycosylase (UNG), mRNA [NM_003362]	**1.008**
DDA1	Homo sapiens DET1 and DDB1 associated 1 (DDA1), mRNA [NM_024050]	**1.007**
COMMD5	Homo sapiens COMM domain containing 5 (COMMD5), mRNA [NM_014066]	**1.007**
KIAA0427	Homo sapiens KIAA0427 (KIAA0427), mRNA [NM_014772]	**1.007**
LEPREL2	Homo sapiens leprecan-like 2 (LEPREL2), mRNA [NM_014262]	**1.007**
ARHGEF11	Homo sapiens Rho guanine nucleotide exchange factor (GEF) 11 (ARHGEF11), mRNA [NM_198236]	**1.006**
RCC2	Homo sapiens regulator of chromosome condensation 2 (RCC2), mRNA [NM_018715]	**1.006**
MAP7D1	Homo sapiens MAP7 domain containing 1 (MAP7D1), mRNA [NM_018067]	**1.005**
MTCH1	Homo sapiens mitochondrial carrier homolog 1 (C. elegans) (MTCH1), mRNA [NM_014341]	**1.005**
TPM3	Homo sapiens tropomyosin 3 (TPM3), mRNA [NM_001043352]	**1.004**
LOC554202	Homo sapiens hypothetical LOC554202, mRNA (cDNA clone MGC:30053 IMAGE:5139119), [BC021861]	**1.004**
LPAR5	Homo sapiens lysophosphatidic acid receptor 5 (LPAR5), mRNA [NM_020400]	**1.004**
AP2A1	Homo sapiens adaptor-related protein complex 2, alpha 1 subunit (AP2A1), tmRNA [NM_014203]	**1.003**
MED12	Homo sapiens mediator complex subunit 12 (MED12), mRNA [NM_005120]	**1.003**
ANAPC2	Homo sapiens anaphase promoting complex subunit 2 (ANAPC2), mRNA [NM_013366]	**1.002**
ANXA6	Homo sapiens annexin A6 (ANXA6), mRNA [NM_001155]	**1.002**
RABEP2	Homo sapiens rabaptin, RAB GTPase binding effector protein 2 (RABEP2), mRNA [NM_024816]	**1.002**
UBE2Z	Homo sapiens ubiquitin-conjugating enzyme E2Z (UBE2Z), mRNA [NM_023079]	**1.002**
AKT2	Homo sapiens v-akt murine thymoma viral oncogene homolog 2 (AKT2), mRNA [NM_001626]	**1.002**
HNRNPA2B1	Homo sapiens heterogeneous nuclear ribonucleoprotein A2/B1 (HNRNPA2B1), tmRNA [NM_002137]	**1.001**
HRAS	Homo sapiens v-Ha-ras Harvey rat sarcoma viral oncogene homolog (HRAS), mRNA [NM_005343]	**1.000**
