# Supplementary material for: Genotoxicity and molecular response of silver nanoparticle (NP)-based hydrogel
Source: J Nanobiotechnology. 2012 May 1;10:16. doi: 10.1186/1477-3155-10-16 (PMC3430588; doi:10.1186/1477-3155-10-16)
Supplement: Additional file 6 — Down-regulated genes in cells exposed to Hydrogel for 24 h. Fold-change is logarithmic ratio (log2 ratio) to expression level in control. [file 1477-3155-10-16-S6.pdf]

Additional File 6. Down-regulated genes in cells exposed to Hydrogel for 24 h. Fold-change is logarithmic ratio (log_2 ratio) to expression level in control.

**GeneName**	**Description**	**Fold-change (log2 ratio)**
NOX1	Homo sapiens NADPH oxidase 1 (NOX1), transcript variant NOH-1L, mRNA [NM_007052]	**-2.468**
LOC100128519	Homo sapiens misc_RNA (LOC100128519), miscRNA [XR_038473]	**-2.415**
C18orf56	Homo sapiens chromosome 18 open reading frame 56 (C18orf56), mRNA [NM_001012716]	**-2.374**
IKZF4	Homo sapiens IKAROS family zinc finger 4 (Eos) (IKZF4), mRNA [NM_022465]	**-2.320**
F13A1	Homo sapiens coagulation factor XIII, A1 polypeptide (F13A1), mRNA [NM_000129]	**-2.294**
LOC388889	Homo sapiens cDNA: FLJ22849 fis, clone KAIA987. [AK026502]	**-2.289**
LOC392335	Homo sapiens misc_RNA (LOC392335), miscRNA [XR_037043]	**-2.242**
USP11	Homo sapiens ubiquitin specific peptidase 11 (USP11), mRNA [NM_004651]	**-2.228**
CLEC14A	Homo sapiens C-type lectin domain family 14, member A (CLEC14A), mRNA [NM_175060]	**-2.212**
TMEM154	Homo sapiens transmembrane protein 154 (TMEM154), mRNA [NM_152680]	**-2.206**
LOC653071	Homo sapiens similar to CG32820-PA, isoform A, mRNA (cDNA clone IMAGE:4812880) [BC068588]	**-2.200**
CDC14A	Homo sapiens CDC14 cell division cycle 14 homolog A (S. cerevisiae) (CDC14A), mRNA [NM_003672]	**-2.193**
C20orf71	Homo sapiens chromosome 20 open reading frame 71 (C20orf71), mRNA [NM_178466]	**-2.166**
LOC100133154	Homo sapiens hypothetical protein LOC100133154 (LOC100133154), mRNA [XM_001714925]	**-2.118**
LOC344178	Homo sapiens similar to hCG1794703 (LOC344178), mRNA [XM_001721796]	**-2.104**
ZNF319	Homo sapiens zinc finger protein 319 (ZNF319), mRNA [NM_020807]	**-2.097**
KCNS3	Homo sapiens potassium voltage-gated channel, delayed-rectifier, subfamily S, member 3 (KCNS3), mRNA [NM_002252]	**-2.087**
KCNH7	Homo sapiens potassium voltage-gated channel, subfamily H (eag-related), member 7 (KCNH7), mRNA [NM_033272]	**-2.077**
SPRR3	Homo sapiens small proline-rich protein 3 (SPRR3), mRNA [NM_005416]	**-2.055**
MTA2	Homo sapiens metastasis associated 1 family, member 2 (MTA2), mRNA [NM_004739]	**-2.038**
HOXC8	Homo sapiens homeobox C8 (HOXC8), mRNA [NM_022658]	**-2.029**
IL17RB	Homo sapiens interleukin 17 receptor B (IL17RB), mRNA [NM_018725]	**-2.019**
SAMD12	Homo sapiens cDNA clone HTBAYE05 5', mRNA sequence [AV724325]	**-2.018**
DOK3	Homo sapiens cDNA FLJ39939 fis, clone SPLEN2022227, moderately similar to Mus musculus adaptor protein (Dokl) mRNA. [AK097258]	**-1.974**
MGC3207	Homo sapiens translation initiation factor eIF-2B subunit alpha/beta/delta-like	**-1.973**

	protein (MGC3207), mRNA [NM_001031727]	
IGSF2	Homo sapiens immunoglobulin superfamily, member 2 (IGSF2), mRNA [NM_004258]	**-1.972**
KIAA0802	Homo sapiens KIAA0802 (KIAA0802), mRNA [NM_015210]	**-1.937**
CPB1	Homo sapiens carboxypeptidase B1 (tissue) (CPB1), mRNA [NM_001871]	**-1.935**
LOC100132439	Homo sapiens similar to Protein FAM27E3 (LOC100132439), mRNA [XM_001719283]	**-1.904**
PL-5283	Homo sapiens PL-5283 protein (PL-5283), mRNA [NM_001130929]	**-1.891**
HERC3	Homo sapiens hect domain and RLD 3, mRNA (cDNA clone IMAGE:6050308), complete cds. [BC038960]	**-1.885**
IRX3	Homo sapiens iroquois homeobox 3 (IRX3), mRNA [NM_024336]	**-1.880**
KLRC1	Homo sapiens killer cell lectin-like receptor subfamily C, member 1 (KLRC1), mRNA [NM_007328]	**-1.880**
C15orf62	Homo sapiens chromosome 15 open reading frame 62 (C15orf62), , mRNA [NM_001130448]	**-1.875**
AMY1C	Homo sapiens amylase, alpha 1C (salivary) (AMY1C), mRNA [NM_001008219]	**-1.863**
SPNS2	Homo sapiens spinster homolog 2 (Drosophila) (SPNS2), mRNA [NM_001124758]	**-1.846**
DAGLA	Homo sapiens diacylglycerol lipase, alpha (DAGLA), mRNA [NM_006133]	**-1.843**
LOC643684	Homo sapiens hypothetical LOC643684 (LOC643684), mRNA [XM_931745]	**-1.843**
BSPRY	Homo sapiens B-box and SPRY domain containing (BSPRY), mRNA [NM_017688]	**-1.833**
GOLGA8E	Homo sapiens golgi autoantigen, golgin subfamily a, 8E (GOLGA8E), mRNA [NM_001012423]	**-1.833**
ZNF423	Homo sapiens zinc finger protein 423 (ZNF423), mRNA [NM_015069]	**-1.831**
ACCN2	Homo sapiens amiloride-sensitive cation channel 2, neuronal (ACCN2), mRNA [NM_020039]	**-1.830**
LOC389992	Homo sapiens similar to hCG2040259 (LOC389992), mRNA [XM_001720568]	**-1.830**
PNPLA7	Homo sapiens cDNA FLJ44279 fis, clone TRACH2001549, [AK126267]	**-1.813**
SLC45A3	Homo sapiens solute carrier family 45, member 3 (SLC45A3), mRNA [NM_033102]	**-1.810**
NECAB1	Homo sapiens N-terminal EF-hand calcium binding protein 1 (NECAB1), mRNA [NM_022351]	**-1.808**
TREML2	Homo sapiens triggering receptor expressed on myeloid cells-like 2 (TREML2), mRNA [NM_024807]	**-1.807**
SLAMF6	Homo sapiens SLAM family member 6 (SLAMF6), mRNA [NM_052931]	**-1.798**
NUDT12	Homo sapiens nudix (nucleoside diphosphate linked moiety X)-type motif 12 (NUDT12), mRNA [NM_031438]	**-1.785**
ITGA11	Homo sapiens integrin, alpha 11 (ITGA11), mRNA [NM_001004439]	**-1.767**
MAGEA1	Homo sapiens melanoma antigen family A, 1 (directs expression of antigen MZ2-E) (MAGEA1), mRNA [NM_004988]	**-1.755**
KIAA1267	Homo sapiens KIAA1267 (KIAA1267), mRNA [NM_015443]	**-1.753**

F7	Homo sapiens coagulation factor VII (serum prothrombin conversion accelerator) (F7), mRNA [NM_000131]	**-1.744**
DEFB129	Homo sapiens defensin, beta 129 (DEFB129), mRNA [NM_080831]	**-1.738**
DSCR8	Homo sapiens mRNA for malignant melanoma associated protein (MMA-1 gene), splice variant MMA-1d. [AJ783421]	**-1.733**
SOCS2	Homo sapiens suppressor of cytokine signaling 2 (SOCS2), mRNA [NM_003877]	**-1.733**
SPINK4	Homo sapiens serine peptidase inhibitor, Kazal type 4 (SPINK4), mRNA [NM_014471]	**-1.730**
ESAM	Homo sapiens endothelial cell adhesion molecule (ESAM), mRNA [NM_138961]	**-1.723**
ZNF594	Homo sapiens zinc finger protein 594 (ZNF594), mRNA [NM_032530]	**-1.720**
CCDC61	Homo sapiens coiled-coil domain containing 61 (CCDC61), mRNA [NM_001080402]	**-1.718**
LOC199800	Homo sapiens hypothetical protein LOC199800 (LOC199800), mRNA [NM_001101340]	**-1.716**
KLRC3	Homo sapiens killer cell lectin-like receptor subfamily C, member 3 (KLRC3), mRNA [NM_007333]	**-1.714**
SNHG10	Homo sapiens small nucleolar RNA host gene 10 (non-protein coding) (SNHG10), non-coding RNA [NR_003138]	**-1.710**
SLC5A12	Homo sapiens solute carrier family 5 (sodium/glucose cotransporter), member 12 (SLC5A12), mRNA [NM_178498]	**-1.693**
CSAG2	Homo sapiens CSAG family, member 2 (CSAG2), mRNA [NM_001080848]	**-1.691**
S1PR3	Homo sapiens sphingosine-1-phosphate receptor 3 (S1PR3), mRNA [NM_005226]	**-1.684**
SPHAR	Homo sapiens S-phase response (cyclin-related) (SPHAR), mRNA [NM_006542]	**-1.681**
TMC8	Homo sapiens transmembrane channel-like 8 (TMC8), mRNA [NM_152468]	**-1.680**
LAMP3	Homo sapiens lysosomal-associated membrane protein 3 (LAMP3), mRNA [NM_014398]	**-1.679**
C1orf198	Homo sapiens chromosome 1 open reading frame 198 (C1orf198), mRNA [NM_032800]	**-1.677**
FAM154A	Homo sapiens family with sequence similarity 154, member A (FAM154A), mRNA [NM_153707]	**-1.676**
C20orf85	Homo sapiens chromosome 20 open reading frame 85 (C20orf85), mRNA [NM_178456]	**-1.674**
C22orf27	Homo sapiens cDNA FLJ35801 fis, clone TESTI2005937. [AK093120]	**-1.670**
KIAA0492	Homo sapiens mRNA, chromosome 1 specific transcript KIAA0492. [AB007961]	**-1.669**
MYO5C	Homo sapiens myosin VC (MYO5C), mRNA [NM_018728]	**-1.668**
OR7E47P	Homo sapiens olfactory receptor, family 7, subfamily E, member 47 pseudogene, mRNA (cDNA clone IMAGE:5590288). [BC042060]	**-1.668**
NCRNA00115	Homo sapiens non-protein coding RNA 115 (NCRNA00115), non-coding RNA	**-1.665**

	[NR_024321]	
LFNG	Homo sapiens LFNG O-fucosylpeptide 3-beta-N-acetylglucosaminyltransferase (LFNG), mRNA [NM_001040167]	**-1.659**
STX1A	Homo sapiens syntaxin 1A (brain) (STX1A), mRNA [NM_004603]	**-1.657**
CFLP1	Homo sapiens cofilin pseudogene 1, mRNA (cDNA clone IMAGE:5168640). [BC031631]	**-1.654**
RP11-265F14.2	Homo sapiens elastase 2B (ELA2B), mRNA [NM_015849]	**-1.636**
KIAA1324	Homo sapiens clone DNA59770 AEPG2426 (UNQ2426) mRNA. [AY358366]	**-1.634**
CSMD3	Homo sapiens CUB and Sushi multiple domains 3 (CSMD3), mRNA [NM_198123]	**-1.625**
C17orf39	Homo sapiens chromosome 17 open reading frame 39 (C17orf39), mRNA [NM_024052]	**-1.616**
MYLC2PL	Myosin light chain 2, lymphocyte-specific (Precursor lymphocyte-specific regulatory light chain) [Source:UniProtKB/Swiss-Prot;Acc:Q9BUA6] [ENST00000223167]	**-1.614**
LOC728344	Homo sapiens misc_RNA (LOC728344), miscRNA [XR_018424]	**-1.613**
ZSCAN1	Homo sapiens zinc finger and SCAN domain containing 1 (ZSCAN1), mRNA [NM_182572]	**-1.613**
GAS7	Homo sapiens growth arrest-specific 7 (GAS7), mRNA [NM_201433]	**-1.602**
DDIT4L	Homo sapiens DNA-damage-inducible transcript 4-like (DDIT4L), mRNA [NM_145244]	**-1.596**
LOC388630	Homo sapiens hypothetical LOC388630 (LOC388630), mRNA [XM_371250]	**-1.589**
LOC791120	Homo sapiens hypothetical LOC791120 (LOC791120), non-coding RNA [NR_015357]	**-1.586**
EFCAB10	Homo sapiens cDNA clone IMAGE:6616931, partial cds. [BC062748]	**-1.581**
ZNF710	Homo sapiens mRNA for FLJ00306 protein. [AK160373]	**-1.578**
TMSB15A	Homo sapiens thymosin-like 8 (TMSL8), mRNA [NM_021992]	**-1.576**
COX4I2	Homo sapiens cytochrome c oxidase subunit IV isoform 2 (lung) (COX4I2), nuclear gene encoding mitochondrial protein, mRNA [NM_032609]	**-1.574**
RASD2	Homo sapiens RASD family, member 2 (RASD2), mRNA [NM_014310]	**-1.570**
C10orf95	Homo sapiens chromosome 10 open reading frame 95 (C10orf95), mRNA [NM_024886]	**-1.567**
ZNF780A	Homo sapiens zinc finger protein 780A (ZNF780A), mRNA [NM_001010880]	**-1.564**
ZNF509	Homo sapiens zinc finger protein 509 (ZNF509), mRNA [NM_145291]	**-1.563**
RNF214	Homo sapiens ring finger protein 214 (RNF214), mRNA [NM_001077239]	**-1.561**
SPATA9	Homo sapiens cDNA FLJ35906 fis, clone TESTI2009727. [AK093225]	**-1.561**
BCORL1	Homo sapiens BCL6 co-repressor-like 1 (BCORL1), mRNA [NM_021946]	**-1.555**
DKFZp686E2433	Homo sapiens similar to hypothetical protein 9630041N07 (DKFZp686E2433), mRNA [NM_001136116]	**-1.555**
E2F3	Homo sapiens E2F transcription factor 3 (E2F3), mRNA [NM_001949]	**-1.554**
ADAMTS4	Homo sapiens ADAM metallopeptidase with thrombospondin type 1 motif, 4 (ADAMTS4), mRNA [NM_005099]	**-1.553**
PHB2	Homo sapiens prohibitin 2 (PHB2), mRNA [NM_007273]	**-1.551**

MDGA1	Homo sapiens MAM domain containing glycosylphosphatidylinositol anchor 1 (MDGA1), mRNA [NM_153487]	**-1.549**
SVEP1	Homo sapiens cDNA FLJ14964 fis, clone PLACE4000581, moderately similar to FIBROPELLIN I PRECURSOR. [AK027870]	**-1.548**
GUCY2G	Homo sapiens guanylate cyclase 2G homolog (mouse) pseudogene (GUCY2G), mRNA [XM_001718434]	**-1.543**
PLAT	Homo sapiens plasminogen activator, tissue (PLAT), mRNA [NM_000930]	**-1.543**
FZD10	Homo sapiens frizzled homolog 10 (Drosophila) (FZD10), mRNA [NM_007197]	**-1.542**
FZD3	Homo sapiens frizzled homolog 3 (Drosophila) (FZD3), mRNA [NM_017412]	**-1.539**
LOC100144602	Homo sapiens hypothetical, mRNA (cDNA clone IMAGE:4429392). [BC017721]	**-1.538**
B3GAT2	Homo sapiens beta-1,3-glucuronyltransferase 2 (glucuronosyltransferase S) (B3GAT2), mRNA [NM_080742]	**-1.533**
SSH3	Homo sapiens slingshot homolog 3 (Drosophila) (SSH3), mRNA [NM_017857]	**-1.532**
ART3	Homo sapiens ADP-ribosyltransferase 3 (ART3), mRNA [NM_001179]	**-1.531**
LOC646446	Homo sapiens similar to hCG2040301 (LOC646446), mRNA [XM_001722653]	**-1.530**
RFX2	Homo sapiens regulatory factor X, 2 (influences HLA class II expression) (RFX2), mRNA [NM_000635]	**-1.530**
VWCE	Homo sapiens von Willebrand factor C and EGF domains (VWCE), mRNA [NM_152718]	**-1.527**
LOC203510	Homo sapiens similar to hCG1644442 (LOC203510), mRNA [XM_001719132]	**-1.526**
HIF3A	Homo sapiens hypoxia inducible factor 3, alpha subunit (HIF3A), mRNA [NM_022462]	**-1.523**
NKX3-1	Homo sapiens NK3 homeobox 1 (NKX3-1), mRNA [NM_006167]	**-1.523**
TMPRSS12	Homo sapiens transmembrane protease, serine 12 (TMPRSS12), mRNA [NM_182559]	**-1.521**
LOC729684	Homo sapiens misc_RNA (LOC729684), miscRNA [XR_039360]	**-1.519**
FAM84B	Homo sapiens family with sequence similarity 84, member B (FAM84B), mRNA [NM_174911]	**-1.517**
ESF1	Homo sapiens ESF1, nucleolar pre-rRNA processing protein, homolog (S. cerevisiae) (ESF1), mRNA [NM_016649]	**-1.516**
DEFB4	Homo sapiens defensin, beta 4 (DEFB4), mRNA [NM_004942]	**-1.513**
C17orf63	Homo sapiens chromosome 17 open reading frame 63 (C17orf63), mRNA [NM_018182]	**-1.511**
HTR5A	Homo sapiens 5-hydroxytryptamine (serotonin) receptor 5A (HTR5A), mRNA [NM_024012]	**-1.509**
TRIM74	Homo sapiens tripartite motif-containing 74 (TRIM74), mRNA [NM_198853]	**-1.505**
RGS9BP	Homo sapiens regulator of G protein signaling 9 binding protein (RGS9BP), mRNA [NM_207391]	**-1.504**
POPDC2	Homo sapiens popeye domain containing 2 (POPDC2), mRNA [NM_022135]	**-1.503**
LOC257396	Homo sapiens cDNA FLJ40574 fis, clone THYMU2007036. [AK097893]	**-1.501**
IGH@	Homo sapiens cDNA FLJ27104 fis, clone SPL04981, highly similar to Ig	**-1.498**

	gamma-2 chain C region. [AK130614]	
CYP2A13	Homo sapiens cytochrome P450, family 2, subfamily A, polypeptide 13 (CYP2A13), mRNA [NM_000766]	**-1.497**
MNT	Homo sapiens MAX binding protein (MNT), mRNA [NM_020310]	**-1.497**
SOX8	Homo sapiens SRY (sex determining region Y)-box 8 (SOX8), mRNA [NM_014587]	**-1.495**
C1orf56	Homo sapiens chromosome 1 open reading frame 56 (C1orf56), mRNA [NM_017860]	**-1.494**
LOC100128822	Homo sapiens cDNA clone IMAGE:5286843. [BC036622]	**-1.492**
GKAP1	Homo sapiens G kinase anchoring protein 1 (GKAP1), mRNA [NM_025211]	**-1.491**
LOC653773	Homo sapiens misc_RNA (LOC653773), partial miscRNA [XR_042355]	**-1.491**
NR4A1	Homo sapiens nuclear receptor subfamily 4, group A, member 1 (NR4A1), mRNA [NM_002135]	**-1.491**
LOC392382	Homo sapiens misc_RNA (LOC392382), miscRNA [XR_019110]	**-1.487**
FLJ40142	Homo sapiens FLJ40142 protein (FLJ40142), mRNA [NM_207435]	**-1.484**
NRCAM	Homo sapiens neuronal cell adhesion molecule (NRCAM), mRNA [NM_001037132]	**-1.483**
ELA2A	Homo sapiens elastase 2A (ELA2A), mRNA [NM_033440]	**-1.481**
LOC728198	Homo sapiens similar to transcription associated factor TAFII31L (LOC728198), mRNA [XM_001126120]	**-1.481**
LOC340508	Homo sapiens hypothetical protein LOC340508 (LOC340508), non-coding RNA [NR_002942]	**-1.480**
ANO3	Homo sapiens anoctamin 3 (ANO3), mRNA [NM_031418]	**-1.478**
LOC100134119	Homo sapiens similar to hCG2018924 (LOC100134119), mRNA [XM_001713939]	**-1.478**
LOC100131149	Homo sapiens misc_RNA (LOC100131149), miscRNA [XR_039101]	**-1.475**
ATP6V1G2	Homo sapiens ATPase, H+ transporting, lysosomal 13kDa, V1 subunit G2 (ATP6V1G2), mRNA [NM_130463]	**-1.473**
LOC126235	Full-length cDNA clone CS0DE004YN04 of Placenta of Homo sapiens (human). [CR622909]	**-1.471**
LOC391282	Homo sapiens similar to ribosomal protein L23a (LOC391282), mRNA [XM_372878]	**-1.468**
RRM1	Homo sapiens ribonucleotide reductase M1 (RRM1), mRNA [NM_001033]	**-1.464**
TMEM25	Homo sapiens transmembrane protein 25 (TMEM25), mRNA [NM_032780]	**-1.461**
ABAT	Homo sapiens 4-aminobutyrate aminotransferase (ABAT), nuclear gene encoding mitochondrial protein, mRNA [NM_000663]	**-1.460**
DLX4	Homo sapiens distal-less homeobox 4 (DLX4), mRNA [NM_138281]	**-1.459**
SCEL	Homo sapiens sciellin (SCEL), mRNA [NM_144777]	**-1.457**
CLIP4	Homo sapiens CAP-GLY domain containing linker protein family, member 4 (CLIP4), mRNA [NM_024692]	**-1.457**
ADAMTS5	Homo sapiens ADAM metallopeptidase with thrombospondin type 1 motif, 5 (ADAMTS5), mRNA [NM_007038]	**-1.452**
LOC284454	Homo sapiens mRNA; cDNA DKFZp686K181 (from clone DKFZp686K181).	**-1.447**

	[BX640708]	
ELP2P	Homo sapiens endozepine-like peptide 2 pseudogene (ELP2P), non-coding RNA [NR_024120]	**-1.446**
ICK	Homo sapiens intestinal cell (MAK-like) kinase (ICK), mRNA [NM_016513]	**-1.446**
ZNF555	Homo sapiens zinc finger protein 555 (ZNF555), mRNA [NM_152791]	**-1.445**
XK	Homo sapiens X-linked Kx blood group (McLeod syndrome) (XK), mRNA [NM_021083]	**-1.442**
HMP19	Homo sapiens HMP19 protein (HMP19), mRNA [NM_015980]	**-1.441**
PLEKHA7	Homo sapiens pleckstrin homology domain containing, family A member 7 (PLEKHA7), mRNA [NM_175058]	**-1.441**
CCDC68	Homo sapiens coiled-coil domain containing 68 (CCDC68), mRNA [NM_025214]	**-1.440**
HOXB2	Homo sapiens homeobox B2 (HOXB2), mRNA [NM_002145]	**-1.440**
PRR12	Homo sapiens proline rich 12 (PRR12), mRNA [NM_020719]	**-1.440**
ZCRB1	Homo sapiens zinc finger CCHC-type and RNA binding motif 1 (ZCRB1), mRNA [NM_033114]	**-1.439**
EED	Homo sapiens embryonic ectoderm development (EED), mRNA [NM_152991]	**-1.436**
PSCA	Homo sapiens prostate stem cell antigen (PSCA), mRNA [NM_005672]	**-1.436**
HS6ST1	Homo sapiens heparan sulfate 6-O-sulfotransferase 1 (HS6ST1), mRNA [NM_004807]	**-1.433**
HIG2	Homo sapiens hypoxia-inducible protein 2 (HIG2), mRNA [NM_013332]	**-1.429**
TBX15	Homo sapiens T-box 15 (TBX15), mRNA [NM_152380]	**-1.429**
LOC100133616	Putative uncharacterized protein FLJ35883 [Source:UniProtKB/Swiss-Prot; Acc:Q8NA34] [ENST00000357301]	**-1.428**
LOC440983	Homo sapiens hypothetical gene supported by BC066916, mRNA (cDNA clone IMAGE:4838452). [BC066916]	**-1.428**
AR	Homo sapiens androgen receptor (AR), mRNA [NM_000044]	**-1.427**
ITM2B	Homo sapiens integral membrane protein 2B (ITM2B), mRNA [NM_021999]	**-1.422**
ANO8	Homo sapiens anoctamin 8 (ANO8), mRNA [NM_020959]	**-1.420**
TSPAN10	Homo sapiens tetraspanin 10 (TSPAN10), mRNA [NM_031945]	**-1.420**
C20orf29	Homo sapiens chromosome 20 open reading frame 29 (C20orf29), mRNA [NM_018347]	**-1.419**
ZNF284	Zinc finger protein 284 [Source:UniProtKB/Swiss-Prot;Acc:Q2VY69] [ENST00000328297]	**-1.416**
DAND5	Homo sapiens DAN domain family, member 5 (DAND5), mRNA [NM_152654]	**-1.414**
GALC	Homo sapiens galactosylceramidase (GALC), mRNA [NM_000153]	**-1.413**
TAF1C	Homo sapiens TATA box binding protein (TBP)-associated factor, RNA polymerase I (TAF1C), mRNA [NM_005679]	**-1.413**
ATE1	Homo sapiens arginyltransferase 1 (ATE1), mRNA [NM_001001976]	**-1.410**
LIPA	Homo sapiens lipase A, lysosomal acid, cholesterol esterase (LIPA), mRNA [NM_000235]	**-1.404**
LOC441461	Homo sapiens hypothetical gene supported by BC030123, mRNA (cDNA clone IMAGE:4815474). [BC030123]	**-1.404**

HEY1	Homo sapiens hairy/enhancer-of-split related with YRPW motif 1 (HEY1), mRNA [NM_001040708]	**-1.403**
C8orf22	Homo sapiens chromosome 8 open reading frame 22 (C8orf22), mRNA [NM_001007176]	**-1.402**
C19orf66	Homo sapiens chromosome 19 open reading frame 66 (C19orf66), mRNA [NM_018381]	**-1.401**
FLJ25694	Homo sapiens cDNA FLJ46084 fis, clone TESTI2006543. [AK127969]	**-1.400**
LONRF2	Homo sapiens LON peptidase N-terminal domain and ring finger 2 (LONRF2), mRNA [NM_198461]	**-1.398**
TRIM66	Homo sapiens tripartite motif-containing 66 (TRIM66), mRNA [NM_014818]	**-1.398**
LOC51190	Homo sapiens neutral sphingomyelinase mRNA. [AF069740]	**-1.397**
C9orf103	Homo sapiens chromosome 9 open reading frame 103 (C9orf103), mRNA [NM_001001551]	**-1.396**
FTMT	Homo sapiens ferritin mitochondrial (FTMT), nuclear gene encoding mitochondrial protein, mRNA [NM_177478]	**-1.396**
UPB1	Homo sapiens ureidopropionase, beta (UPB1), mRNA [NM_016327]	**-1.395**
LOC283481	Homo sapiens hypothetical protein LOC283481, mRNA (cDNA clone IMAGE:5296747). [BC033993]	**-1.394**
PER2	Homo sapiens period homolog 2 (Drosophila) (PER2), mRNA [NM_022817]	**-1.394**
NAT8L	Homo sapiens N-acetyltransferase 8-like (GCN5-related, putative) (NAT8L), mRNA [NM_178557]	**-1.393**
RNF168	Homo sapiens ring finger protein 168 (RNF168), mRNA [NM_152617]	**-1.393**
RUFY1	Homo sapiens RUN and FYVE domain containing 1 (RUFY1), mRNA [NM_025158]	**-1.393**
UCA1	Homo sapiens urothelial cancer associated 1 (UCA1), non-coding RNA [NR_015379]	**-1.391**
RNF122	Homo sapiens ring finger protein 122 (RNF122), mRNA [NM_024787]	**-1.389**
PRAGMIN	Homo sapiens homolog of rat pragma of Rnd2 (PRAGMIN), mRNA [NM_001080826]	**-1.388**
LOC343495	Homo sapiens misc_RNA (LOC343495), miscRNA [XR_016540]	**-1.386**
SUZ12P	full-length cDNA clone CS0DC012YL18 of Neuroblastoma Cot 25-normalized of Homo sapiens (human). [CR597846]	**-1.386**
PDGFRL	Homo sapiens platelet-derived growth factor receptor-like (PDGFRL), mRNA [NM_006207]	**-1.385**
LOC339483	Homo sapiens cDNA FLJ38790 fis, clone LIVER2002842. [AK096109]	**-1.384**
RREB1	Homo sapiens ras responsive element binding protein 1 (RREB1), mRNA [NM_001003699]	**-1.384**
CXCL1	Homo sapiens chemokine (C-X-C motif) ligand 1 (melanoma growth stimulating activity, alpha) (CXCL1), mRNA [NM_001511]	**-1.383**
SERPINA1	Homo sapiens serpin peptidase inhibitor, clade A (alpha-1 antiproteinase, antitrypsin), member 1 (SERPINA1), mRNA [NM_001002236]	**-1.382**
ZNF737	Homo sapiens zinc finger protein 737, mRNA (cDNA clone IMAGE:4854518), [BC015765]	**-1.382**

SLC26A11	Homo sapiens solute carrier family 26, member 11 (SLC26A11), mRNA [NM_173626]	**-1.380**
MBNL3	Homo sapiens cDNA FLJ38120 fis, clone D3OST3000195. [AK095439]	**-1.378**
FRS3	Homo sapiens fibroblast growth factor receptor substrate 3 (FRS3), mRNA [NM_006653]	**-1.377**
PRDM12	Homo sapiens PR domain containing 12 (PRDM12), mRNA [NM_021619]	**-1.377**
FBXL16	Homo sapiens F-box and leucine-rich repeat protein 16 (FBXL16), mRNA [NM_153350]	**-1.376**
OR7E13P	Homo sapiens olfactory-like receptor PJCG2 (PJCG2) mRNA, [AF238487]	**-1.376**
ZNF48	Zinc finger protein 48 (Zinc finger protein 553) [Source:UniProtKB/Swiss-Prot ;Acc:Q96MX3] [ENST00000320159]	**-1.375**
MID2	Homo sapiens midline 2 (MID2), mRNA [NM_012216]	**-1.373**
C20orf197	Homo sapiens chromosome 20 open reading frame 197 (C20orf197), mRNA [NM_173644]	**-1.372**
PBX4	Homo sapiens pre-B-cell leukemia homeobox 4 (PBX4), mRNA [NM_025245]	**-1.372**
EIF1B	Homo sapiens eukaryotic translation initiation factor 1B (EIF1B), mRNA [NM_005875]	**-1.371**
SAR1P3	Homo sapiens SAR1 gene homolog (S. cerevisiae) pseudogene 3 (SAR1P3), mRNA [XM_001714154]	**-1.371**
C6orf105	Homo sapiens chromosome 6 open reading frame 105 (C6orf105), mRNA [NM_032744]	**-1.370**
LOC390413	Homo sapiens misc_RNA (LOC390413), miscRNA [XR_018341]	**-1.369**
COL8A2	Homo sapiens collagen, type VIII, alpha 2 (COL8A2), mRNA [NM_005202]	**-1.368**
AREG	Homo sapiens amphiregulin (AREG), mRNA [NM_001657]	**-1.367**
BCL3	Homo sapiens B-cell CLL/lymphoma 3 (BCL3), mRNA [NM_005178]	**-1.367**
LOC729332	Homo sapiens hypothetical LOC729332 (LOC729332), mRNA [XM_001129827]	**-1.366**
TTC9	Homo sapiens tetratricopeptide repeat domain 9 (TTC9), mRNA [NM_015351]	**-1.366**
IL17RD	Homo sapiens interleukin 17 receptor D (IL17RD), mRNA [NM_017563]	**-1.365**
ASB9	Homo sapiens ankyrin repeat and SOCS box-containing 9 (ASB9), mRNA [NM_001031739]	**-1.364**
LOC100132658	Homo sapiens misc_RNA (LOC100132658), miscRNA [XR_038952]	**-1.362**
C7orf53	Homo sapiens chromosome 7 open reading frame 53 (C7orf53), mRNA [NM_182597]	**-1.358**
RAD52	Homo sapiens RAD52 homolog (S. cerevisiae) (RAD52), mRNA [NM_134424]	**-1.358**
ZNF521	Homo sapiens zinc finger protein 521 (ZNF521), mRNA [NM_015461]	**-1.357**
PRRT3	Homo sapiens proline-rich transmembrane protein 3 (PRRT3), mRNA [NM_207351]	**-1.355**
ZNF177	Homo sapiens zinc finger protein 177 (ZNF177), mRNA [NM_003451]	**-1.351**
LRP5L	Homo sapiens low density lipoprotein receptor-related protein 5-like (LRP5L), mRNA [NM_182492]	**-1.350**
LOC100131323	Homo sapiens misc_RNA (LOC100131323), miscRNA [XR_039461]	**-1.349**
LOC728774	Homo sapiens similar to hCG1994130 (LOC728774), mRNA [XM_001129390]	**-1.349**

MST150	Homo sapiens MSTP150 (MST150), mRNA [NM_032947]	**-1.348**
SLC4A11	Homo sapiens solute carrier family 4, sodium borate transporter, member 11 (SLC4A11), mRNA [NM_032034]	**-1.347**
DPF3	Homo sapiens cDNA FLJ42956 fis, clone BRSTN2009899. [AK124946]	**-1.342**
LOC645231	full-length cDNA clone CS0DI026YJ08 of Placenta Cot 25-normalized of Homo sapiens (human). [CR590757]	**-1.338**
AIG1	Homo sapiens androgen-induced 1 (AIG1), mRNA [NM_016108]	**-1.336**
GCSH	Homo sapiens glycine cleavage system protein H (aminomethyl carrier) (GCSH), mRNA [NM_004483]	**-1.336**
RNF215	Homo sapiens ring finger protein 215 (RNF215), mRNA [NM_001017981]	**-1.335**
SPRYD4	Homo sapiens SPRY domain containing 4 (SPRYD4), mRNA [NM_207344]	**-1.335**
ZNF167	Homo sapiens zinc finger protein 167 (ZNF167), mRNA [NM_025169]	**-1.335**
DHDH	Homo sapiens dihydrodiol dehydrogenase (dimeric) (DHDH), mRNA [NM_014475]	**-1.333**
FKSG2	Homo sapiens apoptosis inhibitor (FKSG2), mRNA [NM_021631]	**-1.333**
ZNF449	Homo sapiens cDNA FLJ23614 fis, clone ADKA03275. [AK074194]	**-1.333**
ZNF486	Homo sapiens zinc finger protein 486 (ZNF486), mRNA [NM_052852]	**-1.333**
C17orf97	Homo sapiens chromosome 17 open reading frame 97 (C17orf97), mRNA [NM_001013672]	**-1.332**
LOC100129385	Putative uncharacterized protein C9orf92 [Source:UniProtKB/Swiss-Prot; Acc:A6NGG3] [ENST00000380683]	**-1.332**
NET1	Homo sapiens neuroepithelial cell transforming 1 (NET1), mRNA [NM_001047160]	**-1.332**
TRUB1	Homo sapiens TruB pseudouridine (psi) synthase homolog 1 (E. coli) (TRUB1), mRNA [NM_139169]	**-1.332**
C2orf42	Homo sapiens chromosome 2 open reading frame 42 (C2orf42), mRNA [NM_017880]	**-1.330**
ABCC6	Homo sapiens ATP-binding cassette, sub-family C (CFTR/MRP), member 6 (ABCC6), mRNA [NM_001171]	**-1.328**
APCDD1	Homo sapiens adenomatosis polyposis coli down-regulated 1 (APCDD1), mRNA [NM_153000]	**-1.328**
HSPA12A	Homo sapiens heat shock 70kDa protein 12A (HSPA12A), mRNA [NM_025015]	**-1.326**
MECP2	Homo sapiens methyl CpG binding protein 2 (Rett syndrome) (MECP2), mRNA [NM_004992]	**-1.326**
VGLL3	Homo sapiens vestigial like 3 (Drosophila) (VGLL3), mRNA [NM_016206]	**-1.325**
ABCD4	Homo sapiens ATP-binding cassette, sub-family D (ALD), member 4 (ABCD4), mRNA [NM_005050]	**-1.323**
ARNT2	Homo sapiens aryl-hydrocarbon receptor nuclear translocator 2 (ARNT2), mRNA [NM_014862]	**-1.323**
C2orf60	Homo sapiens chromosome 2 open reading frame 60 (C2orf60), mRNA [NM_001039693]	**-1.322**
LOC286071	Homo sapiens cDNA FLJ34440 fis, clone HLUNG2001214. [AK091759]	**-1.322**

ZNF828	Homo sapiens zinc finger protein 828 (ZNF828), mRNA [NM_032436]	**-1.322**
C18orf55	Homo sapiens chromosome 18 open reading frame 55 (C18orf55), mRNA [NM_014177]	**-1.322**
RPL32P3	Homo sapiens ribosomal protein L32 pseudogene 3 (RPL32P3), non-coding RNA [NR_003111]	**-1.321**
E2F8	Homo sapiens E2F transcription factor 8 (E2F8), mRNA [NM_024680]	**-1.319**
SSPN	Homo sapiens sarcospan (Kras oncogene-associated gene) (SSPN), mRNA [NM_005086]	**-1.319**
FAM117B	Homo sapiens family with sequence similarity 117, member B (FAM117B), mRNA [NM_173511]	**-1.318**
TBC1D8B	Homo sapiens TBC1 domain family, member 8B (with GRAM domain) (TBC1D8B), mRNA [NM_017752]	**-1.316**
CECR1	Homo sapiens cat eye syndrome chromosome region, candidate 1 (CECR1), mRNA [NM_017424]	**-1.314**
LOC344595	Homo sapiens hypothetical LOC344595, mRNA (cDNA clone IMAGE:5760770). [BC039550]	**-1.311**
MMP12	Homo sapiens matrix metallopeptidase 12 (macrophage elastase) (MMP12), mRNA [NM_002426]	**-1.310**
TMEM129	Homo sapiens transmembrane protein 129 (TMEM129), mRNA [NM_138385]	**-1.309**
LOC100129104	Full-length cDNA clone CS0DI026YO05 of Placenta Cot 25-normalized of Homo sapiens (human). [CR610759]	**-1.308**
WIPI1	Homo sapiens WD repeat domain, phosphoinositide interacting 1 (WIPI1), mRNA [NM_017983]	**-1.308**
CMTM8	Homo sapiens CKLF-like MARVEL transmembrane domain containing 8 (CMTM8), mRNA [NM_178868]	**-1.307**
FLJ12547	Homo sapiens cDNA FLJ12547 fis, clone NT2RM4000634. [AK022609]	**-1.307**
PLAGL2	Homo sapiens pleiomorphic adenoma gene-like 2 (PLAGL2), mRNA [NM_002657]	**-1.307**
CACNB1	Homo sapiens calcium channel, voltage-dependent, beta 1 subunit (CACNB1), mRNA [NM_000723]	**-1.307**
FAP	Homo sapiens fibroblast activation protein, alpha (FAP), mRNA [NM_004460]	**-1.305**
BFSP1	Homo sapiens beaded filament structural protein 1, filensin (BFSP1), mRNA [NM_001195]	**-1.304**
DND1	Homo sapiens dead end homolog 1 (zebrafish) (DND1), mRNA [NM_194249]	**-1.304**
OXCT2	Homo sapiens 3-oxoacid CoA transferase 2 (OXCT2), mRNA [NM_022120]	**-1.304**
ADCY10	Homo sapiens adenylate cyclase 10 (soluble) (ADCY10), mRNA [NM_018417]	**-1.303**
SEMA4C	Homo sapiens sema domain, immunoglobulin domain (Ig), transmembrane domain (TM) and short cytoplasmic domain, semaphorin 4C (SEMA4C), mRNA [NM_017789]	**-1.302**
TRPV3	Homo sapiens transient receptor potential cation channel, subfamily V, member 3 (TRPV3), mRNA [NM_145068]	**-1.302**
TSPYL2	Homo sapiens TSPY-like 2 (TSPYL2), mRNA [NM_022117]	**-1.302**
MRPL16	Homo sapiens mitochondrial ribosomal protein L16 (MRPL16),mRNA	**-1.301**

	[NM_017840]	
KLRC2	Homo sapiens killer cell lectin-like receptor subfamily C, member 2 (KLRC2), mRNA [NM_002260]	**-1.300**
TAF6	Homo sapiens TAF6 RNA polymerase II, TATA box binding protein (TBP)-associated factor, 80kDa (TAF6), mRNA [NM_005641]	**-1.300**
ID2	Homo sapiens inhibitor of DNA binding 2, dominant negative helix-loop-helix protein (ID2), mRNA [NM_002166]	**-1.297**
IL17RE	Homo sapiens interleukin 17 receptor E (IL17RE), mRNA [NM_153483]	**-1.297**
LOC730834	DB090170 TESTI4 Homo sapiens cDNA clone TESTI4038997 5', mRNA sequence [DB090170]	**-1.297**
C13orf33	Homo sapiens chromosome 13 open reading frame 33 (C13orf33), mRNA [NM_032849]	**-1.296**
HOXC9	Homo sapiens homeobox C9 (HOXC9), mRNA [NM_006897]	**-1.296**
RTN4R	Homo sapiens reticulon 4 receptor (RTN4R), mRNA [NM_023004]	**-1.296**
TRIM62	Homo sapiens tripartite motif-containing 62 (TRIM62), mRNA [NM_018207]	**-1.296**
SPIN4	Homo sapiens spindlin family, member 4 (SPIN4), mRNA [NM_001012968]	**-1.295**
HCFC2	Homo sapiens host cell factor C2 (HCFC2), mRNA [NM_013320]	**-1.293**
LOC130728	Homo sapiens misc_RNA (LOC130728), miscRNA [XR_019248]	**-1.292**
LOC646993	Homo sapiens similar to high-mobility group box 3 (LOC646993), mRNA [XM_929965]	**-1.291**
PRRT2	Homo sapiens proline-rich transmembrane protein 2 (PRRT2), mRNA [NM_145239]	**-1.289**
SLC10A4	Homo sapiens solute carrier family 10 (sodium/bile acid cotransporter family), member 4 (SLC10A4), mRNA [NM_152679]	**-1.287**
CHRFAM7A	full-length cDNA clone CS0DD004YC02 of Neuroblastoma Cot 50-normalized of Homo sapiens (human). [CR605947]	**-1.286**
LOC730091	Homo sapiens cDNA FLJ31839 fis, clone NT2RP7000086. [AK056401]	**-1.286**
KCNG3	Homo sapiens potassium voltage-gated channel, subfamily G, member 3 (KCNG3), mRNA [NM_133329]	**-1.285**
JMY	Homo sapiens junction-mediating and regulatory protein (JMY), mRNA [NM_152405]	**-1.283**
LOC170082	TFIIS central domain-containing protein 1 [Source:UniProtKB/Swiss-Prot; Acc:Q8N8B7] [ENST00000380600]	**-1.283**
CENPK	Homo sapiens centromere protein K (CENPK), mRNA [NM_022145]	**-1.280**
LQK1	Homo sapiens LQK1 hypothetical protein long isoform (LQK1) mRNA, alternatively spliced. [AY030238]	**-1.280**
PSKH1	Homo sapiens protein serine kinase H1 (PSKH1), mRNA [NM_006742]	**-1.280**
C15orf39	Homo sapiens chromosome 15 open reading frame 39 (C15orf39), mRNA [NM_015492]	**-1.279**
CDSN	Homo sapiens corneodesmosin (CDSN), mRNA [NM_001264]	**-1.278**
FAM113B	Homo sapiens family with sequence similarity 113, member B (FAM113B), mRNA [NM_138371]	**-1.278**
KIAA0895L	Homo sapiens cDNA FLJ34984 fis, clone OCBBF2001639. [AK092303]	**-1.278**

CTNNBIP1	Homo sapiens catenin, beta interacting protein 1 (CTNNBIP1), mRNA [NM_020248]	**-1.275**
TMEM55A	Homo sapiens transmembrane protein 55A (TMEM55A), mRNA [NM_018710]	**-1.275**
LOC100130171	Homo sapiens misc_RNA (LOC100130171), miscRNA [XR_038676]	**-1.274**
SFTA3	Homo sapiens surfactant associated 3 (SFTA3), mRNA [NM_001101341]	**-1.274**
TTC7B	Homo sapiens tetratricopeptide repeat domain 7B (TTC7B), mRNA [NM_001010854]	**-1.274**
LOC100128626	Homo sapiens misc_RNA (LOC100128626), miscRNA [XR_038662]	**-1.271**
CBWD6	Homo sapiens clone 1659351 unknown mRNA. [AF293368]	**-1.270**
UBLCP1	Homo sapiens ubiquitin-like domain containing CTD phosphatase 1 (UBLCP1), mRNA [NM_145049]	**-1.269**
C10orf140	Homo sapiens chromosome 10 open reading frame 140 (C10orf140), mRNA [NM_207371]	**-1.267**
C16orf35	Homo sapiens chromosome 16 open reading frame 35 (C16orf35), mRNA [NM_001039476]	**-1.267**
LOC284570	Homo sapiens, clone IMAGE:4941949, mRNA. [BC040156]	**-1.267**
C1orf226	Homo sapiens chromosome 1 open reading frame 226 (C1orf226), mRNA [NM_001135240]	**-1.266**
GNAO1	Homo sapiens cDNA clone IMAGE:4181241. [BC012202]	**-1.265**
GSK3A	Homo sapiens glycogen synthase kinase 3 alpha (GSK3A), mRNA [NM_019884]	**-1.265**
MRPS18B	Homo sapiens mitochondrial ribosomal protein S18B (MRPS18B),mRNA [NM_014046]	**-1.265**
LOC728705	Homo sapiens cDNA FLJ31150 fis, clone IMR322001534. [AK055712]	**-1.264**
C9orf68	Homo sapiens chromosome 9 open reading frame 68 (C9orf68), mRNA [NM_001039395]	**-1.263**
CAND2	Homo sapiens cullin-associated and neddylation-dissociated 2 (putative) (CAND2), mRNA [NM_012298]	**-1.263**
USP54	Homo sapiens ubiquitin specific peptidase 54 (USP54), mRNA [NM_152586]	**-1.262**
LOC644189	Homo sapiens cDNA FLJ35203 fis, clone PLACE6018441, moderately similar to Mus musculus peroxisomal long chain acyl-CoA thioesterase Ib (Pte1b) gene. [AK092522]	**-1.261**
ZNF550	Homo sapiens zinc finger protein 550, mRNA (cDNA clone IMAGE:6044705). [BC053858]	**-1.261**
NF-E4	Homo sapiens, clone IMAGE:5404753, mRNA. [BC036938]	**-1.259**
ZNF354B	Homo sapiens zinc finger protein 354B (ZNF354B), mRNA [NM_058230]	**-1.259**
ZNF671	Homo sapiens zinc finger protein 671 (ZNF671), mRNA [NM_024833]	**-1.259**
FOXD2	Homo sapiens forkhead box D2 (FOXD2), mRNA [NM_004474]	**-1.258**
RFFL	Homo sapiens ring finger and FYVE-like domain containing 1 (RFFL), mRNA [NM_057178]	**-1.258**
BHLHE22	Homo sapiens basic helix-loop-helix domain containing, class B, 5 (BHLHB5), mRNA [NM_152414]	**-1.257**
EID2	Homo sapiens EP300 interacting inhibitor of differentiation 2 (EID2), mRNA	**-1.257**

	[NM_153232]	
LHX2	Homo sapiens LIM homeobox 2 (LHX2), mRNA [NM_004789]	**-1.257**
LOC440348	Homo sapiens similar to nuclear pore complex interacting protein (LOC440348), mRNA [NM_001018059]	**-1.255**
ZNF200	Homo sapiens zinc finger protein 200 (ZNF200), mRNA [NM_003454]	**-1.255**
AGXT2L1	Homo sapiens alanine-glyoxylate aminotransferase 2-like 1 (AGXT2L1), mRNA [NM_031279]	**-1.253**
PARP4	Homo sapiens poly (ADP-ribose) polymerase family, member 4 (PARP4), mRNA [NM_006437]	**-1.253**
C10orf32	Homo sapiens chromosome 10 open reading frame 32 (C10orf32), mRNA [NM_144591]	**-1.252**
C15orf5	Homo sapiens chromosome 15 open reading frame 5, mRNA (cDNA clone MGC:97283 IMAGE:7262532),[BC069765]	**-1.252**
CXCR4	Homo sapiens chemokine (C-X-C motif) receptor 4 (CXCR4), mRNA [NM_001008540]	**-1.252**
HIST1H3J	Homo sapiens histone cluster 1, H3j (HIST1H3J), mRNA [NM_003535]	**-1.252**
LOC100132086	Homo sapiens misc_RNA (LOC100132086), miscRNA [XR_037769]	**-1.251**
THAP2	Homo sapiens THAP domain containing, apoptosis associated protein 2 (THAP2), mRNA [NM_031435]	**-1.251**
MADCAM1	Homo sapiens mucosal vascular addressin cell adhesion molecule 1 (MADCAM1), mRNA [NM_130760]	**-1.250**
THAP10	Homo sapiens THAP domain containing 10 (THAP10), mRNA [NM_020147]	**-1.249**
TFPI2	Homo sapiens tissue factor pathway inhibitor 2 (TFPI2), mRNA [NM_006528]	**-1.248**
LOC492311	Homo sapiens similar to bovine IgA regulatory protein (LOC492311), mRNA [NM_001007189]	**-1.247**
LOC643783	Homo sapiens hypothetical LOC643783 (LOC643783), mRNA [XM_931798]	**-1.246**
RECQL	Homo sapiens RecQ protein-like (DNA helicase Q1-like) (RECQL), mRNA [NM_032941]	**-1.246**
MTMR11	Homo sapiens myotubularin related protein 11 (MTMR11), mRNA [NM_181873]	**-1.245**
BEND5	Homo sapiens BEN domain containing 5 (BEND5), mRNA [NM_024603]	**-1.244**
ARP11	Homo sapiens ARP11 mRNA for actin-related protein, complete cds. [AB039791]	**-1.242**
TSLP	Homo sapiens thymic stromal lymphopoietin (TSLP), mRNA [NM_033035]	**-1.242**
CISH	Homo sapiens cytokine inducible SH2-containing protein (CISH), mRNA [NM_145071]	**-1.241**
GLRXL	Homo sapiens glutaredoxin (thioltransferase)-like (GLRXL), mRNA [NM_001123388]	**-1.241**
LOC727933	Homo sapiens hypothetical LOC727933 (LOC727933), mRNA [XM_001128972]	**-1.241**
CA5A	Homo sapiens carbonic anhydrase VA, mitochondrial (CA5A),mRNA [NM_001739]	**-1.240**
CAPN6	Homo sapiens calpain 6 (CAPN6), mRNA [NM_014289]	**-1.240**

BEND3	Homo sapiens BEN domain containing 3 (BEND3), mRNA [NM_001080450]	**-1.239**
SIGLEC9	Homo sapiens sialic acid binding Ig-like lectin 9 (SIGLEC9), mRNA [NM_014441]	**-1.239**
ZNF790	Homo sapiens zinc finger protein 790 (ZNF790), mRNA [NM_206894]	**-1.238**
FGF12	Homo sapiens fibroblast growth factor 12 (FGF12), mRNA [NM_004113]	**-1.238**
ZNF618	Homo sapiens zinc finger protein 618 (ZNF618), mRNA [NM_133374]	**-1.238**
DDX58	Homo sapiens DEAD (Asp-Glu-Ala-Asp) box polypeptide 58 (DDX58), mRNA [NM_014314]	**-1.237**
PAQR8	Homo sapiens progestin and adipoQ receptor family member VIII (PAQR8), mRNA [NM_133367]	**-1.237**
KIAA1524	Homo sapiens KIAA1524 (KIAA1524), mRNA [NM_020890]	**-1.236**
DDC	Homo sapiens dopa decarboxylase (aromatic L-amino acid decarboxylase) (DDC), mRNA [NM_000790]	**-1.236**
RHOH	Homo sapiens ras homolog gene family, member H (RHOH), mRNA [NM_004310]	**-1.236**
MPHOSPH6	Homo sapiens M-phase phosphoprotein 6 (MPHOSPH6), mRNA [NM_005792]	**-1.235**
CTSK	Homo sapiens cathepsin K (CTSK), mRNA [NM_000396]	**-1.233**
TBC1D24	Homo sapiens TBC1 domain family, member 24 (TBC1D24), mRNA [NM_020705]	**-1.233**
BMP7	Homo sapiens bone morphogenetic protein 7 (BMP7), mRNA [NM_001719]	**-1.232**
RLTPR	Homo sapiens mRNA for FLJ00330 protein. [AK090421]	**-1.232**
RHD	Homo sapiens Rh blood group, D antigen (RHD), mRNA [NM_016124]	**-1.231**
ANXA7	Homo sapiens cDNA clone IMAGE:2455346 3' similar to gb:J04543 ANNEXIN VII (HUMAN);, mRNA sequence [AI922845]	**-1.230**
SLC31A2	Homo sapiens solute carrier family 31 (copper transporters), member 2 (SLC31A2), mRNA [NM_001860]	**-1.230**
FAM162A	Homo sapiens family with sequence similarity 162, member A (FAM162A), mRNA [NM_014367]	**-1.229**
KLRG1	Homo sapiens killer cell lectin-like receptor subfamily G, member 1 (KLRG1), mRNA [NM_005810]	**-1.229**
MNAT1	Homo sapiens menage a trois homolog 1, cyclin H assembly factor (Xenopus laevis) (MNAT1), mRNA [NM_002431]	**-1.229**
KCNQ1DN	Homo sapiens KCNQ1 downstream neighbor (KCNQ1DN), non-coding RNA [NR_024627]	**-1.228**
REEP2	Homo sapiens receptor accessory protein 2 (REEP2), mRNA [NM_016606]	**-1.228**
ATOH1	Homo sapiens atonal homolog 1 (Drosophila) (ATOH1), mRNA [NM_005172]	**-1.227**
KLRG2	Homo sapiens cDNA FLJ44186 fis, clone THYMU2038797, [AK126174]	**-1.227**
NR4A3	Homo sapiens nuclear receptor subfamily 4, group A, member 3 (NR4A3), mRNA [NM_173199]	**-1.226**
ZNF442	Homo sapiens zinc finger protein 442 (ZNF442), mRNA [NM_030824]	**-1.226**
FAM63A	Homo sapiens mRNA for KIAA1390 protein. [AB037811]	**-1.226**
PCMTD1	Homo sapiens protein-L-isoaspartate (D-aspartate) O-methyltransferase domain containing 1 (PCMTD1), mRNA [NM_052937]	**-1.221**

MED4	Homo sapiens mediator complex subunit 4 (MED4), mRNA [NM_014166]	**-1.221**
RADIL	Homo sapiens Rap GTPase interactor (RADIL), mRNA [NM_018059]	**-1.221**
POLB	Homo sapiens polymerase (DNA directed), beta (POLB), mRNA [NM_002690]	**-1.220**
CMC1	Homo sapiens COX assembly mitochondrial protein homolog (CMC1), mRNA [NM_182523]	**-1.219**
MCART3P	Homo sapiens similar to mitochondrial carrier triple repeat 1, mRNA (cDNA clone MGC:33544 IMAGE:4821649). [BC024198]	**-1.219**
ZNF770	Homo sapiens zinc finger protein 770 (ZNF770), mRNA [NM_014106]	**-1.219**
AFAP1L2	Homo sapiens actin filament associated protein 1-like 2 (AFAP1L2), mRNA [NM_032550]	**-1.218**
CLDN11	Homo sapiens full length insert cDNA clone YO11A01. [AF085871]	**-1.217**
DNAJC25	Homo sapiens DnaJ (Hsp40) homolog, subfamily C , member 25 (DNAJC25), mRNA [NM_001015882]	**-1.215**
ZBED2	Homo sapiens zinc finger, BED-type containing 2 (ZBED2), mRNA [NM_024508]	**-1.215**
EID2B	Homo sapiens EP300 interacting inhibitor of differentiation 2B (EID2B), mRNA [NM_152361]	**-1.214**
MBP	Homo sapiens myelin basic protein (MBP), mRNA [NM_001025100]	**-1.214**
CECR4	Homo sapiens cat eye syndrome chromosome region, candidate 4 (non-protein coding) (CECR4), non-coding RNA [NR_024482]	**-1.213**
KLRC4	Homo sapiens killer cell lectin-like receptor subfamily C, member 4 (KLRC4), mRNA [NM_013431]	**-1.213**
CNIH	Homo sapiens cornichon homolog (Drosophila) (CNIH), mRNA [NM_005776]	**-1.211**
RGL1	Homo sapiens ral guanine nucleotide dissociation stimulator-like 1 (RGL1), mRNA [NM_015149]	**-1.211**
LOC644246	Homo sapiens hypothetical protein LOC644246, mRNA (cDNA clone IMAGE:4730995). [BC020847]	**-1.210**
LOC253482	Homo sapiens misc_RNA (LOC253482), miscRNA [XR_016415]	**-1.209**
FLJ22536	Homo sapiens cDNA FLJ37399 fis, clone BRAMY2027587. [AK094718]	**-1.209**
ALG8	Homo sapiens asparagine-linked glycosylation 8, alpha-1,3-glucosyltransferase homolog (S. cerevisiae) (ALG8), mRNA [NM_001007027]	**-1.208**
EFNA5	Homo sapiens ephrin-A5 (EFNA5), mRNA [NM_001962]	**-1.208**
ENPP4	Homo sapiens ectonucleotide pyrophosphatase/phosphodiesterase 4 (ENPP4), mRNA [NM_014936]	**-1.208**
RAB33B	Homo sapiens RAB33B, member RAS oncogene family (RAB33B), mRNA [NM_031296]	**-1.208**
ZC3H12A	Homo sapiens zinc finger CCCH-type containing 12A (ZC3H12A), mRNA [NM_025079]	**-1.207**
C13orf23	Homo sapiens chromosome 13 open reading frame 23 (C13orf23), mRNA [NM_025138]	**-1.207**
GPM6B	Homo sapiens glycoprotein M6B (GPM6B), mRNA [NM_001001996]	**-1.206**
SEPW1	Homo sapiens selenoprotein W, 1 (SEPW1), mRNA [NM_003009]	**-1.206**
ST6GAL1	Homo sapiens ST6 beta-galactosamide alpha-2,6-sialyltranferase 1 (ST6GAL1),	**-1.206**

	mRNA [NM_173216]	
CLN8	Homo sapiens ceroid-lipofuscinosis, neuronal 8 (epilepsy, progressive with mental retardation) (CLN8), mRNA [NM_018941]	**-1.205**
KIAA0746	Homo sapiens KIAA0746 protein (KIAA0746), mRNA [NM_015187]	**-1.204**
TP63	Homo sapiens tumor protein p63 (TP63), mRNA [NM_003722]	**-1.203**
ZFP112	Homo sapiens zinc finger protein 112 homolog (mouse) (ZFP112), mRNA [NM_013380]	**-1.203**
CNFN	Homo sapiens cornifelin (CNFN), mRNA [NM_032488]	**-1.202**
LOC643205	Homo sapiens hypothetical LOC643205 (LOC643205), mRNA [XM_001716733]	**-1.202**
MYB	Homo sapiens v-myb myeloblastosis viral oncogene homolog (avian) (MYB), mRNA [NM_005375]	**-1.202**
RPS6P1	Homo sapiens misc_RNA (RPS6P1), miscRNA [XR_016837]	**-1.201**
EFR3B	Homo sapiens EFR3 homolog B (S. cerevisiae) (EFR3B), mRNA [NM_014971]	**-1.200**
LOC90499	Homo sapiens mRNA; cDNA DKFZp434H0923 (from clone DKFZp434H0923). [AL137712]	**-1.199**
MAP3K10	Homo sapiens mitogen-activated protein kinase kinase kinase 10 (MAP3K10), mRNA [NM_002446]	**-1.199**
PDGFA	Homo sapiens platelet-derived growth factor alpha polypeptide (PDGFA), mRNA [NM_002607]	**-1.199**
NACAP1	Homo sapiens nascent-polypeptide-associated complex alpha polypeptide pseudogene 1 (NACAP1), non-coding RNA [NR_002182]	**-1.198**
APEX2	Homo sapiens APEX nuclease (apurinic/apyrimidinic endonuclease) 2 (APEX2), nuclear gene encoding mitochondrial protein, mRNA [NM_014481]	**-1.196**
FEM1B	Homo sapiens fem-1 homolog b (C. elegans) (FEM1B), mRNA [NM_015322]	**-1.195**
RAD18	Homo sapiens RAD18 homolog (S. cerevisiae) (RAD18), mRNA [NM_020165]	**-1.195**
RPAP1	Homo sapiens RNA polymerase II associated protein 1 (RPAP1), mRNA [NM_015540]	**-1.195**
SNRPG	Homo sapiens small nuclear ribonucleoprotein polypeptide G (SNRPG), mRNA [NM_003096]	**-1.195**
HPRT1	Homo sapiens hypoxanthine phosphoribosyltransferase 1 (HPRT1), mRNA [NM_000194]	**-1.194**
TRDN	Homo sapiens mRNA for triadin (TRDN gene), Trisk 51 isoform. [AJ489257]	**-1.194**
SENP6	Homo sapiens SUMO1/sentrin specific peptidase 6 (SENP6), mRNA [NM_015571]	**-1.190**
AGBL1	Homo sapiens cDNA FLJ32310 fis, clone PROST2003102. [AK056872]	**-1.189**
TSC1	Homo sapiens tuberous sclerosis 1 (TSC1), mRNA [NM_000368]	**-1.189**
UCN2	Homo sapiens urocortin 2 (UCN2), mRNA [NM_033199]	**-1.189**
NEUROG3	Homo sapiens neurogenin 3 (NEUROG3), mRNA [NM_020999]	**-1.188**
TMEM72	Homo sapiens transmembrane protein 72 (TMEM72), mRNA [NM_001123376]	**-1.188**
SPTLC3	Homo sapiens serine palmitoyltransferase, long chain base subunit 3 (SPTLC3), mRNA [NM_018327]	**-1.188**
C6orf159	Homo sapiens chromosome 6 open reading frame 159 (C6orf159), mRNA	**-1.187**

	[NM_001009994]	
LOC100131575	Homo sapiens misc_RNA (LOC100131575), miscRNA [XR_038726]	**-1.187**
ZFHX3	Homo sapiens zinc finger homeobox 3 (ZFHX3), mRNA [NM_006885]	**-1.184**
SLITRK6	Homo sapiens SLIT and NTRK-like family, member 6 (SLITRK6), mRNA [NM_032229]	**-1.183**
C6orf206	Homo sapiens chromosome 6 open reading frame 206 (C6orf206), mRNA [NM_152732]	**-1.181**
LRP4	Homo sapiens low density lipoprotein receptor-related protein 4 (LRP4), mRNA [NM_002334]	**-1.181**
ZUFSP	Homo sapiens zinc finger with UFM1-specific peptidase domain (ZUFSP), mRNA [NM_145062]	**-1.180**
ZNF777	Homo sapiens zinc finger protein 777 (ZNF777), mRNA [NM_015694]	**-1.178**
C7orf29	Homo sapiens chromosome 7 open reading frame 29 (C7orf29), mRNA [NM_138434]	**-1.177**
FAM49B	Homo sapiens family with sequence similarity 49, member B (FAM49B), mRNA [NM_016623]	**-1.177**
SSX2IP	Homo sapiens synovial sarcoma, X breakpoint 2 interacting protein (SSX2IP), mRNA [NM_014021]	**-1.177**
CUZD1	Homo sapiens CUB and zona pellucida-like domains 1 (CUZD1), mRNA [NM_022034]	**-1.175**
FYCO1	Homo sapiens FYVE and coiled-coil domain containing 1 (FYCO1), mRNA [NM_024513]	**-1.175**
OSR2	Homo sapiens odd-skipped related 2 (Drosophila) (OSR2), mRNA [NM_053001]	**-1.175**
C9orf125	Transmembrane protein C9orf125 [Source:UniProtKB/Swiss-Prot;Acc: Q9BRR3] [ENST00000374851]	**-1.175**
FLJ27352	Homo sapiens cDNA FLJ27352 fis, clone TST05165. [AK130862]	**-1.174**
SALL2	Homo sapiens sal-like 2 (Drosophila) (SALL2), mRNA [NM_005407]	**-1.174**
ETFDH	Homo sapiens electron-transferring-flavoprotein dehydrogenase (ETFDH), mRNA [NM_004453]	**-1.173**
LOC100130476	Homo sapiens cDNA FLJ42179 fis, clone THYMU2030796. [AK124173]	**-1.173**
RFTN1	Homo sapiens raftlin, lipid raft linker 1 (RFTN1), mRNA [NM_015150]	**-1.173**
TLE6	Homo sapiens transducin-like enhancer of split 6 (E(sp1) homolog, Drosophila), mRNA (cDNA clone IMAGE:3687767). [BC007329]	**-1.173**
ITGB1BP2	Homo sapiens integrin beta 1 binding protein (melusin) 2 (ITGB1BP2), mRNA [NM_012278]	**-1.172**
LOC645733	Homo sapiens similar to hCG1812929 (LOC645733), mRNA [XM_001130623]	**-1.171**
LGR6	Homo sapiens leucine-rich repeat-containing G protein-coupled receptor 6 (LGR6), mRNA [NM_001017403]	**-1.170**
NSBP1	Homo sapiens nucleosomal binding protein 1 (NSBP1), mRNA [NM_030763]	**-1.169**
CGREF1	Homo sapiens cell growth regulator with EF-hand domain 1 (CGREF1), mRNA [NM_006569]	**-1.168**
LOC100128266	Homo sapiens misc_RNA (LOC100128266), miscRNA [XR_037888]	**-1.168**

GLI3	Homo sapiens GLI-Kruppel family member GLI3 (GLI3), mRNA [NM_000168]	**-1.167**
NUDT9	Homo sapiens nudix (nucleoside diphosphate linked moiety X)-type motif 9 (NUDT9), mRNA [NM_024047]	**-1.166**
LOC401022	Homo sapiens hypothetical LOC401022, mRNA (cDNA clone IMAGE:4827714). [BC030713]	**-1.164**
LOH12CR2	Homo sapiens loss of heterozygosity, 12, chromosomal region 2 (LOH12CR2), non-coding RNA [NR_024061]	**-1.164**
SEMA3D	Homo sapiens sema domain, immunoglobulin domain (Ig), short basic domain, secreted, (semaphorin) 3D (SEMA3D), mRNA [NM_152754]	**-1.164**
C1orf135	Homo sapiens chromosome 1 open reading frame 135 (C1orf135), mRNA [NM_024037]	**-1.163**
MTHFD2L	Homo sapiens methylenetetrahydrofolate dehydrogenase (NADP+ dependent) 2-like, mRNA (cDNA clone IMAGE:4794959), complete cds. [BC037529]	**-1.163**
C1orf133	Homo sapiens chromosome 1 open reading frame 133 (C1orf133), non-coding RNA [NR_024337]	**-1.162**
LOC401097	Homo sapiens Similar to LOC166075, mRNA (cDNA clone IMAGE:5173621),. [BC031660]	**-1.161**
CABYR	Homo sapiens calcium binding tyrosine-(Y)-phosphorylation regulated (CABYR), mRNA [NM_012189]	**-1.160**
G0S2	Homo sapiens G0/G1switch 2 (G0S2), mRNA [NM_015714]	**-1.160**
ZAN	Homo sapiens zonadhesin (ZAN), mRNA [NM_173059]	**-1.160**
LOC732275	Homo sapiens similar to hCG1645603 (LOC732275), non-coding RNA [NR_024406]	**-1.159**
FAM86A	Homo sapiens family with sequence similarity 86, member A (FAM86A), mRNA [NM_201400]	**-1.158**
RPL31P4	Homo sapiens misc_RNA (LOC729646), miscRNA [XR_037308]	**-1.158**
LOC647500	Homo sapiens misc_RNA (LOC647500), miscRNA [XR_041430]	**-1.158**
UTP15	Homo sapiens UTP15, U3 small nucleolar ribonucleoprotein, homolog (UTP15), mRNA [NM_032175]	**-1.157**
DISP1	Homo sapiens dispatched homolog 1 (DISP1), mRNA [NM_032890]	**-1.155**
SSBP1	Homo sapiens single-stranded DNA binding protein 1 (SSBP1), mRNA [NM_003143]	**-1.155**
BAMBI	Homo sapiens BMP and activin membrane-bound inhibitor homolog (Xenopus laevis) (BAMBI), mRNA [NM_012342]	**-1.154**
SLC29A4	Homo sapiens solute carrier family 29 (nucleoside transporters), member 4 (SLC29A4), mRNA [NM_001040661]	**-1.154**
TMEM51	Homo sapiens transmembrane protein 51 (TMEM51), mRNA [NM_018022]	**-1.154**
ZCCHC10	Homo sapiens zinc finger, CCHC domain containing 10 (ZCCHC10), mRNA [NM_017665]	**-1.154**
ZNF43	Homo sapiens zinc finger protein 43 (ZNF43), mRNA [NM_003423]	**-1.154**
C8orf73	Homo sapiens chromosome 8 open reading frame 73 (C8orf73), mRNA [NM_001100878]	**-1.153**

GAA	Homo sapiens glucosidase, alpha; acid (GAA), mRNA [NM_000152]	**-1.151**
LOC100128485	Homo sapiens similar to conserved hypothetical protein (LOC100128485), mRNA [XM_001717043]	**-1.150**
PABPC3	Homo sapiens poly(A) binding protein, cytoplasmic 3 (PABPC3), mRNA [NM_030979]	**-1.150**
ANGPTL4	Homo sapiens angiopoietin-like 4 (ANGPTL4), mRNA [NM_139314]	**-1.149**
BAG4	BAG family molecular chaperone regulator 4 (BAG-4) [Source:UniProtKB/Swiss-Prot;Acc:O95429] [ENST00000287322]	**-1.149**
C10orf122	Homo sapiens chromosome 10 open reading frame 122 (C10orf122), mRNA [NM_001128202]	**-1.149**
C1orf38	Homo sapiens chromosome 1 open reading frame 38 (C1orf38), mRNA [NM_001039477]	**-1.149**
FAM89A	Homo sapiens family with sequence similarity 89, member A (FAM89A), mRNA [NM_198552]	**-1.149**
ZNF580	Homo sapiens zinc finger protein 580 (ZNF580), mRNA [NM_016202]	**-1.147**
C14orf109	Homo sapiens chromosome 14 open reading frame 109 (C14orf109), mRNA [NM_001098621]	**-1.145**
CD93	Homo sapiens CD93 molecule (CD93), mRNA [NM_012072]	**-1.145**
GGA3	Homo sapiens golgi associated, gamma adaptin ear containing, ARF binding protein 3 (GGA3), mRNA [NM_138619]	**-1.145**
LINGO2	Homo sapiens leucine rich repeat and Ig domain containing 2 (LINGO2), mRNA [NM_152570]	**-1.145**
PROC	Homo sapiens protein C (inactivator of coagulation factors Va and VIIIa) (PROC), mRNA [NM_000312]	**-1.145**
LOC100128328	Homo sapiens hypothetical protein LOC100128328 (LOC100128328), mRNA [XM_001715053]	**-1.143**
LOC100130332	Homo sapiens similar to PRO2474 (LOC100130332), mRNA [XM_001726675]	**-1.142**
TBX19	Homo sapiens T-box 19 (TBX19), mRNA [NM_005149]	**-1.142**
CASP9	Homo sapiens caspase 9, apoptosis-related cysteine peptidase (CASP9), mRNA [NM_001229]	**-1.141**
MMP25	Homo sapiens matrix metallopeptidase 25 (MMP25), mRNA [NM_022468]	**-1.141**
EZH2	Homo sapiens enhancer of zeste homolog 2 (Drosophila) (EZH2), mRNA [NM_004456]	**-1.140**
GNGT1	Homo sapiens guanine nucleotide binding protein (G protein), gamma transducing activity polypeptide 1 (GNGT1), mRNA [NM_021955]	**-1.138**
LOC285550	Homo sapiens cDNA FLJ42660 fis, clone BRAMY2010808. [AK124651]	**-1.138**
NDUFC2	Homo sapiens NADH dehydrogenase (ubiquinone) 1, subcomplex unknown, 2, 14.5kDa (NDUFC2), mRNA [NM_004549]	**-1.138**
RAP1B	Homo sapiens RAP1B, member of RAS oncogene family (RAP1B), mRNA [NM_015646]	**-1.138**
LOC400960	Homo sapiens cDNA FLJ32260 fis, clone PROST1000334. [AK056822]	**-1.138**
NOX4	Homo sapiens NADPH oxidase 4 (NOX4), mRNA [NM_016931]	**-1.138**
ABHD1	Homo sapiens abhydrolase domain containing 1, mRNA (cDNA clone	**-1.135**

	IMAGE:4812401), with apparent retained intron. [BC028378]	
ADO	Homo sapiens 2-aminoethanethiol (cysteamine) dioxygenase (ADO), mRNA [NM_032804]	**-1.135**
GNG2	Homo sapiens guanine nucleotide binding protein (G protein), gamma 2 (GNG2), mRNA [NM_053064]	**-1.135**
GFOD1	Homo sapiens glucose-fructose oxidoreductase domain containing 1 (GFOD1), mRNA [NM_018988]	**-1.132**
LOC650392	Homo sapiens hypothetical protein LOC650392, mRNA (cDNA clone IMAGE:5242623). [BC028099]	**-1.132**
MGC26597	Homo sapiens PIP5K1A pseudogene, mRNA (cDNA clone IMAGE:4828163). [BC028580]	**-1.132**
ZYG11A	Protein zyg-11 homolog A [Source:UniProtKB/Swiss-Prot;Acc:Q6WRX3] [ENST00000371528]	**-1.132**
CBR1	Homo sapiens carbonyl reductase 1 (CBR1), mRNA [NM_001757]	**-1.131**
OIP5	Homo sapiens Opa interacting protein 5 (OIP5), mRNA [NM_007280]	**-1.130**
IRAK1BP1	Homo sapiens interleukin-1 receptor-associated kinase 1 binding protein 1 (IRAK1BP1), mRNA [NM_001010844]	**-1.128**
SPAG4L	Homo sapiens sperm associated antigen 4-like (SPAG4L), mRNA [NM_080675]	**-1.128**
LOC100129720	full-length cDNA clone CS0DI044YN21 of Placenta Cot 25-normalized of Homo sapiens (human). [CR619772]	**-1.127**
TUBA8	Homo sapiens tubulin, alpha 8 (TUBA8), mRNA [NM_018943]	**-1.127**
GRK5	Homo sapiens G protein-coupled receptor kinase 5 (GRK5), mRNA [NM_005308]	**-1.126**
LOC100125556	Homo sapiens family with sequence similarity 86, member A pseudogene (LOC100125556), non-coding RNA [NR_024251]	**-1.126**
PPA1	Homo sapiens pyrophosphatase (inorganic) 1 (PPA1), mRNA [NM_021129]	**-1.126**
SCHIP1	Homo sapiens schwannomin interacting protein 1 (SCHIP1), mRNA [NM_014575]	**-1.126**
ST18	Homo sapiens suppression of tumorigenicity 18 (breast carcinoma) (zinc finger protein) (ST18), mRNA [NM_014682]	**-1.126**
TTBK1	Homo sapiens cDNA FLJ16105 fis, clone TESTI2025924, weakly similar to casein kinase I, epsilon isofoem (EC 2.7.1.-). [AK131217]	**-1.126**
LEPROTL1	Homo sapiens leptin receptor overlapping transcript-like 1 (LEPROTL1), mRNA [NM_015344]	**-1.125**
MGP	Homo sapiens matrix Gla protein (MGP), mRNA [NM_000900]	**-1.125**
ATN1	Homo sapiens atrophin 1 (ATN1), mRNA [NM_001007026]	**-1.123**
GPHB5	Homo sapiens glycoprotein hormone beta 5 (GPHB5), mRNA [NM_145171]	**-1.123**
PJA1	Homo sapiens praja ring finger 1 (PJA1), mRNA [NM_022368]	**-1.122**
CCRN4L	Homo sapiens CCR4 carbon catabolite repression 4-like (S. cerevisiae) (CCRN4L), mRNA [NM_012118]	**-1.120**
CCT6B	Homo sapiens chaperonin containing TCP1, subunit 6B (zeta 2) (CCT6B), mRNA [NM_006584]	**-1.120**
HS6ST3	Homo sapiens heparan sulfate 6-O-sulfotransferase 3 (HS6ST3), mRNA	**-1.120**

	[NM_153456]	
SERPINB9	Homo sapiens serpin peptidase inhibitor, clade B (ovalbumin), member 9 (SERPINB9), mRNA [NM_004155]	**-1.120**
C18orf18	Homo sapiens chromosome 18 open reading frame 18, mRNA (cDNA clone MGC:17515 IMAGE:3457488). [BC010538]	**-1.119**
LOC100128993	Homo sapiens similar to hCG2036572 (LOC100128993), mRNA [XM_001715896]	**-1.118**
hCG_1815491	Homo sapiens PNAS-108 mRNA, partial sequence. [AF275804]	**-1.117**
LOC728137	Homo sapiens similar to testis specific protein, Y-linked 1 (LOC728137), mRNA [NM_001077697]	**-1.116**
TBL2	Homo sapiens transducin (beta)-like 2 (TBL2), mRNA [NM_012453]	**-1.115**
ABCA10	Homo sapiens ATP-binding cassette, sub-family A (ABC1), member 10 (ABCA10), mRNA [NM_080282]	**-1.114**
CES2	Homo sapiens carboxylesterase 2 (intestine, liver) (CES2), mRNA [NM_198061]	**-1.114**
DMD	Homo sapiens dystrophin (DMD), mRNA [NM_004010]	**-1.114**
LOC202451	Homo sapiens, clone IMAGE:2899977, mRNA. [BC022980]	**-1.114**
FLJ22662	Homo sapiens hypothetical protein FLJ22662 (FLJ22662), mRNA [NM_024829]	**-1.113**
LRFN3	Homo sapiens leucine rich repeat and fibronectin type III domain containing 3 (LRFN3), mRNA [NM_024509]	**-1.113**
NUDT6	Homo sapiens nudix (nucleoside diphosphate linked moiety X)-type motif 6 (NUDT6), mRNA [NM_198041]	**-1.113**
ACP5	Homo sapiens acid phosphatase 5, tartrate resistant (ACP5), mRNA [NM_001611]	**-1.112**
AKAP1	Homo sapiens A kinase (PRKA) anchor protein 1 (AKAP1),mRNA [NM_003488]	**-1.112**
C13orf15	Homo sapiens chromosome 13 open reading frame 15 (C13orf15), mRNA [NM_014059]	**-1.112**
KRT3	Homo sapiens keratin 3 (KRT3), mRNA [NM_057088]	**-1.111**
MPP1	Homo sapiens membrane protein, palmitoylated 1, 55kDa (MPP1), mRNA [NM_002436]	**-1.110**
SLC25A24	Homo sapiens solute carrier family 25,member 24 (SLC25A24), mRNA [NM_213651]	**-1.110**
CHAF1B	Homo sapiens chromatin assembly factor 1, subunit B (p60) (CHAF1B), mRNA [NM_005441]	**-1.109**
ZNF706	Homo sapiens zinc finger protein 706 (ZNF706), mRNA [NM_001042510]	**-1.109**
IDI2	Homo sapiens isopentenyl-diphosphate delta isomerase 2 (IDI2), mRNA [NM_033261]	**-1.108**
SPRN	Homo sapiens shadow of prion protein homolog (zebrafish) (SPRN), mRNA [NM_001012508]	**-1.107**
LOC100133528	Homo sapiens similar to HIG1 domain family, member 1A (LOC100133528), mRNA [XM_001721514]	**-1.106**

ZNF268	H.sapiens HZF3 mRNA for zinc finger protein. [X78926]	**-1.106**
OR7E24	Homo sapiens olfactory receptor, family 7, subfamily E, member 24 (OR7E24), mRNA [NM_001079935]	**-1.105**
C4orf12	Homo sapiens cDNA FLJ42672 fis, clone BRAMY2026533. [AK124663]	**-1.105**
FGF7	Homo sapiens fibroblast growth factor 7 (keratinocyte growth factor) (FGF7), mRNA [NM_002009]	**-1.104**
RAB15	Homo sapiens RAB15, member RAS onocogene family (RAB15), mRNA [NM_198686]	**-1.104**
ARHGAP12	Homo sapiens Rho GTPase activating protein 12 (ARHGAP12), mRNA [NM_018287]	**-1.103**
COL15A1	Homo sapiens collagen, type XV, alpha 1 (COL15A1), mRNA [NM_001855]	**-1.100**
MGC3771	Homo sapiens hypothetical LOC81854 (MGC3771), non-coding RNA [NR_024166]	**-1.100**
MTL5	Homo sapiens metallothionein-like 5, testis-specific (tesmin) (MTL5), mRNA [NM_004923]	**-1.100**
RASGRP4	Homo sapiens RAS guanyl releasing protein 4 (RASGRP4), mRNA [NM_170604]	**-1.100**
SCFD1	Homo sapiens sec1 family domain containing 1 (SCFD1), mRNA [NM_016106]	**-1.100**
LOC100131971	Homo sapiens similar to 40S ribosomal protein S26 (LOC100131971), mRNA [XM_001720153]	**-1.099**
KIAA1211	Homo sapiens KIAA1211 protein (KIAA1211), mRNA [NM_020722]	**-1.098**
STK40	Homo sapiens serine/threonine kinase 40 (STK40), mRNA [NM_032017]	**-1.096**
TRAF5	Homo sapiens TNF receptor-associated factor 5 (TRAF5), mRNA [NM_004619]	**-1.096**
USP36	Homo sapiens ubiquitin specific peptidase 36, mRNA (cDNA clone IMAGE:5756922). [BC038983]	**-1.096**
ARPC3	Homo sapiens actin related protein 2/3 complex, subunit 3, 21kDa (ARPC3), mRNA [NM_005719]	**-1.095**
BDKRB2	Homo sapiens bradykinin receptor B2 (BDKRB2), mRNA [NM_000623]	**-1.095**
LOC400657	Homo sapiens hypothetical LOC400657 (LOC400657), non-coding RNA [NR_024484]	**-1.095**
HBA2	Homo sapiens hemoglobin, alpha 2 (HBA2), mRNA [NM_000517]	**-1.094**
LAT2	Linker for activation of T-cells family member 2 (Non-T-cell activation linker)(Linker for activation of B-cells) [Source:UniProtKB/Swiss-Prot;Acc: Q9GZY6] [ENST00000398475]	**-1.094**
ZNF557	Homo sapiens zinc finger protein 557 (ZNF557), mRNA [NM_024341]	**-1.093**
LOC728537	Homo sapiens cDNA clone IMAGE:5271446. [BC039374]	**-1.092**
SH3TC2	Homo sapiens SH3 domain and tetratricopeptide repeats 2 (SH3TC2), mRNA [NM_024577]	**-1.091**
HERPUD1	Homo sapiens homocysteine-inducible, endoplasmic reticulum stress-inducible, ubiquitin-like domain member 1 (HERPUD1), mRNA [NM_014685]	**-1.090**
RORA	Homo sapiens RAR-related orphan receptor A (RORA), mRNA [NM_134260]	**-1.090**
ZFP36L2	Homo sapiens zinc finger protein 36, C3H type-like 2 (ZFP36L2), mRNA	**-1.090**

	[NM_006887]	
C10orf10	Homo sapiens chromosome 10 open reading frame 10 (C10orf10), mRNA [NM_007021]	**-1.089**
PRG4	Homo sapiens proteoglycan 4 (PRG4), mRNA [NM_005807]	**-1.089**
TMEM74	Homo sapiens transmembrane protein 74 (TMEM74), mRNA [NM_153015]	**-1.089**
ZNF180	Homo sapiens zinc finger protein 180 (ZNF180), mRNA [NM_013256]	**-1.089**
BEGAIN	Homo sapiens brain-enriched guanylate kinase-associated homolog (rat) (BEGAIN), mRNA [NM_020836]	**-1.088**
DKFZp564N2472	Homo sapiens hypothetical protein DKFZp564N2472 (DKFZp564N2472), mRNA [NM_182595]	**-1.088**
FAM131C	Homo sapiens family with sequence similarity 131, member C (FAM131C), mRNA [NM_182623]	**-1.088**
LOC387763	Protein Ag2 homolog [Source:UniProtKB/Swiss-Prot;Acc:Q7Z7L8] [ENST00000339446]	**-1.088**
ARMCX2	Homo sapiens armadillo repeat containing, X-linked 2 (ARMCX2), mRNA [NM_014782]	**-1.087**
C7orf28B	Homo sapiens chromosome 7 open reading frame 28B (C7orf28B), mRNA [NM_198097]	**-1.087**
MICAL2	Homo sapiens microtubule associated monoxygenase, calponin and LIM domain containing 2 (MICAL2), mRNA [NM_014632]	**-1.087**
PAG1	Homo sapiens phosphoprotein associated with glycosphingolipid microdomains 1 (PAG1), mRNA [NM_018440]	**-1.087**
TNFAIP1	Homo sapiens tumor necrosis factor, alpha-induced protein 1 (endothelial) (TNFAIP1), mRNA [NM_021137]	**-1.087**
WDR72	Homo sapiens WD repeat domain 72 (WDR72), mRNA [NM_182758]	**-1.087**
KCNAB3	Homo sapiens potassium voltage-gated channel, shaker-related subfamily, beta member 3 (KCNAB3), mRNA [NM_004732]	**-1.086**
ARMETL1	Homo sapiens arginine-rich, mutated in early stage tumors-like 1 (ARMETL1), mRNA [NM_001029954]	**-1.085**
RAB11FIP4	Homo sapiens RAB11 family interacting protein 4 (class II) (RAB11FIP4), mRNA [NM_032932]	**-1.085**
SEMA6B	Homo sapiens sema domain, transmembrane domain (TM), and cytoplasmic domain, (semaphorin) 6B (SEMA6B), mRNA [NM_032108]	**-1.084**
FER1L4	Homo sapiens fer-1-like 4 (C. elegans) (FER1L4), non-coding RNA [NR_024377]	**-1.083**
THAP1	Homo sapiens THAP domain containing, apoptosis associated protein 1 (THAP1), mRNA [NM_018105]	**-1.081**
TMEM170B	Homo sapiens transmembrane protein 170B (TMEM170B), mRNA [NM_001100829]	**-1.081**
FGGY	Homo sapiens FGGY carbohydrate kinase domain containing (FGGY), mRNA [NM_018291]	**-1.080**
BEST2	Homo sapiens bestrophin 2 (BEST2), mRNA [NM_017682]	**-1.078**
SOLH	Homo sapiens small optic lobes homolog (Drosophila) (SOLH), mRNA	**-1.077**

	[NM_005632]	
SPATA2	Homo sapiens spermatogenesis associated 2 (SPATA2), mRNA [NM_006038]	**-1.076**
NFKBIA	Homo sapiens nuclear factor of kappa light polypeptide gene enhancer in B-cells inhibitor, alpha (NFKBIA), mRNA [NM_020529]	**-1.075**
SEMA6C	Homo sapiens sema domain, transmembrane domain (TM), and cytoplasmic domain, (semaphorin) 6C (SEMA6C), mRNA [NM_030913]	**-1.075**
CTBS	Homo sapiens chitobiase, di-N-acetyl- (CTBS), mRNA [NM_004388]	**-1.075**
APLN	Homo sapiens apelin (APLN), mRNA [NM_017413]	**-1.074**
C6orf155	Homo sapiens cDNA FLJ13189 fis, clone NT2RP3004253. [AK023251]	**-1.074**
CASP5	Homo sapiens caspase 5, apoptosis-related cysteine peptidase (CASP5), mRNA [NM_004347]	**-1.074**
SRD5A3	Homo sapiens steroid 5 alpha-reductase 3 (SRD5A3), mRNA [NM_024592]	**-1.074**
ZNF697	Homo sapiens zinc finger protein 697 (ZNF697), mRNA [NM_001080470]	**-1.074**
EPCAM	Homo sapiens epithelial cell adhesion molecule (EPCAM), mRNA [NM_002354]	**-1.073**
GTPBP3	Homo sapiens GTP binding protein 3 (mitochondrial) (GTPBP3), mRNA [NM_133644]	**-1.073**
LOC100130967	Putative uncharacterized protein C6orf99 [Source:UniProtKB/Swiss-Prot; Acc:Q4VX62] [ENST00000367073]	**-1.072**
HEBP1	Homo sapiens heme binding protein 1 (HEBP1), mRNA [NM_015987]	**-1.071**
HEATR5A	Homo sapiens HEAT repeat containing 5A (HEATR5A), mRNA [NM_015473]	**-1.070**
LOC346887	Homo sapiens similar to solute carrier family 16 (monocarboxylic acid transporters), member 14, mRNA (cDNA clone IMAGE:5726657). [BC040619]	**-1.070**
SYT5	Homo sapiens synaptotagmin V (SYT5), mRNA [NM_003180]	**-1.070**
KIAA0329	Homo sapiens KIAA0329 (KIAA0329), mRNA [NM_014844]	**-1.069**
CLOCK	Homo sapiens clock homolog (mouse) (CLOCK), mRNA [NM_004898]	**-1.068**
LOC100127980	Homo sapiens cDNA FLJ33508 fis, clone BRAMY2005094. [AK090827]	**-1.067**
IPPK	Homo sapiens inositol 1,3,4,5,6-pentakisphosphate 2-kinase (IPPK), mRNA [NM_022755]	**-1.066**
KLHL29	Homo sapiens cDNA FLJ37281 fis, clone BRAMY2013200. [AK094600]	**-1.065**
CLIC2	Homo sapiens chloride intracellular channel 2 (CLIC2), mRNA [NM_001289]	**-1.064**
SNX6	Homo sapiens sorting nexin 6 (SNX6), mRNA [NM_021249]	**-1.064**
UNQ5840	Homo sapiens clone DNA139632 VGSA5840 (UNQ5840) mRNA. [AY358772]	**-1.064**
LOC645676	Homo sapiens cDNA FLJ44595 fis, clone BLADE2004849. [AK126559]	**-1.063**
C2orf67	Homo sapiens chromosome 2 open reading frame 67 (C2orf67), mRNA [NM_152519]	**-1.062**
RBPMS2	Homo sapiens RNA binding protein with multiple splicing 2 (RBPMS2), mRNA [NM_194272]	**-1.062**
SLC44A3	Homo sapiens solute carrier family 44, member 3 (SLC44A3), mRNA [NM_152369]	**-1.062**
IRF2BP1	Homo sapiens interferon regulatory factor 2 binding protein 1 (IRF2BP1), mRNA [NM_015649]	**-1.061**
LOC100128164	Homo sapiens mRNA; cDNA DKFZp313A137 (from clone DKFZp313A137).	**-1.061**

	[AL833309]	
ATP8B3	Homo sapiens cDNA FLJ43235 fis, clone HCHON2004007, highly similar to Potential phospholipid-transporting ATPase IK (EC 3.6.3.13). [AK125225]	**-1.060**
GLRB	Homo sapiens glycine receptor, beta (GLRB), mRNA [NM_000824]	**-1.059**
GNG11	Homo sapiens guanine nucleotide binding protein (G protein), gamma 11 (GNG11), mRNA [NM_004126]	**-1.059**
LOC644450	Homo sapiens hypothetical protein LOC644450, mRNA (cDNA clone IMAGE:4606942). [BC022881]	**-1.059**
THBD	Homo sapiens thrombomodulin (THBD), mRNA [NM_000361]	**-1.059**
DNM3	Homo sapiens dynamin 3 (DNM3), mRNA [NM_015569]	**-1.058**
FAM136A	Homo sapiens family with sequence similarity 136, member A (FAM136A), mRNA [NM_032822]	**-1.058**
ORMDL1	Homo sapiens ORM1-like 1 (S. cerevisiae) (ORMDL1), mRNA [NM_016467]	**-1.058**
RIMBP3	Homo sapiens RIMS binding protein 3 (RIMBP3), mRNA [NM_015672]	**-1.058**
WFDC11	Homo sapiens WAP four-disulfide core domain 11 (WFDC11), mRNA [NM_147197]	**-1.058**
ZNF107	Homo sapiens zinc finger protein 107 (ZNF107), mRNA [NM_016220]	**-1.057**
C8orf83	Homo sapiens chromosome 8 open reading frame 83 (C8orf83), non-coding RNA [NR_015339]	**-1.057**
UGP2	Homo sapiens UDP-glucose pyrophosphorylase 2 (UGP2), mRNA [NM_006759]	**-1.057**
LYRM5	Homo sapiens LYR motif containing 5 (LYRM5), mRNA [NM_001001660]	**-1.056**
HSPC159	Homo sapiens galectin-related protein (HSPC159), mRNA [NM_014181]	**-1.056**
XIRP2	Homo sapiens xin actin-binding repeat containing 2 (XIRP2), mRNA [NM_152381]	**-1.054**
C5orf36	Homo sapiens chromosome 5 open reading frame 36 (C5orf36), mRNA [NM_173665]	**-1.053**
LOC100131053	Homo sapiens cDNA FLJ38245 fis, clone FCBBF2007186. [AK095564]	**-1.053**
HIC2	Homo sapiens hypermethylated in cancer 2 (HIC2), mRNA [NM_015094]	**-1.052**
SGTB	Homo sapiens small glutamine-rich tetratricopeptide repeat (TPR)-containing, beta (SGTB), mRNA [NM_019072]	**-1.052**
FAM179A	Homo sapiens family with sequence similarity 179, member A (FAM179A), mRNA [NM_199280]	**-1.052**
C3orf26	Homo sapiens chromosome 3 open reading frame 26 (C3orf26), mRNA [NM_032359]	**-1.051**
TAF12	Homo sapiens TAF12 RNA polymerase II, TATA box binding protein (TBP)-associated factor, (TAF12), mRNA [NM_005644]	**-1.051**
RNF2	Homo sapiens ring finger protein 2 (RNF2), mRNA [NM_007212]	**-1.049**
SLC25A22	Homo sapiens solute carrier family 25 (mitochondrial carrier: glutamate), member 22 (SLC25A22), mRNA [NM_024698]	**-1.049**
AFAP1	Homo sapiens actin filament associated protein 1 (AFAP1), mRNA [NM_001134647]	**-1.048**
EAF2	Homo sapiens ELL associated factor 2 (EAF2), mRNA [NM_018456]	**-1.048**

CCRK	Homo sapiens cell cycle related kinase (CCRK), mRNA [NM_001039803]	**-1.047**
IKZF2	Homo sapiens IKAROS family zinc finger 2 (Helios) (IKZF2), mRNA [NM_001079526]	**-1.047**
LOC730107	Homo sapiens similar to Glycine cleavage system H protein, mitochondrial (LOC730107), mRNA [XM_001721064]	**-1.047**
ZNF138	Homo sapiens zinc finger protein 138 (ZNF138), mRNA [NM_006524]	**-1.047**
SWAP70	Homo sapiens SWAP-70 protein, mRNA (cDNA clone IMAGE:2900736), containing frame-shift errors. [BC000134]	**-1.046**
ZNF480	Homo sapiens zinc finger protein 480 (ZNF480), mRNA [NM_144684]	**-1.046**
KATNAL2	Homo sapiens katanin p60 subunit A-like 2 (KATNAL2), mRNA [NM_031303]	**-1.046**
ALDH6A1	Homo sapiens aldehyde dehydrogenase 6 family, member A1 (ALDH6A1), mRNA [NM_005589]	**-1.045**
CHRNB3	Homo sapiens cholinergic receptor, nicotinic, beta 3 (CHRNB3), mRNA [NM_000749]	**-1.045**
EPN2	Homo sapiens epsin 2 (EPN2), mRNA [NM_014964]	**-1.045**
RASL10A	Homo sapiens RAS-like, family 10, member A (RASL10A), mRNA [NM_001007279]	**-1.045**
ENY2	Homo sapiens cDNA FLJ38332 fis, clone FCBBF3025528. [AK095651]	**-1.045**
STEAP1	Homo sapiens six transmembrane epithelial antigen of the prostate 1 (STEAP1), mRNA [NM_012449]	**-1.044**
PLA2R1	Human 180 kDa transmembrane PLA2 receptor mRNA. [U17033]	**-1.044**
ZC3HAV1L	Homo sapiens cDNA FLJ40837 fis, clone TRACH2013495. [AK098156]	**-1.043**
STYK1	Homo sapiens serine/threonine/tyrosine kinase 1 (STYK1), mRNA [NM_018423]	**-1.042**
C9orf127	Homo sapiens chromosome 9 open reading frame 127 (C9orf127), mRNA [NM_016446]	**-1.041**
GBP5	Homo sapiens guanylate binding protein 5 (GBP5), mRNA [NM_052942]	**-1.041**
GRRP1	Homo sapiens glycine/arginine rich protein 1 (GRRP1), mRNA [NM_024869]	**-1.041**
ZNF630	Homo sapiens zinc finger protein 630 (ZNF630), mRNA [NM_001037735]	**-1.041**
F8A1	Homo sapiens coagulation factor VIII-associated (intronic transcript) 1 (F8A1), mRNA [NM_012151]	**-1.040**
MCART2	Homo sapiens mitochondrial carrier triple repeat 2 (MCART2), mRNA [NM_001034172]	**-1.040**
PRCP	Homo sapiens prolylcarboxypeptidase (angiotensinase C) (PRCP),mRNA [NM_199418]	**-1.040**
RFX1	Homo sapiens regulatory factor X, 1 (influences HLA class II expression) (RFX1), mRNA [NM_002918]	**-1.040**
TBX1	Homo sapiens T-box 1 (TBX1), mRNA [NM_080647]	**-1.039**
C17orf58	Homo sapiens chromosome 17 open reading frame 58 (C17orf58), mRNA [NM_181655]	**-1.038**
C2orf3	Homo sapiens chromosome 2 open reading frame 3 (C2orf3), mRNA [NM_003203]	**-1.037**
DKFZP434B061	Homo sapiens mRNA; cDNA DKFZp434B061 (from clone DKFZp434B061);.	**-1.037**

	[AL117481]	
LOC257039	Homo sapiens similar to hCG2040268 (LOC257039), mRNA [XM_172230]	**-1.037**
XPA	Homo sapiens xeroderma pigmentosum, complementation group A (XPA), mRNA [NM_000380]	**-1.036**
FAM71D	Homo sapiens family with sequence similarity 71, member D (FAM71D), mRNA [NM_173526]	**-1.035**
PIH1D2	Homo sapiens PIH1 domain containing 2 (PIH1D2), mRNA [NM_138789]	**-1.035**
PLAC8	Homo sapiens placenta-specific 8 (PLAC8), mRNA [NM_016619]	**-1.035**
ZNF462	Homo sapiens mRNA; cDNA DKFZp762N2316 (from clone DKFZp762N2316). [AL359561]	**-1.035**
C1orf97	Homo sapiens cDNA FLJ27348 fis, clone TST04156. [AK130858]	**-1.034**
CROCCL1	Homo sapiens ciliary rootlet coiled-coil, rootletin-like 1, mRNA (cDNA clone MGC:12760 IMAGE:4111573). [BC006312]	**-1.034**
NID2	Homo sapiens nidogen 2 (osteonidogen) (NID2), mRNA [NM_007361]	**-1.034**
RTCD1	Homo sapiens RNA terminal phosphate cyclase domain 1 (RTCD1), mRNA [NM_003729]	**-1.034**
TBX3	Homo sapiens T-box 3 (TBX3), mRNA [NM_016569]	**-1.034**
AEBP1	Homo sapiens AE binding protein 1 (AEBP1), mRNA [NM_001129]	**-1.033**
C15orf23	Homo sapiens chromosome 15 open reading frame 23 (C15orf23), mRNA [NM_001142761]	**-1.033**
PLEKHG4	Homo sapiens pleckstrin homology domain containing, family G (with RhoGef domain) member 4 (PLEKHG4), mRNA [NM_015432]	**-1.033**
RABEPK	Homo sapiens Rab9 effector protein with kelch motifs (RABEPK), mRNA [NM_005833]	**-1.033**
PITPNM2	Homo sapiens phosphatidylinositol transfer protein, membrane-associated 2 (PITPNM2), mRNA [NM_020845]	**-1.032**
GSTA2	Homo sapiens glutathione S-transferase alpha 2 (GSTA2), mRNA [NM_000846]	**-1.031**
HSPB2	Homo sapiens heat shock 27kDa protein 2 (HSPB2), mRNA [NM_001541]	**-1.031**
SLC5A3	Homo sapiens solute carrier family 5 (sodium/myo-inositol cotransporter), member 3 (SLC5A3), mRNA [NM_006933]	**-1.031**
RG9MTD1	Homo sapiens RNA (guanine-9-) methyltransferase domain containing 1 (RG9MTD1), mRNA [NM_017819]	**-1.029**
ZNF83	Homo sapiens zinc finger protein 83 (ZNF83), mRNA [NM_018300]	**-1.029**
FAM13C1	Homo sapiens family with sequence similarity 13, member C1 (FAM13C1), mRNA [NM_001001971]	**-1.028**
FKBP2	Homo sapiens FK506 binding protein 2, 13kDa (FKBP2), mRNA [NM_004470]	**-1.028**
LOC338799	Homo sapiens hypothetical locus LOC338799 (LOC338799), non-coding RNA [NR_002809]	**-1.028**
TCF7L2	Homo sapiens transcription factor 7-like 2 (T-cell specific, HMG-box) (TCF7L2), mRNA [NM_030756]	**-1.028**
TTRAP	Homo sapiens TRAF and TNF receptor associated protein (TTRAP), mRNA [NM_016614]	**-1.028**
FAAH2	Homo sapiens fatty acid amide hydrolase 2 (FAAH2), mRNA [NM_174912]	**-1.027**

GLRX	Homo sapiens glutaredoxin (thioltransferase) (GLRX), mRNA [NM_002064]	**-1.027**
FGFR1OP	Homo sapiens FGFR1 oncogene partner (FGFR1OP), mRNA [NM_007045]	**-1.026**
SHD	Homo sapiens Src homology 2 domain containing transforming protein D (SHD), mRNA [NM_020209]	**-1.026**
JAKMIP3	Homo sapiens janus kinase and microtubule interacting protein 3 (JAKMIP3), mRNA [NM_001105521]	**-1.025**
NOS1	Homo sapiens nitric oxide synthase 1 (neuronal) (NOS1), mRNA [NM_000620]	**-1.025**
NANOS1	Homo sapiens nanos homolog 1 (Drosophila) (NANOS1), mRNA [NM_199461]	**-1.023**
C4orf15	Homo sapiens cDNA FLJ52073 complete cds. [AK293948]	**-1.023**
PCSK1	Homo sapiens proprotein convertase subtilisin/kexin type 1 (PCSK1), mRNA [NM_000439]	**-1.023**
BZW2	Homo sapiens basic leucine zipper and W2 domains 2 (BZW2), mRNA [NM_014038]	**-1.022**
GSTT2	Homo sapiens glutathione S-transferase theta 2 (GSTT2), mRNA [NM_000854]	**-1.022**
VCY	Homo sapiens variable charge, Y-linked (VCY), mRNA [NM_004679]	**-1.022**
BPNT1	Homo sapiens 3'(2'), 5'-bisphosphate nucleotidase 1 (BPNT1), mRNA [NM_006085]	**-1.021**
C14orf128	Homo sapiens chromosome 14 open reading frame 128, mRNA (cDNA clone MGC:15504 IMAGE:2990071). [BC007251]	**-1.020**
TMEM64	Homo sapiens transmembrane protein 64 (TMEM64), mRNA [NM_001008495]	**-1.020**
C1orf113	Homo sapiens chromosome 1 open reading frame 113 (C1orf113), mRNA [NM_024676]	**-1.019**
MGC16121	Homo sapiens hypothetical protein MGC16121, mRNA (cDNA clone IMAGE:3627113). [BC007360]	**-1.019**
STON1	Homo sapiens stonin 1 (STON1), mRNA [NM_006873]	**-1.019**
ZNF573	Homo sapiens zinc finger protein 573 (ZNF573), mRNA [NM_152360]	**-1.019**
IQCD	Homo sapiens IQ motif containing D (IQCD), mRNA [NM_138451]	**-1.018**
LOC645294	Homo sapiens misc_RNA (LOC645294), miscRNA [XR_019042]	**-1.018**
PRDM13	Homo sapiens PR domain containing 13 (PRDM13), mRNA [NM_021620]	**-1.017**
BANK1	Homo sapiens B-cell scaffold protein with ankyrin repeats 1 (BANK1), mRNA [NM_017935]	**-1.016**
DCPS	Homo sapiens decapping enzyme, scavenger (DCPS), mRNA [NM_014026]	**-1.016**
SYT12	Homo sapiens synaptotagmin XII (SYT12), mRNA [NM_177963]	**-1.016**
ZNF30	Homo sapiens zinc finger protein 30 (ZNF30), mRNA [NM_194325]	**-1.016**
CGNL1	Homo sapiens cingulin-like 1 (CGNL1), mRNA [NM_032866]	**-1.015**
FLJ20712	Homo sapiens cDNA FLJ20712 fis, clone HUV01027. [AK000719]	**-1.015**
SULF1	Homo sapiens sulfatase 1 (SULF1), mRNA [NM_015170]	**-1.015**
BRAF	Homo sapiens v-raf murine sarcoma viral oncogene homolog B1 (BRAF), mRNA [NM_004333]	**-1.013**
FAM175B	Homo sapiens family with sequence similarity 175, member B (FAM175B), mRNA [NM_032182]	**-1.013**
LOC645726	Homo sapiens misc_RNA (LOC645726), miscRNA [XR_018230]	**-1.013**

KIAA1377	Homo sapiens KIAA1377 (KIAA1377), mRNA [NM_020802]	**-1.012**
SYT17	Homo sapiens synaptotagmin XVII (SYT17), mRNA [NM_016524]	**-1.012**
TBC1D8	Homo sapiens cDNA FLJ40805 fis, clone TRACH2009060. [AK098124]	**-1.012**
ARL6IP5	Homo sapiens ADP-ribosylation-like factor 6 interacting protein 5 (ARL6IP5), mRNA [NM_006407]	**-1.012**
DCP1A	Homo sapiens DCP1 decapping enzyme homolog A (S. cerevisiae) (DCP1A), mRNA [NM_018403]	**-1.012**
DCUN1D3	Homo sapiens DCN1, defective in cullin neddylation 1, domain containing 3 (S. cerevisiae) (DCUN1D3), mRNA [NM_173475]	**-1.010**
DNAJC18	Homo sapiens DnaJ (Hsp40) homolog, subfamily C, member 18 (DNAJC18), mRNA [NM_152686]	**-1.009**
FAM149B1	Protein FAM149B1 [Source:UniProtKB/ Swiss-Prot; Acc:Q96BN6] [ENST00000242505]	**-1.008**
SUSD1	Homo sapiens sushi domain containing 1 (SUSD1), mRNA [NM_022486]	**-1.008**
ZNF409	Homo sapiens mRNA for KIAA1056 protein. [AB028979]	**-1.008**
C10orf57	Homo sapiens chromosome 10 open reading frame 57 (C10orf57), mRNA [NM_025125]	**-1.007**
PRDM10	Homo sapiens PR domain containing 10 (PRDM10), mRNA [NM_199437]	**-1.007**
TNS1	Homo sapiens tensin 1 (TNS1), mRNA [NM_022648]	**-1.007**
ZNF350	Homo sapiens zinc finger protein 350 (ZNF350), mRNA [NM_021632]	**-1.006**
ZNF692	Homo sapiens zinc finger protein 692 (ZNF692), mRNA [NM_017865]	**-1.006**
INPP5E	Homo sapiens inositol polyphosphate-5-phosphatase, 72 kDa (INPP5E), mRNA [NM_019892]	**-1.005**
UCHL3	Homo sapiens ubiquitin carboxyl-terminal esterase L3 (ubiquitin thiolesterase) (UCHL3), mRNA [NM_006002]	**-1.005**
ZNF165	Homo sapiens zinc finger protein 165 (ZNF165), mRNA [NM_003447]	**-1.005**
NCRNA00081	Homo sapiens non-protein coding RNA 81 (NCRNA00081), non-coding RNA [NR_024140]	**-1.005**
MYNN	Homo sapiens myoneurin (MYNN), mRNA [NM_018657]	**-1.004**
KPNA5	Homo sapiens karyopherin alpha 5 (importin alpha 6) (KPNA5), mRNA [NM_002269]	**-1.004**
SLC48A1	Homo sapiens cDNA FLJ20489 fis, clone KAT08285. [AK000496]	**-1.004**
DUSP18	Homo sapiens dual specificity phosphatase 18 (DUSP18), mRNA [NM_152511]	**-1.003**
FAM102A	Homo sapiens family with sequence similarity 102, member A (FAM102A), mRNA [NM_001035254]	**-1.003**
SMARCD3	Homo sapiens SWI/SNF related, matrix associated,subfamily d, member 3 (SMARCD3), mRNA [NM_003078]	**-1.003**
MYSM1	Homo sapiens mRNA for KIAA1915 protein. [AB067502]	**-1.002**
TBCE	Homo sapiens tubulin folding cofactor E (TBCE), mRNA [NM_001079515]	**-1.002**
RBPJL	Homo sapiens recombination signal binding protein for immunoglobulin kappa J region-like (RBPJL), mRNA [NM_014276]	**-1.001**
USP6NL	Homo sapiens USP6 N-terminal like, mRNA (cDNA clone IMAGE:4047207). [BC010351]	**-1.001**

FLJ20674	Homo sapiens hypothetical protein FLJ20674 (FLJ20674), mRNA [NM_019086]	**-1.001**
APC2	Homo sapiens adenomatosis polyposis coli 2 (APC2), mRNA [NM_005883]	**-1.000**
FAM49A	Homo sapiens family with sequence similarity 49, member A (FAM49A), mRNA [NM_030797]	**-1.000**
GIN1	Homo sapiens gypsy retrotransposon integrase 1 (GIN1), mRNA [NM_017676]	**-1.000**
LOC729338	Homo sapiens CETN4 pseudogene (LOC729338), non-coding RNA [NR_024041]	**-1.000**
MED7	Homo sapiens mediator complex subunit 7 (MED7), mRNA [NM_004270]	**-1.000**
RIMKLA	Homo sapiens ribosomal modification protein rimK-like family member A (RIMKLA), mRNA [NM_173642]	**-1.000**
