# Supplementary material for: Genotoxicity and molecular response of silver nanoparticle (NP)-based hydrogel
Source: J Nanobiotechnology. 2012 May 1;10:16. doi: 10.1186/1477-3155-10-16 (PMC3430588; doi:10.1186/1477-3155-10-16)
Supplement: Additional file 7 — Up-regulated genes in cells exposed to Hydrogel for 48h. Fold-change is logarithmic ratio (log2 ratio) to expression level in control. [file 1477-3155-10-16-S7.pdf]

Additional File 7. Up-regulated genes in cells exposed to Hydrogel for 48h. Fold-change is logarithmic ratio (log_2 ratio) to expression level in control.

GeneName	Description	Fold-change (log_2 ratio)
RGSL1	Homo sapiens mRNA; cDNA DKFZp434E169 (from clone DKFZp434E169). [AL136902]	**6.644**
PRKAG3	Homo sapiens protein kinase, AMP-activated, gamma 3 non-catalytic subunit (PRKAG3), mRNA [NM_017431]	**5.376**
GDF2	Homo sapiens growth differentiation factor 2 (GDF2), mRNA [NM_016204]	**5.177**
ANKRD31	Ankyrin repeat domain-containing protein 31 [Source:UniProtKB/Swiss-Prot;Acc:Q8N7Z5] [ENST00000274361]	**4.491**
POF1B	Homo sapiens premature ovarian failure, 1B (POF1B), mRNA [NM_024921]	**3.773**
TMPRSS9	Homo sapiens cDNA FLJ16193 fis, clone BRTHA2018011, weakly similar to Epithin (EC 3.4.21.-). [AK131261]	**3.328**
LOC646976	Homo sapiens cDNA FLJ38763 fis, clone KIDNE2014119. [AK096082]	**3.100**
GAGE3	Human GAGE-3 protein mRNA, complete cds. [U19144]	**2.719**
ADAMTS9	Homo sapiens ADAM metallopeptidase with thrombospondin type 1 motif, 9 (ADAMTS9), mRNA [NM_182920]	**2.701**
SPATA18	Homo sapiens spermatogenesis associated 18 homolog (rat) (SPATA18), mRNA [NM_145263]	**2.476**
SPEF1	Homo sapiens sperm flagellar 1 (SPEF1), mRNA [NM_015417]	**2.331**
TCF15	Homo sapiens transcription factor 15 (basic helix-loop-helix) (TCF15), mRNA [NM_004609]	**1.941**
C9orf33	Homo sapiens HSFE-1 mRNA, partial cds. [AF072164]	**1.676**
C15orf21	Homo sapiens chromosome 15 open reading frame 21 (C15orf21), non-coding RNA [NR_022014]	**1.582**
C4B	Homo sapiens complement component 4B (Chido blood group) (C4B), mRNA [NM_001002029]	**1.533**
VCX3A	Homo sapiens variable charge, X-linked 3A (VCX3A), mRNA [NM_016379]	**1.476**
MPL	Homo sapiens myeloproliferative leukemia virus oncogene (MPL), mRNA [NM_005373]	**1.437**
P2RY6	Homo sapiens pyrimidinergic receptor P2Y, G-protein coupled, 6 (P2RY6), mRNA [NM_176798]	**1.406**
ZNF619	Homo sapiens zinc finger protein 619 (ZNF619), mRNA [NM_173656]	**1.372**
VCX	Homo sapiens variable charge, X-linked (VCX), mRNA [NM_013452]	**1.343**
VCX2	Homo sapiens variable charge, X-linked 2 (VCX2), mRNA [NM_016378]	**1.335**
BASP1	Homo sapiens brain abundant, membrane attached signal protein 1 (BASP1), mRNA [NM_006317]	**1.287**
CLEC4A	Homo sapiens C-type lectin domain family 4, member A (CLEC4A), mRNA [NM_016184]	**1.279**
FOSB	Homo sapiens FBJ murine osteosarcoma viral oncogene homolog B (FOSB), mRNA [NM_006732]	**1.272**
CYP1A1	Homo sapiens cytochrome P450, family 1, subfamily A, polypeptide 1 (CYP1A1), mRNA [NM_000499]	**1.271**
CRAT	Homo sapiens carnitine acetyltransferase (CRAT), mRNA [NM_000755]	**1.270**
RPS16	Homo sapiens ribosomal protein S16 (RPS16), mRNA [NM_001020]	**1.269**

LOC100132673	Homo sapiens misc_RNA (LOC100132673), miscRNA [XR_039018]	**1.236**
FBLN2	Homo sapiens fibulin 2 (FBLN2), mRNA [NM_001004019]	**1.228**
GNB2L1	Homo sapiens guanine nucleotide binding protein (G protein), beta polypeptide 2-like 1 (GNB2L1), mRNA [NM_006098]	**1.227**
HOXA3	Homo sapiens homeobox A3 (HOXA3), mRNA [NM_153631]	**1.219**
DNAL4	Homo sapiens dynein, axonemal, light chain 4 (DNAL4), mRNA [NM_005740]	**1.216**
STRA6	Homo sapiens stimulated by retinoic acid gene 6 homolog (mouse) (STRA6), mRNA [NM_022369]	**1.210**
CYP11B1	Homo sapiens cytochrome P450, family 11, subfamily B, polypeptide 1 (CYP11B1), mRNA [NM_000497]	**1.205**
CASP1	Homo sapiens caspase 1, apoptosis-related cysteine peptidase (interleukin 1, beta, convertase) (CASP1), mRNA [NM_033292]	**1.199**
FIBIN	Homo sapiens fin bud initiation factor homolog (zebrafish) (FIBIN), mRNA [NM_203371]	**1.195**
IFIH1	Homo sapiens interferon induced with helicase C domain 1 (IFIH1), mRNA [NM_022168]	**1.194**
SUSD4	Homo sapiens sushi domain containing 4 (SUSD4), mRNA [NM_017982]	**1.183**
ST6GALNAC1	Homo sapiens ST6 (alpha-N-acetyl-neuraminyl-2,3-beta-galactosyl-1,3)-N-acetylgalacto saminide alpha-2,6-sialyltransferase 1 (ST6GALNAC1), mRNA [NM_018414]	**1.179**
NEFM	Homo sapiens neurofilament 3 (150kDa medium), mRNA (cDNA clone IMAGE:3163440), partial cds. [BC002421]	**1.169**
RHOB	Homo sapiens ras homolog gene family, member B (RHOB), mRNA [NM_004040]	**1.162**
ANPEP	Homo sapiens alanyl (membrane) aminopeptidase (ANPEP), mRNA [NM_001150]	**1.161**
ABCG2	Homo sapiens ATP-binding cassette, sub-family G (WHITE), member 2 (ABCG2), mRNA [NM_004827]	**1.159**
ADC	Homo sapiens arginine decarboxylase (ADC), mRNA [NM_052998]	**1.146**
DNAH11	Homo sapiens dynein, axonemal, heavy chain 11 (DNAH11), mRNA [NM_003777]	**1.144**
C16orf3	Homo sapiens chromosome 16 open reading frame 3 (C16orf3), mRNA [NM_001214]	**1.143**
MAFA	Homo sapiens v-maf musculoaponeurotic fibrosarcoma oncogene homolog A (avian) (MAFA), mRNA [NM_201589]	**1.142**
POLL	Homo sapiens polymerase (DNA directed), lambda (POLL), mRNA [NM_013274]	**1.142**
YPEL1	Homo sapiens yippee-like 1 (Drosophila) (YPEL1), mRNA [NM_013313]	**1.140**
RPL41	Homo sapiens ribosomal protein L41 (RPL41), mRNA [NM_001035267]	**1.119**
NYX	Homo sapiens nyctalopin (NYX), mRNA [NM_022567]	**1.118**
RPLP0	Homo sapiens ribosomal protein, large, P0 (RPLP0), mRNA [NM_053275]	**1.118**
ISLR	Homo sapiens immunoglobulin superfamily containing leucine-rich repeat (ISLR), mRNA [NM_005545]	**1.115**
RPL18A	Homo sapiens ribosomal protein L18a (RPL18A), mRNA [NM_000980]	**1.109**
DUSP27	Homo sapiens dual specificity phosphatase 27 (putative) (DUSP27), mRNA [NM_001080426]	**1.103**
RCOR2	Homo sapiens REST corepressor 2 (RCOR2), mRNA [NM_173587]	**1.102**
FGL1	Homo sapiens fibrinogen-like 1 (FGL1), mRNA [NM_201553]	**1.099**
HLA-DMA	Homo sapiens major histocompatibility complex, class II, DM alpha (HLA-DMA), mRNA [NM_006120]	**1.097**
SOS1	Homo sapiens son of sevenless homolog 1 (Drosophila) (SOS1), mRNA [NM_005633]	**1.092**

LOC731275	Homo sapiens hypothetical LOC731275 (LOC731275), mRNA [XM_001726998]	**1.091**
TNKS1BP1	Homo sapiens tankyrase 1 binding protein 1, 182kDa (TNKS1BP1), mRNA [NM_033396]	**1.089**
RIMS1	Homo sapiens regulating synaptic membrane exocytosis 1 (RIMS1), mRNA [NM_014989]	**1.088**
NKAIN4	Homo sapiens Na+/K+ transporting ATPase interacting 4 (NKAIN4), mRNA [NM_152864]	**1.086**
RRAD	Homo sapiens Ras-related associated with diabetes (RRAD), mRNA [NM_004165]	**1.086**
FHL3	Homo sapiens four and a half LIM domains 3 (FHL3), mRNA [NM_004468]	**1.082**
C11orf42	Homo sapiens chromosome 11 open reading frame 42 (C11orf42), mRNA [NM_173525]	**1.080**
ARSD	Homo sapiens arylsulfatase D (ARSD), mRNA [NM_009589]	**1.075**
NPB	Homo sapiens cDNA clone IMAGE:5019903, partial cds. [BC073815]	**1.073**
TMCC3	Homo sapiens transmembrane and coiled-coil domain family 3 (TMCC3), mRNA [NM_020698]	**1.068**
AGTRAP	Homo sapiens angiotensin II receptor-associated protein (AGTRAP), mRNA [NM_001040196]	**1.065**
SLC12A7	Homo sapiens solute carrier family 12 (potassium/chloride transporters), member 7 (SLC12A7), mRNA [NM_006598]	**1.058**
CAPNS1	Homo sapiens calpain, small subunit 1 (CAPNS1), mRNA [NM_001749]	**1.055**
GGT8P	Homo sapiens gamma-glutamyltransferase 8 pseudogene (GGT8P), non-coding RNA [NR_003503]	**1.050**
SRP14	Homo sapiens signal recognition particle 14kDa (homologous Alu RNA binding protein) (SRP14), mRNA [NM_003134]	**1.050**
AMDHD1	Homo sapiens amidohydrolase domain containing 1 (AMDHD1), mRNA [NM_152435]	**1.047**
LOC645427	Homo sapiens cDNA FLJ37088 fis, clone BRACE2017124. [AK094407]	**1.047**
C9orf131	Homo sapiens chromosome 9 open reading frame 131 (C9orf131), mRNA [NM_203299]	**1.046**
GAPDH	Homo sapiens glyceraldehyde-3-phosphate dehydrogenase (GAPDH), mRNA [NM_002046]	**1.046**
LOC441377	PREDICTED: Homo sapiens similar to ribosomal protein S26 (LOC441377), mRNA [XM_496991]	**1.041**
CRCT1	Homo sapiens cysteine-rich C-terminal 1 (CRCT1), mRNA [NM_019060]	**1.040**
KIAA1486	Homo sapiens KIAA1486 protein (KIAA1486), mRNA [NM_020864]	**1.039**
ASF1B	Homo sapiens ASF1 anti-silencing function 1 homolog B (S. cerevisiae) (ASF1B), mRNA [NM_018154]	**1.029**
CTSS	Homo sapiens cathepsin S (CTSS), mRNA [NM_004079]	**1.026**
FCHO1	Homo sapiens FCH domain only 1 (FCHO1), mRNA [NM_015122]	**1.026**
ORM1	Homo sapiens orosomucoid 1 (ORM1), mRNA [NM_000607]	**1.026**
ACAP3	Homo sapiens ArfGAP with coiled-coil, ankyrin repeat and PH domains 3 (ACAP3), mRNA [NM_030649]	**1.023**
TLE2	Homo sapiens transducin-like enhancer of split 2 (E(sp1) homolog, Drosophila) (TLE2), mRNA [NM_003260]	**1.022**
TAP2	Homo sapiens transporter 2, ATP-binding cassette, sub-family B (MDR/TAP) (TAP2), mRNA [NM_000544]	**1.019**
MGC16075	Homo sapiens hypothetical protein MGC16075, mRNA (cDNA clone MGC:16075 IMAGE:3616854), complete cds. [BC007354]	**1.010**
C1S	Homo sapiens complement component 1, s subcomponent (C1S), mRNA [NM_001734]	**1.008**
AKAP7	Homo sapiens A kinase (PRKA) anchor protein 7 (AKAP7), mRNA [NM_016377]	**1.007**

LCE1D	Homo sapiens late cornified envelope 1D (LCE1D), mRNA [NM_178352]	**1.006**
SCGB3A2	Homo sapiens secretoglobin, family 3A, member 2 (SCGB3A2), mRNA [NM_054023]	**1.006**
CABP7	Homo sapiens calcium binding protein 7 (CABP7), mRNA [NM_182527]	**1.005**
LOC100128364	Homo sapiens cDNA FLJ38910 fis, clone NT2NE2006813, weakly similar to Cell Surface GlycoproteinL1 Precursor. [AK096229]	**1.004**
MGC16142	Homo sapiens hypothetical protein MGC16142, mRNA (cDNA clone IMAGE:3630861), partial cds. [BC007365]	**1.004**
LRSAM1	Homo sapiens leucine rich repeat and sterile alpha motif containing 1 (LRSAM1), mRNA [NM_138361]	**1.000**
LY6G5C	Homo sapiens lymphocyte antigen 6 complex, locus G5C (LY6G5C), mRNA [NM_025262]	**1.000**
MARCO	Homo sapiens macrophage receptor with collagenous structure (MARCO), mRNA [NM_006770]	**1.000**
