# Supplementary material for: Genotoxicity and molecular response of silver nanoparticle (NP)-based hydrogel
Source: J Nanobiotechnology. 2012 May 1;10:16. doi: 10.1186/1477-3155-10-16 (PMC3430588; doi:10.1186/1477-3155-10-16)
Supplement: Additional file 9 — Common up-regulated genes in cells exposed to silver-NP-hydrogel for 24 h and 48 h. Fold-change is logarithmic ratio (log2 ratio) to expression level in control. [file 1477-3155-10-16-S9.pdf]

Additional File 9. Common up-regulated genes in cells exposed to silver-NP-hydrogel for 24 h and 48 h. Fold-change is logarithmic ratio (log_2 ratio) to expression level in control.

**GeneName**	**Description**	**Fold-change (log_2 ratio)(24h)**	**Fold-change (log_2 ratio)(48h)**
MT1F	Homo sapiens metallothionein 1F (MT1F), mRNA [NM_005949]	**4.511**	**5.638**
MT1A	Homo sapiens metallothionein 1A (MT1A), mRNA [NM_005946]	**4.244**	**4.340**
MT2A	Homo sapiens metallothionein 2A (MT2A), mRNA [NM_005953]	**4.300**	**3.922**
MT1B	Homo sapiens metallothionein 1B (MT1B), mRNA [NM_005947]	**3.925**	**3.900**
MT1G	Homo sapiens metallothionein 1G (MT1G), mRNA [NM_005950]	**4.126**	**3.853**
MT1H	Homo sapiens metallothionein 1H (MT1H), mRNA [NM_005951]	**3.776**	**3.749**
MT1X	Homo sapiens metallothionein 1X (MT1X), mRNA [NM_005952]	**3.691**	**3.681**
MT1JP	Homo sapiens MTB (MTB) mRNA, complete cds. [AF348994]	**3.477**	**4.942**
MT1L	Homo sapiens metallothionein 1L (gene/pseudogene) (MT1L), non-coding RNA [NR_001447]	**3.651**	**3.160**
GCNT3	Homo sapiens glucosaminyl (N-acetyl) transferase 3, mucin type (GCNT3), mRNA [NM_004751]	**2.989**	**4.091**
GPNMB	Homo sapiens glycoprotein (transmembrane) nmb, mRNA (cDNA clone IMAGE:3345861), [BC011595]	**2.815**	**3.735**
SPINK1	Homo sapiens serine peptidase inhibitor, Kazal type 1 (SPINK1), mRNA [NM_003122]	**2.814**	**5.205**
CCL26	Homo sapiens chemokine (C-C motif) ligand 26 (CCL26), mRNA [NM_006072]	**2.881**	**2.802**
KRT34	Homo sapiens keratin 34 (KRT34), mRNA [NM_021013]	**2.554**	**3.699**
SQSTM1	Human phosphotyrosine independent ligand p62B B-cell isoform for the Lck SH2 domain mRNA, [U46752]	**2.817**	**2.539**
MT1M	Homo sapiens metallothionein 1M (MT1M), mRNA [NM_176870]	**3.854**	**2.513**
LIPG	Homo sapiens lipase, endothelial (LIPG), mRNA [NM_006033]	**2.477**	**2.748**
HMOX1	Homo sapiens heme oxygenase (decycling) 1 (HMOX1), mRNA [NM_002133]	**2.926**	**2.410**
LY96	Homo sapiens lymphocyte antigen 96 (LY96), mRNA [NM_015364]	**2.294**	**4.702**
PCYT2	Homo sapiens phosphate cytidylyltransferase 2, ethanolamine	**2.325**	**2.278**

	(PCYT2), mRNA [NM_002861]		
HKDC1	Homo sapiens hexokinase domain containing 1 (HKDC1), mRNA [NM_025130]	**2.231**	**2.882**
PPP1R15A	Homo sapiens protein phosphatase 1, regulatory (inhibitor) subunit 15A (PPP1R15A), mRNA [NM_014330]	**2.229**	**2.478**
ACSS2	Homo sapiens acyl-CoA synthetase short-chain family member 2 (ACSS2), mRNA [NM_018677]	**2.225**	**2.411**
MVD	Homo sapiens mevalonate (diphospho) decarboxylase (MVD), mRNA [NM_002461]	**2.344**	**2.221**
ARL4C	Homo sapiens ADP-ribosylation factor-like 4C (ARL4C), mRNA [NM_005737]	**2.197**	**2.386**
TM4SF19	Homo sapiens transmembrane 4 L six family member 19 (TM4SF19), mRNA [NM_138461]	**2.167**	**3.783**
PDE2A	Homo sapiens phosphodiesterase 2A, cGMP-stimulated (PDE2A), mRNA [NM_002599]	**2.117**	**2.777**
TM7SF2	Homo sapiens transmembrane 7 superfamily member 2 (TM7SF2), mRNA [NM_003273]	**2.100**	**2.981**
PLA2G3	Homo sapiens phospholipase A2, group III (PLA2G3), mRNA [NM_015715]	**2.093**	**2.746**
MT1E	Homo sapiens unknown mRNA. [AF495759]	**2.471**	**2.087**
IDI1	Homo sapiens isopentenyl-diphosphate delta isomerase 1 (IDI1), mRNA [NM_004508]	**2.089**	**2.074**
IFIT3	Homo sapiens interferon-induced protein with tetratricopeptide repeats 3 (IFIT3), mRNA [NM_001549]	**2.058**	**3.378**
UAP1L1	Homo sapiens UDP-N-acteylglucosamine pyrophosphorylase 1-like 1 (UAP1L1), mRNA [NM_207309]	**2.020**	**2.148**
HSPA6	Homo sapiens heat shock 70kDa protein 6 (HSP70B') (HSPA6), mRNA [NM_002155]	**3.145**	**2.015**
INSIG1	Homo sapiens insulin induced gene 1 (INSIG1), mRNA [NM_198336]	**1.991**	**2.430**
GDF15	Homo sapiens growth differentiation factor 15 (GDF15), mRNA [NM_004864]	**1.936**	**3.111**
MGC23284	Homo sapiens hypothetical LOC197187 (MGC23284), non-coding RNA [NR_024399]	**1.894**	**2.685**
TRIM16L	Homo sapiens tripartite motif-containing 16-like (TRIM16L), mRNA [NM_001037330]	**2.058**	**1.885**
FOLR3	Homo sapiens folate receptor 3 (gamma) (FOLR3), mRNA [NM_000804]	**1.994**	**1.871**
TMEM156	Homo sapiens transmembrane protein 156 (TMEM156), mRNA [NM_024943]	**1.865**	**2.054**
CTSD	Homo sapiens cathepsin D (CTSD), mRNA [NM_001909]	**1.848**	**2.064**
SPP1	Homo sapiens secreted phosphoprotein 1 (SPP1), mRNA [NM_001040058]	**1.844**	**2.711**

PSAP	Homo sapiens prosaposin (PSAP), mRNA [NM_001042465]	**2.256**	**1.823**
UPP1	Homo sapiens uridine phosphorylase 1 (UPP1),mRNA [NM_181597]	**1.819**	**2.022**
TOM1	Homo sapiens target of myb1 (chicken) (TOM1), mRNA [NM_005488]	**1.995**	**1.817**
DUSP5	Homo sapiens dual specificity phosphatase 5 (DUSP5), mRNA [NM_004419]	**1.806**	**2.599**
TBC1D2	Homo sapiens TBC1 domain family, member 2 (TBC1D2), mRNA [NM_018421]	**1.826**	**1.778**
AMDHD2	Homo sapiens amidohydrolase domain containing 2 (AMDHD2), mRNA [NM_015944]	**2.377**	**1.757**
IGFN1	Homo sapiens immunoglobulin-like and fibronectin type III domain containing 1 (IGFN1), mRNA [NM_178275]	**1.736**	**1.759**
SLC12A8	Homo sapiens solute carrier family 12, member 8 (SLC12A8), mRNA [NM_024628]	**2.177**	**1.719**
GNPDA1	Homo sapiens glucosamine-6-phosphate deaminase 1 (GNPDA1), mRNA [NM_005471]	**1.967**	**1.705**
MVK	Homo sapiens mevalonate kinase (MVK),mRNA [NM_000431]	**1.790**	**1.702**
POR	Homo sapiens P450 (cytochrome) oxidoreductase (POR), mRNA [NM_000941]	**2.073**	**1.691**
GREB1	Homo sapiens GREB1 protein (GREB1),mRNA [NM_148903]	**1.943**	**1.684**
GPR175	Homo sapiens G protein-coupled receptor 175 (GPR175), mRNA [NM_016372]	**1.679**	**1.789**
CLEC2B	Homo sapiens C-type lectin domain family 2, member B (CLEC2B), mRNA [NM_005127]	**1.662**	**3.039**
TMEM144	Homo sapiens transmembrane protein 144 (TMEM144), mRNA [NM_018342]	**1.654**	**1.713**
NEU1	Homo sapiens sialidase 1 (lysosomal sialidase) (NEU1), mRNA [NM_000434]	**1.650**	**1.953**
G6PD	Homo sapiens glucose-6-phosphate dehydrogenase (G6PD), mRNA [NM_000402]	**1.992**	**1.648**
C1S	Homo sapiens complement component 1, s subcomponent (C1S), mRNA [NM_001734]	**1.639**	**2.858**
SPANXD	Homo sapiens SPANX family, member D (SPANXD), mRNA [NM_032417]	**1.634**	**2.988**
CSF2RA	Homo sapiens colony stimulating factor 2 receptor, alpha, low-affinity (granulocyte-macrophage) (CSF2RA),mRNA [NM_172247]	**1.618**	**1.949**
F2RL2	Homo sapiens coagulation factor II (thrombin) receptor-like 2 (F2RL2), mRNA [NM_004101]	**1.613**	**2.308**
PSG9	Homo sapiens pregnancy specific beta-1-glycoprotein 9 (PSG9), mRNA [NM_002784]	**1.611**	**3.295**

IL6R	Homo sapiens interleukin 6 receptor (IL6R),mRNA [NM_000565]	**1.599**	**2.146**
SH2D5	Homo sapiens SH2 domain containing 5 (SH2D5), mRNA [NM_001103161]	**1.599**	**3.394**
FDFT1	Homo sapiens farnesyl-diphosphate farnesyltransferase 1 (FDFT1), mRNA [NM_004462]	**1.574**	**1.756**
ALDOC	Homo sapiens aldolase C, fructose-bisphosphate (ALDOC), mRNA [NM_005165]	**1.564**	**2.475**
INPPL1	Homo sapiens inositol polyphosphate phosphatase-like 1 (INPPL1), mRNA [NM_001567]	**2.439**	**1.560**
IFI35	Homo sapiens interferon-induced protein 35 (IFI35), mRNA [NM_005533]	**1.711**	**1.547**
CPA4	Homo sapiens carboxypeptidase A4 (CPA4), mRNA [NM_016352]	**2.145**	**1.542**
LSS	Homo sapiens lanosterol synthase (2,3-oxidosqualene-lanosterol cyclase) (LSS), mRNA [NM_001001438]	**1.540**	**1.953**
DDIT3	Homo sapiens DNA-damage-inducible transcript 3 (DDIT3), mRNA [NM_004083]	**1.527**	**2.174**
CYP51A1	Homo sapiens cytochrome P450, family 51, subfamily A, polypeptide 1 (CYP51A1), mRNA [NM_000786]	**2.056**	**1.520**
GEM	Homo sapiens GTP binding protein overexpressed in skeletal muscle (GEM), mRNA [NM_005261]	**1.516**	**1.571**
NUPR1	Homo sapiens nuclear protein 1 (NUPR1),mRNA [NM_001042483]	**1.511**	**1.552**
RHEBL1	Homo sapiens Ras homolog enriched in brain like 1 (RHEBL1), mRNA [NM_144593]	**1.940**	**1.510**
ANKRD1	Homo sapiens ankyrin repeat domain 1 (cardiac muscle) (ANKRD1), mRNA [NM_014391]	**1.962**	**1.503**
CDKN1A	Homo sapiens cyclin-dependent kinase inhibitor 1A (p21, Cip1) (CDKN1A), mRNA [NM_000389]	**1.646**	**1.502**
OSGIN1	Homo sapiens oxidative stress induced growth inhibitor 1 (OSGIN1), mRNA [NM_013370]	**1.501**	**2.488**
BLVRB	Homo sapiens biliverdin reductase B (flavin reductase (NADPH)) (BLVRB), mRNA [NM_000713]	**1.524**	**1.500**
CLCN7	Homo sapiens chloride channel 7 (CLCN7), mRNA [NM_001287]	**2.021**	**1.492**
TNFSF9	Homo sapiens tumor necrosis factor (ligand) superfamily, member 9 (TNFSF9), mRNA [NM_003811]	**2.177**	**1.488**
HS1BP3	Homo sapiens HCLS1 binding protein 3, mRNA (cDNA clone IMAGE:5207261), [BC027947]	**1.485**	**2.233**
FABP3	Homo sapiens fatty acid binding protein 3, (FABP3), mRNA [NM_004102]	**1.483**	**1.879**
AGTRAP	Homo sapiens angiotensin II receptor-associated protein	**1.464**	**1.538**

	(AGTRAP), mRNA [NM_001040196]		
SPANXA1	Homo sapiens sperm protein associated with the nucleus, X-linked, family member A1 (SPANXA1), mRNA [NM_013453]	**1.460**	**2.579**
FTL	Homo sapiens ferritin, light polypeptide (FTL), mRNA [NM_000146]	**1.457**	**1.904**
GSR	Homo sapiens glutathione reductase (GSR), mRNA [NM_000637]	**1.904**	**1.444**
SLC25A1	Homo sapiens solute carrier family 25 (mitochondrial carrier; citrate transporter), member 1 (SLC25A1), mRNA [NM_005984]	**1.434**	**1.475**
FDPSL2A	Homo sapiens MGC44478 (FDPSL2A), non-coding RNA [NR_003262]	**1.707**	**1.425**
ORAI3	Homo sapiens ORAI calcium release-activated calcium modulator 3 (ORAI3), mRNA [NM_152288]	**1.424**	**1.797**
TMEM97	Homo sapiens transmembrane protein 97 (TMEM97), mRNA [NM_014573]	**1.948**	**1.422**
SPANXB2	Homo sapiens SPANX family, member B2 (SPANXB2), mRNA [NM_145664]	**1.410**	**2.828**
NOXO1	Homo sapiens NADPH oxidase organizer 1 (NOXO1), mRNA [NM_144603]	**1.405**	**1.676**
HSD17B7	Homo sapiens hydroxysteroid (17-beta) dehydrogenase 7 (HSD17B7), mRNA [NM_016371]	**1.555**	**1.393**
WDR66	Homo sapiens WD repeat domain 66 (WDR66), mRNA [NM_144668]	**1.384**	**2.851**
PGD	Homo sapiens phosphogluconate dehydrogenase (PGD), mRNA [NM_002631]	**2.081**	**1.381**
LOC100129113	Homo sapiens cDNA FLJ37158 fis, clone BRACE2026293. [AK094477]	**2.316**	**1.375**
PHLDA3	Homo sapiens pleckstrin homology-like domain, family A, member 3 (PHLDA3), mRNA [NM_012396]	**1.592**	**1.373**
AGPAT2	Homo sapiens 1-acylglycerol-3-phosphate O-acyltransferase 2 (lysophosphatidic acid acyltransferase, beta) (AGPAT2), mRNA [NM_006412]	**2.000**	**1.368**
CLDN4	Homo sapiens claudin 4 (CLDN4), mRNA [NM_001305]	**1.885**	**1.362**
SV2A	Homo sapiens synaptic vesicle glycoprotein 2A (SV2A), mRNA [NM_014849]	**1.360**	**1.464**
PPM2C	Homo sapiens protein phosphatase 2C, magnesium-dependent, catalytic subunit (PPM2C), mRNA [NM_018444]	**1.899**	**1.357**
SLC16A6	Homo sapiens solute carrier family 16, member 6 (monocarboxylic acid transporter 7) (SLC16A6), mRNA [NM_004694]	**1.351**	**1.782**
LOC284344	Homo sapiens cDNA FLJ40353 fis, clone TESTI2033520,	**1.345**	**3.220**

	weakly similar to Biliary Glycoprotein 1 Precursor. [AK097672]		
TRIML2	Homo sapiens tripartite motif family-like 2 (TRIML2), mRNA [NM_173553]	**1.344**	**2.343**
PSG1	Homo sapiens pregnancy specific beta-1-glycoprotein 1 (PSG1), mRNA [NM_006905]	**1.340**	**2.820**
C6orf223	Homo sapiens chromosome 6 open reading frame 223 (C6orf223), mRNA [NM_153246]	**1.333**	**1.701**
LOC344887	Homo sapiens mRNA; cDNA DKFZp686B14224 (from clone DKFZp686B14224). [BX640843]	**1.331**	**2.171**
PPAP2C	Homo sapiens phosphatidic acid phosphatase type 2C (PPAP2C), mRNA [NM_177543]	**1.566**	**1.327**
NPC1	Homo sapiens Niemann-Pick disease, type C1 (NPC1), mRNA [NM_000271]	**1.321**	**1.379**
SLC2A6	Homo sapiens solute carrier family 2 (facilitated glucose transporter), member 6 (SLC2A6), mRNA [NM_017585]	**1.847**	**1.321**
CPN2	Homo sapiens carboxypeptidase N, polypeptide 2 (CPN2), mRNA [NM_001080513]	**1.320**	**1.646**
KIFC3	Homo sapiens kinesin family member C3 (KIFC3), mRNA [NM_005550]	**2.513**	**1.318**
VCX2	Homo sapiens variable charge, X-linked 2 (VCX2), mRNA [NM_016378]	**1.318**	**1.399**
ALDOA	Homo sapiens aldolase A, fructose-bisphosphate (ALDOA), mRNA [NM_184041]	**1.382**	**1.317**
C6orf1	Homo sapiens chromosome 6 open reading frame 1 (C6orf1), mRNA [NM_178508]	**1.310**	**1.843**
KIAA1486	Homo sapiens KIAA1486 protein (KIAA1486), mRNA [NM_020864]	**1.297**	**1.839**
MAFF	Homo sapiens v-maf musculoaponeurotic fibrosarcoma oncogene homolog F (avian) (MAFF), mRNA [NM_012323]	**1.377**	**1.296**
SNX8	Homo sapiens sorting nexin 8 (SNX8), mRNA [NM_013321]	**1.305**	**1.296**
HSD17B7P2	Homo sapiens 17-beta-hydroxysteroid dehydrogenase type VII isoform mRNA, [AF165514]	**1.403**	**1.293**
CASP1	Homo sapiens caspase 1, apoptosis-related cysteine peptidase (interleukin 1, beta, convertase) (CASP1),mRNA [NM_033292]	**1.291**	**3.273**
GADD45A	Homo sapiens growth arrest and DNA-damage-inducible, alpha (GADD45A), mRNA [NM_001924]	**1.290**	**1.318**
S100P	Homo sapiens S100 calcium binding protein P (S100P), mRNA [NM_005980]	**1.290**	**1.841**
CTSL1	Homo sapiens cathepsin L1 (CTSL1), mRNA [NM_001912]	**1.287**	**2.085**
FTH1	Homo sapiens ferritin, heavy polypeptide 1 (FTH1), mRNA [NM_002032]	**1.666**	**1.286**

NCRNA00087	Homo sapiens non-protein coding RNA 87 (NCRNA00087), non-coding RNA [NR_024493]	**1.284**	**2.014**
MCOLN1	Homo sapiens mucolipin 1 (MCOLN1), mRNA [NM_020533]	**1.698**	**1.282**
STXBP1	Homo sapiens syntaxin binding protein 1 (STXBP1), mRNA [NM_001032221]	**1.280**	**1.530**
RAP1GAP	Homo sapiens RAP1 GTPase activating protein (RAP1GAP), mRNA [NM_002885]	**1.272**	**2.434**
SLC17A5	Homo sapiens solute carrier family 17 (anion/sugar transporter), member 5 (SLC17A5), mRNA [NM_012434]	**1.269**	**1.622**
TXNRD1	Homo sapiens thioredoxin reductase 1 (TXNRD1), mRNA [NM_003330]	**2.455**	**1.269**
ATF3	Homo sapiens activating transcription factor 3 (ATF3), mRNA [NM_001040619]	**1.302**	**1.266**
DOK7	Homo sapiens docking protein 7 (DOK7), mRNA [NM_173660]	**1.265**	**1.550**
NPB	Homo sapiens cDNA clone IMAGE:5019903, [BC073815]	**1.265**	**1.671**
GGT8P	Homo sapiens gamma-glutamyltransferase 8 pseudogene (GGT8P), non-coding RNA [NR_003503]	**1.815**	**1.261**
NAV3	Homo sapiens neuron navigator 3 (NAV3), mRNA [NM_014903]	**1.260**	**1.554**
MYO16	Homo sapiens myosin XVI (MYO16), mRNA [NM_015011]	**1.256**	**2.458**
ALDH1B1	Homo sapiens aldehyde dehydrogenase 1 family, member B1 (ALDH1B1), mRNA [NM_000692]	**1.251**	**1.411**
ACLY	Human ATP:citrate lyase mRNA, complete cds. [U18197]	**1.907**	**1.247**
IDH1	Homo sapiens isocitrate dehydrogenase 1 (NADP+), soluble (IDH1), mRNA [NM_005896]	**1.568**	**1.244**
IL1A	Homo sapiens interleukin 1, alpha (IL1A), mRNA [NM_000575]	**1.244**	**2.761**
RASA3	Homo sapiens mRNA for Ins(1,3,4,5)P4-binding protein. [X89399]	**1.854**	**1.234**
RAB7L1	Homo sapiens RAB7, member RAS oncogene family-like 1 (RAB7L1), mRNA [NM_003929]	**1.315**	**1.233**
KIF26A	Homo sapiens kinesin family member 26A (KIF26A), mRNA [NM_015656]	**1.543**	**1.229**
SH3TC1	Homo sapiens SH3 domain and tetratricopeptide repeats 1 (SH3TC1), mRNA [NM_018986]	**1.228**	**1.599**
GBA	Homo sapiens glucosidase, beta; acid (includes glucosylceramidase) (GBA), mRNA [NM_001005749]	**1.412**	**1.226**
PSG11	Homo sapiens pregnancy specific beta-1-glycoprotein 11 (PSG11), mRNA [NM_002785]	**1.224**	**1.479**
C7orf57	Homo sapiens chromosome 7 open reading frame 57 (C7orf57), mRNA [NM_001100159]	**1.588**	**1.221**

LAMA1	Homo sapiens laminin, alpha 1 (LAMA1), mRNA [NM_005559]	**1.498**	**1.221**
FASN	Homo sapiens fatty acid synthase (FASN), mRNA [NM_004104]	**1.694**	**1.220**
C19orf20	Homo sapiens chromosome 19 open reading frame 20 (C19orf20), mRNA [NM_033513]	**1.218**	**1.262**
RRAGC	Homo sapiens Ras-related GTP binding C (RRAGC), mRNA [NM_022157]	**1.217**	**1.453**
ATP6V1B2	Homo sapiens ATPase, H+ transporting, lysosomal, V1 subunit B2 (ATP6V1B2), mRNA [NM_001693]	**1.216**	**1.345**
RHOB	Homo sapiens ras homolog gene family, member B (RHOB), mRNA [NM_004040]	**1.216**	**1.654**
GAPDH	Homo sapiens glyceraldehyde-3-phosphate dehydrogenase (GAPDH), mRNA [NM_002046]	**1.869**	**1.215**
RRAGD	Homo sapiens Ras-related GTP binding D (RRAGD), mRNA [NM_021244]	**2.488**	**1.215**
COMMD7	Homo sapiens COMM domain containing 7 (COMMD7), mRNA [NM_053041]	**1.214**	**1.273**
PSG6	Homo sapiens pregnancy specific beta-1-glycoprotein 6 (PSG6), transcript variant 1, mRNA [NM_002782]	**1.209**	**3.438**
FOLR1	Homo sapiens folate receptor 1 (adult) (FOLR1), mRNA [NM_016725]	**1.204**	**2.980**
MMAB	Homo sapiens methylmalonic aciduria (cobalamin deficiency) cblB type (MMAB), mRNA [NM_052845]	**1.303**	**1.203**
B4GALNT1	Homo sapiens beta-1,4-N-acetyl-galactosaminyl transferase 1 (B4GALNT1), mRNA [NM_001478]	**1.203**	**1.466**
IL17RC	Homo sapiens interleukin 17 receptor C (IL17RC), mRNA [NM_153461]	**1.688**	**1.199**
FADS2	Homo sapiens fatty acid desaturase 2 (FADS2), mRNA [NM_004265]	**1.195**	**1.599**
SLC30A1	Homo sapiens solute carrier family 30 (zinc transporter), member 1 (SLC30A1), mRNA [NM_021194]	**1.194**	**2.076**
CPEB1	Homo sapiens cytoplasmic polyadenylation element binding protein 1 (CPEB1), mRNA [NM_030594]	**1.193**	**2.092**
EPS8	Homo sapiens epidermal growth factor receptor pathway substrate 8 (EPS8), mRNA [NM_004447]	**1.192**	**1.298**
HMGCS1	Homo sapiens 3-hydroxy-3-methylglutaryl-Coenzyme A synthase 1 (soluble) (HMGCS1), mRNA [NM_002130]	**2.061**	**1.192**
FADS3	Homo sapiens fatty acid desaturase 3 (FADS3), mRNA [NM_021727]	**1.440**	**1.186**
HYAL1	Homo sapiens hyaluronoglucosaminidase 1 (HYAL1), mRNA [NM_007312]	**1.288**	**1.184**
FSTL3	Homo sapiens follistatin-like 3 (secreted glycoprotein) (FSTL3), mRNA [NM_005860]	**1.218**	**1.183**

STARD4	Homo sapiens StAR-related lipid transfer (START) domain containing 4 (STARD4), mRNA [NM_139164]	**1.182**	**1.595**
C1orf201	Homo sapiens chromosome 1 open reading frame 201 (C1orf201), mRNA [NM_178122]	**1.181**	**1.672**
HABP4	Homo sapiens hyaluronan binding protein 4 (HABP4), mRNA [NM_014282]	**1.824**	**1.181**
SRXN1	Homo sapiens sulfiredoxin 1 homolog (S. cerevisiae) (SRXN1), mRNA [NM_080725]	**1.181**	**1.392**
EPGN	Homo sapiens epithelial mitogen homolog (mouse) (EPGN), mRNA [NM_001013442]	**1.365**	**1.179**
LRRC61	Homo sapiens leucine rich repeat containing 61 (LRRC61), mRNA [NM_023942]	**1.176**	**1.253**
FAIM3	Homo sapiens Fas apoptotic inhibitory molecule 3 (FAIM3), mRNA [NM_005449]	**1.174**	**2.278**
MAPKAPK3	Homo sapiens mitogen-activated protein kinase-activated protein kinase 3 (MAPKAPK3), mRNA [NM_004635]	**1.174**	**1.334**
FAM46B	Homo sapiens family with sequence similarity 46, member B (FAM46B), mRNA [NM_052943]	**1.505**	**1.173**
IFIT2	Homo sapiens interferon-induced protein with tetratricopeptide repeats 2 (IFIT2), mRNA [NM_001547]	**1.172**	**3.467**
DTNA	Homo sapiens dystrobrevin, alpha (DTNA),mRNA [NM_001392]	**1.169**	**2.463**
TNIP1	Homo sapiens TNFAIP3 interacting protein 1 (TNIP1), mRNA [NM_006058]	**1.163**	**1.346**
BEX2	Homo sapiens brain expressed X-linked 2 (BEX2), mRNA [NM_032621]	**1.157**	**1.875**
PLEKHM2	Homo sapiens pleckstrin homology domain containing, family M (with RUN domain) member 2 (PLEKHM2), mRNA [NM_015164]	**1.908**	**1.157**
METTL7B	Homo sapiens methyltransferase like 7B (METTL7B), mRNA [NM_152637]	**1.153**	**1.549**
RAB4B	Homo sapiens RAB4B, member RAS oncogene family (RAB4B), mRNA [NM_016154]	**1.651**	**1.152**
CLIC2	Homo sapiens chloride intracellular channel 2 (CLIC2), mRNA [NM_001289]	**1.150**	**1.443**
NAPA	Homo sapiens N-ethylmaleimide-sensitive factor attachment protein, alpha (NAPA), mRNA [NM_003827]	**1.422**	**1.148**
LDLR	Homo sapiens low density lipoprotein receptor (LDLR), mRNA [NM_000527]	**1.944**	**1.144**
TOX3	Homo sapiens TOX high mobility group box family member 3 (TOX3), mRNA [NM_001080430]	**1.142**	**1.890**
YIF1B	Homo sapiens Yip1 interacting factor homolog B (S. cerevisiae) (YIF1B), transcript variant 2, mRNA [NM_033557]	**1.258**	**1.140**

CRAT	Homo sapiens carnitine acetyltransferase (CRAT),mRNA [NM_000755]	**1.138**	**1.146**
SLC7A11	Homo sapiens solute carrier family 7, (cationic amino acid transporter, y+ system) member 11 (SLC7A11), mRNA [NM_014331]	**1.787**	**1.137**
CD180	Homo sapiens CD180 molecule (CD180), mRNA [NM_005582]	**1.136**	**1.451**
C1orf85	Homo sapiens chromosome 1 open reading frame 85 (C1orf85), mRNA [NM_144580]	**1.127**	**1.864**
TAPBPL	Homo sapiens TAP binding protein-like (TAPBPL), mRNA [NM_018009]	**1.122**	**1.303**
CARD16	Homo sapiens caspase recruitment domain family, member 16 (CARD16), mRNA [NM_001017534]	**1.117**	**1.387**
GAS6	Homo sapiens growth arrest-specific 6 (GAS6), mRNA [NM_000820]	**2.560**	**1.117**
TMEM55B	Homo sapiens transmembrane protein 55B (TMEM55B), mRNA [NM_144568]	**1.888**	**1.116**
SEMA3F	Homo sapiens sema domain, immunoglobulin domain , short basic domain, (semaphorin) 3F (SEMA3F), mRNA [NM_004186]	**1.210**	**1.115**
FDPS	Homo sapiens farnesyl diphosphate synthase (farnesyl pyrophosphate synthetase, dimethylallyltranstransferase, geranyltranstransferase) (FDPS), mRNA [NM_002004]	**1.114**	**1.146**
NQO2	Homo sapiens NAD(P)H dehydrogenase, quinone 2 (NQO2), mRNA [NM_000904]	**1.114**	**1.243**
RCOR2	Homo sapiens REST corepressor 2 (RCOR2), mRNA [NM_173587]	**1.114**	**1.670**
DHRS9	Homo sapiens dehydrogenase/reductase (SDR family) member 9 (DHRS9), mRNA [NM_005771]	**1.111**	**1.830**
TMEM161A	Homo sapiens transmembrane protein 161A (TMEM161A), mRNA [NM_017814]	**1.369**	**1.110**
KIAA0415	Homo sapiens KIAA0415 (KIAA0415), mRNA [NM_014855]	**1.713**	**1.103**
RAG1AP1	Homo sapiens recombination activating gene 1 activating protein 1 (RAG1AP1), mRNA [NM_018845]	**1.650**	**1.102**
NDRG1	Homo sapiens N-myc downstream regulated 1 (NDRG1), mRNA [NM_006096]	**1.098**	**1.491**
CEBPB	Homo sapiens CCAAT/enhancer binding protein (C/EBP), beta (CEBPB), mRNA [NM_005194]	**1.343**	**1.098**
ATP6AP1	Homo sapiens ATPase, H+ transporting, lysosomal accessory protein 1 (ATP6AP1), mRNA [NM_001183]	**1.188**	**1.095**
VAT1	Homo sapiens vesicle amine transport protein 1 homolog (T. californica) (VAT1), mRNA [NM_006373]	**1.095**	**1.708**
CYP4F11	Homo sapiens cytochrome P450, family 4, subfamily F,	**1.461**	**1.094**

	polypeptide 11 (CYP4F11), mRNA [NM_021187]		
TSC22D3	Homo sapiens TSC22 domain family, member 3 (TSC22D3), mRNA [NM_004089]	**1.358**	**1.094**
HRK	Homo sapiens harakiri, BCL2 interacting protein (contains only BH3 domain) (HRK), mRNA [NM_003806]	**1.093**	**1.953**
MAP2K2	Homo sapiens mitogen-activated protein kinase kinase 2 (MAP2K2), mRNA [NM_030662]	**1.950**	**1.091**
POTEF	Homo sapiens POTE ankyrin domain family, member F (POTEF), mRNA [NM_001099771]	**2.276**	**1.089**
FLCN	Homo sapiens mRNA; cDNA DKFZp547A118 (from clone DKFZp547A118). [AL831885]	**1.087**	**1.594**
CDIPT	Homo sapiens CDP-diacylglycerol--inositol 3-phosphatidyltransferase (CDIPT), mRNA [NM_006319]	**2.129**	**1.085**
GRAMD1A	Homo sapiens GRAM domain containing 1A (GRAMD1A), mRNA [NM_020895]	**1.362**	**1.085**
PTPRR	Homo sapiens protein tyrosine phosphatase, receptor type, R (PTPRR), mRNA [NM_002849]	**1.085**	**3.828**
SLC39A1	Homo sapiens solute carrier family 39 (zinc transporter), member 1 (SLC39A1), mRNA [NM_014437]	**1.861**	**1.084**
SERPINE1	Homo sapiens serpin peptidase inhibitor, clade E member 1 (SERPINE1), mRNA [NM_000602]	**1.766**	**1.082**
ABHD3	Homo sapiens abhydrolase domain containing 3 (ABHD3), mRNA [NM_138340]	**1.360**	**1.081**
MGAT4B	Homo sapiens mannosyl (alpha-1,3-)-glycoprotein beta-1,4-N-acetylglucosaminyltransferase, isozyme B (MGAT4B), mRNA [NM_054013]	**2.268**	**1.081**
FNIP2	Homo sapiens folliculin interacting protein 2 (FNIP2), mRNA [NM_020840]	**1.080**	**1.556**
PLEKHG1	Homo sapiens pleckstrin homology domain containing, family G (with RhoGef domain) member 1 (PLEKHG1), mRNA [NM_001029884]	**1.072**	**1.301**
LPIN1	Homo sapiens lipin 1 (LPIN1), mRNA [NM_145693]	**1.533**	**1.070**
TNFRSF14	Homo sapiens tumor necrosis factor receptor superfamily, member 14 (TNFRSF14), mRNA [NM_003820]	**1.069**	**2.261**
HIF1AN	Homo sapiens hypoxia inducible factor 1, alpha subunit inhibitor (HIF1AN), mRNA [NM_017902]	**1.107**	**1.068**
GGTLC2	Homo sapiens gamma-glutamyltransferase light chain 2 (GGTLC2), mRNA [NM_199127]	**1.067**	**1.121**
LPAR5	Homo sapiens lysophosphatidic acid receptor 5 (LPAR5), mRNA [NM_020400]	**1.065**	**1.197**
UST	Homo sapiens uronyl-2-sulfotransferase (UST), mRNA [NM_005715]	**1.065**	**1.246**
DUSP10	Homo sapiens dual specificity phosphatase 10 (DUSP10), mRNA [NM_007207]	**1.230**	**1.064**

NOV	Homo sapiens nephroblastoma overexpressed gene (NOV), mRNA [NM_002514]	**1.057**	**2.021**
ZBTB41	Homo sapiens zinc finger and BTB domain containing 41 (ZBTB41), mRNA [NM_194314]	**1.056**	**1.196**
GPR115	Homo sapiens G protein-coupled receptor 115 (GPR115), mRNA [NM_153838]	**1.055**	**1.267**
SULT1A1	Homo sapiens sulfotransferase family, cytosolic, 1A, phenol-preferring, member 1 (SULT1A1), mRNA [NM_177529]	**1.138**	**1.055**
SOCS3	Homo sapiens suppressor of cytokine signaling 3 (SOCS3), mRNA [NM_003955]	**1.140**	**1.053**
MED16	Homo sapiens mediator complex subunit 16 (MED16), mRNA [NM_005481]	**1.253**	**1.049**
CTSA	Homo sapiens cathepsin A (CTSA), mRNA [NM_000308]	**1.284**	**1.046**
TAPBP	Homo sapiens TAP binding protein (tapasin) (TAPBP), mRNA [NM_172208]	**1.042**	**1.109**
GALE	Homo sapiens UDP-galactose-4-epimerase (GALE), mRNA [NM_000403]	**1.655**	**1.041**
CDC25B	Homo sapiens cell division cycle 25 homolog B (S. pombe) (CDC25B), mRNA [NM_021873]	**1.222**	**1.040**
SDSL	Homo sapiens serine dehydratase-like (SDSL), mRNA [NM_138432]	**1.040**	**1.418**
ENO1	Homo sapiens enolase 1, (alpha) (ENO1), mRNA [NM_001428]	**1.349**	**1.039**
TMBIM1	Homo sapiens transmembrane BAX inhibitor motif containing 1 (TMBIM1), mRNA [NM_022152]	**1.328**	**1.038**
TNFAIP8L3	Homo sapiens tumor necrosis factor, alpha-induced protein 8-like 3 (TNFAIP8L3), mRNA [NM_207381]	**1.195**	**1.038**
C6orf145	Homo sapiens chromosome 6 open reading frame 145 (C6orf145), mRNA [NM_183373]	**1.121**	**1.037**
MMP15	Homo sapiens matrix metallopeptidase 15 (membrane-inserted) (MMP15), mRNA [NM_002428]	**1.037**	**1.209**
SIRT6	Homo sapiens sirtuin (silent mating type information regulation 2 homolog) 6 (S. cerevisiae) (SIRT6), mRNA [NM_016539]	**1.159**	**1.037**
RPUSD3	Homo sapiens RNA pseudouridylate synthase domain containing 3 (RPUSD3), mRNA [NM_173659]	**1.036**	**1.122**
IL8	Homo sapiens interleukin 8 (IL8), mRNA [NM_000584]	**1.450**	**1.035**
ASAH1	Homo sapiens cDNA: FLJ21558 fis, clone COL06372. [AK025211]	**1.034**	**1.439**
CD276	Homo sapiens CD276 molecule (CD276),mRNA [NM_001024736]	**1.058**	**1.033**
HMGCR	Homo sapiens 3-hydroxy-3-methylglutaryl-Coenzyme A reductase (HMGCR), mRNA [NM_000859]	**1.030**	**1.674**

ATG2A	Homo sapiens ATG2 autophagy related 2 homolog A (S. cerevisiae) (ATG2A), mRNA [NM_015104]	**1.751**	**1.027**
CTNS	Homo sapiens cystinosis, nephropathic (CTNS), transcript variant 2, mRNA [NM_004937]	**1.024**	**1.277**
C10orf58	Homo sapiens chromosome 10 open reading frame 58 (C10orf58), mRNA [NM_032333]	**1.023**	**1.716**
GIPC1	Homo sapiens GIPC PDZ domain containing family, member 1 (GIPC1), mRNA [NM_005716]	**1.938**	**1.023**
GRINA	Homo sapiens glutamate receptor, ionotropic, N-methyl D-aspartate-associated protein 1 (glutamate binding) (GRINA), mRNA [NM_000837]	**1.022**	**1.476**
LOC407835	Homo sapiens mitogen-activated protein kinase kinase 2 pseudogene (LOC407835), non-coding RNA [NR_002144]	**2.185**	**1.020**
ZFPL1	Homo sapiens zinc finger protein-like 1 (ZFPL1), mRNA [NM_006782]	**1.353**	**1.018**
SPNS1	Homo sapiens spinster homolog 1 (Drosophila) (SPNS1), mRNA [NM_032038]	**1.765**	**1.017**
PLCL1	Homo sapiens phospholipase C-like 1 (PLCL1), mRNA [NM_006226]	**1.010**	**1.114**
COX1	Cytochrome c oxidase subunit 1 (EC 1.9.3.1)(Cytochrome c oxidase polypeptide I) [Source:UniProtKB/ Swiss-Prot; Acc:P00395] [ENST00000361624]	**1.008**	**1.087**
GPC1	Homo sapiens glypican 1 (GPC1), mRNA [NM_002081]	**1.705**	**1.008**
CORO1B	Homo sapiens coronin, actin binding protein, 1B (CORO1B), mRNA [NM_020441]	**1.004**	**1.216**
PDK4	Homo sapiens pyruvate dehydrogenase kinase, isozyme 4 (PDK4), mRNA [NM_002612]	**1.004**	**1.685**
CTRB2	Homo sapiens chymotrypsinogen B2 (CTRB2), mRNA [NM_001025200]	**1.021**	**1.002**
UBTD1	Homo sapiens ubiquitin domain containing 1 (UBTD1), mRNA [NM_024954]	**1.415**	**1.001**
CBX4	Homo sapiens chromobox homolog 4 (Pc class homolog, Drosophila) (CBX4), mRNA [NM_003655]	**1.179**	**1.000**
