# Supplementary material for: Genotoxicity and molecular response of silver nanoparticle (NP)-based hydrogel
Source: J Nanobiotechnology. 2012 May 1;10:16. doi: 10.1186/1477-3155-10-16 (PMC3430588; doi:10.1186/1477-3155-10-16)
Supplement: Additional file 10 — Common down-regulated genes in cells exposed to silver-NP-hydrogel for 24h and 48 h. Fold-change is logarithmic ratio (log2 ratio) to expression level in control. [file 1477-3155-10-16-S10.pdf]

Additional File 10. Common down-regulated genes in cells exposed to silver-NP-hydrogel for 24h and 48 h. Fold-change is logarithmic ratio (log_2 ratio) to expression level in control.

GeneName	Description	Fold-change (log2 ratio)(24h)	Fold-change (log2ratio)(48h)
DLC1	Homo sapiens deleted in liver cancer 1 (DLC1), mRNA [NM_182643]	**-2.870**	**-2.473**
LFNG	Homo sapiens LFNG O-fucosylpeptide 3-beta-N-acetylglucosaminyltransferase (LFNG), mRNA [NM_001040167]	**-2.339**	**-2.134**
IL17RB	Homo sapiens interleukin 17 receptor B (IL17RB), mRNA [NM_018725]	**-2.308**	**-2.044**
GPX1	Homo sapiens glutathione peroxidase 1 (GPX1), mRNA [NM_201397]	**-1.940**	**-1.957**
PLCXD3	Homo sapiens phosphatidylinositol-specific phospholipase C, X domain containing 3 (PLCXD3), mRNA [NM_001005473]	**-1.918**	**-2.403**
ADRA2A	Homo sapiens adrenergic, alpha-2A-, receptor (ADRA2A), mRNA [NM_000681]	**-2.113**	**-1.904**
TNFRSF25	Homo sapiens tumor necrosis factor receptor superfamily, member 25 (TNFRSF25), mRNA [NM_148965]	**-1.829**	**-1.987**
SPATA9	Homo sapiens cDNA FLJ35906 fis, clone TESTI2009727. [AK093225]	**-1.776**	**-1.946**
NOX4	Homo sapiens NADPH oxidase 4 (NOX4), mRNA [NM_016931]	**-2.216**	**-1.755**
DLX2	Homo sapiens distal-less homeobox 2 (DLX2), mRNA [NM_004405]	**-1.721**	**-2.128**
SNHG10	Homo sapiens small nucleolar RNA host gene 10 (non-protein coding) (SNHG10), non-coding RNA [NR_003138]	**-1.677**	**-2.772**
ITGA11	Homo sapiens integrin, alpha 11 (ITGA11), mRNA [NM_001004439]	**-2.988**	**-1.647**
AFAP1L2	Homo sapiens actin filament associated protein 1-like 2 (AFAP1L2), mRNA [NM_032550]	**-2.262**	**-1.628**
TFPI2	Homo sapiens tissue factor pathway inhibitor 2 (TFPI2), mRNA [NM_006528]	**-1.619**	**-1.658**
EDN2	Homo sapiens endothelin 2 (EDN2), mRNA [NM_001956]	**-3.356**	**-1.610**
GOLGA8E	Homo sapiens golgi autoantigen, golgin subfamily a, 8E (GOLGA8E), mRNA [NM_001012423]	**-1.600**	**-2.086**
LOC100132439	Homo sapiens similar to Protein FAM27E3 (LOC100132439), mRNA [XM_001719283]	**-1.575**	**-1.603**
COL9A3	Homo sapiens collagen, type IX, alpha 3 (COL9A3), mRNA [NM_001853]	**-1.993**	**-1.571**
ODAM	Homo sapiens odontogenic, ameloblast asssociated (ODAM), mRNA [NM_017855]	**-1.619**	**-1.564**
MGC3207	Homo sapiens translation initiation factor eIF-2B subunit alpha/beta/delta-like protein (MGC3207), mRNA [NM_001031727]	**-1.560**	**-1.751**
ZNF594	Homo sapiens zinc finger protein 594 (ZNF594), mRNA [NM_032530]	**-1.875**	**-1.547**
E2F8	Homo sapiens E2F transcription factor 8 (E2F8), mRNA [NM_024680]	**-2.073**	**-1.541**
LOC392335	Homo sapiens misc_RNA (LOC392335), miscRNA [XR_037043]	**-1.534**	**-2.181**
C10orf140	Homo sapiens chromosome 10 open reading frame 140 (C10orf140), mRNA [NM_207371]	**-1.532**	**-2.500**
ID2	Homo sapiens inhibitor of DNA binding 2, dominant negative helix-loop-helix protein (ID2), mRNA [NM_002166]	**-1.529**	**-3.424**
C18orf56	Homo sapiens chromosome 18 open reading frame 56 (C18orf56), mRNA	**-2.574**	**-1.507**

	[NM_001012716]		
KIAA0802	Homo sapiens KIAA0802 (KIAA0802), mRNA [NM_015210]	**-1.486**	**-2.011**
CDC14A	Homo sapiens CDC14 cell division cycle 14 homolog A (CDC14A), mRNA [NM_003672]	**-1.478**	**-3.711**
LOC653071	Homo sapiens similar to CG32820-PA, isoform A, mRNA (cDNA clone IMAGE:4812880),[BC068588]	**-1.476**	**-3.032**
GPM6B	Homo sapiens glycoprotein M6B (GPM6B), mRNA [NM_001001996]	**-1.466**	**-1.576**
COL15A1	Homo sapiens collagen, type XV, alpha 1 (COL15A1), mRNA [NM_001855]	**-1.503**	**-1.463**
NPTX1	Homo sapiens neuronal pentraxin I (NPTX1), mRNA [NM_002522]	**-1.528**	**-1.457**
C1orf133	Homo sapiens chromosome 1 open reading frame 133 (C1orf133), non-coding RNA [NR_024337]	**-1.630**	**-1.454**
MYLK2	Homo sapiens myosin light chain kinase 2 (MYLK2), mRNA [NM_033118]	**-1.519**	**-1.444**
MGC16121	Homo sapiens hypothetical protein MGC16121, mRNA (cDNA clone IMAGE:3627113), complete cds. [BC007360]	**-2.590**	**-1.418**
LOC203510	Homo sapiens similar to hCG1644442 (LOC203510), mRNA [XM_001719132]	**-1.412**	**-2.524**
C15orf23	Homo sapiens chromosome 15 open reading frame 23 (C15orf23),mRNA [NM_001142761]	**-1.411**	**-1.421**
LOC344178	Homo sapiens similar to hCG1794703 (LOC344178), mRNA [XM_001721796]	**-1.398**	**-3.203**
PL-5283	Homo sapiens PL-5283 protein (PL-5283), mRNA [NM_001130929]	**-1.398**	**-1.705**
LOC100128519	Homo sapiens misc_RNA (LOC100128519), miscRNA [XR_038473]	**-1.396**	**-2.967**
RASGRP1	Homo sapiens RAS guanyl releasing protein 1 (calcium and DAG-regulated) (RASGRP1), mRNA [NM_005739]	**-2.079**	**-1.390**
LGR6	Homo sapiens leucine-rich repeat-containing G protein-coupled receptor 6 (LGR6), mRNA [NM_001017403]	**-1.386**	**-1.405**
USP11	Homo sapiens ubiquitin specific peptidase 11 (USP11), mRNA [NM_004651]	**-1.648**	**-1.382**
LOC340508	Homo sapiens hypothetical protein LOC340508 (LOC340508), non-coding RNA [NR_002942]	**-1.572**	**-1.367**
SLCO1C1	Homo sapiens solute carrier organic anion transporter family, member 1C1 (SLCO1C1), mRNA [NM_017435]	**-1.362**	**-1.644**
FGFBP3	Homo sapiens fibroblast growth factor binding protein 3 (FGFBP3), mRNA [NM_152429]	**-1.463**	**-1.349**
KANK4	Homo sapiens KN motif and ankyrin repeat domains 4 (KANK4), mRNA [NM_181712]	**-1.336**	**-1.670**
KLRC1	Homo sapiens killer cell lectin-like receptor subfamily C, member 1 (KLRC1), mRNA [NM_007328]	**-2.133**	**-1.335**
LOC791120	Homo sapiens hypothetical LOC791120 (LOC791120), non-coding RNA [NR_015357]	**-1.709**	**-1.330**
C13orf33	Homo sapiens chromosome 13 open reading frame 33 (C13orf33), mRNA [NM_032849]	**-1.400**	**-1.321**
LOC729983	Homo sapiens hypothetical LOC729983 (LOC729983), mRNA [XM_001718065]	**-1.313**	**-1.584**
FAM179A	Homo sapiens family with sequence similarity 179, member A (FAM179A), mRNA [NM_199280]	**-1.311**	**-2.160**

LOC730834	DB090170 TESTI4 Homo sapiens cDNA clone TESTI4038997 5', mRNA sequence [DB090170]	**-1.305**	**-1.923**
HOXC8	Homo sapiens homeobox C8 (HOXC8), mRNA [NM_022658]	**-2.360**	**-1.303**
IGFBP5	Homo sapiens insulin-like growth factor binding protein 5 (IGFBP5), mRNA [NM_000599]	**-1.301**	**-2.339**
FAM83D	Homo sapiens family with sequence similarity 83, member D (FAM83D), mRNA [NM_030919]	**-1.366**	**-1.300**
KAL1	Homo sapiens Kallmann syndrome 1 sequence (KAL1), mRNA [NM_000216]	**-1.562**	**-1.272**
ELP2P	Homo sapiens endozepine-like peptide 2 pseudogene (ELP2P), non-coding RNA [NR_024120]	**-1.255**	**-1.322**
CCDC74B	Homo sapiens coiled-coil domain containing 74B (CCDC74B), mRNA [NM_207310]	**-1.250**	**-1.815**
EID2	Homo sapiens EP300 interacting inhibitor of differentiation 2 (EID2), mRNA [NM_153232]	**-1.248**	**-1.251**
CCIN	Homo sapiens calicin (CCIN), mRNA [NM_005893]	**-1.396**	**-1.247**
CIT	Homo sapiens citron (rho-interacting, serine/threonine kinase 21) (CIT), mRNA [NM_007174]	**-1.291**	**-1.242**
HMGB2	Homo sapiens high-mobility group box 2 (HMGB2), mRNA [NM_002129]	**-1.239**	**-1.322**
FAM100B	Homo sapiens family with sequence similarity 100, member B (FAM100B), mRNA [NM_182565]	**-1.236**	**-1.337**
FGF18	Homo sapiens fibroblast growth factor 18 (FGF18), mRNA [NM_003862]	**-1.233**	**-1.396**
IGFL2	Homo sapiens IGF-like family member 2 (IGFL2), mRNA [NM_001002915]	**-1.393**	**-1.227**
HSD11B2	Homo sapiens hydroxysteroid (11-beta) dehydrogenase 2 (HSD11B2), mRNA [NM_000196]	**-1.219**	**-1.944**
NR4A2	Homo sapiens nuclear receptor subfamily 4, group A, member 2 (NR4A2), mRNA [NM_006186]	**-1.216**	**-1.327**
THAP2	Homo sapiens THAP domain containing, apoptosis associated protein 2 (THAP2), mRNA [NM_031435]	**-1.205**	**-1.408**
ABCD4	Homo sapiens ATP-binding cassette, sub-family D (ALD), member 4 (ABCD4), mRNA [NM_005050]	**-1.195**	**-1.526**
LOC338620	Homo sapiens hypothetical protein LOC338620, mRNA (cDNA clone IMAGE:6023208), partial cds. [BC043009]	**-1.227**	**-1.189**
TXNIP	Homo sapiens thioredoxin interacting protein (TXNIP), mRNA [NM_006472]	**-1.185**	**-1.882**
ADAMTS3	Homo sapiens ADAM metallopeptidase with thrombospondin type 1 motif, 3 (ADAMTS3), mRNA [NM_014243]	**-1.184**	**-1.201**
NMU	Homo sapiens neuromedin U (NMU), mRNA [NM_006681]	**-1.419**	**-1.180**
DLX4	Homo sapiens distal-less homeobox 4 (DLX4), mRNA [NM_138281]	**-1.543**	**-1.179**
LOC100130171	Homo sapiens misc_RNA (LOC100130171), miscRNA [XR_038676]	**-1.173**	**-2.243**
ITM2B	Homo sapiens integral membrane protein 2B (ITM2B), mRNA [NM_021999]	**-1.167**	**-1.773**
MGC12935	Homo sapiens hypothetical protein MGC12935, mRNA (cDNA clone IMAGE:4309284), [BC004565]	**-1.464**	**-1.158**
GNGT1	Homo sapiens guanine nucleotide binding protein (G protein), gamma transducing activity polypeptide 1 (GNGT1), mRNA [NM_021955]	**-1.339**	**-1.156**

GCNT1	Homo sapiens glucosaminyl (N-acetyl) transferase 1, core 2 (beta-1,6-N-acetylglucosaminyltransferase) (GCNT1), mRNA [NM_001490]	**-1.404**	**-1.148**
IKZF4	Homo sapiens IKAROS family zinc finger 4 (Eos) (IKZF4), mRNA [NM_022465]	**-1.951**	**-1.148**
IGF1	Homo sapiens insulin-like growth factor 1 (somatomedin C) (IGF1), mRNA [NM_000618]	**-1.146**	**-1.263**
ANO8	Homo sapiens anoctamin 8 (ANO8), mRNA [NM_020959]	**-1.143**	**-1.702**
GPER	Homo sapiens G protein-coupled estrogen receptor 1 (GPER),mRNA [NM_001039966]	**-1.143**	**-1.661**
MICALCL	Homo sapiens MICAL C-terminal like (MICALCL), mRNA [NM_032867]	**-1.143**	**-2.147**
GNRH1	Homo sapiens gonadotropin-releasing hormone 1 (luteinizing-releasing hormone) (GNRH1), mRNA [NM_000825]	**-1.140**	**-1.216**
TMC8	Homo sapiens transmembrane channel-like 8 (TMC8), mRNA [NM_152468]	**-1.134**	**-1.466**
GABRE	Homo sapiens gamma-aminobutyric acid (GABA) A receptor, epsilon (GABRE), mRNA [NM_004961]	**-1.567**	**-1.132**
RBP1	Homo sapiens retinol binding protein 1, cellular (RBP1), mRNA [NM_002899]	**-1.130**	**-1.618**
LOC100129397	Homo sapiens cDNA FLJ38522 fis, clone HCHON2000818. [AK095841]	**-1.129**	**-1.435**
ZNF486	Homo sapiens zinc finger protein 486 (ZNF486), mRNA [NM_052852]	**-1.129**	**-1.874**
SVIP	Homo sapiens small VCP/p97-interacting protein (SVIP), mRNA [NM_148893]	**-1.126**	**-1.495**
FLJ45244	Homo sapiens hypothetical locus FLJ45244 (FLJ45244), non-coding RNA [NR_015415]	**-1.122**	**-1.148**
MEX3A	Homo sapiens mex-3 homolog A (C. elegans) (MEX3A), mRNA [NM_001093725]	**-1.383**	**-1.122**
PLAGL2	Homo sapiens pleiomorphic adenoma gene-like 2 (PLAGL2), mRNA [NM_002657]	**-1.116**	**-1.382**
RIBC2	Homo sapiens RIB43A domain with coiled-coils 2 (RIBC2), mRNA [NM_015653]	**-1.115**	**-1.183**
KIF18B	Homo sapiens hypothetical protein LOC146909, mRNA (cDNA clone IMAGE:4418755), [BC048263]	**-1.114**	**-1.704**
GNG11	Homo sapiens guanine nucleotide binding protein (G protein), gamma 11 (GNG11), mRNA [NM_004126]	**-1.226**	**-1.107**
CCDC34	Homo sapiens coiled-coil domain containing 34 (CCDC34),mRNA [NM_080654]	**-1.105**	**-1.368**
PDGFRB	Homo sapiens platelet-derived growth factor receptor, beta polypeptide (PDGFRB), mRNA [NM_002609]	**-1.104**	**-2.142**
KCTD16	Homo sapiens potassium channel tetramerisation domain containing 16 (KCTD16), mRNA [NM_020768]	**-1.103**	**-1.503**
RPL32P3	Homo sapiens ribosomal protein L32 pseudogene 3 (RPL32P3), non-coding RNA [NR_003111]	**-1.097**	**-1.831**
SEPW1	Homo sapiens selenoprotein W, 1 (SEPW1), mRNA [NM_003009]	**-2.313**	**-1.092**
SULF2	Homo sapiens sulfatase 2 (SULF2), mRNA [NM_018837]	**-1.251**	**-1.091**
LOC339483	Homo sapiens cDNA FLJ38790 fis, clone LIVER2002842. [AK096109]	**-1.860**	**-1.083**
C15orf5	Homo sapiens chromosome 15 open reading frame 5, mRNA (cDNA clone	**-1.127**	**-1.081**

	MGC:97283 IMAGE:7262532), [BC069765]		
FGF12	Homo sapiens fibroblast growth factor 12 (FGF12), mRNA [NM_004113]	**-1.984**	**-1.076**
EFCAB10	Homo sapiens cDNA clone IMAGE:6616931, partial cds. [BC062748]	**-1.074**	**-2.506**
TYMS	Homo sapiens thymidylate synthetase (TYMS), mRNA [NM_001071]	**-1.411**	**-1.074**
FLJ10357	Homo sapiens hypothetical protein FLJ10357 (FLJ10357), mRNA [NM_018071]	**-1.212**	**-1.070**
KIAA1524	Homo sapiens KIAA1524 (KIAA1524), mRNA [NM_020890]	**-1.068**	**-1.643**
MATN3	Homo sapiens matrilin 3 (MATN3), mRNA [NM_002381]	**-1.068**	**-1.069**
TRIM66	Homo sapiens tripartite motif-containing 66 (TRIM66), mRNA [NM_014818]	**-1.067**	**-1.898**
FAM64A	Homo sapiens family with sequence similarity 64, member A (FAM64A), mRNA [NM_019013]	**-1.272**	**-1.062**
MYB	Homo sapiens v-myb myeloblastosis viral oncogene homolog (avian) (MYB), mRNA [NM_005375]	**-1.416**	**-1.062**
C18orf55	Homo sapiens chromosome 18 open reading frame 55 (C18orf55), mRNA [NM_014177]	**-1.056**	**-1.585**
C5orf13	Homo sapiens chromosome 5 open reading frame 13 (C5orf13), mRNA [NM_004772]	**-1.054**	**-1.098**
MKX	Homo sapiens mohawk homeobox (MKX), mRNA [NM_173576]	**-1.054**	**-1.110**
ZBED2	Homo sapiens zinc finger, BED-type containing 2 (ZBED2), mRNA [NM_024508]	**-2.175**	**-1.050**
ZNF284	Zinc finger protein 284 [Source:UniProtKB/ Swiss-Prot;Acc:Q2VY69] [ENST00000328297]	**-1.049**	**-1.219**
LOC100133154	Homo sapiens hypothetical protein LOC100133154 (LOC100133154), mRNA [XM_001714925]	**-2.075**	**-1.048**
RNF214	Homo sapiens ring finger protein 214 (RNF214), mRNA [NM_001077239]	**-1.048**	**-1.125**
TMEM129	Homo sapiens transmembrane protein 129 (TMEM129), mRNA [NM_138385]	**-1.048**	**-1.587**
TUBA8	Homo sapiens tubulin, alpha 8 (TUBA8), mRNA [NM_018943]	**-1.048**	**-1.142**
MGC26597	Homo sapiens PIP5K1A pseudogene, mRNA (cDNA clone IMAGE:4828163). [BC028580]	**-1.150**	**-1.046**
RASSF5	Homo sapiens Ras association (RalGDS/AF-6) domain family member 5 (RASSF5), mRNA [NM_182663]	**-1.045**	**-1.238**
MAGEA1	Homo sapiens melanoma antigen family A, 1 (directs expression of antigen MZ2-E) (MAGEA1), mRNA [NM_004988]	**-1.044**	**-2.361**
NSBP1	Homo sapiens nucleosomal binding protein 1 (NSBP1), mRNA [NM_030763]	**-1.043**	**-1.642**
DEPDC7	Homo sapiens DEP domain containing 7 (DEPDC7), mRNA [NM_139160]	**-1.042**	**-1.177**
KIAA1466	Homo sapiens mRNA for KIAA1466 protein, partial cds. [AB040899]	**-1.040**	**-1.343**
NCRNA00115	Homo sapiens non-protein coding RNA 115 (NCRNA00115), non-coding RNA [NR_024321]	**-2.292**	**-1.038**
SHC3	Homo sapiens SHC (Src homology 2 domain containing) transforming protein 3 (SHC3), mRNA [NM_016848]	**-1.037**	**-1.106**
MYCBP	Homo sapiens c-myc binding protein (MYCBP), mRNA [NM_012333]	**-1.037**	**-1.100**
SALL2	Homo sapiens sal-like 2 (Drosophila) (SALL2), mRNA [NM_005407]	**-1.191**	**-1.034**
ZCRB1	Homo sapiens zinc finger CCHC-type and RNA binding motif 1 (ZCRB1),	**-1.034**	**-1.903**

	mRNA [NM_033114]		
LOC390413	Homo sapiens misc_RNA (LOC390413), miscRNA [XR_018341]	**-1.032**	**-2.125**
LOC729684	Homo sapiens misc_RNA (LOC729684), miscRNA [XR_039360]	**-1.026**	**-1.658**
PLA2R1	Human 180 kDa transmembrane PLA2 receptor mRNA, complete cds. [U17033]	**-1.025**	**-2.230**
IRAK1BP1	Homo sapiens interleukin-1 receptor-associated kinase 1 binding protein 1 (IRAK1BP1), mRNA [NM_001010844]	**-1.024**	**-1.080**
CENPK	Homo sapiens centromere protein K (CENPK), mRNA [NM_022145]	**-1.023**	**-1.724**
LOC389992	Homo sapiens similar to hCG2040259 (LOC389992), mRNA [XM_001720568]	**-1.023**	**-2.597**
BIC	Homo sapiens BIC transcript (BIC), non-coding RNA [NR_001458]	**-1.019**	**-1.059**
RACGAP1	Homo sapiens Rac GTPase activating protein 1 (RACGAP1),mRNA [NM_013277]	**-1.067**	**-1.014**
RAVER2	Homo sapiens cDNA FLJ10770 fis, clone NT2RP4000159. [AK001632]	**-1.338**	**-1.013**
AP4S1	Homo sapiens adaptor-related protein complex 4, sigma 1 subunit (AP4S1), mRNA [NM_001128126]	**-1.010**	**-1.152**
BEND3	Homo sapiens BEN domain containing 3 (BEND3), mRNA [NM_001080450]	**-1.006**	**-1.488**
TMSB15A	Homo sapiens thymosin-like 8 (TMSL8), mRNA [NM_021992]	**-2.391**	**-1.006**
LOC646993	Homo sapiens similar to high-mobility group box 3 (LOC646993), mRNA [XM_929965]	**-1.005**	**-1.516**
INSL4	Homo sapiens insulin-like 4 (placenta) (INSL4), mRNA [NM_002195]	**-1.151**	**-1.003**
LOC401588	Homo sapiens hypothetical LOC401588 (LOC401588), non-coding RNA [NR_015378]	**-1.132**	**-1.003**
LOC402360	Homo sapiens similar to hCG1742476 (LOC402360), mRNA [XM_001722407]	**-1.002**	**-1.188**
