# Supplementary material for: Genotoxicity and molecular response of silver nanoparticle (NP)-based hydrogel
Source: J Nanobiotechnology. 2012 May 1;10:16. doi: 10.1186/1477-3155-10-16 (PMC3430588; doi:10.1186/1477-3155-10-16)
Supplement: Additional file 11 — Common up-regulated genes in cells exposed to hydrogel for 24 h and 48 h. Fold-change is logarithmic ratio (log2 ratio) to expression level in control. [file 1477-3155-10-16-S11.pdf]

Additional File 11. Common up-regulated genes in cells exposed to hydrogel for 24 h and 48 h. Fold-change is logarithmic ratio (log_2 ratio) to expression level in control.

GeneName	Description	Fold-change (log_2ratio)(24h)	Fold-change (log_2ratio)(48h)
GDF2	Homo sapiens growth differentiation factor 2 (GDF2), mRNA [NM_016204]	**4.960**	**5.177**
PRKAG3	Homo sapiens protein kinase, AMP-activated, gamma 3 non-catalytic subunit (PRKAG3), mRNA [NM_017431]	**4.541**	**5.376**
LOC646976	Homo sapiens cDNA FLJ38763 fis, clone KIDNE2014119. [AK096082]	**2.838**	**3.100**
ANKRD31	Ankyrin repeat domain-containing protein 31 [Source:UniProtKB/Swiss-Prot;Acc:Q8N7Z5] [ENST00000274361]	**2.427**	**4.491**
CRAT	Homo sapiens carnitine acetyltransferase (CRAT),mRNA [NM_000755]	**1.380**	**1.270**
RPS16	Homo sapiens ribosomal protein S16 (RPS16), mRNA [NM_001020]	**1.428**	**1.269**
LOC100132673	Homo sapiens misc_RNA (LOC100132673), miscRNA [XR_039018]	**1.879**	**1.236**
GNB2L1	Homo sapiens guanine nucleotide binding protein (G protein), beta polypeptide 2-like 1 (GNB2L1), mRNA [NM_006098]	**1.812**	**1.227**
HOXA3	Homo sapiens homeobox A3 (HOXA3), mRNA [NM_153631]	**1.438**	**1.219**
RHOB	Homo sapiens ras homolog gene family, member B (RHOB), mRNA [NM_004040]	**2.239**	**1.162**
ANPEP	Homo sapiens alanyl (membrane) aminopeptidase (ANPEP), mRNA [NM_001150]	**1.384**	**1.161**
POLL	Homo sapiens polymerase (DNA directed), lambda (POLL), mRNA [NM_013274]	**1.193**	**1.142**
RPL18A	Homo sapiens ribosomal protein L18a (RPL18A), mRNA [NM_000980]	**1.830**	**1.109**
CYP1A1	Homo sapiens cytochrome P450, family 1, subfamily A, polypeptide 1 (CYP1A1), mRNA [NM_000499]	**1.104**	**1.271**
RCOR2	Homo sapiens REST corepressor 2 (RCOR2), mRNA [NM_173587]	**1.238**	**1.102**
SOS1	Homo sapiens son of sevenless homolog 1 (Drosophila) (SOS1), mRNA [NM_005633]	**1.338**	**1.092**
LOC731275	Homo sapiens hypothetical LOC731275 (LOC731275), mRNA [XM_001726998]	**1.132**	**1.091**
NKAIN4	Homo sapiens Na+/K+ transporting ATPase interacting 4 (NKAIN4), mRNA [NM_152864]	**1.447**	**1.086**
FHL3	Homo sapiens four and a half LIM domains 3 (FHL3), mRNA [NM_004468]	**1.394**	**1.082**
AGTRAP	Homo sapiens angiotensin II receptor-associated protein	**1.614**	**1.065**

	(AGTRAP), mRNA [NM_001040196]		
CAPNS1	Homo sapiens calpain, small subunit 1 (CAPNS1),mRNA [NM_001749]	**1.141**	**1.055**
GGT8P	Homo sapiens gamma-glutamyltransferase 8 pseudogene (GGT8P), non-coding RNA [NR_003503]	**1.585**	**1.050**
GAPDH	Homo sapiens glyceraldehyde-3-phosphate dehydrogenase (GAPDH), mRNA [NM_002046]	**2.412**	**1.046**
ASF1B	Homo sapiens ASF1 anti-silencing function 1 homolog B (S. cerevisiae) (ASF1B), mRNA [NM_018154]	**1.159**	**1.029**
ORM1	Homo sapiens orosomucoid 1 (ORM1), mRNA [NM_000607]	**1.955**	**1.026**
ACAP3	Homo sapiens ArfGAP with coiled-coil, ankyrin repeat and PH domains 3 (ACAP3), mRNA [NM_030649]	**1.278**	**1.023**
LCE1D	Homo sapiens late cornified envelope 1D (LCE1D), mRNA [NM_178352]	**1.133**	**1.006**
