# Supplementary material for: Genotoxicity and molecular response of silver nanoparticle (NP)-based hydrogel
Source: J Nanobiotechnology. 2012 May 1;10:16. doi: 10.1186/1477-3155-10-16 (PMC3430588; doi:10.1186/1477-3155-10-16)
Supplement: Additional file 12 — Common down-regulated genes in cells exposed to hydrogel for 24 h and 48 h. Fold-change is logarithmic ratio (log2 ratio) to expression level in control. [file 1477-3155-10-16-S12.pdf]

Additional File 12. Common down-regulated genes in cells exposed to hydrogel for 24 h and 48 h. Fold-change is logarithmic ratio (log_2 ratio) to expression level in control.

GeneName	Description	Fold-change (log_2ratio)(24h)	Fold-change (log_2ratio)(48h)
LOC100128519	Homo sapiens misc_RNA (LOC100128519), miscRNA [XR_038473]	**-2.415**	**-2.824**
LOC392335	Homo sapiens misc_RNA (LOC392335), miscRNA [XR_037043]	**-2.242**	**-2.207**
LOC653071	Homo sapiens similar to CG32820-PA, isoform A, mRNA (cDNA clone IMAGE:4812880), [BC068588]	**-2.200**	**-2.376**
CDC14A	Homo sapiens CDC14 cell division cycle 14 homolog A (S. cerevisiae) (CDC14A), mRNA [NM_003672]	**-2.193**	**-2.654**
LOC344178	Homo sapiens similar to hCG1794703 (LOC344178), mRNA [XM_001721796]	**-2.104**	**-2.285**
KIAA0802	Homo sapiens KIAA0802 (KIAA0802), mRNA [NM_015210]	**-1.937**	**-1.939**
HERC3	Homo sapiens hect domain and RLD 3, mRNA (cDNA clone IMAGE:6050308), [BC038960]	**-1.885**	**-2.902**
DAGLA	Homo sapiens diacylglycerol lipase, alpha (DAGLA), mRNA [NM_006133]	**-1.843**	**-2.241**
GOLGA8E	Homo sapiens golgi autoantigen, golgin subfamily a, 8E (GOLGA8E), mRNA [NM_001012423]	**-1.833**	**-1.865**
LOC389992	Homo sapiens similar to hCG2040259 (LOC389992), mRNA [XM_001720568]	**-1.830**	**-2.482**
MAGEA1	Homo sapiens melanoma antigen family A, 1 (directs expression of antigen MZ2-E) (MAGEA1), mRNA [NM_004988]	**-1.755**	**-2.158**
PL-5283	Homo sapiens PL-5283 protein (PL-5283), mRNA [NM_001130929]	**-1.891**	**-1.754**
SNHG10	Homo sapiens small nucleolar RNA host gene 10 (non-protein coding) (SNHG10), non-coding RNA [NR_003138]	**-1.710**	**-2.012**
KIAA0492	Homo sapiens mRNA, chromosome 1 specific transcript KIAA0492. [AB007961]	**-1.669**	**-2.239**
LOC728344	Homo sapiens misc_RNA (LOC728344), miscRNA [XR_018424]	**-1.613**	**-2.054**
ZNF319	Homo sapiens zinc finger protein 319 (ZNF319), mRNA [NM_020807]	**-2.097**	**-1.603**
NUDT12	Homo sapiens nudix (nucleoside diphosphate linked moiety X)-type motif 12 (NUDT12), mRNA [NM_031438]	**-1.785**	**-1.593**
EFCAB10	Homo sapiens cDNA clone IMAGE:6616931, partial cds. [BC062748]	**-1.581**	**-2.424**
ZNF509	Homo sapiens zinc finger protein 509 (ZNF509), mRNA [NM_145291]	**-1.563**	**-2.192**
SPATA9	Homo sapiens cDNA FLJ35906 fis, clone TESTI2009727. [AK093225]	**-1.561**	**-1.900**
LOC646446	Homo sapiens similar to hCG2040301 (LOC646446), mRNA [XM_001722653]	**-1.530**	**-2.322**

LOC203510	Homo sapiens similar to hCG1644442 (LOC203510), mRNA [XM_001719132]	**-1.526**	**-1.895**
ESF1	Homo sapiens ESF1, nucleolar pre-rRNA processing protein, homolog (ESF1), mRNA [NM_016649]	**-1.516**	**-1.785**
LOC653773	Homo sapiens misc_RNA (LOC653773), miscRNA [XR_042355]	**-1.491**	**-2.162**
LOC392382	Homo sapiens misc_RNA (LOC392382), miscRNA [XR_019110]	**-1.487**	**-2.146**
LOC100131149	Homo sapiens misc_RNA (LOC100131149), miscRNA [XR_039101]	**-1.475**	**-1.595**
LOC126235	Full-length cDNA clone CS0DE004YN04 of Placenta of Homo sapiens (human). [CR622909]	**-1.471**	**-1.676**
LOC391282	Homo sapiens similar to ribosomal protein L23a (LOC391282), mRNA [XM_372878]	**-1.468**	**-1.858**
LOC729684	Homo sapiens misc_RNA (LOC729684), miscRNA [XR_039360]	**-1.519**	**-1.447**
CCDC68	Homo sapiens coiled-coil domain containing 68 (CCDC68), mRNA [NM_025214]	**-1.440**	**-1.570**
ZCRB1	Homo sapiens zinc finger CCHC-type and RNA binding motif 1 (ZCRB1), mRNA [NM_033114]	**-1.439**	**-1.847**
ITM2B	Homo sapiens integral membrane protein 2B (ITM2B), mRNA [NM_021999]	**-1.422**	**-1.588**
ANO8	Homo sapiens anoctamin 8 (ANO8), mRNA [NM_020959]	**-1.420**	**-1.444**
LOC100132439	Homo sapiens similar to Protein FAM27E3 (LOC100132439), mRNA [XM_001719283]	**-1.904**	**-1.411**
TRIM66	Homo sapiens tripartite motif-containing 66 (TRIM66), mRNA [NM_014818]	**-1.398**	**-1.585**
FTMT	Homo sapiens ferritin mitochondrial (FTMT), mRNA [NM_177478]	**-1.396**	**-1.738**
LOC343495	Homo sapiens misc_RNA (LOC343495), miscRNA [XR_016540]	**-1.386**	**-2.374**
ZNF737	Homo sapiens zinc finger protein 737, mRNA (cDNA clone IMAGE:4854518), [BC015765]	**-1.382**	**-2.317**
SAR1P3	Homo sapiens SAR1 gene homolog (S. cerevisiae) pseudogene 3 (SAR1P3), mRNA [XM_001714154]	**-1.371**	**-2.114**
LOC390413	Homo sapiens misc_RNA (LOC390413), miscRNA [XR_018341]	**-1.369**	**-1.942**
LOC729332	Homo sapiens hypothetical LOC729332 (LOC729332), mRNA [XM_001129827]	**-1.366**	**-1.813**
MGC3207	Homo sapiens translation initiation factor eIF-2B subunit alpha/beta/delta-like protein (MGC3207), mRNA [NM_001031727]	**-1.973**	**-1.363**
LOC100132658	Homo sapiens misc_RNA (LOC100132658), miscRNA [XR_038952]	**-1.362**	**-2.428**
LOC100131323	Homo sapiens misc_RNA (LOC100131323), miscRNA [XR_039461]	**-1.349**	**-1.774**
LOC728774	Homo sapiens similar to hCG1994130 (LOC728774), mRNA [XM_001129390]	**-1.349**	**-1.980**

LOC645231	Full-length cDNA clone CS0DI026YJ08 of Placenta Cot 25-normalized of Homo sapiens (human). [CR590757]	**-1.338**	**-1.949**
RNF215	Homo sapiens ring finger protein 215 (RNF215), mRNA [NM_001017981]	**-1.335**	**-1.924**
FKSG2	Homo sapiens apoptosis inhibitor (FKSG2), mRNA [NM_021631]	**-1.333**	**-2.014**
ZNF486	Homo sapiens zinc finger protein 486 (ZNF486), mRNA [NM_052852]	**-1.333**	**-2.132**
TRUB1	Homo sapiens TruB pseudouridine (psi) synthase homolog 1 (E. coli) (TRUB1), mRNA [NM_139169]	**-1.332**	**-1.342**
LRP5L	Homo sapiens low density lipoprotein receptor-related protein 5-like (LRP5L), mRNA [NM_182492]	**-1.350**	**-1.326**
TBX15	Homo sapiens T-box 15 (TBX15), mRNA [NM_152380]	**-1.429**	**-1.326**
ARNT2	Homo sapiens aryl-hydrocarbon receptor nuclear translocator 2 (ARNT2), mRNA [NM_014862]	**-1.323**	**-1.892**
RPL32P3	Homo sapiens ribosomal protein L32 pseudogene 3 (RPL32P3), non-coding RNA [NR_003111]	**-1.321**	**-1.417**
EIF1B	Homo sapiens eukaryotic translation initiation factor 1B (EIF1B), mRNA [NM_005875]	**-1.371**	**-1.317**
TMEM129	Homo sapiens transmembrane protein 129 (TMEM129), mRNA [NM_138385]	**-1.309**	**-1.596**
ABCD4	Homo sapiens ATP-binding cassette, sub-family D (ALD), member 4 (ABCD4), mRNA [NM_005050]	**-1.323**	**-1.301**
ID2	Homo sapiens inhibitor of DNA binding 2, dominant negative helix-loop-helix protein (ID2), mRNA [NM_002166]	**-1.297**	**-3.766**
LOC730834	DB090170 TESTI4 Homo sapiens cDNA clone TESTI4038997 5', mRNA sequence [DB090170]	**-1.297**	**-1.517**
LOC130728	Homo sapiens misc_RNA (LOC130728), miscRNA [XR_019248]	**-1.292**	**-1.535**
LOC646993	Homo sapiens similar to high-mobility group box 3 (LOC646993), mRNA [XM_929965]	**-1.291**	**-1.469**
CENPK	Homo sapiens centromere protein K (CENPK), mRNA [NM_022145]	**-1.280**	**-1.351**
LOC100130171	Homo sapiens misc_RNA (LOC100130171), miscRNA [XR_038676]	**-1.274**	**-1.322**
LOC100128626	Homo sapiens misc_RNA (LOC100128626), miscRNA [XR_038662]	**-1.271**	**-1.899**
CBWD6	Homo sapiens clone 1659351 unknown mRNA. [AF293368]	**-1.270**	**-1.800**
USP11	Homo sapiens ubiquitin specific peptidase 11 (USP11), mRNA [NM_004651]	**-2.228**	**-1.268**
C10orf140	Homo sapiens chromosome 10 open reading frame 140 (C10orf140), mRNA [NM_207371]	**-1.267**	**-1.304**
RNF214	Homo sapiens ring finger protein 214 (RNF214),mRNA [NM_001077239]	**-1.561**	**-1.263**

PARP4	Homo sapiens poly (ADP-ribose) polymerase family, member 4 (PARP4), mRNA [NM_006437]	**-1.253**	**-1.747**
HIST1H3J	Homo sapiens histone cluster 1, H3j (HIST1H3J), mRNA [NM_003535]	**-1.252**	**-1.601**
LOC100132086	Homo sapiens misc_RNA (LOC100132086), miscRNA [XR_037769]	**-1.251**	**-2.270**
PLEKHA7	Homo sapiens pleckstrin homology domain containing, family A member 7 (PLEKHA7), mRNA [NM_175058]	**-1.441**	**-1.247**
RECQL	Homo sapiens RecQ protein-like (DNA helicase Q1-like) (RECQL), mRNA [NM_032941]	**-1.246**	**-1.275**
TMEM154	Homo sapiens transmembrane protein 154 (TMEM154), mRNA [NM_152680]	**-2.206**	**-1.228**
ZNF442	Homo sapiens zinc finger protein 442 (ZNF442), mRNA [NM_030824]	**-1.226**	**-1.512**
POLB	Homo sapiens polymerase (DNA directed), beta (POLB), mRNA [NM_002690]	**-1.220**	**-1.704**
IL17RB	Homo sapiens interleukin 17 receptor B (IL17RB), mRNA [NM_018725]	**-2.019**	**-1.219**
C18orf55	Homo sapiens chromosome 18 open reading frame 55 (C18orf55), mRNA [NM_014177]	**-1.322**	**-1.215**
LOC253482	Homo sapiens misc_RNA (LOC253482), miscRNA [XR_016415]	**-1.209**	**-1.252**
LOC643205	Homo sapiens hypothetical LOC643205 (LOC643205), mRNA [XM_001716733]	**-1.202**	**-1.592**
RPS6P1	Homo sapiens misc_RNA (RPS6P1), miscRNA [XR_016837]	**-1.201**	**-1.744**
NACAP1	Homo sapiens nascent-polypeptide-associated complex alpha polypeptide pseudogene 1 (NACAP1), non-coding RNA [NR_002182]	**-1.198**	**-1.621**
KIAA1524	Homo sapiens KIAA1524 (KIAA1524), mRNA [NM_020890]	**-1.236**	**-1.197**
SNRPG	Homo sapiens small nuclear ribonucleoprotein polypeptide G (SNRPG), mRNA [NM_003096]	**-1.195**	**-1.455**
MNAT1	Homo sapiens menage a trois homolog 1, cyclin H assembly factor (Xenopus laevis) (MNAT1), mRNA [NM_002431]	**-1.229**	**-1.186**
LOC728198	Homo sapiens similar to transcription associated factor TAFII31L (LOC728198), mRNA [XM_001126120]	**-1.481**	**-1.175**
FLJ27352	Homo sapiens cDNA FLJ27352 fis, clone TST05165. [AK130862]	**-1.174**	**-1.234**
LOC100128266	Homo sapiens misc_RNA (LOC100128266), miscRNA [XR_037888]	**-1.168**	**-1.938**
FAM84B	Homo sapiens family with sequence similarity 84, member B (FAM84B), mRNA [NM_174911]	**-1.517**	**-1.165**
FAM86A	Homo sapiens family with sequence similarity 86, member A (FAM86A), mRNA [NM_201400]	**-1.158**	**-1.235**
IKZF4	Homo sapiens IKAROS family zinc finger 4 (Eos) (IKZF4),	**-2.320**	**-1.158**

	mRNA [NM_022465]		
RPL31P4	Homo sapiens misc_RNA (LOC729646), miscRNA [XR_037308]	**-1.158**	**-1.456**
CLIP4	Homo sapiens CAP-GLY domain containing linker protein family, member 4 (CLIP4), mRNA [NM_024692]	**-1.457**	**-1.158**
LOC340508	Homo sapiens hypothetical protein LOC340508 (LOC340508), non-coding RNA [NR_002942]	**-1.480**	**-1.157**
SSBP1	Homo sapiens single-stranded DNA binding protein 1 (SSBP1), mRNA [NM_003143]	**-1.155**	**-1.434**
ZNF43	Homo sapiens zinc finger protein 43 (ZNF43), mRNA [NM_003423]	**-1.154**	**-1.929**
PABPC3	Homo sapiens poly(A) binding protein, cytoplasmic 3 (PABPC3), mRNA [NM_030979]	**-1.150**	**-1.828**
BAG4	BAG family molecular chaperone regulator 4 (BAG-4)(Bcl-2-associated athanogene 4) [Source:UniProtKB/ Swiss-Prot; Acc:O95429] [ENST00000287322]	**-1.149**	**-1.359**
LOC100128328	Homo sapiens hypothetical protein LOC100128328 (LOC100128328), mRNA [XM_001715053]	**-1.143**	**-1.482**
NSBP1	Homo sapiens nucleosomal binding protein 1 (NSBP1), mRNA [NM_030763]	**-1.169**	**-1.141**
LOC285550	Homo sapiens cDNA FLJ42660 fis, clone BRAMY2010808. [AK124651]	**-1.138**	**-1.216**
GNG2	Homo sapiens guanine nucleotide binding protein (G protein), gamma 2 (GNG2), mRNA [NM_053064]	**-1.135**	**-1.191**
CBR1	Homo sapiens carbonyl reductase 1 (CBR1), mRNA [NM_001757]	**-1.131**	**-1.131**
IRAK1BP1	Homo sapiens interleukin-1 receptor-associated kinase 1 binding protein 1 (IRAK1BP1), mRNA [NM_001010844]	**-1.128**	**-1.221**
LOC100129720	Full-length cDNA clone CS0DI044YN21 of Placenta Cot 25-normalized of Homo sapiens (human). [CR619772]	**-1.127**	**-1.534**
NUDT9	Homo sapiens nudix (nucleoside diphosphate linked moiety X)-type motif 9 (NUDT9), mRNA [NM_024047]	**-1.166**	**-1.125**
TUBA8	Homo sapiens tubulin, alpha 8 (TUBA8), mRNA [NM_018943]	**-1.127**	**-1.110**
LOC100125556	Homo sapiens family with sequence similarity 86, member A pseudogene (LOC100125556), non-coding RNA [NR_024251]	**-1.126**	**-1.105**
ZFP36L2	Homo sapiens zinc finger protein 36, C3H type-like 2 (ZFP36L2), mRNA [NM_006887]	**-1.090**	**-1.289**
C10orf10	Homo sapiens chromosome 10 open reading frame 10 (C10orf10), mRNA [NM_007021]	**-1.089**	**-1.269**
TMC8	Homo sapiens transmembrane channel-like 8 (TMC8), mRNA [NM_152468]	**-1.680**	**-1.079**
LOC100133154	Homo sapiens hypothetical protein LOC100133154 (LOC100133154), mRNA [XM_001714925]	**-2.118**	**-1.073**
HEATR5A	Homo sapiens HEAT repeat containing 5A (HEATR5A), mRNA	**-1.070**	**-1.330**

	[NM_015473]		
IPPK	Homo sapiens inositol 1,3,4,5,6-pentakisphosphate 2-kinase (IPPK), mRNA [NM_022755]	**-1.066**	**-1.143**
ADO	Homo sapiens 2-aminoethanethiol (cysteamine) dioxygenase (ADO), mRNA [NM_032804]	**-1.135**	**-1.065**
KLRC3	Homo sapiens killer cell lectin-like receptor subfamily C, member 3 (KLRC3), mRNA [NM_007333]	**-1.714**	**-1.064**
ZNF594	Homo sapiens zinc finger protein 594 (ZNF594), mRNA [NM_032530]	**-1.720**	**-1.058**
ZNF107	Homo sapiens zinc finger protein 107 (ZNF107),mRNA [NM_016220]	**-1.057**	**-1.084**
C5orf36	Homo sapiens chromosome 5 open reading frame 36 (C5orf36), mRNA [NM_173665]	**-1.053**	**-1.883**
FAM179A	Homo sapiens family with sequence similarity 179, member A (FAM179A), mRNA [NM_199280]	**-1.052**	**-1.927**
LOC400657	Homo sapiens hypothetical LOC400657 (LOC400657), non-coding RNA [NR_024484]	**-1.095**	**-1.047**
LOC730107	Homo sapiens similar to Glycine cleavage system H protein, mitochondrial (LOC730107), mRNA [XM_001721064]	**-1.047**	**-1.329**
C1orf198	Homo sapiens chromosome 1 open reading frame 198 (C1orf198), mRNA [NM_032800]	**-1.677**	**-1.039**
LOC257039	Homo sapiens similar to hCG2040268 (LOC257039), mRNA [XM_172230]	**-1.037**	**-1.338**
TBX3	Homo sapiens T-box 3 (TBX3), mRNA [NM_016569]	**-1.034**	**-1.495**
LOC100133528	Homo sapiens similar to HIG1 domain family, member 1A (LOC100133528), mRNA [XM_001721514]	**-1.106**	**-1.033**
GLRXL	Homo sapiens glutaredoxin (thioltransferase)-like (GLRXL), mRNA [NM_001123388]	**-1.241**	**-1.032**
EID2	Homo sapiens EP300 interacting inhibitor of differentiation 2 (EID2), mRNA [NM_153232]	**-1.257**	**-1.029**
FAM13C1	Homo sapiens family with sequence similarity 13, member C1 (FAM13C1), mRNA [NM_001001971]	**-1.028**	**-1.155**
RAP1B	Homo sapiens RAP1B, member of RAS oncogene family (RAP1B), mRNA [NM_015646]	**-1.138**	**-1.028**
TCF7L2	Homo sapiens transcription factor 7-like 2 (T-cell specific, HMG-box) (TCF7L2), mRNA [NM_030756]	**-1.028**	**-2.422**
FGFR1OP	Homo sapiens FGFR1 oncogene partner (FGFR1OP), mRNA [NM_007045]	**-1.026**	**-1.244**
BPNT1	Homo sapiens 3'(2'), 5'-bisphosphate nucleotidase 1 (BPNT1), mRNA [NM_006085]	**-1.021**	**-1.124**
LOC645294	Homo sapiens misc_RNA (LOC645294), miscRNA [XR_019042]	**-1.018**	**-1.956**
FAM175B	Homo sapiens family with sequence similarity 175, member B (FAM175B), mRNA [NM_032182]	**-1.013**	**-1.586**

NCRNA00081	Homo sapiens non-protein coding RNA 81 (NCRNA00081), non-coding RNA [NR_024140]	**-1.005**	**-1.052**
MYSM1	Homo sapiens mRNA for KIAA1915 protein, partial cds. [AB067502]	**-1.002**	**-1.712**
MED7	Homo sapiens mediator complex subunit 7 (MED7), mRNA [NM_004270]	**-1.000**	**-1.183**
