# Supplementary material for: Genotoxicity and molecular response of silver nanoparticle (NP)-based hydrogel
Source: J Nanobiotechnology. 2012 May 1;10:16. doi: 10.1186/1477-3155-10-16 (PMC3430588; doi:10.1186/1477-3155-10-16)
Supplement: Additional file 13 — Common up-regulated genes in cells exposed to hydrogel and silver-NP-hydrogel for 48h. Fold-change is logarithmic ratio (log2 ratio) to expression level in control. [file 1477-3155-10-16-S13.pdf]

Additional File 13. Common up-regulated genes in cells exposed to hydrogel and silver-NP-hydrogel for 48h. Fold-change is logarithmic ratio (log_2 ratio) to expression level in control.

GeneName	Description	Fold-change (log_2 ratio)
VCX3A	Homo sapiens variable charge, X-linked 3A (VCX3A), mRNA [NM_016379]	**1.476**
VCX2	Homo sapiens variable charge, X-linked 2 (VCX2), mRNA [NM_016378]	**1.335**
BASP1	Homo sapiens brain abundant, membrane attached signal protein 1 (BASP1), mRNA [NM_006317]	**1.287**
CYP1A1	Homo sapiens cytochrome P450, family 1, subfamily A, polypeptide 1 (CYP1A1), mRNA [NM_000499]	**1.271**
HOXA3	Homo sapiens homeobox A3 (HOXA3), mRNA [NM_153631]	**1.219**
CASP1	Homo sapiens caspase 1, apoptosis-related cysteine peptidase (interleukin 1, beta, convertase) (CASP1), mRNA [NM_033292]	**1.199**
IFIH1	Homo sapiens interferon induced with helicase C domain 1 (IFIH1), mRNA [NM_022168]	**1.194**
RHOB	Homo sapiens ras homolog gene family, member B (RHOB), mRNA [NM_004040]	**1.162**
ANPEP	Homo sapiens alanyl (membrane) aminopeptidase (ANPEP), mRNA [NM_001150]	**1.161**
ABCG2	Homo sapiens ATP-binding cassette, sub-family G (WHITE), member 2 (ABCG2), mRNA [NM_004827]	**1.159**
CRAT	Homo sapiens carnitine acetyltransferase (CRAT), nuclear gene encoding mitochondrial protein, mRNA [NM_000755]	**1.146**
C16orf3	Homo sapiens chromosome 16 open reading frame 3 (C16orf3), mRNA [NM_001214]	**1.143**
MAFA	Homo sapiens v-maf musculoaponeurotic fibrosarcoma oncogene homolog A (avian) (MAFA), mRNA [NM_201589]	**1.142**
RPL41	Homo sapiens ribosomal protein L41 (RPL41), mRNA [NM_001035267]	**1.107**
DUSP27	Homo sapiens dual specificity phosphatase 27 (putative) (DUSP27), mRNA [NM_001080426]	**1.103**
RCOR2	Homo sapiens REST corepressor 2 (RCOR2), mRNA [NM_173587]	**1.102**
FBLN2	Homo sapiens fibulin 2 (FBLN2), mRNA [NM_001004019]	**1.099**
HLA-DMA	Homo sapiens major histocompatibility complex, class II, DM alpha (HLA-DMA), mRNA [NM_006120]	**1.097**
VCX	Homo sapiens variable charge, X-linked (VCX), mRNA [NM_013452]	**1.092**
RRAD	Homo sapiens Ras-related associated with diabetes (RRAD), mRNA [NM_004165]	**1.086**
NPB	Homo sapiens cDNA clone IMAGE:5019903, partial cds. [BC073815]	**1.073**
AGTRAP	Homo sapiens angiotensin II receptor-associated protein (AGTRAP), mRNA [NM_001040196]	**1.065**
FGL1	Homo sapiens fibrinogen-like 1 (FGL1), mRNA [NM_201553]	**1.060**
SLC12A7	Homo sapiens solute carrier family 12 (potassium/chloride transporters), member 7 (SLC12A7), mRNA [NM_006598]	**1.058**
GGT8P	Homo sapiens gamma-glutamyltransferase 8 pseudogene (GGT8P), non-coding RNA [NR_003503]	**1.050**
AMDHD1	Homo sapiens amidohydrolase domain containing 1 (AMDHD1), mRNA	**1.047**

	[NM_152435]	
GAPDH	Homo sapiens glyceraldehyde-3-phosphate dehydrogenase (GAPDH), mRNA [NM_002046]	**1.046**
KIAA1486	Homo sapiens KIAA1486 protein (KIAA1486), mRNA [NM_020864]	**1.039**
FIBIN	Homo sapiens fin bud initiation factor homolog (zebrafish) (FIBIN), mRNA [NM_203371]	**1.033**
CTSS	Homo sapiens cathepsin S (CTSS), mRNA [NM_004079]	**1.026**
TAP2	Homo sapiens transporter 2, ATP-binding cassette, sub-family B (MDR/TAP) (TAP2), mRNA [NM_000544]	**1.019**
MPL	Homo sapiens myeloproliferative leukemia virus oncogene (MPL), mRNA [NM_005373]	**1.011**
C1S	Homo sapiens complement component 1, s subcomponent (C1S), mRNA [NM_001734]	**1.008**
