# Supplementary material for: Genotoxicity and molecular response of silver nanoparticle (NP)-based hydrogel
Source: J Nanobiotechnology. 2012 May 1;10:16. doi: 10.1186/1477-3155-10-16 (PMC3430588; doi:10.1186/1477-3155-10-16)
Supplement: Additional file 14 — Common down-regulated genes in cells exposed to hydrogel and silver-NP-hydrogel for 48h. Fold-change is logarithmic ratio (log2 ratio) to expression level in control. [file 1477-3155-10-16-S14.pdf]

Additional File 14. Common down-regulated genes in cells exposed to hydrogel and silver-NP-hydrogel for 48h. Fold-change is logarithmic ratio (log_2 ratio) to expression level in control.

GeneName	Description	Fold-change (log_2 ratio)
ID2	Homo sapiens inhibitor of DNA binding 2, dominant negative helix-loop-helix protein (ID2), mRNA [NM_002166]	**-3.424**
TRIM73	Homo sapiens tripartite motif-containing 73, mRNA (cDNA clone IMAGE:5181460). [BC033812]	**-2.989**
LOC100128519	Homo sapiens misc_RNA (LOC100128519), miscRNA [XR_038473]	**-2.824**
CDC14A	Homo sapiens CDC14 cell division cycle 14 homolog A (S. cerevisiae) (CDC14A), mRNA [NM_003672]	**-2.654**
HSPD1P5	Homo sapiens misc_RNA (LOC345041), miscRNA [XR_018747]	**-2.601**
LOC389992	Homo sapiens similar to hCG2040259 (LOC389992), mRNA [XM_001720568]	**-2.482**
HERC3	Homo sapiens hect domain and RLD 3, mRNA (cDNA clone IMAGE:6050308). [BC038960]	**-2.476**
LOC100132658	Homo sapiens misc_RNA (LOC100132658), miscRNA [XR_038952]	**-2.428**
EFCAB10	Homo sapiens cDNA clone IMAGE:6616931. [BC062748]	**-2.424**
LOC653071	Homo sapiens similar to CG32820-PA, isoform A, mRNA (cDNA clone IMAGE:4812880), [BC068588]	**-2.376**
LOC343495	Homo sapiens misc_RNA (LOC343495), miscRNA [XR_016540]	**-2.374**
LOC646446	Homo sapiens similar to hCG2040301 (LOC646446), mRNA [XM_001722653]	**-2.322**
ZNF737	Homo sapiens zinc finger protein 737, mRNA (cDNA clone IMAGE:4854518), [BC015765]	**-2.317**
ARVCF	Homo sapiens armadillo repeat gene deletes in velocardiofacial syndrome (ARVCF), mRNA [NM_001670]	**-2.299**
LOC344178	Homo sapiens similar to hCG1794703 (LOC344178), mRNA [XM_001721796]	**-2.285**
LOC100132086	Homo sapiens misc_RNA (LOC100132086), miscRNA [XR_037769]	**-2.270**
UBE2D3P	Homo sapiens similar to Putative ubiquitin-conjugating enzyme E2 D3-like protein (LOC100128647), mRNA [XM_001725173]	**-2.261**
DAGLA	Homo sapiens diacylglycerol lipase, alpha (DAGLA), mRNA [NM_006133]	**-2.241**
KIAA0492	Homo sapiens mRNA, chromosome 1 specific transcript KIAA0492. [AB007961]	**-2.239**
ZNF509	Homo sapiens zinc finger protein 509 (ZNF509), mRNA [NM_145291]	**-2.192**
LOC392335	Homo sapiens misc_RNA (LOC392335), miscRNA [XR_037043]	**-2.181**
LOC653773	Homo sapiens misc_RNA (LOC653773), partial miscRNA [XR_042355]	**-2.162**
MAGEA1	Homo sapiens melanoma antigen family A, 1 (directs expression of antigen MZ2-E) (MAGEA1), mRNA [NM_004988]	**-2.158**
LOC392382	Homo sapiens misc_RNA (LOC392382), miscRNA [XR_019110]	**-2.146**
TCF7L2	Homo sapiens transcription factor 7-like 2 (T-cell specific, HMG-box) (TCF7L2), mRNA [NM_030756]	**-2.127**
SAR1P3	Homo sapiens SAR1 gene homolog (S. cerevisiae) pseudogene 3 (SAR1P3), mRNA [XM_001714154]	**-2.114**

LOC728344	Homo sapiens misc_RNA (LOC728344), miscRNA [XR_018424]	**-2.054**
LOC643015	Homo sapiens misc_RNA (LOC643015), miscRNA [XR_018290]	**-2.039**
LOC283523	Homo sapiens similar to telomeric repeat binding factor (NIMA-interacting) 1 (LOC283523), mRNA [XM_001714856]	**-2.030**
LOC441666	Homo sapiens zinc finger protein 91 pseudogene (LOC441666), non-coding RNA [NR_024380]	**-2.025**
FKSG2	Homo sapiens apoptosis inhibitor (FKSG2), mRNA [NM_021631]	**-2.014**
SNHG10	Homo sapiens small nucleolar RNA host gene 10 (non-protein coding) (SNHG10), non-coding RNA [NR_003138]	**-2.012**
LOC728774	Homo sapiens similar to hCG1994130 (LOC728774), mRNA [XM_001129390]	**-1.980**
LOC100128701	Homo sapiens misc_RNA (LOC100128701), miscRNA [XR_037462]	**-1.960**
LOC645231	Full-length cDNA clone CS0DI026YJ08 of Placenta Cot 25-normalized of Homo sapiens (human). [CR590757]	**-1.949**
ID1	Homo sapiens inhibitor of DNA binding 1, dominant negative helix-loop-helix protein (ID1), mRNA [NM_002165]	**-1.943**
LOC390413	Homo sapiens misc_RNA (LOC390413), miscRNA [XR_018341]	**-1.942**
KIAA0802	Homo sapiens KIAA0802 (KIAA0802), mRNA [NM_015210]	**-1.939**
LOC100128266	Homo sapiens misc_RNA (LOC100128266), miscRNA [XR_037888]	**-1.938**
ZNF43	Homo sapiens zinc finger protein 43 (ZNF43), mRNA [NM_003423]	**-1.929**
FAM179A	Homo sapiens family with sequence similarity 179, member A (FAM179A), mRNA [NM_199280]	**-1.927**
SPATA9	Homo sapiens cDNA FLJ35906 fis, clone TESTI2009727. [AK093225]	**-1.900**
LOC203510	Homo sapiens similar to hCG1644442 (LOC203510), mRNA [XM_001719132]	**-1.895**
ARNT2	Homo sapiens aryl-hydrocarbon receptor nuclear translocator 2 (ARNT2), mRNA [NM_014862]	**-1.892**
C5orf36	Homo sapiens chromosome 5 open reading frame 36 (C5orf36), mRNA [NM_173665]	**-1.883**
ZNF486	Homo sapiens zinc finger protein 486 (ZNF486), mRNA [NM_052852]	**-1.874**
GOLGA8E	Homo sapiens golgi autoantigen, golgin subfamily a, 8E (GOLGA8E), mRNA [NM_001012423]	**-1.865**
LOC391282	Homo sapiens similar to ribosomal protein L23a (LOC391282), mRNA [XM_372878]	**-1.851**
ZCRB1	Homo sapiens zinc finger CCHC-type and RNA binding motif 1 (ZCRB1), mRNA [NM_033114]	**-1.847**
PABPC3	Homo sapiens poly(A) binding protein, cytoplasmic 3 (PABPC3), mRNA [NM_030979]	**-1.828**
tcag7.873	Homo sapiens hypothetical LOC402644 (LOC402644), mRNA [NM_001126493]	**-1.814**
LOC729332	PREDICTED: Homo sapiens hypothetical LOC729332 (LOC729332), mRNA [XM_001129827]	**-1.813**
RPS12	Homo sapiens ribosomal protein S12 (RPS12), mRNA [NM_001016]	**-1.806**
CBWD6	Homo sapiens clone 1659351 unknown mRNA. [AF293368]	**-1.800**
GSPT2	Homo sapiens G1 to S phase transition 2 (GSPT2), mRNA [NM_018094]	**-1.787**

LOC390876	Homo sapiens similar to ribosomal protein L35 (LOC390876), mRNA [XM_001718967]	**-1.786**
LOC100131323	Homo sapiens misc_RNA (LOC100131323), miscRNA [XR_039461]	**-1.774**
LOC100130794	Homo sapiens similar to NADH dehydrogenase [ubiquinone] iron-sulfur protein 5 (NADH-ubiquinone oxidoreductase 15 kDa subunit) (LOC100130794), mRNA [XM_001724177]	**-1.759**
LOC100130518	Zinc finger protein ENSP00000350085 [Source:UniProtKB/Swiss-Prot;Acc: A6NN14] [ENST00000357491]	**-1.750**
PARP4	Homo sapiens poly (ADP-ribose) polymerase family, member 4 (PARP4), mRNA [NM_006437]	**-1.747**
RPS6P1	Homo sapiens misc_RNA (RPS6P1), miscRNA [XR_016837]	**-1.744**
ZNF479	Homo sapiens zinc finger protein 479 (ZNF479), mRNA [NM_033273]	**-1.743**
C18orf32	Homo sapiens chromosome 18 open reading frame 32 (C18orf32), mRNA [NM_001035005]	**-1.727**
ESF1	Homo sapiens ESF1, nucleolar pre-rRNA processing protein, homolog (S. cerevisiae) (ESF1), mRNA [NM_016649]	**-1.726**
LOC388532	Homo sapiens hypothetical LOC388532 (LOC388532), mRNA [XM_001721671]	**-1.723**
LOC643981	Homo sapiens misc_RNA (LOC643981), miscRNA [XR_018444]	**-1.718**
MYSM1	Homo sapiens mRNA for KIAA1915 protein. [AB067502]	**-1.712**
PL-5283	Homo sapiens PL-5283 protein (PL-5283), mRNA [NM_001130929]	**-1.705**
TCEB1P3	Homo sapiens transcription elongation factor B (SIII), polypeptide 1 (15kDa, elongin C) pseudogene 3 (TCEB1P3), mRNA [XM_927664]	**-1.700**
SUMO1P3	Homo sapiens SUMO1 pseudogene 3 (SUMO1P3), non-coding RNA [NR_002190]	**-1.689**
LOC100130211	Homo sapiens similar to translation elongation factor 1 alpha 1-like 14 (LOC100130211), mRNA [XM_001717632]	**-1.681**
LOC400061	Homo sapiens misc_RNA (LOC400061), miscRNA [XR_019557]	**-1.671**
LOC100128626	Homo sapiens misc_RNA (LOC100128626), miscRNA [XR_038662]	**-1.666**
LOC100129743	Homo sapiens similar to hCG2042936 (LOC100129743), mRNA [XM_001719980]	**-1.642**
NACAP1	Homo sapiens nascent-polypeptide-associated complex alpha polypeptide pseudogene 1 (NACAP1), non-coding RNA [NR_002182]	**-1.621**
LOC400750	Homo sapiens misc_RNA (LOC400750), miscRNA [XR_039070]	**-1.619**
HIST1H3J	Homo sapiens histone cluster 1, H3j (HIST1H3J), mRNA [NM_003535]	**-1.601**
NUDT12	Homo sapiens nudix (nucleoside diphosphate linked moiety X)-type motif 12 (NUDT12), mRNA [NM_031438]	**-1.593**
LOC643205	Homo sapiens hypothetical LOC643205 (LOC643205), mRNA [XM_001716733]	**-1.592**
ITM2B	Homo sapiens integral membrane protein 2B (ITM2B), mRNA [NM_021999]	**-1.588**
TMEM129	Homo sapiens transmembrane protein 129 (TMEM129), mRNA [NM_138385]	**-1.587**
FAM175B	Homo sapiens family with sequence similarity 175, member B (FAM175B), mRNA [NM_032182]	**-1.586**

LOC442459	Homo sapiens X-ray repair complementing defective repair pseudogene (LOC442459), non-coding RNA [NR_024608]	**-1.586**
TRIM66	Homo sapiens tripartite motif-containing 66 (TRIM66), mRNA [NM_014818]	**-1.585**
SMN2	Homo sapiens survival of motor neuron 2, centromeric (SMN2), mRNA [NM_022877]	**-1.578**
POLB	Homo sapiens polymerase (DNA directed), beta (POLB), mRNA [NM_002690]	**-1.572**
LOC100131581	Homo sapiens cDNA FLJ35225 fis, clone PROST2001116. [AK092544]	**-1.570**
hCG_1771830	Homo sapiens zinc finger protein LOC654254 (LOC654254), mRNA [NM_001137608]	**-1.568**
LOC728927	Homo sapiens cDNA FLJ57041 complete cds, moderately similar to Zinc finger protein 92. [AK301806]	**-1.566**
RPL31P10	Homo sapiens misc_RNA (RPL31P10), miscRNA [XR_018695]	**-1.560**
LOC730167	Homo sapiens similar to protein tyrosine phosphatase 4a1 (LOC730167), mRNA [XM_001134097]	**-1.552**
ZNF141	Homo sapiens zinc finger protein 141 (ZNF141), mRNA [NM_003441]	**-1.545**
LOC100131149	Homo sapiens misc_RNA (LOC100131149), miscRNA [XR_039101]	**-1.535**
LOC130728	Homo sapiens misc_RNA (LOC130728), miscRNA [XR_019248]	**-1.535**
LOC646973	Homo sapiens similar to eukaryotic translation elongation factor 1 beta 2 (LOC646973), mRNA [XR_019410]	**-1.526**
TBC1D3B	Homo sapiens TBC1 domain family, member 3B (TBC1D3B), mRNA [NM_001001417]	**-1.523**
LOC730834	DB090170 TESTI4 Homo sapiens cDNA clone TESTI4038997 5', mRNA sequence [DB090170]	**-1.517**
WWC2	Homo sapiens WW and C2 domain containing 2 (WWC2), mRNA [NM_024949]	**-1.513**
LOC645294	Homo sapiens misc_RNA (LOC645294), miscRNA [XR_019042]	**-1.507**
ANKRD50	Homo sapiens ankyrin repeat domain 50 (ANKRD50), mRNA [NM_020337]	**-1.503**
LOC376693	Homo sapiens hypothetical LOC376693, mRNA (cDNA clone MGC:45392 IMAGE:5526694), complete cds. [BC030568]	**-1.503**
LOC729046	Homo sapiens misc_RNA (LOC729046), miscRNA [XR_015710]	**-1.493**
ZNF319	Homo sapiens zinc finger protein 319 (ZNF319), mRNA [NM_020807]	**-1.492**
LOC644384	Full-length cDNA clone CS0DI028YD16 of Placenta Cot 25-normalized of Homo sapiens (human). [CR595167]	**-1.483**
LOC100128328	Homo sapiens hypothetical protein LOC100128328 (LOC100128328), mRNA [XM_001715053]	**-1.482**
LOC646993	Homo sapiens similar to high-mobility group box 3 (LOC646993), mRNA [XM_929965]	**-1.469**
SNRPG	Homo sapiens small nuclear ribonucleoprotein polypeptide G (SNRPG), mRNA [NM_003096]	**-1.455**
HSP90AB6P	Homo sapiens heat shock protein 90Bf (HSP90Bf) mRNA. [AY956767]	**-1.450**
LOC100128203	Homo sapiens similar to hCG2040272 (LOC100128203), mRNA [XM_001715724]	**-1.449**
ZNF665	Homo sapiens zinc finger protein 665 (ZNF665), mRNA [NM_024733]	**-1.448**

LOC729684	Homo sapiens misc_RNA (LOC729684), miscRNA [XR_039360]	**-1.447**
ZNF730	Homo sapiens cDNA FLJ16640 fis, clone TESTI4028938, moderately similar to Zinc finger protein 85. [AK131472]	**-1.447**
ANO8	Homo sapiens anoctamin 8 (ANO8), mRNA [NM_020959]	**-1.444**
LOC646909	Homo sapiens misc_RNA (LOC646909), miscRNA [XR_019013]	**-1.442**
LOC100129742	Homo sapiens misc_RNA (LOC100129742), miscRNA [XR_036968]	**-1.437**
SSBP1	Homo sapiens single-stranded DNA binding protein 1 (SSBP1), mRNA [NM_003143]	**-1.434**
LOC646999	Homo sapiens hypothetical LOC646999 (LOC646999), non-coding RNA [NR_024390]	**-1.430**
PTBP1	Homo sapiens polypyrimidine tract binding protein 1 (PTBP1), mRNA [NM_002819]	**-1.418**
RPL32P3	Homo sapiens ribosomal protein L32 pseudogene 3 (RPL32P3), non-coding RNA [NR_003111]	**-1.417**
ZNF850P	Homo sapiens mRNA; cDNA DKFZp686J154 (from clone DKFZp686J154). [CR627133]	**-1.416**
FRG1B	Homo sapiens FSHD region gene 1 family, member B (FRG1B), non-coding RNA [NR_003579]	**-1.414**
C9orf41	UPF0586 protein C9orf41 [Source:UniProtKB/Swiss-Prot;Acc:Q8N4J0] [ENST00000376834]	**-1.412**
LOC100132439	Homo sapiens similar to Protein FAM27E3 (LOC100132439), mRNA [XM_001719283]	**-1.411**
C3orf64	Homo sapiens chromosome 3 open reading frame 64 (C3orf64), mRNA [NM_173654]	**-1.405**
ZNF728	Homo sapiens similar to Zinc finger protein 208 (LOC388523), mRNA [XM_001726961]	**-1.405**
EIF1AY	Homo sapiens eukaryotic translation initiation factor 1A, Y-linked (EIF1AY), mRNA [NM_004681]	**-1.392**
RPL31P4	Homo sapiens misc_RNA (LOC729646), miscRNA [XR_037308]	**-1.387**
LOC442160	Homo sapiens misc_RNA (LOC442160), miscRNA [XR_042339]	**-1.386**
LOC100128974	Homo sapiens misc_RNA (LOC100128974), miscRNA [XR_037045]	**-1.382**
FMC1	Homo sapiens formation of mitochondrial complexes 1 homolog (S. cerevisiae) (FMC1), nuclear gene encoding mitochondrial protein, mRNA [NM_197964]	**-1.369**
LOC389842	Homo sapiens similar to RanBP1 (LOC389842), mRNA [XM_372200]	**-1.366**
ZNF675	Homo sapiens zinc finger protein 675 (ZNF675), mRNA [NM_138330]	**-1.364**
MGC3207	Homo sapiens translation initiation factor eIF-2B subunit alpha/beta/delta-like protein (MGC3207), mRNA [NM_001031727]	**-1.363**
BAG4	BAG family molecular chaperone regulator 4 (BAG-4)(Bcl-2-associated athanogene4)[Source:UniProtKB/Swiss-Prot;Acc:O95429] [ENST00000287322]	**-1.359**
ZNF680	Homo sapiens zinc finger protein 680 (ZNF680), mRNA [NM_178558]	**-1.358**
LMNB2	Homo sapiens lamin B2 (LMNB2), mRNA [NM_032737]	**-1.355**
CENPK	Homo sapiens centromere protein K (CENPK), mRNA [NM_022145]	**-1.351**
hCG_18290	Homo sapiens hCG18290 (LOC644907), mRNA [NM_001090027]	**-1.350**

LOC100131737	Homo sapiens misc_RNA (LOC100131737), miscRNA [XR_038332]	**-1.350**
LOC257039	Homo sapiens similar to hCG2040268 (LOC257039), mRNA [XM_172230]	**-1.338**
TRUB1	Homo sapiens TruB pseudouridine (psi) synthase homolog 1 (E. coli) (TRUB1), mRNA [NM_139169]	**-1.338**
DKFZp313P036	Homo sapiens mRNA; cDNA DKFZp313P036 (from clone DKFZp313P036). [BX537874]	**-1.335**
LOC100130289	Homo sapiens misc_RNA (LOC100130289), miscRNA [XR_039183]	**-1.333**
LOC730107	Homo sapiens similar to Glycine cleavage system H protein, mitochondrial (LOC730107), mRNA [XM_001721064]	**-1.329**
LRP5L	Homo sapiens low density lipoprotein receptor-related protein 5-like (LRP5L), mRNA [NM_182492]	**-1.326**
ZNF429	Homo sapiens zinc finger protein 429 (ZNF429), mRNA [NM_001001415]	**-1.324**
USP38	Homo sapiens ubiquitin specific peptidase 38 (USP38), mRNA [NM_032557]	**-1.323**
LOC100130171	Homo sapiens misc_RNA (LOC100130171), miscRNA [XR_038676]	**-1.322**
LOC341378	Homo sapiens similar to Golgi-associated microtubule-binding protein (LOC341378), mRNA [XM_001715424]	**-1.319**
EIF1B	Homo sapiens eukaryotic translation initiation factor 1B (EIF1B), mRNA [NM_005875]	**-1.317**
PMCH	Homo sapiens pro-melanin-concentrating hormone (PMCH), mRNA [NM_002674]	**-1.315**
C10orf140	Homo sapiens chromosome 10 open reading frame 140 (C10orf140), mRNA [NM_207371]	**-1.304**
ABCD4	Homo sapiens ATP-binding cassette, sub-family D (ALD), member 4 (ABCD4), mRNA [NM_005050]	**-1.301**
DHX40	Homo sapiens DEAH (Asp-Glu-Ala-His) box polypeptide 40 (DHX40), mRNA [NM_024612]	**-1.301**
LOC344328	Homo sapiens misc_RNA (LOC344328), miscRNA [XR_019373]	**-1.296**
ZNF85	Homo sapiens zinc finger protein 85 (ZNF85), mRNA [NM_003429]	**-1.292**
tcag7.1239	Homo sapiens misc_RNA (LOC643438), miscRNA [XR_015268]	**-1.290**
LOC641844	Homo sapiens misc_RNA (LOC641844), miscRNA [XR_018036]	**-1.284**
KIAA0831	Homo sapiens KIAA0831 (KIAA0831), mRNA [NM_014924]	**-1.279**
RECQL	Homo sapiens RecQ protein-like (DNA helicase Q1-like) (RECQL), mRNA [NM_032941]	**-1.275**
ZNF676	Homo sapiens zinc finger protein 676 (ZNF676), mRNA [NM_001001411]	**-1.271**
MICALCL	Homo sapiens MICAL C-terminal like (MICALCL), mRNA [NM_032867]	**-1.270**
INTS2	Homo sapiens integrator complex subunit 2 (INTS2), mRNA [NM_020748]	**-1.268**
USP11	Homo sapiens ubiquitin specific peptidase 11 (USP11), mRNA [NM_004651]	**-1.268**
TMEM181	Homo sapiens transmembrane protein 181 (TMEM181), mRNA [NM_020823]	**-1.264**
LOC645683	Homo sapiens ribosomal protein L13a pseudogene (LOC645683), non-coding RNA [NR_004844]	**-1.256**
LOC643014	Homo sapiens misc_RNA (LOC643014), miscRNA [XR_018450]	**-1.255**
SVIP	Homo sapiens small VCP/p97-interacting protein (SVIP), mRNA [NM_148893]	**-1.255**
LOC253482	Homo sapiens misc_RNA (LOC253482), miscRNA [XR_016415]	**-1.252**

ACAA2	Homo sapiens acetyl-Coenzyme A acyltransferase 2 (ACAA2), nuclear gene encoding mitochondrial protein, mRNA [NM_006111]	**-1.251**
LOC440737	Homo sapiens similar to ribosomal protein L35 (LOC440737), mRNA [XM_496446]	**-1.249**
LOC339843	Homo sapiens misc_RNA (LOC339843), miscRNA [XR_016598]	**-1.247**
CRYAA	Homo sapiens crystallin, alpha A (CRYAA), mRNA [NM_000394]	**-1.245**
ZNF626	Homo sapiens zinc finger protein 626 (ZNF626), mRNA [NM_001076675]	**-1.243**
RASSF5	Homo sapiens Ras association (RalGDS/AF-6) domain family member 5 (RASSF5), mRNA [NM_182663]	**-1.238**
CAPZA2	Homo sapiens capping protein (actin filament) muscle Z-line, alpha 2 (CAPZA2), mRNA [NM_006136]	**-1.237**
FAM86A	Homo sapiens family with sequence similarity 86, member A (FAM86A), mRNA [NM_201400]	**-1.235**
IL17RB	Homo sapiens interleukin 17 receptor B (IL17RB), mRNA [NM_018725]	**-1.219**
LOC285550	Homo sapiens cDNA FLJ42660 fis, clone BRAMY2010808. [AK124651]	**-1.216**
C18orf55	Homo sapiens chromosome 18 open reading frame 55 (C18orf55), mRNA [NM_014177]	**-1.215**
DNAJC5	Full-length cDNA clone CS0DN003YL17 of Adult brain of Homo sapiens (human). [CR607484]	**-1.213**
LOC90834	Homo sapiens, clone IMAGE:3535910, mRNA. [BC001742]	**-1.213**
SRP14P1	Homo sapiens signal recognition particle 14kDa (homologous Alu RNA binding protein) pseudogene 1 (SRP14P1), non-coding RNA [NR_003273]	**-1.213**
FBXO10	Homo sapiens F-box protein 10 (FBXO10), mRNA [NM_012166]	**-1.204**
ZNF345	Homo sapiens zinc finger protein 345 (ZNF345), mRNA [NM_003419]	**-1.204**
LOC646119	Homo sapiens similar to hCG2040247 (LOC646119), mRNA [XM_929084]	**-1.200**
KIAA1524	Homo sapiens KIAA1524 (KIAA1524), mRNA [NM_020890]	**-1.197**
LOC401076	Homo sapiens misc_RNA (LOC401076), miscRNA [XR_018154]	**-1.194**
AL022344.6	Homo sapiens similar to hCG1640833 (LOC100129622), mRNA [XM_001724175]	**-1.189**
ZNF826	Homo sapiens zinc finger protein 826 (ZNF826), mRNA [NM_001039884]	**-1.188**
SPINK5L3	Homo sapiens serine PI Kazal type 5-like 3 (SPINK5L3), mRNA [NM_001040129]	**-1.187**
MNAT1	Homo sapiens menage a trois homolog 1, cyclin H assembly factor (Xenopus laevis) (MNAT1), mRNA [NM_002431]	**-1.186**
PLCXD3	Homo sapiens phosphatidylinositol-specific phospholipase C, X domain containing 3 (PLCXD3), mRNA [NM_001005473]	**-1.185**
KIAA1586	Homo sapiens KIAA1586 (KIAA1586), mRNA [NM_020931]	**-1.184**
MED7	Homo sapiens mediator complex subunit 7 (MED7), mRNA [NM_004270]	**-1.183**
INE1	Homo sapiens inactivation escape 1 (non-protein coding) (INE1), non-coding RNA [NR_024616]	**-1.178**
NSL1	Homo sapiens NSL1, MIND kinetochore complex component, homolog (S. cerevisiae) (NSL1), mRNA [NM_015471]	**-1.178**
ZIC2	Homo sapiens Zic family member 2 (odd-paired homolog, Drosophila) (ZIC2),	**-1.173**

	mRNA [NM_007129]	
STX2	Homo sapiens syntaxin 2 (STX2), mRNA [NM_001980]	**-1.168**
NACA2	Homo sapiens nascent polypeptide-associated complex alpha subunit 2 (NACA2), mRNA [NM_199290]	**-1.162**
LOC729608	Homo sapiens similar to Brix domain containing 1 (LOC729608), mRNA [XM_001130778]	**-1.161**
LOC731688	Homo sapiens misc_RNA (LOC731688), miscRNA [XR_015992]	**-1.160**
LOC340508	Homo sapiens hypothetical protein LOC340508 (LOC340508), non-coding RNA [NR_002942]	**-1.157**
ZNF223	Homo sapiens zinc finger protein 223 (ZNF223), mRNA [NM_013361]	**-1.157**
APBA3	Homo sapiens amyloid beta (A4) precursor protein-binding, family A, member 3 (APBA3), mRNA [NM_004886]	**-1.156**
FAM13C1	Homo sapiens family with sequence similarity 13, member C1 (FAM13C1), mRNA [NM_001001971]	**-1.155**
LYSMD3	Homo sapiens LysM, putative peptidoglycan-binding, domain containing 3 (LYSMD3), mRNA [NM_198273]	**-1.151**
IKZF4	Homo sapiens IKAROS family zinc finger 4 (Eos) (IKZF4), mRNA [NM_022465]	**-1.148**
ITGA2	Homo sapiens integrin, alpha 2 (CD49B, alpha 2 subunit of VLA-2 receptor) (ITGA2), mRNA [NM_002203]	**-1.146**
LOC392522	Homo sapiens misc_RNA (LOC392522), miscRNA [XR_018292]	**-1.146**
hCG_21078	Homo sapiens hCG21078 (LOC389435), mRNA [NM_001089587]	**-1.144**
IPPK	Homo sapiens inositol 1,3,4,5,6-pentakisphosphate 2-kinase (IPPK), mRNA [NM_022755]	**-1.143**
PRR11	Homo sapiens cDNA FLJ11029 fis, clone PLACE1004156. [AK001891]	**-1.143**
LOC440396	Homo sapiens similar to Heterogeneous nuclear ribonucleoprotein A1 (hnRNP core protein A1) (HDP-1) (LOC440396), non-coding RNA [NR_002943]	**-1.142**
NSBP1	Homo sapiens nucleosomal binding protein 1 (NSBP1), mRNA [NM_030763]	**-1.141**
LOC147804	Homo sapiens tropomyosin 3 pseudogene (LOC147804), non-coding RNA [NR_003148]	**-1.140**
LRRC37A3	Homo sapiens leucine rich repeat containing 37, member A3 (LRRC37A3), mRNA [NM_199340]	**-1.138**
TTC14	Homo sapiens tetratricopeptide repeat domain 14 (TTC14), mRNA [NM_001042601]	**-1.137**
ZC3H7B	Homo sapiens zinc finger CCCH-type containing 7B (ZC3H7B), mRNA [NM_017590]	**-1.137**
ANXA2P3	Homo sapiens annexin A2 pseudogene 3 (ANXA2P3), non-coding RNA [NR_001446]	**-1.136**
LOC100130288	Homo sapiens cDNA clone IMAGE:5295205, with apparent retained intron. [BC043212]	**-1.135**
ZFP36L2	Homo sapiens zinc finger protein 36, C3H type-like 2 (ZFP36L2), mRNA [NM_006887]	**-1.131**
ISL1	Homo sapiens ISL LIM homeobox 1 (ISL1), mRNA [NM_002202]	**-1.127**

LOC392425	Homo sapiens hypothetical LOC392425 (LOC392425), mRNA [XM_001714831]	**-1.126**
RNF214	Homo sapiens ring finger protein 214 (RNF214), mRNA [NM_001077239]	**-1.125**
BPNT1	Homo sapiens 3'(2'), 5'-bisphosphate nucleotidase 1 (BPNT1), mRNA [NM_006085]	**-1.124**
SNRPC	Homo sapiens small nuclear ribonucleoprotein polypeptide C (SNRPC), mRNA [NM_003093]	**-1.124**
C15orf37	Homo sapiens chromosome 15 open reading frame 37 (C15orf37), mRNA [NM_175898]	**-1.123**
FLJ31813	Homo sapiens cDNA FLJ31813 fis, clone NT2RI2009517. [AK056375]	**-1.123**
NAP1L1	Homo sapiens nucleosome assembly protein 1-like 1 (NAP1L1), mRNA [NM_004537]	**-1.119**
DFFB	Homo sapiens DNA fragmentation factor, 40kDa, beta polypeptide (caspase-activated DNase) (DFFB), mRNA [NM_004402]	**-1.118**
LOC401602	Homo sapiens misc_RNA (LOC401602), miscRNA [XR_018322]	**-1.117**
PUS7L	Homo sapiens pseudouridylate synthase 7 homolog (S. cerevisiae)-like (PUS7L), mRNA [NM_031292]	**-1.111**
FLJ11292	Homo sapiens hypothetical protein FLJ11292, mRNA (cDNA clone MGC:151043 IMAGE:40125985). [BC117434]	**-1.111**
TUBA8	Homo sapiens tubulin, alpha 8 (TUBA8), mRNA [NM_018943]	**-1.110**
QKI	Homo sapiens quaking homolog, KH domain RNA binding (mouse) (QKI), mRNA [NM_006775]	**-1.106**
LOC100125556	Homo sapiens family with sequence similarity 86, member A pseudogene (LOC100125556), non-coding RNA [NR_024251]	**-1.105**
LOC400013	Homo sapiens misc_RNA (LOC400013), miscRNA [XR_019347]	**-1.104**
GALNT4	Homo sapiens UDP-N-acetyl-alpha-D-galactosamine:polypeptide N-acetyl-galactosaminyltransferase 4 (GalNAc-T4), mRNA [NM_003774]	**-1.103**
GARS	Homo sapiens glycyl-tRNA synthetase (GARS), mRNA [NM_002047]	**-1.100**
ZNF251	Homo sapiens zinc finger protein 251 (ZNF251), mRNA [NM_138367]	**-1.097**
LOC391160	Homo sapiens misc_RNA (LOC391160), miscRNA [XR_018489]	**-1.096**
GOLIM4	Homo sapiens golgi integral membrane protein 4 (GOLIM4), mRNA [NM_014498]	**-1.087**
ZNF117	Homo sapiens zinc finger protein 117 (ZNF117), mRNA [NM_015852]	**-1.087**
ABHD10	Homo sapiens abhydrolase domain containing 10 (ABHD10), mRNA [NM_018394]	**-1.084**
HSPA9	Homo sapiens heat shock 70kDa protein 9 (mortalin) (HSPA9), nuclear gene encoding mitochondrial protein, mRNA [NM_004134]	**-1.084**
LOC100129720	Full-length cDNA clone CS0DI044YN21 of Placenta Cot 25-normalized of Homo sapiens (human). [CR619772]	**-1.084**
ZNF107	Homo sapiens zinc finger protein 107 (ZNF107), mRNA [NM_016220]	**-1.084**
IRAK1BP1	Homo sapiens interleukin-1 receptor-associated kinase 1 binding protein 1 (IRAK1BP1), mRNA [NM_001010844]	**-1.080**
TMC8	Homo sapiens transmembrane channel-like 8 (TMC8), mRNA [NM_152468]	**-1.079**

RPL24	Homo sapiens ribosomal protein L24 (RPL24), mRNA [NM_000986]	**-1.077**
CUL2	Homo sapiens cullin 2 (CUL2), mRNA [NM_003591]	**-1.074**
RHOBTB3	Homo sapiens Rho-related BTB domain containing 3 (RHOBTB3), mRNA [NM_014899]	**-1.073**
TMEM56	Homo sapiens transmembrane protein 56 (TMEM56), mRNA [NM_152487]	**-1.067**
LOC285216	Homo sapiens cDNA FLJ34909 fis, clone NT2RI2009301, moderately similar to Biofunctional methylenetetrahydroflate dehydrogenase/cyclohydrofolate, mitochondrial precursor. [AK092228]	**-1.066**
LOC220429	Homo sapiens CTAGE family, member 5 pseudogene, mRNA (cDNA clone IMAGE:5270026). [BC030655]	**-1.065**
SNRPN	Homo sapiens clone Rt-16 SNURF-SNRPN mRNA, downstream untranslated exons, alternatively spliced. [AF400500]	**-1.063**
ZNF813	Homo sapiens zinc finger protein 813 (ZNF813), mRNA [NM_001004301]	**-1.062**
FLYWCH2	Homo sapiens FLYWCH family member 2 (FLYWCH2), mRNA [NM_138439]	**-1.061**
ACTR2	Homo sapiens ARP2 actin-related protein 2 homolog (yeast) (ACTR2), mRNA [NM_001005386]	**-1.060**
DDX6	Homo sapiens DEAD (Asp-Glu-Ala-Asp) box polypeptide 6 (DDX6), mRNA [NM_004397]	**-1.060**
ZNF594	Homo sapiens zinc finger protein 594 (ZNF594), mRNA [NM_032530]	**-1.058**
NCRNA00081	Homo sapiens non-protein coding RNA 81 (NCRNA00081), non-coding RNA [NR_024140]	**-1.052**
LOC729779	Homo sapiens misc_RNA (LOC729779), miscRNA [XR_016024]	**-1.052**
TBX15	Homo sapiens T-box 15 (TBX15), mRNA [NM_152380]	**-1.052**
CCDC68	Homo sapiens coiled-coil domain containing 68 (CCDC68), mRNA [NM_025214]	**-1.051**
LOC100132816	Homo sapiens hypothetical protein LOC100132816 (LOC100132816), mRNA [XM_001718703]	**-1.051**
LOC344332	Homo sapiens similar to hCG1812048 (LOC344332), mRNA [XM_001125944]	**-1.050**
LOC388621	Homo sapiens hypothetical LOC388621 (LOC388621), mRNA [XM_001725488]	**-1.050**
SULT1E1	Homo sapiens sulfotransferase family 1E, estrogen-preferring, member 1 (SULT1E1), mRNA [NM_005420]	**-1.049**
ZNF254	Homo sapiens zinc finger protein 254 (ZNF254), mRNA [NM_203282]	**-1.049**
LOC100133154	Homo sapiens hypothetical protein LOC100133154 (LOC100133154), mRNA [XM_001714925]	**-1.048**
LOC645884	Homo sapiens cDNA FLJ25739 fis, clone TST05834. [AK098605]	**-1.048**
LOC100127983	Homo sapiens hypothetical protein LOC100127983 (LOC100127983), mRNA [XM_001722571]	**-1.047**
FGFR1OP	Homo sapiens FGFR1 oncogene partner (FGFR1OP), mRNA [NM_007045]	**-1.047**
LOC400657	Homo sapiens hypothetical LOC400657 (LOC400657), non-coding RNA [NR_024484]	**-1.047**
CKAP4	Homo sapiens cytoskeleton-associated protein 4 (CKAP4), mRNA [NM_006825]	**-1.046**

SAMHD1	Homo sapiens SAM domain and HD domain 1 (SAMHD1), mRNA [NM_015474]	**-1.041**
LOC730232	Homo sapiens misc_RNA (LOC730232), miscRNA [XR_016075]	**-1.037**
ATP5E	Homo sapiens ATP synthase, H+ transporting, mitochondrial F1 complex, epsilon subunit (ATP5E), nuclear gene encoding mitochondrial protein, mRNA [NM_006886]	**-1.037**
KRT18P46	Homo sapiens keratin 18 pseudogene 46 (KRT18P46), mRNA [XM_001720107]	**-1.036**
ZNF431	Homo sapiens zinc finger protein 431 (ZNF431), mRNA [NM_133473]	**-1.035**
LOC100133528	Homo sapiens similar to HIG1 domain family, member 1A (LOC100133528), mRNA [XM_001721514]	**-1.033**
LOC648795	Homo sapiens hypothetical LOC648795 (LOC648795), mRNA [XM_001717954]	**-1.032**
TRPM6	Homo sapiens transient receptor potential cation channel, subfamily M, member 6 (TRPM6), mRNA [NM_017662]	**-1.032**
ZNF525	Homo sapiens zinc finger protein 525 (ZNF525), non-coding RNA [NR_003699]	**-1.032**
CHMP4C	Homo sapiens chromatin modifying protein 4C (CHMP4C), mRNA [NM_152284]	**-1.030**
KRT19P2	Homo sapiens mRNA for keratin 19, partial cds, isolate:K19-141. [AB041269]	**-1.030**
EID2	Homo sapiens EP300 interacting inhibitor of differentiation 2 (EID2), mRNA [NM_153232]	**-1.029**
RNF215	Homo sapiens ring finger protein 215 (RNF215), mRNA [NM_001017981]	**-1.029**
C15orf40	Homo sapiens chromosome 15 open reading frame 40 (C15orf40), mRNA [NM_144597]	**-1.029**
POLI	Homo sapiens polymerase (DNA directed) iota (POLI), mRNA [NM_007195]	**-1.028**
CPSF2	Homo sapiens cleavage and polyadenylation specific factor 2, 100kDa (CPSF2), mRNA [NM_017437]	**-1.027**
LOC441383	Homo sapiens hypothetical gene supported by AF086559; BC065734, mRNA (cDNA clone IMAGE:30352956). [BC065734]	**-1.026**
NNT	NAD(P) transhydrogenase, mitochondrial Precursor (EC 1.6.1.2)(Nicotinamide nucleotide transhydrogenase) [Source:UniProtKB/Swiss-Prot;Acc:Q13423] [ENST00000264663]	**-1.026**
SRP19	Homo sapiens signal recognition particle 19kDa (SRP19), mRNA [NM_003135]	**-1.026**
AP4S1	Homo sapiens adaptor-related protein complex 4, sigma 1 subunit (AP4S1), mRNA [NM_001128126]	**-1.021**
C8orf38	Homo sapiens cDNA FLJ23887 fis, clone LNG14332. [AK074467]	**-1.019**
ZNF155	Homo sapiens zinc finger protein 155 (ZNF155), mRNA [NM_003445]	**-1.019**
IFT74	Homo sapiens intraflagellar transport 74 homolog (Chlamydomonas) (IFT74), mRNA [NM_025103]	**-1.015**
SPAG1	Homo sapiens sperm associated antigen 1 (SPAG1), mRNA [NM_003114]	**-1.013**
LOC727835	Homo sapiens misc_RNA (LOC727835), miscRNA [XR_015767]	**-1.010**
C12orf24	Homo sapiens chromosome 12 open reading frame 24 (C12orf24), mRNA [NM_013300]	**-1.005**
LOC284230	Homo sapiens similar to mCG7611 (LOC284230), mRNA [XM_208185]	**-1.005**

PXMP2	Homo sapiens peroxisomal membrane protein 2, 22kDa, mRNA (cDNA clone IMAGE:4098463). [BC009836]	**-1.003**
CCDC74B	Homo sapiens coiled-coil domain containing 74B (CCDC74B), mRNA [NM_207310]	**-1.003**
