# Supplementary material for: Genotoxicity and molecular response of silver nanoparticle (NP)-based hydrogel
Source: J Nanobiotechnology. 2012 May 1;10:16. doi: 10.1186/1477-3155-10-16 (PMC3430588; doi:10.1186/1477-3155-10-16)
Supplement: Additional file 15 — Down-regulated genes related to mitosis pathway at silver-NP-hydrogel treatment for 48h. [file 1477-3155-10-16-S15.pdf]

Additional File 15. Down-regulated genes related to mitosis pathway at silver-NP-hydrogel treatment for 48h.

**No.**	**Gene Name**	**Gene ID**	**Description**
1	CDC14A	NM_003672	CDC14 cell division cycle 14 homolog A
2	FAM179A	NM_199280	family with sequence similarity 179, member A
3	KIF18B	BC048263	hypothetical protein LOC146909
4	IQGAP2	NM_006633	IQ motif containing GTPase activating protein 2
5	CHL1	NM_006614	cell adhesion molecule with close homolog of L1
6	MYLK2	NM_033118	myosin light chain kinase 2
7	CCNA1	NM_003914	cyclin A1
8	RASGRP1	NM_005739	RAS guanyl releasing protein 1
9	PTTG3	NR_002734	pituitary tumor-transforming 3
10	CIT	NM_007174	citron (rho-interacting, serine/threonine kinase 21)
11	SEPT13	NR_024271	septin 13 (SEPT13), non-coding RNA
12	NOS1	NM_000620	nitric oxide synthase 1 (neuronal)
13	RPS6KB1	NM_003161	ribosomal protein S6 kinase, 70kDa, polypeptide 1
14	POLI	NM_007195	polymerase (DNA directed) iota
15	TUBA8	NM_018943	tubulin, alpha 8
16	ZFP36L2	NM_006887	zinc finger protein 36, C3H type-like 2
17	PRPF4B	NM_003913	PRP4 pre-mRNA processing factor 4 homolog B
18	DIS3	NM_014953	DIS3 mitotic control homolog (S. cerevisiae)
19	UBE2V2	NM_003350	ubiquitin-conjugating enzyme E2 variant 2
20	SASS6	NM_194292	spindle assembly 6 homolog (C. elegans) A
21	ACTR2	NM_001005386	ARP2 actin-related protein 2 homolog (yeast)
22	CUL2	NM_003591	cullin 2A
23	RINT1	NM_021930	RAD50 interactor 1
24	ZC3H13	NM_015070	zinc finger CCCH-type containing 13
25	CCNJ	NM_019084	cyclin J
26	ZCCHC11	NM_001009881	zinc finger, CCHC domain containing 11
27	CENPF	NM_016343	centromere protein F, 350/400ka (mitosin)
