# Supplementary material for: Genotoxicity and molecular response of silver nanoparticle (NP)-based hydrogel
Source: J Nanobiotechnology. 2012 May 1;10:16. doi: 10.1186/1477-3155-10-16 (PMC3430588; doi:10.1186/1477-3155-10-16)

Additional figure 1. The size and size distribution of the silver nanoparticles determined by transmission electron microscopy (TEM). A: X 10000, bar = 200 nm; B: X 20000, bar = 100 nm.

A

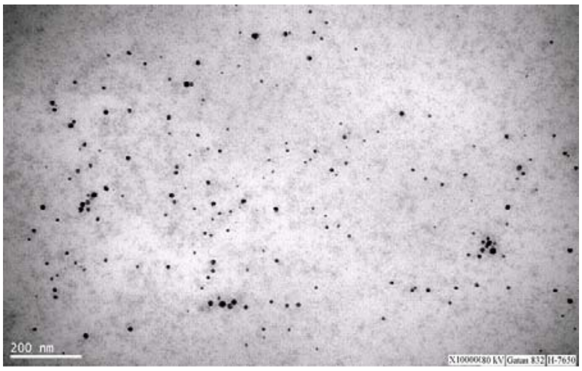

B

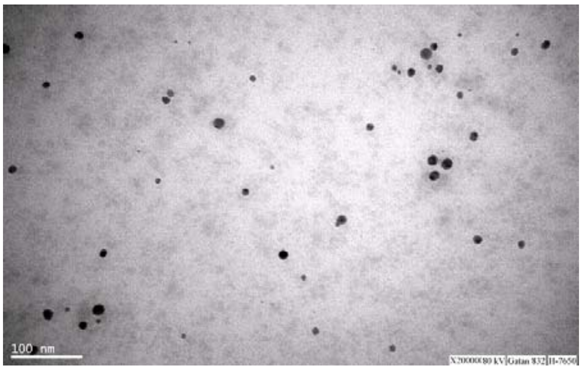

Supplement: Additional file 16. Figure S1 — The size and size distribution of the silver nanoparticles determined by transmission electron microscopy (TEM). A: X 10000, bar = 200 nm; B: X 20000, bar = 100 nm. [file 1477-3155-10-16-S16.pdf]
